# Patterns and Constraints in the Evolution of Sperm Individualization Genes in Insects, with an Emphasis on Beetles

**DOI:** 10.3390/genes10100776

**Published:** 2019-10-04

**Authors:** Helena I. Vizán-Rico, Christoph Mayer, Malte Petersen, Duane D. McKenna, Xin Zhou, Jesús Gómez-Zurita

**Affiliations:** 1 Animal Biodiversity and Evolution, Institute of Evolutionary Biology (CSIC-Universitat Pompeu Fabra), 08003 Barcelona, Spain; helena.vizan@ibe.upf-csic.es; 2 Center for Molecular Biodiversity Research, Zoological Research Museum Alexander Koenig, 53113 Bonn, Germany; c.mayer.zfmk@uni-bonn.de (C.M.); malte.petersen@senckenberg.de (M.P.); 3 Center for Biodiversity Research, Department of Biological Sciences, University of Memphis, Memphis, TN 38152, USA; dmckenna@memphis.edu; 4 Department of Entomology, College of Plant Protection, China Agricultural University, Beijing 100193, China; xinzhoucaddis@icloud.com

**Keywords:** Coleoptera, evolutionary rates, gene network, Insecta, phylogenetic inference, sex-biased genes

## Abstract

Gene expression profiles can change dramatically between sexes and sex bias may contribute specific macroevolutionary dynamics for sex-biased genes. However, these dynamics are poorly understood at large evolutionary scales due to the paucity of studies that have assessed orthology and functional homology for sex-biased genes and the pleiotropic effects possibly constraining their evolutionary potential. Here, we explore the correlation of sex-biased expression with macroevolutionary processes that are associated with sex-biased genes, including duplications and accelerated evolutionary rates. Specifically, we examined these traits in a group of 44 genes that orchestrate sperm individualization during spermatogenesis, with both unbiased and sex-biased expression. We studied these genes in the broad evolutionary framework of the Insecta, with a particular focus on beetles (order Coleoptera). We studied data mined from 119 insect genomes, including 6 beetle models, and from 19 additional beetle transcriptomes. For the subset of physically and/or genetically interacting proteins, we also analyzed how their network structure may condition the mode of gene evolution. The collection of genes was highly heterogeneous in duplication status, evolutionary rates, and rate stability, but there was statistical evidence for sex bias correlated with faster evolutionary rates, consistent with theoretical predictions. Faster rates were also correlated with clocklike (insect amino acids) and non-clocklike (beetle nucleotides) substitution patterns in these genes. Statistical associations (higher rates for central nodes) or lack thereof (centrality of duplicated genes) were in contrast to some current evolutionary hypotheses, highlighting the need for more research on these topics.

## 1. Introduction

Phenotypic and physiological differences among closely related species with highly similar genomes are expected to be the result of differences in the expression profiles of key genes (e.g., [1]). In this regard, understanding the mechanisms underlying differences between males and females of the same species becomes of particular interest. Conspecific individuals of different sexes share most, if not all, of their genome and genetics but sometimes display striking anatomical and physiological differences. Studies using model organisms have demonstrated the existence of significant differences in gene expression profiles between sexes. For example, approximately 30% of genes in the vinegar fly (order Diptera), *Drosophila melanogaster*, show sex-biased expression, and most of these genes are specific to reproductive tissues [2,3,4]. In fact, it has been proposed that most gene expression in *Drosophila* is sex biased at some point, exhibiting this bias either throughout the life cycle or in specific developmental stages [5]. Similarly, 5–15% of the genes in the mosquito (order Diptera) *Anopheles gambiae* genome show differential expression between males and females [6], and approximately 20% of the X-chromosome genes of *Tribolium castaneum* (order Coleoptera) are regulated differently in each sex [7].

The existence and need for biases in gene expression imply several evolutionary mechanisms that, on the one hand, allow for the bias to occur and, on the other hand, condition the dynamics of changes in the affected genes through time [8]. Sex bias in gene expression can be achieved through linkage to sex chromosomes and dosage compensation, sex-specific alternative splicing, and other mechanisms [9,10,11,12]. However, these mechanisms primarily affect the expression of regulatory elements, which in turn condition the action of the genes themselves, e.g., following a particular sex-specific splicing or protein maturation pathway. Gene duplication is another mechanism that directly allows for new gene expression profiles, including sex-biased ones [13]. Gene duplication offers an immediate solution to differential expression needs by potentially allowing each copy of a gene to acquire unique functionality. It is now viewed as having played an important, if poorly understood, role in the evolution of sex-biased gene expression [14]. Moreover, gene duplication could also be related, in part, to the relaxation of evolutionary constraints on one of the resulting gene copies, which could, in turn, lead to more rapid gene evolution [15]. Rapid gene evolution has classically been proposed as a consequence of sex-biased and particularly male-biased genes [4,5,16,17,18]. However, it is not entirely clear whether it is the bias in expression that results in faster evolutionary rates or if it is because of other features of these genes, such as their frequent tissue specificity, which is also correlated with faster evolutionary rates [19].

It is generally accepted that gene duplication is a major force altering the diversity and characteristics of sex-biased genes, but the connection between sex-biased gene expression and evolutionary rates remains poorly understood [8]. So far, these associations have been studied in just a handful of model organisms, and even though it is theoretically plausible that evolutionary processes and functional patterns are related, it is too early to invoke a general rule. Working toward this generalization first requires determining the unequivocal orthology of sex-biased genes between model and non-model species [20]. Furthermore, it requires assuming that orthology and structural homology correlate with functional homology [21,22]. Another problem lies in the actual definition of sex-biased genes. The concept is intuitive and unambiguous: a sex-biased gene is one with different levels of expression between males and females [23]. However, it is also a quantitative one: how different do the expression levels have to be to elicit the activation of the particular evolutionary mechanisms mentioned above? Other non-trivial issues include the occurrence of pleiotropy, the fact that sex-biased genes may be expressed for alternative functions in different tissues and not necessarily related or restricted to one sex, and protein–protein interactions, so that a specific function takes place through physical and genetic modulation by other proteins. Pleiotropy and protein–protein interactions could modulate or limit the evolutionary dynamics of genes, obscuring or changing the expectations derived from the study of model species.

In this study, we aimed to explore the correlation of sex-biased expression with gene duplications and accelerated evolutionary rates in a large evolutionary framework, using non-model organisms for which no gene expression analyses are available. Our work was informed by previous studies involving a model organism (*D. melanogaster*) and used phylogenetic approaches. The obvious candidates for sex-biased genes are those involved in processes that are exclusive to one sex, for example, spermatogenesis in males [18,24]. Thus, in order to test for these differences, we selected a male reproduction functional group, i.e., a coherent set of genes working together toward a specific reproductive function in males, including genes that are male biased in *Drosophila* spp. and genes that are expressed both in female and in male tissues or non-reproductive tissues. In particular, the present study focused on an integrated male reproductive function—sperm individualization—which is known to involve the action of both constitutive and sex-biased genes in *D. melanogaster* with different degrees of tissue specificity. Sperm individualization is one of the final stages in spermatogenesis that resolves spermatids as individual cells from the syncytial male germline cysts [25]. In a very simplified manner, this process involves a number of stages where (1) a syncytial cyst forms around all spermatids resulting from a primary spermatocyte, (2) an individualization complex formed through cytoskeletal mechanisms and membrane formation encapsulates each of the spermatids, and (3) the syncytial cytoplasm is discarded [26]. We investigated the phylogeny and evolution of these genes across the class Insecta, with particular emphasis on the species-rich order Coleoptera (beetles). The insects we studied included several model organisms for which both orthology assessment and expression studies were publicly available (e.g., modENCODE and OrthoDB projects; [27,28]). Given that beetles are proportionally underrepresented in the genomic and gene profiling literature, we mined relevant data from the 1KITE project (http://1kite.org/), thereby broadening representation of beetles in our study and facilitating orthology assessment via phylogenetic approaches [29].

## 2. Materials and Methods

### 2.1. Selection of Functional Group and Expression Profiles

**Figure 1:**
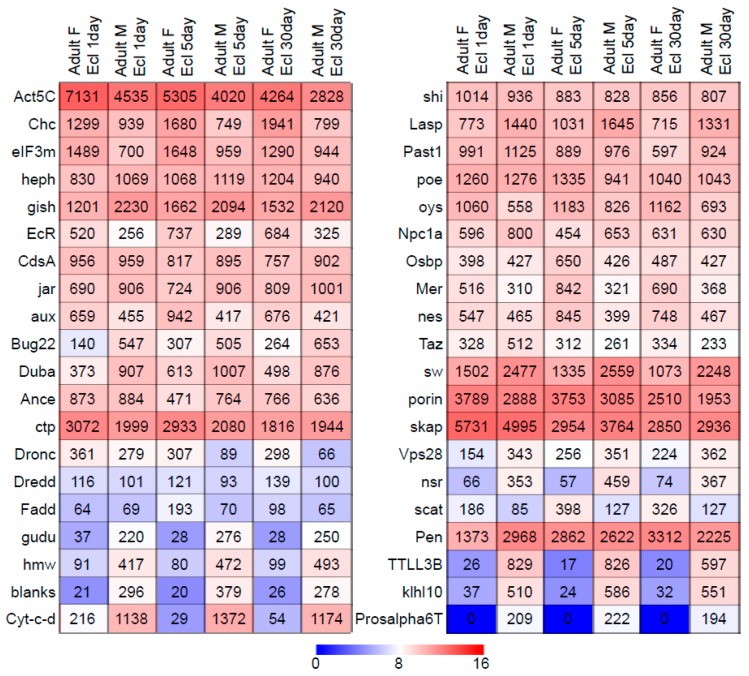
Heatmap visualization of gene expression scores (log_2_ of the actual value) of sperm individualization genes in *Drosophila melanogaster* as derived from RNA-seq data from different stages of adult male and female flies [27].

The gene browser AmiGO2 [30] was used to search for genes belonging to the gene ontology category “sperm individualization” (GO:0007291), a category that comprises all genes recognized to participate in the aforementioned processes. With this query, we obtained 54 genes, of which 1 was reported only for mammals (*Spem1*) and was not further considered, and the remaining 53 genes had been previously characterized in *Drosophila melanogaster*. The DNA coding sequences (CDSs) of these genes were retrieved (in September 2017) from FlyBase [31]. A preliminary *blastx* default search was conducted using these CDSs as query sequences, revealing that nine of these genes lacked obvious putative homologs in organisms other than Diptera. These genes (*dj*, *dud*, *fan*, *mst101(3)*, *nkg*, *ntc*, *soti*, *TTLL3B*, and *yuri*; named based on *Drosophila* gene nomenclature) were excluded from subsequent analyses. The remaining 44 genes (Table 1) were retained for use in our phylogenetic study and were functionally categorized as (i) unbiased or (ii) sex biased, according to their expression profiles in *Drosophila* using data publicly available in modENCODE [27]. These expression profiles were mined from Affymetrix tiling arrays (Figure 1), designed to study transcription levels in a large number of *Drosophila* cell lines and developmental stages, using modMINE [32]. When the expression profiles of males were less than twofold higher or not more than twofold lower than those measured in females, they were not considered indicative of being biased (a criterion applied in previous studies; e.g., [17]). Five of the genes of interest (*Cul3*, *Dark*, *didum*, *mlt*, and *orb2*) lacked data in the Affymetrix tiling array experiments, and we deduced their sex-based functional profile based on RNA-seq transcriptome profiles available in modENCODE [27].

### 2.2. Retrieval of Sperm Individualization Gene Orthologs in Insects

The FlyBase IDs for the 44 genes of interest were used as queries to find putative orthologs and their corresponding eukaryotic orthologous group (EOG) identifiers in OrthoDB v9.1 [33]. We retrieved all insect amino acid sequences for each EOG from the database, together with descriptive information about the number of hits and taxonomic redundancy, as well as data on the relative amino acid sequence divergence of each orthologous group as a proxy for the evolutionary rate in each EOG [28]. 

The representation of Coleoptera in OrthoDB is currently restricted to six species of three infraorders of the suborder Polyphaga (Table 2). In order to increase the representation of Coleoptera in the sample, we mined the genes of interest from transcriptomic data from beetle species available from 1KITE. The species studied included representatives from all four suborders of Coleoptera (Table 2). Moreover, we also searched for these genes in published RNA-seq data from testis of *Calligrapha multipunctata* (Chrysomelidae), which we expected to be enriched in sperm individualization genes [22]. In order to identify the 44 genes of interest in the assembled beetle transcriptomes, we used the software pipeline Orthograph version 0.5.14 [34]. This software predicts the orthology of nucleotide sequences by mapping their amino acid translation to genes of known ortholog groups using a graph-based best reciprocal hit approach. The pipeline also performs an automatic correction for sequence orientation, frameshifts, and translation. For all Orthograph searches, we used the official gene sets (OGSs) of three reference species: *D. melanogaster* (dmel_r6.11; http://flybase.org/, [35]); the red flour beetle, *Tribolium castaneum* (v3.0; http://beetlebase.org/, [36]); and the leaf-cutting ant, *Acromyrmex echinatior* (v3.8; http://hymenopteragenome.org/acromyrmex/, [37,38]). 

Each OGS included the 44 genes belonging to the EOGs of interest. Additionally, Orthograph required a tab-delimited file listing the name of the gene for each EOG and each reference species (obtained from OrthoDB). With this information, Orthograph retrieved from each OGS the genes of interest and aligned the amino acid sequences to create a profile hidden Markov model with which to conduct a forward search for respective candidate homologs in each of the beetle transcriptomes. The resulting hits were compared with a BLAST search against all genes in all OGSs (reverse search), and for each match between the best hit of the reverse search and the ortholog group of the original forward search, the corresponding transcript was assigned to that specific ortholog group [34]. Each Orthograph search produced the single best hit from each of the 1KITE transcriptomes mined for the study and generated separate files for each EOG, one with the original nucleotide data and one with their amino acid sequence translations, including the sequences of both the beetle targets and the reference species.

### 2.3. Phylogenetic Analyses of Amino Acid Sequences in Insects

Insect amino acid sequences from each EOG and those obtained from the output of Orthograph were aligned with the G-INS-i algorithm of MAFFT v7 [39]. Long autapomorphic insertions in these alignments, possibly corresponding to unrecognized introns, were trimmed manually, as were sequence ends of doubtful quality, typically showing as sequences unaligned beyond one point and longer than the remaining sequences in the alignment, suggesting that the reading frame had been lost and, therefore, the correct start or stop codons were not found either. In a few cases, the protein was retrieved from OrthoDB or the beetle transcripts as disjoint amino acid fragments coming from non-overlapping sequenced transcripts of the same gene. In these cases, the full protein length was reconstituted, and gaps between fragments were filled with missing data. Sequences were secondarily removed from the alignments if they (i) consisted of short fragments usually spanning less than 50% of the gene; (ii) were highly similar and monophyletic for a given species; and/or (iii) were highly divergent in the context of the variability of the alignment, the latter two features assessed based on preliminary phylogenetic analyses of the data.

The resulting purged alignments (deposited in Zenodo.org: 10.5281/zenodo.3380181) were analyzed using SMS [40] to identify the models of amino acid sequence evolution best fitting the data. The resulting models were used in maximum likelihood (ML) tree searches executed using the program PhyML v3.0 [41]. Since some of the genes of interest are multi-copy (in principle, OrthoDB identifies duplicated genes from isoforms resulting from alternative splicing), several gene alignments included many more sequences than taxa, and phylogenetic analyses allowed us to easily recognize when these extra sequences represented gene duplications affecting particular taxa or entire clades. In the former case, one representative of an intraspecific duplication was retained, and in the latter, duplicated versions of the gene were separated into independent alignments, which we realigned with MAFFT. Of the gene variants studied, the one including the sperm individualization gene copy in *Drosophila* was analyzed, assessing the best-fitting evolutionary model again with SMS. ML gene trees were inferred using PhyML, and statistical measures of nodal support were estimated via 100 bootstrap pseudoreplicates.

### 2.4. Phylogenetic Analyses of Nucleotide Sequences in Beetles

Nucleotide sequence matrices of the genes of interest for Coleoptera were generated by combining the sequences retrieved using Orthograph with the corresponding orthologs of model beetle species (Table 2). Data from model beetle species and from a hemipteroid (to be used as an outgroup in the analyses) were obtained with *blastn* searches against the nucleotide collection (nr/nt) at NCBI. The match of the retrieved nucleotide sequences with the amino acid sequence obtained from OrthoDB for the same organisms was confirmed with a subsequent *blastx* search against the reference proteins (refseq_protein) database, also at NCBI. Nucleotide sequences were aligned using the G-INS-i algorithm implemented in the program MAFFT. Low-quality ends were trimmed and short sequences removed, as above. The aligned sequences were also translated into amino acid sequences to assist the alignment by finding reading frame problems and highly divergent regions, which were secondarily removed. ML phylogenetic analyses were implemented using these aligned datasets and the same methods described above for the amino acid data.

### 2.5. Estimation of Evolutionary Rates

With very few exceptions, the ML gene trees based on amino acid sequences recovered Hymenoptera and Diptera each as monophyletic and usually with strong (typically 98–100%) bootstrap support. These two clades have particularly well-established age estimates based on independent analyses. They were used as calibration points in Bayesian analyses of evolutionary rates and node dating for each gene tree using the software BEAST v1.8.4 [42]. The nodes for these two clades were consistently constrained as monophyletic in all analyses to avoid uninformative topologies, particularly for genes with low phylogenetic signal, and the calibration densities for the time to their most recent ancestors were modeled as follows. For Hymenoptera, we specified a crown age of 309 Ma (291–347 Ma) after [43], approximately modeled in BEAST as a normal distribution with mean = 309 and Stdev = 10; in turn, the crown age of Diptera was assumed to be 265 Ma (256–269 Ma) according to [44] and approximately modeled as a normal distribution with mean = 265 and Stdev = 5. The analyses used substitution models as determined with SMS, an uncorrelated lognormal relaxed clock [45], and a tree prior under the Yule process. The analyses were run initially for 100 million generations, sampling every 10,000th generation, but in most cases, they had to be replicated and results combined until there was good mixing of parameters and all produced stable estimates with acceptably high effective sample sizes (ESS >> 200). In a few cases, typically involving datasets that clearly deviated from a molecular clock (i.e., value of ucld.stdev > 3), the multiple analyses produced erratic results; here, stable results were obtained using an exponential relaxed distribution. Evolutionary rates, as well as node ages, were calculated using Tracer 1.6 [46] on the annotated maximum clade credibility trees obtained by summarizing the post burn-in trees with LogCombiner 1.8.4 and TreeAnnotator 1.8.4 [42]. Nucleotide substitution rates in beetles were assessed using a similar strategy but with constraining the age of Coleoptera using a normal distribution covering the age range based on the estimate for this order as deduced from the previous analyses. Specifically, we extracted this age as the concordant overlap of all confidence intervals for this parameter in the amino-acid-based trees where Coleoptera was monophyletic.

### 2.6. Statistical Analyses

We tested the hypothesis of no differences in the evolutionary rates of sex-biased genes relative to unbiased genes using a Mann–Whitney *U* test [47] at a 0.05 significance level, as implemented in the function “wilcox.test” of the R package Stats 3.6.0 [48]. The same test was used to investigate rate differences between genes found as single-copy and as members of multigene families, as well as between genes coordinated in the gene cascade for sperm individualization versus genes participating in this function but not implicated in this interaction network (see below). Finally, genes were tested for differences in absolute evolutionary rates between two main categories based on the overall constancy of those evolutionary rates: genes with relatively homogeneous rates (parameter ucld.stdev < 0.6) and genes with heterogeneous rates (ucld.stdev > 0.6). These tests were conducted using substitution rates estimated from the insect amino acid data and substitution rates for beetles estimated from nucleotide data. In order to recognize possible interactions of the explanatory variables used in these tests, chi-squared permutation contingency tests of independence were run for each pair of categorical variables used to rank all genes, including expression bias, paralogy, network interaction, and rate heterogeneity. These tests used the “perm.ind.test” function of the R package wPerm 1.0.1 [49] with 9999 randomization replicates. In all tests, sample sizes allowed for low type I error rates, between 5% and 10% (Power = 0.80).

### 2.7. Analyses Constrained by Gene Interactions

Public databases were used to define the subset of physically or genetically interacting genes among those sharing sperm individualization as a unifying function. Specifically, we established the interaction network of *Drosophila melanogaster* as an interaction model by extracting the information about specific protein–protein physical interactions from BioGRID version 3.4 [50] and that about genetic interactions in metabolic pathways from FlyBase [31]. The obtained graph included 21 nodes (i.e., genes) and 28 edges (i.e., interactions), and the architecture of interactions was used to explore correlations with the evolutionary properties of this subset of genes and with other gene characteristics, including evolutionary rates, patterns of gene duplication, and sex-biased gene expression. Since these genes are not isolated in their function and their interactions, we also considered the total number of known interactions per gene, as shown in BioGRID, as a measure to modulate node importance. We tentatively corrected node importance in every case, calculating the logarithm of the product between node centrality and the absolute number of known interactions per node.

Statistical network analyses were carried out with the aid of R tools implemented in the “igraph” package [51] on the undirected connected graph representing all interacting genes. Measures of node centrality or node "importance" in the networks were obtained relative to the number of receiving edges (“closeness”) or their rank (“eigen_centrality”). We estimated the correlation between these variables and evolutionary rates and gene paralogy based on the Spearman rank-order correlation coefficients. Additionally, the network community structure was explored with several node modularity optimization algorithms in “igraph”, including the Clauset–Newman–Moore algorithm (command “cluster_fast_greedy”) and the Louvain method (command “cluster_louvain”; [52]), as well as exact modularity maximization (command “cluster_optimal”) using the algorithm published by [53]. Modularity was also estimated by considering edges instead of nodes and using the algorithm (command “cluster_edge_betweenness”) proposed by [54]. We tested for the existence of differences in evolutionary rates for each resulting group using the Kruskal–Wallis test [55]. Additionally, the homogeneity of rates between bipartitions of the network defined by each of the edges separating groups was investigated using a Mann–Whitney *U* test at a 0.05 significance level.

## 3. Results

### 3.1. Characteristics of Datasets: Composition of Sequence Alignments

The 44 investigated genes involved in sperm individualization (GO:0007291) were present in the subclass Pterygota (winged insects), both in Palaeoptera (mayflies and odonates), which were used as outgroups in all analyses, and Neoptera (the remaining orders of winged insects). Most of these genes showed unbiased patterns of gene expression in *Drosophila*, except for eight genes (Table 1). Given the lack of similar functional studies in most other insects, these eight genes represented our hypothesis for biased expression in the insect and beetle datasets. The median length of the associated proteins ranged from 89 amino acids in the case of *ctp* to 2949 amino acids in the case of *poe*, with an average of 638 ± 529 amino acids per protein.

**Figure 2:**
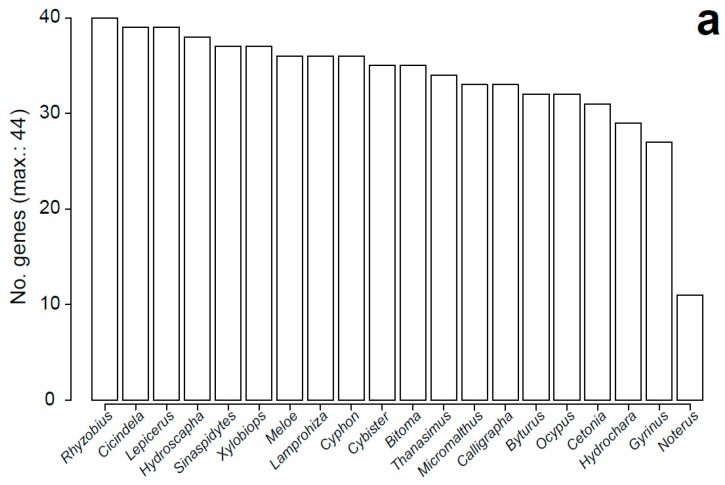

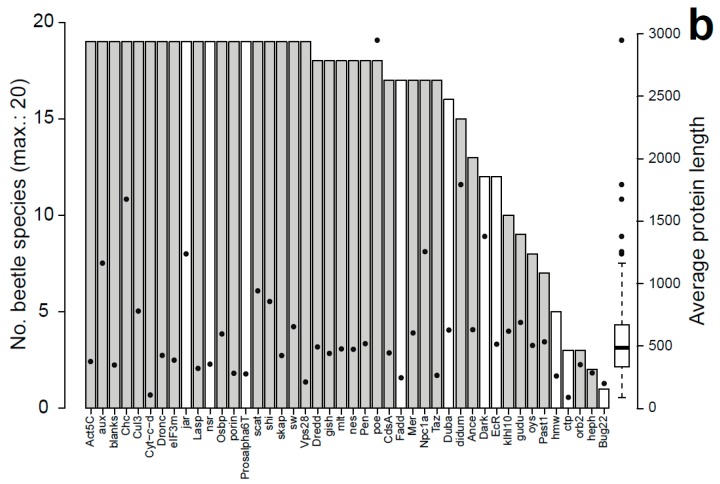
Performance of Orthograph searches of sperm individualization gene orthologs in beetle transcriptomes. (**a**) Number of genes retrieved from each of the non-model beetle species transcriptomes. (**b**) Number of beetle species yielding ortholog sequences for each of the sperm individualization genes analyzed, with information on the average protein length and showing genes absent in one or more OrthoDB beetle model species as white columns.

For most of the genes, OrthoDB contributed the amino acid sequences of the six beetle model species to the Coleoptera subset (Figure 2b). The only exceptions were *Bug22*, *ctp*, *Dark*, *EcR*, *Fadd*, *jar*, *nsr*, and *Prosalpha6T*, which lacked data for one of the species, *Duba* for two, and *hmw* for five. Mapping of orthologous genes using Orthograph from transcriptomes of a selection of 19 beetle species from the 1KITE Project and one testis-specific transcriptome from another beetle species resulted in positive hits in all cases, although with different success rates, possibly related to the quality or source of the transcriptomes. No single species yielded ortholog sequence data for all tested genes, with *Rhyzobius pseudopulcher* retrieving the highest number of genes (40 out of 44) and two water beetle species, *Gyrinus marinus* and *Noterus clavicornis*, retrieving the lowest (27 and 11 genes, respectively). For 70% of the beetle species, we retrieved at least 75% of the genes (Figure 2a). In turn, for all genes analyzed, we found orthologs in the beetle transcriptomes, but with different success rates (Figure 2b). A large proportion of genes (43.2%) were found in at least 19 out of 20 beetle species, and most of them (72.7%) were found in 15 or more beetle species. Conversely, eight genes could not be found in at least half of the species analyzed, with genes such as *hmw*, *ctp*, *orb2*, *heph*, and *Bug22*, showing the lowest recovery frequencies (*n* ≤ 5). The proteins encoded by these genes were shorter than the average but were also typically lacking recognized orthologs in some of the beetle model species (Figure 2b).

### 3.2. Characteristics of Datasets: Gene Duplications

**Figure 3:**
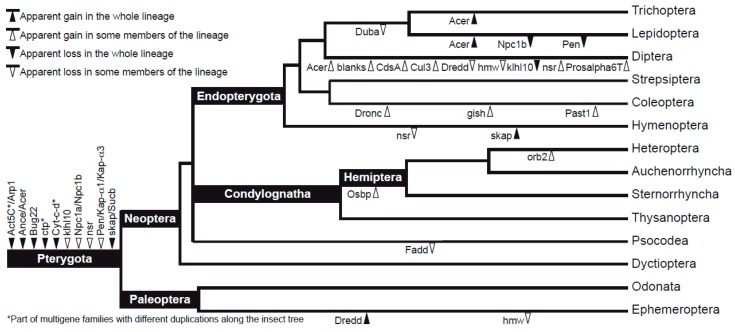
Schematic consensus phylogeny of insects (drawn from trees in [56,57,58]) showing the inferred evolutionary gains and losses of sperm individualization genes for major insect lineages.

At the time of this study, OrthoDB curated data for 119 insect species. When more than 119 sequences were retrieved for a particular gene, this informed in most cases of potential multi-copy genes (Table 3). Their actual presence was confirmed in the ML trees when including all the sequences retrieved from OrthoDB and the beetle sequences mined from 1KITE. In these cases, we used the annotation of the *D. melanogaster* sequence to recognize the sperm individualization paralog of interest. Figure 3 shows a diagram of gene duplications (and some secondary gene losses) as recognized in this study. 

For a total of 21 genes, we had no evidence for duplications or losses in the winged insect lineage. However, 18 genes showed duplications in Pterygota or parts of this evolutionary lineage. Two of these genes, *Act5C* and *Cyt-c-d*, were found as part of multigene families, and it was difficult to tell individual copies apart with the available data and as a result of their high similarity. *Act5C* is part of a gene complex with a deep split in all insects, including actin-related proteins (Arp1 in *Drosophila*) and several actins resulting from various duplications. We found evidence for at least six actin-like gene copies in Acalyptratae flies (fruit and peacock flies, among others), five in mosquitoes, four in Hymenoptera and Palaeoptera, at least three in Coleoptera and the hemipteroids, and at least two among Lepidoptera. *Act5C*, in particular, is highly conserved in the whole of Pterygota, and most of the available beetle sequences were retrieved close to this specific *Drosophila* paralog in the phylogeny. In turn, *Cyt-c-d* was revealed as a member of a multigene family in most insect groups, including odonates, some hemipteroids, beetles, and some dipterans. The proteins encoded by these genes are short and highly conserved, so paralogs could not be resolved easily, but most beetle sequences retrieved by Orthograph were more similar to the *Cyt-c-p* copy of the gene in *Drosophila*. Finally, *ctp* corresponded to a very short fragment, highly conserved and with evidence for paralogy, though it was not possible to discriminate gene copies. Given the difficulty of discerning orthologs, these three genes were not considered in downstream analyses. 

Seven of the duplicated genes—namely, *Ance*/*Acer*, *Bug22*, *klhl10*, *Npc1a*/*Npc1b*, *nsr*, *Pen/Kap-α1*/*Kap-α3*, and *skap*/*Sucb*—were duplicated in all studied insects and, in some cases, with one of the copies being subsequently lost or further multiplied in particular lineages. For example, orthologs of the *Npc1b* and *Pen* copies were lost in Lepidoptera, the sister copy of *klhl10* was lost in Diptera, and one copy of *nsr* was lost in aculeate Hymenoptera. Acalyptratae (Diptera) had three additional copies of *nsr* (four in *Bactrocera* tephritid peacock flies); *Acer* was duplicated independently in Trichoptera, Lepidoptera, and some Diptera; and *skap* had an additional copy among the Hymenoptera. Overall, 10 genes had lineage-specific duplications. *Dredd* had several copies in *Ephemera* alone; *orb2* and *Osbp* were duplicated in some hemipterans; and for *Dronc*, *gish*, and *Past1*, we found evidence for duplications in Coleoptera. Finally, the remaining four genes were duplicated in Diptera: *blanks* and *Cul3*, with fast-evolving copies in some dipterans; *CdsA* in some nematocerans (midges and moth-flies); and *Prosalpha6* in *Drosophila* alone (wherein only the paralog *Prosalpha6T*, perhaps missing in all the other insects, is male biased). Apart from the lineage-specific losses found for *Npc1b*, *Pen*, and the sister copies of *klhl10* and *nsr*, other gene losses detected in our data set affected *Dredd* (missing in mosquitoes [Diptera: Culicidae]), *Duba* (lacking in Trichoptera and Lepidoptera), *Fadd* (absent in some Hemiptera), and *hmw* (not recorded in *Ephemera* [Ephemeroptera] or *Anopheles* [Diptera]).

### 3.3. Evolutionary Rates of Sperm Individualization Genes in Insecta and Coleoptera

**Figure 4:**
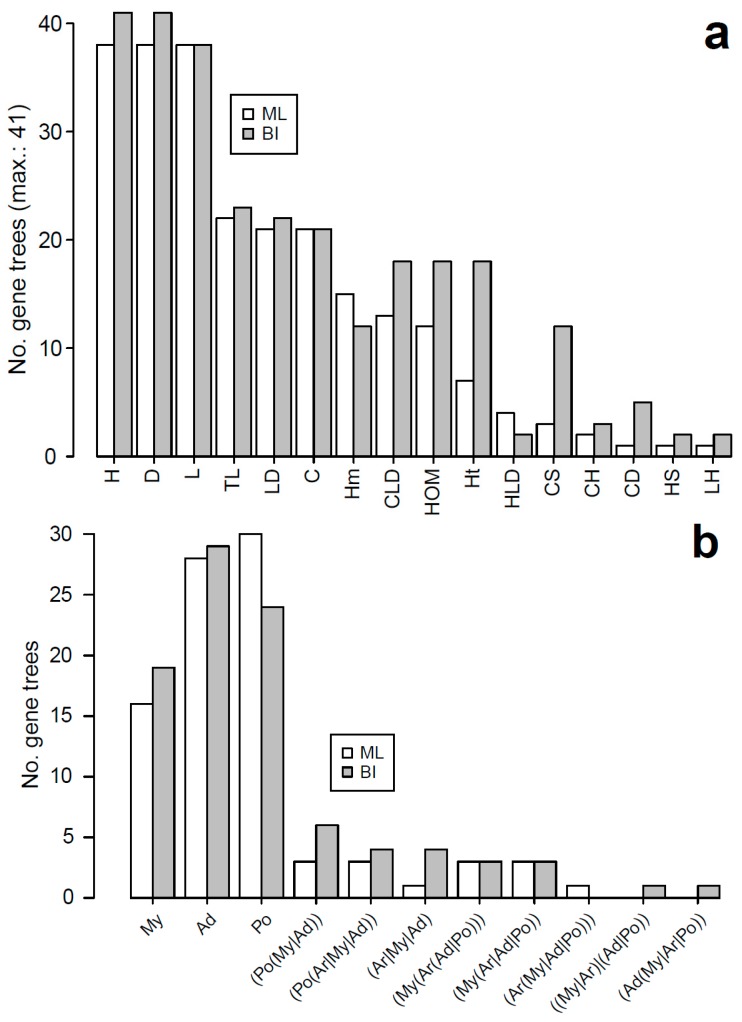
Frequency of maximum likelihood (ML) and Bayesian inference (BI) sperm individualization gene trees resolving particular higher taxa or relationships among them in insects (**a**) and beetles (**b**). Key: Ad, Adephaga; Ar, Archostemata; C, Coleoptera; D, Diptera; H, Hymenoptera; Hm, Hemiptera; HOM, Holometabola; Ht, Heteroptera; L, Lepidoptera; My, Myxophaga; Po, Polyphaga; S, Strepsiptera; T, Trichoptera.

Amino acid sequence matrices of the orthologous sperm individualization genes of insects and nucleotide sequence data of Coleoptera were used to infer gene trees under ML and Bayesian inference and to estimate evolutionary rates (Supplementary Files S1–S4). In general, both methods produced similar gene trees, e.g., with respect to resolving the relationships of the insect orders and some infraordinal relationships (Figure 4a), usually with relatively strong nodal support, and consistent with the current systematic knowledge for insects [56]. However, most trees had relatively poorly resolved deep relationships, particularly within the hemimetabolous insect orders, which were represented by relatively few taxa. In turn, in most beetle trees, the suborders represented by several species were retrieved as monophyletic, but there was no consensus among trees on subordinal relationships (Figure 4b). However, in most cases the topologies were consistent with Polyphaga being sister to the other three suborders (Adephaga, Myxophaga, and Archostemata).

Based on the previous phylogenies, the amino acid substitution rates for 41 proteins encoded by sperm individualization genes (excluding the 3 proteins for which orthology could not be confirmed) spanned nearly two orders of magnitude, from 0.000237 amino acid changes per lineage and million years (subs./l./Ma) in the protein orb2 to 0.009667 subs./l./Ma in the protein hmw (Table 4). The average substitution rate for the whole dataset was 0.00239 ± 0.003012 subs./l./Ma. Slightly over half (56%) of these proteins, typically those with lower overall substitution rates, exhibited evolutionary rates inconsistent with a molecular clock, i.e., rates on individual branches with more substantial departures from the mean (ucld.stdev ≥ 0.6).

**Figure 5:**
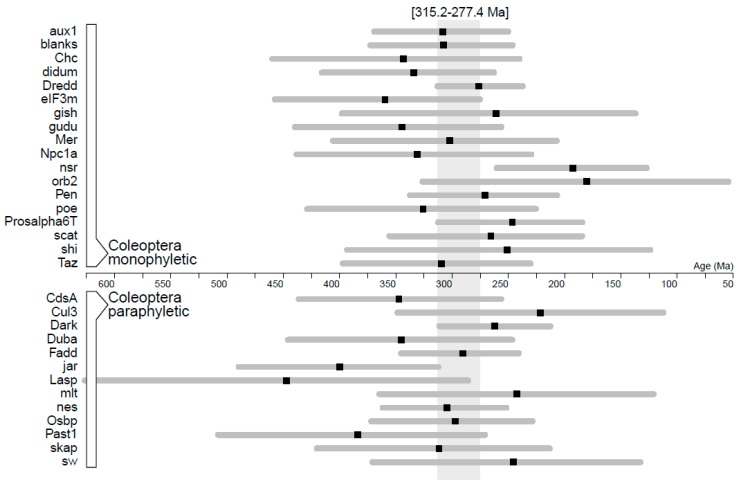
Inferred ages and 95% credibility intervals for the Coleoptera clade (top panel) or a Coleoptera + Strepsiptera clade (bottom panel) based on the molecular clock analyses of amino acid sequence data of sperm individualization genes. The full overlap of age estimates of monophyletic Coleoptera identifies an interval (shaded area) consistent with the proposed age of the group based on fossil data and used here as age prior for the evolutionary rate analyses in beetles.

The analyses of evolutionary rates yielded age estimates for the clade Coleoptera with averages ranging between 180.4 Ma, in the case of *orb2*, and 451.5 Ma, in the case of *Lasp*, with broad confidence intervals of 186.2 ± 62.49 Ma on average (Table 4). Coleoptera was recovered as monophyletic in 18 of the analyses, and the overlap of the age confidence intervals obtained for each gene covered a period between 277.4 and 315.2 Ma (except in the case of *nsr*, which yielded an age much younger than the oldest known beetle fossils) (Figure 5). This time interval was used to restrict the age of Coleoptera in subsequent analyses, and it was consistent with most clade age estimates for Coleoptera obtained in analyses where the beetle clade also included Strepsiptera.

The above time constraint for Coleoptera produced instantaneous nucleotide substitution rates ranging from 0.00208 subs./l./Ma in the case of the gene *nes* to 0.01190 subs./l./Ma in the case of *Cul3*, with an average substitution rate for the whole set of genes investigated of 0.00452 ± 0.002083 subs./l./Ma (Table 5). Slightly over half these genes had substitution rates relatively consistent with a molecular clock (ucld.stdev < 0.6), and in contrast to the case of the amino acid sequence analyses, the genes departing from the molecular clock were those with higher nucleotide substitution rates.

### 3.4. Analysis of Rate Differences

Permutation tests of independence produced non-significant results for every pair of independent variables used in subsequent tests, suggesting that there were no interactions among them. The null hypothesis that sperm individualization genes with or without duplications in the insect lineage had the same evolutionary rates was not rejected (Mann–Whitney *U* = 183, *p* = 0.758; also treating hemipteroid *orb2* and *Osbp* duplications as non-duplicated genes: *U* = 157, *p* = 0.497). Similarly, this hypothesis was not rejected in the case of genes working in coordination in a gene interaction network (like the one deduced for *Drosophila*) tested against genes dissociated from this network (Mann–Whitney *U* = 190, *p* = 0.632). However, when genes were split into two categories according to their predicted sex expression bias, or according to whether they evolved in a clocklike fashion, the null hypothesis of no differences in their evolutionary rates was rejected at the 0.05 significance level (Mann–Whitney *U* = 52, *p* = 0.019 and Mann–Whitney *U* = 323, *p* = 0.002, respectively). In these cases, sex-biased and clock-constrained genes would have slightly faster rates, except for the male-biased gene *hmw*, a fast-evolving protein departing nonetheless from a molecular clock. The same tests, when applied to nucleotide substitution rates of the genes of interest in beetles, produced non-significant results when rate differences were tested for predicted expression biases (*U* = 115, *p* = 0.817), gene duplications in the beetle lineage (*U* = 181, *p* = 0.551), or their predicted coordination in an interaction network (*U* = 156, *p* = 0.118). The test produced a clear significant result when rate differences were tested against the clocklike behavior of data (*U* = 5, *p* << 0.001), with the genes departing from the molecular clock having much higher rates (genes[ucld.stdev < 0.6]: 0.00314 ± 0.000533 versus genes[ucld.stdev ≥ 0.6]: 0.00620 ± 0.002030).

### 3.5. Evolutionary Patterns in the Sperm Individualization Interaction Network

**Figure 6:**
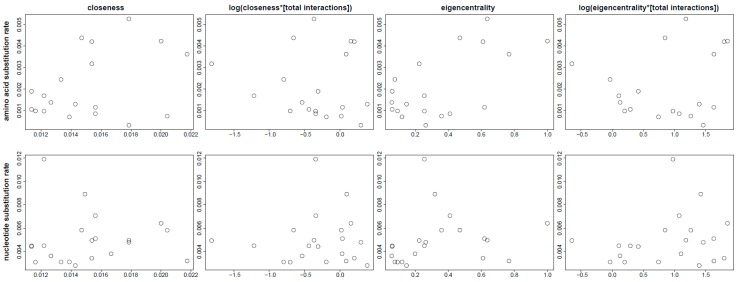
Biplots of the correlation between different measures of node importance in the interaction network of sperm individualization genes and their amino acid evolutionary rates in insects (top panels) and nucleotide substitution rates in beetles (bottom panels).

The results of the Spearman’s rank correlation tests between amino acid substitution rates and measures of node importance based on the number of receiving edges (S = 956.6, rho = 0.1609, *p* = 0.5106), rank (S = 712.6, rho = 0.3749, *p* = 0.1138), or their respective corrections considering the total number of genetic interactions of the nodes of interest (S[edges] = 1340.0, rho = −0.1754, *p* = 0.4709; S[rank] = 1054.0, rho = 0.0754, *p* = 0.7592) were all non-significant (Figure 6). Similarly, the correlations between nucleotide substitution rates in beetles and node centrality measures based on the number of receiving edges (S = 1115.3, rho = 0.2758, *p* = 0.2263) or their tentative correction based on edges (S = 1520, rho = 0.0130, *p* = 0.9573) and rank (S = 1180, rho = 0.2338, *p* = 0.3063) were non-significant. However, when node centrality was assessed based on the first eigenvector of the adjacency matrix, their correlation with nucleotide substitution rates was significant (S = 770.5, rho = 0.4997, *p* = 0.0211), suggesting a slight effect of more densely connected regions of the network having significantly higher evolutionary rates. In turn, there was no evidence for a correlation between genes being single copy or duplicated and any centrality measure without (edges: S = 1772.9, rho = −0.1512, *p* = 0.5129; rank: S = 2127.7, rho = −0.3816, *p* = 0.0878) or with correction (edges: S = 1368.7, rho = 0.1112, *p* = 0.6312; rank: S = 1980.5, rho = −0.2860, *p* = 0.2088).

**Figure 7:**
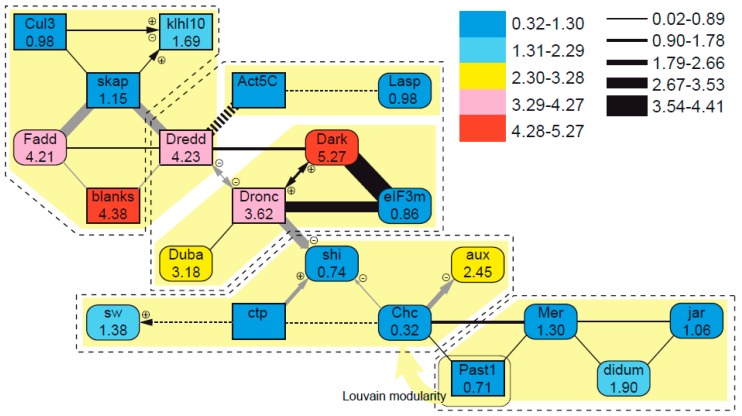
Mutual interaction network of sperm individualization genes in *Drosophila* including protein–protein (lines) and genetic/regulatory interactions (arrows), the latter with information on the enhancing and/or repressing modulation effects. Nodes represent interacting proteins, and they are color coded according to their inferred amino acid evolutionary rates. Edges represent documented interactions between proteins, with their width being proportional to the evolutionary rate differences between interacting proteins (dashed lines are used when their evolutionary rate data are missing). Dashed-line and solid-background polygons show the edge-based and node-based partitions of the network, respectively. More details and alternative partitioning schemes are described in the main text.

Network modularity measures split the gene interaction network into four groups when using edge-based partitioning or five groups when using node-based partitioning, with considerable agreement between strategies (Figure 7). Exact modularity and the Clauset–Newman–Moore algorithm produced identical groupings, differing from the edge-based solution in the transfer of one node (*Dredd*) to an adjacent group and the split of two nodes (*Act5C* and *Lasp*) as an additional group. The Louvain modularity produced groups identical to the other node-based methods, but transferring one node (*Past1*) into the adjacent group. None of these global partitioning strategies showed statistical differences in amino acid substitution rates (“edge_betweenness”, chi-sq = 2.8835, *p* = 0.4099; “optimal”, chi-sq = 2.5958, *p* = 0.6276; “louvain”, chi-sq = 2.4258, *p* = 0.6580) or nucleotide substitution rates (“edge_betweenness”, chi-sq = 4.9143, *p* = 0.1782; “optimal”, chi-sq = 5.4316, *p* = 0.2458; “louvain”, chi-sq = 4.9848, *p =* 0.2889). However, when different group bipartitions of the network were considered, the edge between *Dronc* and *shi* (ABC and DE clusters in Figure 7) delimited groups with different amino acid substitution rates (*U* = 72, *p* = 0.0279) and different nucleotide substitution rates when beetle data were considered both for edge (*U* = 83, *p* = 0.0409) and for node (*U* = 93, *p* = 0.0062) partitions. Nucleotide substitution rates were also statistically significantly different across the edges joining *Dredd* and *Dronc* (*U* = 80, *p* = 0.0200; AB and CDE clusters in Figure 7) and *Chc* and *Past1* (*U* = 69, *p* = 0.0147; ABCD and E clusters in Figure 7).

## 4. Discussion

### 4.1. Data Mining Genomic and Transcriptomic Resources: Sequence Quality

The results of studies exploiting genomic and transcriptomic resources depend on their quality and curatorial status, regardless of how complex and efficient the bioinformatic approaches used to extract this information are [57,58]. Usually, the scale and complexity of studies using “big data” prevent end-user control of their quality [59], and data may include unnoticed errors (e.g., incorrect taxonomic assignments or shifts in reading frames) or may have escaped objective quality filters (e.g., low sequence quality or assembly problems). Here, we used several public databases of annotated sequence data, including GenBank, FlyBase, modENCODE, OrthoDB, and BioGRID, as well as the partially released 1KITE database. Each may have contributed particular biases to the results, but the amount of data was still amenable to manual control of the different analytical steps, allowing for the recognition of problems and for hopefully avoiding them by iterative analytical exploration and filtering of the data.

The first challenge we had to address after mining the sequence data, and before all analyses, was filtering what we interpreted as noisy sequence data or suspicious annotations in the data sets. Sequence quality was a major concern when using data directly mined from sequence repositories. Thus, we identified, through iterative assessment, two main criteria for the total or partial removal of potentially noisy data. These were (i) long autapomorphic insertions in amino acid sequences which may result from unrecognized introns and (ii) highly divergent, unalignable regions, typically at the ends of sequences, due to compensated nucleotide gains/losses in that part of the sequence, locally affecting the reading frame. Reiterated multiple sequence alignments also allowed recomposing the proteins that appeared in OrthoDB as non-overlapping fragments for some taxa into a single sequence. However, when this situation affected duplicated genes, there was a risk of joining fragments of non-orthologous proteins, which we addressed by using phylogenetic trees to inform manual curation [29]. We gained additional insight into the aforementioned problems by merging annotated and curated amino acid sequence data from OrthoDB with translated nucleotide sequence data from 1KITE beetle transcriptomes. Some of the latter sequences showed precisely the same translation problems affecting homology as were found for the insect protein data, and they were filtered according to the same criteria specified above.

### 4.2. Data Mining Genomic and Transcriptomic Resources: Orthology Assessment

Orthology assessment was particularly crucial in the examination of beetle data mined directly from raw transcriptomes, and here, this assessment was particularly important because orthology provided our best hypothesis for conserved gene function. For most EOGs of interest for which we searched the transcriptomes, the pipeline yielded a phylogenetically cohesive group of potentially orthologous sequences with their paralogs when they were present in the transcriptome. The efficiency of Orthograph in this respect was demonstrated when mistakes were made. For example, a bad specification of the EOG corresponding to the gene *Pen* initially resulted in predicted beetle orthologs for one of the other importin-alpha genes in insects, which could be identified and corrected in our iterative phylogenetic approach. For six genes, however, the analyses picked up at least two paralogs. Two corresponded to *Act5C* and *Cyt-c-d*, which we already described as challenging to separate in the respective duplicated copies, even using phylogenies. The other four are more difficult to explain, and recognizing them required phylogenetically informed decisions; they were removed from the analyses a posteriori. Of these, two were genes for which we revealed duplications in beetles, *Dronc* and *gish* (for the latter, we found the beetle-specific paralog only in *Xylobiops* [Bostrichidae]). The other two were *Npc1a*, for which the correct sperm individualization ortholog was identified in 16 beetle transcriptomes and its paralog *Npc1b* in *Lepicerus* sp., and *klhl10*, for which the copy missing in Diptera was found in *Micromalthus debilis* and *Lamprohiza splendidula*. For all of these genes, we have strong evidence hinting at them being duplicated in the beetle genomes, yet we retrieved one of the copies in most species and the other copy in one or just a few transcriptomes. If these genes are indeed duplicated, the reason why both copies were not found consistently in all beetle transcriptomes may be related to how the program Orthograph works, i.e., retrieving a single best reciprocal hit, the putative ortholog. In these circumstances, and analogously to ranked results of BLAST searches, the correct, biologically meaningful sequence may be missed after yielding a suboptimal hit, perhaps because of sequence quality and/or length issues or the absence of the ortholog of interest in some of the transcriptomes.

### 4.3. Evolutionary Dynamics of Sperm Individualization Genes

All qualitative traits that were used to rank sperm individualization genes in insects were statistically independent. This implies that, at least for this subset of genes, some evolutionary predictions do not apply, including the association of sex-biased gene expression with an origin attributed to gene duplications [18]. Apart from duplications, we also recorded gene losses, because it has been hypothesized that the rate of turnover (i.e., lack of 1:1 orthology) for sex-biased—particularly male-biased—genes may be higher than for other genes [60,61]. Among sperm individualization genes, we found lineage-specific losses for both biased and unbiased genes without statistical differences between groups (chi-sq = 2.4529, *p* = 0.1450), and our phylogenetic analyses, in fact, show that preservation and genomic dosage of sperm individualization genes are generally highly conserved across the Insecta despite their long evolutionary history.

In our analysis of the correlation between the different ways in which we ranked sperm individualization genes and their inferred evolutionary rates, only two instances of statistically significant differences were obtained. The first relates to the overall homogeneity of substitution rates both for insect amino acid and for beetle nucleotide sequence data (even if with opposite signs). The second and most interesting, considering the deep evolutionary time considered and the assumption of conservation of gene functionality across this time scale, was for sex-biased genes, which had different and significantly higher evolutionary rates than unbiased genes. The fact that sex-biased genes, and, more specifically, male-biased genes, evolve more rapidly than unbiased genes is a well-known general evolutionary pattern documented from a diversity of organisms [5,60,61,62,63,64,65,66,67,68,69]. However, it is surprising that this signature is still present across some 400 million years of evolution when it remains unclear whether gene functionality and sex bias in their expression have been conserved. If these features changed during the course of evolution, it is still possible that faster rates in this case could be related to other expression features, such as tissue specificity and narrow expression profiles [65]. Indeed, faster rates of evolution associated with sex-biased expression have been explained as the result of several potential causes, including participation in specific processes such as spermatogenesis [62,69], activation in reproductive tissues relative to genes expressed in several tissues [13,70], linkage to the homogametic sex chromosome [13,70], relatively low levels of expression [71], or circumscription to specific stages of development [5]. These correlations are far from universal, and there are exceptions to each of the proposed patterns [5,68,69], much depending on the organism under study but also on their life histories. For example, female mating behavior in different species of *Anopheles* [Diptera: Culicidae]—some species of which are polyandrous, while others mate once in their lifetime—may have different impacts on sperm competition and selection and, consequently, on the evolutionary dynamics of sperm-related genes [69]. Moreover, while these factors could potentially lead to faster rates of evolution in sex-biased genes, protein–protein interactions could effectively constrain them [72], a possibility that will be discussed below.

### 4.4. Evolutionary Dynamics of Interacting Sperm Individualization Genes

Genetic interactions act as a dominant force explaining evolutionary rates, and the nature and type of interaction may prevail over other factors, such as the characteristics of gene expression [73]. There are hypotheses on how these two features may interact, such as the expected negative correlation between the number of protein interactions and evolutionary rates, or the proposition that interacting proteins should evolve at similar rates [74]. The micro- and macroevolutionary analyses of the effect of these interactions have facilitated significant advances in our understanding of these processes. On the one hand, our knowledge on the structure of genetic interaction networks, also for non-model organisms, is more detailed. On the other hand, the development of explicit, quantitative methods allows us to evaluate the architecture and properties of the networks relative to the biological features of their elements, particularly in the case of metabolic networks [75,76,77,78].

Among typical macroevolutionary patterns related to the protein–protein interaction network structure, it has been proposed that duplicated genes tend to be more highly connected in such networks [77]. The sperm individualization network shows an area that concentrates duplicated and relatively highly connected proteins (e.g., *Dredd*, *Dronc*, and *skap*); however, there was no statistical support for a correlation between these features. Correlations were found, nonetheless, for evolutionary rates when the undirected network was bipartitioned, adding statistical support to the intuitive notion of faster-evolving genes and proteins (*blanks*, *Dark*, *Dredd*, *Dronc*, *Duba*, and *Fadd*) appearing concentrated in one region of the network. Furthermore, we found a positive correlation between rank-based centrality and nucleotide substitution rates for beetles. In general, the opposite trend tends to be the norm, and highly connected genes usually show slower rates of evolution, maybe because the protein function depends on more topological interactions with other proteins, which constrain the possibility of change [74,75]. However, this is a controversial topic, and other examples of faster-evolving core proteins in an interaction network exist, such as the analysis of transcriptional networks in yeast [79]. In any case, it is too early to draw conclusions about the evolutionary trends in sperm individualization genes. Significant results were only obtained for beetles, for which we lack empirical evidence of the same gene interactions known in *Drosophila*, and different evolutionary dynamics seem to operate depending on the overall function of the network. This highlights the necessity for further research in beetles beyond the model organism *T. castaneum*.

The lack of significant or consistent results between analyses employing insect amino acid and beetle nucleotide sequence data may be explained, in part, by the partial view of the actual interactions in which sperm individualization genes participate. It is possible that the real nature and number of these interactions is not captured by the necessarily crude correction applied here (i.e., total number of receiving edges in the interactome). The structural measures obtained from the interaction network are intrinsic and the represented network of interactions is not isolated; therefore, these measures can show some biases [75]. A poorly connected node in the sperm individualization network can have many connections to other functional domains of the cell. For example, the proteins *Fadd* and *Mer* physically interact with the products of three and four other sperm individualization genes, respectively; however, in the complete interactome of *Drosophila*, they are known to interact physically or genetically with 100 and 165 other proteins, respectively. As already mentioned, another possibility is that the interaction network described for *Drosophila* is not universal for insects, totally or partially, and that the enforced topology is unable to capture evolutionary constraints for these genes in insects, or that the actual evolutionary dynamics of beetles are different from general trends in insects. Nevertheless, we tried to find intrinsic patterns that could be associated with the coordination of the genes of interest in a specific function, and at least in the case of beetles, there could be a signature worth exploring from a functional point of view. 

While we identified statistically significant differences in the rate of amino acid substitution in insects depending on hypothesized sex-biased expression, the study of nucleotide substitution rates in beetles for the same genes did not reveal any significant pattern. A somewhat reverse pattern was obtained in our exploration of evolutionary rates constrained by the architecture of a hypothesized network of interaction, wherein mainly nucleotide substitution rates of beetles showed some correlation with this architecture. This apparent contradiction and the complexity of the factors involved in explaining evolutionary rates make it difficult to fully explain these patterns satisfactorily. Before we can do that, we need more in-depth insight into the temporal and spatial expression profiles, effective function, genetic interactions, and pleiotropic effects of these genes in every single species, but also to incorporate information on their life history, which is likely to influence their evolutionary dynamics. 

## Figures and Tables

**Table 1:** Genes belonging to the ontology category “sperm individualization” (GO:0007291) in insects. Genes are identified by their names and their corresponding FlyBase ID in the *Drosophila melanogaster* genome. Information on the general function of the gene and sex biases in expression profiles is also given.

Gene	FlyBase ID	Function	Expression Profile
*Act5C*	FBgn0000042	cytoskeleton structure	unbiased
*Ance*	FBgn0012037	peptidase	unbiased
*aux*	FBgn0037218	ATP binding cofactor of kinase	unbiased
*blanks*	FBgn0035608	siRNA binding	male biased
*Bug22*	FBgn0032248	cilium organization and assembly	unbiased
*CdsA*	FBgn0010350	enzyme (CDP diglyceride synthetase)	unbiased
*Chc*	FBgn0000319	coated vesicles structure	unbiased
*ctp*	FBgn0011760	dynein complex assembly	unbiased
*Cul3*	FBgn0261268	protein binding	unbiased
*Cyt-c-d*	FBgn0086907	electron carrier	male biased
*Dark*	FBgn0263864	apoptosome assembly	unbiased
*didum*	FBgn0261397	unconventional myosin	unbiased
*Dredd*	FBgn0020381	enzyme (caspase)	unbiased
*Dronc*	FBgn0026404	enzyme (caspase)	unbiased
*Duba*	FBgn0036180	enzyme (deubiquitinase)	unbiased
*EcR*	FBgn0000546	transcription factor	unbiased
*eIF3m*	FBgn0033902	translation initiation factor	unbiased
*Fadd*	FBgn0038928	protein binding	unbiased
*gish*	FBgn0250823	enzyme (protein kinase)	unbiased
*gudu*	FBgn0031905	NA	male biased
*heph*	FBgn0011224	mRNA binding (translation repression)	unbiased
*hmw*	FBgn0038607	motile cilium assembly	male biased
*jar*	FBgn0011225	myosin	unbiased
*klhl10*	FBgn0040038	substrate recruiting for ubiquitin ligase complex	male biased
*Lasp*	FBgn0063485	actin/myosin scaffolding	unbiased
*Mer*	FBgn0086384	cytoskeletal protein binding	unbiased
*mlt*	FBgn0265512	microtubule removal	unbiased
*nes*	FBgn0026630	enzyme (lysophospholipid acyltransferase)	unbiased
*Npc1a*	FBgn0024320	sterol metabolism	unbiased
*nsr*	FBgn0034740	dynein complex assembly	male biased
*orb2*	FBgn0264307	translation factor	unbiased
*Osbp*	FBgn0020626	protein binding	unbiased
*oys*	FBgn0033476	enzyme (lysophospholipid acyltransferase)	unbiased
*Past1*	FBgn0016693	membrane assembly	unbiased
*Pen*	FBgn0011823	protein binding	unbiased
*poe*	FBgn0011230	calmodulin binding	unbiased
*porin*	FBgn0004363	membrane channel protein	unbiased
*Prosalpha6T*	FBgn0032492	enzyme (protease)	male biased
*scat*	FBgn0011232	protein binding	female biased
*shi*	FBgn0003392	GTPase for microtubule motility	unbiased
*skap*	FBgn0037643	ATP binding enzyme	unbiased
*sw*	FBgn0003654	dynein complex assembly	unbiased
*Taz*	FBgn0026619	enzyme (phospholipid transacylase)	unbiased
*Vps28*	FBgn0021814	vesicular trafficking	unbiased

**Table 2:** Beetle species used in the current study and their current systematic placement. Unless specified otherwise, gene sequence data were obtained from 1KITE.

Suborder Infraorder	Superfamily	Family	Species	Library ID (1KITE)
Archostemata		Micromalthidae	*Micromalthus debilis*	INSqzbTABRAAPEI-210
Adephaga		Aspidytidae	*Sinaspidytes wrasei*	WHINSnuyTAAARAAPEI-47
		Carabidae	*Cicindela hybrida*	INShauTBARAAPEI-21
		Dytiscidae	*Cybister lateralimarginalis*	INSnfrTADRAAPEI-16
		Gyrinidae	*Gyrinus marinus*	INSnfrTBERAAPEI-19
		Noteridae	*Noterus clavicornis*	INShkeTALRAAPEI-37
Myxophaga		Hydroscaphidae	*Hydroscapha redfordi*	INSntgTARRAAPEI-208
		Lepiceridae	*Lepicerus* sp.	INSytvTAJRAAPEI-19
Polyphaga				
“basal Polyphaga”	Scirtoidea	Scirtidae	*Cyphon laevipennis*	INSjdsTBDRAAPEI-47
Bostrichiformia	Bostrichoidea	Bostrichidae	*Xylobiops basilaris*	WHANIsrmTMCLRAAPEI-11
Cucujiformia	Chrysomeloidea	Cerambycidae	*Anoplophora glabripennis* ^a^	-
		Chrysomelidae	*Calligrapha multipunctata* ^b^	-
			*Leptinotarsa decemlineata* ^a^	-
	Cleroidea	Byturidae	*Byturus ochraceus*	INShkeTAORAAPEI-43
		Cleridae	*Thanasimus formicarius*	INShkeTCERAAPEI-79
	Coccinelloidea	Coccinellidae	*Rhyzobius pseudopulcher*	WHANIsrmTMABRAAPEI-9
	Curculionoidea	Curculionidae	*Dendroctonus ponderosae* ^a^	-
				
	Tenebrionoidea	Meloidae	*Meloe violaceus*	INShauTAYRAAPEI-19
		Tenebrionidae	*Tribolium castaneum* ^a^	-
		Zopheridae	*Bitoma cylindrica*	WHANIsrmTMAPRAAPEI-39
Elateriformia	Buprestoidea	Buprestidae	*Agrilus planipennis* ^a^	-
	Elateroidea	Lampyridae	*Lamprohiza splendidula*	INShkeTCGRAAPEI-87
				
Scarabaeiformia	Scarabaeoidea	Scarabaeidae	*Cetonia aurata pisana*	WHANIsrmTMAVRAAPEI-53
			*Onthophagus taurus* ^a^	-
Staphyliniformia	Hydrophiloidea	Hydrophilidae	*Hydrochara caraboides*	INShauTASRAAPEI-13
	Staphylinoidea	Staphylinidae	*Ocypus brunnipes*	INShkeTCMRAAPEI-45

^a^ Beetle model species and data obtained from OrthoDB; ^b^ Data available from [22].

**Table 3:** Summary of sequence characteristics of genes retrieved from OrthoDB. The table lists the number of sequences (N) and species (Sp; with a maximum of 119 species), number of species in which the gene is single copy (Single), the median protein length (L), and relative evolutionary rate (r) as tabulated in OrthoDB. Furthermore, the number (n) of aligned sequences in this study and the alignment lengths (Length), as well as the inferred optimal evolutionary model, are given.

Gene	*N*	Sp	Single	L	r	*n*	Length	Model
*Act5C*	517	115	7	376	0.60	-	-	-
*Ance*	238	109	30	631	0.92	90	621	LG + G + I
*aux*	136	112	94	1164	1.21	126	1755	JTT + G + I + F
*blanks*	172	107	71	348	1.55	122	719	JTT + G + I + F
*Bug22*	201	115	36	200	0.61	83	270	LG + G + I
*CdsA*	118	109	101	445	0.76	122	574	JTT + G + I + F
*Chc*	123	115	110	1676	0.63	130	1726	JTT + G + I
*ctp*	121	98	79	89	0.57	-	-	-
*Cul3*	153	116	91	780	0.73	133	858	JTT + G + I
*Cyt-c-d*	148	110	74	108	0.65	-	-	-
*Dark*	119	106	94	1378	1.99	112	2193	JTT + G + I + F
*didum*	126	113	102	1793	1.10	124	2175	LG + G + I
*Dredd*	91	81	75	493	1.76	98	676	JTT + G + I + F
*Dronc*	128	96	78	425	1.67	119	654	WAG + G + I + F
*Duba*	105	99	93	628	0.95	113	1087	JTT + G + I + F
*EcR*	121	113	105	515	0.83	120	576	JTT + G + I
*eIF3m*	116	113	111	387	0.77	130	394	JTT + G + I
*Fadd*	96	93	90	246	1.77	108	353	JTT + G + I + F
*gish*	129	113	99	441	0.73	124	401	JTT + G + I
*gudu*	125	115	106	689	1.01	124	658	LG + G + I
*heph*	206	114	47	285	0.78	114	597	JTT + G + I + F
*hmw*	78	76	74	260	1.38	80	925	JTT + G + I + F
*jar*	131	111	97	1238	0.86	129	1375	JTT + G + I + F
*klhl10*	214	109	54	619	0.96	113	630	LG + G + I
*Lasp*	113	105	97	321	0.79	121	298	JTT + G + I
*Mer*	120	112	105	605	0.87	129	686	JTT + G + I
*mlt*	124	112	103	477	1.10	130	651	LG + G + I + F
*nes*	122	112	104	474	1.21	128	472	LG + G + I + F
*Npc1a*	216	116	23	1256	0.98	124	1435	LG + G + I
*nsr*	317	115	38	355	0.92	129	563	JTT + G + I
*orb2*	115	106	98	351	0.62	105	293	JTT + G + I
*Osbp*	158	112	79	597	0.91	130	1094	JTT + G + I
*oys*	121	108	97	505	1.03	115	463	LG + G + I
*Past1*	124	114	104	534	0.66	120	564	LG + G + I
*Pen*	342	116	7	519	0.83	121	593	LG + G + I + F
*poe*	183	115	84	2949	1.08	129	3846	JTT + G + I + F
*porin*	124	108	98	282	0.86	127	286	LG + G + I + F
*Prosalpha6T*	126	108	91	277	0.77	125	312	LG + G + I + F
*scat*	130	116	103	942	1.11	133	1233	JTT + G + I
*shi*	133	113	94	857	0.67	132	1005	LG + G + I
*skap*	247	116	8	424	0.80	128	476	LG + G + I
*sw*	125	115	109	655	0.80	133	755	JTT + G + I
*Taz*	105	102	99	265	0.91	118	303	LG + G + I + F
*Vps28*	135	112	94	212	0.72	131	213	LG + G + I

**Table 4:** Characteristics of amino acid datasets of sperm individualization proteins of insects deduced from information in public databases (B: unbiased [0] and sex-biased [1] genes; N: non-interacting [0] and interacting [1]), as well as information deduced from their phylogenetic analyses, including duplications (D: single [0] and multicopy [1]), evolutionary rates, evolutionary rate heterogeneity (ucld.stdev), and the estimated age of the clade Coleoptera.

Gene	B/D/N	Substitution Rate (×10^−3^)	ucld.stdev	Age Coleoptera
*hmw*	1/0/0	9.67 ± 1.349	2.972	-
*Dark*	0/0/1	5.27 ± 0.282	0.418	264.2 [214.3–314.2]
*blanks*	1/1/1	4.38 ± 0.382	0.483	310.6 [248.2–376.1]
*Dredd*	0/0/1	4.23 ± 0.219	0.309	278.4 [239.2–315.8]
*Fadd*	0/0/1	4.21 ± 0.277	0.345	-
*Dronc*	0/1/1	3.62 ± 0.221	0.391	379.8 [339.5–426.0] ^b^
*Duba*	0/0/1	3.18 ± 0.257	0.566	348.2 [248.5–450.2] ^b^
*nsr*	1/1/0	3.00 ± 0.279	0.672	194.1 [127.4–262.5]
*Bug22*	0/1/0	2.82 ± 0.278	0.559	-
*scat*	1/0/0	2.73 ± 0.259	>3 ^a^	267.8 [185.8–359.0]
*aux*	0/0/1	2.45 ± 0.154	0.386	311.0 [252.3–372.7]
*nes*	0/0/0	2.27 ± 0.130	0.423	307.2 [253.7–365.2] ^b^
*poe*	0/0/0	2.10 ± 0.174	>3 ^a^	328.7 [227.3–433.1]
*Osbp*	0/1/0	2.05 ± 0.180	0.601	299.6 [230.3–375.4] ^b^
*Npc1a*	0/1/0	1.91 ± 0.157	>3 ^a^	334.2 [231.5–442.6]
*didum*	0/0/1	1.90 ± 0.126	0.511	337.0 [264.9–419.9]
*Pen*	0/1/0	1.78 ± 0.120	0.531	273.2 [208.2–340.5]
*klhl10*	1/1/1	1.69 ± 0.124	0.816	-
*Prosalpha6T*	1/1/0	1.68 ± 0.151	0.559	248.5 [185.6–315.1]
*oys*	0/0/0	1.57 ± 0.127	0.558	-
*gudu*	1/0/0	1.45 ± 0.115	0.553	347.7 [258.1–444.1]
*Ance*	0/1/0	1.42 ± 0.099	0.396	-
*sw*	0/0/1	1.38 ± 0.171	3.712	247.5 [133.2–374.3]
*Taz*	0/0/0	1.33 ± 0.111	0.607	312.2 [231.9–401.0]
*Mer*	0/0/1	1.30 ± 0.130	0.936	304.5 [208.4–409.9]
*skap*	0/1/1	1.15 ± 0.116	0.557	314.4 [214.9–424.3] ^b^
*CdsA*	0/0/0	1.15 ± 0.103	0.629	350.5 [258.6–440.6] ^b^
*jar*	0/0/1	1.06 ± 0.086	0.482	403.2 [315.0–494.6] ^b^
*porin*	0/0/0	0.99 ± 0.106	0.779	-
*Lasp*	0/0/1	0.98 ± 0.138	0.930	451.5 [288.4–633.0] ^b^
*Cul3*	0/1/1	0.98 ± 0.130	3.798	223.1 [112.6–351.7] ^b^
*EcR*	0/0/0	0.93 ± 0.091	0.789	-
*heph*	0/0/0	0.90 ± 0.118	3.919	-
*eIF3m*	0/0/1	0.86 ± 0.084	0.480	363.0 [277.4–462.1]
*shi*	0/0/1	0.74 ± 0.092	3.847	253.2 [124.3–397.0]
*Past1*	0/1/1	0.71 ± 0.069	0.713	387.4 [273.0–513.2] ^b^
*Vps28*	0/0/0	0.63 ± 0.079	0.822	-
*gish*	0/1/0	0.47 ± 0.070	4.174	262.9 [137.6–401.9]
*mlt*	0/0/0	0.42 ± 0.523	3.268	244.6 [121.4–368.1] ^b^
*Chc*	0/0/1	0.32 ± 0.035	0.700	346.6 [242.1–464.4]
*orb2*	0/1/0	0.24 ± 0.035	1.414	180.4 [52.1–328.6]

^a^ Data analyzed under exponential relaxed clock, with ucld.stdev estimated from inconclusive runs under an uncorrelated lognormal relaxed clock; ^b^ Coleoptera is rendered paraphyletic by the inclusion of Strepsiptera.

**Table 5:** Characteristics of the nucleotide phylogenetic data sets of sperm individualization genes in Coleoptera. The number of species (N), length of nucleotide sequence alignments (L), the determined evolutionary model, inferred evolutionary rates, and information on rate heterogeneity (ucld.stdev) are given for each gene.

Gene	N	L	Model	Substitution Rate (×10^−3^)	ucld.stdev
*Cul3*	25	2196	TN93 + G + I	11.90 ± 2.603	2.707
*gish*	24	1197	GTR + G + I	9.34 ± 1.981	2.821
*Act5C*	15	1128	GTR + G + I	8.91 ± 2.232	2.839
*scat*	25	1974	GTR + G + I	7.26 ± 1.397	2.958
*eIF3m*	25	1155	GTR + G + I	7.06 ± 1.394	2.872
*poe*	23	3291	GTR + G + I	6.90 ± 1.322	2.844
*Dredd*	23	732	GTR + G + I	6.41 ± 1.255	3.012
*CdsA*	21	1323	GTR + G + I	6.35 ± 1.229	2.954
*blanks*	25	672	GTR + G + I	5.82 ± 1.172	2.994
*shi*	25	2610	GTR + G + I	5.81 ± 1.161	2.918
*skap*	24	1302	GTR + G + I	5.09 ± 0.984	3.033
*Dark*	14	1863	GTR + G + I	4.96 ± 0.905	2.947
*Duba*	20	858	GTR + G + I	4.93 ± 0.997	2.916
*Prosalpha6T*	25	822	GTR + G + I	4.90 ± 0.959	3.041
*Chc*	22	4944	GTR + G + I	4.78 ± 0.410	0.270
*klhl10*	14	1779	GTR + G + I	4.48 ± 0.849	2.952
*jar*	25	3393	GTR + G + I	4.47 ± 0.840	3.005
*didum*	21	5073	GTR + G + I	4.41 ± 0.817	3.029
*oys*	14	1347	GTR + G + I	4.41 ± 0.839	3.019
*ctp*	8	267	GTR + G	3.79 ± 1.107	0.232
*mlt*	24	1248	GTR + G + I	3.61 ± 0.464	0.474
*sw*	25	1455	GTR + G + I	3.61 ± 0.347	0.344
*Taz*	23	774	GTR + G + I	3.52 ± 0.278	0.340
*Fadd*	19	228	GTR + G + I	3.41 ± 0.495	0.463
*nsr*	24	780	GTR + G + I	3.39 ± 0.464	0.409
*orb2*	8	834	GTR + G + I	3.36 ± 0.834	0.477
*Npc1a*	23	3756	GTR + G + I	3.32 ± 0.280	0.255
*Dronc*	24	954	GTR + G + I	3.17 ± 0.265	0.221
*EcR*	17	1278	GTR + G + I	3.13 ± 0.396	0.382
*Vps28*	26	573	GTR + G + I	3.12 ± 0.509	0.169
*Osbp*	25	1881	GTR + G + I	3.09 ± 0.285	0.365
*Past1*	13	1566	GTR + G + I	3.09 ± 0.344	0.186
*aux*	18	2130	GTR + G + I	3.09 ± 0.295	0.252
*Lasp*	25	423	GTR + G + I	3.08 ± 0.495	0.110
*gudu*	15	1848	GTR + G + I	2.93 ± 0.280	0.450
*Pen*	24	1440	GTR + G + I	2.92 ± 0.252	0.296
*porin*	25	849	GTR + G + I	2.78 ± 0.393	0.499
*Mer*	23	1701	GTR + G + I	2.78 ± 0.260	0.329
*Ance*	18	1716	GTR + G + I	2.59 ± 0.198	0.299
*hmw*	8	201	GTR + G	2.57 ± 0.457	0.118
*nes*	25	1317	GTR + G + I	2.08 ± 0.171	0.538

## Supplementary Materials

The following are available online at https://www.mdpi.com/2073-4425/10/10/776/s1, File S1. Maximum likelihood trees based on the amino acid alignments of different sperm individualization proteins in insects. File S2. Bayesian inference trees based on the amino acid alignments of different sperm individualization proteins in insects. File S3. Maximum likelihood trees based on the nucleotide alignments of different sperm individualization genes in beetles. File S4. Bayesian inference trees based on the nucleotide alignments of different sperm individualization genes in beetles.
