# Supplementary material for: Patterns and Constraints in the Evolution of Sperm Individualization Genes in Insects, with an Emphasis on Beetles
Source: Genes (Basel). 2019 Oct 4;10(10):776. doi: 10.3390/genes10100776 (PMC6826512; doi:10.3390/genes10100776)
Supplement: Supplementary file 1 [file genes-10-00776-s001.pdf]

File S1. Maximum likelihood trees based on the amino acid alignments of different sperm individualization proteins in insects.

Tree	Gene	Model	Gamma	Invariant	Log likelihood
**1**	*Ance**	LG	0.975	0.075	-36264.30406
**2**	*aux*	LG	0.861	0.042	-101341.43324
**3**	*blanks*	JTT	1.000	0.006	-51030.51327
**4**	*Bug22**	LG	0.970	0.076	-11344.29415
**5**	*CdsA*	JTT	0.662	0.164	-20188.93347
**6**	*Chc*	JTT	0.646	0.429	-27341.73092
**7**	*Cul3**	JTT	0.617	0.203	-21620.12771
**8**	*Dark*	JTT	1.873	0.003	-175174.99008
**9**	*didum*	LG	0.896	0.094	-118892.32085
**10**	*Dredd**	JTT	1.420	0.006	-57705.56572
**11**	*Dronc*	WAG	1.405	0.013	-53433.37737
**12**	*Duba*	JTT	0.992	0.055	-43655.01217
**13**	*EcR*	JTT	0.663	0.140	-13958.07538
**14**	*eIF3m*	JTT	0.817	0.145	-15809.34033
**15**	*Fadd*	JTT	1.307	0.004	-30734.15211
**16**	*gish*	JTT	0.678	0.335	-7663.02559
**17**	*gudu*	LG	0.921	0.038	-38783.13178
**18**	*heph*	JTT	0.965	0.170	-15480.89741
**19**	*hmw*	JTT	1.149	0.007	-35247.66673
**20**	*jar*	LG	0.857	0.181	-62829.80003
**21**	*klhl10**	LG	0.913	0.017	-35556.78931
**22**	*Lasp*	JTT	0.844	0.249	-9432.56476
**23**	*Mer*	JTT	0.699	0.123	-21840.64371
**24**	*mlt*	LG	0.628	0.095	-34633.21523
**25**	*nes*	LG	1.073	0.040	-42646.95754
**26**	*Npc1a**	LG	0.833	0.156	-85235.55965
**27**	*nsr**	JTT	0.854	0.036	-28961.01227
**28**	*orb2*	JTT	0.489	0.214	-3222.50637
**29**	*Osbp*	JTT	0.863	0.082	-51127.87538
**30**	*oys*	LG	0.984	0.066	-29099.88610
**31**	*Past1*	LG	0.711	0.302	-16096.65909
**32**	*Pen**	LG	0.930	0.090	-38161.29725
**33**	*poe*	JTT	0.769	0.060	-163837.44235
**34**	*porin*	LG	1.341	0.028	-15895.84791
**35**	*Prosalpha6T*	LG	0.792	0.197	-15675.74518
**36**	*scat*	JTT	0.939	0.037	-72098.82783
**37**	*shi*	LG	0.650	0.287	-23338.29850
**38**	*skap**	LG	0.831	0.257	-19572.71029
**39**	*sw*	JTT	0.960	0.066	-27022.45452
**40**	*Taz*	LG	1.034	0.157	-17370.63510
**41**	*Vps28*	LG	0.720	0.100	-6256.72767

*Orthologs to the *Drosophila* paralog with sperm individualization function.

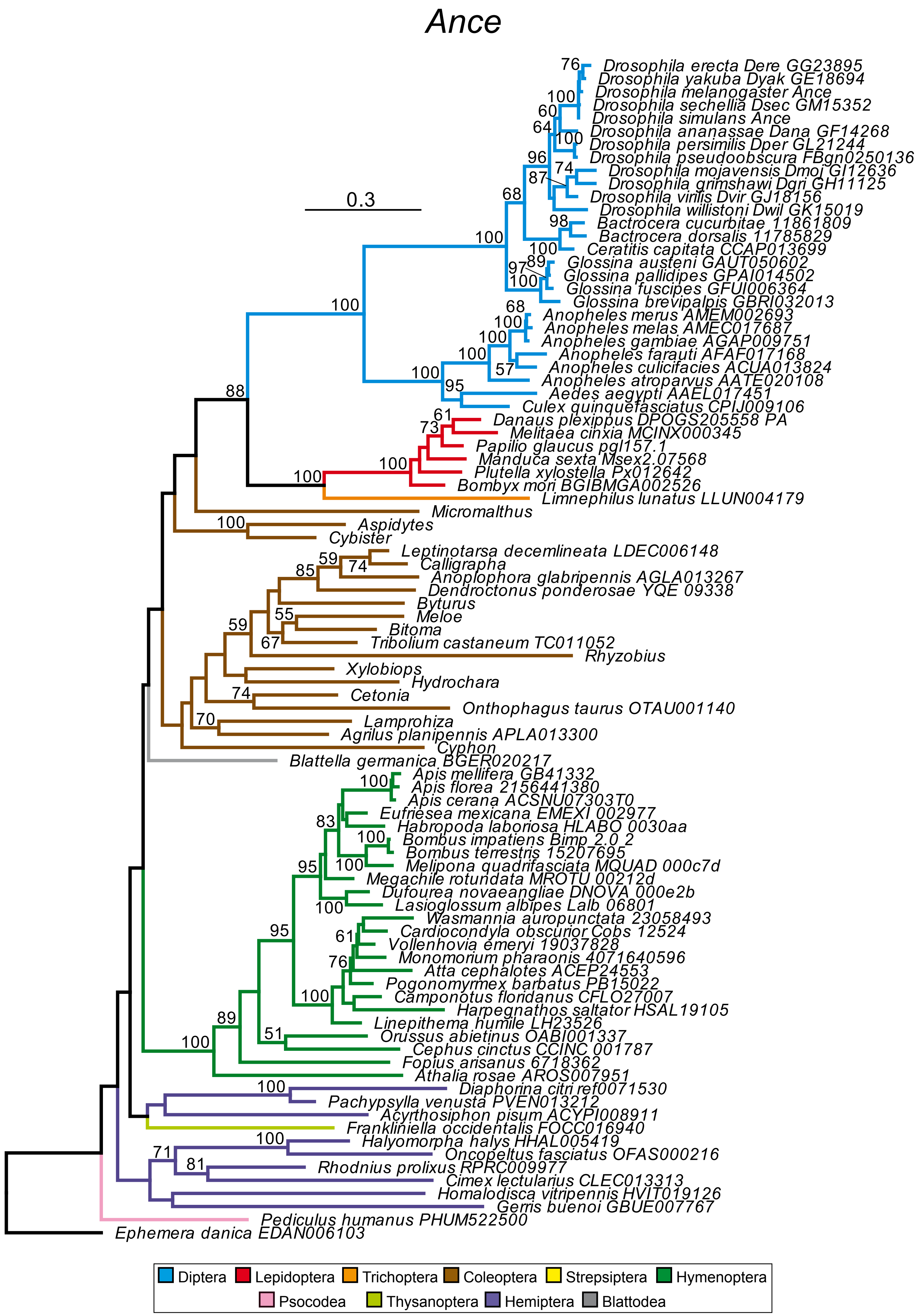

Ance
0.3
Drosophila erecta Dere GG23895
Drosophila yakuba Dyak GE18694
Drosophila melanogaster Ance
Drosophila sechellia Dsec GM15352
Drosophila simulans Ance
Drosophila ananassae Dana GF14268
Drosophila persimilis Dper GL21244
Drosophila pseudoobscura FBgn0250136
Drosophila mojavensis Dmoj GI12636
Drosophila grimshawi Dgri GH11125
Drosophila virilis Dvir GJ18156
Drosophila willistoni Dwil GK15019
Bactrocera cucurbitae 11861809
Bactrocera dorsalis 11785829
Ceratitis capitata CCAP013699
Glossina austeni GAUT050602
Glossina pallidipes GPAI014502
Glossina fuscipes GFUI006364
Glossina brevipalpis GBRI032013
Anopheles merus AMEM002693
Anopheles melas AMEC017687
Anopheles gambiae AGAP009751
Anopheles farauti AFAF017168
Anopheles culicifacies ACUA013824
Anopheles atroparvus AATE020108
Aedes aegypti AAEL017451
Culex quinquefasciatus CPIJ009106
Danaus plexippus DPOGS205558 PA
Melitaea cinxia MCINX000345
Papilio glaucus pgl157.1
Manduca sexta Msex2.07568
Plutella xylostella Px012642
Bombyx mori BGIBMGA002526
Limnephilus lunatus LLUN004179
Micromalthus
Aspidytes
Cybister
Leptinotarsa decemlineata LDEC006148
Calligrapha
Anoplophora glabripennis AGLA013267
Dendroctonus ponderosae YQE 09338
Byturus
Meloe
Bitoma
Tribolium castaneum TC011052
Rhyzobius
Xylobiops
Hydrochara
Cetonia
Onthophagus taurus OTAU001140
Lamprohiza
Agrilus planipennis APLA013300
Cyphon
Blattella germanica BGER020217
Apis mellifera GB41332
Apis florea 2156441380
Apis cerana ACSNU07303T0
Eufriesea mexicana EMEXI 002977
Habropoda laboriosa HLABO 0030aa
Bombus impatiens Bimp 2.0 2
Bombus terrestris 15207695
Melipona quadrifasciata MQUAD 000c7d
Megachile rotundata MROTU 00212d
Dufourea novaeangliae DNOVA 000e2b
Lasioglossum albipes Lalb 06801
Wasmannia auropunctata 23058493
Cardiocondyla obscurior Cobs 12524
Vollenhovia emeryi 19037828
Monomorium pharaonis 4071640596
Atta cephalotes ACEP24553
Pogonomyrmex barbatus PB15022
Camponotus floridanus CFLO27007
Harpegnathos saltator HSAL19105
Linepithema humile LH23526
Orussus abietinus OABI001337
Cephus cinctus CCINC 001787
Fopius arisanus 6718362
Athalia rosae AROS007951
Diaphorina citri ref0071530
Pachypsylla venusta PVEN013212
Acyrthosiphon pisum ACYPI008911
Frankliniella occidentalis FOCC016940
Halyomorpha halys HHAL005419
Oncopeltus fasciatus OFAS000216
Rhodnius prolixus RPRC009977
Cimex lectularius CLEC013313
Homalodisca vitripennis HVIT019126
Gerris buenoi GBUE007767
Pediculus humanus PHUM522500
Ephemera danica EDAN006103
Diptera
Lepidoptera
Trichoptera
Coleoptera
Strepsiptera
Hymenoptera
Psocodea
Thysanoptera
Hemiptera
Blattodea

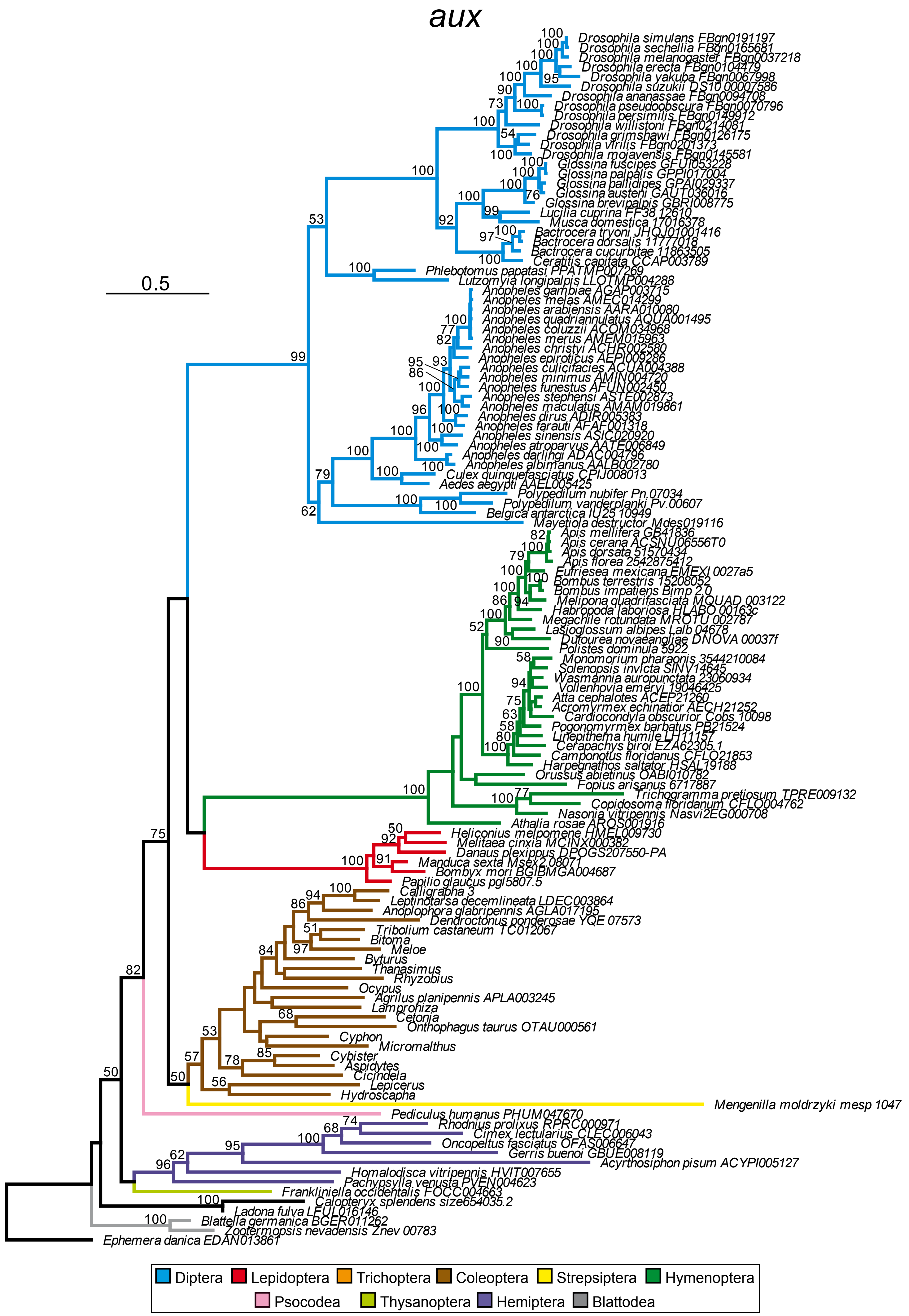
aux
0.5
Drosophila simulans FBgn0191197
Drosophila sechellia FBgn0165681
Drosophila melanogaster FBgn0037218
Drosophila erecta FBgn0104479
Drosophila yakuba FBgn0067998
Drosophila suzukii DS10 00007586
Drosophila ananassae FBgn0094708
Drosophila pseudoobscura FBgn0070796
Drosophila persimilis FBgn0149912
Drosophila willistoni FBgn0214081
Drosophila grimshawi FBgn0126175
Drosophila virilis FBgn0201373
Drosophila mojavensis FBgn0145581
Glossina fuscipes GFUI053228
Glossina palpalis GPPI017004
Glossina pallidipes GPAI029337
Glossina austeni GAUT036016
Glossina brevipalpis GBRI008775
Lucilia cuprina FF38 12610
Musca domestica 17016378
Bactrocera tryoni JHQJ01001416
Bactrocera dorsalis 11777018
Bactrocera cucurbitae 11863505
Ceratitis capitata CCAP003789
Phlebotomus papatasi PPATMP007269
Lutzomyia longipalpis LLOTMP004288
Anopheles gambiae AGAP003715
Anopheles melas AMEC014299
Anopheles arabiensis AARA010080
Anopheles quadriannulatus AQUA001495
Anopheles coluzzii ACOM034968
Anopheles merus AMEM015963
Anopheles christyi ACHR002580
Anopheles epiroticus AEPI009286
Anopheles culicifacies ACUA004388
Anopheles minimus AMIN004720
Anopheles funestus AFUN002450
Anopheles stephensi ASTE002873
Anopheles maculatus AMAM019861
Anopheles dirus ADIR005383
Anopheles farauti AFAF001318
Anopheles sinensis ASIC020920
Anopheles atroparvus AATE006849
Anopheles darlingi ADAC004796
Anopheles albimanus AALB002780
Culex quinquefasciatus CPIJ008013
Aedes aegypti AAEL005425
Polypedilum nubifer Pn.07034
Polypedilum vanderplanki Pv.00607
Belgica antarctica IU25 10949
Mayetiola destructor Mdes019116
Apis mellifera GB41836
Apis cerana ACSNU06556T0
Apis dorsata 51570434
Apis florea 2542875412
Eufriesea mexicana EMEXI 0027a5
Bombus terrestris 15208052
Bombus impatiens Bimp 2.0
Melipona quadrifasciata MQUAD 003122
Habropoda laboriosa HLABO 00163c
Megachile rotundata MROTU 002787
Lasioglossum albipes Lalb 04678
Dufourea novaeangliae DNOVA 00037f
Polistes dominula 5922
Monomorium pharaonis 3544210084
Solenopsis invicta SINV14645
Wasmannia auropunctata 23060934
Vollenhovia emeryi 19046425
Atta cephalotes ACEP21260
Acromyrmex echinatior AECH21252
Cardiocondyla obscurior Cobs 10098
Pogonomyrmex barbatus PB21524
Linepithema humile LH11157
Cerapachys biroi EZA62305.1
Camponotus floridanus CFLO21853
Harpegnathos saltator HSAL19188
Orussus abietinus OABI010782
Fopius arisanus 6717887
Trichogramma pretiosum TPRE009132
Copidosoma floridanum CFLO004762
Nasonia vitripennis Nasvi2EG000708
Athalia rosae AROS001916
Heliconius melpomene HMEL009730
Melitaea cinxia MCINX000382
Danaus plexippus DPOGS207550-PA
Manduca sexta Msex2.08071
Bombyx mori BGIBMGA004687
Papilio glaucus pgl5807.5
Calligrapha 3
Leptinotarsa decemlineata LDEC003864
Anoplophora glabripennis AGLA017195
Dendroctonus ponderosae YQE 07573
Tribolium castaneum TC012067
Bitoma
Meloe
Byturus
Thanasimus
Rhyzobius
Ocypus
Agrilus planipennis APLA003245
Lamprohiza
Cetonia
Onthophagus taurus OTAU000561
Cyphon
Micromalthus
Cybister
Aspidytes
Cicindela
Lepicerus
Hydroscapha
Mengenilla moldrzyki mesp 1047
Pediculus humanus PHUM047670
Rhodnius prolixus RPRC000971
Cimex lectularius CLEC006043
Oncopeltus fasciatus OFAS006647
Gerris buenoi GBUE008119
Acyrthosiphon pisum ACYPI005127
Homalodisca vitripennis HVIT007655
Pachypsylla venusta PVEN004623
Frankliniella occidentalis FOCC004663
Calopteryx splendens size654035.2
Ladona fulva LFUL016146
Blattella germanica BGER011262
Zootermopsis nevadensis Znev 00783
Ephemera danica EDAN013861
Diptera
Lepidoptera
Trichoptera
Coleoptera
Strepsiptera
Hymenoptera
Psocodea
Thysanoptera
Hemiptera
Blattodea

blanks

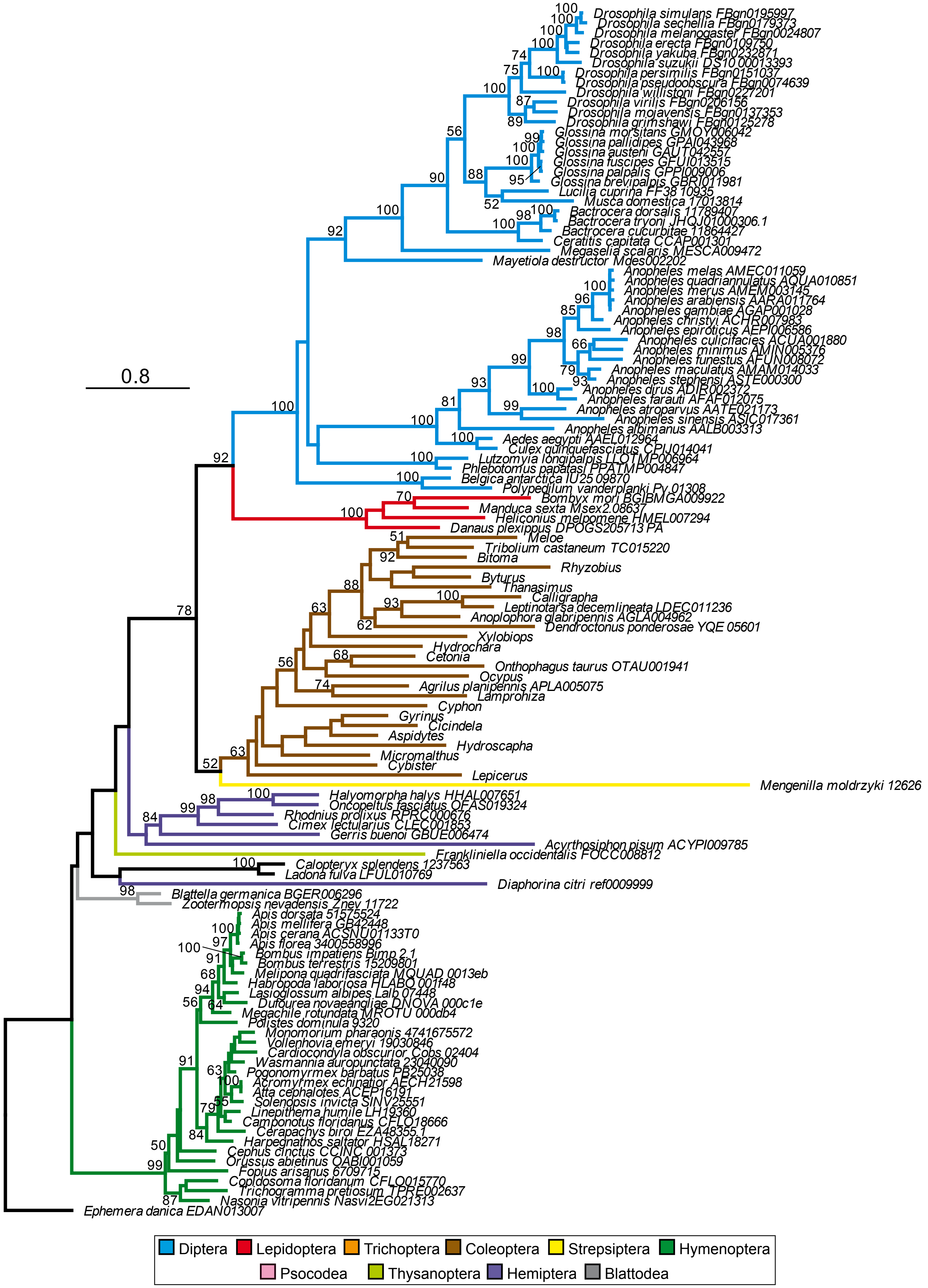
0.8
Drosophila simulans FBgn0195997
Drosophila sechellia FBgn0179373
Drosophila melanogaster FBgn0024807
Drosophila erecta FBgn0109750
Drosophila yakuba FBgn0232871
Drosophila suzukii DS10 00013393
Drosophila persimilis FBgn0151037
Drosophila pseudoobscura FBgn0074639
Drosophila willistoni FBgn0227201
Drosophila virilis FBgn0206156
Drosophila mojavensis FBgn0137353
Drosophila grimshawi FBgn0125278
Glossina morsitans GMOY006042
Glossina pallidipes GPAI043968
Glossina austeni GAUT042557
Glossina fuscipes GFUI013515
Glossina palpalis GPPI009006
Glossina brevipalpis GBRI011981
Lucilia cuprina FF38 10935
Musca domestica 17013814
Bactrocera dorsalis 11789407
Bactrocera tryoni JHQJ01000306.1
Bactrocera cucurbitae 11864427
Ceratitis capitata CCAP001301
Megaselia scalaris MESCA009472
Mayetiola destructor Mdes002202
Anopheles melas AMEC011059
Anopheles quadriannulatus AQUA010851
Anopheles merus AMEM003145
Anopheles arabiensis AARA011764
Anopheles gambiae AGAP001028
Anopheles christyi ACHR007983
Anopheles epiroticus AEPI006586
Anopheles culicifacies ACUA001880
Anopheles minimus AMIN005376
Anopheles funestus AFUN008072
Anopheles maculatus AMAM014033
Anopheles stephensi ASTE000300
Anopheles dirus ADIR002372
Anopheles farauti AFAF012075
Anopheles atroparvus AATE021173
Anopheles sinensis ASIC017361
Anopheles albimanus AALB003313
Aedes aegypti AAEL012964
Culex quinquefasciatus CPIJ014041
Lutzomyia longipalpis LLOTMP006964
Phlebotomus papatasi PPATMP004847
Belgica antarctica IU25 09870
Polypedilum vanderplanki Py 01308
Bombyx mori BGIBMGA009922
Manduca sexta Msex2.08637
Heliconius melpomene HMEL007294
Danaus plexippus DPOGS205713 PA
Meloe
Tribolium castaneum TC015220
Bitoma
Rhyzobius
Byturus
Thanasimus
Calligrapha
Leptinotarsa decemlineata LDEC011236
Anoplophora glabripennis AGLA004962
Dendroctonus ponderosae YQE 05601
Xylobiops
Hydrochara
Cetonia
Onthophagus taurus OTAU001941
Ocypus
Agrilus planipennis APLA005075
Lamprohiza
Cyphon
Gyrinus
Cicindela
Aspidytes
Hydroscapha
Micromalthus
Cybister
Lepicerus
Mengenilla moldrzyki 12626
Halyomorpha halys HHAL007651
Oncopeltus fasciatus OFAS019324
Rhodnius prolixus RPRC000676
Cimex lectularius CLEC001853
Gerris buenoi GBUE006474
Acyrthosiphon pisum ACYPI009785
Frankliniella occidentalis FOCC008812
Calopteryx splendens 1237563
Ladona fulva LFUL010769
Diaphorina citri ref0009999
Blattella germanica BGER006296
Zootermopsis nevadensis Znev 11722
Apis dorsata 51575524
Apis mellifera GB42448
Apis cerana ACSNU01133T0
Apis florea 3400558996
Bombus impatiens Bimp 2.1
Bombus terrestris 15209801
Melipona quadrifasciata MQUAD 0013eb
Habropoda laboriosa HLABO 001f48
Lasioglossum albipes Lalb 07448
Dufourea novaeangliae DNOVA 000c1e
Megachile rotundata MROTU 000db4
Polistes dominula 9320
Monomorium pharaonis 4741675572
Vollenhovia emeryi 19030846
Cardiocondyla obscurior Cobs 02404
Wasmannia auropunctata 23040090
Pogonomyrmex barbatus PB25038
Acromyrmex echinatior AECH21598
Atta cephalotes ACEP16191
Solenopsis invicta SINV25551
Linepithema humile LH19360
Camponotus floridanus CFLO18666
Cerapachys biroi EZA48355.1
Harpegnathos saltator HSAL18271
Cephus cinctus CCINC 001373
Orussus abietinus OABI001059
Fopius arisanus 6709715
Copidosoma floridanum CFLO015770
Trichogramma pretiosum TPRE002637
Nasonia vitripennis Nasvi2EG021313
Ephemera danica EDAN013007
Diptera
Lepidoptera
Trichoptera
Coleoptera
Strepsiptera
Hymenoptera
Psocodea
Thysanoptera
Hemiptera
Blattodea

Bug22

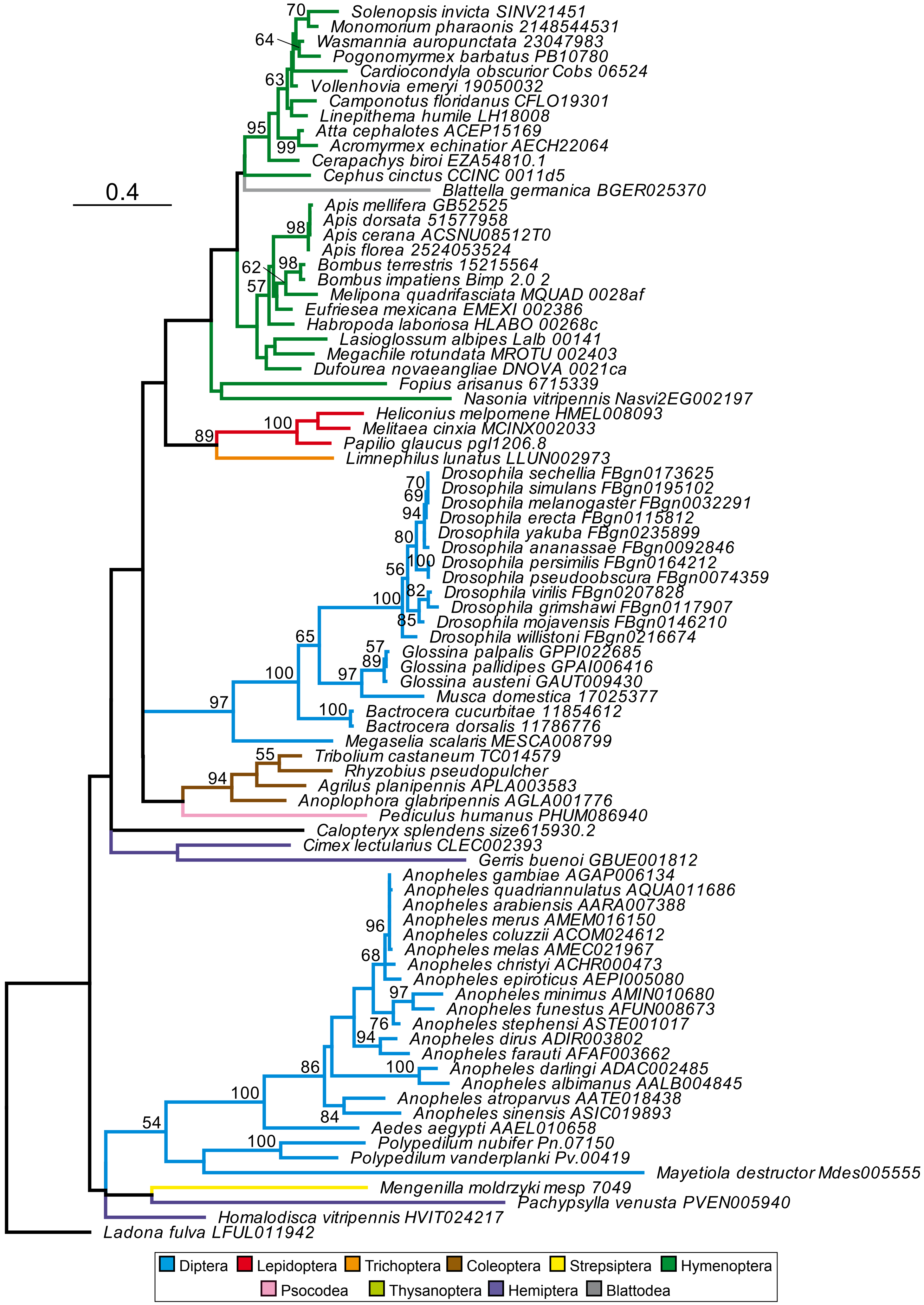

0.4
Solenopsis invicta SINV21451
Monomorium pharaonis 2148544531
Wasmannia auropunctata 23047983
Pogonomyrmex barbatus PB10780
Cardiocondyla obscurior Cobs 06524
Vollenhovia emeryi 19050032
Camponotus floridanus CFLO19301
Linepithema humile LH18008
Atta cephalotes ACEP15169
Acromyrmex echinatior AECH22064
Cerapachys biroi EZA54810.1
Cephus cinctus CCINC 0011d5
Blattella germanica BGER025370
Apis mellifera GB52525
Apis dorsata 51577958
Apis cerana ACSNU08512T0
Apis florea 2524053524
Bombus terrestris 15215564
Bombus impatiens Bimp 2.0 2
Melipona quadrifasciata MQUAD 0028af
Eufriesea mexicana EMEXI 002386
Habropoda laboriosa HLABO 00268c
Lasioglossum albipes Lalb 00141
Megachile rotundata MROTU 002403
Dufourea novaeangliae DNOVA 0021ca
Fopius arisanus 6715339
Nasonia vitripennis Nasvi2EG002197
Heliconius melpomene HMEL008093
Melitaea cinxia MCINX002033
Papilio glaucus pgl1206.8
Limnephilus lunatus LLUN002973
Drosophila sechellia FBgn0173625
Drosophila simulans FBgn0195102
Drosophila melanogaster FBgn0032291
Drosophila erecta FBgn0115812
Drosophila yakuba FBgn0235899
Drosophila ananassae FBgn0092846
Drosophila persimilis FBgn0164212
Drosophila pseudoobscura FBgn0074359
Drosophila virilis FBgn0207828
Drosophila grimshawi FBgn0117907
Drosophila mojavensis FBgn0146210
Drosophila willistoni FBgn0216674
Glossina palpalis GPPI022685
Glossina pallidipes GPAI006416
Glossina austeni GAUT009430
Musca domestica 17025377
Bactrocera cucurbitae 11854612
Bactrocera dorsalis 11786776
Megaselia scalaris MESCA008799
Tribolium castaneum TC014579
Rhyzobius pseudopulcher
Agrilus planipennis APLA003583
Anoplophora glabripennis AGLA001776
Pediculus humanus PHUM086940
Calopteryx splendens size615930.2
Cimex lectularius CLEC002393
Gerris buenoi GBUE001812
Anopheles gambiae AGAP006134
Anopheles quadriannulatus AQUA011686
Anopheles arabiensis AARA007388
Anopheles merus AMEM016150
Anopheles coluzzii ACOM024612
Anopheles melas AMEC021967
Anopheles christyi ACHR000473
Anopheles epiroticus AEPI005080
Anopheles minimus AMIN010680
Anopheles funestus AFUN008673
Anopheles stephensi ASTE001017
Anopheles dirus ADIR003802
Anopheles farauti AFAF003662
Anopheles darlingi ADAC002485
Anopheles albimanus AALB004845
Anopheles atroparvus AATE018438
Anopheles sinensis ASIC019893
Aedes aegypti AAEL010658
Polypedilum nubifer Pn.07150
Polypedilum vanderplanki Pv.00419
Mayetiola destructor Mdes005555
Mengenilla moldrzyki mesp 7049
Pachypsylla venusta PVEN005940
Homalodisca vitripennis HVIT024217
Ladona fulva LFUL011942
Diptera
Lepidoptera
Trichoptera
Coleoptera
Strepsiptera
Hymenoptera
Psocodea
Thysanoptera
Hemiptera
Blattodea

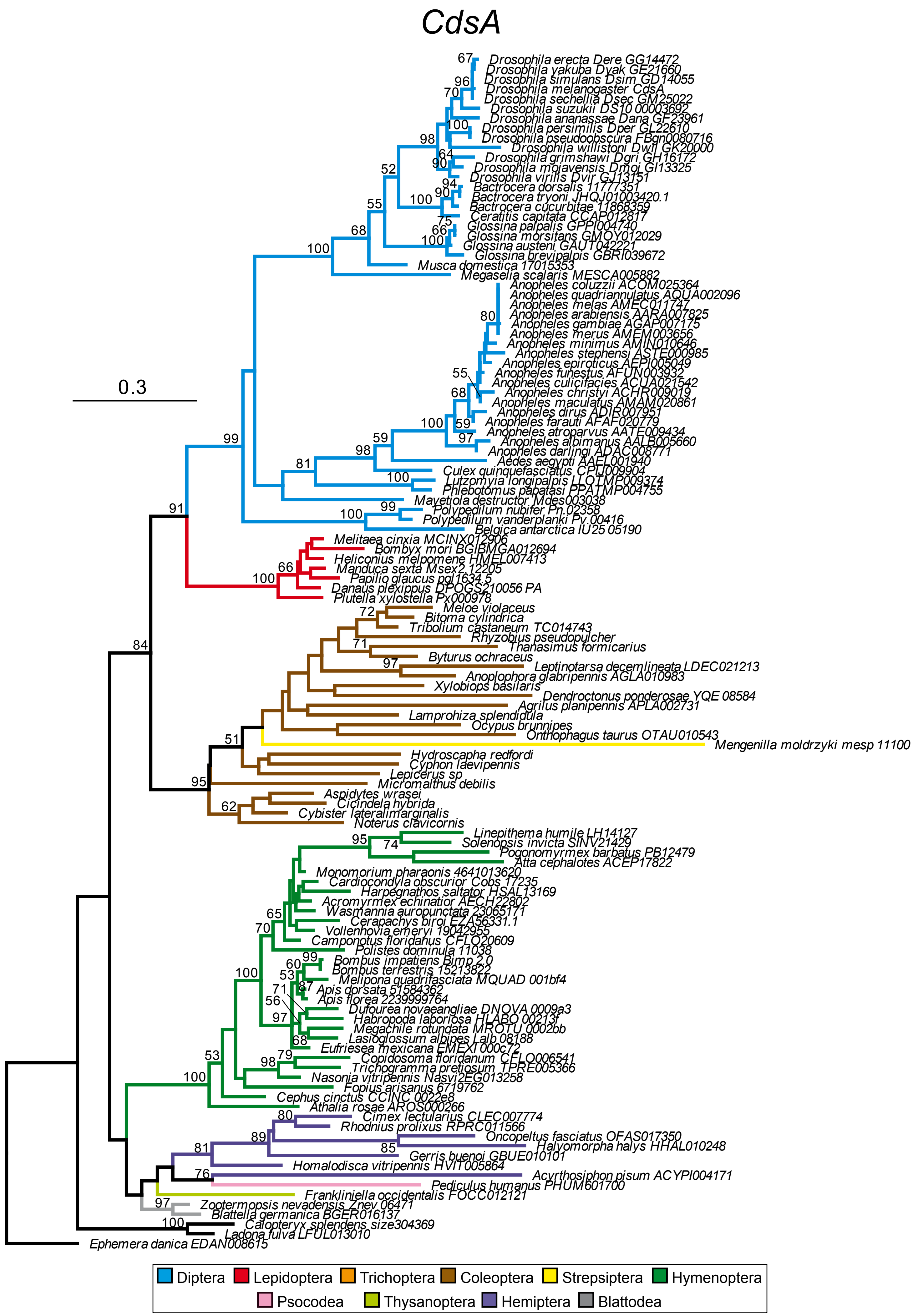
CdsA
0.3
Drosophila erecta Dere GG14472
Drosophila yakuba Dyak GE21660
Drosophila simulans Dsim GD14055
Drosophila melanogaster CdsA
Drosophila sechellia Dsec GM25022
Drosophila suzukii DS10 00003692
Drosophila ananassae Dana GF23961
Drosophila persimilis Dper GL22610
Drosophila pseudoobscura FBgn0080716
Drosophila willistoni Dwil GK20000
Drosophila grimshawi Dgri GH16172
Drosophila mojavensis Dmoj GI13325
Drosophila virilis Dvir GJ13151
Bactrocera dorsalis 11777351
Bactrocera tryoni JHQJ01003420.1
Bactrocera cucurbitae 11868359
Ceratitis capitata CCAP012817
Glossina palpalis GPPI004740
Glossina morsitans GMOY012029
Glossina austeni GAUT042221
Glossina brevipalpis GBRI039672
Musca domestica 17015353
Megaselia scalaris MESCA005882
Anopheles coluzzii ACOM025364
Anopheles quadriannulatus AQUA002096
Anopheles melas AMEC011747
Anopheles arabiensis AARA007825
Anopheles gambiae AGAP007175
Anopheles merus AMEM003656
Anopheles minimus AMIN010646
Anopheles stephensi ASTE000985
Anopheles epiroticus AEPI005049
Anopheles funestus AFUN003932
Anopheles culicifacies ACUA021542
Anopheles christyi ACHR009019
Anopheles maculatus AMAM020861
Anopheles dirus ADIR007951
Anopheles farauti AFAF020779
Anopheles atroparvus AATE009434
Anopheles albimanus AALB005660
Anopheles darlingi ADAC008771
Aedes aegypti AAEL001940
Culex quinquefasciatus CPIJ009904
Lutzomyia longipalpis LLOTMP009374
Phlebotomus papatasi PPATMP004755
Mayetiola destructor Mdes003038
Polypedilum nubifer Pn.02358
Polypedilum vanderplanki Pv.00416
Belgica antarctica IU25 05190
Melitaea cinxia MCINX012906
Bombyx mori BGIBMGA012694
Heliconius melpomene HMEL007413
Manduca sexta Msex2.12205
Papilio glaucus pgl1634.5
Danaus plexippus DPOGS210056 PA
Plutella xylostella Px000978
Meloe violaceus
Bitoma cylindrica
Tribolium castaneum TC014743
Rhyzobius pseudopulcher
Thanasimus formicarius
Byturus ochraceus
Leptinotarsa decemlineata LDEC021213
Anoplophora glabripennis AGLA010983
Xylobiops basilaris
Dendroctonus ponderosae YQE 08584
Agrilus planipennis APLA002731
Lamprohiza splendidula
Ocypus brunnipes
Onthophagus taurus OTAU010543
Mengenilla moldrzyki mesp 11100
Hydroscapha redfordi
Cyphon laevipennis
Lepicerus sp
Micromalthus debilis
Aspidytes wrasei
Cicindela hybrida
Cybister lateralimarginalis
Noterus clavicornis
Linepithema humile LH14127
Solenopsis invicta SINV21429
Pogonomyrmex barbatus PB12479
Atta cephalotes ACEP17822
Monomorium pharaonis 4641013620
Cardiocondyla obscurior Cobs 17235
Harpegnathos saltator HSAL13169
Acromyrmex echinatior AECH22802
Wasmannia auropunctata 23065171
Cerapachys biroi EZA56331.1
Vollenhovia emeryi 19042955
Camponotus floridanus CFLO20609
Polistes dominula 11038
Bombus impatiens Bimp 2.0
Bombus terrestris 15213822
Melipona quadrifasciata MQUAD 001bf4
Apis dorsata 51584362
Apis florea 2239999764
Dufourea novaeangliae DNOVA 0009a3
Habropoda laboriosa HLABO 00213f
Megachile rotundata MROTU 0002bb
Lasioglossum albipes Lalb 08188
Eufriesea mexicana EMEXI 000c72
Copidosoma floridanum CFLO006541
Trichogramma pretiosum TPRE005366
Nasonia vitripennis Nasvi2EG013258
Fopius arisanus 6719762
Cephus cinctus CCINC 0022e8
Athalia rosae AROS000266
Cimex lectularius CLEC007774
Rhodnius prolixus RPRC011566
Oncopeltus fasciatus OFAS017350
Halyomorpha halys HHAL010248
Gerris buenoi GBUE010101
Homalodisca vitripennis HVIT005864
Acyrthosiphon pisum ACYPI004171
Pediculus humanus PHUM601700
Frankliniella occidentalis FOCC012121
Zootermopsis nevadensis Znev 06471
Blattella germanica BGER016137
Calopteryx splendens size304369
Ladona fulva LFUL013010
Ephemera danica EDAN008615
Diptera
Lepidoptera
Trichoptera
Coleoptera
Strepsiptera
Hymenoptera
Psocodea
Thysanoptera
Hemiptera
Blattodea

Chc

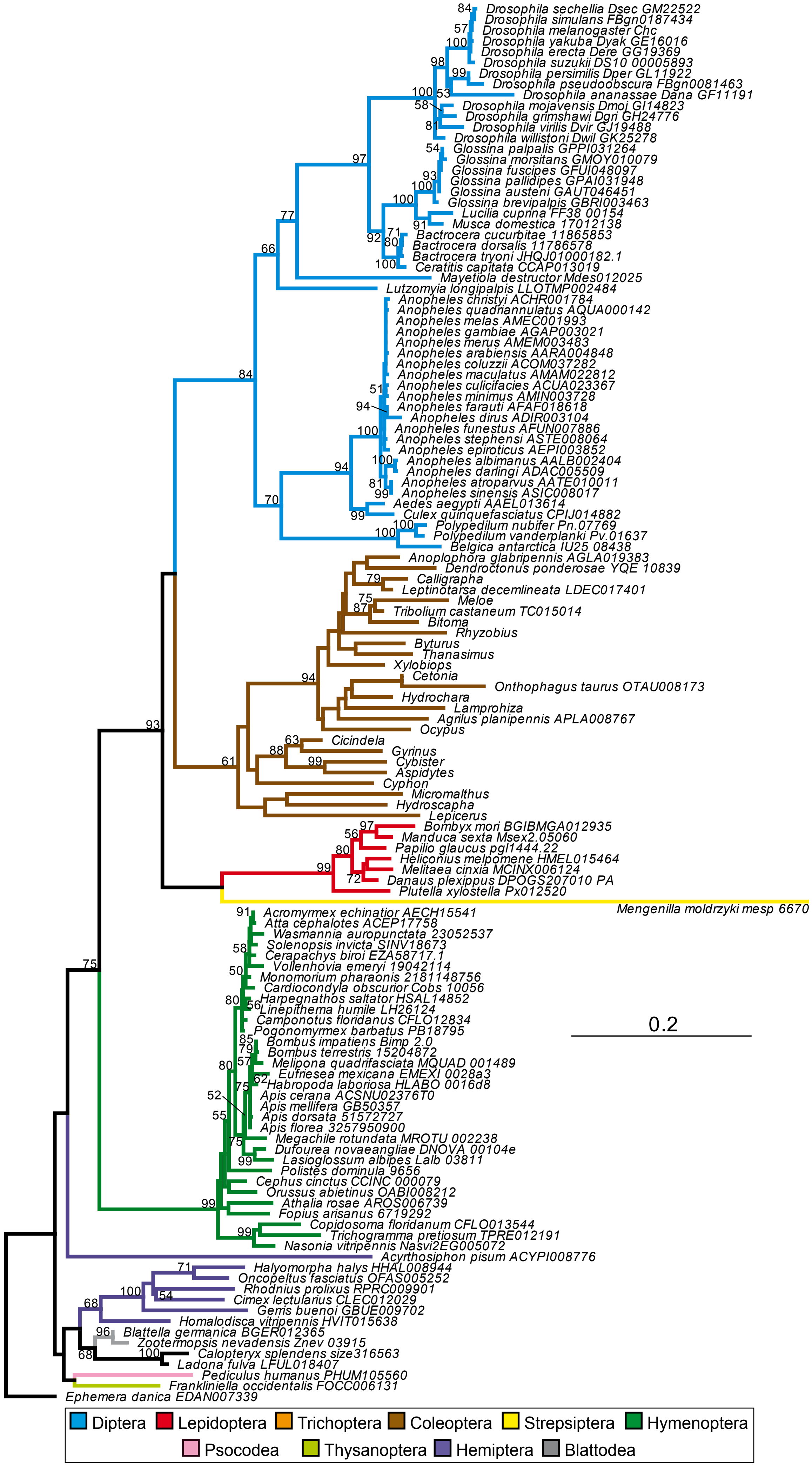
Drosophila sechellia Dsec GM22522
Drosophila simulans FBgn0187434
Drosophila melanogaster Chc
Drosophila yakuba Dyak GE16016
Drosophila erecta Dere GG19369
Drosophila suzukii DS10 00005893
Drosophila persimilis Dper GL11922
Drosophila pseudoobscura FBgn0081463
Drosophila ananassae Dana GF11191
Drosophila mojavensis Dmoj GI14823
Drosophila grimshawi Dgri GH24776
Drosophila virilis Dvir GJ19488
Drosophila willistoni Dwil GK25278
Glossina palpalis GPPI031264
Glossina morsitans GMOY010079
Glossina fuscipes GFUI048097
Glossina pallidipes GPAI031948
Glossina austeni GAUT046451
Glossina brevipalpis GBRI003463
Lucilia cuprina FF38 00154
Musca domestica 17012138
Bactrocera cucurbitae 11865853
Bactrocera dorsalis 11786578
Bactrocera tryoni JHQJ01000182.1
Ceratitis capitata CCAP013019
Mayetiola destructor Mdes012025
Lutzomyia longipalpis LLOTMP002484
Anopheles christyi ACHR001784
Anopheles quadriannulatus AQUA000142
Anopheles melas AMEC001993
Anopheles gambiae AGAP003021
Anopheles merus AMEM003483
Anopheles arabiensis AARA004848
Anopheles coluzzii ACOM037282
Anopheles maculatus AMAM022812
Anopheles culicifacies ACUA023367
Anopheles minimus AMIN003728
Anopheles farauti AFAF018618
Anopheles dirus ADIR003104
Anopheles funestus AFUN007886
Anopheles stephensi ASTE008064
Anopheles epiroticus AEPI003852
Anopheles albimanus AALB002404
Anopheles darlingi ADAC005509
Anopheles atroparvus AATE010011
Anopheles sinensis ASIC008017
Aedes aegypti AAEL013614
Culex quinquefasciatus CPIJ014882
Polypedilum nubifer Pn.07769
Polypedilum vanderplanki Pv.01637
Belgica antarctica IU25 08438
Anoplophora glabripennis AGLA019383
Dendroctonus ponderosae YQE 10839
Calligrapha
Leptinotarsa decemlineata LDEC017401
Meloe
Tribolium castaneum TC015014
Bitoma
Rhyzobius
Byturus
Thanasimus
Xylobiops
Cetonia
Onthophagus taurus OTAU008173
Hydrochara
Lamprohiza
Agrilus planipennis APLA008767
Ocypus
Cicindela
Gyrinus
Cybister
Aspidytes
Cyphon
Micromalthus
Hydroscapha
Lepicerus
Bombyx mori BGIBMGA012935
Manduca sexta Msex2.05060
Papilio glaucus pgl1444.22
Heliconius melpomene HMEL015464
Melitaea cinxia MCINX006124
Danaus plexippus DPOGS207010 PA
Plutella xylostella Px012520
Mengenilla moldrzyki mesp 6670
Acromyrmex echinatior AECH15541
Atta cephalotes ACEP17758
Wasmannia auropunctata 23052537
Solenopsis invicta SINV18673
Cerapachys biroi EZA58717.1
Vollenhovia emeryi 19042114
Monomorium pharaonis 2181148756
Cardiocondyla obscurior Cobs 10056
Harpegnathos saltator HSAL14852
Linepithema humile LH26124
Camponotus floridanus CFLO12834
Pogonomyrmex barbatus PB18795
Bombus impatiens Bimp 2.0
Bombus terrestris 15204872
Melipona quadrifasciata MQUAD 001489
Eufriesea mexicana EMEXI 0028a3
Habropoda laboriosa HLABO 0016d8
Apis cerana ACSNU02376T0
Apis mellifera GB50357
Apis dorsata 51572727
Apis florea 3257950900
Megachile rotundata MROTU 002238
Dufourea novaeangliae DNOVA 00104e
Lasioglossum albipes Lalb 03811
Polistes dominula 9656
Cephus cinctus CCINC 000079
Orussus abietinus OABI008212
Athalia rosae AROS006739
Fopius arisanus 6719292
Copidosoma floridanum CFLO013544
Trichogramma pretiosum TPRE012191
Nasonia vitripennis Nasvi2EG005072
Acyrthosiphon pisum ACYPI008776
Halyomorpha halys HHAL008944
Oncopeltus fasciatus OFAS005252
Rhodnius prolixus RPRC009901
Cimex lectularius CLEC012029
Gerris buenoi GBUE009702
Homalodisca vitripennis HVIT015638
Blattella germanica BGER012365
Zootermopsis nevadensis Znev 03915
Calopteryx splendens size316563
Ladona fulva LFUL018407
Pediculus humanus PHUM105560
Frankliniella occidentalis FOCC006131
Ephemera danica EDAN007339
0.2
Diptera
Lepidoptera
Trichoptera
Coleoptera
Strepsiptera
Hymenoptera
Psocodea
Thysanoptera
Hemiptera
Blattodea

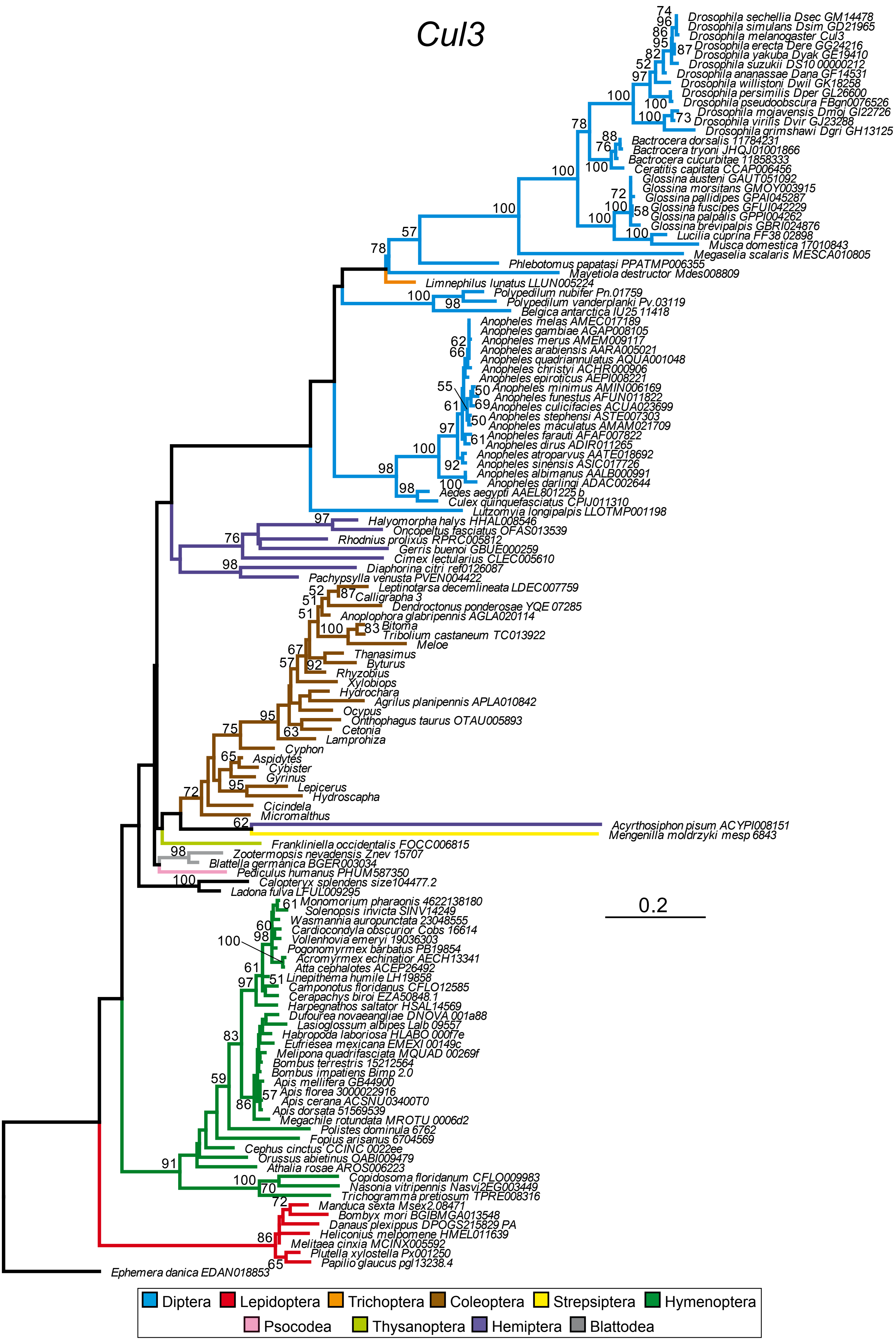

Cul3
Drosophila sechellia Dsec GM14478
Drosophila simulans Dsim GD21965
Drosophila melanogaster Cul3
Drosophila erecta Dere GG24216
Drosophila yakuba Dyak GE19410
Drosophila suzukii DS10 00000212
Drosophila ananassae Dana GF14531
Drosophila willistoni Dwil GK18258
Drosophila persimilis Dper GL26600
Drosophila pseudoobscura FBgn0076526
Drosophila mojavensis Dmoj GI22726
Drosophila virilis Dvir GJ23288
Drosophila grimshawi Dgri GH13125
Bactrocera dorsalis 11784231
Bactrocera tryoni JHQJ01001866
Bactrocera cucurbitae 11858333
Ceratitis capitata CCAP006456
Glossina austeni GAUT051092
Glossina morsitans GMOY003915
Glossina pallidipes GPAI045287
Glossina fuscipes GFUI042229
Glossina palpalis GPPI004262
Glossina brevipalpis GBRI024876
Lucilia cuprina FF38 02898
Musca domestica 17010843
Megaselia scalaris MESCA010805
Phlebotomus papatasi PPATMP006355
Mayetiola destructor Mdes008809
Limnephilus lunatus LLUN005224
Polypedilum nubifer Pn.01759
Polypedilum vanderplanki Pv.03119
Belgica antarctica IU25 11418
Anopheles melas AMEC017189
Anopheles gambiae AGAP008105
Anopheles merus AMEM009117
Anopheles arabiensis AARA005021
Anopheles quadriannulatus AQUA001048
Anopheles christyi ACHR000906
Anopheles epiroticus AEPI008221
Anopheles minimus AMIN006169
Anopheles funestus AFUN011822
Anopheles culicifacies ACUA023699
Anopheles stephensi ASTE007303
Anopheles maculatus AMAM021709
Anopheles farauti AFAF007822
Anopheles dirus ADIR011265
Anopheles atroparvus AATE018692
Anopheles sinensis ASIC017726
Anopheles albimanus AALB000991
Anopheles darlingi ADAC002644
Aedes aegypti AAEL801225 b
Culex quinquefasciatus CPIJ011310
Lutzomyia longipalpis LLOTMP001198
Halyomorpha halys HHAL008546
Oncopeltus fasciatus OFAS013539
Rhodnius prolixus RPRC005812
Gerris buenoi GBUE000259
Cimex lectularius CLEC005610
Diaphorina citri ref0126087
Pachypsylla venusta PVEN004422
Leptinotarsa decemlineata LDEC007759
Calligrapha 3
Dendroctonus ponderosae YQE 07285
Anoplophora glabripennis AGLA020114
Bitoma
Tribolium castaneum TC013922
Meloe
Thanasimus
Byturus
Rhyzobius
Xylobiops
Hydrochara
Agrilus planipennis APLA010842
Ocypus
Onthophagus taurus OTAU005893
Cetonia
Lamprohiza
Cyphon
Aspidytes
Cybister
Gyrinus
Lepicerus
Hydroscapha
Cicindela
Micromalthus
Acyrthosiphon pisum ACYPI008151
Mengenillia moldrzyki mesp 6843
Frankliniella occidentalis FOCC006815
Zootermopsis nevadensis Znev 15707
Blattella germanica BGER003034
Pediculus humanus PHUM587350
Calopteryx splendens size104477.2
Ladona fulva LFUL009295
0.2
Monomorium pharaonis 4622138180
Solenopsis invicta SINV14249
Wasmannia auropunctata 23048555
Cardiocondyla obscurior Cobs 16614
Vollenhovia emeryi 19036303
Pogonomyrmex barbatus PB19854
Acromyrmex echinatior AECH13341
Atta cephalotes ACEP26492
Linepithema humile LH19858
Camponotus floridanus CFLO12585
Cerapachys biroi EZA50848.1
Harpegnathos saltator HSAL14569
Dufourea novaeangliae DNOVA 001a88
Lasioglossum albipes Lalb 09557
Habropoda laboriosa HLABO 000f7e
Eufriesea mexicana EMEXI 00149c
Melipona quadrifasciata MQUAD 00269f
Bombus terrestris 15212564
Bombus impatiens Bimp 2.0
Apis mellifera GB44900
Apis florea 3000022916
Apis cerana ACSNU03400T0
Apis dorsata 51569539
Megachile rotundata MROTU 0006d2
Polistes dominula 6762
Fopius arisanus 6704569
Cephus cinctus CCINC 0022ee
Orussus abietinus OABI009479
Athalia rosae AROS006223
Copidosoma floridanum CFLO009983
Nasonia vitripennis Nasvi2EG003449
Trichogramma pretiosum TPRE008316
Manduca sexta Msex2.08471
Bombyx mori BGIBMGA013548
Danaus plexippus DPOGS215829 PA
Heliconius melpomene HMEL011639
Melitaea cinxia MCINX005592
Plutella xylostella Px001250
Papilio glaucus pgl13238.4
Ephemera danica EDAN018853
Diptera
Lepidoptera
Trichoptera
Coleoptera
Strepsiptera
Hymenoptera
Psocodea
Thysanoptera
Hemiptera
Blattodea

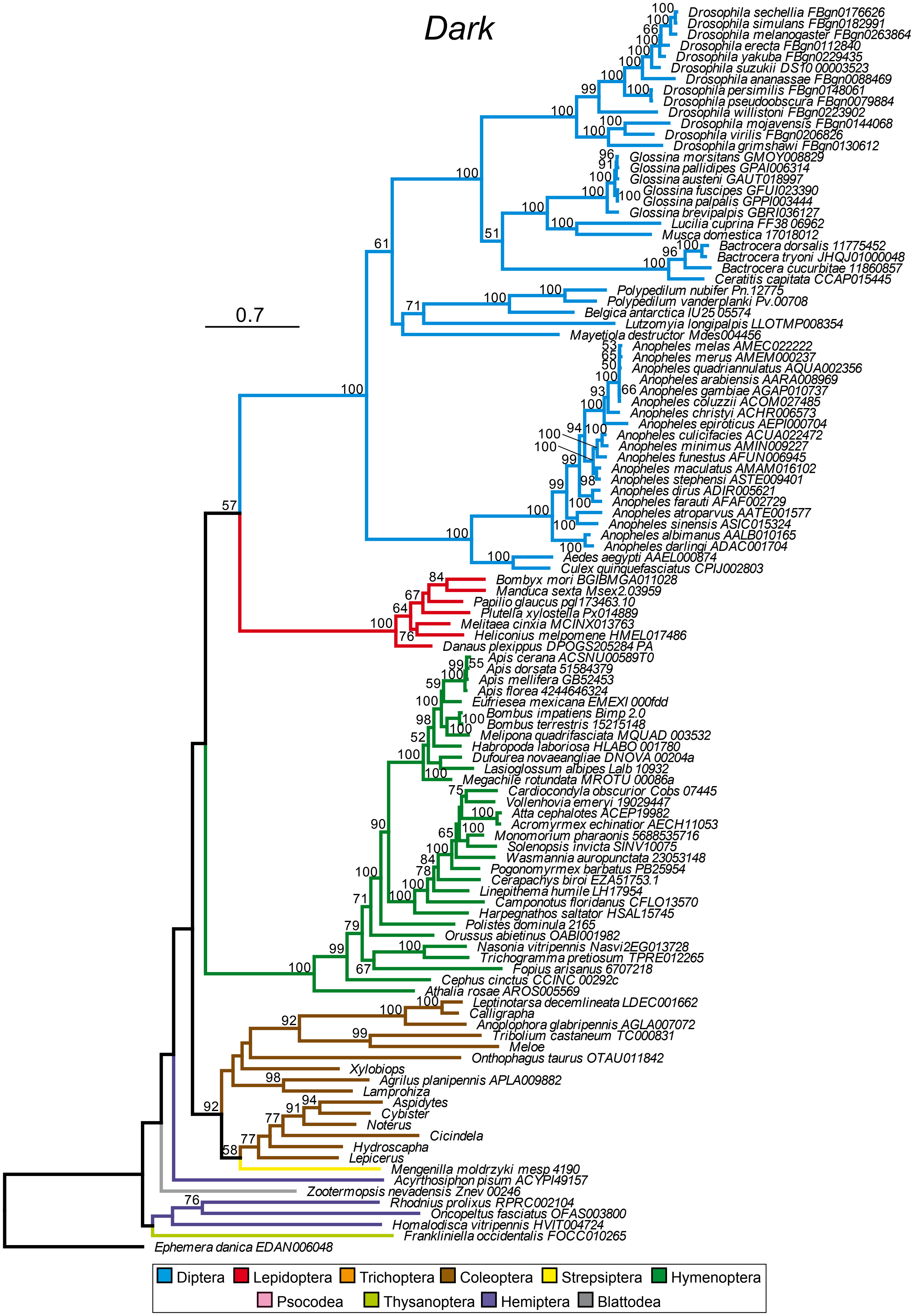
Dark
0.7
Drosophila sechellia FBgn0176626
Drosophila simulans FBgn0182991
Drosophila melanogaster FBgn0263864
Drosophila erecta FBgn0112840
Drosophila yakuba FBgn0229435
Drosophila suzukii DS10 00003523
Drosophila ananassae FBgn0088469
Drosophila persimilis FBgn0148061
Drosophila pseudoobscura FBgn0079884
Drosophila willistoni FBgn0223902
Drosophila mojavensis FBgn0144068
Drosophila virilis FBgn0206826
Drosophila grimshawi FBgn0130612
Glossina morsitans GMOY008829
Glossina pallidipes GPAI006314
Glossina austeni GAUT018997
Glossina fuscipes GFUI023390
Glossina palpalis GPPI003444
Glossina brevipalpis GBRI036127
Lucilia cuprina FF38 06962
Musca domestica 17018012
Bactrocera dorsalis 11775452
Bactrocera tryoni JHQJ01000048
Bactrocera cucurbitae 11860857
Ceratitis capitata CCAP015445
Polypedilum nubifer Pn.12775
Polypedilum vanderplanki Pv.00708
Belgica antarctica IU25 05574
Lutzomyia longipalpis LLOTMP008354
Mayetiola destructor Mdes004456
Anopheles melas AMEC022222
Anopheles merus AMEM000237
Anopheles quadriannulatus AQUA002356
Anopheles arabiensis AARA008969
Anopheles gambiae AGAP010737
Anopheles coluzzii ACOM027485
Anopheles christyi ACHR006573
Anopheles epiroticus AEPI000704
Anopheles culicifacies ACUA022472
Anopheles minimus AMIN009227
Anopheles funestus AFUN006945
Anopheles maculatus AMAM016102
Anopheles stephensi ASTE009401
Anopheles dirus ADIR005621
Anopheles farauti AFAF002729
Anopheles atroparvus AATE001577
Anopheles sinensis ASIC015324
Anopheles albimanus AALB010165
Anopheles darlingi ADAC001704
Aedes aegypti AAEL000874
Culex quinquefasciatus CPIJ002803
Bombyx mori BGIBMGA011028
Manduca sexta Msex2.03959
Papilio glaucus pgl173463.10
Plutella xylostella Px014889
Melitaea cinxia MCINX013763
Heliconius melpomene HMEL017486
Danaus plexippus DPOGS205284 PA
Apis cerana ACSNU00589T0
Apis dorsata 51584379
Apis mellifera GB52453
Apis florea 4244646324
Eufriesea mexicana EMEXI 000fdd
Bombus impatiens Bimp 2.0
Bombus terrestris 15215148
Melipona quadrifasciata MQUAD 003532
Habropoda laboriosa HLABO 001780
Dufourea novaeangliae DNOVA 00204a
Lasioglossum albipes Lalb 10932
Megachile rotundata MROTU 00086a
Cardiocondyla obscurior Cobs 07445
Vollenhovia emeryi 19029447
Atta cephalotes ACEP19982
Acromyrmex echinatior AECH11053
Monomorium pharaonis 5688535716
Solenopsis invicta SINV10075
Wasmannia auropunctata 23053148
Pogonomyrmex barbatus PB25954
Cerapachys biroi EZA51753.1
Linepithema humile LH17954
Camponotus floridanus CFLO13570
Harpegnathos saltator HSAL15745
Polistes dominula 2165
Orussus abietinus OABI001982
Nasonia vitripennis Nasvi2EG013728
Trichogramma pretiosum TPRE012265
Fopius arisanus 6707218
Cephus cinctus CCINC 00292c
Athalia rosae AROS005569
Leptinotarsa decemlineata LDEC001662
Calligrapha
Anoplophora glabripennis AGLA007072
Tribolium castaneum TC000831
Meloe
Onthophagus taurus OTAU011842
Xylobiops
Agrilus planipennis APLA009882
Lamprohiza
Aspidytes
Cybister
Noterus
Cicindela
Hydroscapha
Lepicerus
Mengenilla moldrzyki mesp 4190
Acyrthosiphon pisum ACYPI49157
Zootermopsis nevadensis Znev 00246
Rhodnius prolixus RPRC002104
Oncopeltus fasciatus OFAS003800
Homalodisca vitripennis HVIT004724
Frankliniella occidentalis FOCC010265
Ephemera danica EDAN006048
Diptera
Lepidoptera
Trichoptera
Coleoptera
Strepsiptera
Hymenoptera
Psocodea
Thysanoptera
Hemiptera
Blattodea

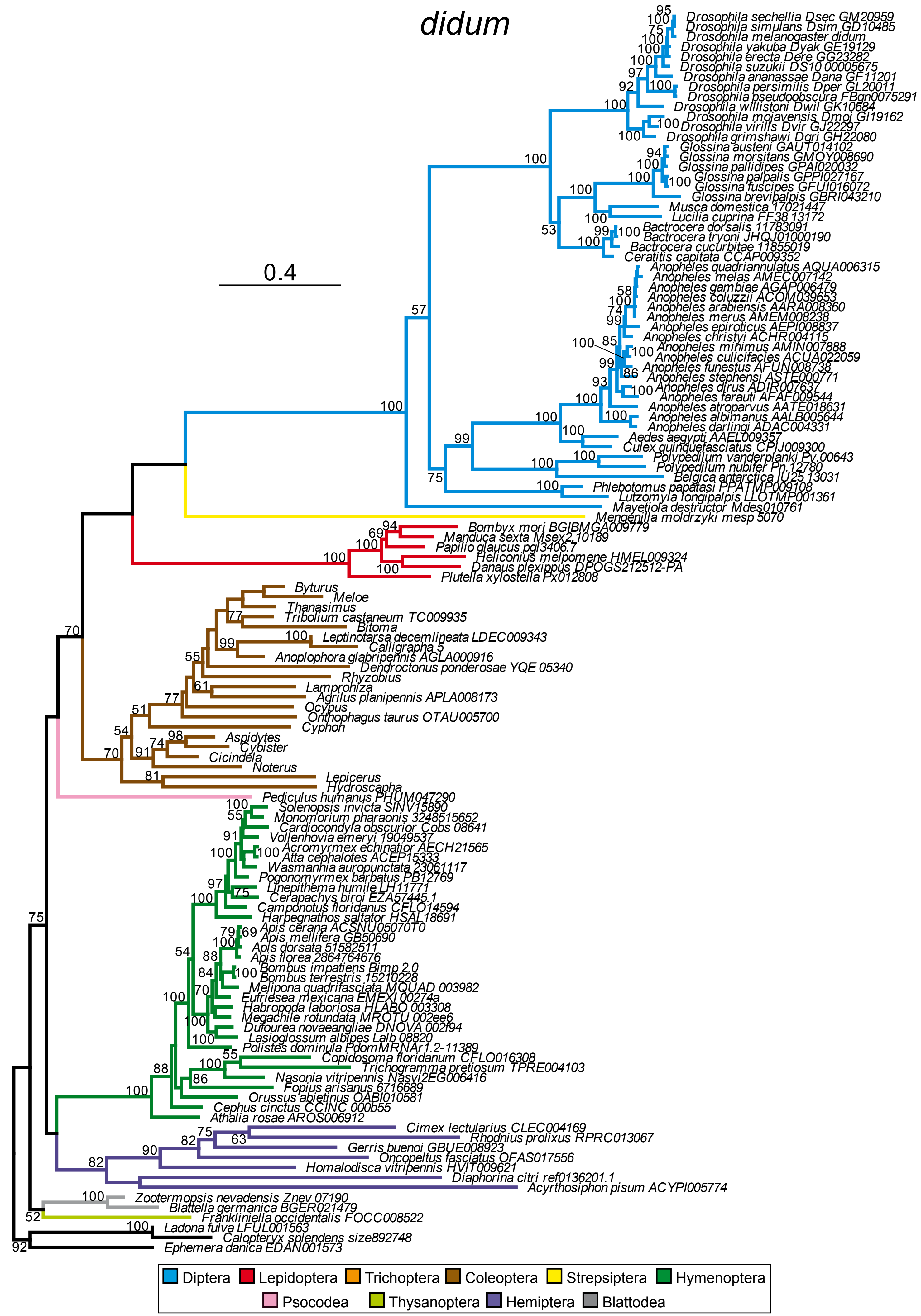
didum
0.4
Drosophila sechellia Dsec GM20959
Drosophila simulans Dsim GD10485
Drosophila melanogaster didum
Drosophila yakuba Dyak GE19129
Drosophila erecta Dere GG23282
Drosophila suzukii DS10 00005675
Drosophila ananassae Dana GF11201
Drosophila persimilis Dper GL20011
Drosophila pseudoobscura FBgn0075291
Drosophila willistoni Dwil GK10684
Drosophila mojavensis Dmoj GI19162
Drosophila virilis Dvir GJ22297
Drosophila grimshawi Dgri GH22080
Glossina austeni GAUT014102
Glossina morsitans GMOY008690
Glossina pallidipes GPAI020032
Glossina palpalis GPPI027167
Glossina fuscipes GFUI016072
Glossina brevipalpis GBRI043210
Musca domestica 17021447
Lucilia cuprina FF38 13172
Bactrocera dorsalis 11783091
Bactrocera tryoni JHQJ01000190
Bactrocera cucurbitae 11855019
Ceratitis capitata CCAP009352
Anopheles quadriannulatus AQUA006315
Anopheles melas AMEC007142
Anopheles gambiae AGAP006479
Anopheles coluzzii ACOM039653
Anopheles arabiensis AARA008360
Anopheles merus AMEM008238
Anopheles epiroticus AEPI008837
Anopheles christyi ACHR004115
Anopheles minimus AMIN007888
Anopheles culicifacies ACUA022059
Anopheles funestus AFUN008738
Anopheles stephensi ASTE000771
Anopheles dirus ADIR007637
Anopheles farauti AFAF009544
Anopheles atroparvus AATE018631
Anopheles albimanus AALB005644
Anopheles darlingi ADAC004331
Aedes aegypti AAEL009357
Culex quinquefasciatus CPIJ009300
Polypedilum vanderplanki Pv.00643
Polypedilum nubifer Pn.12780
Belgica antarctica IU25 13031
Phlebotomus papatasi PPATMP009108
Lutzomyia longipalpis LLOTMP001361
Mayetiola destructor Mdes010761
Mengenilla moldrzyki mesp 5070
Bombyx mori BGIBMGA009779
Manduca sexta Msex2.10189
Papilio glaucus pgl3406.7
Heliconius melpomene HMEL009324
Danaus plexippus DPOGS212512-PA
Plutella xylostella Px012808
Byturus
Meloe
Thanasimus
Tribolium castaneum TC009935
Bitoma
Leptinotarsa decemlineata LDEC009343
Calligrapha 5
Anoplophora glabripennis AGLA000916
Dendroctonus ponderosae YQE 05340
Rhyzobius
Lamprohiza
Agrilus planipennis APLA008173
Ocypus
Onthophagus taurus OTAU005700
Cyphon
Aspidytes
Cybister
Cicindela
Noterus
Lepicerus
Hydroscapha
Pediculus humanus PHUM047290
Solenopsis invicta SINV15890
Monomorium pharaonis 3248515652
Cardiocondyla obscurior Cobs 08641
Vollenhovia emeryi 19049537
Acromyrmex echinatior AECH21565
Atta cephalotes ACEP15333
Wasmannia auropunctata 23061117
Pogonomyrmex barbatus PB12769
Linepithema humile LH11771
Cerapachys biroi EZA57445.1
Camponotus floridanus CFLO14594
Harpegnathos saltator HSAL18691
Apis cerana ACSNU0507070
Apis mellifera GB50690
Apis dorsata 51582511
Apis florea 2864764676
Bombus impatiens Bimp 2.0
Bombus terrestris 15210228
Melipona quadrifasciata MQUAD 003982
Eufriesea mexicana EMEXI 00274a
Habropoda laboriosa HLABO 003308
Megachile rotundata MROTU 002ee6
Dufourea novaeangliae DNOVA 002f94
Lasioglossum albipes Lalb 08820
Polistes dominula PdomMRNAr1.2-11389
Copidosoma floridanum CFLO016308
Trichogramma pretiosum TPRE004103
Nasonia vitripennis Nasvi2EG006416
Fopius arisanus 6716689
Orussus abietinus OABI010581
Cephus cinctus CCINC 000b55
Athalia rosae AROS006912
Cimex lectularius CLEC004169
Rhodnius prolixus RPRC013067
Gerris buenoi GBUE008923
Oncopeltus fasciatus OFAS017556
Homalodisca vitripennis HVIT009621
Diaphorina citri ref0136201.1
Acyrthosiphon pisum ACYPI005774
Zootermopsis nevadensis Znev 07190
Blattella germanica BGER021479
Frankliniella occidentalis FOCC008522
Ladona fulva LFUL001563
Calopteryx splendens size892748
Ephemera danica EDAN001573
Diptera
Lepidoptera
Trichoptera
Coleoptera
Strepsiptera
Hymenoptera
Psocodea
Thysanoptera
Hemiptera
Blattodea

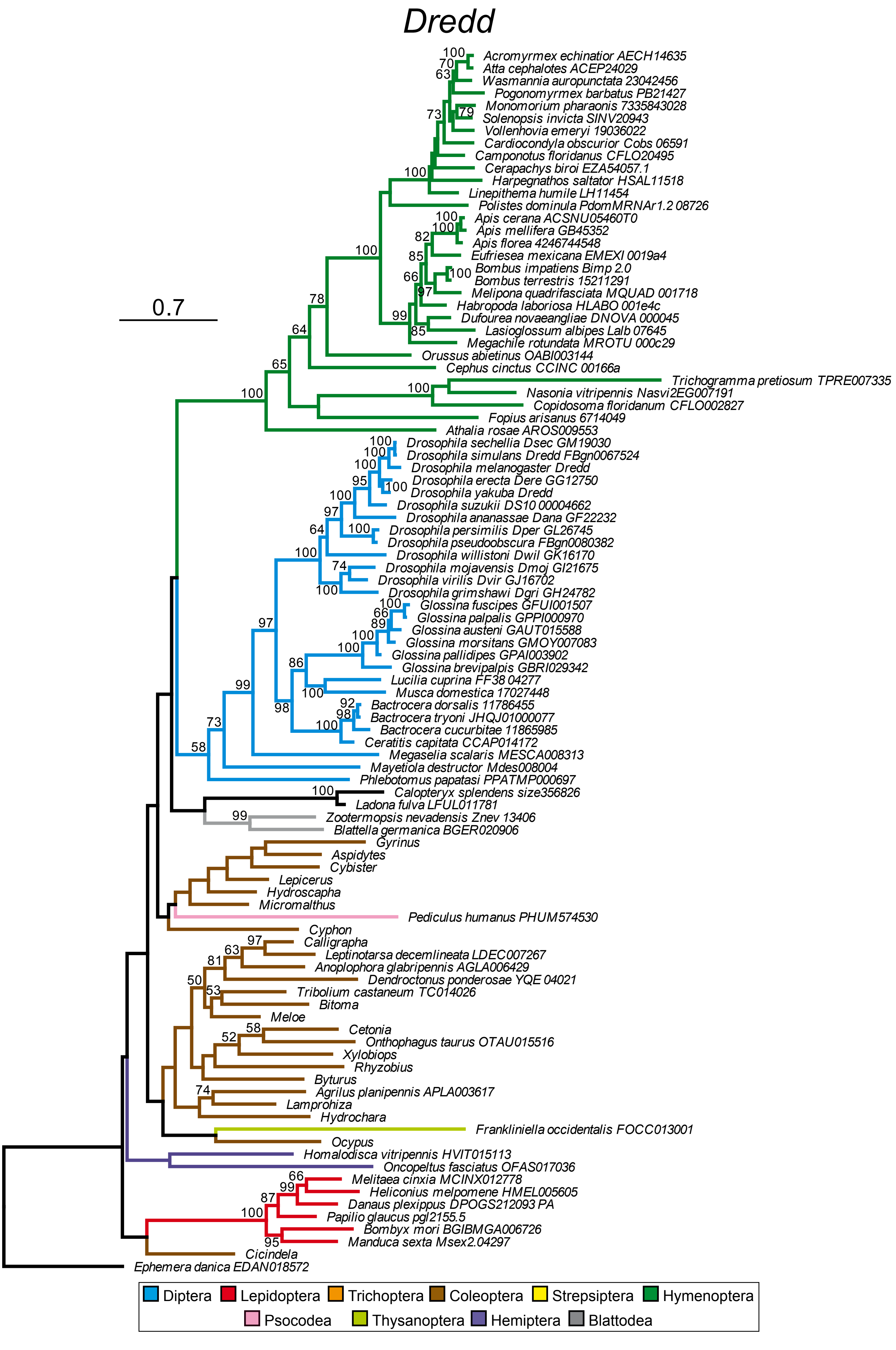
Dredd
0.7
Acromyrmex echinatior AECH14635
Atta cephalotes ACEP24029
Wasmannia auropunctata 23042456
Pogonomyrmex barbatus PB21427
Monomorium pharaonis 7335843028
Solenopsis invicta SINV20943
Vollenhovia emeryi 19036022
Cardiocondyla obscurior Cobs 06591
Camponotus floridanus CFLO20495
Cerapachys biroi EZA54057.1
Harpegnathos saltator HSAL11518
Linepithema humile LH11454
Polistes dominula PdomMRNAr1.2 08726
Apis cerana ACSNU05460T0
Apis mellifera GB45352
Apis florea 4246744548
Eufriesea mexicana EMEXI 0019a4
Bombus impatiens Bimp 2.0
Bombus terrestris 15211291
Melipona quadrifasciata MQUAD 001718
Habropoda laboriosa HLABO 001e4c
Dufourea novaeangliae DNOVA 000045
Lasioglossum albipes Lalb 07645
Megachile rotundata MROTU 000c29
Orussus abietinus OABI003144
Cephus cinctus CCINC 00166a
Trichogramma pretiosum TPRE007335
Nasonia vitripennis Nasvi2EG007191
Copidosoma floridanum CFLO002827
Fopius arisanus 6714049
Athalia rosae AROS009553
Drosophila sechellia Dsec GM19030
Drosophila simulans Dredd FBgn0067524
Drosophila melanogaster Dredd
Drosophila erecta Dere GG12750
Drosophila yakuba Dredd
Drosophila suzukii DS10 00004662
Drosophila ananassae Dana GF22232
Drosophila persimilis Dper GL26745
Drosophila pseudoobscura FBgn0080382
Drosophila willistoni Dwil GK16170
Drosophila mojavensis Dmoj GI21675
Drosophila virilis Dvir GJ16702
Drosophila grimshawi Dgri GH24782
Glossina fuscipes GFUI001507
Glossina palpalis GPPI000970
Glossina austeni GAUT015588
Glossina morsitans GMOY007083
Glossina pallidipes GPAI003902
Glossina brevipalpis GBRI029342
Lucilia cuprina FF38 04277
Musca domestica 17027448
Bactrocera dorsalis 11786455
Bactrocera tryoni JHQJ01000077
Bactrocera cucurbitae 11865985
Ceratitis capitata CCAP014172
Megaselia scalaris MESCA008313
Mayetiola destructor Mdes008004
Phlebotomus papatasi PPATMP000697
Calopteryx splendens size356826
Ladona fulva LFUL011781
Zootermopsis nevadensis Znev 13406
Blattella germanica BGER020906
Gyrinus
Aspidytes
Cybister
Lepicerus
Hydroscapha
Micromalthus
Pediculus humanus PHUM574530
Cyphon
Calligrapha
Leptinotarsa decemlineata LDEC007267
Anoplophora glabripennis AGLA006429
Dendroctonus ponderosae YQE 04021
Tribolium castaneum TC014026
Bitoma
Meloe
Cetonia
Onthophagus taurus OTAU015516
Xylobiops
Rhyzobius
Byturus
Agrilus planipennis APLA003617
Lamprohiza
Hydrochara
Frankliniella occidentalis FOCC013001
Ocypus
Homalodisca vitripennis HVIT015113
Oncopeltus fasciatus OFAS017036
Melitaea cinxia MCINX012778
Heliconius melpomene HMEL005605
Danaus plexippus DPOGS212093 PA
Papilio glaucus pgl2155.5
Bombyx mori BGIBMGA006726
Manduca sexta Msex2.04297
Cicindela
Ephemera danica EDAN018572
Diptera
Lepidoptera
Trichoptera
Coleoptera
Strepsiptera
Hymenoptera
Psocodea
Thysanoptera
Hemiptera
Blattodea

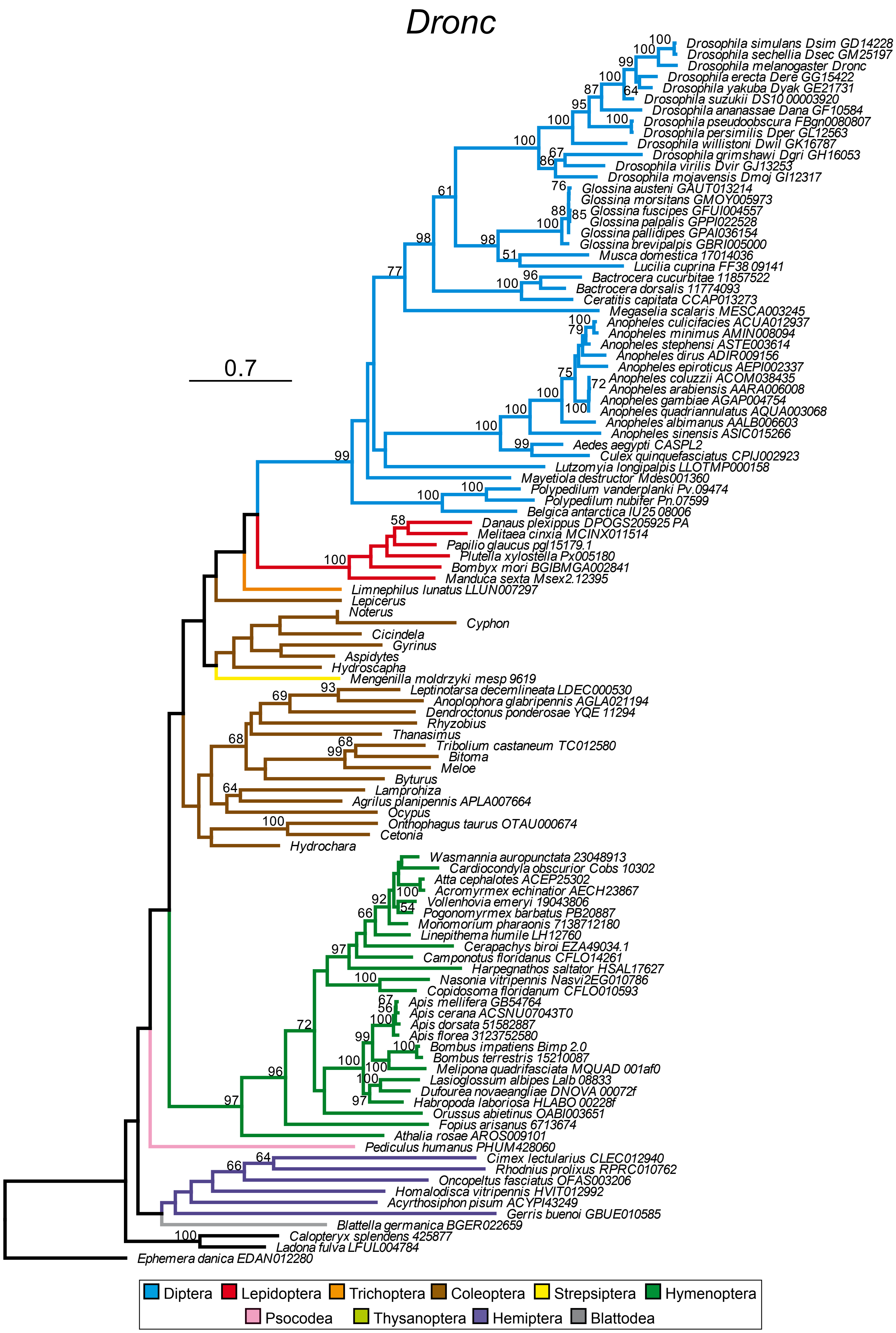

Dronc
0.7
Drosophila simulans Dsim GD14228
Drosophila sechellia Dsec GM25197
Drosophila melanogaster Dronc
Drosophila erecta Dere GG15422
Drosophila yakuba Dyak GE21731
Drosophila suzukii DS10 00003920
Drosophila ananassae Dana GF10584
Drosophila pseudoobscura FBgn0080807
Drosophila persimilis Dper GL12563
Drosophila willistoni Dwil GK16787
Drosophila grimshawi Dgri GH16053
Drosophila virilis Dvir GJ13253
Drosophila mojavensis Dmoj GI12317
Glossina austeni GAUT013214
Glossina morsitans GMOY005973
Glossina fuscipes GFUI004557
Glossina palpalis GPPI022528
Glossina pallidipes GPAI036154
Glossina brevipalpis GBRI005000
Musca domestica 17014036
Lucilia cuprina FF38 09141
Bactrocera cucurbitae 11857522
Bactrocera dorsalis 11774093
Ceratitis capitata CCAP013273
Megaselia scalaris MESCA003245
Anopheles culicifacies ACUA012937
Anopheles minimus AMIN008094
Anopheles stephensi ASTE003614
Anopheles dirus ADIR009156
Anopheles epiroticus AEPI002337
Anopheles coluzzii ACOM038435
Anopheles arabiensis AARA006008
Anopheles gambiae AGAP004754
Anopheles quadriannulatus AQUA003068
Anopheles albimanus AALB006603
Anopheles sinensis ASIC015266
Aedes aegypti CASPL2
Culex quinquefasciatus CPIJ002923
Lutzomyia longipalpis LLOTMP000158
Mayetiola destructor Mdes001360
Polypedilum vanderplanki Pv.09474
Polypedilum nubifer Pn.07599
Belgica antarctica IU25 08006
Danaus plexippus DPOGS205925 PA
Melitaea cinxia MCINX011514
Papilio glaucus pgl15179.1
Plutella xylostella Px005180
Bombyx mori BGIBMGA002841
Manduca sexta Msex2.12395
Limnephilus lunatus LLUN007297
Lepicerus
Noterus
Cyphon
Cicindela
Gyrinus
Aspidytes
Hydroscapha
Mengenilla moldrzyki mesp 9619
Leptinotarsa decemlineata LDEC000530
Anoplophora glabripennis AGLA021194
Dendroctonus ponderosae YQE 11294
Rhyzobius
Thanasimus
Tribolium castaneum TC012580
Bitoma
Meloe
Byturus
Lamprohiza
Agrilus planipennis APLA007664
Ocypus
Onthophagus taurus OTAU000674
Cetonia
Hydrochara
Wasmannia auropunctata 23048913
Cardiocondyla obscurior Cobs 10302
Atta cephalotes ACEP25302
Acromyrmex echinatior AECH23867
Vollenhovia emeryi 19043806
Pogonomyrmex barbatus PB20887
Monomorium pharaonis 7138712180
Linepithema humile LH12760
Cerapachys biroi EZA49034.1
Camponotus floridanus CFLO14261
Harpegnathos saltator HSAL17627
Nasonia vitripennis Nasvi2EG010786
Copidosoma floridanum CFLO010593
Apis mellifera GB54764
Apis cerana ACSNU07043T0
Apis dorsata 51582887
Apis florea 3123752580
Bombus impatiens Bimp 2.0
Bombus terrestris 15210087
Melipona quadrifasciata MQUAD 001af0
Lasioglossum albipes Lalb 08833
Dufourea novaeangliae DNOVA 00072f
Habropoda laboriosa HLABO 00228f
Orussus abietinus OABI003651
Fopius arisanus 6713674
Athalia rosae AROS009101
Pediculus humanus PHUM428060
Cimex lectularius CLEC012940
Rhodnius prolixus RPRC010762
Oncopeltus fasciatus OFAS003206
Homalodisca vitripennis HVIT012992
Acyrthosiphon pisum ACYPI43249
Gerris buenoi GBUE010585
Blattella germanica BGER022659
Calopteryx splendens 425877
Ladona fulva LFUL004784
Ephemera danica EDAN012280
Diptera
Lepidoptera
Trichoptera
Coleoptera
Strepsiptera
Hymenoptera
Psocodea
Thysanoptera
Hemiptera
Blattodea

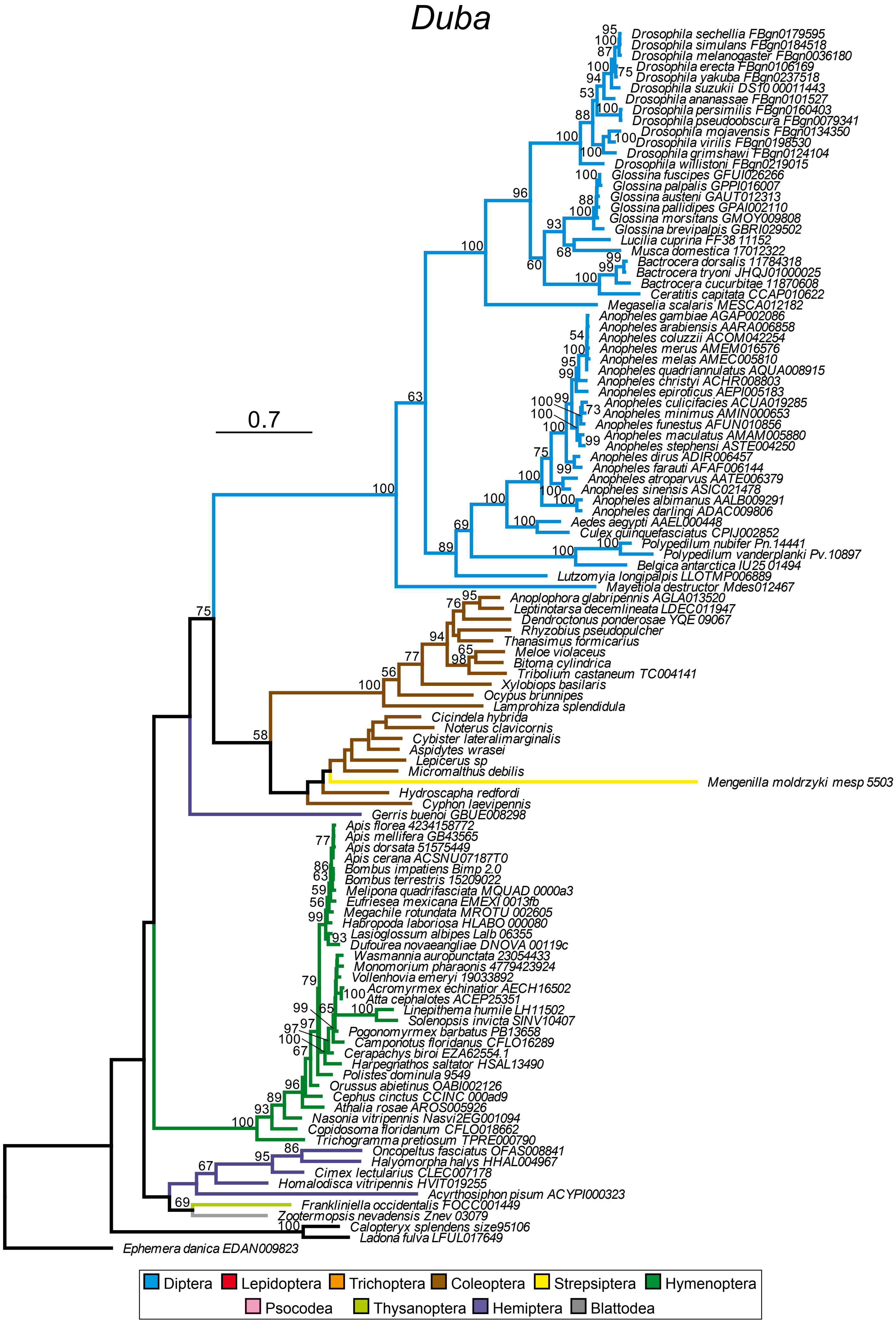

Duba
0.7
Drosophila sechellia FBgn0179595
Drosophila simulans FBgn0184518
Drosophila melanogaster FBgn0036180
Drosophila erecta FBgn0106169
Drosophila yakuba FBgn0237518
Drosophila suzukii DS10 00011443
Drosophila ananassae FBgn0101527
Drosophila persimilis FBgn0160403
Drosophila pseudoobscura FBgn0079341
Drosophila mojavensis FBgn0134350
Drosophila virilis FBgn0198530
Drosophila grimshawi FBgn0124104
Drosophila willistoni FBgn0219015
Glossina fuscipes GFUI026266
Glossina palpalis GPPI016007
Glossina austeni GAUT012313
Glossina pallidipes GPAI002110
Glossina morsitans GMOY009808
Glossina brevipalpis GBRI029502
Lucilia cuprina FF38 11152
Musca domestica 17012322
Bactrocera dorsalis 11784318
Bactrocera tryoni JHQJ01000025
Bactrocera cucurbitae 11870608
Ceratitis capitata CCAP010622
Megaselia scalaris MESCA012182
Anopheles gambiae AGAP002086
Anopheles arabiensis AARA006858
Anopheles coluzzii ACOM042254
Anopheles merus AMEM016576
Anopheles melas AMEC005810
Anopheles quadriannulatus AQUA008915
Anopheles christyi ACHR008803
Anopheles epiroticus AEPI005183
Anopheles culicifacies ACUA019285
Anopheles minimus AMIN000653
Anopheles funestus AFUN010856
Anopheles maculatus AMAM005880
Anopheles stephensi ASTE004250
Anopheles dirus ADIR006457
Anopheles farauti AFAF006144
Anopheles atroparvus AATE006379
Anopheles sinensis ASIC021478
Anopheles albimanus AALB009291
Anopheles darlingi ADAC009806
Aedes aegypti AAEL000448
Culex quinquefasciatus CPIJ002852
Polypedilum nubifer Pn.14441
Polypedilum vanderplanki Pv.10897
Belgica antarctica IU25 01494
Lutzomyia longipalpis LLOTMP006889
Mayetiola destructor Mdes012467
Anoplophora glabripennis AGLA013520
Leptinotarsa decemlineata LDEC011947
Dendroctonus ponderosae YQE 09067
Rhyzobius pseudopulcher
Thanasimus formicarius
Meloe violaceus
Bitoma cylindrica
Tribolium castaneum TC004141
Xylobiops basilaris
Ocypus brunnipes
Lamprohiza splendidula
Cicindela hybrida
Noterus clavicornis
Cybister lateralimarginalis
Aspidytes wrasei
Lepicerus sp
Micromalthus debilis
Mengenilla moldrzyki mesp 5503
Hydroscapha redfordi
Cyphon laevipennis
Gerris buenoi GBUE008298
Apis florea 4234158772
Apis mellifera GB43565
Apis dorsata 51575449
Apis cerana ACSNU07187T0
Bombus impatiens Bimp 2.0
Bombus terrestris 15209022
Melipona quadrifasciata MQUAD 0000a3
Eufriesea mexicana EMEXI 0013fb
Megachile rotundata MROTU 002605
Habropoda laboriosa HLABO 000080
Lasioglossum albipes Lalb 06355
Dufourea novaeangliae DNOVA 00119c
Wasmannia auropunctata 23054433
Monomorium pharaonis 4779423924
Vollenhovia emeryi 19033892
Acromyrmex echinatior AECH16502
Atta cephalotes ACEP25351
Linepithema humile LH11502
Solenopsis invicta SINV10407
Pogonomyrmex barbatus PB13658
Camponotus floridanus CFLO16289
Cerapachys biroi EZA62554.1
Harpegnathos saltator HSAL13490
Polistes dominula 9549
Orussus abietinus OABI002126
Cephus cinctus CCINC 000ad9
Athalia rosae AROS005926
Nasonia vitripennis Nasvi2EG001094
Copidosoma floridanum CFLO018662
Trichogramma pretiosum TPRE000790
Oncopeltus fasciatus OFAS008841
Halyomorpha halys HHAL004967
Cimex lectularius CLEC007178
Homalodisca vitripennis HVIT019255
Acyrthosiphon pisum ACYPI000323
Frankliniella occidentalis FOCC001449
Zootermopsis nevadensis Znev 03079
Calopteryx splendens size95106
Ladona fulva LFUL017649
Ephemera danica EDAN009823
Diptera
Lepidoptera
Trichoptera
Coleoptera
Strepsiptera
Hymenoptera
Psocodea
Thysanoptera
Hemiptera
Blattodea

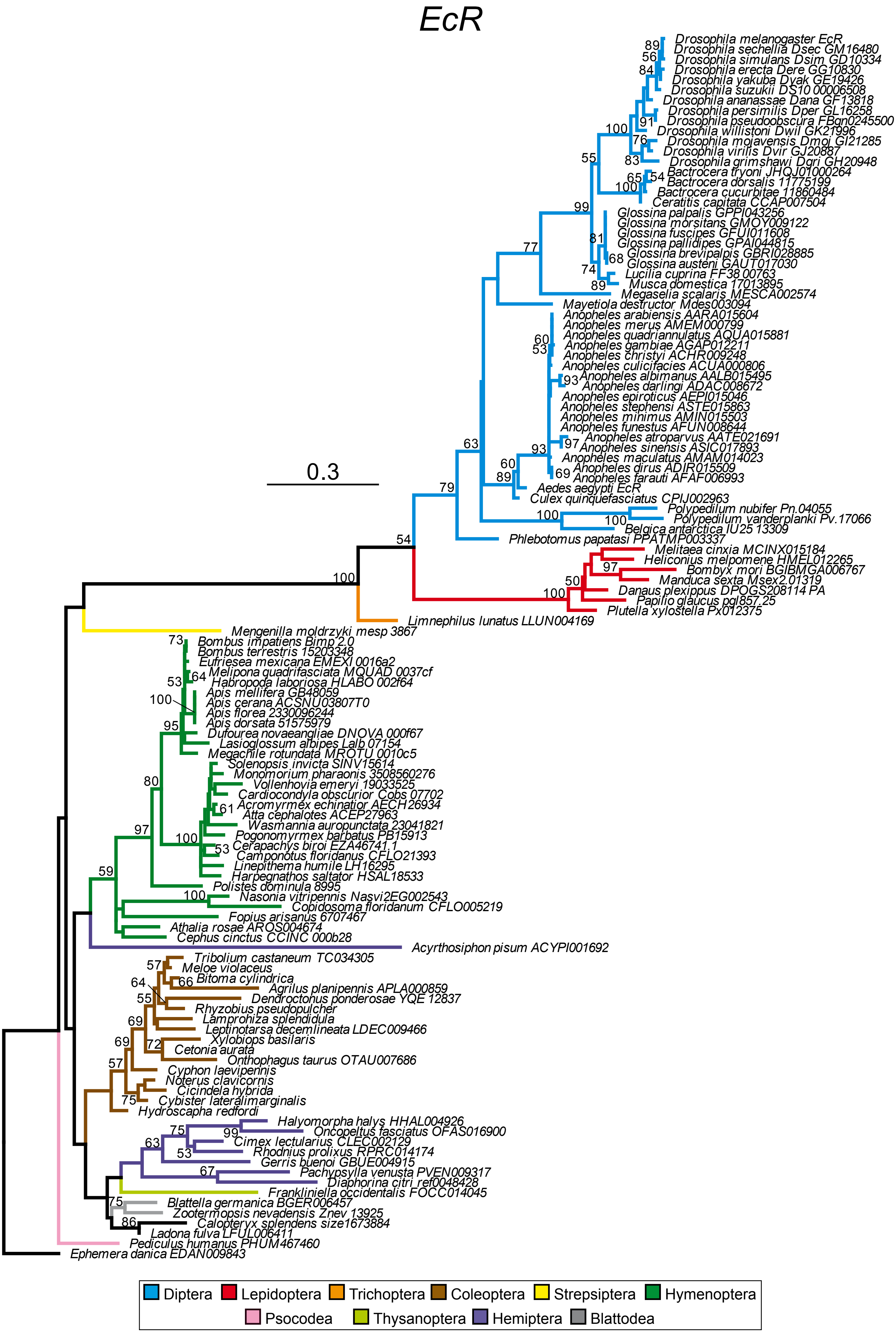
EcR
0.3
Drosophila melanogaster EcR
Drosophila sechellia Dsec GM16480
Drosophila simulans Dsim GD10334
Drosophila erecta Dere GG10830
Drosophila yakuba Dyak GE19426
Drosophila suzukii DS10 00006508
Drosophila ananassae Dana GF13818
Drosophila persimilis Dper GL16258
Drosophila pseudoobscura FBgn0245500
Drosophila willistoni Dwil GK21996
Drosophila mojavensis Dmoj GI21285
Drosophila virilis Dvir GJ20887
Drosophila grimshawi Dgri GH20948
Bactrocera tryoni JHQJ01000264
Bactrocera dorsalis 11775199
Bactrocera cucurbitae 11860484
Ceratitis capitata CCAP007504
Glossina palpalis GPPI043256
Glossina morsitans GMOY009122
Glossina fuscipes GFUI011608
Glossina pallidipes GPAI044815
Glossina brevipalpis GBRI028885
Glossina austeni GAUT017030
Lucilia cuprina FF38 00763
Musca domestica 17013895
Megaselia scalaris MESCA002574
Mayetiola destructor Mdes003094
Anopheles arabiensis AARA015604
Anopheles merus AMEM000799
Anopheles quadriannulatus AQUA015881
Anopheles gambiae AGAP012211
Anopheles christyi ACHR009248
Anopheles culicifacies ACUA000806
Anopheles albimanus AALB015495
Anopheles darlingi ADAC008672
Anopheles epiroticus AEPI015046
Anopheles stephensi ASTE015863
Anopheles minimus AMIN015503
Anopheles funestus AFUN008644
Anopheles atroparvus AATE021691
Anopheles sinensis ASIC017893
Anopheles maculatus AMAM014023
Anopheles dirus ADIR015509
Anopheles farauti AFAF006993
Aedes aegypti EcR
Culex quinquefasciatus CPIJ002963
Polypedilum nubifer Pn.04055
Polypedilum vanderplanki Pv.17066
Belgica antarctica IU25 13309
Phlebotomus papatasi PPATMP003337
Melitaea cinxia MCINX015184
Heliconius melpomene HMEL012265
Bombyx mori BGIBMGA006767
Manduca sexta Msex2.01319
Danaus plexippus DPOGS208114 PA
Papilio glaucus pgl857.25
Plutella xylostella Px012375
Limnephilus lunatus LLUN004169
Mengenilla moldrzyki mesp 3867
Bombus impatiens Bimp 2.0
Bombus terrestris 15203348
Eufriesea mexicana EMEXI 0016a2
Melipona quadrifasciata MQUAD 0037cf
Habropoda laboriosa HLABO 002f64
Apis mellifera GB48059
Apis cerana ACSNU03807T0
Apis florea 2330096244
Apis dorsata 51575979
Dufourea novaeangliae DNOVA 000f67
Lasioglossum albipes Lalb 07154
Megachile rotundata MROTU 0010c5
Solenopsis invicta SINV15614
Monomorium pharaonis 3508560276
Vollenhovia emeryi 19033525
Cardiocondyla obscurior Cobs 07702
Acromyrmex echinatior AECH26934
Atta cephalotes ACEP27963
Wasmannia auropunctata 23041821
Pogonomyrmex barbatus PB15913
Cerapachys biroi EZA46741.1
Camponotus floridanus CFLO21393
Linepithema humile LH16295
Harpegnathos saltator HSAL18533
Polistes dominula 8995
Nasonia vitripennis Nasvi2EG002543
Copidosoma floridanum CFLO005219
Fopius arisanus 6707467
Athalia rosae AROS004674
Cephus cinctus CCINC 000b28
Acyrthosiphon pisum ACYPI001692
Tribolium castaneum TC034305
Meloe violaceus
Bitoma cylindrica
Agrilus planipennis APLA000859
Dendroctonus ponderosae YQE 12837
Rhyzobius pseudopulcher
Lamprohiza splendidula
Leptinotarsa decemlineata LDEC009466
Xylobiops basilaris
Cetonia aurata
Onthophagus taurus OTAU007686
Cyphon laevipennis
Noterus clavicornis
Cicindela hybrida
Cybister lateralimarginalis
Hydroscapha redfordi
Halyomorpha halys HHAL004926
Oncopeltus fasciatus OFAS016900
Cimex lectularius CLEC002129
Rhodnius prolixus RPRC014174
Gerris buenoi GBUE004915
Pachypsylla venusta PVEN009317
Diaphorina citri ref0048428
Frankliniella occidentalis FOCC014045
Blattella germanica BGER006457
Zootermopsis nevadensis Znev 13925
Calopteryx splendens size1673884
Ladona fulva LFUL006411
Pediculus humanus PHUM467460
Ephemera danica EDAN009843
Diptera
Lepidoptera
Trichoptera
Coleoptera
Strepsiptera
Hymenoptera
Psocodea
Thysanoptera
Hemiptera
Blattodea

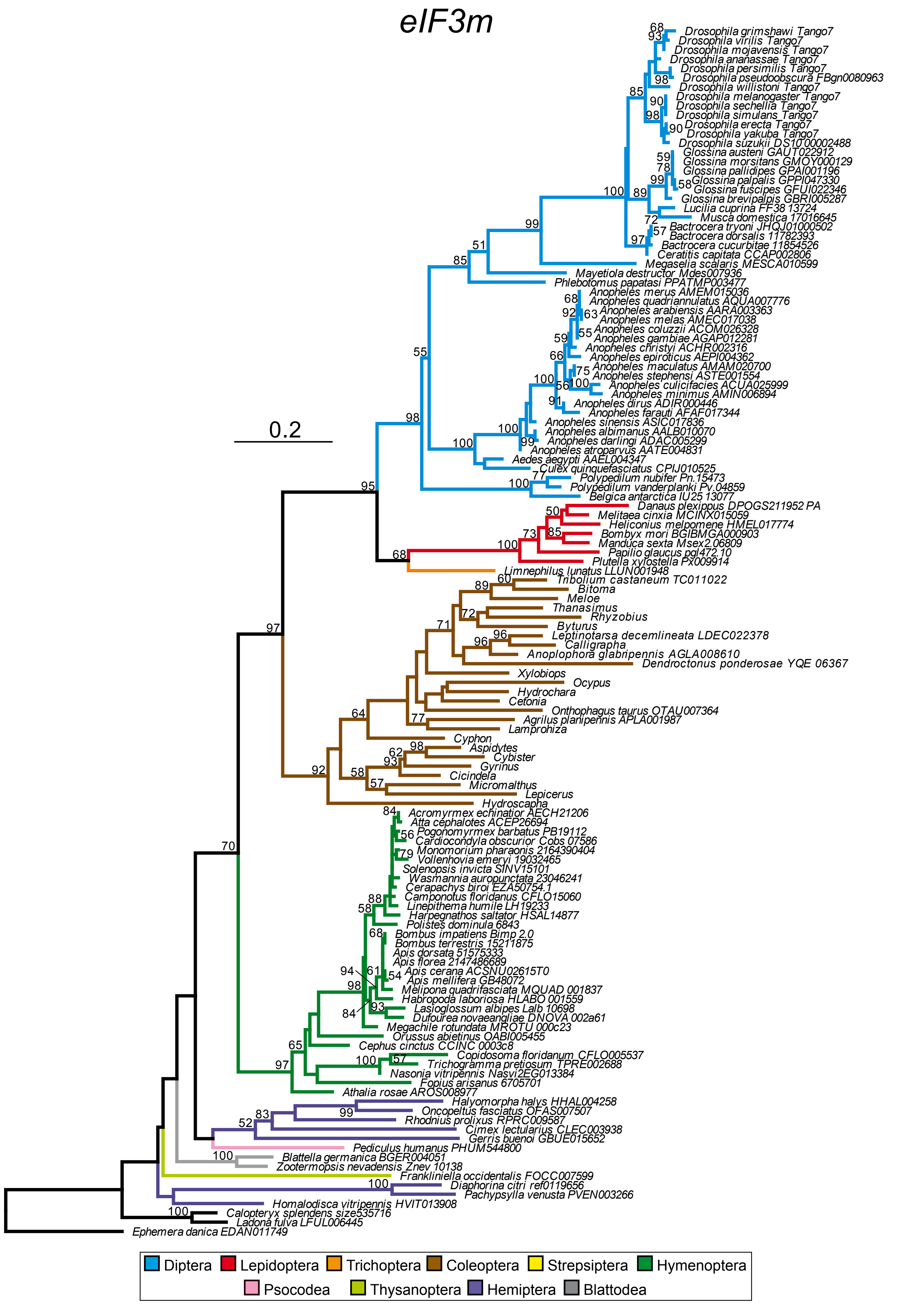
eIF3m
0.2
Drosophila grimshawi Tango7
Drosophila virilis Tango7
Drosophila mojavensis Tango7
Drosophila ananassae Tango7
Drosophila persimilis Tango7
Drosophila pseudoobscura FBgn0080963
Drosophila willistoni Tango7
Drosophila melanogaster Tango7
Drosophila sechellia Tango7
Drosophila simulans Tango7
Drosophila erecta Tango7
Drosophila yakuba Tango7
Drosophila suzukii DS10 00002488
Glossina austeni GAUT022912
Glossina morsitans GMOY000129
Glossina pallidipes GPAI001196
Glossina palpalis GPPI047330
Glossina fuscipes GFUI022346
Glossina brevipalpis GBRI005287
Lucilia cuprina FF38 13724
Musca domestica 17016645
Bactrocera tryoni JHQJ01000502
Bactrocera dorsalis 11782393
Bactrocera cucurbitae 11854526
Ceratitis capitata CCAP002806
Megaselia scalaris MESCA010599
Mayetiola destructor Mdes007936
Phlebotomus papatasi PPATMP003477
Anopheles merus AMEM015036
Anopheles quadriannulatus AQUA007776
Anopheles arabiensis AARA003363
Anopheles melas AMEC017038
Anopheles coluzzii ACOM026328
Anopheles gambiae AGAP012281
Anopheles christyi ACHR002316
Anopheles epiroticus AEPI004362
Anopheles maculatus AMAM020700
Anopheles stephensi ASTE001554
Anopheles culicifacies ACUA025999
Anopheles minimus AMIN006894
Anopheles dirus ADIR000446
Anopheles farauti AFAF017344
Anopheles sinensis ASIC017836
Anopheles albimanus AALB010070
Anopheles darlingi ADAC005299
Anopheles atroparvus AATE004831
Aedes aegypti AAEL004347
Culex quinquefasciatus CPIJ010525
Polypedilum nubifer Pn.15473
Polypedilum vanderplanki Pv.04859
Belgica antarctica IU25 13077
Danaus plexippus DPOGS211952 PA
Melitaea cinxia MCINX015059
Heliconius melpomene HMEL017774
Bombyx mori BGIBMGA000903
Manduca sexta Msex2.06809
Papilio glaucus pgl472.10
Plutella xylostella Px009914
Limnephilus lunatus LLUN001948
Tribolium castaneum TC011022
Bitoma
Meloe
Thanasimus
Rhyzobius
Byturus
Leptinotarsa decemlineata LDEC022378
Calligrapha
Anoplophora glabripennis AGLA008610
Dendroctonus ponderosae YQE 06367
Xylobiops
Ocypus
Hydrochara
Cetonia
Onthophagus taurus OTAU007364
Agrilus planipennis APLA001987
Lamprohiza
Cyphon
Aspidytes
Cybister
Gyrinus
Cicindela
Micromalthus
Lepicerus
Hydroscapha
Acromyrmex echinatior AECH21206
Atta cephalotes ACEP26694
Pogonomyrmex barbatus PB19112
Cardiocondyla obscurior Cobs 07586
Monomorium pharaonis 2164390404
Vollenhovia emeryi 19032465
Solenopsis invicta SINV15101
Wasmannia auropunctata 23046241
Cerapachys biroi EZA50754.1
Camponotus floridanus CFLO15060
Linepithema humile LH19233
Harpegnathos saltator HSAL14877
Polistes dominula 6843
Bombus impatiens Bimp 2.0
Bombus terrestris 15211875
Apis dorsata 51575333
Apis florea 2147486689
Apis cerana ACSNU02615T0
Apis mellifera GB48072
Melipona quadrifasciata MQUAD 001837
Habropoda laboriosa HLABO 001559
Lasioglossum albipes Lalb 10698
Dufourea novaeangliae DNOVA 002a61
Megachile rotundata MROTU 000c23
Orussus abietinus OABI005455
Cephus cinctus CCINC 0003c8
Copidosoma floridanum CFLO005537
Trichogramma pretiosum TPRE002688
Nasonia vitripennis Nasvi2EG013384
Fopius arisanus 6705701
Athalia rosae AROS008977
Halyomorpha halys HHAL004258
Oncopeltus fasciatus OFAS007507
Rhodnius prolixus RPRC009587
Cimex lectularius CLEC003938
Gerris buenoi GBUE015652
Pediculus humanus PHUM544800
Blattella germanica BGER004051
Zootermopsis nevadensis Znev 10138
Frankliniella occidentalis FOCC007599
Diaphorina citri ref0119656
Pachypsylla venusta PVEN003266
Homalodisca vitripennis HVIT013908
Calopteryx splendens size535716
Ladona fulva LFUL006445
Ephemera danica EDAN011749
Diptera
Lepidoptera
Trichoptera
Coleoptera
Strepsiptera
Hymenoptera
Psocodea
Thysanoptera
Hemiptera
Blattodea

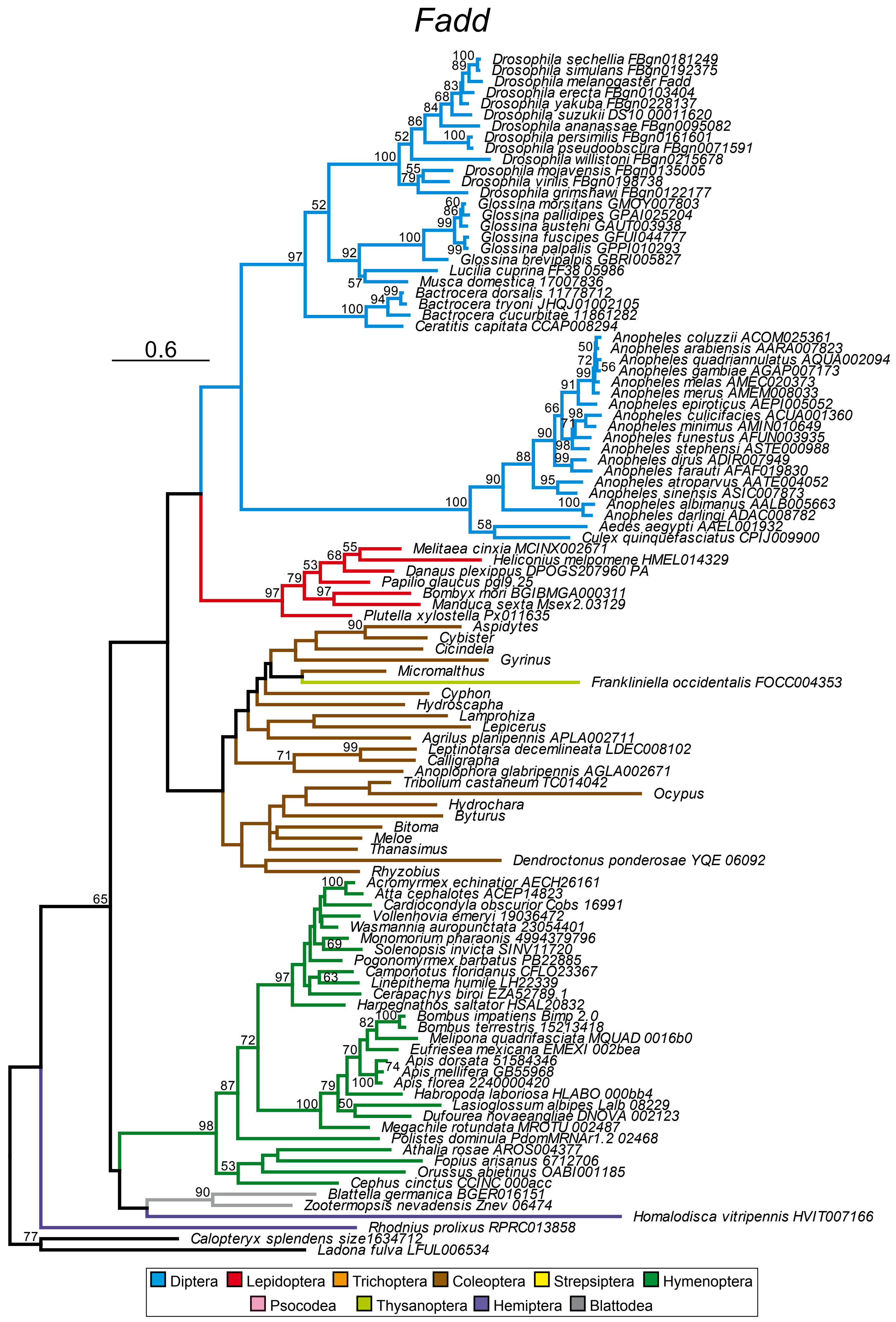
Fadd
0.6
Drosophila sechellia FBgn0181249
Drosophila simulans FBgn0192375
Drosophila melanogaster Fadd
Drosophila erecta FBgn0103404
Drosophila yakuba FBgn0228137
Drosophila suzukii DS10 00011620
Drosophila ananassae FBgn0095082
Drosophila persimilis FBgn0161601
Drosophila pseudoobscura FBgn0071591
Drosophila willistoni FBgn0215678
Drosophila mojavensis FBgn0135005
Drosophila virilis FBgn0198738
Drosophila grimshawi FBgn0122177
Glossina morsitans GMOY007803
Glossina pallidipes GPAI025204
Glossina austeni GAUT003938
Glossina fuscipes GFUI044777
Glossina palpalis GPPI010293
Glossina brevipalpis GBRI005827
Lucilia cuprina FF38 05986
Musca domestica 17007836
Bactrocera dorsalis 11778712
Bactrocera tryoni JHQJ01002105
Bactrocera cucurbitae 11861282
Ceratitis capitata CCAP008294
Anopheles coluzzii ACOM025361
Anopheles arabiensis AARA007823
Anopheles quadriannulatus AQUA002094
Anopheles gambiae AGAP007173
Anopheles melas AMEC020373
Anopheles merus AMEM008033
Anopheles epiroticus AEPI005052
Anopheles culicifacies ACUA001360
Anopheles minimus AMIN010649
Anopheles funestus AFUN003935
Anopheles stephensi ASTE000988
Anopheles dirus ADIR007949
Anopheles farauti AFAF019830
Anopheles atroparvus AATE004052
Anopheles sinensis ASIC007873
Anopheles albimanus AALB005663
Anopheles darlingi ADAC008782
Aedes aegypti AAEL001932
Culex quinquefasciatus CPIJ009900
Melitaea cinxia MCINX002671
Heliconius melpomene HMEL014329
Danaus plexippus DPOGS207960 PA
Papilio glaucus pgl9.25
Bombyx mori BGIBMGA000311
Manduca sexta Msex2.03129
Plutella xylostella Px011635
Aspidytes
Cybister
Cicindela
Gyrinus
Micromalthus
Frankliniella occidentalis FOCC004353
Cyphon
Hydroscapha
Lamprohiza
Lepicerus
Agrilus planipennis APLA002711
Leptinotarsa decemlineata LDEC008102
Calligrapha
Anoplophora glabripennis AGLA002671
Tribolium castaneum TC014042
Ocypus
Hydrochara
Byturus
Bitoma
Meloe
Thanasimus
Dendroctonus ponderosae YQE 06092
Rhyzobius
Acromyrmex echinatior AECH26161
Atta cephalotes ACEP14823
Cardiocondyla obscurior Cobs 16991
Vollenhovia emeryi 19036472
Wasmannia auropunctata 23054401
Monomorium pharaonis 4994379796
Solenopsis invicta SINV11720
Pogonomyrmex barbatus PB22885
Camponotus floridanus CFLO23367
Linepithema humile LH22339
Cerapachys biroi EZA52789.1
Harpegnathos saltator HSAL20832
Bombus impatiens Bimp 2.0
Bombus terrestris 15213418
Melipona quadrifasciata MQUAD 0016b0
Eufriesea mexicana EMEXI 002bea
Apis dorsata 51584346
Apis mellifera GB55968
Apis florea 2240000420
Habropoda laboriosa HLABO 000bb4
Lasioglossum albipes Lalb 08229
Dufourea novaeangliae DNOVA 002123
Megachile rotundata MROTU 002487
Polistes dominula PdomMRNAr1.2 02468
Athalia rosae AROS004377
Fopius arisanus 6712706
Orussus abietinus OABI001185
Cephus cinctus CCINC 000acc
Blattella germanica BGER016151
Zootermopsis nevadensis Znev 06474
Homalodisca vitripennis HVIT007166
Rhodnius prolixus RPRC013858
Calopteryx splendens size1634712
Ladona fulva LFUL006534
Diptera
Lepidoptera
Trichoptera
Coleoptera
Strepsiptera
Hymenoptera
Psocodea
Thysanoptera
Hemiptera
Blattodea

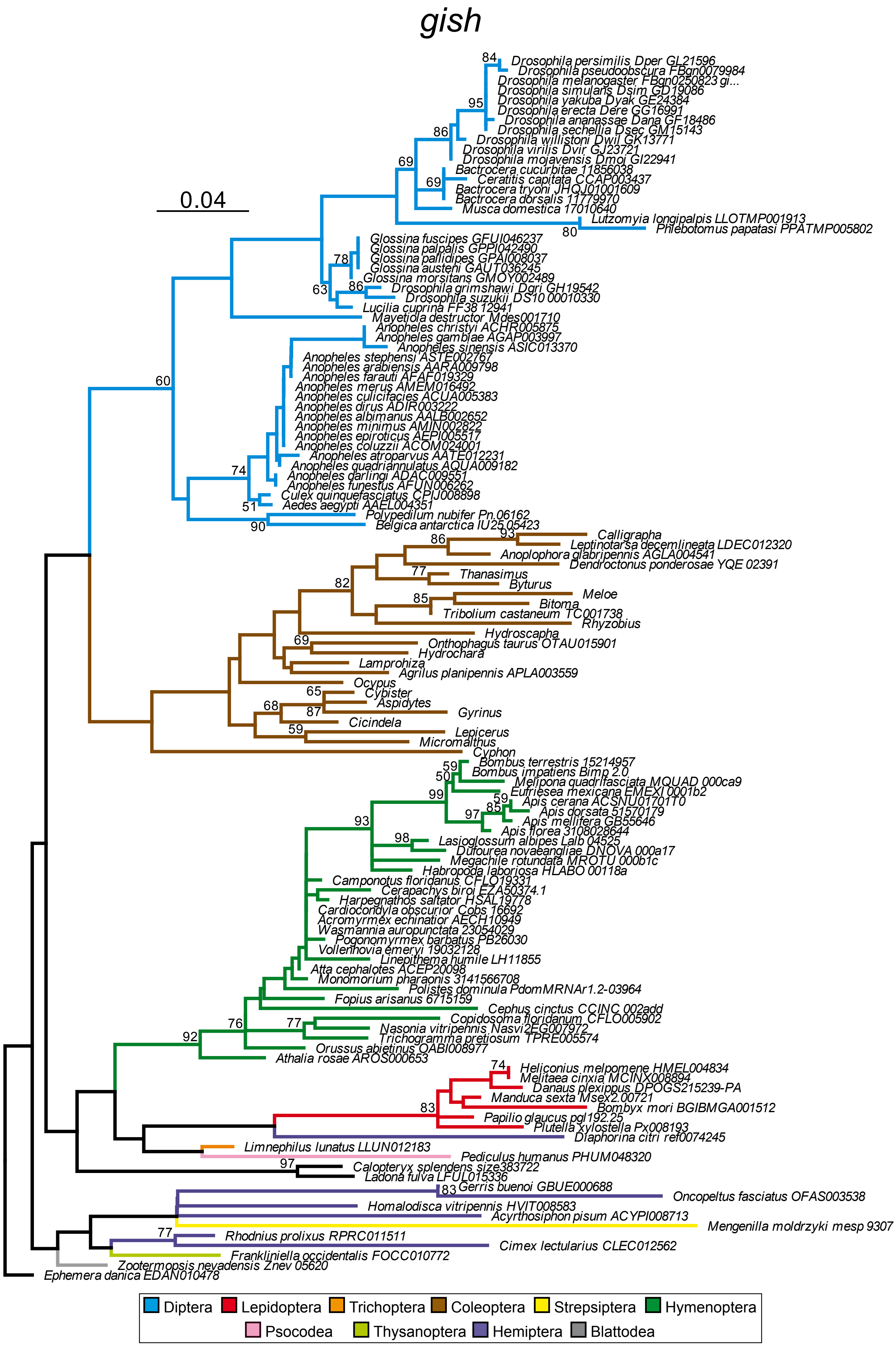

gish
0.04
Drosophila persimilis Dper GL21596
Drosophila pseudoobscura FBgn0079984
Drosophila melanogaster FBgn0250823 gi...
Drosophila simulans Dsim GD19086
Drosophila yakuba Dyak GE24384
Drosophila erecta Dere GG16991
Drosophila ananassae Dana GF18486
Drosophila sechellia Dsec GM15143
Drosophila willistoni Dwil GK13771
Drosophila virilis Dvir GJ23721
Drosophila mojavensis Dmoj GI22941
Bactrocera cucurbitae 11856038
Ceratitis capitata CCAP003437
Bactrocera tryoni JHQJ01001609
Bactrocera dorsalis 11779970
Musca domestica 17010640
Lutzomyia longipalpis LLOTMP001913
Phlebotomus papatasi PPATMP005802
Glossina fuscipes GFUI046237
Glossina palpalis GPPI042490
Glossina pallidipes GPAI008037
Glossina austeni GAUT036245
Glossina morsitans GMOY002489
Drosophila grimshawi Dgri GH19542
Drosophila suzukii DS10 00010330
Lucilia cuprina FF38 12941
Mayetiola destructor Mdes001710
Anopheles christyi ACHR005875
Anopheles gambiae AGAP003997
Anopheles sinensis ASIC013370
Anopheles stephensi ASTE002767
Anopheles arabiensis AARA009798
Anopheles farauti AFAF019329
Anopheles merus AMEM016492
Anopheles culicifacies ACUA005383
Anopheles dirus ADIR003222
Anopheles albimanus AALB002652
Anopheles minimus AMIN002822
Anopheles epiroticus AEPI005517
Anopheles coluzzii ACOM024001
Anopheles atroparvus AATE012231
Anopheles quadriannulatus AQUA009182
Anopheles darlingi ADAC009551
Anopheles funestus AFUN006262
Culex quinquefasciatus CPIJ008898
Aedes aegypti AAEL004351
Polypedilum nubifer Pn.06162
Belgica antarctica IU25 05423
Calligrapha
Leptinotarsa decemlineata LDEC012320
Anoplophora glabripennis AGLA004541
Dendroctonus ponderosae YQE 02391
Thanasimus
Byturus
Meloe
Bitoma
Tribolium castaneum TC001738
Rhyzobius
Hydroscapha
Onthophagus taurus OTAU015901
Hydrochara
Lamprohiza
Agrilus planipennis APLA003559
Ocypus
Cybister
Aspidytes
Gyrinus
Cicindela
Lepicerus
Micromalthus
Cyphon
Bombus terrestris 15214957
Bombus impatiens Bimp 2.0
Melipona quadrifasciata MQUAD 000ca9
Eufriesea mexicana EMEXI 0001b2
Apis cerana ACSNU01701T0
Apis dorsata 51570179
Apis mellifera GB55646
Apis florea 3108028644
Lasioglossum albipes Lalb 04525
Dufourea novaeangliae DNOVA 000a17
Megachile rotundata MROTU 000b1c
Habropoda laboriosa HLABO 00118a
Camponotus floridanus CFLO19331
Cerapachys biroi EZA50374.1
Harpegnathos saltator HSAL19778
Cardiocondyla obscurior Cobs 16692
Acromyrmex echinatior AECH10949
Wasmannia auropunctata 23054029
Pogonomyrmex barbatus PB26030
Vollenhovia emeryi 19032128
Linepithema humile LH11855
Atta cephalotes ACEP20098
Monomorium pharaonis 3141566708
Polistes dominula PdomMRNAr1.2-03964
Fopius arisanus 6715159
Cephus cinctus CCINC 002add
Copidosoma floridanum CFLO005902
Nasonia vitripennis Nasvi2EG007972
Trichogramma pretiosum TPRE005574
Orussus abietinus OABI008977
Athalia rosae AROS000653
Heliconius melpomene HMEL004834
Melitaea cinxia MCINX008894
Danaus plexippus DPOGS215239-PA
Manduca sexta Msex2.00721
Bombyx mori BGIBMGA001512
Papilio glaucus pgl192.25
Plutella xylostella Px008193
Diaphorina citri ref0074245
Limnephilus lunatus LLUN012183
Pediculus humanus PHUM048320
Calopteryx splendens size383722
Ladona fulva LFUL015336
Gerris buenoi GBUE000688
Oncopeltus fasciatus OFAS003538
Homalodisca vitripennis HVIT008583
Acyrthosiphon pisum ACYPI008713
Mengenilla moldrzyki mesp 9307
Rhodnius prolixus RPRC011511
Cimex lectularius CLEC012562
Frankliniella occidentalis FOCC010772
Zootermopsis nevadensis Znev 05620
Ephemera danica EDAN010478
84
95
86
69
69
80
78
63
86
60
74
51
90
93
86
77
82
85
69
65
68
87
59
59
50
99
59
85
97
93
98
76
77
92
74
83
97
83
77
Diptera
Lepidoptera
Trichoptera
Coleoptera
Strepsiptera
Hymenoptera
Psocodea
Thysanoptera
Hemiptera
Blattodea

gudu

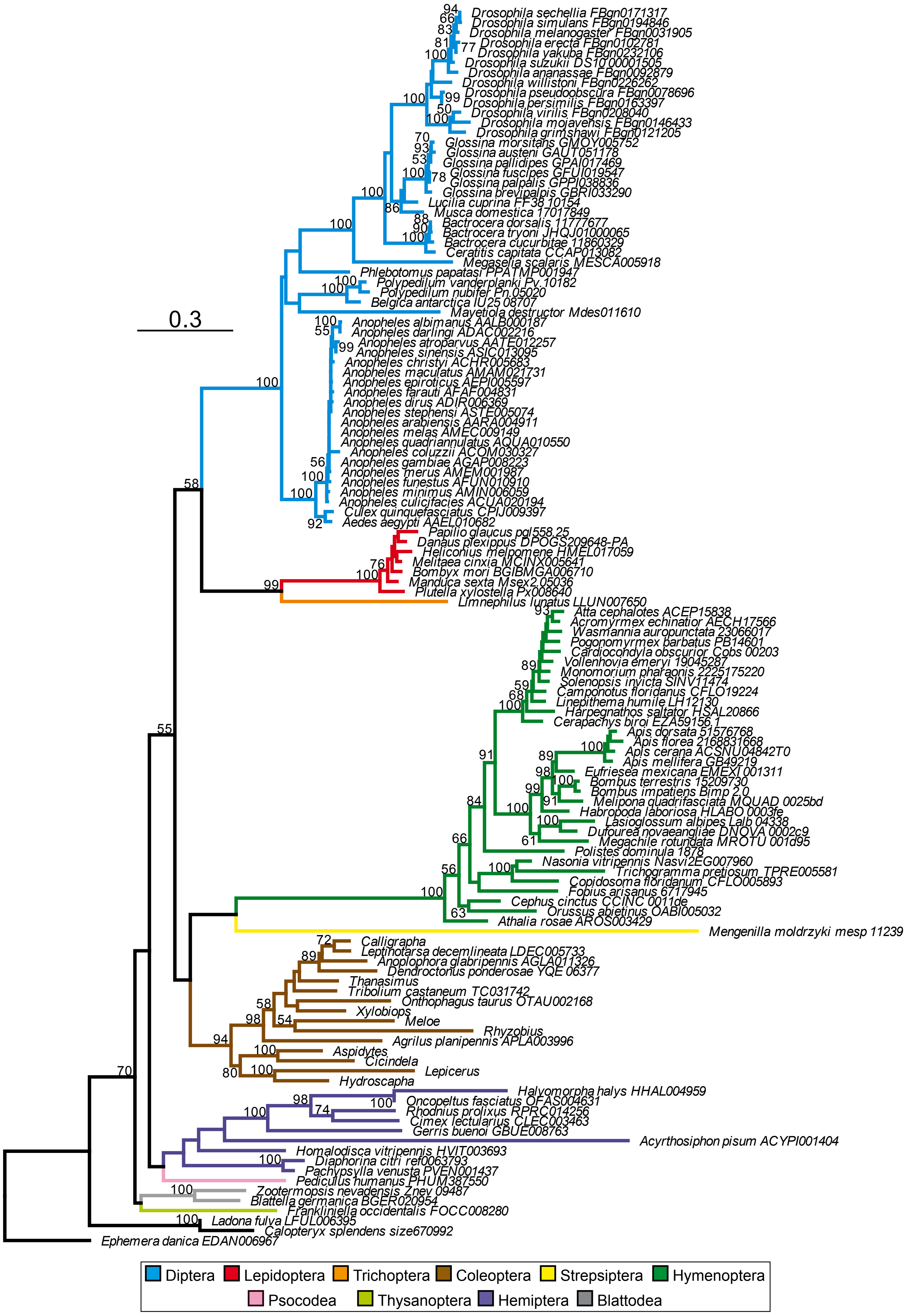
0.3
Drosophila sechellia FBgn0171317
Drosophila simulans FBgn0194846
Drosophila melanogaster FBgn0031905
Drosophila erecta FBgn0102781
Drosophila yakuba FBgn0232106
Drosophila suzukii DS10 00001505
Drosophila ananassae FBgn0092879
Drosophila willistoni FBgn0226262
Drosophila pseudoobscura FBgn0078696
Drosophila persimilis FBgn0163397
Drosophila virilis FBgn0208040
Drosophila mojavensis FBgn0146433
Drosophila grimshawi FBgn0121205
Glossina morsitans GMOY005752
Glossina austeni GAUT051178
Glossina pallidipes GPAI017469
Glossina fuscipes GFUI019547
Glossina palpalis GPPI038836
Glossina brevipalpis GBRI033290
Lucilia cuprina FF38 10154
Musca domestica 17017849
Bactrocera dorsalis 11777677
Bactrocera tryoni JHQJ01000065
Bactrocera cucurbitae 11860329
Ceratitis capitata CCAP013082
Megaselia scalaris MESCA005918
Phlebotomus papatasi PPATMP001947
Polypedilum vanderplanki Pv.10182
Polypedilum nubifer Pn.05020
Belgica antarctica IU25 08707
Mayetiola destructor Mdes011610
Anopheles albimanus AALB000187
Anopheles darlingi ADAC002216
Anopheles atroparvus AATE012257
Anopheles sinensis ASIC013095
Anopheles christyi ACHR005683
Anopheles maculatus AMAM021731
Anopheles epiroticus AEPI005597
Anopheles farauti AFAF004831
Anopheles dirus ADIR006369
Anopheles stephensi ASTE005074
Anopheles arabiensis AARA004911
Anopheles melas AMEC009149
Anopheles quadriannulatus AQUA010550
Anopheles coluzzii ACOM030327
Anopheles gambiae AGAP008223
Anopheles merus AMEM001987
Anopheles funestus AFUN010910
Anopheles minimus AMIN006059
Anopheles culicifacies ACUA020194
Culex quinquefasciatus CPIJ009397
Aedes aegypti AAEL010682
Papilio glaucus pgl558.25
Danaus plexippus DPOGS209648-PA
Heliconius melpomene HMEL017059
Melitaea cinxia MCINX005641
Bombyx mori BGIBMGA006710
Manduca sexta Msex2.05036
Plutella xylostella Px008640
Limnephilus lunatus LLUN007650
Atta cephalotes ACEP15838
Acromyrmex echinatior AECH17566
Wasmannia auropunctata 23066017
Pogonomyrmex barbatus PB14601
Cardiocondyla obscurior Cobs 00203
Vollenhovia emeryi 19045287
Monomorium pharaonis 2225175220
Solenopsis invicta SINV11474
Camponotus floridanus CFLO19224
Linepithema humile LH12130
Harpegnathos saltator HSAL20866
Cerapachys biroi EZA59156.1
Apis dorsata 51576768
Apis florea 2168831668
Apis cerana ACSNU04842T0
Apis mellifera GB49219
Eufriesea mexicana EMEXI 001311
Bombus terrestris 15209730
Bombus impatiens Bimp 2.0
Melipona quadrifasciata MQUAD 0025bd
Habropoda laboriosa HLABO 0003fe
Lasioglossum albipes Lalb 04338
Dufourea novaeangliae DNOVA 0002c9
Megachile rotundata MROTU 001d95
Polistes dominula 1878
Nasonia vitripennis Nasvi2EG007960
Trichogramma pretiosum TPRE005581
Copidosoma floridanum CFLO005893
Fopius arisanus 6717945
Cephus cinctus CCINC 0011de
Orussus abietinus OABI005032
Athalia rosae AROS003429
Mengenilla moldrzyki mesp 11239
Calligrapha
Leptinotarsa decemlineata LDEC005733
Anoplophora glabripennis AGLA011326
Dendroctonus ponderosae YQE 06377
Thanasimus
Tribolium castaneum TC031742
Onthophagus taurus OTAU002168
Xylobiops
Meloe
Rhyzobius
Agrilus planipennis APLA003996
Aspidytes
Cicindela
Lepicerus
Hydroscapha
Halyomorpha halys HHAL004959
Oncopeltus fasciatus OFAS004631
Rhodnius prolixus RPRC014256
Cimex lectularius CLEC003463
Gerris buenoi GBUE008763
Acyrthosiphon pisum ACYPI001404
Homalodisca vitripennis HVIT003693
Diaphorina citri ref0063793
Pachypsylla venusta PVEN001437
Pediculus humanus PHUM387550
Zootermopsis nevadensis Znev 09487
Blattella germanica BGER020954
Frankliniella occidentalis FOCC008280
Ladona fulva LFUL006395
Calopteryx splendens size670992
Ephemera danica EDAN006967
Diptera
Lepidoptera
Trichoptera
Coleoptera
Strepsiptera
Hymenoptera
Psocodea
Thysanoptera
Hemiptera
Blattodea

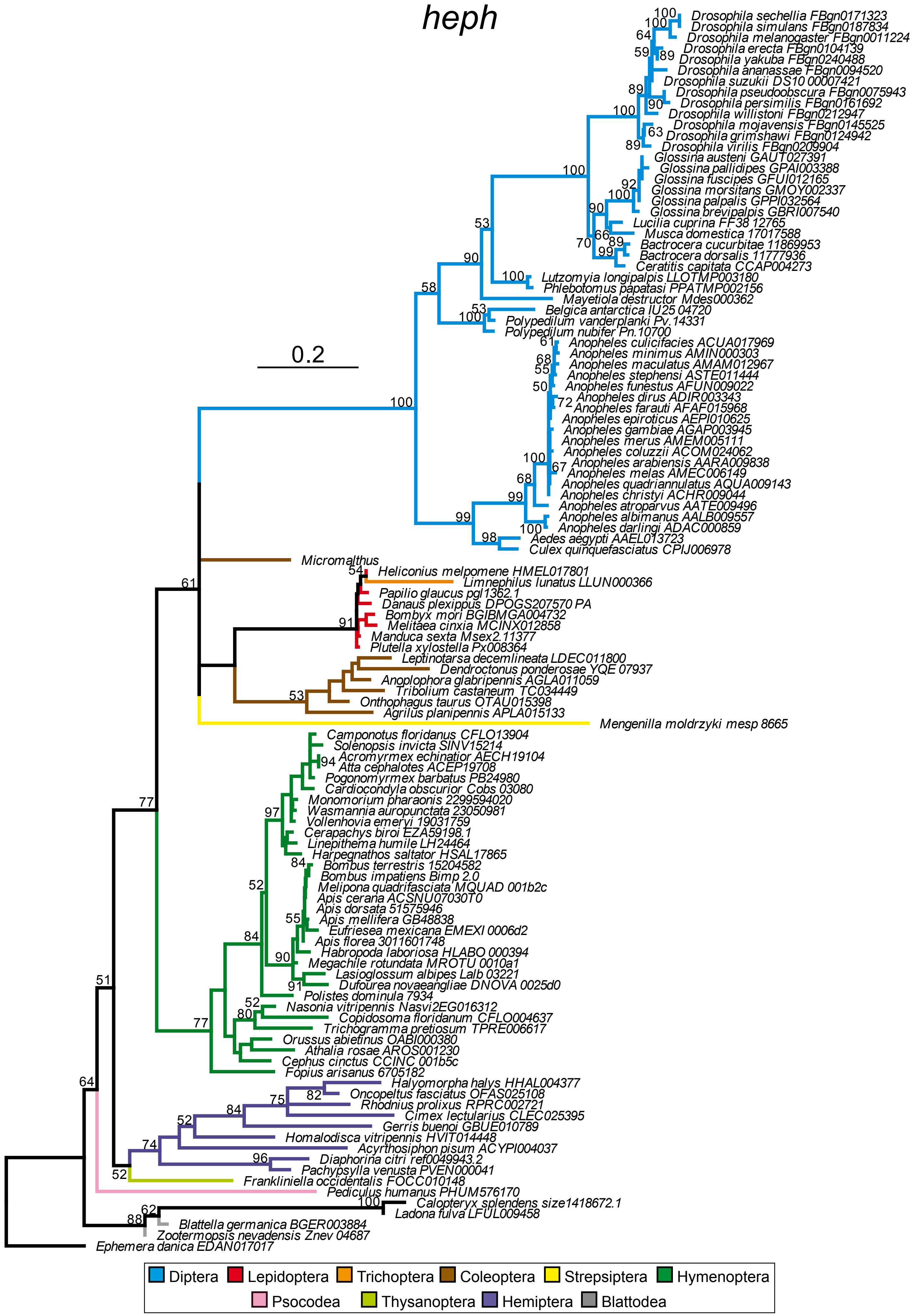

heph
0.2
Drosophila sechellia FBgn0171323
Drosophila simulans FBgn0187834
Drosophila melanogaster FBgn0011224
Drosophila erecta FBgn0104139
Drosophila yakuba FBgn0240488
Drosophila ananassae FBgn0094520
Drosophila suzukii DS10 00007421
Drosophila pseudoobscura FBgn0075943
Drosophila persimilis FBgn0161692
Drosophila willistoni FBgn0212947
Drosophila mojavensis FBgn0145525
Drosophila grimshawi FBgn0124942
Drosophila virilis FBgn0209904
Glossina austeni GAUT027391
Glossina pallidipes GPAI003388
Glossina fuscipes GFUI012165
Glossina morsitans GMOY002337
Glossina palpalis GPPI032564
Glossina brevipalpis GBRI007540
Lucilia cuprina FF38 12765
Musca domestica 17017588
Bactrocera cucurbitae 11869953
Bactrocera dorsalis 11777936
Ceratitis capitata CCAP004273
Lutzomyia longipalpis LLOTMP003180
Phlebotomus papatasi PPATMP002156
Mayetiola destructor Mdes000362
Belgica antarctica IU25 04720
Polypedilum vanderplanki Pv.14331
Polypedilum nubifer Pn.10700
Anopheles culicifacies ACUA017969
Anopheles minimus AMIN000303
Anopheles maculatus AMAM012967
Anopheles stephensi ASTE011444
Anopheles funestus AFUN009022
Anopheles dirus ADIR003343
Anopheles farauti AFAF015968
Anopheles epiroticus AEPI010625
Anopheles gambiae AGAP003945
Anopheles merus AMEM005111
Anopheles coluzzii ACOM024062
Anopheles arabiensis AARA009838
Anopheles melas AMEC006149
Anopheles quadriannulatus AQUA009143
Anopheles christyi ACHR009044
Anopheles atroparvus AATE009496
Anopheles albimanus AALB009557
Anopheles darlingi ADAC000859
Aedes aegypti AAEL013723
Culex quinquefasciatus CPIJ006978
Micromalthus
Heliconius melpomene HMEL017801
Limnephilus lunatus LLUN000366
Papilio glaucus pgl1362.1
Danaus plexippus DPOGS207570 PA
Bombyx mori BGIBMGA004732
Melitaea cinxia MCINX012858
Manduca sexta Msex2.11377
Plutella xylostella Px008364
Leptinotarsa decemlineata LDEC011800
Dendroctonus ponderosae YQE 07937
Anoplophora glabripennis AGLA011059
Tribolium castaneum TC034449
Onthophagus taurus OTAU015398
Agrilus planipennis APLA015133
Mengenilla moldrzyki mesp 8665
Camponotus floridanus CFLO13904
Solenopsis invicta SINV15214
Acromyrmex echinatior AECH19104
Atta cephalotes ACEP19708
Pogonomyrmex barbatus PB24980
Cardiocondyla obscurior Cobs 03080
Monomorium pharaonis 2299594020
Wasmannia auropunctata 23050981
Vollenhovia emeryi 19031759
Cerapachys biroi EZA59198.1
Linepithema humile LH24464
Harpegnathos saltator HSAL17865
Bombus terrestris 15204582
Bombus impatiens Bimp 2.0
Melipona quadrifasciata MQUAD 001b2c
Apis cerana ACSNU07030T0
Apis dorsata 51575946
Apis mellifera GB48838
Eufriesea mexicana EMEXI 0006d2
Apis florea 3011601748
Habropoda laboriosa HLABO 000394
Megachile rotundata MROTU 0010a1
Lasioglossum albipes Lalb 03221
Dufourea novaeangliae DNOVA 0025d0
Polistes dominula 7934
Nasonia vitripennis Nasvi2EG016312
Copidosoma floridanum CFLO004637
Trichogramma pretiosum TPRE006617
Orussus abietinus OABI000380
Athalia rosae AROS001230
Cephus cinctus CCINC 001b5c
Fopius arisanus 6705182
Halyomorpha halys HHAL004377
Oncopeltus fasciatus OFAS025108
Rhodnius prolixus RPRC002721
Cimex lectularius CLEC025395
Gerris buenoi GBUE010789
Homalodisca vitripennis HVIT014448
Acyrthosiphon pisum ACYPI004037
Diaphorina citri ref0049943.2
Pachypsylla venusta PVEN000041
Frankliniella occidentalis FOCC010148
Pediculus humanus PHUM576170
Calopteryx splendens size1418672.1
Ladona fulva LFUL009458
Blattella germanica BGER003884
Zootermopsis nevadensis Znev 04687
Ephemera danica EDAN017017
Diptera
Lepidoptera
Trichoptera
Coleoptera
Strepsiptera
Hymenoptera
Psocodea
Thysanoptera
Hemiptera
Blattodea

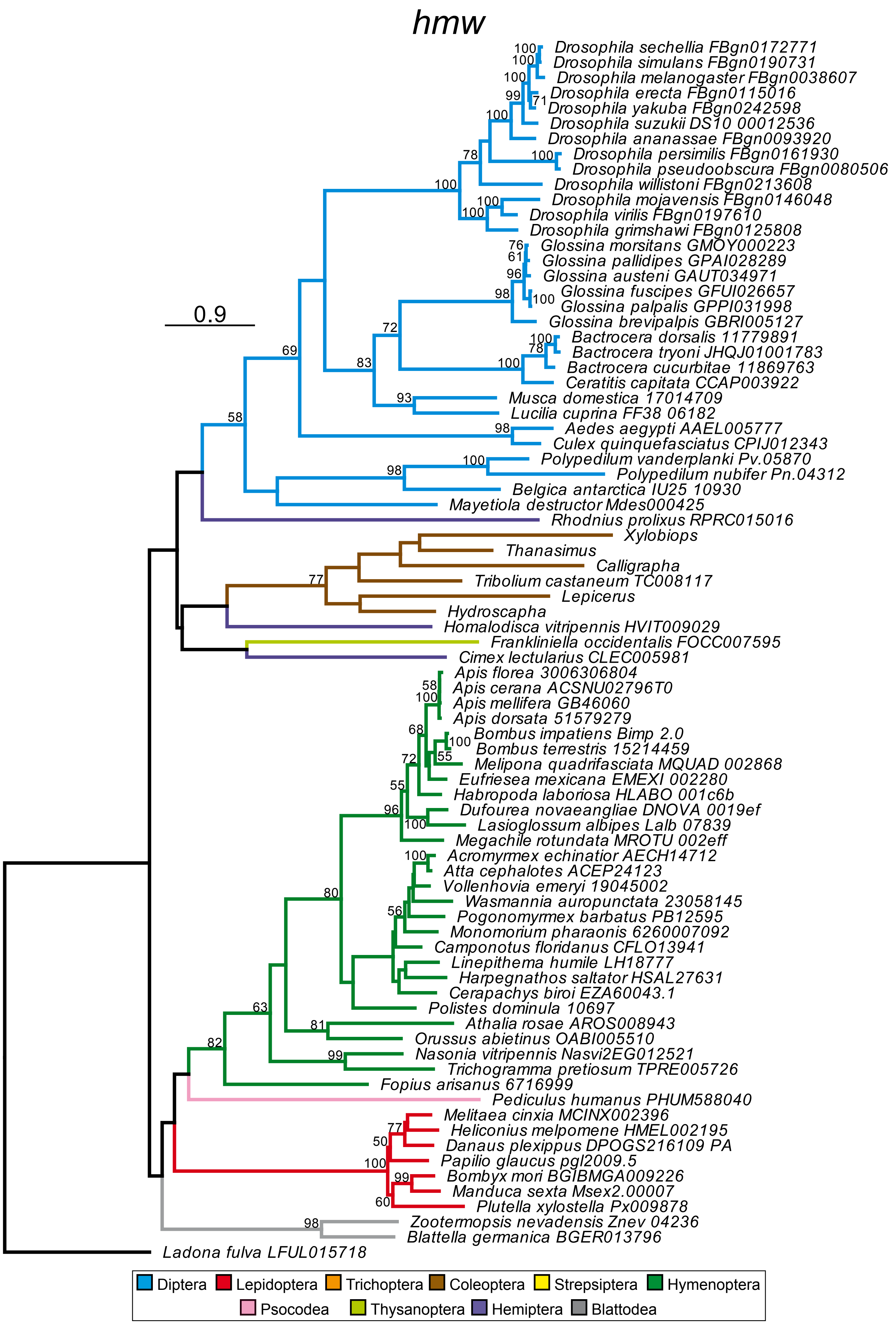

hmw
0.9
Drosophila sechellia FBgn0172771
Drosophila simulans FBgn0190731
Drosophila melanogaster FBgn0038607
Drosophila erecta FBgn0115016
Drosophila yakuba FBgn0242598
Drosophila suzukii DS10 00012536
Drosophila ananassae FBgn0093920
Drosophila persimilis FBgn0161930
Drosophila pseudoobscura FBgn0080506
Drosophila willistoni FBgn0213608
Drosophila mojavensis FBgn0146048
Drosophila virilis FBgn0197610
Drosophila grimshawi FBgn0125808
Glossina morsitans GMOY000223
Glossina pallidipes GPAI028289
Glossina austeni GAUT034971
Glossina fuscipes GFUI026657
Glossina palpalis GPPI031998
Glossina brevipalpis GBRI005127
Bactrocera dorsalis 11779891
Bactrocera tryoni JHQJ01001783
Bactrocera cucurbitae 11869763
Ceratitis capitata CCAP003922
Musca domestica 17014709
Lucilia cuprina FF38 06182
Aedes aegypti AAEL005777
Culex quinquefasciatus CPIJ012343
Polypedilum vanderplanki Pv.05870
Polypedilum nubifer Pn.04312
Belgica antarctica IU25 10930
Mayetiola destructor Mdes000425
Rhodnius prolixus RPRC015016
Xylobiops
Thanasimus
Calligrapha
Tribolium castaneum TC008117
Lepicerus
Hydroscapha
Homalodisca vitripennis HVIT009029
Frankliniella occidentalis FOCC007595
Cimex lectularius CLEC005981
Apis florea 3006306804
Apis cerana ACSNU02796T0
Apis mellifera GB46060
Apis dorsata 51579279
Bombus impatiens Bimp 2.0
Bombus terrestris 15214459
Melipona quadrifasciata MQUAD 002868
Eufriesea mexicana EMEXI 002280
Habropoda laboriosa HLABO 001c6b
Dufourea novaeangliae DNOVA 0019ef
Lasioglossum albipes Lalb 07839
Megachile rotundata MROTU 002eff
Acromyrmex echinatior AECH14712
Atta cephalotes ACEP24123
Vollenhovia emeryi 19045002
Wasmannia auropunctata 23058145
Pogonomyrmex barbatus PB12595
Monomorium pharaonis 6260007092
Camponotus floridanus CFLO13941
Linepithema humile LH18777
Harpegnathos saltator HSAL27631
Cerapachys biroi EZA60043.1
Polistes dominula 10697
Athalia rosae AROS008943
Orussus abietinus OABI005510
Nasonia vitripennis Nasvi2EG012521
Trichogramma pretiosum TPRE005726
Fopius arisanus 6716999
Pediculus humanus PHUM588040
Melitaea cinxia MCINX002396
Heliconius melpomene HMEL002195
Danaus plexippus DPOGS216109 PA
Papilio glaucus pgl2009.5
Bombyx mori BGIBMGA009226
Manduca sexta Msex2.00007
Plutella xylostella Px009878
Zootermopsis nevadensis Znev 04236
Blattella germanica BGER013796
Ladona fulva LFUL015718
Diptera
Lepidoptera
Trichoptera
Coleoptera
Strepsiptera
Hymenoptera
Psocodea
Thysanoptera
Hemiptera
Blattodea

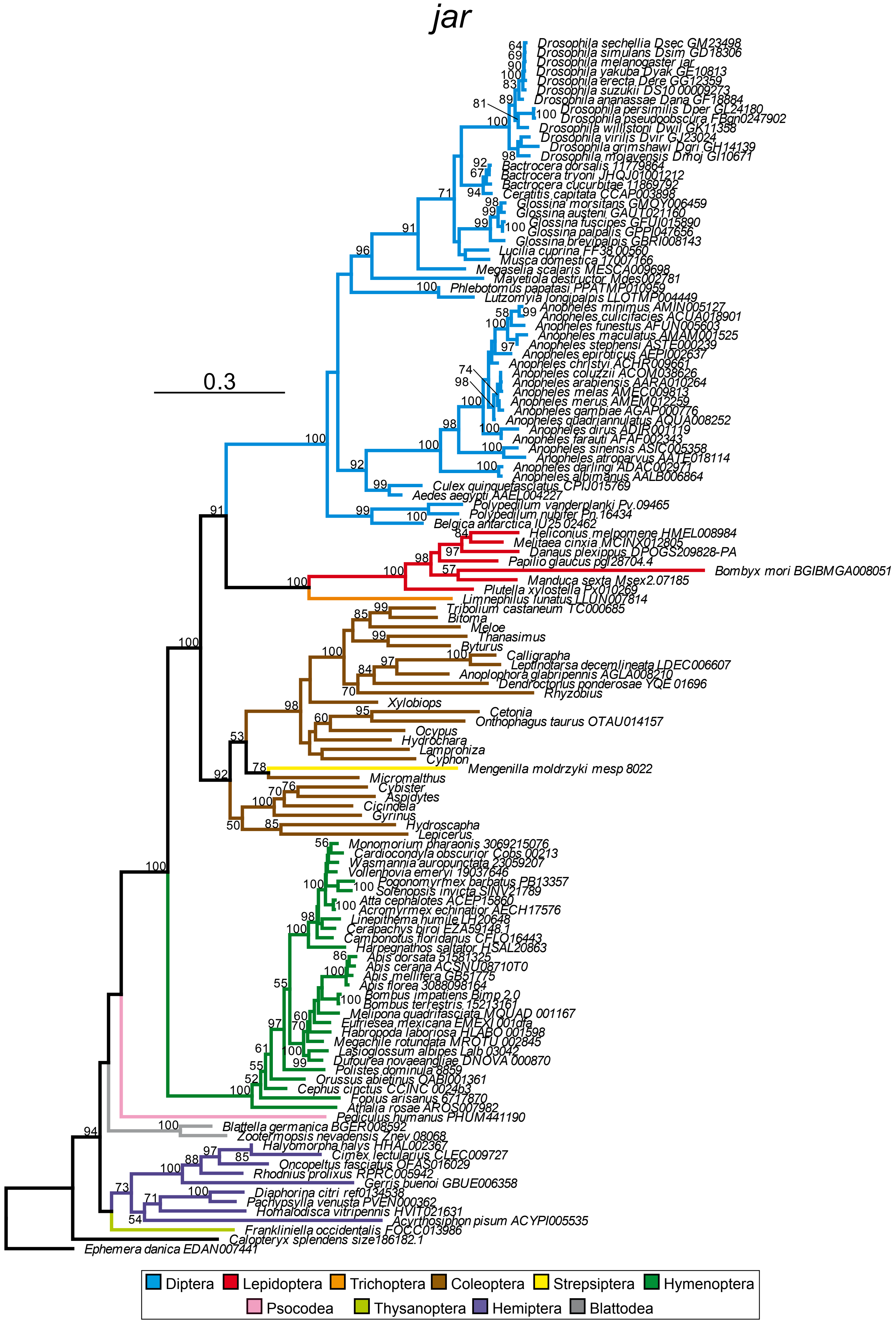
jar
0.3
Drosophila sechellia Dsec GM23498
Drosophila simulans Dsim GD18306
Drosophila melanogaster jar
Drosophila yakuba Dyak GE10813
Drosophila erecta Dere GG12359
Drosophila suzukii DS10 00009273
Drosophila ananassae Dana GF18884
Drosophila persimilis Dper GL24180
Drosophila pseudoobscura FBgn0247902
Drosophila willistoni Dwil GK11358
Drosophila virilis Dvir GJ23024
Drosophila grimshawi Dgri GH14139
Drosophila mojavensis Dmoj GI10671
Bactrocera dorsalis 11779864
Bactrocera tryoni JHQJ01001212
Bactrocera cucurbitae 11869792
Ceratitis capitata CCAP003898
Glossina morsitans GMOY006459
Glossina austeni GAUT021160
Glossina fuscipes GFUI015890
Glossina palpalis GPPI047656
Glossina brevipalpis GBRI008143
Lucilia cuprina FF38 00560
Musca domestica 17007166
Megaselia scalaris MESCA009698
Mayetiola destructor Mdes002781
Phlebotomus papatasi PPATMP010959
Lutzomyia longipalpis LLOTMP004449
Anopheles minimus AMIN005127
Anopheles culicifacies ACUA018901
Anopheles funestus AFUN005603
Anopheles maculatus AMAM001525
Anopheles stephensi ASTE000239
Anopheles epiroticus AEPI002637
Anopheles christyi ACHR009661
Anopheles coluzzii ACOM038626
Anopheles arabiensis AARA010264
Anopheles melas AMEC009813
Anopheles merus AMEM012259
Anopheles gambiae AGAP000776
Anopheles quadriannulatus AQUA008252
Anopheles dirus ADIR001119
Anopheles farauti AFAF002343
Anopheles sinensis ASIC005358
Anopheles atroparvus AATE018114
Anopheles darlingi ADAC002971
Anopheles albimanus AALB006864
Culex quinquefasciatus CPIJ015769
Aedes aegypti AAEL004227
Polypedilum vanderplanki Pv.09465
Polypedilum nubifer Pn.16434
Belgica antarctica IU25 02462
Heliconius melpomene HMEL008984
Melitaea cinxia MCINX012805
Danaus plexippus DPOGS209828-PA
Papilio glaucus pgl28704.4
Bombyx mori BGIBMGA008051
Manduca sexta Msex2.07185
Plutella xylostella Px010269
Limnephilus lunatus LLUN007814
Tribolium castaneum TC000685
Bitoma
Meloe
Thanasimus
Byturus
Calligrapha
Leptinotarsa decemlineata LDEC006607
Anoplophora glabripennis AGLA008210
Dendroctonus ponderosae YQE 01696
Rhyzobius
Xylobiops
Cetonia
Onthophagus taurus OTAU014157
Ocypus
Hydrochara
Lamprohiza
Cyphon
Mengenilla moldrzyki mesp 8022
Micromalthus
Cybister
Aspidytes
Cicindela
Gyrinus
Hydroscapha
Lepicerus
Monomorium pharaonis 3069215076
Cardiocondyla obscurior Cobs 00213
Wasmannia auropunctata 23059207
Vollenhovia emeryi 19037646
Pogonomyrmex barbatus PB13357
Solenopsis invicta SINV21789
Atta cephalotes ACEP15860
Acromyrmex echinatior AECH17576
Linepithema humile LH20648
Cerapachys biroi EZA59148.1
Camponotus floridanus CFLO16443
Harpegnathos saltator HSAL20863
Apis dorsata 51581325
Apis cerana ACSNU08710T0
Apis mellifera GB51775
Apis florea 3088098164
Bombus impatiens Bimp 2.0
Bombus terrestris 15213161
Melipona quadrifasciata MQUAD 001167
Eufriesea mexicana EMEX 001dfa
Habropoda laboriosa HLABO 001598
Megachile rotundata MROTU 002845
Lasioglossum albipes Lalb 03042
Dufourea novaeangliae DNOVA 000870
Polistes dominula 8859
Orussus abietinus OABI001361
Cephus cinctus CCINC 0024b3
Fopius arisanus 6717870
Athalia rosae AROS007982
Pediculus humanus PHUM441190
Blattella germanica BGER008592
Zootermopsis nevadensis Znev 08068
Halyomorpha halys HHAL002367
Cimex lectularius CLEC009727
Oncopeltus fasciatus OFAS016029
Rhodnius prolixus RPRC005942
Gerris buenoi GBUE006358
Diaphorina citri ref0134538
Pachypsylla venusta PVEN000362
Homalodisca vitripennis HVIT021631
Acyrthosiphon pisum ACYPI005535
Frankliniella occidentalis FOCC013986
Calopteryx splendens size186182.1
Ephemera danica EDAN007441
Diptera
Lepidoptera
Trichoptera
Coleoptera
Strepsiptera
Hymenoptera
Psocodea
Thysanoptera
Hemiptera
Blattodea

klhl10

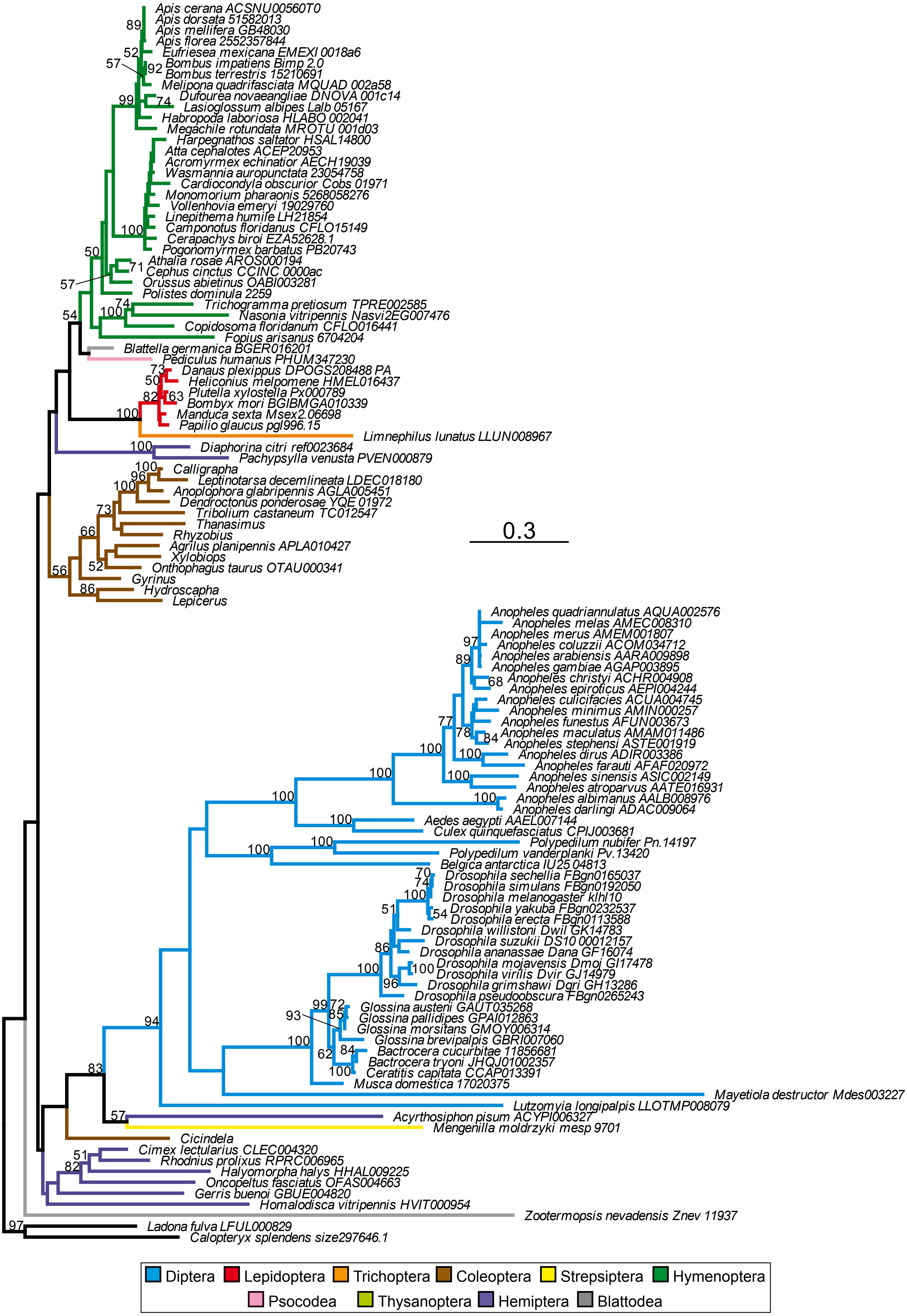
Apis cerana ACSNU00560T0
Apis dorsata 51582013
Apis mellifera GB48030
Apis florea 2552357844
Eufriesea mexicana EMEXI 0018a6
Bombus impatiens Bimp 2.0
Bombus terrestris 15210691
Melipona quadrifasciata MQUAD 002a58
Dufourea novaeangliae DNOVA 001c14
Lasioglossum albipes Lalb 05167
Habropoda laboriosa HLABO 002041
Megachile rotundata MROTU 001d03
Harpegnathos saltator HSAL14800
Atta cephalotes ACEP20953
Acromyrmex echinatior AECH19039
Wasmannia auropunctata 23054758
Cardiocondyla obscurior Cobs 01971
Monomorium pharaonis 5268058276
Vollenhovia emeryi 19029760
Linepithema humile LH21854
Camponotus floridanus CFLO15149
Cerapachys biroi EZA52628.1
Pogonomyrmex barbatus PB20743
Athalia rosae AROS000194
Cephus cinctus CCINC 0000ac
Orussus abietinus OABI003281
Polistes dominula 2259
Trichogramma pretiosum TPRE002585
Nasonia vitripennis Nasvi2EG007476
Copidosoma floridanum CFLO016441
Fopius arisanus 6704204
Blattella germanica BGER016201
Pediculus humanus PHUM347230
Danaus plexippus DPOGS208488 PA
Heliconius melpomene HMEL016437
Plutella xylostella Px000789
Bombyx mori BGIBMGA010339
Manduca sexta Msex2.06698
Papilio glaucus pgl996.15
Limnephilus lunatus LLUN008967
Diaphorina citri ref0023684
Pachypsylla venusta PVEN000879
Calligrapha
Leptinotarsa decemlineata LDEC018180
Anoplophora glabripennis AGLA005451
Dendroctonus ponderosae YQE 01972
Tribolium castaneum TC012547
Thanasimus
Rhyzobius
Agrilus planipennis APLA010427
Xylobiops
Onthophagus taurus OTAU000341
Gyrinus
Hydroscapha
Lepicerus
0.3
Anopheles quadriannulatus AQUA002576
Anopheles melas AMEC008310
Anopheles merus AMEM001807
Anopheles coluzzii ACOM034712
Anopheles arabiensis AARA009898
Anopheles gambiae AGAP003895
Anopheles christyi ACHR004908
Anopheles epiroticus AEPI004244
Anopheles culicifacies ACUA004745
Anopheles minimus AMIN000257
Anopheles funestus AFUN003673
Anopheles maculatus AMAM011486
Anopheles stephensi ASTE001919
Anopheles dirus ADIR003386
Anopheles farauti AFAF020972
Anopheles sinensis ASIC002149
Anopheles atroparvus AATE016931
Anopheles albimanus AALB008976
Anopheles darlingi ADAC009064
Aedes aegypti AAEL007144
Culex quinquefasciatus CPIJ003681
Polypedilum nubifer Pn.14197
Polypedilum vanderplanki Pv.13420
Belgica antarctica IU25 04813
Drosophila sechellia FBgn0165037
Drosophila simulans FBgn0192050
Drosophila melanogaster klhl10
Drosophila yakuba FBgn0232537
Drosophila erecta FBgn0113588
Drosophila willistoni Dwil GK14783
Drosophila suzukii DS10 00012157
Drosophila ananassae Dana GF16074
Drosophila mojavensis Dmoj GI17478
Drosophila virilis Dvir GJ14979
Drosophila grimshawi Dgri GH13286
Drosophila pseudoobscura FBgn0265243
Glossina austeni GAUT035268
Glossina pallidipes GPAI012863
Glossina morsitans GMOY006314
Glossina brevipalpis GBRI007060
Bactrocera cucurbitae 11856681
Bactrocera tryoni JHQJ01002357
Ceratitis capitata CCAP013391
Musca domestica 17020375
Mayetiola destructor Mdes003227
Lutzomyia longipalpis LLOTMP008079
Acyrthosiphon pisum ACYPI006327
Mengenilla moldrzyki mesp 9701
Cicindela
Cimex lectularius CLEC004320
Rhodnius prolixus RPRC006965
Halyomorpha halys HHAL009225
Oncopeltus fasciatus OFAS004663
Gerris buenoi GBUE004820
Homalodisca vitripennis HVIT000954
Zootermopsis nevadensis Znev 11937
Ladona fulva LFUL000829
Calopteryx splendens size297646.1
Diptera
Lepidoptera
Trichoptera
Coleoptera
Strepsiptera
Hymenoptera
Psocodea
Thysanoptera
Hemiptera
Blattodea

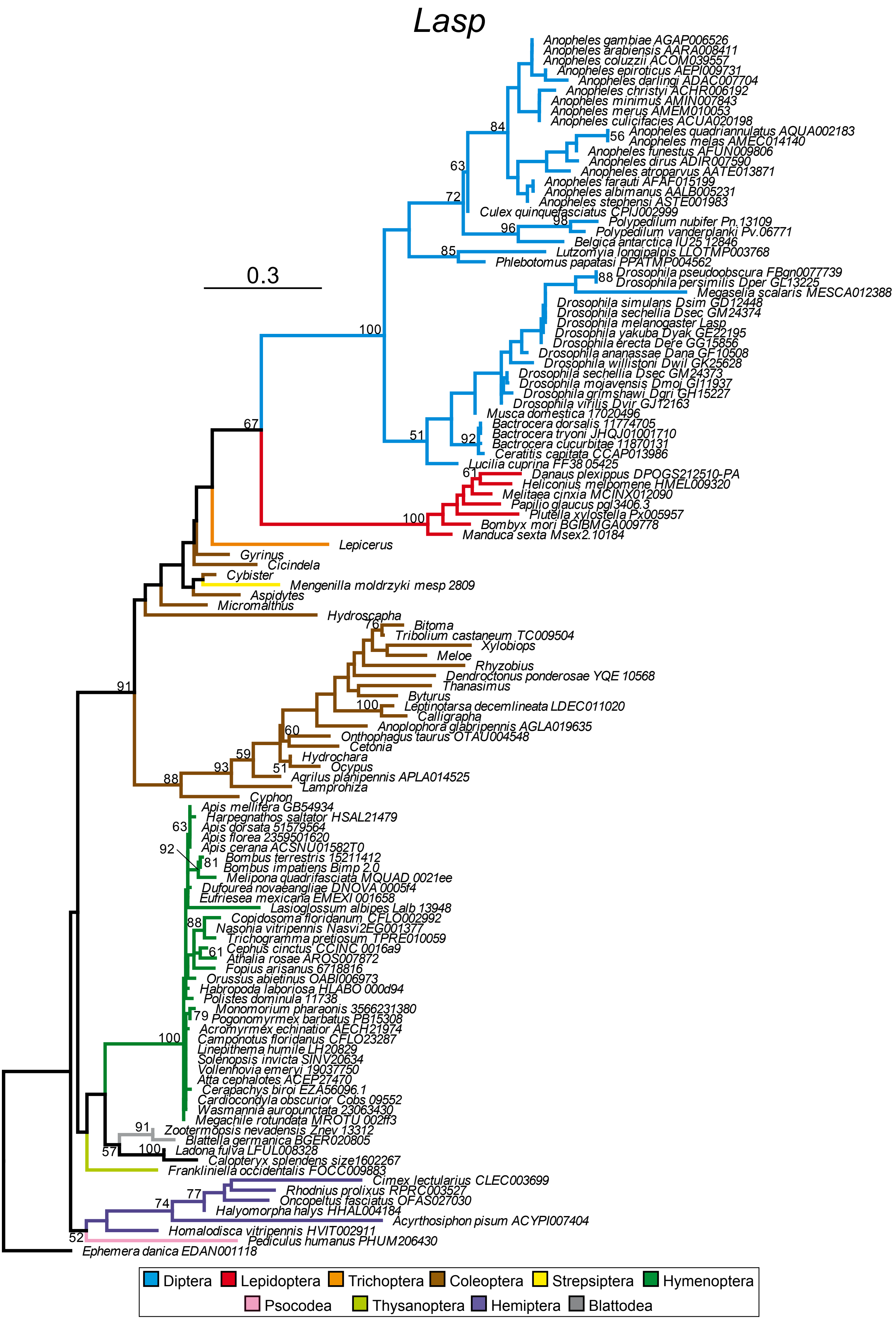

Lasp
0.3
Anopheles gambiae AGAP006526
Anopheles arabiensis AARA008411
Anopheles coluzzii ACOM039557
Anopheles epiroticus AEPI009731
Anopheles darlingi ADAC007704
Anopheles christyi ACHR006192
Anopheles minimus AMIN007843
Anopheles merus AMEM010053
Anopheles culicifacies ACUA020198
Anopheles quadriannulatus AQUA002183
Anopheles melas AMEC014140
Anopheles funestus AFUN009806
Anopheles dirus ADIR007590
Anopheles atroparvus AATE013871
Anopheles farauti AFAF015199
Anopheles albimanus AALB005231
Anopheles stephensi ASTE001983
Culex quinquefasciatus CPIJ002999
Polypedilum nubifer Pn.13109
Polypedilum vanderplanki Pv.06771
Belgica antarctica IU25 12846
Lutzomyia longipalpis LLOTMP003768
Phlebotomus papatasi PPATMP004562
Drosophila pseudoobscura FBgn0077739
Drosophila persimilis Dper GL13225
Megaselia scalaris MESCA012388
Drosophila simulans Dsim GD12448
Drosophila sechellia Dsec GM24374
Drosophila melanogaster Lasp
Drosophila yakuba Dyak GE22195
Drosophila erecta Dere GG15856
Drosophila ananassae Dana GF10508
Drosophila willistoni Dwil GK25628
Drosophila sechellia Dsec GM24373
Drosophila mojavensis Dmoj GI11937
Drosophila grimshawi Dgri GH15227
Drosophila virilis Dvir GJ12163
Musca domestica 17020496
Bactrocera dorsalis 11774705
Bactrocera tryoni JHQJ01001710
Bactrocera cucurbitae 11870131
Ceratitis capitata CCAP013986
Lucilia cuprina FF38 05425
Danaus plexippus DPOGS212510-PA
Heliconius melpomene HMEL009320
Melitaea cinxia MCINX012090
Papilio glaucus pgl3406.3
Plutella xylostella Px005957
Bombyx mori BGIBMGA009778
Manduca sexta Msex2.10184
Lepicerus
Gyrinus
Cicindela
Cybister
Mengenilla moldrzyki mesp 2809
Aspidytes
Micromalthus
Hydroscapha
Bitoma
Tribolium castaneum TC009504
Xylobiops
Meloe
Rhyzobius
Dendroctonus ponderosae YQE 10568
Thanasimus
Byturus
Leptinotarsa decemlineata LDEC011020
Calligrapha
Anoplophora glabripennis AGLA019635
Onthophagus taurus OTAU004548
Cetonia
Hydrochara
Ocypus
Agrilus planipennis APLA014525
Lamprohiza
Cyphon
Apis mellifera GB54934
Harpegnathos saltator HSAL21479
Apis dorsata 51579564
Apis florea 2359501620
Apis cerana ACSNU01582T0
Bombus terrestris 15211412
Bombus impatiens Bimp 2.0
Melipona quadrifasciata MQUAD 0021ee
Dufourea novaeangliae DNOVA 0005f4
Eufriesea mexicana EMEXI 001658
Lasioglossum albipes Lalb 13948
Copidosoma floridanum CFLO002992
Nasonia vitripennis Nasvi2EG001377
Trichogramma pretiosum TPRE010059
Cephus cinctus CCINC 0016a9
Athalia rosae AROS007872
Fopius arisanus 6718816
Orussus abietinus OABI006973
Habropoda laboriosa HLABO 000d94
Polistes dominula 11738
Monomorium pharaonis 3566231380
Pogonomyrmex barbatus PB15308
Acromyrmex echinatior AECH21974
Camponotus floridanus CFLO23287
Linepithema humile LH20829
Solenopsis invicta SINV20634
Vollenhovia emeryi 19037750
Atta cephalotes ACEP27470
Cerapachys biroi EZA56096.1
Cardiocondyla obscurior Cobs 09552
Wasmannia auropunctata 23063430
Megachile rotundata MROTU 002ff3
Zootermopsis nevadensis Znev 13312
Blattella germanica BGER020805
Ladona fulva LFUL008328
Calopteryx splendens size1602267
Frankliniella occidentalis FOCC009883
Cimex lectularius CLEC003699
Rhodnius prolixus RPRC003527
Oncopeltus fasciatus OFAS027030
Halyomorpha halys HHAL004184
Acyrthosiphon pisum ACYPI007404
Homalodisca vitripennis HVIT002911
Pediculus humanus PHUM206430
Ephemera danica EDAN001118
Diptera
Lepidoptera
Trichoptera
Coleoptera
Strepsiptera
Hymenoptera
Psocodea
Thysanoptera
Hemiptera
Blattodea

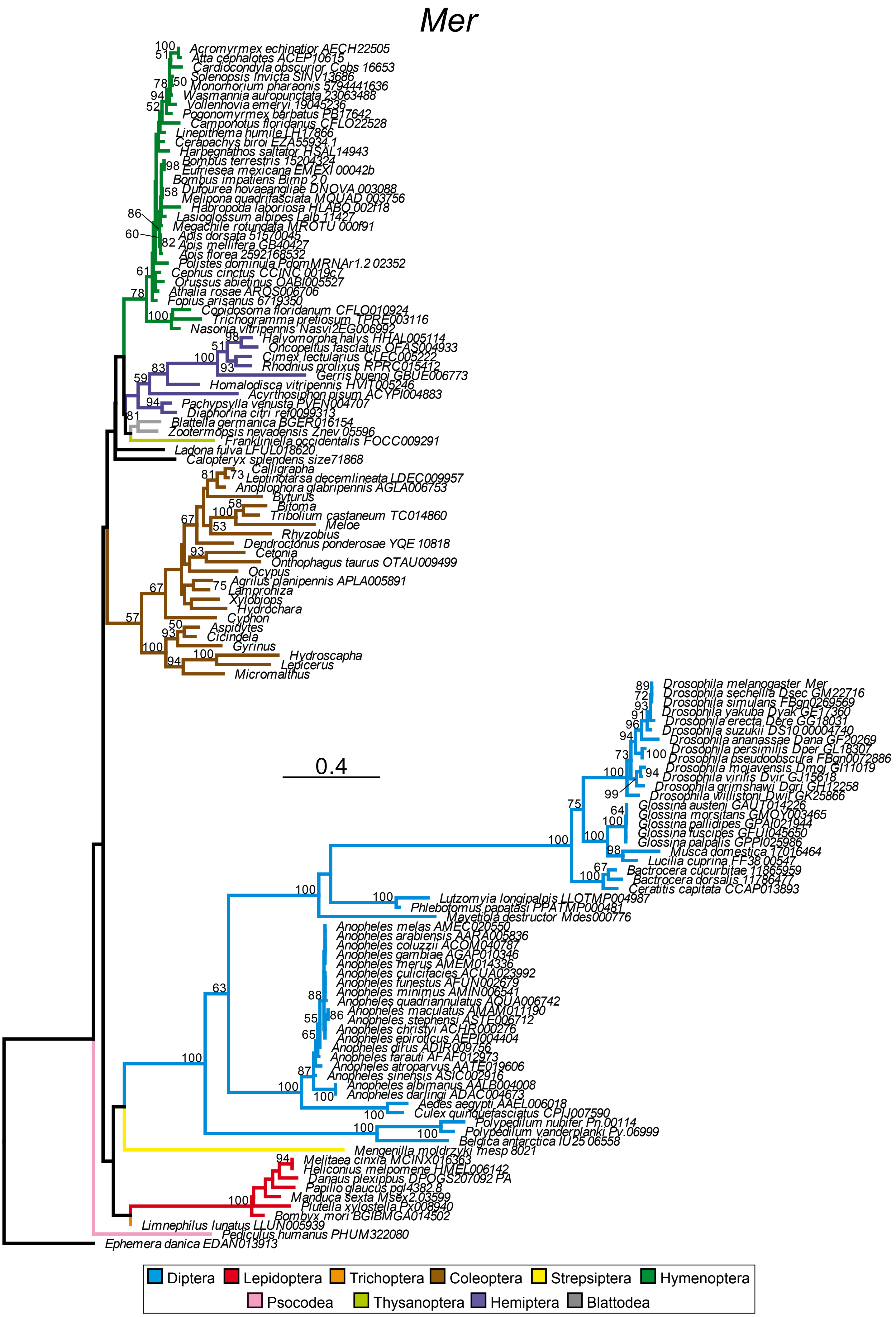
Mer
Acromyrmex echinatior AECH22505
Atta cephalotes ACEP10615
Cardiocondyla obscurior Cobs 16653
Solenopsis invicta SINV13686
Monomorium pharaonis 5794441636
Wasmannia auropunctata 23063488
Vollenhovia emeryi 19045236
Pogonomyrmex barbatus PB17642
Camponotus floridanus CFLO22528
Linepithema humile LH17866
Cerapachys biroi EZA55934.1
Harpegnathos saltator HSAL14943
Bombus terrestris 15204324
Eufriesea mexicana EMEXI 00042b
Bombus impatiens Bimp 2.0
Dufourea novaeangliae DNOVA 003088
Melipona quadrifasciata MQUAD 003756
Habropoda laboriosa HLABO 002f18
Lasioglossum albipes Lalb 11427
Megachile rotundata MROTU 000f91
Apis dorsata 51570045
Apis mellifera GB40427
Apis florea 2592168532
Polistes dominula PdomMRNAr1.2 02352
Cephus cinctus CCINC 0019c7
Orussus abietinus OABI005527
Athalia rosae AROS006706
Fopius arisanus 6719350
Copidosoma floridanum CFLO010924
Trichogramma pretiosum TPRE003116
Nasonia vitripennis Nasvi2EG006992
Halyomorpha halys HHAL005114
Oncopeltus fasciatus OFAS004933
Cimex lectularius CLEC005222
Rhodnius prolixus RPRC015412
Gerris buenoi GBUE006773
Homalodisca vitripennis HVIT005246
Acyrthosiphon pisum ACYPI004883
Pachypsylla venusta PVEN004707
Diaphorina citri ref0099313
Blattella germanica BGER016154
Zootermopsis nevadensis Znev 05596
Frankliniella occidentalis FOCC009291
Ladona fulva LFUL018620
Calopteryx splendens size71868
Calligrapha
Leptinotarsa decemlineata LDEC009957
Anoplophora glabripennis AGLA006753
Byturus
Bitoma
Tribolium castaneum TC014860
Meloe
Rhyzobius
Dendroctonus ponderosae YQE 10818
Cetonia
Onthophagus taurus OTAU009499
Ocypus
Agrilus planipennis APLA005891
Lamprohiza
Xylobiops
Hydrochara
Cyphon
Aspidytes
Cicindela
Gyrinus
Hydroscapha
Lepicerus
Micromalthus
0.4
Drosophila melanogaster Mer
Drosophila sechellia Dsec GM22716
Drosophila simulans FBgn0269569
Drosophila yakuba Dyak GE17360
Drosophila erecta Dere GG18031
Drosophila suzukii DS10 00004740
Drosophila ananassae Dana GF20269
Drosophila persimilis Dper GL18307
Drosophila pseudoobscura FBgn0072886
Drosophila mojavensis Dmoj GI11019
Drosophila virilis Dvir GJ15618
Drosophila grimshawi Dgri GH12258
Drosophila willistoni Dwil GK25866
Glossina austeni GAUT014226
Glossina morsitans GMOY003465
Glossina pallidipes GPAI021944
Glossina fuscipes GFUI045650
Glossina palpalis GPPI025986
Musca domestica 17016464
Lucilia cuprina FF38 00547
Bactrocera cucurbitae 11865959
Bactrocera dorsalis 11786477
Ceratitis capitata CCAP013893
Lutzomyia longipalpis LLOTMP004987
Phlebotomus papatasi PPATMP000481
Mayetiola destructor Mdes000776
Anopheles melas AMEC020550
Anopheles arabiensis AARA005836
Anopheles coluzzii ACOM040787
Anopheles gambiae AGAP010346
Anopheles merus AMEM014336
Anopheles culicifacies ACUA023992
Anopheles funestus AFUN002679
Anopheles minimus AMIN006541
Anopheles quadriannulatus AQUA006742
Anopheles maculatus AMAM011190
Anopheles stephensi ASTE006712
Anopheles christyi ACHR000276
Anopheles epiroticus AEPI004404
Anopheles dirus ADIR009756
Anopheles farauti AFAF012973
Anopheles atroparvus AATE019606
Anopheles sinensis ASIC002916
Anopheles albimanus AALB004008
Anopheles darlingi ADAC004673
Aedes aegypti AAEL006018
Culex quinquefasciatus CPIJ007590
Polypedilum nubifer Pn.00114
Polypedilum vanderplanki Pv.06999
Belgica antarctica IU25 06558
Mengenilla moldrzyki mesp 8021
Melitaea cinxia MCINX016363
Heliconius melpomene HMEL006142
Danaus plexippus DPOGS207092 PA
Papilio glaucus pgl4382.8
Manduca sexta Msex2.03599
Plutella xylostella Px008940
Bombyx mori BGIBMGA014502
Limnephilus lunatus LLUN005939
Pediculus humanus PHUM322080
Ephemera danica EDAN013913
Diptera
Lepidoptera
Trichoptera
Coleoptera
Strepsiptera
Hymenoptera
Psocodea
Thysanoptera
Hemiptera
Blattodea

mlt

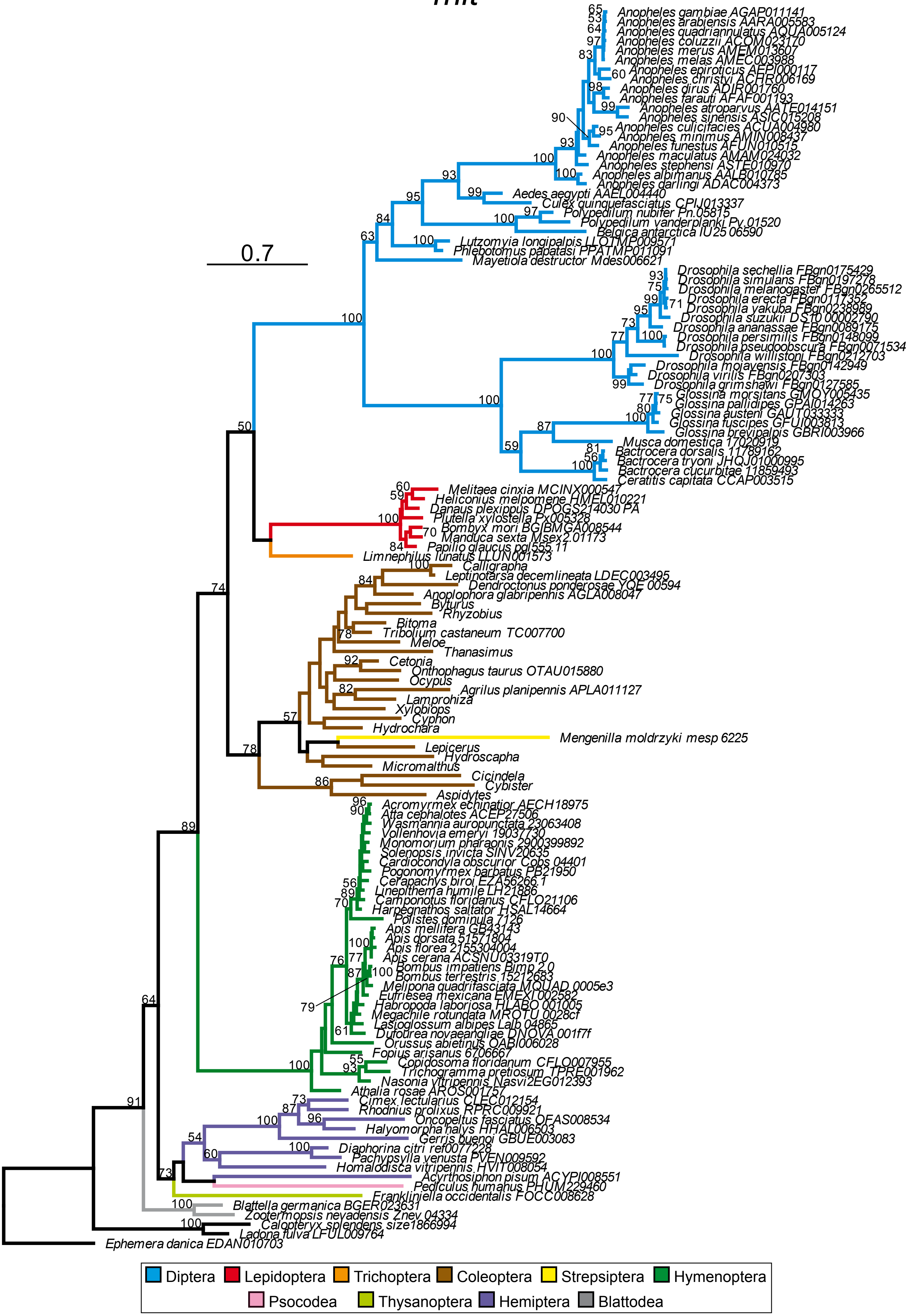

0.7
Anopheles gambiae AGAP011141
Anopheles arabiensis AARA005583
Anopheles quadriannulatus AQUA005124
Anopheles coluzzii ACOM023170
Anopheles merus AMEM013607
Anopheles melas AMEC003988
Anopheles epiroticus AEPI000117
Anopheles christyi ACHR006169
Anopheles dirus ADIR001760
Anopheles farauti AFAF001193
Anopheles atroparvus AATE014151
Anopheles sinensis ASIC015208
Anopheles culicifacies ACUA004980
Anopheles minimus AMIN008437
Anopheles funestus AFUN010515
Anopheles maculatus AMAM024032
Anopheles stephensi ASTE010970
Anopheles albimanus AALB010785
Anopheles darlingi ADAC004373
Aedes aegypti AAEL004440
Culex quinquefasciatus CPIJ013337
Polypedilum nubifer Pn.05815
Polypedilum vanderplanki Pv.01520
Belgica antarctica IU25 06590
Lutzomyia longipalpis LLOTMP009571
Phlebotomus papatasi PPATMP011091
Mayetiola destructor Mdes006621
Drosophila sechellia FBgn0175429
Drosophila simulans FBgn0197278
Drosophila melanogaster FBgn0265512
Drosophila erecta FBgn0117352
Drosophila yakuba FBgn0238989
Drosophila suzukii DS10 00002790
Drosophila ananassae FBgn0089175
Drosophila persimilis FBgn0148099
Drosophila pseudoobscura FBgn0071534
Drosophila willistoni FBgn0212703
Drosophila mojavensis FBgn0142949
Drosophila virilis FBgn0207303
Drosophila grimshawi FBgn0127585
Glossina morsitans GMOY005435
Glossina pallidipes GPAI014263
Glossina austeni GAUT033333
Glossina fuscipes GFUI003813
Glossina brevipalpis GBRI003966
Musca domestica 17020919
Bactrocera dorsalis 11789162
Bactrocera tryoni JHQJ01000995
Bactrocera cucurbitae 11859493
Ceratitis capitata CCAP003515
Melitaea cinxia MCINX000547
Heliconius melpomene HMEL010221
Danaus plexippus DPOGS214030 PA
Plutella xylostella Px005328
Bombyx mori BGIBMGA008544
Manduca sexta Msex2.01173
Papilio glaucus pgl555.11
Limnephilus lunatus LLUN001573
Calligrapha
Leptinotarsa decemlineata LDEC003495
Dendroctonus ponderosae YQE 00594
Anoplophora glabripennis AGLA008047
Byturus
Rhyzobius
Bitoma
Tribolium castaneum TC007700
Meloe
Thanasimus
Cetonia
Onthophagus taurus OTAU015880
Ocypus
Agrilus planipennis APLA011127
Lamprohiza
Xylobiops
Cyphon
Hydrochara
Mengenilla moldrzyki mesp 6225
Lepicerus
Hydroscapha
Micromalthus
Cicindela
Cybister
Aspidytes
Acromyrmex echinatior AECH18975
Atta cephalotes ACEP27506
Wasmannia auropunctata 23063408
Vollenhovia emeryi 19037730
Monomorium pharaonis 2900399892
Solenopsis invicta SINV20635
Cardiocondyla obscurior Cobs 04401
Pogonomyrmex barbatus PB21950
Cerapachys biroi EZA56266.1
Linepithema humile LH21886
Camponotus floridanus CFLO21106
Harpegnathos saltator HSAL14664
Polistes dominula 7126
Apis mellifera GB43143
Apis dorsata 51571804
Apis florea 2155304004
Apis cerana ACSNU03319T0
Bombus impatiens Bimp 2.0
Bombus terrestris 15212683
Melipona quadrifasciata MQUAD 0005e3
Eufriesea mexicana EMEX1002582
Habropoda laboriosa HLABO 001005
Megachile rotundata MROTU 0028cf
Lasioglossum albipes Lalb 04865
Dufourea novaeangliae DNOVA 001f7f
Orussus abietinus OABI006028
Fopius arisanus 6706667
Copidosoma floridanum CFLO007955
Trichogramma pretiosum TPRE001962
Nasonia vitripennis Nasvi2EG012393
Athalia rosae AROS001757
Cimex lectularius CLEC012154
Rhodnius prolixus RPRC009921
Oncopeltus fasciatus OFAS008534
Halyomorpha halys HHAL006503
Gerris buenoi GBUE003083
Diaphorina citri ref0077228
Pachypsylla venusta PVEN009592
Homalodisca vitripennis HVIT008054
Acyrthosiphon pisum ACYPI008551
Pediculus humanus PHUM229460
Frankliniella occidentalis FOCC008628
Blattella germanica BGER023631
Zootermopsis nevadensis Znev 04334
Calopteryx splendens size1866994
Ladona fulva LFUL009764
Ephemera danica EDAN010703
Diptera
Lepidoptera
Trichoptera
Coleoptera
Strepsiptera
Hymenoptera
Psocodea
Thysanoptera
Hemiptera
Blattodea

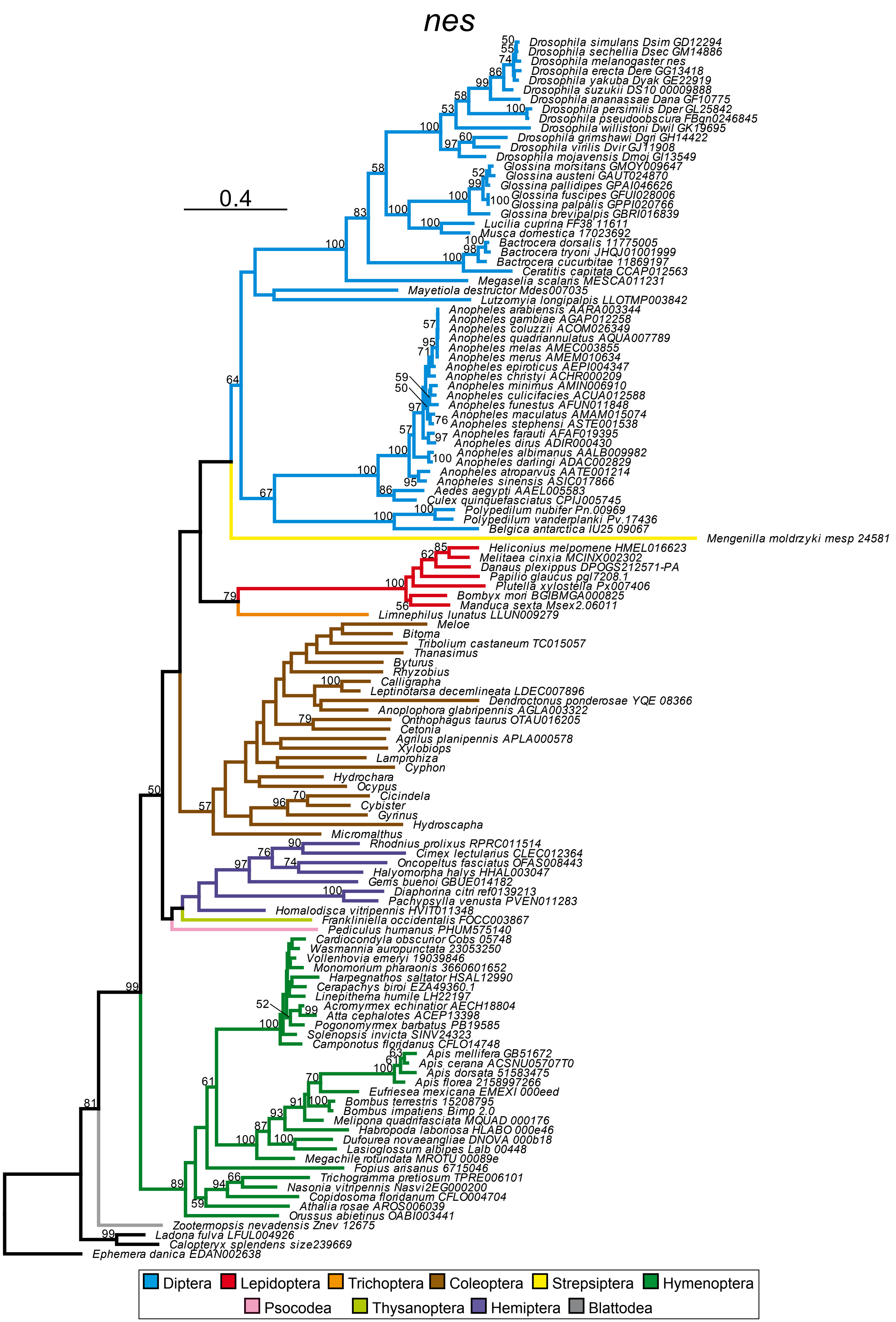
nes
0.4
Drosophila simulans Dsim GD12294
Drosophila sechellia Dsec GM14886
Drosophila melanogaster nes
Drosophila erecta Dere GG13418
Drosophila yakuba Dyak GE22919
Drosophila suzukii DS10 00009888
Drosophila ananassae Dana GF10775
Drosophila persimilis Dper GL25842
Drosophila pseudoobscura FBgn0246845
Drosophila willistoni Dwil GK19695
Drosophila grimshawi Dgri GH14422
Drosophila virilis Dvir GJ11908
Drosophila mojavensis Dmoj GI13549
Glossina morsitans GMOY009647
Glossina austeni GAUT024870
Glossina pallidipes GPAI046626
Glossina fuscipes GFUI028006
Glossina palpalis GPPI020766
Glossina brevipalpis GBRI016839
Lucilia cuprina FF38 11611
Musca domestica 17023692
Bactrocera dorsalis 11775005
Bactrocera tryoni JHQJ01001999
Bactrocera cucurbitae 11869197
Ceratitis capitata CCAP012563
Megaselia scalaris MESCA011231
Mayetiola destructor Mdes007035
Lutzomyia longipalpis LLOTMP003842
Anopheles arabiensis AARA003344
Anopheles gambiae AGAP012258
Anopheles coluzzii ACOM026349
Anopheles quadriannulatus AQUA007789
Anopheles melas AMEC003855
Anopheles merus AMEM010634
Anopheles epiroticus AEPI004347
Anopheles christyi ACHR000209
Anopheles minimus AMIN006910
Anopheles culicifacies ACUA012588
Anopheles funestus AFUN011848
Anopheles maculatus AMAM015074
Anopheles stephensi ASTE001538
Anopheles farauti AFAF019395
Anopheles dirus ADIR000430
Anopheles albimanus AALB009982
Anopheles darlingi ADAC002829
Anopheles atroparvus AATE001214
Anopheles sinensis ASIC017866
Aedes aegypti AAEL005583
Culex quinquefasciatus CPIJ005745
Polypedilum nubifer Pn.00969
Polypedilum vanderplanki Pv.17436
Belgica antarctica IU25 09067
Mengenilla moldrzyki mesp 24581
Heliconius melpomene HMEL016623
Melitaea cinxia MCINX002302
Danaus plexippus DPOGS212571-PA
Papilio glaucus pgl7208.1
Plutella xylostella Px007406
Bombyx mori BGIBMGA000825
Manduca sexta Msex2.06011
Limnephilus lunatus LLUN009279
Meloe
Bitoma
Tribolium castaneum TC015057
Thanasimus
Bytурus
Rhyzobius
Calligrapha
Leptinotarsa decemlineata LDEC007896
Dendroctonus ponderosae YQE 08366
Anoplophora glabripennis AGLA003322
Onthophagus taurus OTAU016205
Cetonia
Agrilus planipennis APLA000578
Xylobiops
Lamprohiza
Cyphon
Hydrochara
Ocypus
Cicindela
Cybister
Gyrinus
Hydroscapha
Micromalthus
Rhodnius prolixus RPRC011514
Cimex lectularius CLEC012364
Oncopeltus fasciatus OFAS008443
Halyomorpha halys HHAL003047
Gerris buenoi GBUE014182
Diaphorina citri ref0139213
Pachypsylla venusta PVEN011283
Homalodisca vitripennis HVIT011348
Frankliniella occidentalis FOCC003867
Pediculus humanus PHUM575140
Cardiocondyla obscurior Cobs 05748
Wasmannia auropunctata 23053250
Vollenhovia emeryi 19039846
Monomorium pharaonis 3660601652
Harpegnathos saltator HSAL12990
Cerapachys biroi EZA49360.1
Linepithema humile LH22197
Acromyrmex echinatior AECH18804
Atta cephalotes ACEP13398
Pogonomyrmex barbatus PB19585
Solenopsis invicta SINV24323
Camponotus floridanus CFLO14748
Apis mellifera GB51672
Apis cerana ACSNU05707T0
Apis dorsata 51583475
Apis florea 2158997266
Eufriesea mexicana EMEXI 000eed
Bombus terrestris 15208795
Bombus impatiens Bimp 2.0
Melipona quadrifasciata MQUAD 000176
Habropoda laboriosa HLABO 000e46
Dufourea novaeangliae DNOVA 000b18
Lasioglossum albipes Lalb 00448
Megachile rotundata MROTU 00089e
Fopius arisanus 6715046
Trichogramma pretiosum TPRE006101
Nasonia vitripennis Nasvi2EG000200
Copidosoma floridanum CFLO004704
Athalia rosae AROS006039
Orussus abietinus OABI003441
Zootermopsis nevadensis Znev 12675
Ladona fulva LFUL004926
Calopteryx splendens size239669
Ephemera danica EDAN002638
Diptera
Lepidoptera
Trichoptera
Coleoptera
Strepsiptera
Hymenoptera
Psocodea
Thysanoptera
Hemiptera
Blattodea

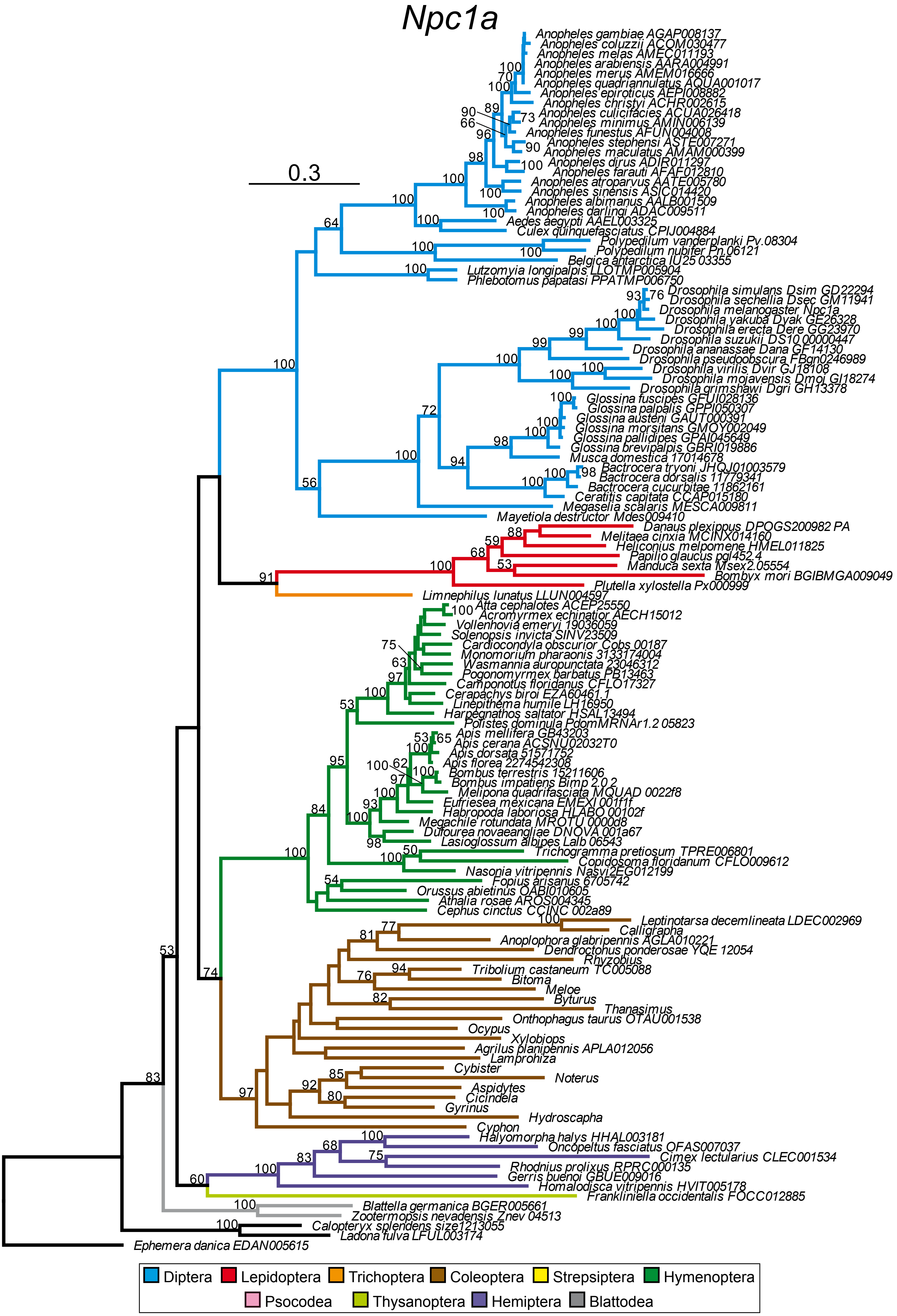
Npc1a
0.3
Anopheles gambiae AGAP008137
Anopheles coluzzii ACOM030477
Anopheles melas AMEC011193
Anopheles arabiensis AARA004991
Anopheles merus AMEM016666
Anopheles quadriannulatus AQUA001017
Anopheles epiroticus AEPI008882
Anopheles christyi ACHR002615
Anopheles culicifacies ACUA026418
Anopheles minimus AMIN006139
Anopheles funestus AFUN004008
Anopheles stephensi ASTE007271
Anopheles maculatus AMAM000399
Anopheles dirus ADIR011297
Anopheles farauti AFAF012810
Anopheles atroparvus AATE005780
Anopheles sinensis ASIC014420
Anopheles albimanus AALB001509
Anopheles darlingi ADAC009511
Aedes aegypti AAEL003325
Culex quinquefasciatus CPIJ004884
Polypedilum vanderplanki Pv.08304
Polypedilum nubifer Pn.06121
Belgica antarctica IU25 03355
Lutzomyia longipalpis LLOTMP005904
Phlebotomus papatasi PPATMP006750
Drosophila simulans Dsim GD22294
Drosophila sechellia Dsec GM11941
Drosophila melanogaster Npc1a
Drosophila yakuba Dyak GE26328
Drosophila erecta Dere GG23970
Drosophila suzukii DS10 00000447
Drosophila ananassae Dana GF14130
Drosophila pseudoobscura FBgn0246989
Drosophila virilis Dvir GJ18108
Drosophila mojavensis Dmoj GI18274
Drosophila grimshawi Dgri GH13378
Glossina fuscipes GFUI028136
Glossina palpalis GPPI050307
Glossina austeni GAUT000391
Glossina morsitans GMOY002049
Glossina pallidipes GPAI045649
Glossina brevipalpis GBRI019886
Musca domestica 17014678
Bactrocera tryoni JHQJ01003579
Bactrocera dorsalis 11779341
Bactrocera cucurbitae 11862161
Ceratitis capitata CCAP015180
Megaselia scalaris MESCA009811
Mayetiola destructor Mdes009410
Danaus plexippus DPOGS200982 PA
Melitaea cinxia MCINX014160
Heliconius melpomene HMEL011825
Papilio glaucus pgl452.4
Manduca sexta Msex2.05554
Bombyx mori BGIBMGA009049
Plutella xylostella Px000999
Limnephilus lunatus LLUN004597
Atta cephalotes ACEP25550
Acromyrmex echinatior AECH15012
Vollenhovia emeryi 19036059
Solenopsis invicta SINV23509
Cardiocondyla obscurior Cobs 00187
Monomorium pharaonis 3133174004
Wasmannia auropunctata 23046312
Pogonomyrmex barbatus PB13463
Camponotus floridanus CFLO17327
Cerapachys biroi EZA60461.1
Linepithema humile LH16950
Harpegnathos saltator HSAL13494
Polistes dominula PdomMRNAr1.2 05823
Apis mellifera GB43203
Apis cerana ACSNU02032T0
Apis dorsata 51571752
Apis florea 2274542308
Bombus terrestris 15211606
Bombus impatiens Bimp 2.0.2
Melipona quadrifasciata MQUAD 0022f8
Eufriesea mexicana EMEXI 001f1f
Habropoda laboriosa HLABO 00102f
Megachile rotundata MROTU 0000d8
Dufourea novaeangliae DNOVA 001a67
Lasioglossum albipes Lalb 06543
Trichogramma pretiosum TPRE006801
Copidosoma floridanum CFLO009612
Nasonia vitripennis Nasvi2EG012199
Fopius arisanus 6705742
Orussus abietinus OABI010605
Athalia rosae AROS004345
Cephus cinctus CCINC 002a89
Leptinotarsa decemlineata LDEC002969
Calligrapha
Anoplophora glabripennis AGLA010221
Dendroctonus ponderosae YQE 12054
Rhyzobius
Tribolium castaneum TC005088
Bitoma
Meloe
Byturus
Thanasimus
Onthophagus taurus OTAU001538
Ocypus
Xylobiops
Agrilus planipennis APLA012056
Lamprohiza
Cybister
Noterus
Aspidytes
Cicindela
Gyrinus
Hydroscapha
Cyphon
Halyomorpha halys HHAL003181
Oncopeltus fasciatus OFAS007037
Cimex lectularius CLEC001534
Rhodnius prolixus RPRC000135
Gerris buenoi GBUE009016
Homalodisca vitripennis HVIT005178
Frankliniella occidentalis FOCC012885
Blattella germanica BGER005661
Zootermopsis nevadensis Znev 04513
Calopteryx splendens size1213055
Ladona fulva LFUL003174
Ephemera danica EDAN005615
Diptera
Lepidoptera
Trichoptera
Coleoptera
Strepsiptera
Hymenoptera
Psocodea
Thysanoptera
Hemiptera
Blattodea

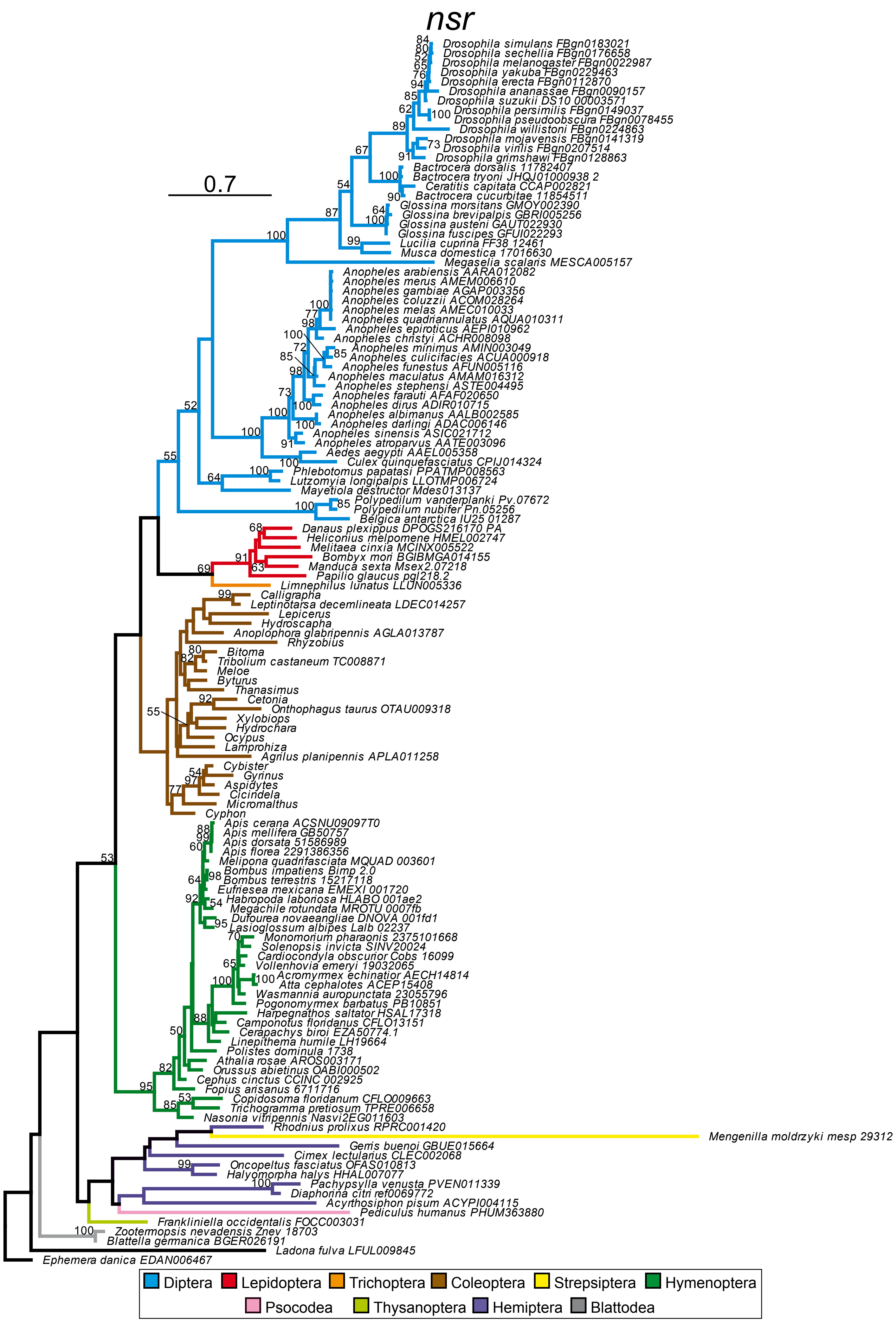

nsr
0.7
Drosophila simulans FBgn0183021
Drosophila sechellia FBgn0176658
Drosophila melanogaster FBgn0022987
Drosophila yakuba FBgn0229463
Drosophila erecta FBgn0112870
Drosophila ananassae FBgn0090157
Drosophila suzukii DS10 00003571
Drosophila persimilis FBgn0149037
Drosophila pseudoobscura FBgn0078455
Drosophila willistoni FBgn0224863
Drosophila mojavensis FBgn0141319
Drosophila virilis FBgn0207514
Drosophila grimshawi FBgn0128863
Bactrocera dorsalis 11782407
Bactrocera tryoni JHQJ01000938 2
Ceratitis capitata CCAP002821
Bactrocera cucurbitae 11854511
Glossina morsitans GMOY002390
Glossina brevipalpis GBRI005256
Glossina austeni GAUT022930
Glossina fuscipes GFUI022293
Lucilia cuprina FF38 12461
Musca domestica 17016630
Megaselia scalaris MESCA005157
Anopheles arabiensis AARA012082
Anopheles merus AMEM006610
Anopheles gambiae AGAP003356
Anopheles coluzzii ACOM028264
Anopheles melas AMEC010033
Anopheles quadriannulatus AQUA010311
Anopheles epiroticus AEPI010962
Anopheles christyi ACHR008098
Anopheles minimus AMIN003049
Anopheles culicifacies ACUA000918
Anopheles funestus AFUN005116
Anopheles maculatus AMAM016312
Anopheles stephensi ASTE004495
Anopheles farauti AFAF020650
Anopheles dirus ADIR010715
Anopheles albimanus AALB002585
Anopheles darlingi ADAC006146
Anopheles sinensis ASIC021712
Anopheles atroparvus AATE003096
Aedes aegypti AAEL005358
Culex quinquefasciatus CPIJ014324
Phlebotomus papatasi PPAPM008563
Lutzomyia longipalpis LLOTMP006724
Mayetiola destructor Mdes013137
Polypedilum vanderplanki Pv.07672
Polypedilum nubifer Pn.05256
Belgica antarctica IU25 01287
Danaus plexippus DPOGS216170 PA
Heliconius melpomene HMEL002747
Melitaea cinxia MCINX005522
Bombyx mori BGIBMGA014155
Manduca sexta Msex2.07218
Papilio glaucus pgl218.2
Limnephilus lunatus LLUN005336
Calligrapha
Leptinotarsa decemlineata LDEC014257
Lepicerus
Hydroscapha
Anoplophora glabripennis AGLA013787
Rhyzobius
Bitoma
Tribolium castaneum TC008871
Meloe
Byturus
Thanasimus
Cetonia
Onthophagus taurus OTAU009318
Xylobiops
Hydrochara
Ocypus
Lamprohiza
Agrilus planipennis APLA011258
Cybister
Gyrinus
Aspidytes
Cicindela
Micromalthus
Cyphon
Apis cerana ACSNU09097T0
Apis mellifera GB50757
Apis dorsata 51586989
Apis florea 2291386356
Melipona quadrifasciata MQUAD 003601
Bombus impatiens Bimp 2.0
Bombus terrestris 15217118
Eufriesea mexicana EMEXI 001720
Habropoda laboriosa HLABO 001ae2
Megachile rotundata MROTU 0007fb
Dufourea novaeangliae DNOVA 001fd1
Lasioglossum albipes Lalb 02237
Monomorium pharaonis 2375101668
Solenopsis invicta SINV20024
Cardiocondyla obscurior Cobs 16099
Vollenhovia emeryi 19032065
Acromyrmex echinatior AECH14814
Atta cephalotes ACEP15408
Wasmannia auropunctata 23055796
Pogonomyrmex barbatus PB10851
Harpegnathos saltator HSAL17318
Camponotus floridanus CFLO13151
Cerapachys biroi EZA50774.1
Linepithema humile LH19664
Polistes dominula 1738
Athalia rosae AROS003171
Orussus abietinus OABI000502
Cephus cinctus CCINC 002925
Fopius arisanus 6711716
Copidosoma floridanum CFLO009663
Trichogramma pretiosum TPRE006658
Nasonia vitripennis Nasvi2EG011603
Rhodnius prolixus RPRC001420
Mengenilla moldrzyki mesp 29312
Gerris buenoi GBUE015664
Cimex lectularius CLEC002068
Oncopeltus fasciatus OFAS010813
Halyomorpha halys HHAL007077
Pachypsylla venusta PVEN011339
Diaphorina citri ref0069772
Acyrthosiphon pisum ACYPI004115
Pediculus humanus PHUM363880
Frankliniella occidentalis FOCC003031
Zootermopsis nevadensis Znev 18703
Blattella germanica BGER026191
Ladona fulva LFUL009845
Ephemera danica EDAN006467
Diptera
Lepidoptera
Trichoptera
Coleoptera
Strepsiptera
Hymenoptera
Psocodea
Thysanoptera
Hemiptera
Blattodea

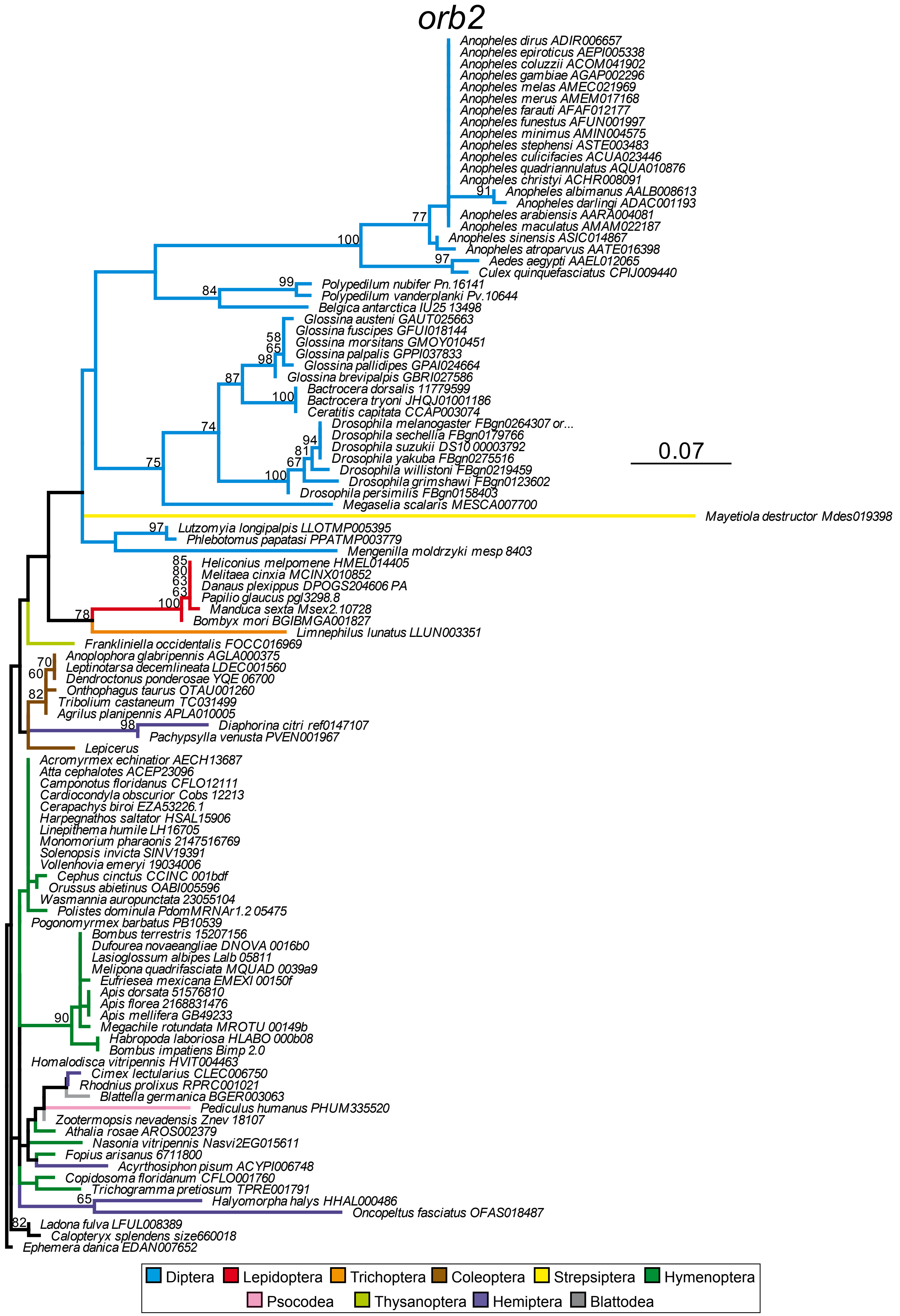
orb2
Anopheles dirus ADIR006657
Anopheles epiroticus AEPI005338
Anopheles coluzzii ACOM041902
Anopheles gambiae AGAP002296
Anopheles melas AMEC021969
Anopheles merus AMEM017168
Anopheles farauti AFAF012177
Anopheles funestus AFUN001997
Anopheles minimus AMIN004575
Anopheles stephensi ASTE003483
Anopheles culicifacies ACUA023446
Anopheles quadriannulatus AQUA010876
Anopheles christyi ACHR008091
Anopheles albimanus AALB008613
Anopheles darlingi ADAC001193
Anopheles arabiensis AARA004081
Anopheles maculatus AMAM022187
Anopheles sinensis ASIC014867
Anopheles atroparvus AATE016398
Aedes aegypti AAEL012065
Culex quinquefasciatus CPIJ009440
Polypedilum nubifer Pn.16141
Polypedilum vanderplanki Pv.10644
Belgica antarctica IU25 13498
Glossina austeni GAUT025663
Glossina fuscipes GFUI018144
Glossina morsitans GMOY010451
Glossina palpalis GPPI037833
Glossina pallidipes GPAI024664
Glossina brevipalpis GBRI027586
Bactrocera dorsalis 11779599
Bactrocera tryoni JHQJ01001186
Ceratitis capitata CCAP003074
Drosophila melanogaster FBgn0264307 or...
Drosophila sechellia FBgn0179766
Drosophila suzukii DS10 00003792
Drosophila yakuba FBgn0275516
Drosophila willistoni FBgn0219459
Drosophila grimshawi FBgn0123602
Drosophila persimilis FBgn0158403
Megaselia scalaris MESCA007700
Mayetiola destructor Mdes019398
Lutzomyia longipalpis LLOTMP005395
Phlebotomus papatasi PPATMP003779
Mengenilla moldrzyki mesp 8403
Heliconius melpomene HMEL014405
Melitaea cinxia MCINX010852
Danaus plexippus DPOGS204606 PA
Papilio glaucus pgl3298.8
Manduca sexta Msex2.10728
Bombyx mori BGIBMGA001827
Limnephilus lunatus LLUN003351
Frankliniella occidentalis FOCC016969
Anoplophora glabripennis AGLA000375
Leptinotarsa decemlineata LDEC001560
Dendroctonus ponderosae YQE 06700
Onthophagus taurus OTAU001260
Tribolium castaneum TC031499
Agrilus planipennis APLA010005
Diaphorina citri ref0147107
Pachypsylla venusta PVEN001967
Lepicerus
Acromyrmex echinatior AECH13687
Atta cephalotes ACEP23096
Camponotus floridanus CFLO12111
Cardiocondyla obscurior Cobs 12213
Cerapachys biroi EZA53226.1
Harpegnathos saltator HSAL15906
Linepithema humile LH16705
Monomorium pharaonis 2147516769
Solenopsis invicta SINV19391
Vollenhovia emeryi 19034006
Cephus cinctus CCINC 001bdf
Orussus abietinus OABI005596
Wasmannia auropunctata 23055104
Polistes dominula PdomMRNAr1.2 05475
Pogonomyrmex barbatus PB10539
Bombus terrestris 15207156
Dufourea novaeangliae DNOVA 0016b0
Lasioglossum albipes Lalb 05811
Melipona quadrifasciata MQUAD 0039a9
Eufriesea mexicana EMEXI 00150f
Apis dorsata 51576810
Apis florea 2168831476
Apis mellifera GB49233
Megachile rotundata MROTU 00149b
Habropoda laboriosa HLABO 000b08
Bombus impatiens Bimp 2.0
Homalodisca vitripennis HVIT004463
Cimex lectularius CLEC006750
Rhodnius prolixus RPRC001021
Blattella germanica BGER003063
Pediculus humanus PHUM335520
Zootermopsis nevadensis Znev 18107
Athalia rosae AROS002379
Nasonia vitripennis Nasvi2EG015611
Fopius arisanus 6711800
Acyrthosiphon pisum ACYPI006748
Copidosoma floridanum CFLO001760
Trichogramma pretiosum TPRE001791
Halyomorpha halys HHAL000486
Oncopeltus fasciatus OFAS018487
Ladona fulva LFUL008389
Calopteryx splendens size660018
Ephemera danica EDAN007652
0.07
Diptera
Lepidoptera
Trichoptera
Coleoptera
Strepsiptera
Hymenoptera
Psocodea
Thysanoptera
Hemiptera
Blattodea

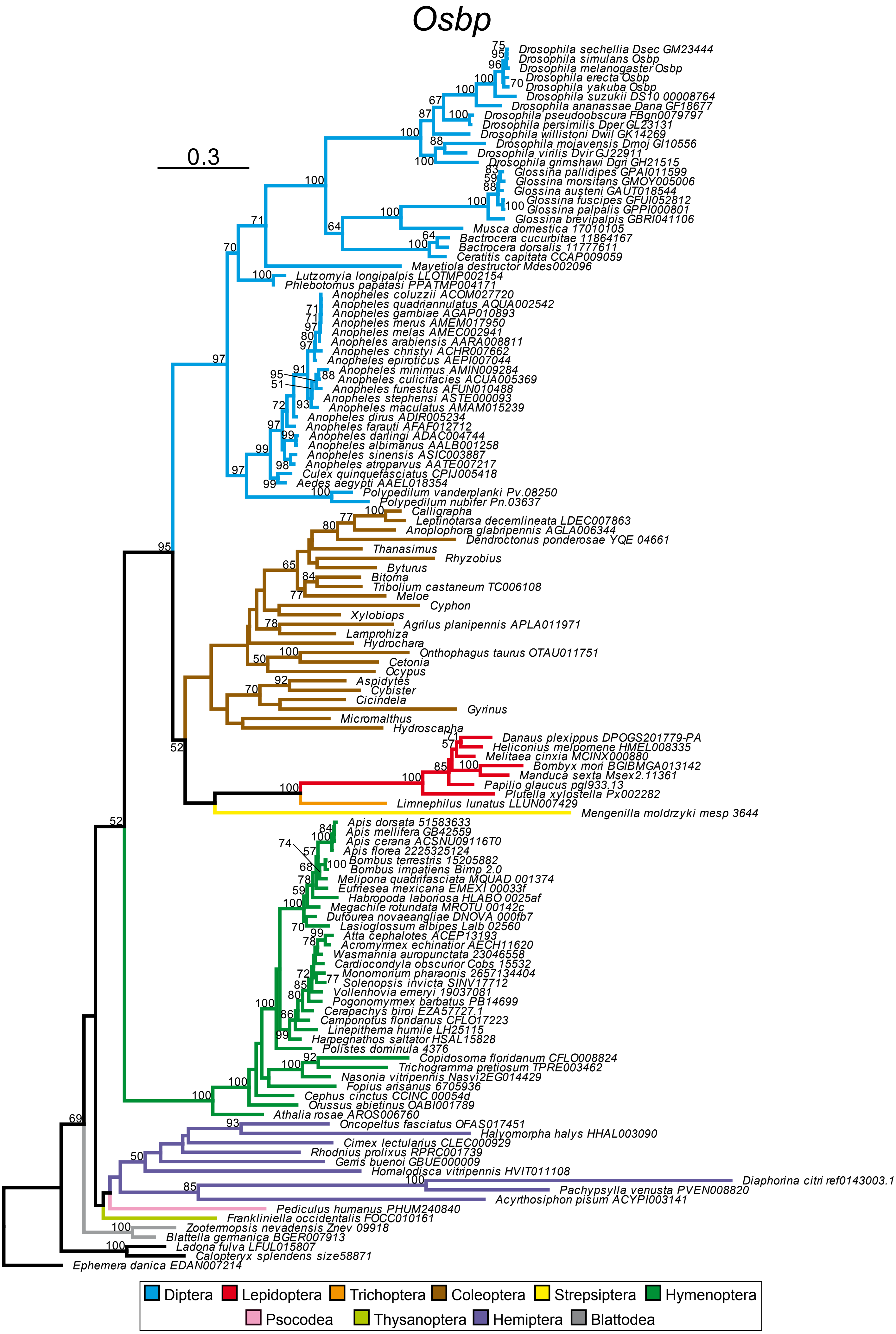

Osbp
0.3
Drosophila sechellia Dsec GM23444
Drosophila simulans Osbp
Drosophila melanogaster Osbp
Drosophila erecta Osbp
Drosophila yakuba Osbp
Drosophila suzukii DS10 00008764
Drosophila ananassae Dana GF18677
Drosophila pseudoobscura FBgn0079797
Drosophila persimilis Dper GL23131
Drosophila willistoni Dwil GK14269
Drosophila mojavensis Dmoj GI10556
Drosophila virilis Dvir GJ22911
Drosophila grimshawi Dgri GH21515
Glossina pallidipes GPAI011599
Glossina morsitans GMOY005006
Glossina austeni GAUT018544
Glossina fuscipes GFUI052812
Glossina palpalis GPPI000801
Glossina brevipalpis GBRI041106
Musca domestica 17010105
Bactrocera cucurbitae 11864167
Bactrocera dorsalis 11777611
Ceratitis capitata CCAP009059
Mayetiola destructor Mdes002096
Lutzomyia longipalpis LLOTMP002154
Phlebotomus papatasi PPATMP004171
Anopheles coluzzii ACOM027720
Anopheles quadriannulatus AQUA002542
Anopheles gambiae AGAP010893
Anopheles merus AMEM017950
Anopheles melas AMEC002941
Anopheles arabiensis AARA008811
Anopheles christyi ACHR007662
Anopheles epiroticus AEPI007044
Anopheles minimus AMIN009284
Anopheles culicifacies ACUA005369
Anopheles funestus AFUN010488
Anopheles stephensi ASTE000093
Anopheles maculatus AMAM015239
Anopheles dirus ADIR005234
Anopheles farauti AFAF012712
Anopheles darlingi ADAC004744
Anopheles albimanus AALB001258
Anopheles sinensis ASIC003887
Anopheles atroparvus AATE007217
Culex quinquefasciatus CPIJ005418
Aedes aegypti AAEL018354
Polypedilum vanderplanki Pv.08250
Polypedilum nubifer Pn.03637
Calligrapha
Leptinotarsa decemlineata LDEC007863
Anoplophora glabripennis AGLA006344
Dendroctonus ponderosae YQE 04661
Thanasimus
Rhyzobius
Bytурus
Bitoma
Tribolium castaneum TC006108
Meloe
Cyphon
Xylobiops
Agrilus planipennis APLA011971
Lamprohiza
Hydrochara
Onthophagus taurus OTAU011751
Cetonia
Ocypus
Aspidytes
Cybister
Cicindela
Gyrinus
Micromalthus
Hydroscapha
Danaus plexippus DPOGS201779-PA
Heliconius melpomene HMEL008335
Melitaea cinxia MCINX000880
Bombyx mori BGIBMGA013142
Manduca sexta Msex2.11361
Papilio glaucus pgl933.13
Plutella xylostella Px002282
Limnephilus lunatus LLUN007429
Mengenilla moldrzyki mesp 3644
Apis dorsata 51583633
Apis mellifera GB42559
Apis cerana ACSNU09116T0
Apis florea 2225325124
Bombus terrestris 15205882
Bombus impatiens Bimp 2.0
Melipona quadrifasciata MQUAD 001374
Eufriesea mexicana EMEXI 00033f
Habropoda laboriosa HLABO 0025af
Megachile rotundata MROTU 00142c
Dufourea novaeangliae DNOVA 000fb7
Lasioglossum albipes Lalb 02560
Atta cephalotes ACEP13193
Acromyrmex echinatior AECH11620
Wasmannia auropunctata 23046558
Cardiocondyla obscurior Cobs 15532
Monomorium pharaonis 2657134404
Solenopsis invicta SINV17712
Vollenhovia emeryi 19037081
Pogonomyrmex barbatus PB14699
Cerapachys biroi EZA57727.1
Camponotus floridanus CFLO17223
Linepithema humile LH25115
Harpegnathos saltator HSAL15828
Polistes dominula 4376
Copidosoma floridanum CFLO008824
Trichogramma pretiosum TPRE003462
Nasonia vitripennis Nasvi2EG014429
Fopius arisanus 6705936
Cephus cinctus CCINC 00054d
Orussus abietinus OABI001789
Athalia rosae AROS006760
Oncopeltus fasciatus OFAS017451
Halyomorpha halys HHAL003090
Cimex lectularius CLEC000929
Rhodnius prolixus RPRC001739
Gerris buenoi GBUE000009
Homalodisca vitripennis HVIT011108
Diaphorina citri ref0143003.1
Pachypsylla venusta PVEN008820
Acyrthosiphon pisum ACYPI003141
Pediculus humanus PHUM240840
Frankliniella occidentalis FOCC010161
Zootermopsis nevadensis Znev 09918
Blattella germanica BGER007913
Ladona fulva LFUL015807
Calopteryx splendens size58871
Ephemera danica EDAN007214
Diptera
Lepidoptera
Trichoptera
Coleoptera
Strepsiptera
Hymenoptera
Psocodea
Thysanoptera
Hemiptera
Blattodea

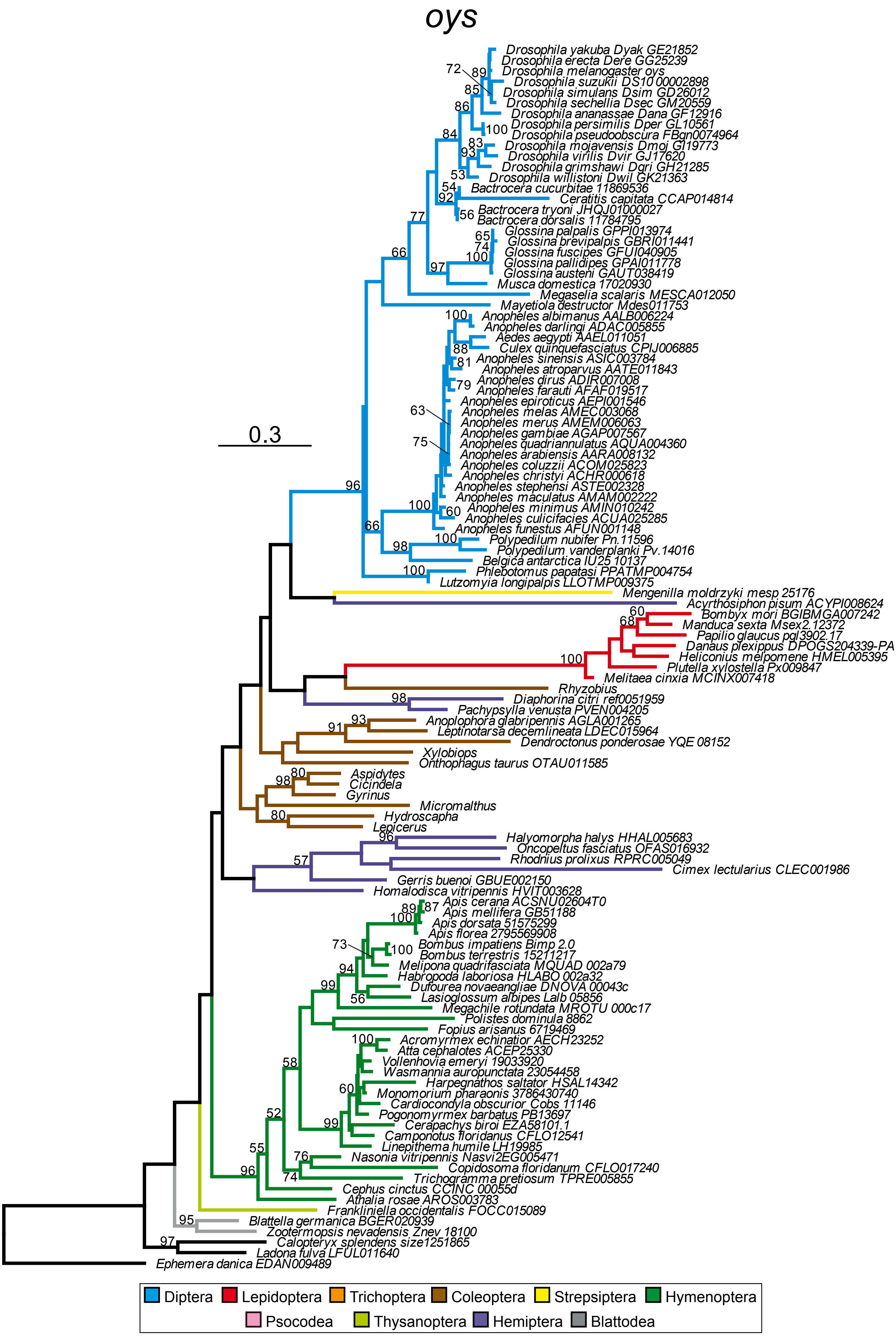

oys
0.3
Drosophila yakuba Dyak GE21852
Drosophila erecta Dere GG25239
Drosophila melanogaster oys
Drosophila suzukii DS10 00002898
Drosophila simulans Dsim GD26012
Drosophila sechellia Dsec GM20559
Drosophila ananassae Dana GF12916
Drosophila persimilis Dper GL10561
Drosophila pseudoobscura FBgn0074964
Drosophila mojavensis Dmoj GI19773
Drosophila virilis Dvir GJ17620
Drosophila grimshawi Dgri GH21285
Drosophila willistoni Dwil GK21363
Bactrocera cucurbitae 11869536
Ceratitis capitata CCAP014814
Bactrocera tryoni JHQJ01000027
Bactrocera dorsalis 11784795
Glossina palpalis GPPI013974
Glossina brevipalpis GBRI011441
Glossina fuscipes GFUI040905
Glossina pallidipes GPAI011778
Glossina austeni GAUT038419
Musca domestica 17020930
Megaselia scalaris MESCA012050
Mayetiola destructor Mdes011753
Anopheles albimanus AALB006224
Anopheles darlingi ADAC005855
Aedes aegypti AAEL011051
Culex quinquefasciatus CPIJ006885
Anopheles sinensis ASIC003784
Anopheles atroparvus AATE011843
Anopheles dirus ADIR007008
Anopheles farauti AFAF019517
Anopheles epiroticus AEPI001546
Anopheles melas AMEC003068
Anopheles merus AMEM006063
Anopheles gambiae AGAP007567
Anopheles quadriannulatus AQUA004360
Anopheles arabiensis AARA008132
Anopheles coluzzii ACOM025823
Anopheles christyi ACHR000618
Anopheles stephensi ASTE002328
Anopheles maculatus AMAM002222
Anopheles minimus AMIN010242
Anopheles culicifacies ACUA025285
Anopheles funestus AFUN001148
Polypedilum nubifer Pn.11596
Polypedilum vanderplanki Pv.14016
Belgica antarctica IU25 10137
Phlebotomus papatasi PPATMP004754
Lutzomyia longipalpis LLOTMP009375
Mengenilla moldrzyki mesp 25176
Acyrthosiphon pisum ACYPI008624
Bombyx mori BGIBMGA007242
Manduca sexta Msex2.12372
Papilio glaucus pgl3902.17
Danaus plexippus DPOGS204339-PA
Heliconius melpomene HMEL005395
Plutella xylostella Px009847
Melitaea cinxia MCINX007418
Rhyzobius
Diaphorina citri ref0051959
Pachypsylla venusta PVEN004205
Anoplophora glabripennis AGLA001265
Leptinotarsa decemlineata LDEC015964
Dendroctonus ponderosae YQE 08152
Xylobiops
Onthophagus taurus OTAU011585
Aspidytes
Cicindela
Gyrinus
Micromalthus
Hydroscapha
Lepicerus
Halyomorpha halys HHAL005683
Oncopeltus fasciatus OFAS016932
Rhodnius prolixus RPRC005049
Cimex lectularius CLEC001986
Gerris buenoi GBUE002150
Homalodisca vitripennis HVIT003628
Apis cerana ACSNU02604T0
Apis mellifera GB51188
Apis dorsata 51575299
Apis florea 2795569908
Bombus impatiens Bimp 2.0
Bombus terrestris 15211217
Melipona quadrifasciata MQUAD 002a79
Habropoda laboriosa HLABO 002a32
Dufourea novaeangliae DNOVA 00043c
Lasioglossum albipes Lalb 05856
Megachile rotundata MROTU 000c17
Polistes dominula 8862
Fopius arisanus 6719469
Acromyrmex echinatior AECH23252
Atta cephalotes ACEP25330
Vollenhovia emeryi 19033920
Wasmannia auropunctata 23054458
Harpegnathos saltator HSAL14342
Monomorium pharaonis 3786430740
Cardiocondyla obscurior Cobs 11146
Pogonomyrmex barbatus PB13697
Cerapachys biroi EZA58101.1
Camponotus floridanus CFLO12541
Linepithema humile LH19985
Nasonia vitripennis Nasvi2EG005471
Copidosoma floridanum CFLO017240
Trichogramma pretiosum TPRE005855
Cephus cinctus CCINC 00055d
Athalia rosae AROS003783
Frankliniella occidentalis FOCC015089
Blattella germanica BGER020939
Zootermopsis nevadensis Znev 18100
Calopteryx splendens size1251865
Ladona fulva LFUL011640
Ephemera danica EDAN009489
Diptera
Lepidoptera
Trichoptera
Coleoptera
Strepsiptera
Hymenoptera
Psocodea
Thysanoptera
Hemiptera
Blattodea

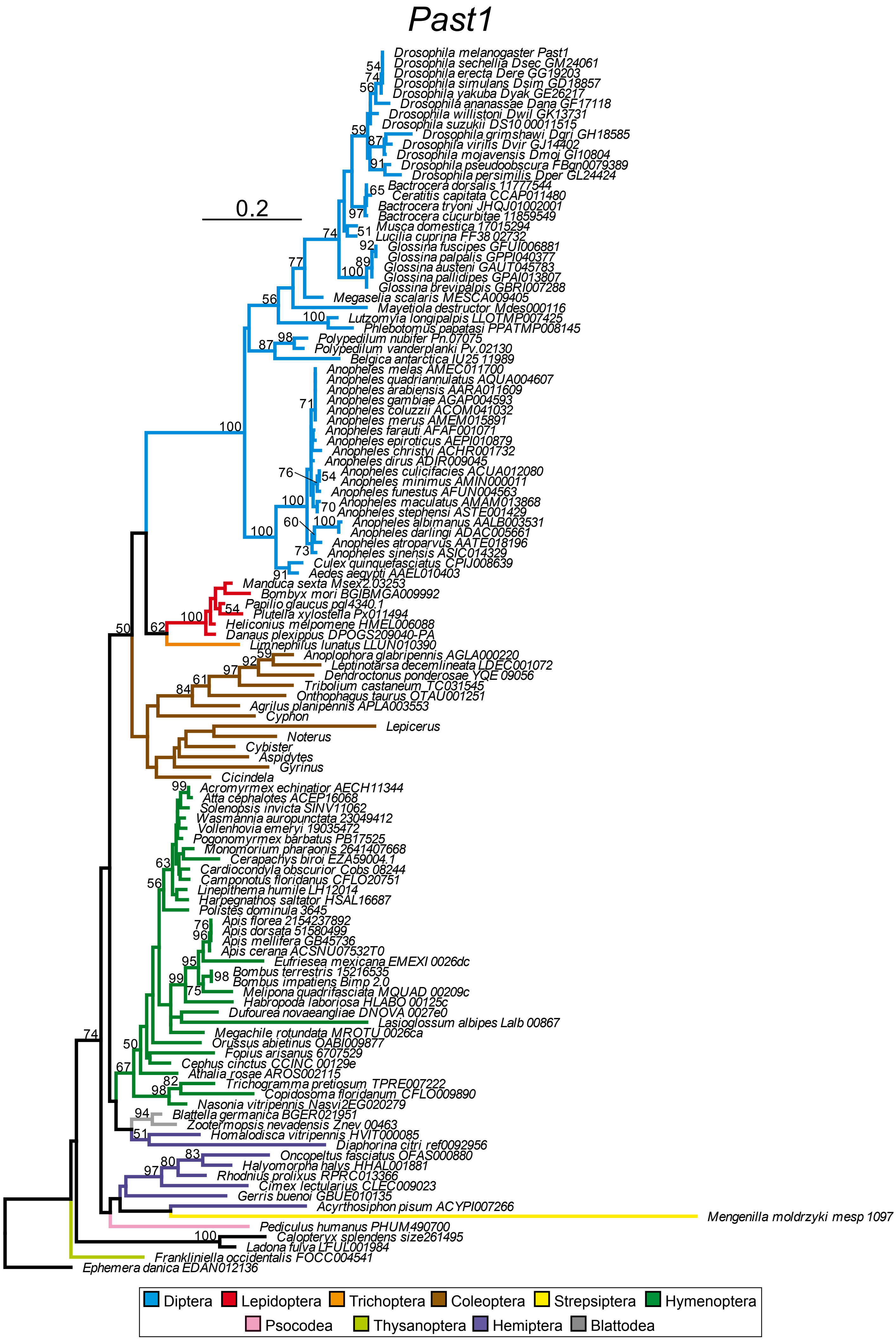

Past1
0.2
Drosophila melanogaster Past1
Drosophila sechellia Dsec GM24061
Drosophila erecta Dere GG19203
Drosophila simulans Dsim GD18857
Drosophila yakuba Dyak GE26217
Drosophila ananassae Dana GF17118
Drosophila willistoni Dwil GK13731
Drosophila suzukii DS10 00011515
Drosophila grimshawi Dgri GH18585
Drosophila virilis Dvir GJ14402
Drosophila mojavensis Dmoj GI10804
Drosophila pseudoobscura FBgn0079389
Drosophila persimilis Dper GL24424
Bactrocera dorsalis 11777544
Ceratitis capitata CCAP011480
Bactrocera tryoni JHQJ01002001
Bactrocera cucurbitae 11859549
Musca domestica 17015294
Lucilia cuprina FF38 02732
Glossina fuscipes GFUI006881
Glossina palpalis GPPI040377
Glossina austeni GAUT045783
Glossina pallidipes GPAI013807
Glossina brevipalpis GBRI007288
Megaselia scalaris MESCA009405
Mayetiola destructor Mdes000116
Lutzomyia longipalpis LLOTMP007425
Phlebotomus papatasi PPATMP008145
Polypedilum nubifer Pn.07075
Polypedilum vanderplanki Pv.02130
Belgica antarctica IU25 11989
Anopheles melas AMEC011700
Anopheles quadriannulatus AQUA004607
Anopheles arabiensis AARA011609
Anopheles gambiae AGAP004593
Anopheles coluzzii ACOM041032
Anopheles merus AMEM015891
Anopheles farauti AFAF001071
Anopheles epiroticus AEPI010879
Anopheles christyi ACHR001732
Anopheles dirus ADIR009045
Anopheles culicifacies ACUA012080
Anopheles minimus AMIN000011
Anopheles funestus AFUN004563
Anopheles maculatus AMAM013868
Anopheles stephensi ASTE001429
Anopheles albimanus AALB003531
Anopheles darlingi ADAC005661
Anopheles atroparvus AATE018196
Anopheles sinensis ASIC014329
Culex quinquefasciatus CPIJ008639
Aedes aegypti AAEL010403
Manduca sexta Msex2.03253
Bombyx mori BGIBMGA009992
Papilio glaucus pgl4340.1
Plutella xylostella Px011494
Heliconius melpomene HMEL006088
Danaus plexippus DPOGS209040-PA
Limnephilus lunatus LLUN010390
Anoplophora glabripennis AGLA000220
Leptinotarsa decemlineata LDEC001072
Dendroctonus ponderosae YQE 09056
Tribolium castaneum TC031545
Onthophagus taurus OTAU001251
Agrilus planipennis APLA003553
Cyphon
Lepicerus
Noterus
Cybister
Aspidytes
Gyrinus
Cicindela
Acromyrmex echinatior AECH11344
Atta cephalotes ACEP16068
Solenopsis invicta SINV11062
Wasmannia auropunctata 23049412
Vollenhovia emeryi 19035472
Pogonomyrmex barbatus PB17525
Monomorium pharaonis 2641407668
Cerapachys biroi EZA59004.1
Cardiocondyla obscurior Cobs 08244
Camponotus floridanus CFLO20751
Linepithema humile LH12014
Harpegnathos saltator HSAL16687
Polistes dominula 3645
Apis florea 2154237892
Apis dorsata 51580499
Apis mellifera GB45736
Apis cerana ACSNU07532T0
Eufriesea mexicana EMEXI 0026dc
Bombus terrestris 15216535
Bombus impatiens Bimp 2.0
Melipona quadrifasciata MQUAD 00209c
Habropoda laboriosa HLABO 00125c
Dufourea novaeangliae DNOVA 0027e0
Lasioglossum albipes Lalb 00867
Megachile rotundata MROTU 0026ca
Orussus abietinus OABI009877
Fopius arisanus 6707529
Cephus cinctus CCINC 00129e
Athalia rosae AROS002115
Trichogramma pretiosum TPRE007222
Copidosoma floridanum CFLO009890
Nasonia vitripennis Nasvi2EG020279
Blattella germanica BGER021951
Zootermopsis nevadensis Znev 00463
Homalodisca vitripennis HVIT000085
Diaphorina citri ref0092956
Oncopeltus fasciatus OFAS000880
Halyomorpha halys HHAL001881
Rhodnius prolixus RPRC013366
Cimex lectularius CLEC009023
Gerris buenoi GBUE010135
Acyrthosiphon pisum ACYPI007266
Mengenilla moldrzyki mesp 1097
Pediculus humanus PHUM490700
Calopteryx splendens size261495
Ladona fulva LFUL001984
Frankliniella occidentalis FOCC004541
Ephemera danica EDAN012136
Diptera
Lepidoptera
Trichoptera
Coleoptera
Strepsiptera
Hymenoptera
Psocodea
Thysanoptera
Hemiptera
Blattodea

Pen

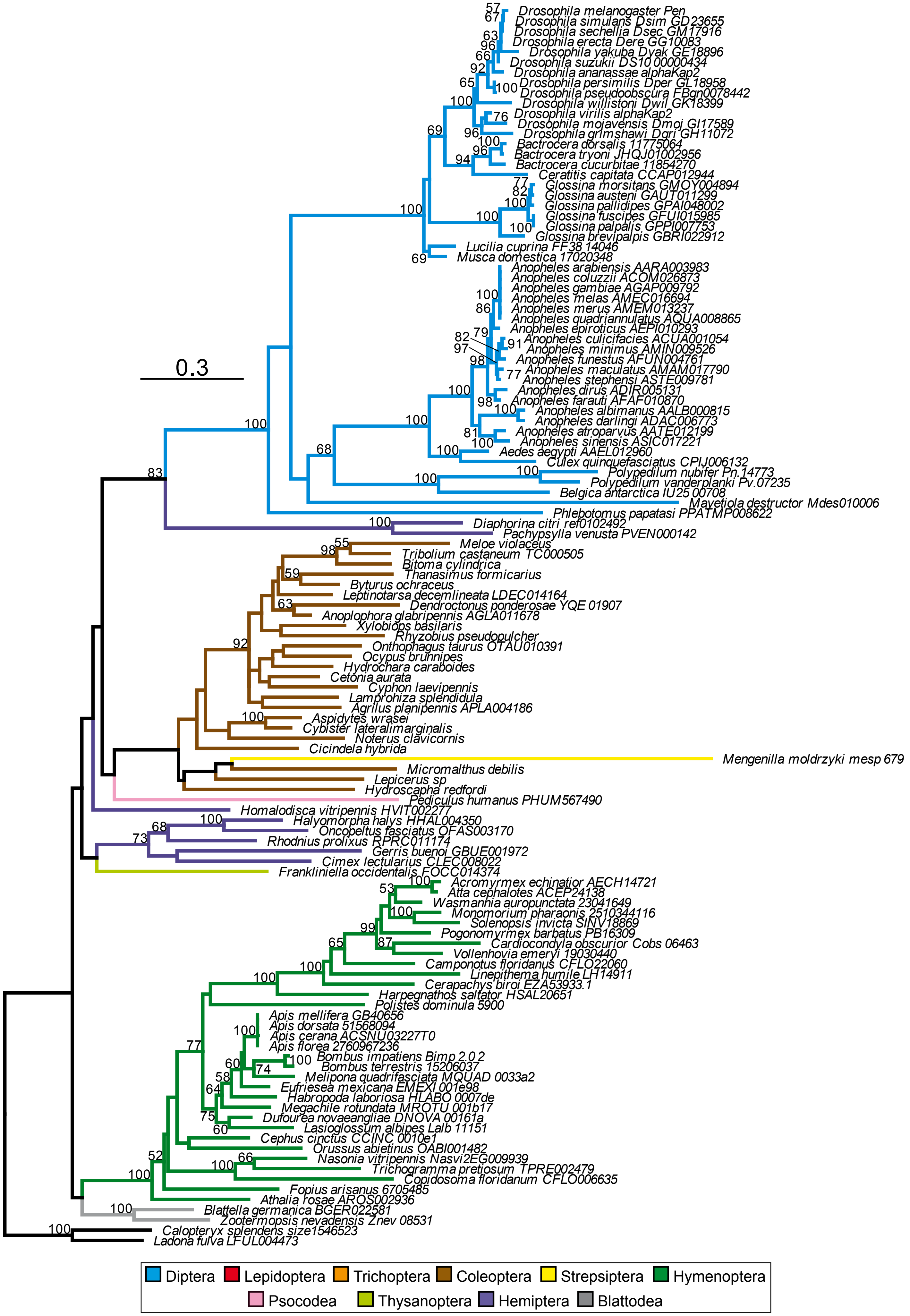
0.3
Drosophila melanogaster Pen
Drosophila simulans Dsim GD23655
Drosophila sechellia Dsec GM17916
Drosophila erecta Dere GG10083
Drosophila yakuba Dyak GE18896
Drosophila suzukii DS10 00000434
Drosophila ananassae alphaKap2
Drosophila persimilis Dper GL18958
Drosophila pseudoobscura FBgn0078442
Drosophila willistoni Dwil GK18399
Drosophila virilis alphaKap2
Drosophila mojavensis Dmoj GI17589
Drosophila grimshawi Dgri GH11072
Bactrocera dorsalis 11775064
Bactrocera tryoni JHQJ01002956
Bactrocera cucurbitae 11854270
Ceratitis capitata CCAP012944
Glossina morsitans GMOY004894
Glossina austeni GAUT011299
Glossina pallidipes GPAI048002
Glossina fuscipes GFUI015985
Glossina palpalis GPPI007753
Glossina brevipalpis GBRI022912
Lucilia cuprina FF38 14046
Musca domestica 17020348
Anopheles arabiensis AARA003983
Anopheles coluzzii ACOM026873
Anopheles gambiae AGAP009792
Anopheles melas AMEC016694
Anopheles merus AMEM013237
Anopheles quadriannulatus AQUA008865
Anopheles epiroticus AEPI010293
Anopheles culicifacies ACUA001054
Anopheles minimus AMIN009526
Anopheles funestus AFUN004761
Anopheles maculatus AMAM017790
Anopheles stephensi ASTE009781
Anopheles dirus ADIR005131
Anopheles farauti AFAF010870
Anopheles albimanus AALB000815
Anopheles darlingi ADAC006773
Anopheles atroparvus AATE012199
Anopheles sinensis ASIC017221
Aedes aegypti AAEL012960
Culex quinquefasciatus CPIJ006132
Polypedilum nubifer Pn.14773
Polypedilum vanderplanki Pv.07235
Belgica antarctica IU25 00708
Mayetiola destructor Mdes010006
Phlebotomus papatasi PPATMP008622
Diaphorina citri ref0102492
Pachypsylla venusta PVEN000142
Meloe violaceus
Tribolium castaneum TC000505
Bitoma cylindrica
Thanasimus formicarius
Byturus ochraceus
Leptinotarsa decemlineata LDEC014164
Dendroctonus ponderosae YQE 01907
Anoplophora glabripennis AGLA011678
Xylobiops basilaris
Rhyzobius pseudopulcher
Onthophagus taurus OTAU010391
Ocypus brunnipes
Hydrochara caraboides
Cetonia aurata
Cyphon laevipennis
Lamprohiza splendidula
Agrilus planipennis APLA004186
Aspidytes wrasei
Cybister lateralimarginalis
Noterus clavicornis
Cicindela hybrida
Mengenilla moldrzyki mesp 679
Micromalthus debilis
Lepicerus sp
Hydroscapha redfordi
Pediculus humanus PHUM567490
Homalodisca vitripennis HVIT002277
Halyomorpha halys HHAL004350
Oncopeltus fasciatus OFAS003170
Rhodnius prolixus RPRC011174
Gerris buenoi GBUE001972
Cimex lectularius CLEC008022
Frankliniella occidentalis FOCC014374
Acromyrmex echinatior AECH14721
Atta cephalotes ACEP24138
Wasmannia auropunctata 23041649
Monomorium pharaonis 2510344116
Solenopsis invicta SINV18869
Pogonomyrmex barbatus PB16309
Cardiocondyla obscurior Cobs 06463
Vollenhovia emeryi 19030440
Camponotus floridanus CFLO22060
Linepithema humile LH14911
Cerapachys biroi EZA53933.1
Harpegnathos saltator HSAL20651
Polistes dominula 5900
Apis mellifera GB40656
Apis dorsata 51568094
Apis cerana ACSNU03227T0
Apis florea 2760967236
Bombus impatiens Bimp 2.0.2
Bombus terrestris 15206037
Melipona quadrifasciata MQUAD 0033a2
Eufriesea mexicana EMEXI 001e98
Habropoda laboriosa HLABO 0007de
Megachile rotundata MROTU 001b17
Dufourea novaeangliae DNOVA 00161a
Lasioglossum albipes Lalb 11151
Cephus cinctus CCINC 0010e1
Orussus abietinus OABI001482
Nasonia vitripennis Nasvi2EG009939
Trichogramma pretiosum TPRE002479
Copidosoma floridanum CFLO006635
Fopius arisanus 6705485
Athalia rosae AROS002936
Blattella germanica BGER022581
Zootermopsis nevadensis Znev 08531
Calopteryx splendens size1546523
Ladona fulva LFUL004473
Diptera
Lepidoptera
Trichoptera
Coleoptera
Strepsiptera
Hymenoptera
Psocodea
Thysanoptera
Hemiptera
Blattodea

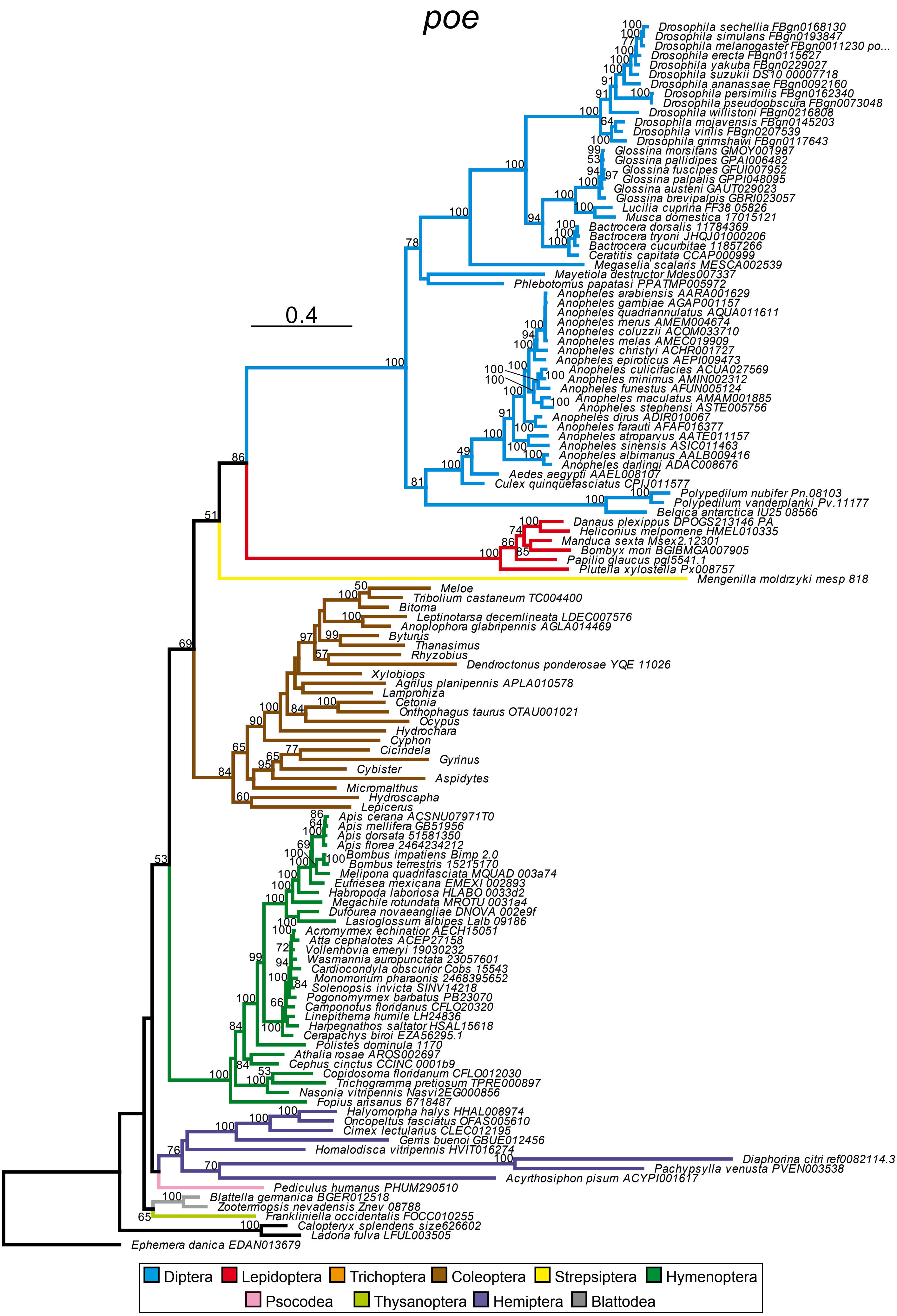
poe
0.4
Drosophila sechellia FBgn0168130
Drosophila simulans FBgn0193847
Drosophila melanogaster FBgn0011230 po...
Drosophila erecta FBgn0115627
Drosophila yakuba FBgn0229027
Drosophila suzukii DS10 00007718
Drosophila ananassae FBgn0092160
Drosophila persimilis FBgn0162340
Drosophila pseudoobscura FBgn0073048
Drosophila willistoni FBgn0216808
Drosophila mojavensis FBgn0145203
Drosophila virilis FBgn0207539
Drosophila grimshawi FBgn0117643
Glossina morsitans GMOY001987
Glossina pallidipes GPAI006482
Glossina fuscipes GFUI007952
Glossina palpalis GPPI048095
Glossina austeni GAUT029023
Glossina brevipalpis GBRI023057
Lucilia cuprina FF38 05826
Musca domestica 17015121
Bactrocera dorsalis 11784369
Bactrocera tryoni JHQJ01000206
Bactrocera cucurbitae 11857266
Ceratitis capitata CCAP000999
Megaselia scalaris MESCA002539
Mayetiola destructor Mdes007337
Phlebotomus papatasi PPATMP005972
Anopheles arabiensis AARA001629
Anopheles gambiae AGAP001157
Anopheles quadriannulatus AQUA011611
Anopheles merus AMEM004674
Anopheles coluzzii ACOM033710
Anopheles melas AMEC019909
Anopheles christyi ACHR001727
Anopheles epiroticus AEPI009473
Anopheles culicifacies ACUA027569
Anopheles minimus AMIN002312
Anopheles funestus AFUN005124
Anopheles maculatus AMAM001885
Anopheles stephensi ASTE005756
Anopheles dirus ADIR010067
Anopheles farauti AFAF016377
Anopheles atroparvus AATE011157
Anopheles sinensis ASIC011463
Anopheles albimanus AALB009416
Anopheles darlingi ADAC008676
Aedes aegypti AAEL008107
Culex quinquefasciatus CPIJ011577
Polypedilum nubifer Pn.08103
Polypedilum vanderplanki Pv.11177
Belgica antarctica IU25 08566
Danaus plexippus DPOGS213146 PA
Heliconius melpomene HMEL010335
Manduca sexta Msex2.12301
Bombyx mori BGIBMGA007905
Papilio glaucus pgl5541.1
Plutella xylostella Px008757
Mengenilla moldrzyki mesp 818
Meloe
Tribolium castaneum TC004400
Bitoma
Leptinotarsa decemlineata LDEC007576
Anoplophora glabripennis AGLA014469
Byturus
Thanasimus
Rhyzobius
Dendroctonus ponderosae YQE 11026
Xylobiops
Agrilus planipennis APLA010578
Lamprohiza
Cetonia
Onthophagus taurus OTAU001021
Ocypus
Hydrochara
Cyphon
Cicindela
Gyrinus
Cybister
Aspidytes
Micromalthus
Hydroscapha
Lepicerus
Apis cerana ACSNU07971T0
Apis mellifera GB51956
Apis dorsata 51581350
Apis florea 2464234212
Bombus impatiens Bimp 2.0
Bombus terrestris 15215170
Melipona quadrifasciata MQUAD 003a74
Eufriesea mexicana EMEXI 002893
Habropoda laboriosa HLABO 0033d2
Megachile rotundata MROTU 0031a4
Dufourea novaeangliae DNOVA 002e9f
Lasioglossum albipes Lalb 09186
Acromyrmex echinatior AECH15051
Atta cephalotes ACEP27158
Vollenhovia emeryi 19030232
Wasmannia auropunctata 23057601
Cardiocondyla obscurior Cobs 15543
Monomorium pharaonis 2468395652
Solenopsis invicta SINV14218
Pogonomyrmex barbatus PB23070
Camponotus floridanus CFLO20320
Linepithema humile LH24836
Harpegnathos saltator HSAL15618
Cerapachys biroi EZA56295.1
Polistes dominula 1170
Athalia rosae AROS002697
Cephus cinctus CCINC 0001b9
Copidosoma floridanum CFLO012030
Trichogramma pretiosum TPRE000897
Nasonia vitripennis Nasvi2EG000856
Fopius arisanus 6718487
Halyomorpha halys HHAL008974
Oncopeltus fasciatus OFAS005610
Cimex lectularius CLEC012195
Gerris buenoi GBUE012456
Homalodisca vitripennis HVIT016274
Diaphorina citri ref0082114.3
Pachypsylla venusta PVEN003538
Acyrthosiphon pisum ACYPI001617
Pediculus humanus PHUM290510
Blattella germanica BGER012518
Zootermopsis nevadensis Znev 08788
Frankliniella occidentalis FOCC010255
Calopteryx splendens size626602
Ladona fulva LFUL003505
Ephemera danica EDAN013679
Diptera
Lepidoptera
Trichoptera
Coleoptera
Strepsiptera
Hymenoptera
Psocodea
Thysanoptera
Hemiptera
Blattodea

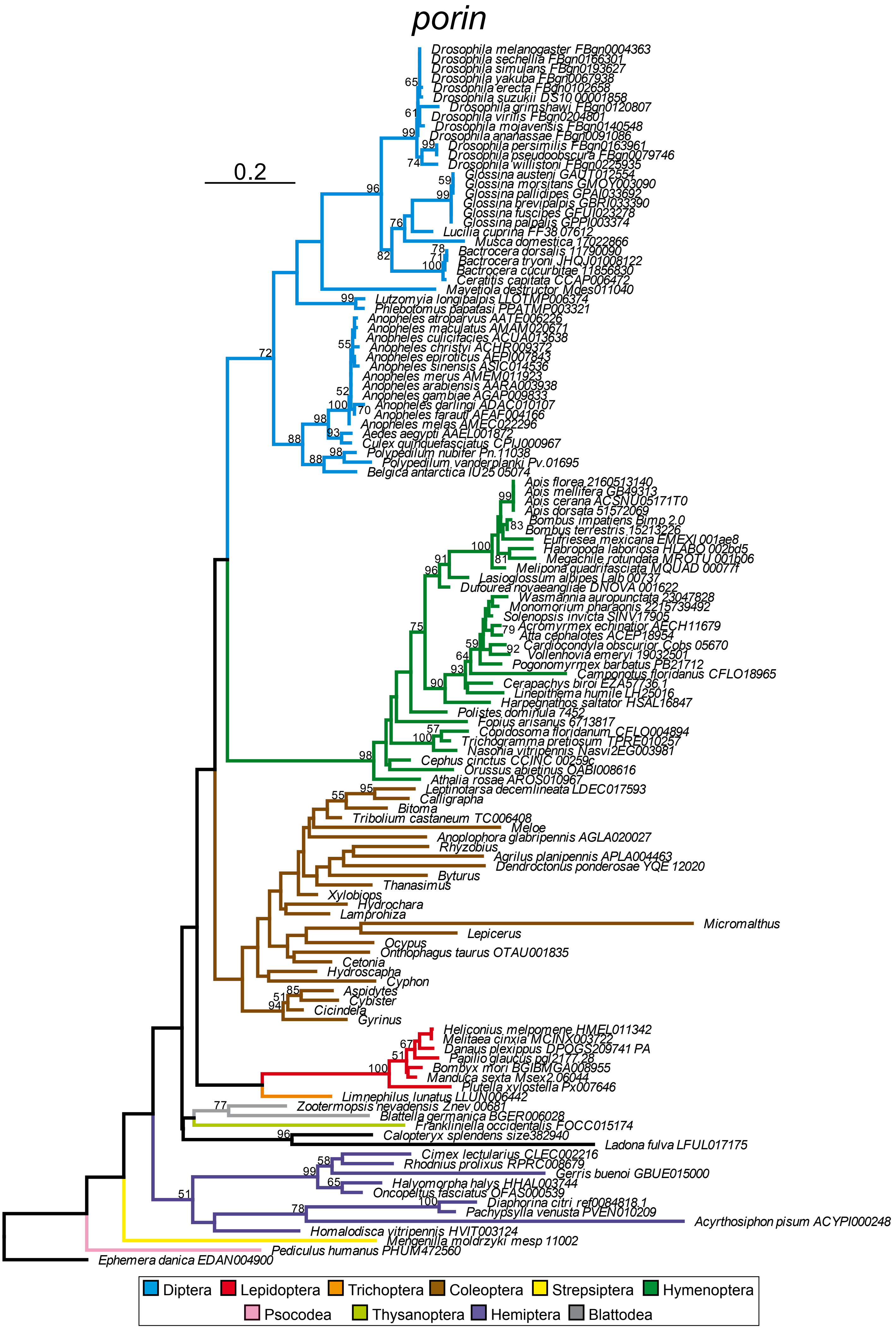

porin
0.2
Drosophila melanogaster FBgn0004363
Drosophila sechellia FBgn0166301
Drosophila simulans FBgn0193627
Drosophila yakuba FBgn0067938
Drosophila erecta FBgn0102658
Drosophila suzukii DS10 00001858
Drosophila grimshawi FBgn0120807
Drosophila virilis FBgn0204801
Drosophila mojavensis FBgn0140548
Drosophila ananassae FBgn0091086
Drosophila persimilis FBgn0163961
Drosophila pseudoobscura FBgn0079746
Drosophila willistoni FBgn0225935
Glossina austeni GAUT012554
Glossina morsitans GMOY003090
Glossina pallidipes GPAI033692
Glossina brevipalpis GBRI033390
Glossina fuscipes GFUI023278
Glossina palpalis GPPI003374
Lucilia cuprina FF38 07612
Musca domestica 17022866
Bactrocera dorsalis 11790090
Bactrocera tryoni JHQJ01008122
Bactrocera cucurbitae 11856830
Ceratitis capitata CCAP006472
Mayetiola destructor Mdes011040
Lutzomyia longipalpis LLOTMP006374
Phlebotomus papatasi PPATMP003321
Anopheles atroparvus AATE006226
Anopheles maculatus AMAM020671
Anopheles culicifacies ACUA013638
Anopheles christyi ACHR009372
Anopheles epiroticus AEPI007843
Anopheles sinensis ASIC014536
Anopheles merus AMEM011923
Anopheles arabiensis AARA003938
Anopheles gambiae AGAP009833
Anopheles darlingi ADAC010107
Anopheles farauti AFAF004166
Anopheles melas AMEC022296
Aedes aegypti AAEL001872
Culex quinquefasciatus CPIJ000967
Polypedilum nubifer Pn.11038
Polypedilum vanderplanki Pv.01695
Belgica antarctica IU25 05074
Apis florea 2160513140
Apis mellifera GB49313
Apis cerana ACSNU05171T0
Apis dorsata 51572069
Bombus impatiens Bimp 2.0
Bombus terrestris 15213226
Eufriesea mexicana EMEXI 001ae8
Habropoda laboriosa HLABO 002bd5
Megachile rotundata MROTU 001b06
Melipona quadrifasciata MQUAD 00077f
Lasioglossum albipes Lalb 00737
Dufourea novaeangliae DNOVA 001622
Wasmannia auropunctata 23047828
Monomorium pharaonis 2215739492
Solenopsis invicta SINV17905
Acromyrmex echinatior AECH11679
Atta cephalotes ACEP18954
Cardiocondyla obscurior Cobs 05670
Vollenhovia emeryi 19032501
Pogonomyrmex barbatus PB21712
Camponotus floridanus CFLO18965
Cerapachys biroi EZA57736.1
Linepithema humile LH25016
Harpegnathos saltator HSAL16847
Polistes dominula 7452
Fopius arisanus 6713817
Copidosoma floridanum CFLO004894
Trichogramma pretiosum TPRE010257
Nasonia vitripennis Nasvi2EG003981
Cephus cinctus CCINC 00259c
Orussus abietinus OABI008616
Athalia rosae AROS010967
Leptinotarsa decemlineata LDEC017593
Calligrapha
Bitoma
Tribolium castaneum TC006408
Meloe
Anoplophora glabripennis AGLA020027
Rhyzobius
Agrilus planipennis APLA004463
Dendroctonus ponderosae YQE 12020
Byturus
Thanasimus
Xylobiops
Hydrochara
Lamprohiza
Micromalthus
Lepicerus
Ocypus
Onthophagus taurus OTAU001835
Cetonia
Hydroscapha
Cyphon
Aspidytes
Cybister
Cicindela
Gyrinus
Heliconius melpomene HMEL011342
Melitaea cinxia MCINX003722
Danaus plexippus DPOGS209741 PA
Papilio glaucus pgl2177.28
Bombyx mori BGIBMGA008955
Manduca sexta Msex2.06044
Plutella xylostella Px007646
Limnephilus lunatus LLUN006442
Zootermopsis nevadensis Znev 00681
Blattella germanica BGER006028
Frankliniella occidentalis FOCC015174
Calopteryx splendens size382940
Ladona fulva LFUL017175
Cimex lectularius CLEC002216
Rhodnius prolixus RPRC008679
Gerris buenoi GBUE015000
Halyomorpha halys HHAL003744
Oncopeltus fasciatus OFAS000539
Diaphorina citri ref0084818.1
Pachypsylla venusta PVEN010209
Acyrthosiphon pisum ACYPI000248
Homalodisca vitripennis HVIT003124
Mengenilla moldrzyki mesp 11002
Pediculus humanus PHUM472560
Ephemera danica EDAN004900
Diptera
Lepidoptera
Trichoptera
Coleoptera
Strepsiptera
Hymenoptera
Psocodea
Thysanoptera
Hemiptera
Blattodea

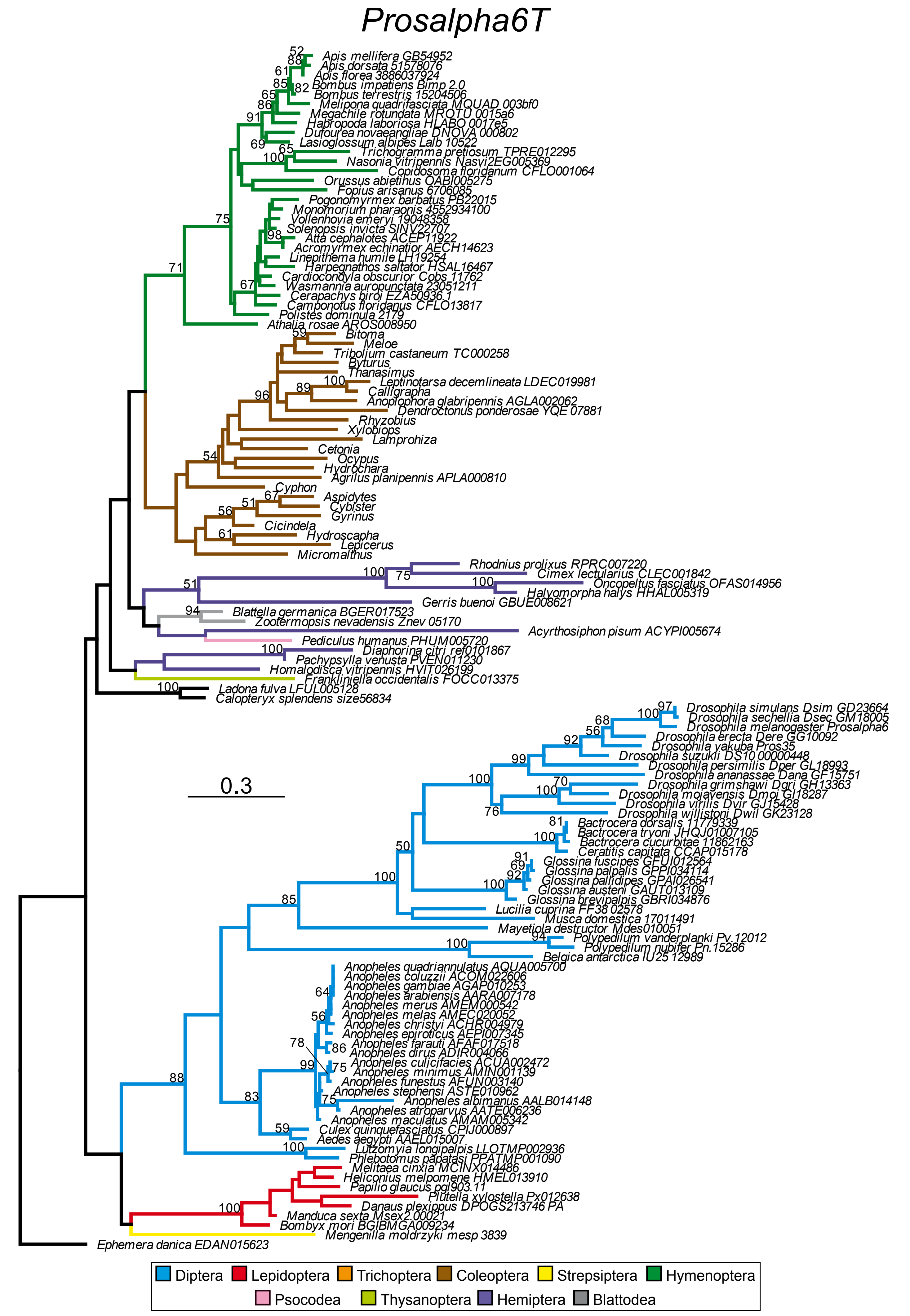
Prosalpha6T
Apis mellifera GB54952
Apis dorsata 51578076
Apis florea 3886037924
Bombus impatiens Bimp 2.0
Bombus terrestris 15204506
Melipona quadrifasciata MQUAD 003bf0
Megachile rotundata MROTU 0015a6
Habropoda laboriosa HLABO 0017e5
Dufourea novaeangliae DNOVA 000802
Lasioglossum albipes Lalb 10522
Trichogramma pretiosum TPRE012295
Nasonia vitripennis Nasvi2EG005369
Copidosoma floridanum CFLO001064
Orussus abietinus OABI005275
Fopius arisanus 6706085
Pogonomyrmex barbatus PB22015
Monomorium pharaonis 4552934100
Vollenhovia emeryi 19048358
Solenopsis invicta SINV22707
Atta cephalotes ACEP11922
Acromyrmex echinatior AECH14623
Linepithema humile LH19254
Harpegnathos saltator HSAL16467
Cardiocondyla obscurior Cobs 11762
Wasmannia auropunctata 23051211
Cerapachys biroi EZA50936.1
Camponotus floridanus CFLO13817
Polistes dominula 2179
Athalia rosae AROS008950
Bitoma
Meloe
Tribolium castaneum TC000258
Byturus
Thanasimus
Leptinotarsa decemlineata LDEC019981
Calligrapha
Anoplophora glabripennis AGLA002062
Dendroctonus ponderosae YQE 07881
Rhyzobius
Xylobiops
Lamprohiza
Cetonia
Ocypus
Hydrochara
Agrilus planipennis APLA000810
Cyphon
Aspidytes
Cybister
Gyrinus
Cicindela
Hydroscapha
Lepicerus
Micromalthus
Rhodnius prolixus RPRC007220
Cimex lectularius CLEC001842
Oncopeltus fasciatus OFAS014956
Halyomorpha halys HHAL005319
Gerris buenoi GBUE008621
Blattella germanica BGER017523
Zootermopsis nevadensis Znev 05170
Acyrthosiphon pisum ACYPI005674
Pediculus humanus PHUM005720
Diaphorina citri ref0101867
Pachypsylla venusta PVEN011230
Homalodisca vitripennis HVIT026199
Frankliniella occidentalis FOCC013375
Ladona fulva LFUL005128
Calopteryx splendens size56834
Drosophila simulans Dsim GD23664
Drosophila sechellia Dsec GM18005
Drosophila melanogaster Prosalpha6
Drosophila erecta Dere GG10092
Drosophila yakuba Pros35
Drosophila suzukii DS10 00000448
Drosophila persimilis Dper GL18993
Drosophila ananassae Dana GF15751
Drosophila grimshawi Dgri GH13363
Drosophila mojavensis Dmoj GI18287
Drosophila virilis Dvir GJ15428
Drosophila willistoni Dwil GK23128
Bactrocera dorsalis 11779339
Bactrocera tryoni JHQJ01007105
Bactrocera cucurbitae 11862163
Ceratitis capitata CCAP015178
Glossina fuscipes GFUI012564
Glossina palpalis GPPI034114
Glossina pallidipes GPAI026541
Glossina austeni GAUT013109
Glossina brevipalpis GBRI034876
Lucilia cuprina FF38 02578
Musca domestica 17011491
Mayetiola destructor Mdes010051
Polypedilum vanderplanki Pv.12012
Polypedilum nubifer Pn.15286
Belgica antarctica IU25 12989
Anopheles quadriannulatus AQUA005700
Anopheles coluzzii ACOM022606
Anopheles gambiae AGAP010253
Anopheles arabiensis AARA007178
Anopheles merus AMEM000542
Anopheles melas AMEC020052
Anopheles christyi ACHR004979
Anopheles epiroticus AEPI007345
Anopheles farauti AFAF017518
Anopheles dirus ADIR004066
Anopheles culicifacies ACUA002472
Anopheles minimus AMIN001139
Anopheles funestus AFUN003140
Anopheles stephensi ASTE010962
Anopheles albimanus AALB014148
Anopheles atroparvus AATE006236
Anopheles maculatus AMAM005342
Culex quinquefasciatus CPIJ000897
Aedes aegypti AAEL015007
Lutzomyia longipalpis LLOTMP002936
Phlebotomus papatasi PPATMP001090
Melitaea cinxia MCINX014486
Heliconius melpomene HMEL013910
Papilio glaucus pgl903.11
Plutella xylostella Px012638
Danaus plexippus DPOGS213746 PA
Manduca sexta Msex2.00021
Bombyx mori BGIBMGA009234
Mengenilla moldrzyki mesp 3839
Ephemera danica EDAN015623
0.3
Diptera
Lepidoptera
Trichoptera
Coleoptera
Strepsiptera
Hymenoptera
Psocodea
Thysanoptera
Hemiptera
Blattodea

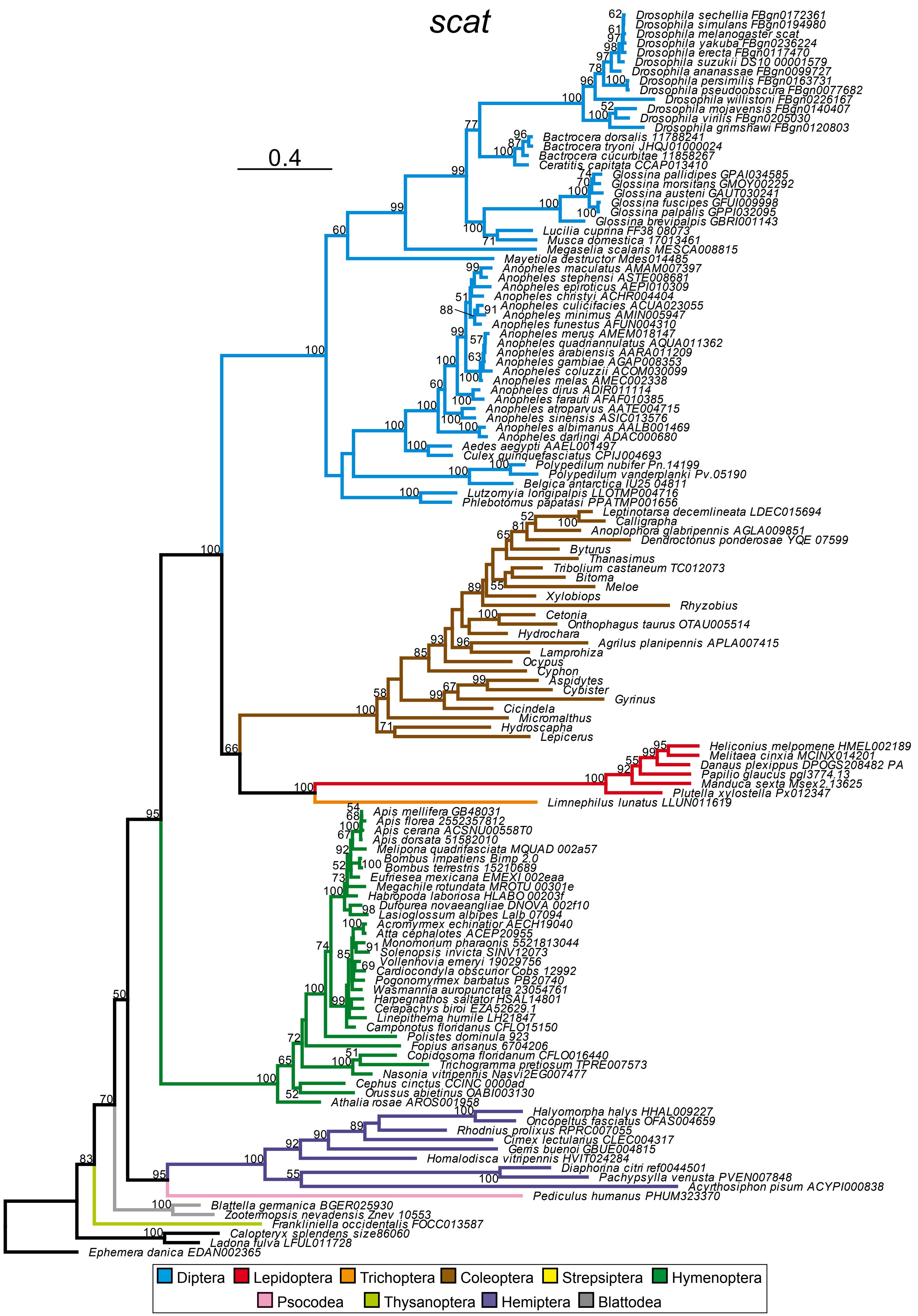
scat
0.4
Drosophila sechellia FBgn0172361
Drosophila simulans FBgn0194980
Drosophila melanogaster scat
Drosophila yakuba FBgn0236224
Drosophila erecta FBgn0117470
Drosophila suzukii DS10 00001579
Drosophila ananassae FBgn0099727
Drosophila persimilis FBgn0163731
Drosophila pseudoobscura FBgn0077682
Drosophila willistoni FBgn0226167
Drosophila mojavensis FBgn0140407
Drosophila virilis FBgn0205030
Drosophila grimshawi FBgn0120803
Bactrocera dorsalis 11788241
Bactrocera tryoni JHQJ01000024
Bactrocera cucurbitae 11858267
Ceratitis capitata CCAP013410
Glossina pallidipes GPAI034585
Glossina morsitans GMOY002292
Glossina austeni GAUT030241
Glossina fuscipes GFUI009998
Glossina palpalis GPPI032095
Glossina brevipalpis GBRI001143
Lucilia cuprina FF38 08073
Musca domestica 17013461
Megaselia scalaris MESCA008815
Mayetiola destructor Mdes014485
Anopheles maculatus AMAM007397
Anopheles stephensi ASTE008681
Anopheles epiroticus AEPI010309
Anopheles christyi ACHR004404
Anopheles culicifacies ACUA023055
Anopheles minimus AMIN005947
Anopheles funestus AFUN004310
Anopheles merus AMEM018147
Anopheles quadriannulatus AQUA011362
Anopheles arabiensis AARA011209
Anopheles gambiae AGAP008353
Anopheles coluzzii ACOM030099
Anopheles melas AMEC002338
Anopheles dirus ADIR011114
Anopheles farauti AFAF010385
Anopheles atroparvus AATE004715
Anopheles sinensis ASIC013576
Anopheles albimanus AALB001469
Anopheles darlingi ADAC000680
Aedes aegypti AAEL001497
Culex quinquefasciatus CPIJ004693
Polypedilum nubifer Pn.14199
Polypedilum vanderplanki Pv.05190
Belgica antarctica IU25 04811
Lutzomyia longipalpis LLOTMP004716
Phlebotomus papatasi PPATMP001656
Leptinotarsa decemlineata LDEC015694
Calligrapha
Anoplophora glabripennis AGLA009851
Dendroctonus ponderosae YQE 07599
Byturus
Thanasimus
Tribolium castaneum TC012073
Bitoma
Meloe
Xylobiops
Rhyzobius
Cetonia
Onthophagus taurus OTAU005514
Hydrochara
Agrilus planipennis APLA007415
Lamprohiza
Ocypus
Cyphon
Aspidytes
Cybister
Gyrinus
Cicindela
Micromalthus
Hydroscapha
Lepicerus
Heliconius melpomene HMEL002189
Melitaea cinxia MCINX014201
Danaus plexippus DPOGS208482 PA
Papilio glaucus pgl3774.13
Manduca sexta Msex2.13625
Plutella xylostella Px012347
Limnephilus lunatus LLUN011619
Apis mellifera GB48031
Apis florea 2552357812
Apis cerana ACSNU00558T0
Apis dorsata 51582010
Melipona quadrifasciata MQUAD 002a57
Bombus impatiens Bimp 2.0
Bombus terrestris 15210689
Eufriesea mexicana EMEXI 002eaa
Megachile rotundata MROTU 00301e
Habropoda laboriosa HLABO 00203f
Dufourea novaeangliae DNOVA 002f10
Lasioglossum albipes Lalb 07094
Acromyrmex echinatior AECH19040
Atta cephalotes ACEP20955
Monomorium pharaonis 5521813044
Solenopsis invicta SINV12073
Vollenhovia emeryi 19029756
Cardiocondyla obscurior Cobs 12992
Pogonomyrmex barbatus PB20740
Wasmannia auropunctata 23054761
Harpegnathos saltator HSAL14801
Cerapachys biroi EZA52629.1
Linepithema humile LH21847
Camponotus floridanus CFLO15150
Polistes dominula 923
Fopius arisanus 6704206
Copidosoma floridanum CFLO016440
Trichogramma pretiosum TPRE007573
Nasonia vitripennis Nasvi2EG007477
Cephus cinctus CCINC 0000ad
Orussus abietinus OABI003130
Athalia rosae AROS001958
Halyomorpha halys HHAL009227
Oncopeltus fasciatus OFAS004659
Rhodnius prolixus RPRC007055
Cimex lectularius CLEC004317
Gerris buenoi GBUE004815
Homalodisca vitripennis HVIT024284
Diaphorina citri ref0044501
Pachypsylla venusta PVEN007848
Acyrthosiphon pisum ACYPI000838
Pediculus humanus PHUM323370
Blattella germanica BGER025930
Zootermopsis nevadensis Znev 10553
Frankliniella occidentalis FOCC013587
Calopteryx splendens size86060
Ladona fulva LFUL011728
Ephemera danica EDAN002365
Diptera
Lepidoptera
Trichoptera
Coleoptera
Strepsiptera
Hymenoptera
Psocodea
Thysanoptera
Hemiptera
Blattodea

shi

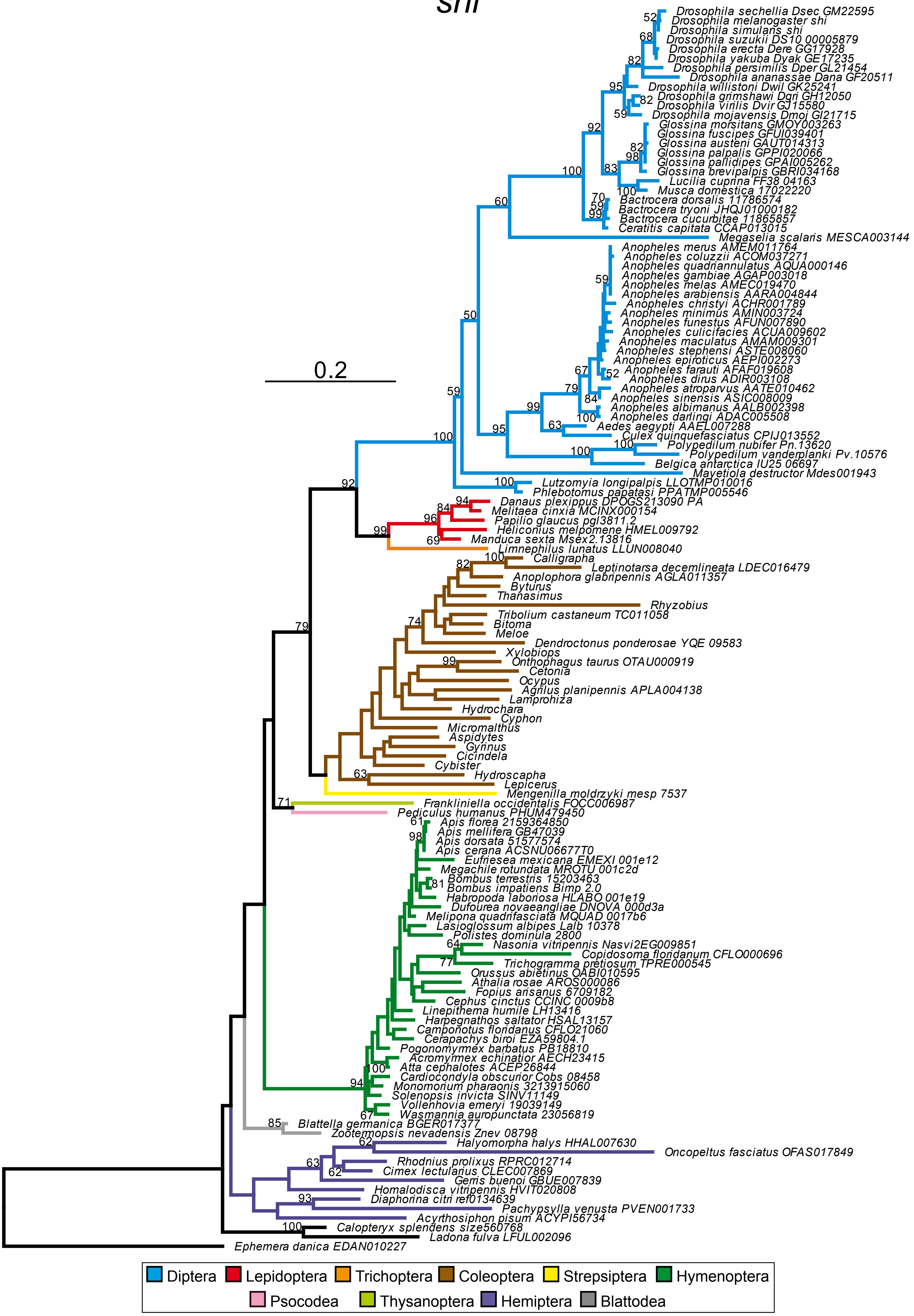
Drosophila sechellia Dsec GM22595
Drosophila melanogaster shi
Drosophila simulans shi
Drosophila suzukii DS10 00005879
Drosophila erecta Dere GG17928
Drosophila yakuba Dyak GE17235
Drosophila persimilis Dper GL21454
Drosophila ananassae Dana GF20511
Drosophila willistoni Dwil GK25241
Drosophila grimshawi Dgri GH12050
Drosophila virilis Dvir GJ15580
Drosophila mojavensis Dmoj GI21715
Glossina morsitans GMOY003263
Glossina fuscipes GFUI039401
Glossina austeni GAUT014313
Glossina palpalis GPPI020066
Glossina pallidipes GPAI005262
Glossina brevipalpis GBRI034168
Lucilia cuprina FF38 04163
Musca domestica 17022220
Bactrocera dorsalis 11786574
Bactrocera tryoni JHQJ01000182
Bactrocera cucurbitae 11865857
Ceratitis capitata CCAP013015
Megaselia scalaris MESCA003144
Anopheles merus AMEM011764
Anopheles coluzzii ACOM037271
Anopheles quadriannulatus AQUA000146
Anopheles gambiae AGAP003018
Anopheles melas AMEC019470
Anopheles arabiensis AARA004844
Anopheles christyi ACHR001789
Anopheles minimus AMIN003724
Anopheles funestus AFUN007890
Anopheles culicifacies ACUA009602
Anopheles maculatus AMAM009301
Anopheles stephensi ASTE008060
Anopheles epiroticus AEPI002273
Anopheles farauti AFAF019608
Anopheles dirus ADIR003108
Anopheles atroparvus AATE010462
Anopheles sinensis ASIC008009
Anopheles albimanus AALB002398
Anopheles darlingi ADAC005508
Aedes aegypti AAEL007288
Culex quinquefasciatus CPIJ013552
Polypedilum nubifer Pn.13620
Polypedilum vanderplanki Pv.10576
Belgica antarctica IU25 06697
Mayetiola destructor Mdes001943
Lutzomyia longipalpis LLOTMP010016
Phlebotomus papatasi PPATMP005546
Danaus plexippus DPOGS213090 PA
Melitaea cinxia MCINX000154
Papilio glaucus pgl3811.2
Heliconius melpomene HMEL009792
Manduca sexta Msex2.13816
Limnephilus lunatus LLUN008040
Calligrapha
Leptinotarsa decemlineata LDEC016479
Anoplophora glabripennis AGLA011357
Byturus
Thanasimus
Rhyzobius
Tribolium castaneum TC011058
Bitoma
Meloe
Dendroctonus ponderosae YQE 09583
Xylobiops
Onthophagus taurus OTAU000919
Cetonia
Ocypus
Agrilus planipennis APLA004138
Lamprohiza
Hydrochara
Cyphon
Micromalthus
Aspidytes
Gyrinus
Cicindela
Cybister
Hydroscapha
Lepicerus
Mengenilla moldrzyki mesp 7537
Frankliniella occidentalis FOCC006987
Pediculus humanus PHUM479450
Apis florea 2159364850
Apis mellifera GB47039
Apis dorsata 51577574
Apis cerana ACSNU06677T0
Eufriesea mexicana EMEXI 001e12
Megachile rotundata MROTU 001c2d
Bombus terrestris 15203463
Bombus impatiens Bimp 2.0
Habropoda laboriosa HLABO 001e19
Dufourea novaeangliae DNOVA 000d3a
Melipona quadrifasciata MQUAD 0017b6
Lasioglossum albipes Lalb 10378
Polistes dominula 2800
Nasonia vitripennis Nasvi2EG009851
Copidosoma floridanum CFLO000696
Trichogramma pretiosum TPRE000545
Orussus abietinus OABI010595
Athalia rosae AROS000086
Fopius arisanus 6709182
Cephus cinctus CCINC 0009b8
Linepithema humile LH13416
Harpegnathos saltator HSAL13157
Camponotus floridanus CFLO21060
Cerapachys biroi EZA59804.1
Pogonomyrmex barbatus PB18810
Acromyrmex echinatior AECH23415
Atta cephalotes ACEP26844
Cardiocondyla obscurior Cobs 08458
Monomorium pharaonis 3213915060
Solenopsis invicta SINV11149
Vollenhovia emeryi 19039149
Wasmannia auropunctata 23056819
Blattella germanica BGER017377
Zootermopsis nevadensis Znev 08798
Halyomorpha halys HHAL007630
Oncopeltus fasciatus OFAS017849
Rhodnius prolixus RPRC012714
Cimex lectularius CLEC007869
Gerris buenoi GBUE007839
Homalodisca vitripennis HVIT020808
Diaphorina citri ref0134639
Pachypsylla venusta PVEN001733
Acyrthosiphon pisum ACYPI56734
Calopteryx splendens size560768
Ladona fulva LFUL002096
Ephemera danica EDAN010227
0.2
Diptera
Lepidoptera
Trichoptera
Coleoptera
Strepsiptera
Hymenoptera
Psocodea
Thysanoptera
Hemiptera
Blattodea

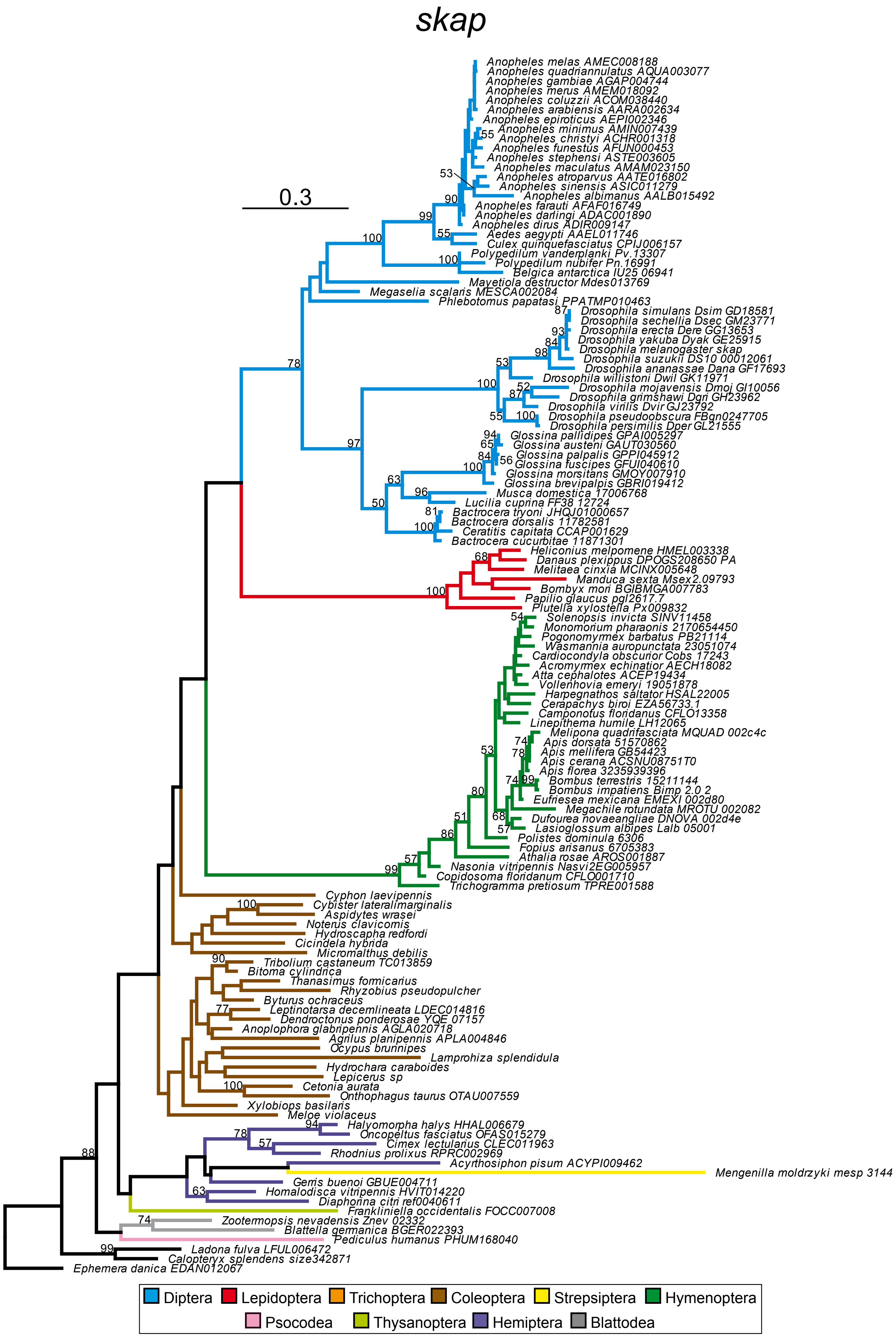

skap
0.3
Anopheles melas AMEC008188
Anopheles quadriannulatus AQUA003077
Anopheles gambiae AGAP004744
Anopheles merus AMEM018092
Anopheles coluzzii ACOM038440
Anopheles arabiensis AARA002634
Anopheles epiroticus AEPI002346
Anopheles minimus AMIN007439
Anopheles christyi ACHR001318
Anopheles funestus AFUN000453
Anopheles stephensi ASTE003605
Anopheles maculatus AMAM023150
Anopheles atroparvus AATE016802
Anopheles sinensis ASIC011279
Anopheles albimanus AALB015492
Anopheles farauti AFAF016749
Anopheles darlingi ADAC001890
Anopheles dirus ADIR009147
Aedes aegypti AAEL011746
Culex quinquefasciatus CPIJ006157
Polypedilum vanderplanki Pv.13307
Polypedilum nubifer Pn.16991
Belgica antarctica IU25 06941
Mayetiola destructor Mdes013769
Megaselia scalaris MESCA002084
Phlebotomus papatasi PPATMP010463
Drosophila simulans Dsim GD18581
Drosophila sechellia Dsec GM23771
Drosophila erecta Dere GG13653
Drosophila yakuba Dyak GE25915
Drosophila melanogaster skap
Drosophila suzukii DS10 00012061
Drosophila ananassae Dana GF17693
Drosophila willistoni Dwil GK11971
Drosophila mojavensis Dmoj GI10056
Drosophila grimshawi Dgri GH23962
Drosophila virilis Dvir GJ23792
Drosophila pseudoobscura FBgn0247705
Drosophila persimilis Dper GL21555
Glossina pallidipes GPAI005297
Glossina austeni GAUT030560
Glossina palpalis GPPI045912
Glossina fuscipes GFUI040610
Glossina morsitans GMOY007910
Glossina brevipalpis GBRI019412
Musca domestica 17006768
Lucilia cuprina FF38 12724
Bactrocera tryoni JHQJ01000657
Bactrocera dorsalis 11782581
Ceratitis capitata CCAP001629
Bactrocera cucurbitae 11871301
Heliconius melpomene HMEL003338
Danaus plexippus DPOGS208650 PA
Melitaea cinxia MCINX005648
Manduca sexta Msex2.09793
Bombyx mori BGIBMGA007783
Papilio glaucus pgl2617.7
Plutella xylostella Px009832
Solenopsis invicta SINV11458
Monomorium pharaonis 2170654450
Pogonomyrmex barbatus PB21114
Wasmannia auropunctata 23051074
Cardiocondyla obscurior Cobs 17243
Acromyrmex echinatior AECH18082
Atta cephalotes ACEP19434
Vollenhovia emeryi 19051878
Harpegnathos saltator HSAL22005
Cerapachys biroi EZA56733.1
Camponotus floridanus CFLO13358
Linepithema humile LH12065
Melipona quadrifasciata MQUAD 002c4c
Apis dorsata 51570862
Apis mellifera GB54423
Apis cerana ACSNU08751T0
Apis florea 3235939396
Bombus terrestris 15211144
Bombus impatiens Bimp 2.0 2
Eufriesea mexicana EMEXI 002d80
Megachile rotundata MROTU 002082
Dufourea novaeangliae DNOVA 002d4e
Lasioglossum albipes Lalb 05001
Polistes dominula 6306
Fopius arisanus 6705383
Athalia rosae AROS001887
Nasonia vitripennis Nasvi2EG005957
Copidosoma floridanum CFLO001710
Trichogramma pretiosum TPRE001588
Cyphon laevipennis
Cybister lateralimarginalis
Aspidytes wrasei
Noterus clavicornis
Hydroscapha redfordi
Cicindela hybrida
Micromalthus debilis
Tribolium castaneum TC013859
Bitoma cylindrica
Thanasimus formicarius
Rhyzobius pseudopulcher
Byturus ochraceus
Leptinotarsa decemlineata LDEC014816
Dendroctonus ponderosae YQE 07157
Anoplophora glabripennis AGLA020718
Agrilus planipennis APLA004846
Ocypus brunnipes
Lamprohiza splendidula
Hydrochara caraboides
Lepicerus sp
Cetonia aurata
Onthophagus taurus OTAU007559
Xylobiops basilaris
Meloe violaceus
Halyomorpha halys HHAL006679
Oncopeltus fasciatus OFAS015279
Cimex lectularius CLEC011963
Rhodnius prolixus RPRC002969
Acyrthosiphon pisum ACYPI009462
Mengenilla moldrzyki mesp 3144
Gerris buenoi GBUE004711
Homalodisca vitripennis HVIT014220
Diaphorina citri ref0040611
Frankliniella occidentalis FOCC007008
Zootermopsis nevadensis Znev 02332
Blattella germanica BGER022393
Pediculus humanus PHUM168040
Ladona fulva LFUL006472
Calopteryx splendens size342871
Ephemera danica EDAN012067
Diptera
Lepidoptera
Trichoptera
Coleoptera
Strepsiptera
Hymenoptera
Psocodea
Thysanoptera
Hemiptera
Blattodea

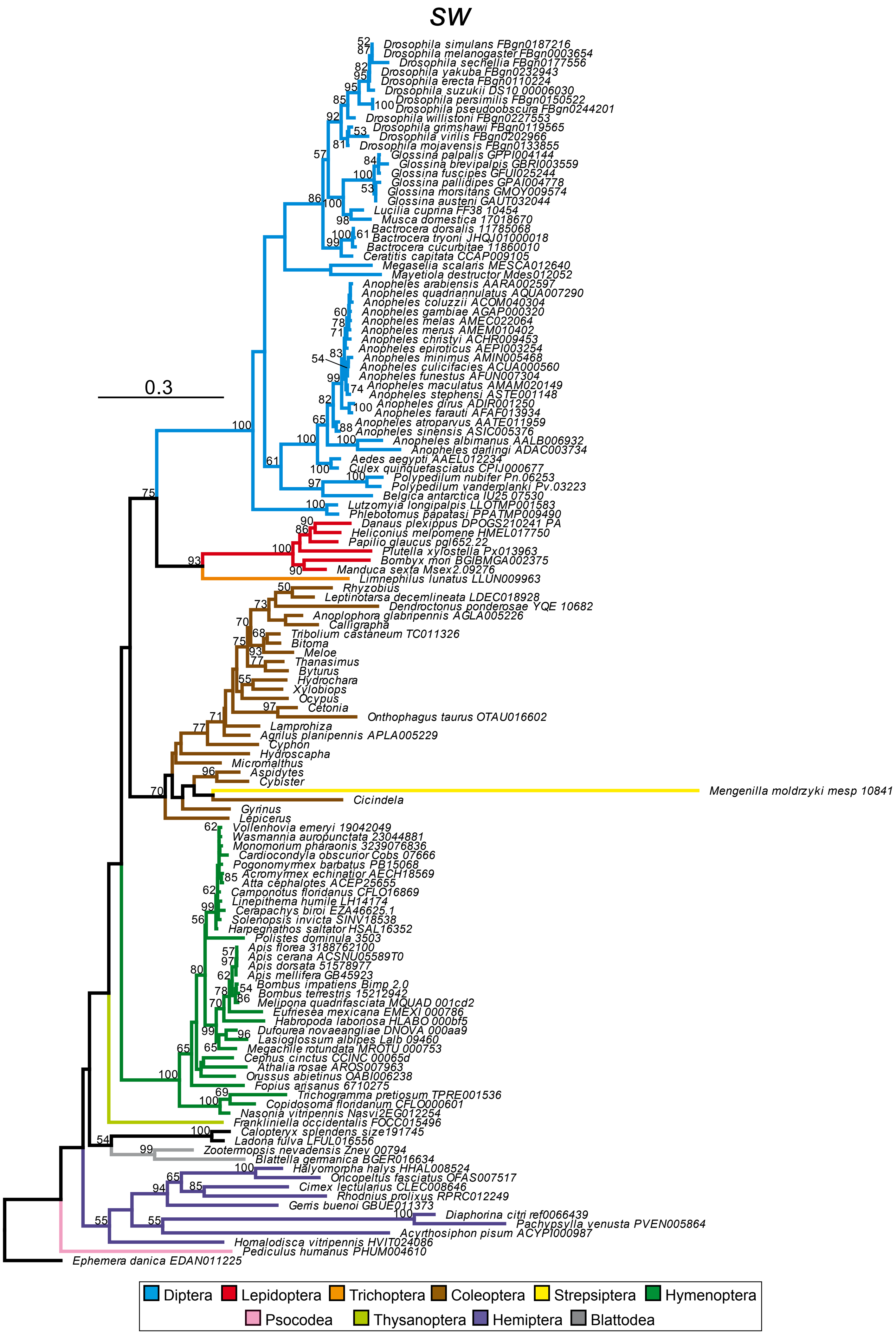

SW
0.3
Drosophila simulans FBgn0187216
Drosophila melanogaster FBgn0003654
Drosophila sechellia FBgn0177556
Drosophila yakuba FBgn0232943
Drosophila erecta FBgn0110224
Drosophila suzukii DS10 00006030
Drosophila persimilis FBgn0150522
Drosophila pseudoobscura FBgn0244201
Drosophila willistoni FBgn0227553
Drosophila grimshawi FBgn0119565
Drosophila virilis FBgn0202966
Drosophila mojavensis FBgn0133855
Glossina palpalis GPPI004144
Glossina brevipalpis GBRI003559
Glossina fuscipes GFUI025244
Glossina pallidipes GPAI004778
Glossina morsitans GMOY009574
Glossina austeni GAUT032044
Lucilia cuprina FF38 10454
Musca domestica 17018670
Bactrocera dorsalis 11785068
Bactrocera tryoni JHQJ01000018
Bactrocera cucurbitae 11860010
Ceratitis capitata CCAP009105
Megaselia scalaris MESCA012640
Mayetiola destructor Mdes012052
Anopheles arabiensis AARA002597
Anopheles quadriannulatus AQUA007290
Anopheles coluzzii ACOM040304
Anopheles gambiae AGAP000320
Anopheles melas AMEC022064
Anopheles merus AMEM010402
Anopheles christyi ACHR009453
Anopheles epiroticus AEPI003254
Anopheles minimus AMIN005468
Anopheles culicifacies ACUA000560
Anopheles funestus AFUN007304
Anopheles maculatus AMAM020149
Anopheles stephensi ASTE001148
Anopheles dirus ADIR001250
Anopheles farauti AFAF013934
Anopheles atroparvus AATE011959
Anopheles sinensis ASIC005376
Anopheles albimanus AALB006932
Anopheles darlingi ADAC003734
Aedes aegypti AAEL012234
Culex quinquefasciatus CPIJ000677
Polypedilum nubifer Pn.06253
Polypedilum vanderplanki Pv.03223
Belgica antarctica IU25 07530
Lutzomyia longipalpis LLOTMP001583
Phlebotomus papatasi PPATMP009490
Danaus plexippus DPOGS210241 PA
Heliconius melpomene HMEL017750
Papilio glaucus pgl652.22
Plutella xylostella Px013963
Bombyx mori BGIBMGA002375
Manduca sexta Msex2.09276
Limnephilus lunatus LLUN009963
Rhyzobius
Leptinotarsa decemlineata LDEC018928
Dendroctonus ponderosae YQE 10682
Anoplophora glabripennis AGLA005226
Calligrapha
Tribolium castaneum TC011326
Bitoma
Meloe
Thanasimus
Byturus
Hydrochara
Xylobiops
Ocypus
Cetonia
Onthophagus taurus OTAU016602
Lamprohiza
Agrilus planipennis APLA005229
Cyphon
Hydroscapha
Micromalthus
Aspidytes
Cybister
Mengenilla moldrzyki mesp 10841
Cicindela
Gyrinus
Lepicerus
Vollenhovia emeryi 19042049
Wasmannia auropunctata 23044881
Monomorium pharaonis 3239076836
Cardiocondyla obscurior Cobs 07666
Pogonomyrmex barbatus PB15068
Acromyrmex echinatior AECH18569
Atta cephalotes ACEP25655
Camponotus floridanus CFLO16869
Linepithema humile LH14174
Cerapachys biroi EZA46625.1
Solenopsis invicta SINV18538
Harpegnathos saltator HSAL16352
Polistes dominula 3503
Apis florea 3188762100
Apis cerana ACSNU05589T0
Apis dorsata 51578977
Apis mellifera GB45923
Bombus impatiens Bimp 2.0
Bombus terrestris 15212942
Melipona quadrifasciata MQUAD 001cd2
Eufriesea mexicana EMEXI 000786
Habropoda laboriosa HLABO 000bf5
Dufourea novaeangliae DNOVA 000aa9
Lasioglossum albipes Lalb 09460
Megachile rotundata MROTU 000753
Cephus cinctus CCINC 00065d
Athalia rosae AROS007963
Orussus abietinus OABI006238
Fopius arisanus 6710275
Trichogramma pretiosum TPRE001536
Copidosoma floridanum CFLO000601
Nasonia vitripennis Nasvi2EG012254
Frankliniella occidentalis FOCC015496
Calopteryx splendens size191745
Ladona fulva LFUL016556
Zootermopsis nevadensis Znev 00794
Blattella germanica BGER016634
Halyomorpha halys HHAL008524
Oncopeltus fasciatus OFAS007517
Cimex lectularius CLEC008646
Rhodnius prolixus RPRC012249
Gerris buenoi GBUE011373
Diaphorina citri ref0066439
Pachypsylla venusta PVEN005864
Acyrthosiphon pisum ACYPI000987
Homalodisca vitripennis HVIT024086
Pediculus humanus PHUM004610
Ephemera danica EDAN011225
Diptera
Lepidoptera
Trichoptera
Coleoptera
Strepsiptera
Hymenoptera
Psocodea
Thysanoptera
Hemiptera
Blattodea

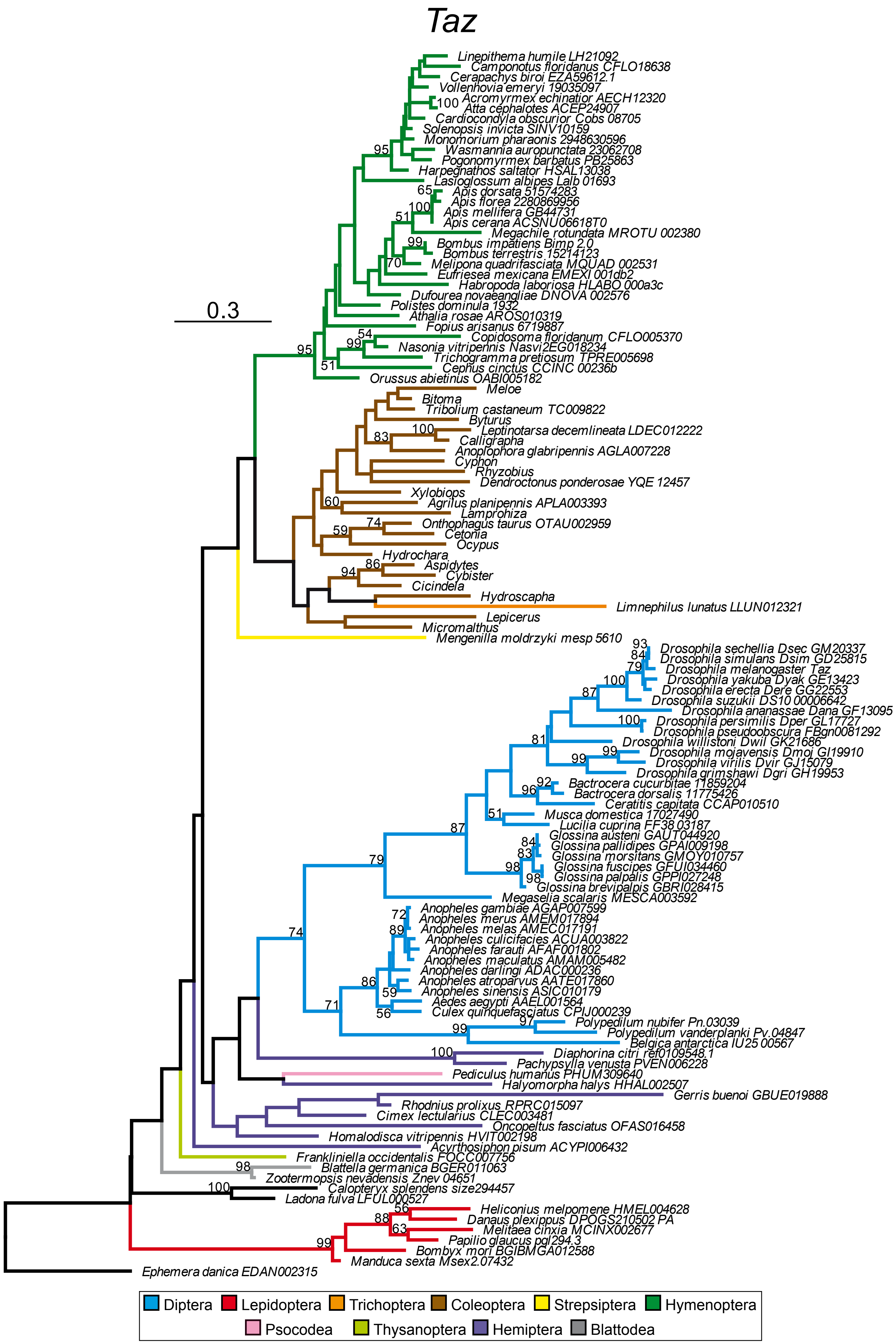
Taz
0.3
Linepithema humile LH21092
Camponotus floridanus CFLO18638
Cerapachys biroi EZA59612.1
Vollenhovia emeryi 19035097
Acromyrmex echinatior AECH12320
Atta cephalotes ACEP24907
Cardiocondyla obscurior Cobs 08705
Solenopsis invicta SINV10159
Monomorium pharaonis 2948630596
Wasmannia auropunctata 23062708
Pogonomyrmex barbatus PB25863
Harpegnathos saltator HSAL13038
Lasioglossum albipes Lalb 01693
Apis dorsata 51574283
Apis florea 2280869956
Apis mellifera GB44731
Apis cerana ACSNU06618T0
Megachile rotundata MROTU 002380
Bombus impatiens Bimp 2.0
Bombus terrestris 15214123
Melipona quadrifasciata MQUAD 002531
Eufriesea mexicana EMEXI 001db2
Habropoda laboriosa HLABO 000a3c
Dufourea novaeangliae DNOVA 002576
Polistes dominula 1932
Athalia rosae AROS010319
Fopius arisanus 6719887
Copidosoma floridanum CFLO005370
Nasonia vitripennis Nasvi2EG018234
Trichogramma pretiosum TPRE005698
Cephus cinctus CCINC 00236b
Orussus abietinus OABI005182
Meloe
Bitoma
Tribolium castaneum TC009822
Byturus
Leptinotarsa decemlineata LDEC012222
Calligrapha
Anoplophora glabripennis AGLA007228
Cyphon
Rhyzobius
Dendroctonus ponderosae YQE 12457
Xylobiops
Agrilus planipennis APLA003393
Lamprohiza
Onthophagus taurus OTAU002959
Cetonia
Ocypus
Hydrochara
Aspidytes
Cybister
Cicindela
Hydroscapha
Limnephilus lunatus LLUN012321
Lepicerus
Micromalthus
Mengenilla moldrzyki mesp 5610
Drosophila sechellia Dsec GM20337
Drosophila simulans Dsim GD25815
Drosophila melanogaster Taz
Drosophila yakuba Dyak GE13423
Drosophila erecta Dere GG22553
Drosophila suzukii DS10 00006642
Drosophila ananassae Dana GF13095
Drosophila persimilis Dper GL17727
Drosophila pseudoobscura FBgn0081292
Drosophila willistoni Dwil GK21686
Drosophila mojavensis Dmoj GI19910
Drosophila virilis Dvir GJ15079
Drosophila grimshawi Dgri GH19953
Bactrocera cucurbitae 11859204
Bactrocera dorsalis 11775426
Ceratitis capitata CCAP010510
Musca domestica 17027490
Lucilia cuprina FF38 03187
Glossina austeni GAUT044920
Glossina pallidipes GPAI009198
Glossina morsitans GMOY010757
Glossina fuscipes GFUI034460
Glossina palpalis GPPI027248
Glossina brevipalpis GBRI028415
Megaselia scalaris MESCA003592
Anopheles gambiae AGAP007599
Anopheles merus AMEM017894
Anopheles melas AMEC017191
Anopheles culicifacies ACUA003822
Anopheles farauti AFAF001802
Anopheles maculatus AMAM005482
Anopheles darlingi ADAC000236
Anopheles atroparvus AATE017860
Anopheles sinensis ASIC010179
Aedes aegypti AAEL001564
Culex quinquefasciatus CPIJ000239
Polypedilum nubifer Pn.03039
Polypedilum vanderplanki Pv.04847
Belgica antarctica IU25 00567
Diaphorina citri ref0109548.1
Pachypsylla venusta PVEN006228
Pediculus humanus PHUM309640
Halyomorpha halys HHAL002507
Gerris buenoi GBUE019888
Rhodnius prolixus RPRC015097
Cimex lectularius CLEC003481
Oncopeltus fasciatus OFAS016458
Homalodisca vitripennis HVIT002198
Acyrthosiphon pisum ACYPI006432
Frankliniella occidentalis FOCC007756
Blattella germanica BGER011063
Zootermopsis nevadensis Znev 04651
Calopteryx splendens size294457
Ladona fulva LFUL000527
Heliconius melpomene HMEL004628
Danaus plexippus DPOGS210502 PA
Melitaea cinxia MCINX002677
Papilio glaucus pgl294.3
Bombyx mori BGIBMGA012588
Manduca sexta Msex2.07432
Ephemera danica EDAN002315
Diptera
Lepidoptera
Trichoptera
Coleoptera
Strepsiptera
Hymenoptera
Psocodea
Thysanoptera
Hemiptera
Blattodea

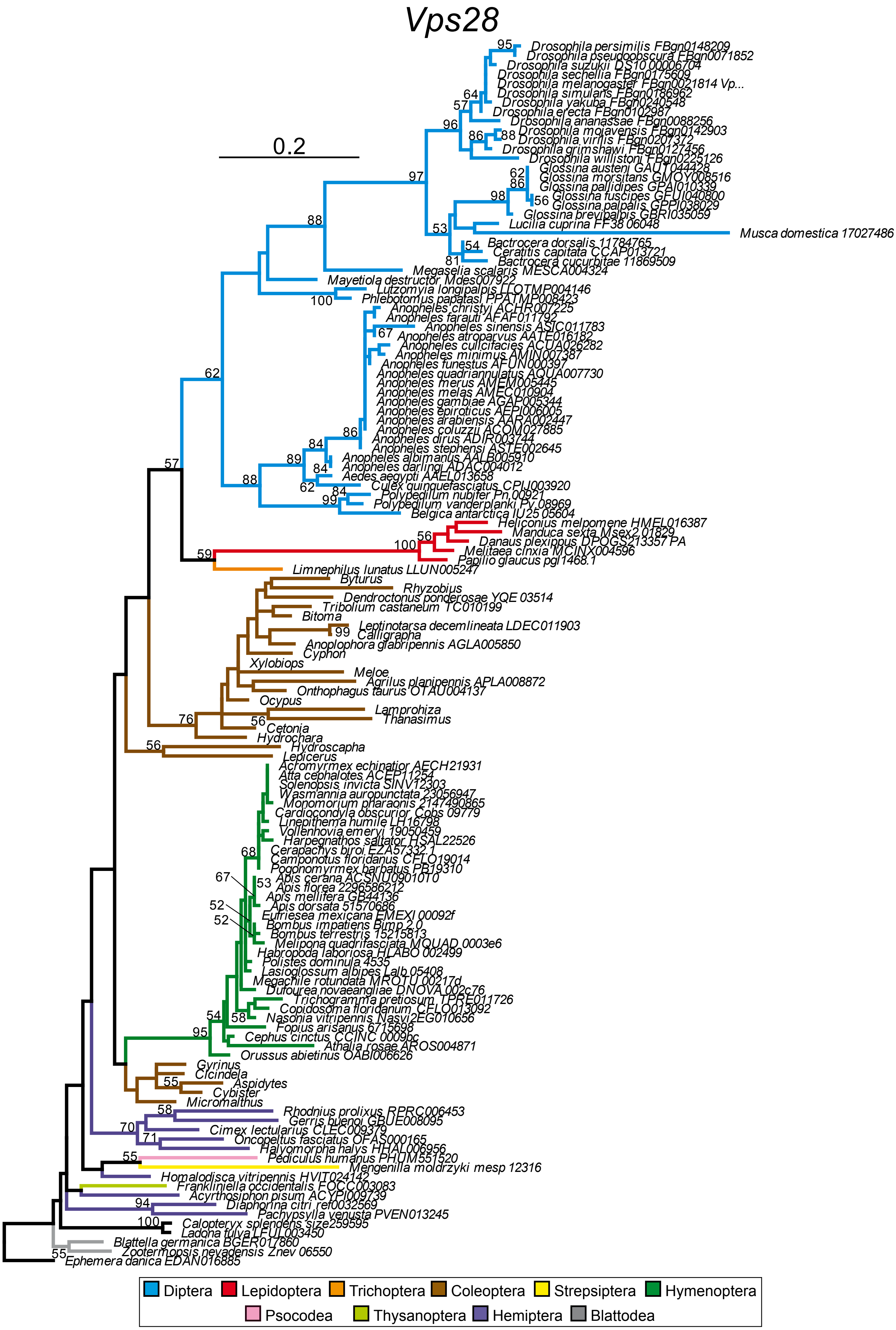

Vps28
0.2
Drosophila persimilis FBgn0148209
Drosophila pseudoobscura FBgn0071852
Drosophila suzukii DS10 00006704
Drosophila sechellia FBgn0175609
Drosophila melanogaster FBgn0021814 Vp...
Drosophila simulans FBgn0186962
Drosophila yakuba FBgn0240548
Drosophila erecta FBgn0102987
Drosophila ananassae FBgn0088256
Drosophila mojavensis FBgn0142903
Drosophila virilis FBgn0207372
Drosophila grimshawi FBgn0127456
Drosophila willistoni FBgn0225126
Glossina austeni GAUT044428
Glossina morsitans GMOY008516
Glossina pallidipes GPAI010339
Glossina fuscipes GFUI040800
Glossina palpalis GPPI038029
Glossina brevipalpis GBRI035059
Lucilia cuprina FF38 06048
Musca domestica 17027486
Bactrocera dorsalis 11784765
Ceratitis capitata CCAP013721
Bactrocera cucurbitae 11869509
Megaselia scalaris MESCA004324
Mayetiola destructor Mdes007922
Lutzomyia longipalpis LLOTMP004146
Phlebotomus papatasi PPATMP008423
Anopheles christyi ACHR007225
Anopheles farauti AFAF011792
Anopheles sinensis ASIC011783
Anopheles atroparvus AATE016182
Anopheles culicifacies ACUA026282
Anopheles minimus AMIN007387
Anopheles funestus AFUN000397
Anopheles quadriannulatus AQUA007730
Anopheles merus AMEM005445
Anopheles melas AMEC010904
Anopheles gambiae AGAP005344
Anopheles epiroticus AEPI006005
Anopheles arabiensis AARA002447
Anopheles coluzzii ACOM027885
Anopheles dirus ADIR003744
Anopheles stephensi ASTE002645
Anopheles albimanus AALB005910
Anopheles darlingi ADAC004012
Aedes aegypti AAEL013658
Culex quinquefasciatus CPIJ003920
Polypedilum nubifer Pn.00921
Polypedilum vanderplanki Pv.08969
Belgica antarctica IU25 05604
Heliconius melpomene HMEL016387
Manduca sexta Msex2.01829
Danaus plexippus DPOGS213357 PA
Melitaea cinxia MCINX004596
Papilio glaucus pgl1468.1
Limnephilus lunatus LLUN005247
Byturus
Rhyzobius
Dendroctonus ponderosae YQE 03514
Tribolium castaneum TC010199
Bitoma
Leptinotarsa decemlineata LDEC011903
Calligrapha
Anoplophora glabripennis AGLA005850
Cyphon
Xylobiops
Meloe
Agrilus planipennis APLA008872
Onthophagus taurus OTAU004137
Ocypus
Lamprohiza
Thanasimus
Cetonia
Hydrochara
Hydroscapha
Lepicerus
Acromyrmex echinatior AECH21931
Atta cephalotes ACEP11254
Solenopsis invicta SINV12303
Wasmannia auropunctata 23056947
Monomorium pharaonis 2147490865
Cardiocondyla obscurior Cobs 09779
Linepithema humile LH16798
Vollenhovia emeryi 19050459
Harpegnathos saltator HSAL22526
Cerapachys biroi EZA57332.1
Camponotus floridanus CFLO19014
Pogonomyrmex barbatus PB19310
Apis cerana ACSNU09010T0
Apis florea 2296586212
Apis mellifera GB44136
Apis dorsata 51570686
Eufriesea mexicana EMEXI 00092f
Bombus impatiens Bimp 2.0
Bombus terrestris 15215873
Melipona quadrifasciata MQUAD 0003e6
Habropoda laboriosa HLABO 002499
Polistes dominula 4535
Lasioglossum albipes Lalb 05408
Megachile rotundata MROTU 00217d
Dufourea novaeangliae DNOVA 002c76
Trichogramma pretiosum TPRE011726
Copidosoma floridanum CFLO013092
Nasonia vitripennis Nasvi2EG010656
Fopius arisanus 6715698
Cephus cinctus CCINC 0009bc
Athalia rosae AROS004871
Orussus abietinus OABI006626
Gyrinus
Cicindela
Aspidytes
Cybister
Micromalthus
Rhodnius prolixus RPRC006453
Gerris buenoi GBUE008095
Cimex lectularius CLEC009379
Oncopeltus fasciatus OFAS000165
Halyomorpha halys HHAL006956
Pediculus humanus PHUM551520
Mengenilla moldrzyki mesp 12316
Homalodisca vitripennis HVIT024142
Frankliniella occidentalis FOCC003083
Acyrthosiphon pisum ACYPI009739
Diaphorina citri ref0032569
Pachypsylla venusta PVEN013245
Calopteryx splendens size259595
Ladona fulva LFUL003450
Blattella germanica BGER017860
Zootermopsis nevadensis Znev 06550
Ephemera danica EDAN016885
Diptera
Lepidoptera
Trichoptera
Coleoptera
Strepsiptera
Hymenoptera
Psocodea
Thysanoptera
Hemiptera
Blattodea

File S2. Bayesian inference trees based on the amino acid alignments of different sperm individualization proteins in insects.

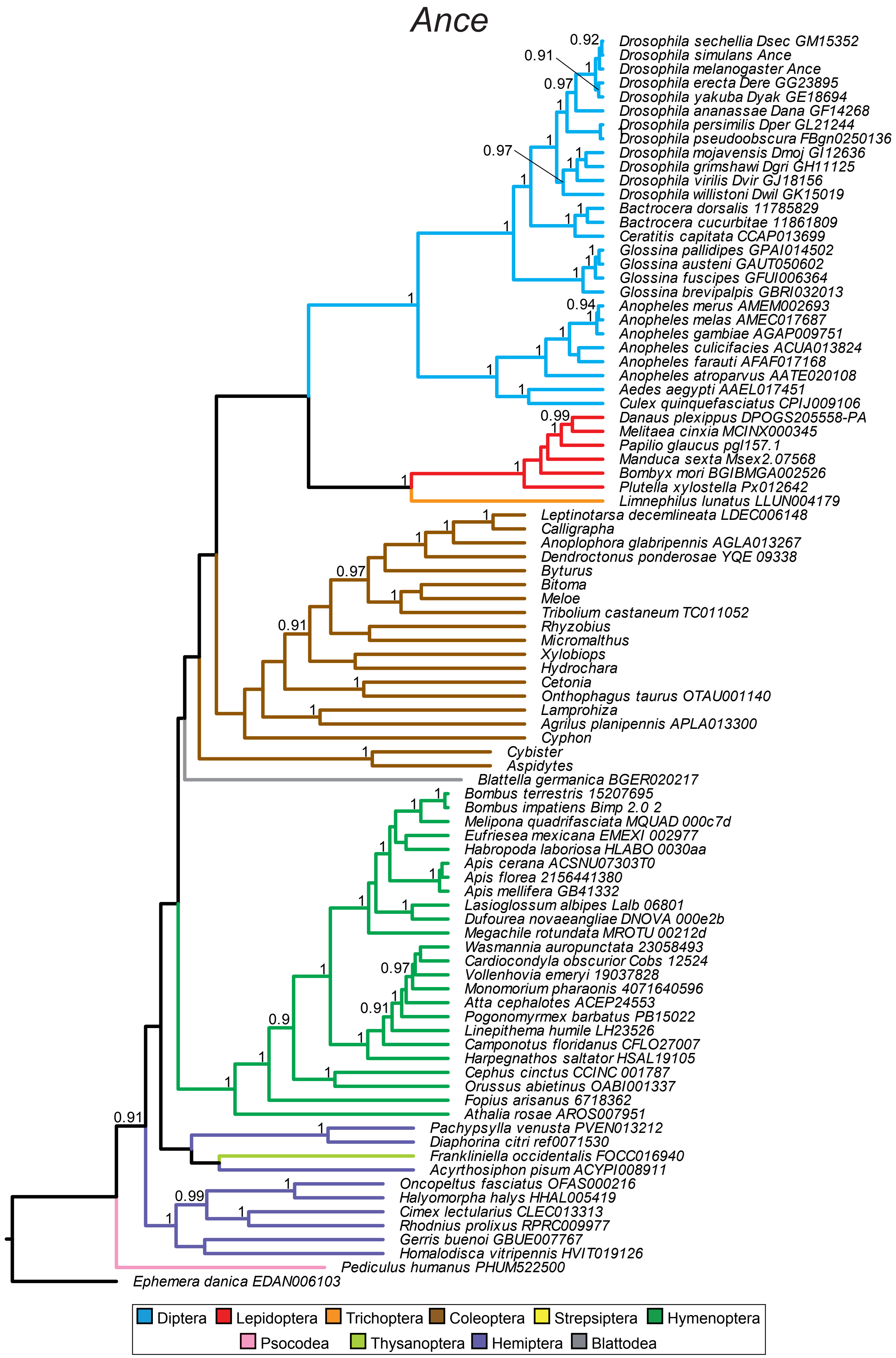
Ance
Drosophila sechellia Dsec GM15352
Drosophila simulans Ance
Drosophila melanogaster Ance
Drosophila erecta Dere GG23895
Drosophila yakuba Dyak GE18694
Drosophila ananassae Dana GF14268
Drosophila persimilis Dper GL21244
Drosophila pseudoobscura FBgn0250136
Drosophila mojavensis Dmoj GI12636
Drosophila grimshawi Dgri GH11125
Drosophila virilis Dvir GJ18156
Drosophila willistoni Dwil GK15019
Bactrocera dorsalis 11785829
Bactrocera cucurbitae 11861809
Ceratitis capitata CCAP013699
Glossina pallidipes GPAI014502
Glossina austeni GAUT050602
Glossina fuscipes GFUI006364
Glossina brevipalpis GBRI032013
Anopheles merus AMEM002693
Anopheles melas AMEC017687
Anopheles gambiae AGAP009751
Anopheles culicifacies ACUA013824
Anopheles farauti AFAF017168
Anopheles atroparvus AATE020108
Aedes aegypti AAEL017451
Culex quinquefasciatus CPIJ009106
Danaus plexippus DPOGS205558-PA
Melitaea cinxia MCINX000345
Papilio glaucus pgl157.1
Manduca sexta Msex2.07568
Bombyx mori BGIBMGA002526
Plutella xylostella Px012642
Limnephilus lunatus LLUN004179
Leptinotarsa decemlineata LDEC006148
Calligrapha
Anoplophora glabripennis AGLA013267
Dendroctonus ponderosae YQE 09338
Byturus
Bitoma
Meloe
Tribolium castaneum TC011052
Rhyzobius
Micromalthus
Xylobiops
Hydrochara
Cetonia
Onthophagus taurus OTAU001140
Lamprohiza
Agrilus planipennis APLA013300
Cyphon
Cybister
Aspidytes
Blattella germanica BGER020217
Bombus terrestris 15207695
Bombus impatiens Bimp 2.0 2
Melipona quadrifasciata MQUAD 000c7d
Eufriesea mexicana EMEXI 002977
Habropoda laboriosa HLABO 0030aa
Apis cerana ACSNU07303T0
Apis florea 2156441380
Apis mellifera GB41332
Lasioglossum albipes Lalb 06801
Dufourea novaeangliae DNOVA 000e2b
Megachile rotundata MROTU 00212d
Wasmannia auropunctata 23058493
Cardiocondyla obscurior Cobs 12524
Vollenhovia emeryi 19037828
Monomorium pharaonis 4071640596
Atta cephalotes ACEP24553
Pogonomyrmex barbatus PB15022
Linepithema humile LH23526
Camponotus floridanus CFLO27007
Harpegnathos saltator HSAL19105
Cephus cinctus CCINC 001787
Orussus abietinus OABI001337
Fopius arisanus 6718362
Athalia rosae AROS007951
Pachypsylla venusta PVEN013212
Diaphorina citri ref0071530
Frankliniella occidentalis FOCC016940
Acyrthosiphon pisum ACYPI008911
Oncopeltus fasciatus OFAS000216
Halyomorpha halys HHAL005419
Cimex lectularius CLEC013313
Rhodnius prolixus RPRC009977
Gerris buenoi GBUE007767
Homalodisca vitripennis HVIT019126
Pediculus humanus PHUM522500
Ephemera danica EDAN006103
Diptera
Lepidoptera
Trichoptera
Coleoptera
Strepsiptera
Hymenoptera
Psocodea
Thysanoptera
Hemiptera
Blattodea

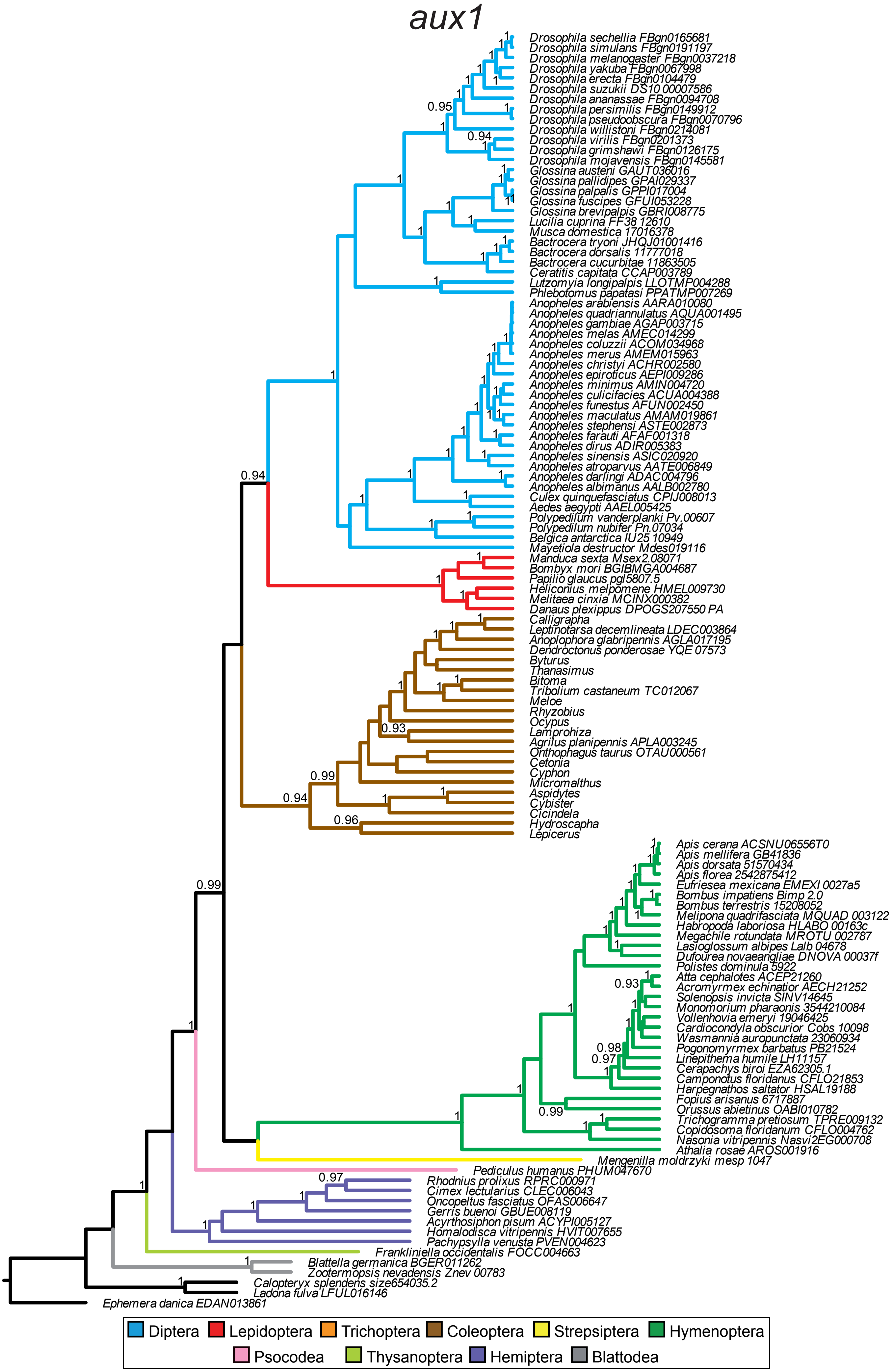
aux1
Drosophila sechellia FBgn0165681
Drosophila simulans FBgn0191197
Drosophila melanogaster FBgn0037218
Drosophila yakuba FBgn0067998
Drosophila erecta FBgn0104479
Drosophila suzukii DS10 00007586
Drosophila ananassae FBgn0094708
Drosophila persimilis FBgn0149912
Drosophila pseudoobscura FBgn0070796
Drosophila willistoni FBgn0214081
Drosophila virilis FBgn0201373
Drosophila grimshawi FBgn0126175
Drosophila mojavensis FBgn0145581
Glossina austeni GAUT036016
Glossina pallidipes GPAI029337
Glossina palpalis GPPI017004
Glossina fuscipes GFUI053228
Glossina brevipalpis GBRI008775
Lucilia cuprina FF38 12610
Musca domestica 17016378
Bactrocera tryoni JHQJ01001416
Bactrocera dorsalis 11777018
Bactrocera cucurbitae 11863505
Ceratitis capitata CCAP003789
Lutzomyia longipalpis LLOTMP004288
Phlebotomus papatasi PPATMP007269
Anopheles arabiensis AARA010080
Anopheles quadriannulatus AQUA001495
Anopheles gambiae AGAP003715
Anopheles melas AMEC014299
Anopheles coluzzii ACOM034968
Anopheles merus AMEM015963
Anopheles christyi ACHR002580
Anopheles epiroticus AEPI009286
Anopheles minimus AMIN004720
Anopheles culicifacies ACUA004388
Anopheles funestus AFUN002450
Anopheles maculatus AMAM019861
Anopheles stephensi ASTE002873
Anopheles farauti AFAF001318
Anopheles dirus ADIR005383
Anopheles sinensis ASIC020920
Anopheles atroparvus AATE006849
Anopheles darlingi ADAC004796
Anopheles albimanus AALB002780
Culex quinquefasciatus CPIJ008013
Aedes aegypti AAEL005425
Polypedilum vanderplanki Pv.00607
Polypedilum nubifer Pn.07034
Belgica antarctica IU25 10949
Mayetiola destructor Mdes019116
Manduca sexta Msex2.08071
Bombyx mori BGIBMGA004687
Papilio glaucus pgl5807.5
Heliconius melpomene HMEL009730
Melitaea cinxia MCINX000382
Danaus plexippus DPOGS207550 PA
Calligrapha
Leptinotarsa decemlineata LDEC003864
Anoplophora glabripennis AGLA017195
Dendroctonus ponderosae YQE 07573
Byturus
Thanasimus
Bitoma
Tribolium castaneum TC012067
Meloe
Rhyzobius
Ocypus
Lamprohiza
Agrilus planipennis APLA003245
Onthophagus taurus OTAU000561
Cetonia
Cyphon
Micromalthus
Aspidytes
Cybister
Cicindela
Hydroscapha
Lepicerus
Apis cerana ACSNU06556T0
Apis mellifera GB41836
Apis dorsata 51570434
Apis florea 2542875412
Eufriesea mexicana EMEXI 0027a5
Bombus impatiens Bimp 2.0
Bombus terrestris 15208052
Melipona quadrifasciata MQUAD 003122
Habropoda laboriosa HLABO 00163c
Megachile rotundata MROTU 002787
Lasioglossum albipes Lalb 04678
Dufourea novaeangliae DNOVA 00037f
Polistes dominula 5922
Atta cephalotes ACEP21260
Acromyrmex echinatior AECH21252
Solenopsis invicta SINV14645
Monomorium pharaonis 3544210084
Vollenhovia emeryi 19046425
Cardiocondyla obscurior Cobs 10098
Wasmannia auropunctata 23060934
Pogonomyrmex barbatus PB21524
Linepithema humile LH11157
Cerapachys biroi EZA62305.1
Camponotus floridanus CFLO21853
Harpegnathos saltator HSAL19188
Fopius arisanus 6717887
Orussus abietinus OABI010782
Trichogramma pretiosum TPRE009132
Copidosoma floridanum CFLO004762
Nasonia vitripennis Nasvi2EG000708
Athalia rosae AROS001916
Mengenilla moldrzyki mesp 1047
Pediculus humanus PHUM047670
Rhodnius prolixus RPRC000971
Cimex lectularius CLEC006043
Oncopeltus fasciatus OFAS006647
Gerris buenoi GBUE008119
Acyrthosiphon pisum ACYPI005127
Homalodisca vitripennis HVIT007655
Pachypsylla venusta PVEN004623
Frankliniella occidentalis FOCC004663
Blattella germanica BGER011262
Zootermopsis nevadensis Znev 00783
Calopteryx splendens size654035.2
Ladona fulva LFUL016146
Ephemera danica EDAN013861
Diptera
Lepidoptera
Trichoptera
Coleoptera
Strepsiptera
Hymenoptera
Psocodea
Thysanoptera
Hemiptera
Blattodea

blanks

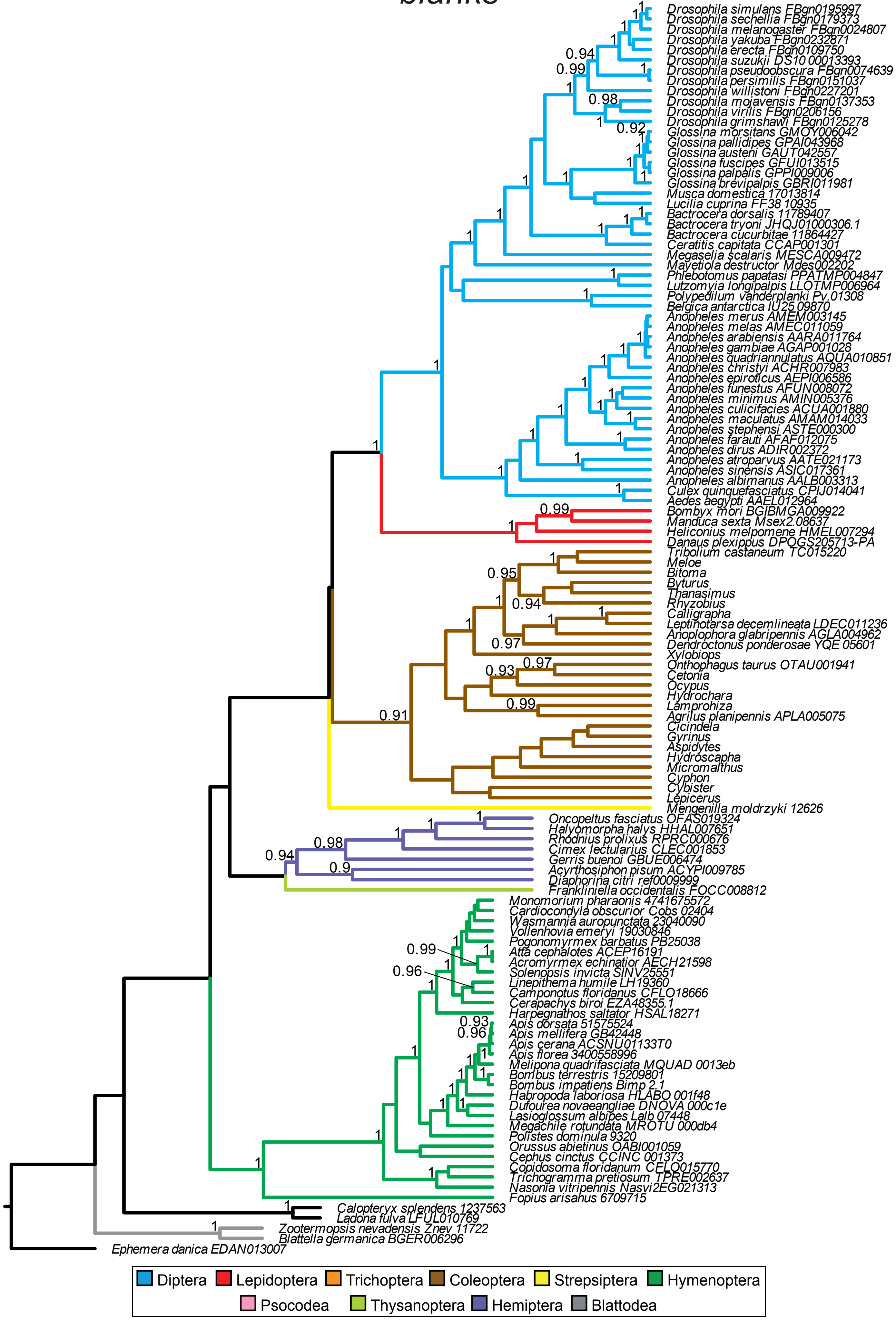
Drosophila simulans FBgn0195997
Drosophila sechellia FBgn0179373
Drosophila melanogaster FBgn0024807
Drosophila yakuba FBgn0232871
Drosophila erecta FBgn0109750
Drosophila suzukii DS10 00013393
Drosophila pseudoobscura FBgn0074639
Drosophila persimilis FBgn0151037
Drosophila willistoni FBgn0227201
Drosophila mojavensis FBgn0137353
Drosophila virilis FBgn0206156
Drosophila grimshawi FBgn0125278
Glossina morsitans GMOY006042
Glossina pallidipes GPAI043968
Glossina austeni GAUT042557
Glossina fuscipes GFUI013515
Glossina palpalis GPPI009006
Glossina brevipalpis GBRI011981
Musca domestica 17013814
Lucilia cuprina FF38 10935
Bactrocera dorsalis 11789407
Bactrocera tryoni JHQJ01000306.1
Bactrocera cucurbitae 11864427
Ceratitis capitata CCAP001301
Megaselia scalaris MESCA009472
Mayetiola destructor Mdes002202
Phlebotomus papatasi PPATMP004847
Lutzomyia longipalpis LLOTMP006964
Polypedilum vanderplanki Pv.01308
Belgica antarctica IU25 09870
Anopheles merus AMEM003145
Anopheles melas AMEC011059
Anopheles arabiensis AARA011764
Anopheles gambiae AGAP001028
Anopheles quadriannulatus AQUA010851
Anopheles christyi ACHR007983
Anopheles epiroticus AEPI006586
Anopheles funestus AFUN008072
Anopheles minimus AMIN005376
Anopheles culicifacies ACUA001880
Anopheles maculatus AMAM014033
Anopheles stephensi ASTE000300
Anopheles farauti AFAF012075
Anopheles dirus ADIR002372
Anopheles atroparvus AATE021173
Anopheles sinensis ASIC017361
Anopheles albimanus AALB003313
Culex quinquefasciatus CPIJ014041
Aedes aegypti AAEL012964
Bombyx mori BGIBMGA009922
Manduca sexta Msex2.08637
Heliconius melpomene HMEL007294
Danaus plexippus DPOGS205713-PA
Tribolium castaneum TC015220
Meloe
Bitoma
Byturus
Thanasimus
Rhyzobius
Calligrapha
Leptinotarsa decemlineata LDEC011236
Anoplophora glabripennis AGLA004962
Dendroctonus ponderosae YQE 05601
Xylobiops
Onthophagus taurus OTAU001941
Cetonia
Ocypus
Hydrochara
Lamprohiza
Agrilus planipennis APLA005075
Cicindela
Gyrinus
Aspidytes
Hydroscapha
Micromalthus
Cyphon
Cybister
Lepicerus
Mengenilla moldrzyki 12626
Oncopeltus fasciatus OFAS019324
Halyomorpha halys HHAL007651
Rhodnius prolixus RPRC000676
Cimex lectularius CLEC001853
Gerris buenoi GBUE006474
Acyrthosiphon pisum ACYPI009785
Diaphorina citri ref0009999
Frankliniella occidentalis FOCC008812
Monomorium pharaonis 4741675572
Cardiocondyla obscurior Cobs 02404
Wasmannia auropunctata 23040090
Vollenhovia emeryi 19030846
Pogonomyrmex barbatus PB25038
Atta cephalotes ACEP16191
Acromyrmex echinatior AECH21598
Solenopsis invicta SINV25551
Linepithema humile LH19360
Camponotus floridanus CFLO18666
Cerapachys biroi EZA48355.1
Harpegnathos saltator HSAL18271
Apis dorsata 51575524
Apis mellifera GB42448
Apis cerana ACSNU01133T0
Apis florea 3400558996
Melipona quadrifasciata MQUAD 0013eb
Bombus terrestris 15209801
Bombus impatiens Bimp 2.1
Habropoda laboriosa HLABO 001f48
Dufourea novaeangliae DNOVA 000c1e
Lasioglossum albipes Lalb 07448
Megachile rotundata MROTU 000db4
Polistes dominula 9320
Orussus abietinus OABI001059
Cephus cinctus CCINC 001373
Copidosoma floridanum CFLO015770
Trichogramma pretiosum TPRE002637
Nasonia vitripennis Nasvi2EG021313
Fopius arisanus 6709715
Calopteryx splendens 1237563
Ladona fulva LFUL010769
Zootermopsis nevadensis Znev 11722
Blattella germanica BGER006296
Ephemera danica EDAN013007
Diptera
Lepidoptera
Trichoptera
Coleoptera
Strepsiptera
Hymenoptera
Psocodea
Thysanoptera
Hemiptera
Blattodea

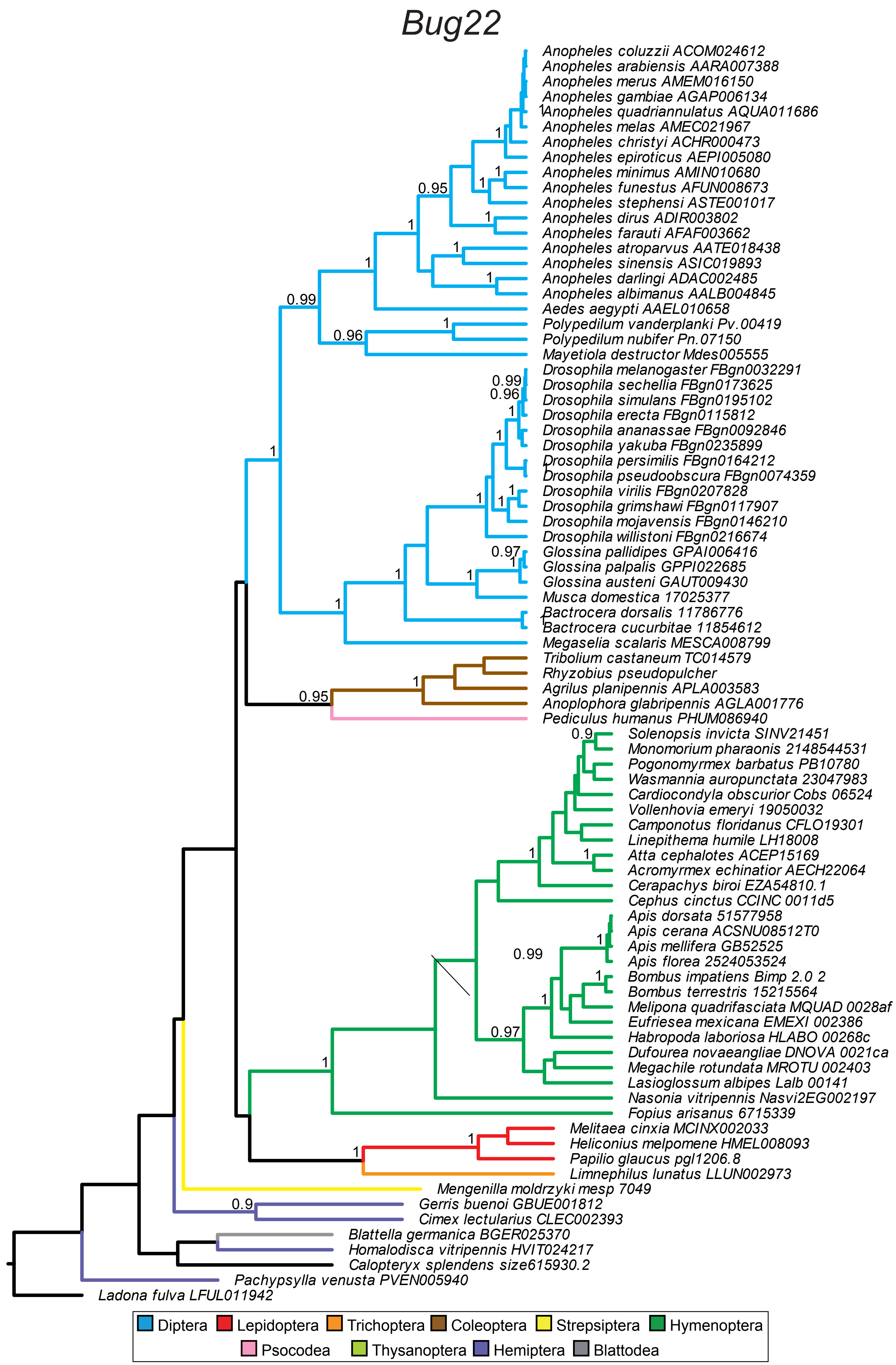
Bug22
Anopheles coluzzii ACOM024612
Anopheles arabiensis AARA007388
Anopheles merus AMEM016150
Anopheles gambiae AGAP006134
Anopheles quadriannulatus AQUA011686
Anopheles melas AMEC021967
Anopheles christyi ACHR000473
Anopheles epiroticus AEPI005080
Anopheles minimus AMIN010680
Anopheles funestus AFUN008673
Anopheles stephensi ASTE001017
Anopheles dirus ADIR003802
Anopheles farauti AFAF003662
Anopheles atroparvus AATE018438
Anopheles sinensis ASIC019893
Anopheles darlingi ADAC002485
Anopheles albimanus AALB004845
Aedes aegypti AAEL010658
Polypedilum vanderplanki Pv.00419
Polypedilum nubifer Pn.07150
Mayetiola destructor Mdes005555
Drosophila melanogaster FBgn0032291
Drosophila sechellia FBgn0173625
Drosophila simulans FBgn0195102
Drosophila erecta FBgn0115812
Drosophila ananassae FBgn0092846
Drosophila yakuba FBgn0235899
Drosophila persimilis FBgn0164212
Drosophila pseudoobscura FBgn0074359
Drosophila virilis FBgn0207828
Drosophila grimshawi FBgn0117907
Drosophila mojavensis FBgn0146210
Drosophila willistoni FBgn0216674
Glossina pallidipes GPAI006416
Glossina palpalis GPPI022685
Glossina austeni GAUT009430
Musca domestica 17025377
Bactrocera dorsalis 11786776
Bactrocera cucurbitae 11854612
Megaselia scalaris MESCA008799
Tribolium castaneum TC014579
Rhyzobius pseudopulcher
Agrilus planipennis APLA003583
Anoplophora glabripennis AGLA001776
Pediculus humanus PHUM086940
Solenopsis invicta SINV21451
Monomorium pharaonis 2148544531
Pogonomyrmex barbatus PB10780
Wasmannia auropunctata 23047983
Cardiocondyla obscurior Cobs 06524
Vollenhovia emeryi 19050032
Camponotus floridanus CFLO19301
Linepithema humile LH18008
Atta cephalotes ACEP15169
Acromyrmex echinatior AECH22064
Cerapachys biroi EZA54810.1
Cephus cinctus CCINC 0011d5
Apis dorsata 51577958
Apis cerana ACSNU08512T0
Apis mellifera GB52525
Apis florea 2524053524
Bombus impatiens Bimp 2.0 2
Bombus terrestris 15215564
Melipona quadrifasciata MQUAD 0028af
Eufriesea mexicana EMEXI 002386
Habropoda laboriosa HLABO 00268c
Dufourea novaeangliae DNOVA 0021ca
Megachile rotundata MROTU 002403
Lasioglossum albipes Lalb 00141
Nasonia vitripennis Nasvi2EG002197
Fopius arisanus 6715339
Melitaea cinxia MCINX002033
Heliconius melpomene HMEL008093
Papilio glaucus pgl1206.8
Limnephilus lunatus LLUN002973
Mengenilla moldrzyki mesp 7049
Gerris buenoi GBUE001812
Cimex lectularius CLEC002393
Blattella germanica BGER025370
Homalodisca vitripennis HVIT024217
Calopteryx splendens size615930.2
Pachypsylla venusta PVEN005940
Ladona fulva LFUL011942
0.95
0.99
0.96
0.99
0.96
0.97
0.95
0.9
0.99
0.97
0.9
Diptera
Lepidoptera
Trichoptera
Coleoptera
Strepsiptera
Hymenoptera
Psocodea
Thysanoptera
Hemiptera
Blattodea

CdsA

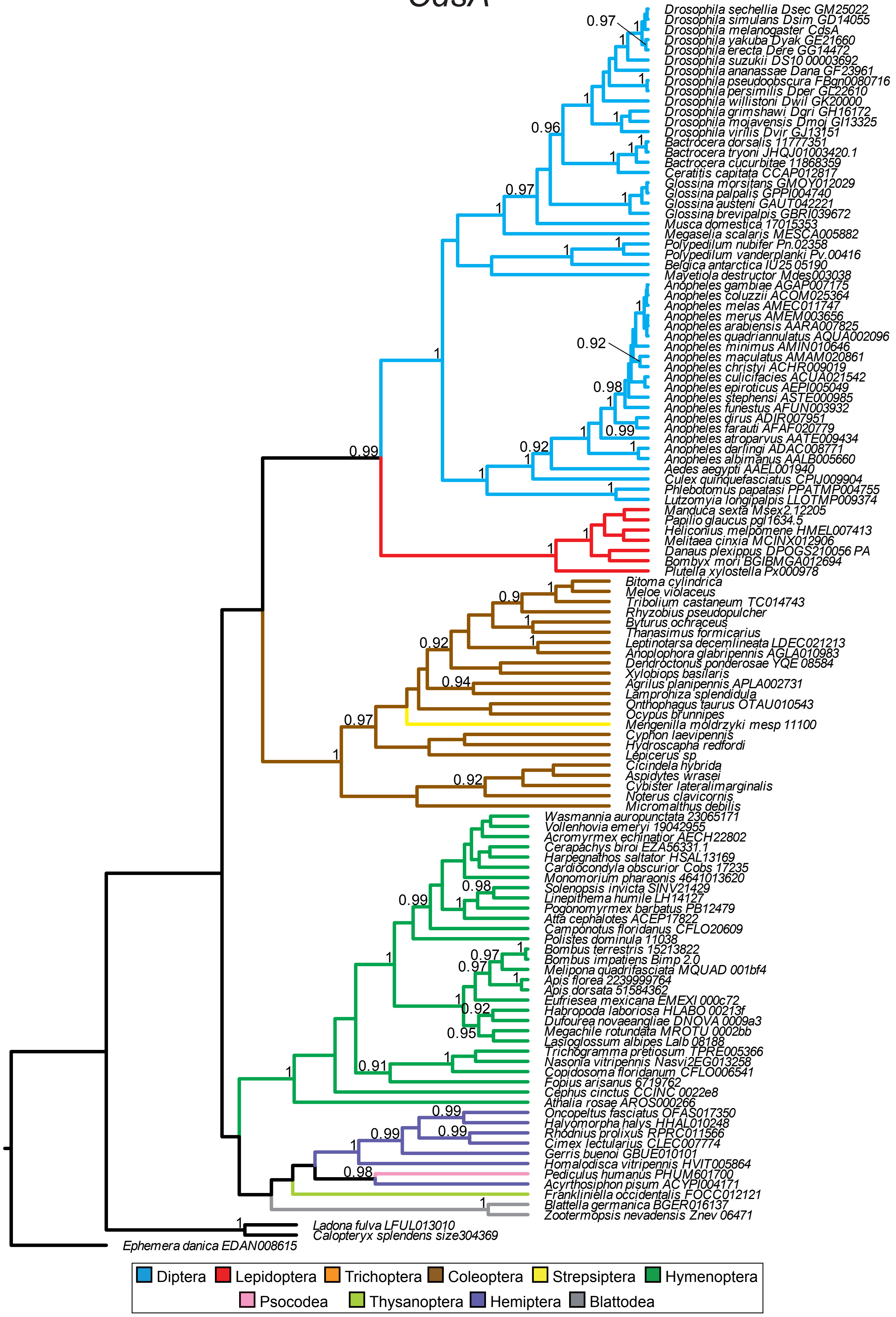

Drosophila sechellia Dsec GM25022
Drosophila simulans Dsim GD14055
Drosophila melanogaster CdsA
Drosophila yakuba Dyak GE21660
Drosophila erecta Dere GG14472
Drosophila suzukii DS10 00003692
Drosophila ananassae Dana GF23961
Drosophila pseudoobscura FBgn0080716
Drosophila persimilis Dper GL22610
Drosophila willistoni Dwil GK20000
Drosophila grimshawi Dgri GH16172
Drosophila mojavensis Dmoj GI13325
Drosophila virilis Dvir GJ13151
Bactrocera dorsalis 11777351
Bactrocera tryoni JHQJ01003420.1
Bactrocera cucurbitae 11868359
Ceratitis capitata CCAP012817
Glossina morsitans GMOY012029
Glossina palpalis GPPI004740
Glossina austeni GAUT042221
Glossina brevipalpis GBRI039672
Musca domestica 17015353
Megaselia scalaris MESCA005882
Polypedilum nubifer Pn.02358
Polypedilum vanderplanki Pv.00416
Belgica antarctica IU25 05190
Mayetiola destructor Mdes003038
Anopheles gambiae AGAP007175
Anopheles coluzzii ACOM025364
Anopheles melas AMEC011747
Anopheles merus AMEM003656
Anopheles arabiensis AARA007825
Anopheles quadriannulatus AQUA002096
Anopheles minimus AMIN010646
Anopheles maculatus AMAM020861
Anopheles christyi ACHR009019
Anopheles culicifacies ACUA021542
Anopheles epiroticus AEPI005049
Anopheles stephensi ASTE000985
Anopheles funestus AFUN003932
Anopheles dirus ADIR007951
Anopheles farauti AFAF020779
Anopheles atroparvus AATE009434
Anopheles darlingi ADAC008771
Anopheles albimanus AALB005660
Aedes aegypti AAEL001940
Culex quinquefasciatus CPIJ009904
Phlebotomus papatasi PPATMP004755
Lutzomyia longipalpis LLOTMP009374
Manduca sexta Msex2.12205
Papilio glaucus pgl1634.5
Heliconius melpomene HMEL007413
Melitaea cinxia MCINX012906
Danaus plexippus DPOGS210056 PA
Bombyx mori BGIBMGA012694
Plutella xylostella Px000978
Bitoma cylindrica
Meloe violaceus
Tribolium castaneum TC014743
Rhyzobius pseudopulcher
Byturus ochraceus
Thanasimus formicarius
Leptinotarsa decemlineata LDEC021213
Anoplophora glabripennis AGLA010983
Dendroctonus ponderosae YQE 08584
Xylobiops basilaris
Agrilus planipennis APLA002731
Lamprohiza splendidula
Onthophagus taurus OTAU010543
Ocypus brunnipes
Mengenilla moldrzyki mesp 11100
Cyphon laevipennis
Hydroscapha redfordi
Lepicerus sp
Cicindela hybrida
Aspidytes wrasei
Cybister lateralimarginalis
Noterus clavicornis
Micromalthus debilis
Wasmannia auropunctata 23065171
Vollenhovia emeryi 19042955
Acromyrmex echinatior AECH22802
Cerapachys biroi EZA56331.1
Harpegnathos saltator HSAL13169
Cardiocondyla obscurior Cobs 17235
Monomorium pharaonis 4641013620
Solenopsis invicta SINV21429
Linepithema humile LH14127
Pogonomyrmex barbatus PB12479
Atta cephalotes ACEP17822
Camponotus floridanus CFLO20609
Polistes dominula 11038
Bombus terrestris 15213822
Bombus impatiens Bimp 2.0
Melipona quadrifasciata MQUAD 001bf4
Apis florea 2239999764
Apis dorsata 51584362
Eufriesea mexicana EMEXI 000c72
Habropoda laboriosa HLABO 00213f
Dufourea novaeangliae DNOVA 0009a3
Megachile rotundata MROTU 0002bb
Lasioglossum albipes Lalb 08188
Trichogramma pretiosum TPRE005366
Nasonia vitripennis Nasvi2EG013258
Copidosoma floridanum CFLO006541
Fopius arisanus 6719762
Cephus cinctus CCINC 0022e8
Athalia rosae AROS000266
Oncopeltus fasciatus OFAS017350
Halyomorpha halys HHAL010248
Rhodnius prolixus RPRC011566
Cimex lectularius CLEC007774
Gerris buenoi GBUE010101
Homalodisca vitripennis HVIT005864
Pediculus humanus PHUM601700
Acyrthosiphon pisum ACYPI004171
Frankliniella occidentalis FOCC012121
Blattella germanica BGER016137
Zootermopsis nevadensis Znev 06471
Ladona fulva LFUL013010
Calopteryx splendens size304369
Ephemera danica EDAN008615
Diptera
Lepidoptera
Trichoptera
Coleoptera
Strepsiptera
Hymenoptera
Psocodea
Thysanoptera
Hemiptera
Blattodea

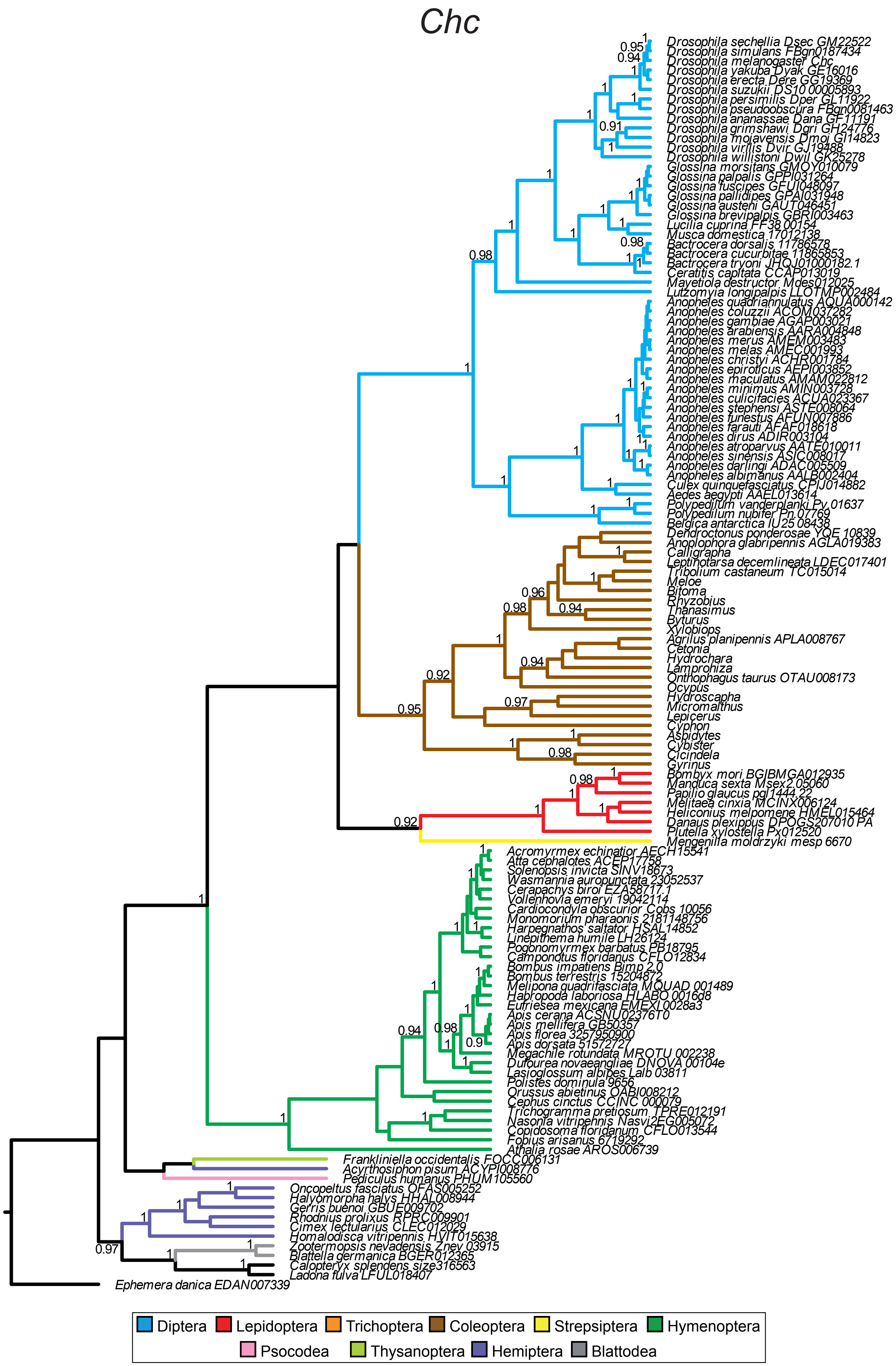

Chc
Drosophila sechellia Dsec GM22522
Drosophila simulans FBgn0187434
Drosophila melanogaster Chc
Drosophila yakuba Dyak GE16016
Drosophila erecta Dere GG19369
Drosophila suzukii DS10 00005893
Drosophila persimilis Dper GL11922
Drosophila pseudoobscura FBgn0081463
Drosophila ananassae Dana GF11191
Drosophila grimshawi Dgri GH24776
Drosophila mojavensis Dmoj GI14823
Drosophila virilis Dvir GJ19488
Drosophila willistoni Dwil GK25278
Glossina morsitans GMOY010079
Glossina palpalis GPPI031264
Glossina fuscipes GFUI048097
Glossina pallidipes GPAI031948
Glossina austeni GAUT046451
Glossina brevipalpis GBRI003463
Lucilia cuprina FF38 00154
Musca domestica 17012138
Bactrocera dorsalis 11786578
Bactrocera cucurbitae 11865853
Bactrocera tryoni JHQJ01000182.1
Ceratitis capitata CCAP013019
Mayetiola destructor Mdes012025
Lutzomyia longipalpis LLOTMP002484
Anopheles quadriannulatus AQUA000142
Anopheles coluzzii ACOM037282
Anopheles gambiae AGAP003021
Anopheles arabiensis AARA004848
Anopheles merus AMEM003483
Anopheles melas AMEC001993
Anopheles christyi ACHR001784
Anopheles epiroticus AEPI003852
Anopheles maculatus AMAM022812
Anopheles minimus AMIN003728
Anopheles culicifacies ACUA023367
Anopheles stephensi ASTE008064
Anopheles funestus AFUN007886
Anopheles farauti AFAF018618
Anopheles dirus ADIR003104
Anopheles atroparvus AATE010011
Anopheles sinensis ASIC008017
Anopheles darlingi ADAC005509
Anopheles albimanus AALB002404
Culex quinquefasciatus CPIJ014882
Aedes aegypti AAEL013614
Polypedilum vanderplanki Pv.01637
Polypedilum nubifer Pn.07769
Belgica antarctica IU25 08438
Dendroctonus ponderosae YQE 10839
Anoplophora glabripennis AGLA019383
Calligrapha
Leptinotarsa decemlineata LDEC017401
Tribolium castaneum TC015014
Meloe
Bitoma
Rhyzobius
Thanasimus
Byturus
Xylobiops
Agrilus planipennis APLA008767
Cetonia
Hydrochara
Lamprohiza
Onthophagus taurus OTAU008173
Ocypus
Hydroscapha
Micromalthus
Lepicerus
Cyphon
Aspidytes
Cybister
Cicindela
Gyrinus
Bombyx mori BGIBMGA012935
Manduca sexta Msex2.05060
Papilio glaucus pgl1444.22
Melitaea cinxia MCINX006124
Heliconius melpomene HMEL015464
Danaus plexippus DPOGS207010 PA
Plutella xylostella Px012520
Mengenilla moldrzyki mesp 6670
Acromyrmex echinatior AECH15541
Atta cephalotes ACEP17758
Solenopsis invicta SINV18673
Wasmannia auropunctata 23052537
Cerapachys biroi EZA58717.1
Vollenhovia emeryi 19042114
Cardiocondyla obscurior Cobs 10056
Monomorium pharaonis 2181148756
Harpegnathos saltator HSAL14852
Linepithema humile LH26124
Pogonomyrmex barbatus PB18795
Camponotus floridanus CFLO12834
Bombus impatiens Bimp 2.0
Bombus terrestris 15204872
Melipona quadrifasciata MQUAD 001489
Habropoda laboriosa HLABO 0016d8
Eufriesea mexicana EMEXI 0028a3
Apis cerana ACSNU02376T0
Apis mellifera GB50357
Apis florea 3257950900
Apis dorsata 51572727
Megachile rotundata MROTU 002238
Dufourea novaeangliae DNOVA 00104e
Lasioglossum albipes Lalb 03811
Polistes dominula 9656
Orussus abietinus OABI008212
Cephus cinctus CCINC 000079
Trichogramma pretiosum TPRE012191
Nasonia vitripennis Nasvi2EG005072
Copidosoma floridanum CFLO013544
Fopius arisanus 6719292
Athalia rosae AROS006739
Frankliniella occidentalis FOCC006131
Acyrthosiphon pisum ACYPI008776
Pediculus humanus PHUM105560
Oncopeltus fasciatus OFAS005252
Halyomorpha halys HHAL008944
Gerris buenoi GBUE009702
Rhodnius prolixus RPRC009901
Cimex lectularius CLEC012029
Homalodisca vitripennis HVIT015638
Zootermopsis nevadensis Znev 03915
Blattella germanica BGER012365
Calopteryx splendens size316563
Ladona fulva LFUL018407
Ephemera danica EDAN007339
Diptera
Lepidoptera
Trichoptera
Coleoptera
Strepsiptera
Hymenoptera
Psocodea
Thysanoptera
Hemiptera
Blattodea

Cul3

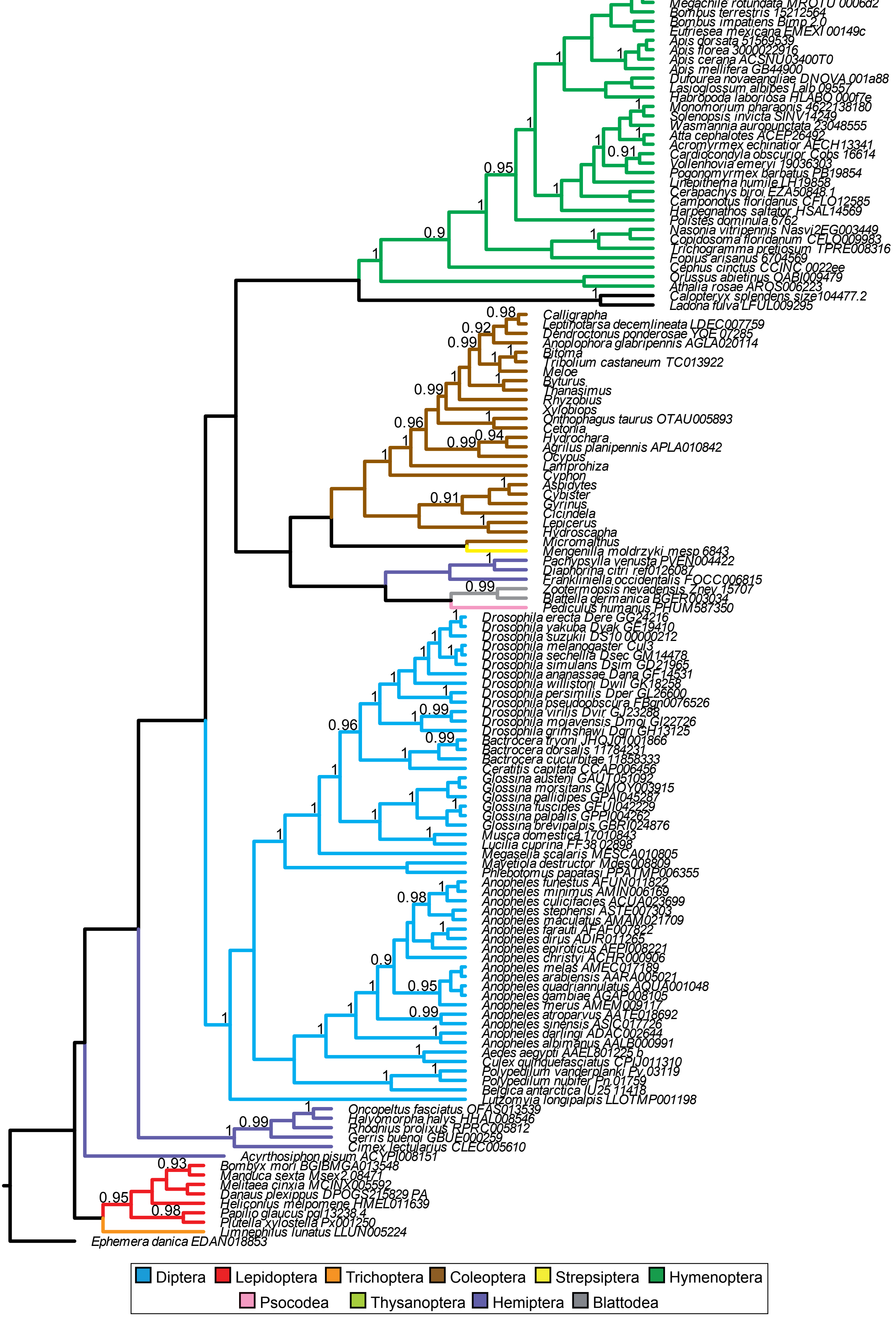
Melipona quadrifasciata MQUAD 00269f
Megachile rotundata MROTU 0006d2
Bombus terrestris 15212564
Bombus impatiens Bimp 2.0
Eufriesea mexicana EMEXI 00149c
Apis dorsata 51569539
Apis florea 3000022916
Apis cerana ACSNU03400T0
Apis mellifera GB44900
Dufourea novaeangliae DNOVA 001a88
Lasioglossum albipes Lalb 09557
Habropoda laboriosa HLABO 000f7e
Monomorium pharaonis 4622138180
Solenopsis invicta SINV14249
Wasmannia auropunctata 23048555
Atta cephalotes ACEP26492
Acromyrmex echinatior AECH13341
Cardiocondyla obscurior Cobs 16614
Vollenhovia emeryi 19036303
Pogonomyrmex barbatus PB19854
Linepithema humile LH19858
Cerapachys biroi EZA50848.1
Camponotus floridanus CFLO12585
Harpegnathos saltator HSAL14569
Polistes dominula 6762
Nasonia vitripennis Nasvi2EG003449
Copidosoma floridanum CFLO009983
Trichogramma pretiosum TPRE008316
Fopius arisanus 6704569
Cephus cinctus CCINC 0022ee
Orussus abietinus OABI009479
Athalia rosae AROS006223
Calopteryx splendens size104477.2
Ladona fulva LFUL009295
Calligrapha
Leptinotarsa decemlineata LDEC007759
Dendroctonus ponderosae YQE 07285
Anoplophora glabripennis AGLA020114
Bitoma
Tribolium castaneum TC013922
Meloe
Byturus
Thanasimus
Rhyzobius
Xylobiops
Onthophagus taurus OTAU005893
Cetonia
Hydrochara
Agrilus planipennis APLA010842
Ocypus
Lamprohiza
Cyphon
Aspidytes
Cybister
Gyrinus
Cicindela
Lepicerus
Hydroscapha
Micromalthus
Mengenilla moldrzyki mesp 6843
Pachypsylla venusta PVEN004422
Diaphorina citri ref0126087
Frankliniella occidentalis FOCC006815
Zootermopsis nevadensis Znev 15707
Blattella germanica BGER003034
Pediculus humanus PHUM587350
Drosophila erecta Dere GG24216
Drosophila yakuba Dyak GE19410
Drosophila suzukii DS10 00000212
Drosophila melanogaster Cul3
Drosophila sechellia Dsec GM14478
Drosophila simulans Dsim GD21965
Drosophila ananassae Dana GF14531
Drosophila willistoni Dwil GK18258
Drosophila persimilis Dper GL26600
Drosophila pseudoobscura FBgn0076526
Drosophila virilis Dvir GJ23288
Drosophila mojavensis Dmoj GI22726
Drosophila grimshawi Dgri GH13125
Bactrocera tryoni JHQJ01001866
Bactrocera dorsalis 11784231
Bactrocera cucurbitae 11858333
Ceratitis capitata CCAP006456
Glossina austeni GAUT051092
Glossina morsitans GMOY003915
Glossina pallidipes GPAI045287
Glossina fuscipes GFUI042229
Glossina palpalis GPPI004262
Glossina brevipalpis GBRI024876
Musca domestica 17010843
Lucilia cuprina FF38 02898
Megaselia scalaris MESCA010805
Mayetiola destructor Mdes008809
Phlebotomus papatasi PPATMP006355
Anopheles funestus AFUN011822
Anopheles minimus AMIN006169
Anopheles culicifacies ACUA023699
Anopheles stephensi ASTE007303
Anopheles maculatus AMAM021709
Anopheles farauti AFAF007822
Anopheles dirus ADIR011265
Anopheles epiroticus AEPI008221
Anopheles christyi ACHR000906
Anopheles melas AMEC017189
Anopheles arabiensis AARA005021
Anopheles quadriannulatus AQUA001048
Anopheles gambiae AGAP008105
Anopheles merus AMEM009117
Anopheles atroparvus AATE018692
Anopheles sinensis ASIC017726
Anopheles darlingi ADAC002644
Anopheles albimanus AALB000991
Aedes aegypti AAEL801225 b
Culex quinquefasciatus CPIJ011310
Polypedilum vanderplanki Pv.03119
Polypedilum nubifer Pn.01759
Belgica antarctica IU25 11418
Lutzomyia longipalpis LLOTMP001198
Oncopeltus fasciatus OFAS013539
Halyomorpha halys HHAL008546
Rhodnius prolixus RPRC005812
Gerris buenoi GBUE000259
Cimex lectularius CLEC005610
Acyrthosiphon pisum ACYPI008151
Bombyx mori BGIBMGA013548
Manduca sexta Msex2.08471
Melitaea cinxia MCINX005592
Danaus plexippus DPOGS215829 PA
Heliconius melpomene HMEL011639
Papilio glaucus pgl13238.4
Plutella xylostella Px001250
Limnephilus lunatus LLUN005224
Ephemera danica EDAN018853
Diptera
Lepidoptera
Trichoptera
Coleoptera
Strepsiptera
Hymenoptera
Psocodea
Thysanoptera
Hemiptera
Blattodea

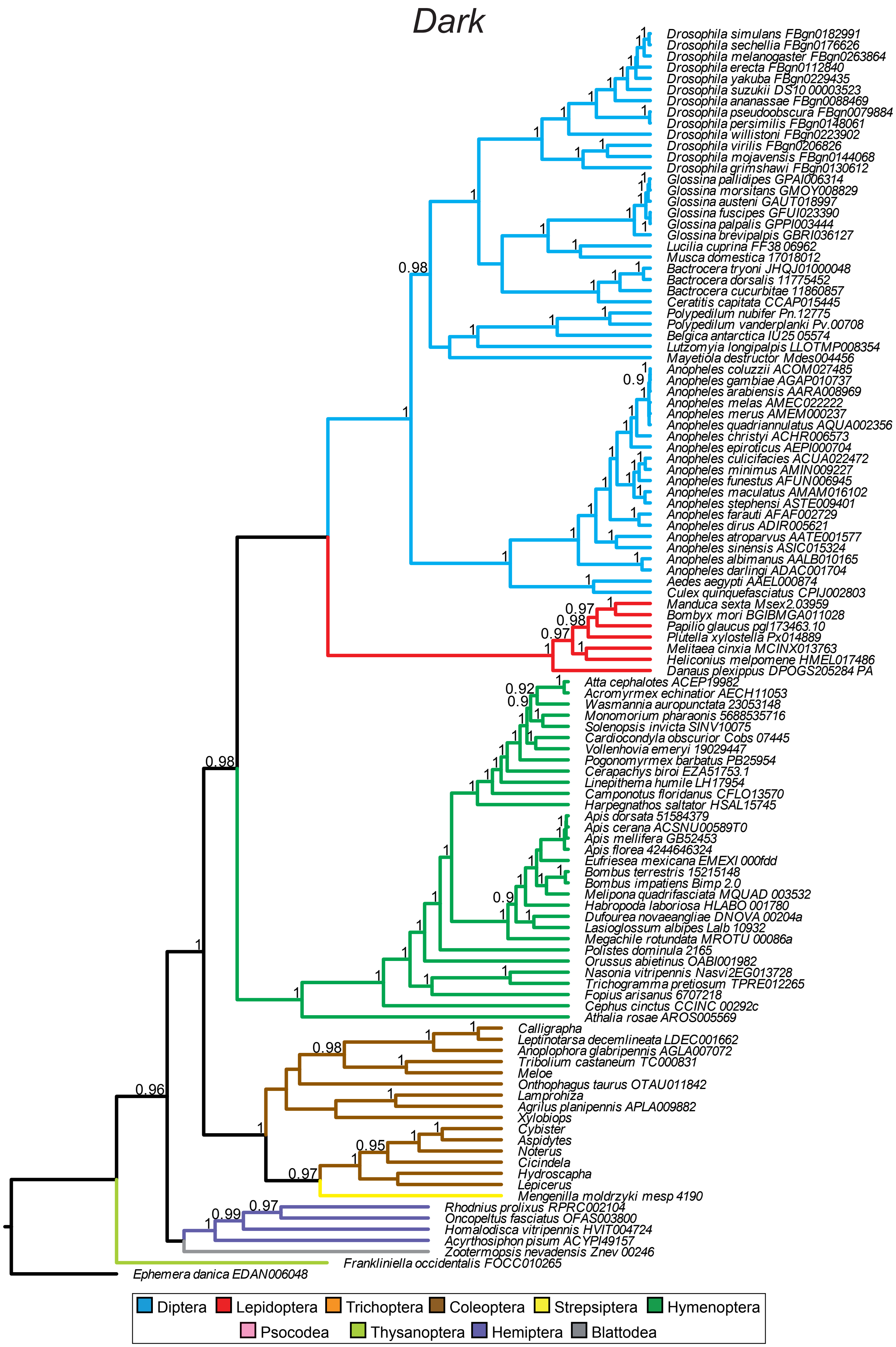

Dark
Drosophila simulans FBgn0182991
Drosophila sechellia FBgn0176626
Drosophila melanogaster FBgn0263864
Drosophila erecta FBgn0112840
Drosophila yakuba FBgn0229435
Drosophila suzukii DS10 00003523
Drosophila ananassae FBgn0088469
Drosophila pseudoobscura FBgn0079884
Drosophila persimilis FBgn0148061
Drosophila willistoni FBgn0223902
Drosophila virilis FBgn0206826
Drosophila mojavensis FBgn0144068
Drosophila grimshawi FBgn0130612
Glossina pallidipes GPAI006314
Glossina morsitans GMOY008829
Glossina austeni GAUT018997
Glossina fuscipes GFUI023390
Glossina palpalis GPPI003444
Glossina brevipalpis GBRI036127
Lucilia cuprina FF38 06962
Musca domestica 17018012
Bactrocera tryoni JHQJ01000048
Bactrocera dorsalis 11775452
Bactrocera cucurbitae 11860857
Ceratitis capitata CCAP015445
Polypedilum nubifer Pn.12775
Polypedilum vanderplanki Pv.00708
Belgica antarctica IU25 05574
Lutzomyia longipalpis LLOTMP008354
Mayetiola destructor Mdes004456
Anopheles coluzzii ACOM027485
Anopheles gambiae AGAP010737
Anopheles arabiensis AARA008969
Anopheles melas AMEC022222
Anopheles merus AMEM000237
Anopheles quadriannulatus AQUA002356
Anopheles christyi ACHR006573
Anopheles epiroticus AEPI000704
Anopheles culicifacies ACUA022472
Anopheles minimus AMIN009227
Anopheles funestus AFUN006945
Anopheles maculatus AMAM016102
Anopheles stephensi ASTE009401
Anopheles farauti AFAF002729
Anopheles dirus ADIR005621
Anopheles atroparvus AATE001577
Anopheles sinensis ASIC015324
Anopheles albimanus AALB010165
Anopheles darlingi ADAC001704
Aedes aegypti AAEL000874
Culex quinquefasciatus CPIJ002803
Manduca sexta Msex2.03959
Bombyx mori BGIBMGA011028
Papilio glaucus pgl173463.10
Plutella xylostella Px014889
Melitaea cinxia MCINX013763
Heliconius melpomene HMEL017486
Danaus plexippus DPOGS205284 PA
Atta cephalotes ACEP19982
Acromyrmex echinatior AECH11053
Wasmannia auropunctata 23053148
Monomorium pharaonis 5688535716
Solenopsis invicta SINV10075
Cardiocondyla obscurior Cobs 07445
Vollenhovia emeryi 19029447
Pogonomyrmex barbatus PB25954
Cerapachys biroi EZA51753.1
Linepithema humile LH17954
Camponotus floridanus CFLO13570
Harpegnathos saltator HSAL15745
Apis dorsata 51584379
Apis cerana ACSNU00589T0
Apis mellifera GB52453
Apis florea 4244646324
Eufriesea mexicana EMEXI 000fdd
Bombus terrestris 15215148
Bombus impatiens Bimp 2.0
Melipona quadrifasciata MQUAD 003532
Habropoda laboriosa HLABO 001780
Dufourea novaeangliae DNOVA 00204a
Lasioglossum albipes Lalb 10932
Megachile rotundata MROTU 00086a
Polistes dominula 2165
Orussus abietinus OABI001982
Nasonia vitripennis Nasvi2EG013728
Trichogramma pretiosum TPRE012265
Fopius arisanus 6707218
Cephus cinctus CCINC 00292c
Athalia rosae AROS005569
Calligrapha
Leptinotarsa decemlineata LDEC001662
Anoplophora glabripennis AGLA007072
Tribolium castaneum TC000831
Meloe
Onthophagus taurus OTAU011842
Lamprohiza
Agrilus planipennis APLA009882
Xylobiops
Cybister
Aspidytes
Noterus
Cicindela
Hydroscapha
Lepicerus
Mengenilla moldrzyki mesp 4190
Rhodnius prolixus RPRC002104
Oncopeltus fasciatus OFAS003800
Homalodisca vitripennis HVIT004724
Acyrthosiphon pisum ACYPI49157
Zootermopsis nevadensis Znev 00246
Frankliniella occidentalis FOCC010265
Ephemera danica EDAN006048
Diptera
Lepidoptera
Trichoptera
Coleoptera
Strepsiptera
Hymenoptera
Psocodea
Thysanoptera
Hemiptera
Blattodea

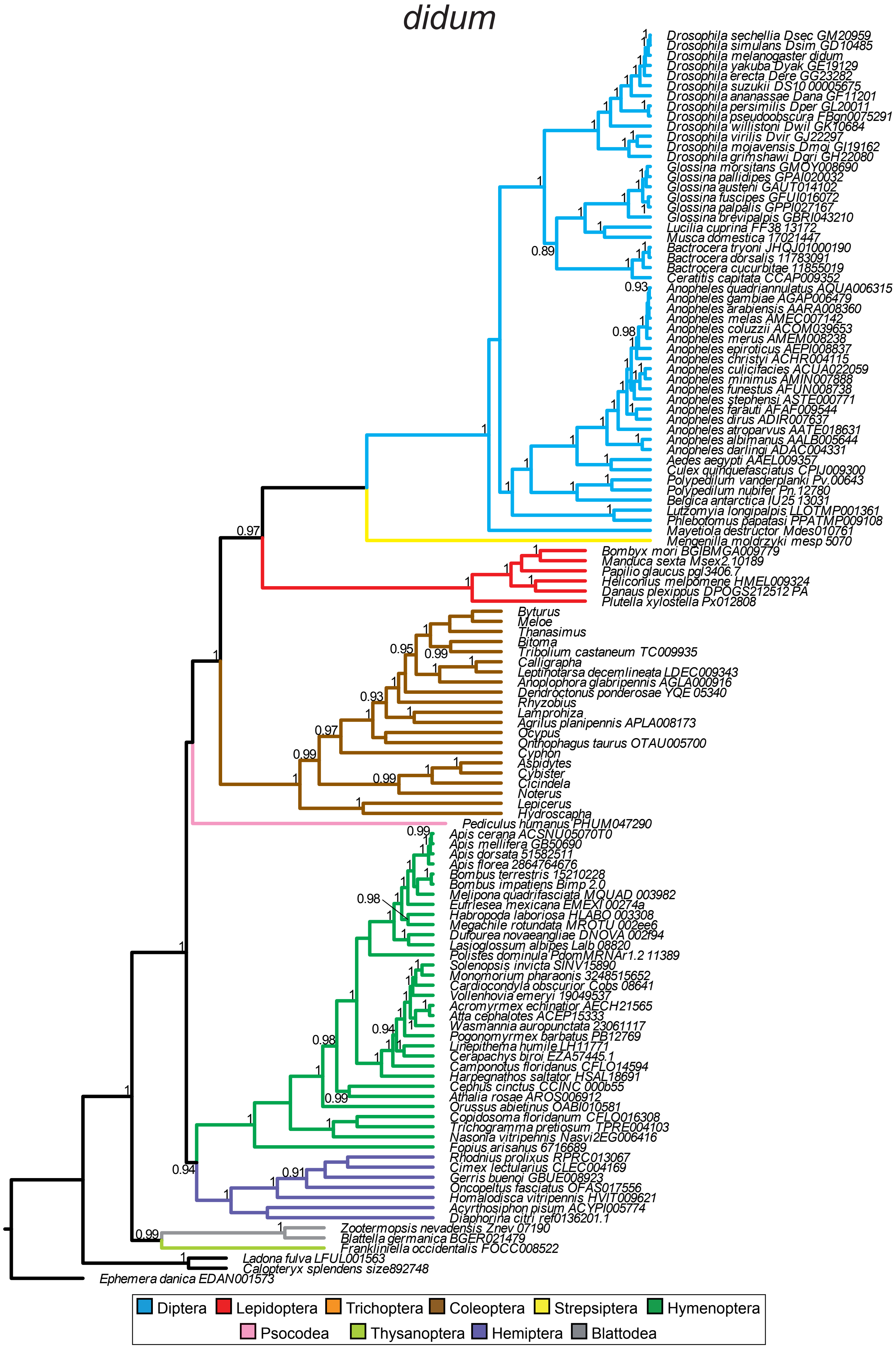
didum
Drosophila sechellia Dsec GM20959
Drosophila simulans Dsim GD10485
Drosophila melanogaster didum
Drosophila yakuba Dyak GE19129
Drosophila erecta Dere GG23282
Drosophila suzukii DS10 00005675
Drosophila ananassae Dana GF11201
Drosophila persimilis Dper GL20011
Drosophila pseudoobscura FBgn0075291
Drosophila willistoni Dwil GK10684
Drosophila virilis Dvir GJ22297
Drosophila mojavensis Dmoj GI19162
Drosophila grimshawi Dgri GH22080
Glossina morsitans GMOY008690
Glossina pallidipes GPAI020032
Glossina austeni GAUT014102
Glossina fuscipes GFUI016072
Glossina palpalis GPPI027167
Glossina brevipalpis GBRI043210
Lucilia cuprina FF38 13172
Musca domestica 17021447
Bactrocera tryoni JHQJ01000190
Bactrocera dorsalis 11783091
Bactrocera cucurbitae 11855019
Ceratitis capitata CCAP009352
Anopheles quadriannulatus AQUA006315
Anopheles gambiae AGAP006479
Anopheles arabiensis AARA008360
Anopheles melas AMEC007142
Anopheles coluzzii ACOM039653
Anopheles merus AMEM008238
Anopheles epiroticus AEPI008837
Anopheles christyi ACHR004115
Anopheles culicifacies ACUA022059
Anopheles minimus AMIN007888
Anopheles funestus AFUN008738
Anopheles stephensi ASTE000771
Anopheles farauti AFAF009544
Anopheles dirus ADIR007637
Anopheles atroparvus AATE018631
Anopheles albimanus AALB005644
Anopheles darlingi ADAC004331
Aedes aegypti AAEL009357
Culex quinquefasciatus CPIJ009300
Polypedilum vanderplanki Pv.00643
Polypedilum nubifer Pn.12780
Belgica antarctica IU25 13031
Lutzomyia longipalpis LLOTMP001361
Phlebotomus papatasi PPATMP009108
Mayetiola destructor Mdes010761
Mengenilla moldrzyki mesp 5070
Bombyx mori BGIBMGA009779
Manduca sexta Msex2.10189
Papilio glaucus pgl3406.7
Heliconius melpomene HMEL009324
Danaus plexippus DPOGS212512 PA
Plutella xylostella Px012808
Byturus
Meloe
Thanasimus
Bitoma
Tribolium castaneum TC009935
Calligrapha
Leptinotarsa decemlineata LDEC009343
Anoplophora glabripennis AGLA000916
Dendroctonus ponderosae YQE 05340
Rhyzobius
Lamprohiza
Agrilus planipennis APLA008173
Ocypus
Onthophagus taurus OTAU005700
Cyphon
Aspidytes
Cybister
Cicindela
Noterus
Lepicerus
Hydroscapha
Pediculus humanus PHUM047290
Apis cerana ACSNU05070T0
Apis mellifera GB50690
Apis dorsata 51582511
Apis florea 2864764676
Bombus terrestris 15210228
Bombus impatiens Bimp 2.0
Melipona quadrifasciata MQUAD 003982
Eufriesea mexicana EMEXI 00274a
Habropoda laboriosa HLABO 003308
Megachile rotundata MROTU 002ee6
Dufourea novaeangliae DNOVA 002f94
Lasioglossum albipes Lalb 08820
Polistes dominula PdomMRNAr1.2 11389
Solenopsis invicta SINV15890
Monomorium pharaonis 3248515652
Cardiocondyla obscurior Cobs 08641
Vollenhovia emeryi 19049537
Acromyrmex echinatior AECH21565
Atta cephalotes ACEP15333
Wasmannia auropunctata 23061117
Pogonomyrmex barbatus PB12769
Linepithema humile LH11771
Cerapachys biroi EZA57445.1
Camponotus floridanus CFLO14594
Harpegnathos saltator HSAL18691
Cephus cinctus CCINC 000b55
Athalia rosae AROS006912
Orussus abietinus OABI010581
Copidosoma floridanum CFLO016308
Trichogramma pretiosum TPRE004103
Nasonia vitripennis Nasvi2EG006416
Fopius arisanus 6716689
Rhodnius prolixus RPRC013067
Cimex lectularius CLEC004169
Gerris buenoi GBUE008923
Oncopeltus fasciatus OFAS017556
Homalodisca vitripennis HVIT009621
Acyrthosiphon pisum ACYPI005774
Diaphorina citri ref0136201.1
Zootermopsis nevadensis Znev 07190
Blattella germanica BGER021479
Frankliniella occidentalis FOCC008522
Ladona fulva LFUL001563
Calopteryx splendens size892748
Ephemera danica EDAN001573
Diptera
Lepidoptera
Trichoptera
Coleoptera
Strepsiptera
Hymenoptera
Psocodea
Thysanoptera
Hemiptera
Blattodea

Dredd

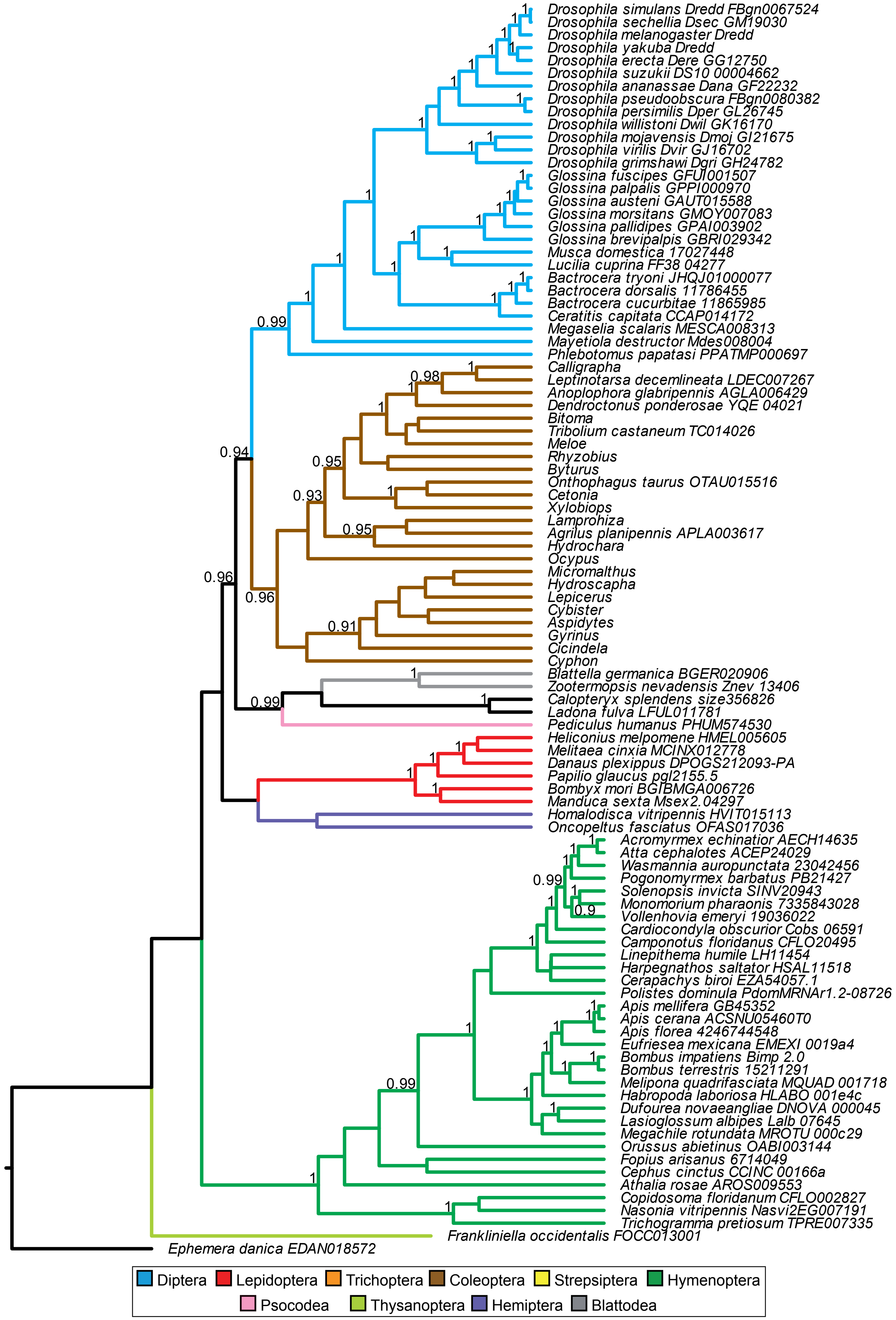

Drosophila simulans Dredd FBgn0067524
Drosophila sechellia Dsec GM19030
Drosophila melanogaster Dredd
Drosophila yakuba Dredd
Drosophila erecta Dere GG12750
Drosophila suzukii DS10 00004662
Drosophila ananassae Dana GF22232
Drosophila pseudoobscura FBgn0080382
Drosophila persimilis Dper GL26745
Drosophila willistoni Dwil GK16170
Drosophila mojavensis Dmoj GI21675
Drosophila virilis Dvir GJ16702
Drosophila grimshawi Dgri GH24782
Glossina fuscipes GFUI001507
Glossina palpalis GPPI000970
Glossina austeni GAUT015588
Glossina morsitans GMOY007083
Glossina pallidipes GPAI003902
Glossina brevipalpis GBRI029342
Musca domestica 17027448
Lucilia cuprina FF38 04277
Bactrocera tryoni JHQJ01000077
Bactrocera dorsalis 11786455
Bactrocera cucurbitae 11865985
Ceratitis capitata CCAP014172
Megaselia scalaris MESCA008313
Mayetiola destructor Mdes008004
Phlebotomus papatasi PPATMP000697
Calligrapha
Leptinotarsa decemlineata LDEC007267
Anoplophora glabripennis AGLA006429
Dendroctonus ponderosae YQE 04021
Bitoma
Tribolium castaneum TC014026
Meloe
Rhyzobius
Byturus
Onthophagus taurus OTAU015516
Cetonia
Xylobiops
Lamprohiza
Agrilus planipennis APLA003617
Hydrochara
Ocypus
Micromalthus
Hydroscapha
Lepicerus
Cybister
Aspidytes
Gyrinus
Cicindela
Cyphon
Blattella germanica BGER020906
Zootermopsis nevadensis Znev 13406
Calopteryx splendens size356826
Ladona fulva LFUL011781
Pediculus humanus PHUM574530
Heliconius melpomene HMEL005605
Melitaea cinxia MCINX012778
Danaus plexippus DPOGS212093-PA
Papilio glaucus pgl2155.5
Bombyx mori BGIBMGA006726
Manduca sexta Msex2.04297
Homalodisca vitripennis HVIT015113
Oncopeltus fasciatus OFAS017036
Acromyrmex echinatior AECH14635
Atta cephalotes ACEP24029
Wasmannia auropunctata 23042456
Pogonomyrmex barbatus PB21427
Solenopsis invicta SINV20943
Monomorium pharaonis 7335843028
Vollenhovia emeryi 19036022
Cardiocondyla obscurior Cobs 06591
Camponotus floridanus CFLO20495
Linepithema humile LH11454
Harpegnathos saltator HSAL11518
Cerapachys biroi EZA54057.1
Polistes dominula PdomMRNAr1.2-08726
Apis mellifera GB45352
Apis cerana ACSNU05460T0
Apis florea 4246744548
Eufriesea mexicana EMEXI 0019a4
Bombus impatiens Bimp 2.0
Bombus terrestris 15211291
Melipona quadrifasciata MQUAD 001718
Habropoda laboriosa HLABO 001e4c
Dufourea novaeangliae DNOVA 000045
Lasioglossum albipes Lalb 07645
Megachile rotundata MROTU 000c29
Orussus abietinus OABI003144
Fopius arisanus 6714049
Cephus cinctus CCINC 00166a
Athalia rosae AROS009553
Copidosoma floridanum CFLO002827
Nasonia vitripennis Nasvi2EG007191
Trichogramma pretiosum TPRE007335
Frankliniella occidentalis FOCC013001
Ephemera danica EDAN018572
Diptera
Lepidoptera
Trichoptera
Coleoptera
Strepsiptera
Hymenoptera
Psocodea
Thysanoptera
Hemiptera
Blattodea

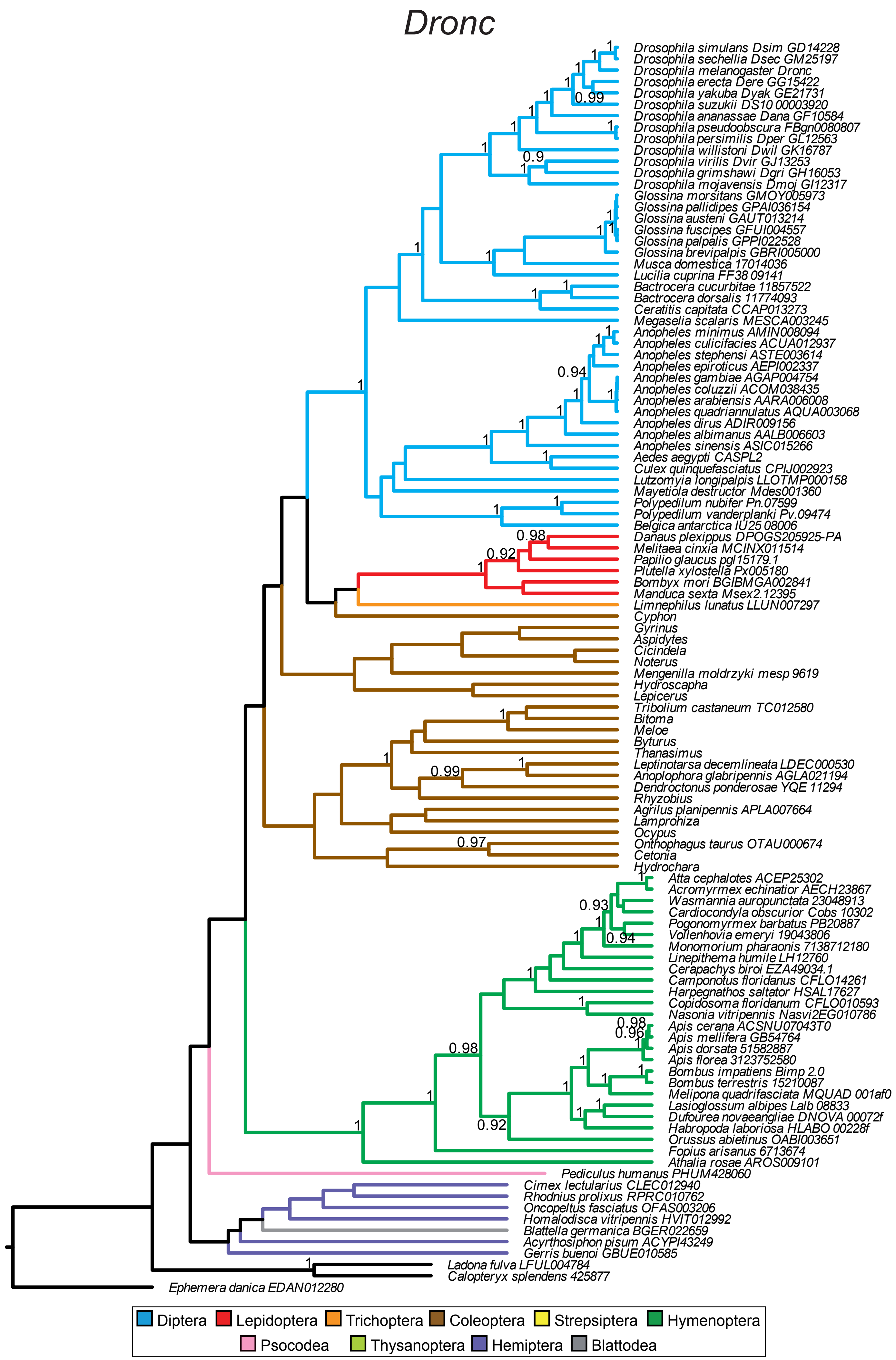
Dronc
Drosophila simulans Dsim GD14228
Drosophila sechellia Dsec GM25197
Drosophila melanogaster Dronc
Drosophila erecta Dere GG15422
Drosophila yakuba Dyak GE21731
Drosophila suzukii DS10 00003920
Drosophila ananassae Dana GF10584
Drosophila pseudoobscura FBgn0080807
Drosophila persimilis Dper GL12563
Drosophila willistoni Dwil GK16787
Drosophila virilis Dvir GJ13253
Drosophila grimshawi Dgri GH16053
Drosophila mojavensis Dmoj GI12317
Glossina morsitans GMOY005973
Glossina pallidipes GPAI036154
Glossina austeni GAUT013214
Glossina fuscipes GFUI004557
Glossina palpalis GPPI022528
Glossina brevipalpis GBRI005000
Musca domestica 17014036
Lucilia cuprina FF38 09141
Bactrocera cucurbitae 11857522
Bactrocera dorsalis 11774093
Ceratitis capitata CCAP013273
Megaselia scalaris MESCA003245
Anopheles minimus AMIN008094
Anopheles culicifacies ACUA012937
Anopheles stephensi ASTE003614
Anopheles epiroticus AEPI002337
Anopheles gambiae AGAP004754
Anopheles coluzzii ACOM038435
Anopheles arabiensis AARA006008
Anopheles quadriannulatus AQUA003068
Anopheles dirus ADIR009156
Anopheles albimanus AALB006603
Anopheles sinensis ASIC015266
Aedes aegypti CASPL2
Culex quinquefasciatus CPIJ002923
Lutzomyia longipalpis LLOTMP000158
Mayetiola destructor Mdes001360
Polypedilum nubifer Pn.07599
Polypedilum vanderplanki Pv.09474
Belgica antarctica IU25 08006
Danaus plexippus DPOGS205925-PA
Melitaea cinxia MCINX011514
Papilio glaucus pgl15179.1
Plutella xylostella Px005180
Bombyx mori BGIBMGA002841
Manduca sexta Msex2.12395
Limnephilus lunatus LLUN007297
Cyphon
Gyrinus
Aspidytes
Cicindela
Noterus
Mengenilla moldrzyki mesp 9619
Hydroscapha
Lepicerus
Tribolium castaneum TC012580
Bitoma
Meloe
Byturus
Thanasimus
Leptinotarsa decemlineata LDEC000530
Anoplophora glabripennis AGLA021194
Dendroctonus ponderosae YQE 11294
Rhyzobius
Agrilus planipennis APLA007664
Lamprohiza
Ocypus
Onthophagus taurus OTAU000674
Cetonia
Hydrochara
Atta cephalotes ACEP25302
Acromyrmex echinatior AECH23867
Wasmannia auropunctata 23048913
Cardiocondyla obscurior Cobs 10302
Pogonomyrmex barbatus PB20887
Vollenhovia emeryi 19043806
Monomorium pharaonis 7138712180
Linepithema humile LH12760
Cerapachys biroi EZA49034.1
Camponotus floridanus CFLO14261
Harpegnathos saltator HSAL17627
Copidosoma floridanum CFLO010593
Nasonia vitripennis Nasvi2EG010786
Apis cerana ACSNU07043T0
Apis mellifera GB54764
Apis dorsata 51582887
Apis florea 3123752580
Bombus impatiens Bimp 2.0
Bombus terrestris 15210087
Melipona quadrifasciata MQUAD 001af0
Lasioglossum albipes Lalb 08833
Dufourea novaeangliae DNOVA 00072f
Habropoda laboriosa HLABO 00228f
Orussus abietinus OABI003651
Fopius arisanus 6713674
Athalia rosae AROS009101
Pediculus humanus PHUM428060
Cimex lectularius CLEC012940
Rhodnius prolixus RPRC010762
Oncopeltus fasciatus OFAS003206
Homalodisca vitripennis HVIT012992
Blattella germanica BGER022659
Acyrthosiphon pisum ACYPI43249
Gerris buenoi GBUE010585
Ladona fulva LFUL004784
Calopteryx splendens 425877
Ephemera danica EDAN012280
Diptera
Lepidoptera
Trichoptera
Coleoptera
Strepsiptera
Hymenoptera
Psocodea
Thysanoptera
Hemiptera
Blattodea

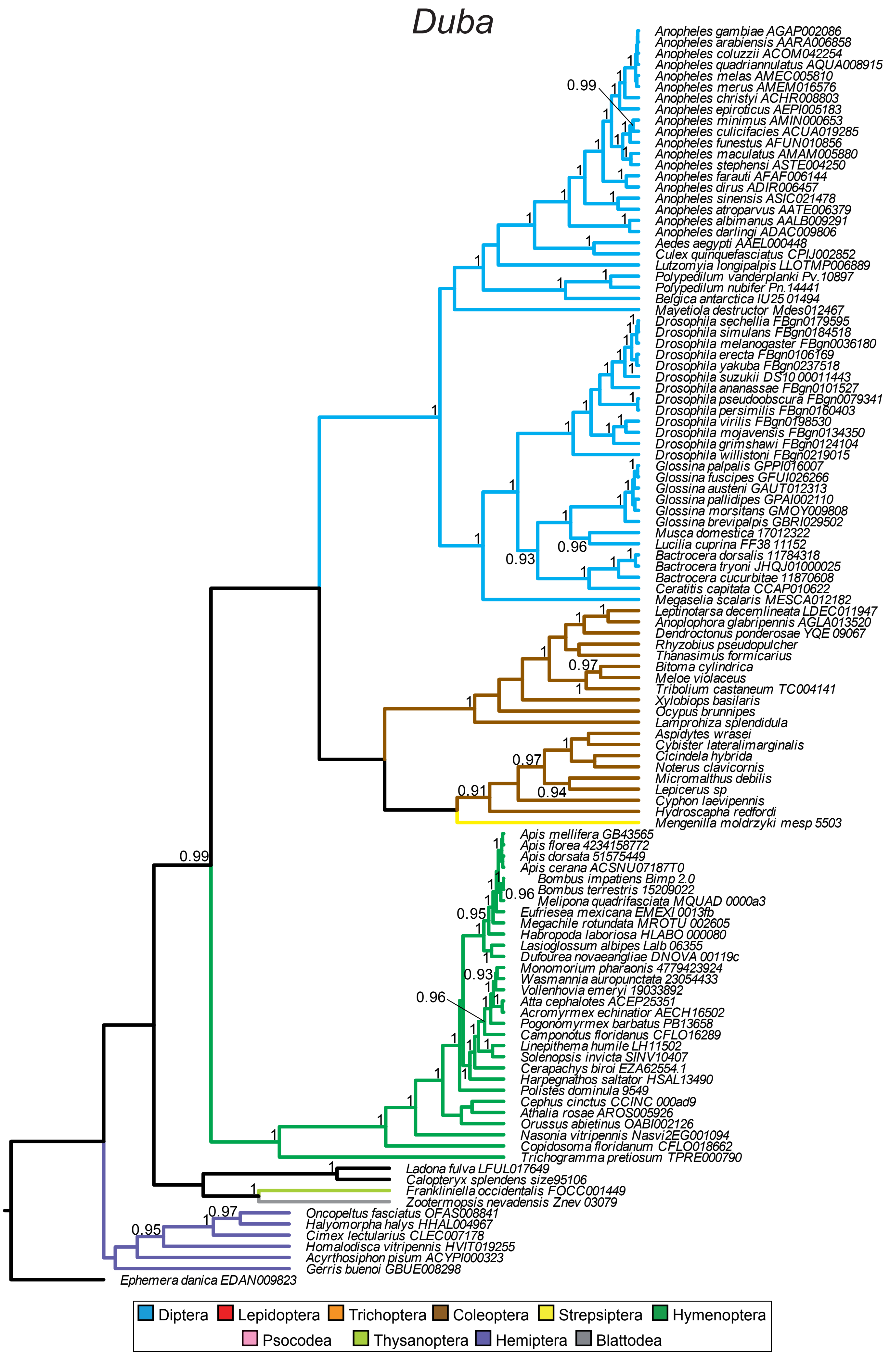
Duba
Anopheles gambiae AGAP002086
Anopheles arabiensis AARA006858
Anopheles coluzzii ACOM042254
Anopheles quadriannulatus AQUA008915
Anopheles melas AMEC005810
Anopheles merus AMEM016576
Anopheles christyi ACHR008803
Anopheles epiroticus AEPI005183
Anopheles minimus AMIN000653
Anopheles culicifacies ACUA019285
Anopheles funestus AFUN010856
Anopheles maculatus AMAM005880
Anopheles stephensi ASTE004250
Anopheles farauti AFAF006144
Anopheles dirus ADIR006457
Anopheles sinensis ASIC021478
Anopheles atroparvus AATE006379
Anopheles albimanus AALB009291
Anopheles darlingi ADAC009806
Aedes aegypti AAEL000448
Culex quinquefasciatus CPIJ002852
Lutzomyia longipalpis LLOTMP006889
Polypedilum vanderplanki Pv.10897
Polypedilum nubifer Pn.14441
Belgica antarctica IU25 01494
Mayetiola destructor Mdes012467
Drosophila sechellia FBgn0179595
Drosophila simulans FBgn0184518
Drosophila melanogaster FBgn0036180
Drosophila erecta FBgn0106169
Drosophila yakuba FBgn0237518
Drosophila suzukii DS10 00011443
Drosophila ananassae FBgn0101527
Drosophila pseudoobscura FBgn0079341
Drosophila persimilis FBgn0160403
Drosophila virilis FBgn0198530
Drosophila mojavensis FBgn0134350
Drosophila grimshawi FBgn0124104
Drosophila willistoni FBgn0219015
Glossina palpalis GPPI016007
Glossina fuscipes GFUI026266
Glossina austeni GAUT012313
Glossina pallidipes GPAI002110
Glossina morsitans GMOY009808
Glossina brevipalpis GBRI029502
Musca domestica 17012322
Lucilia cuprina FF38 11152
Bactrocera dorsalis 11784318
Bactrocera tryoni JHQJ01000025
Bactrocera cucurbitae 11870608
Ceratitis capitata CCAP010622
Megaselia scalaris MESCA012182
Leptinotarsa decemlineata LDEC011947
Anoplophora glabripennis AGLA013520
Dendroctonus ponderosae YQE 09067
Rhyzobius pseudopulcher
Thanasimus formicarius
Bitoma cylindrica
Meloe violaceus
Tribolium castaneum TC004141
Xylobiops basilaris
Ocypus brunnipes
Lamprohiza splendidula
Aspidytes wrasei
Cybister lateralimarginalis
Cicindela hybrida
Noterus clavicornis
Micromalthus debilis
Lepicerus sp
Cyphon laevipennis
Hydroscapha redfordi
Mengenilla moldrzyki mesp 5503
Apis mellifera GB43565
Apis florea 4234158772
Apis dorsata 51575449
Apis cerana ACSNU07187T0
Bombus impatiens Bimp 2.0
Bombus terrestris 15209022
Melipona quadrifasciata MQUAD 0000a3
Eufriesea mexicana EMEXI 0013fb
Megachile rotundata MROTU 002605
Habropoda laboriosa HLABO 000080
Lasioglossum albipes Lalb 06355
Dufourea novaeangliae DNOVA 00119c
Monomorium pharaonis 4779423924
Wasmannia auropunctata 23054433
Vollenhovia emeryi 19033892
Atta cephalotes ACEP25351
Acromyrmex echinatior AECH16502
Pogonomyrmex barbatus PB13658
Camponotus floridanus CFLO16289
Linepithema humile LH11502
Solenopsis invicta SINV10407
Cerapachys biroi EZA62554.1
Harpegnathos saltator HSAL13490
Polistes dominula 9549
Cephus cinctus CCINC 000ad9
Athalia rosae AROS005926
Orussus abietinus OABI002126
Nasonia vitripennis Nasvi2EG001094
Copidosoma floridanum CFLO018662
Trichogramma pretiosum TPRE000790
Ladona fulva LFUL017649
Calopteryx splendens size95106
Frankliniella occidentalis FOCC001449
Zootermopsis nevadensis Znev 03079
Oncopeltus fasciatus OFAS008841
Halyomorpha halys HHAL004967
Cimex lectularius CLEC007178
Homalodisca vitripennis HVIT019255
Acyrthosiphon pisum ACYPI000323
Gerris buenoi GBUE008298
Ephemera danica EDAN009823
Diptera
Lepidoptera
Trichoptera
Coleoptera
Strepsiptera
Hymenoptera
Psocodea
Thysanoptera
Hemiptera
Blattodea

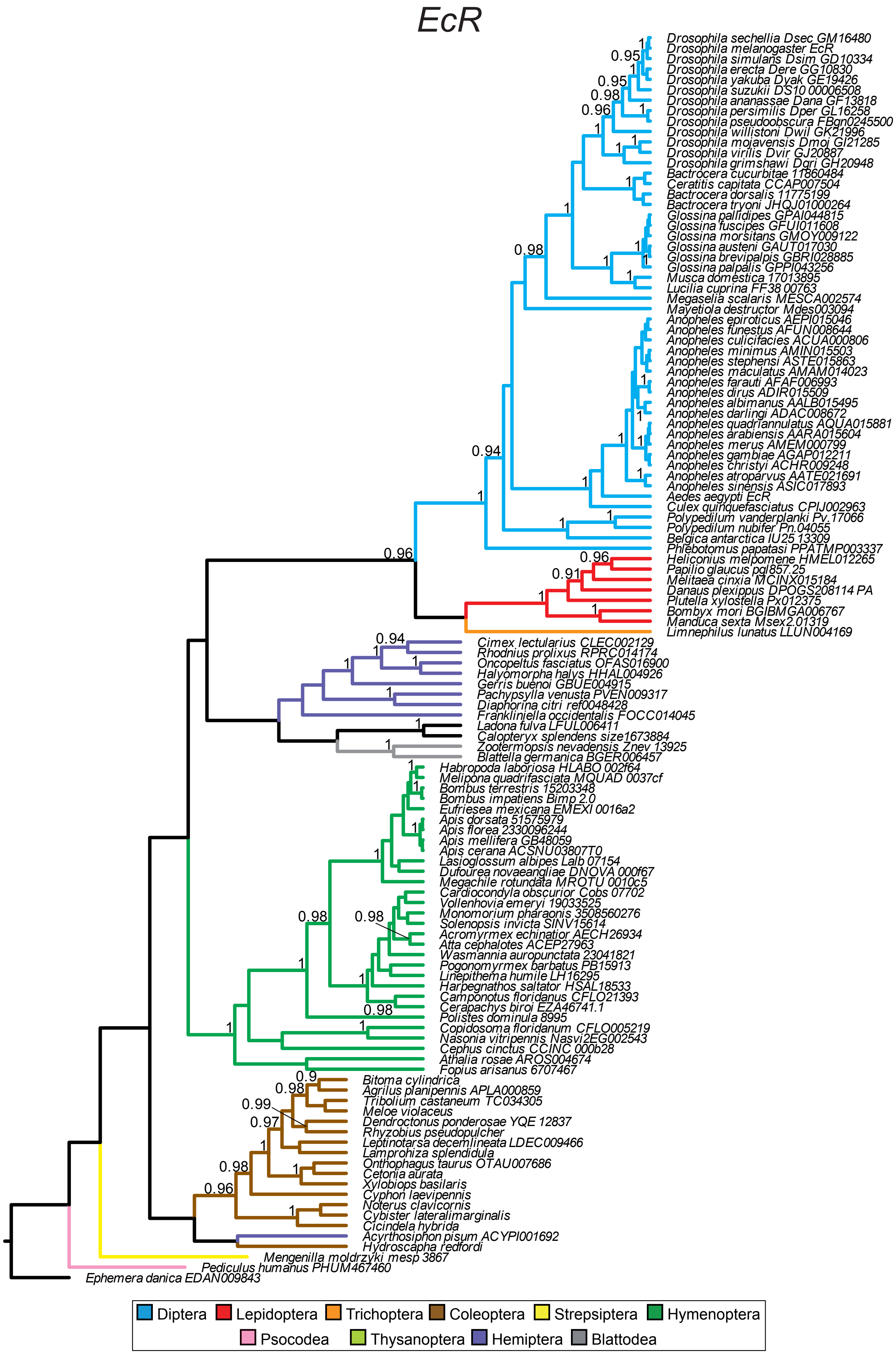

EcR
Drosophila sechellia Dsec GM16480
Drosophila melanogaster EcR
Drosophila simulans Dsim GD10334
Drosophila erecta Dere GG10830
Drosophila yakuba Dyak GE19426
Drosophila suzukii DS10 00006508
Drosophila ananassae Dana GF13818
Drosophila persimilis Dper GL16258
Drosophila pseudoobscura FBgn0245500
Drosophila willistoni Dwil GK21996
Drosophila mojavensis Dmoj GI21285
Drosophila virilis Dvir GJ20887
Drosophila grimshawi Dgri GH20948
Bactrocera cucurbitae 11860484
Ceratitis capitata CCAP007504
Bactrocera dorsalis 11775199
Bactrocera tryoni JHQJ01000264
Glossina pallidipes GPAI044815
Glossina fuscipes GFUI011608
Glossina morsitans GMOY009122
Glossina austeni GAUT017030
Glossina brevipalpis GBRI028885
Glossina palpalis GPPI043256
Musca domestica 17013895
Lucilia cuprina FF38 00763
Megaselia scalaris MESCA002574
Mayetiola destructor Mdes003094
Anopheles epiroticus AEPI015046
Anopheles funestus AFUN008644
Anopheles culicifacies ACUA000806
Anopheles minimus AMIN015503
Anopheles stephensi ASTE015863
Anopheles maculatus AMAM014023
Anopheles farauti AFAF006993
Anopheles dirus ADIR015509
Anopheles albimanus AALB015495
Anopheles darlingi ADAC008672
Anopheles quadriannulatus AQUA015881
Anopheles arabiensis AARA015604
Anopheles merus AMEM000799
Anopheles gambiae AGAP012211
Anopheles christyi ACHR009248
Anopheles atroparvus AATE021691
Anopheles sinensis ASIC017893
Aedes aegypti EcR
Culex quinquefasciatus CPIJ002963
Polypedilum vanderplanki Pv.17066
Polypedilum nubifer Pn.04055
Belgica antarctica IU25 13309
Phlebotomus papatasi PPATMP003337
Heliconius melpomene HMEL012265
Papilio glaucus pgl857.25
Melitaea cinxia MCINX015184
Danaus plexippus DPOGS208114 PA
Plutella xylostella Px012375
Bombyx mori BGIBMGA006767
Manduca sexta Msex2.01319
Limnephilus lunatus LLUN004169
Cimex lectularius CLEC002129
Rhodnius prolixus RPRC014174
Oncopeltus fasciatus OFAS016900
Halyomorpha halys HHAL004926
Gerris buenoi GBUE004915
Pachypsylla venusta PVEN009317
Diaphorina citri ref0048428
Frankliniella occidentalis FOCC014045
Ladona fulva LFUL006411
Calopteryx splendens size1673884
Zootermopsis nevadensis Znev 13925
Blattella germanica BGER006457
Habropoda laboriosa HLABO 002f64
Melipona quadrifasciata MQUAD 0037cf
Bombus terrestris 15203348
Bombus impatiens Bimp 2.0
Eufriesea mexicana EMEXI 0016a2
Apis dorsata 51575979
Apis florea 2330096244
Apis mellifera GB48059
Apis cerana ACSNU03807T0
Lasioglossum albipes Lalb 07154
Dufourea novaeangliae DNOVA 000f67
Megachile rotundata MROTU 0010c5
Cardiocondyla obscurior Cobs 07702
Vollenhovia emeryi 19033525
Monomorium pharaonis 3508560276
Solenopsis invicta SINV15614
Acromyrmex echinatior AECH26934
Atta cephalotes ACEP27963
Wasmannia auropunctata 23041821
Pogonomyrmex barbatus PB15913
Linepithema humile LH16295
Harpegnathos saltator HSAL18533
Camponotus floridanus CFLO21393
Cerapachys biroi EZA46741.1
Polistes dominula 8995
Copidosoma floridanum CFLO005219
Nasonia vitripennis Nasvi2EG002543
Cephus cinctus CCINC 000b28
Athalia rosae AROS004674
Fopius arisanus 6707467
Bitoma cylindrica
Agrilus planipennis APLA000859
Tribolium castaneum TC034305
Meloe violaceus
Dendroctonus ponderosae YQE 12837
Rhyzobius pseudopulcher
Leptinotarsa decemlineata LDEC009466
Lamprohiza splendidula
Onthophagus taurus OTAU007686
Cetonia aurata
Xylobiops basilaris
Cyphon laevipennis
Noterus clavicornis
Cybister lateralimarginalis
Cicindela hybrida
Acyrthosiphon pisum ACYPI001692
Hydroscapha redfordi
Mengenilla moldrzyki mesp 3867
Pediculus humanus PHUM467460
Ephemera danica EDAN009843
Diptera
Lepidoptera
Trichoptera
Coleoptera
Strepsiptera
Hymenoptera
Psocodea
Thysanoptera
Hemiptera
Blattodea

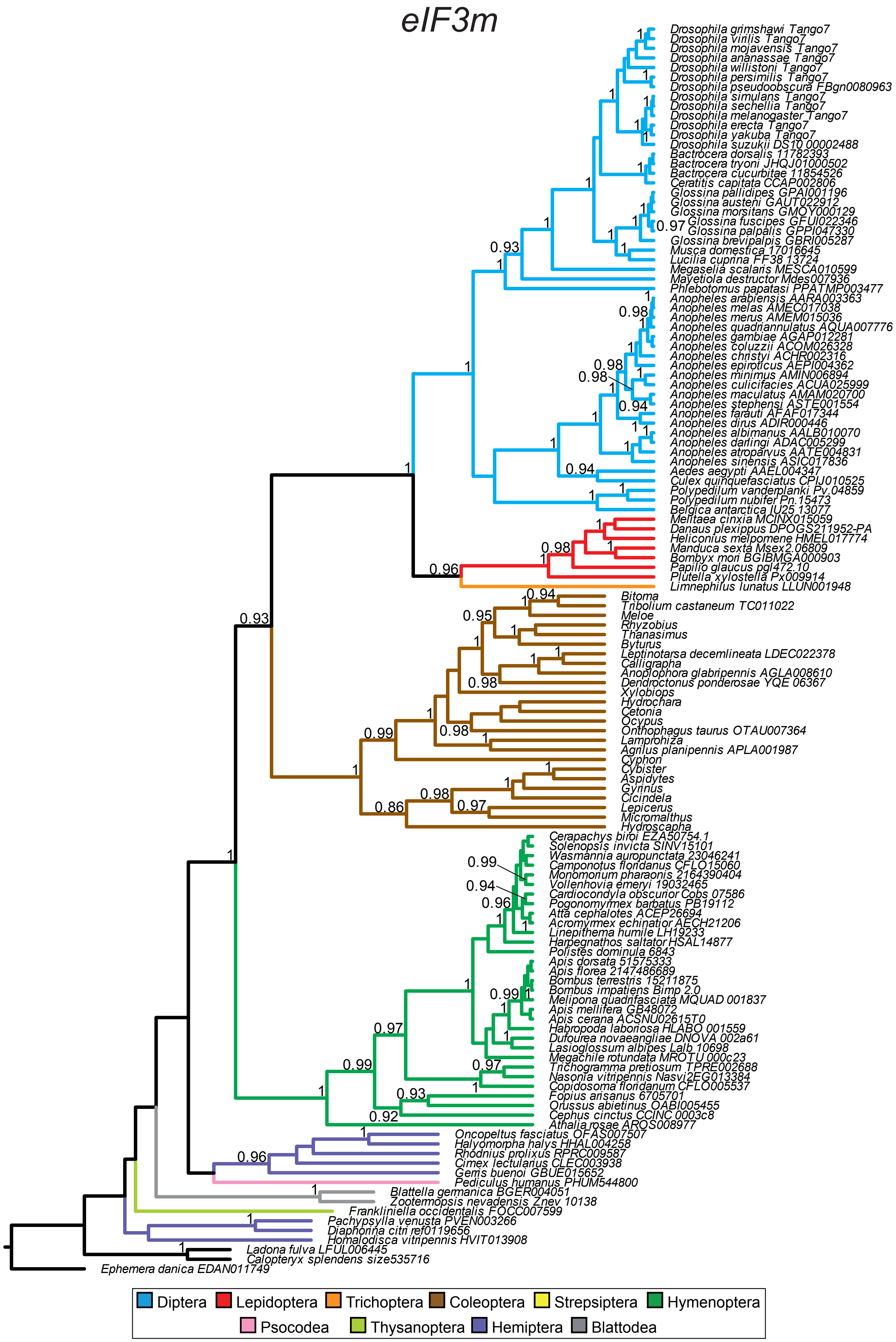

eIF3m
Drosophila grimshawi Tango7
Drosophila virilis Tango7
Drosophila mojavensis Tango7
Drosophila ananassae Tango7
Drosophila willistoni Tango7
Drosophila persimilis Tango7
Drosophila pseudoobscura FBgn0080963
Drosophila simulans Tango7
Drosophila sechellia Tango7
Drosophila melanogaster Tango7
Drosophila erecta Tango7
Drosophila yakuba Tango7
Drosophila suzukii DS10 00002488
Bactrocera dorsalis 11782393
Bactrocera tryoni JHQJ01000502
Bactrocera cucurbitae 11854526
Ceratitis capitata CCAP002806
Glossina pallidipes GPAI001196
Glossina austeni GAUT022912
Glossina morsitans GMOY000129
Glossina fuscipes GFUI022346
Glossina palpalis GPPI047330
Glossina brevipalpis GBRI005287
Musca domestica 17016645
Lucilia cuprina FF38 13724
Megaselia scalaris MESCA010599
Mayetiola destructor Mdes007936
Phlebotomus papatasi PPATMP003477
Anopheles arabiensis AARA003363
Anopheles melas AMEC017038
Anopheles merus AMEM015036
Anopheles quadriannulatus AQUA007776
Anopheles gambiae AGAP012281
Anopheles coluzzii ACOM026328
Anopheles christyi ACHR002316
Anopheles epiroticus AEPI004362
Anopheles minimus AMIN006894
Anopheles culicifacies ACUA025999
Anopheles maculatus AMAM020700
Anopheles stephensi ASTE001554
Anopheles farauti AFAF017344
Anopheles dirus ADIR000446
Anopheles albimanus AALB010070
Anopheles darlingi ADAC005299
Anopheles atroparvus AATE004831
Anopheles sinensis ASIC017836
Aedes aegypti AAEL004347
Culex quinquefasciatus CPIJ010525
Polypedilum vanderplanki Pv.04859
Polypedilum nubifer Pn.15473
Belgica antarctica IU25 13077
Melitaea cinxia MCINX015059
Danaus plexippus DPOGS211952-PA
Heliconius melpomene HMEL017774
Manduca sexta Msex2.06809
Bombyx mori BGIBMGA000903
Papilio glaucus pgl472.10
Plutella xylostella Px009914
Limnephilus lunatus LLUN001948
Bitoma
Tribolium castaneum TC011022
Meloe
Rhyzobius
Thanasimus
Byturus
Leptinotarsa decemlineata LDEC022378
Calligrapha
Anoplophora glabripennis AGLA008610
Dendroctonus ponderosae YQE 06367
Xylobiops
Hydrochara
Cetonia
Ocypus
Onthophagus taurus OTAU007364
Lamprohiza
Agrilus planipennis APLA001987
Cyphon
Cybister
Aspidytes
Gyrinus
Cicindela
Lepicerus
Micromalthus
Hydroscapha
Cerapachys biroi EZA50754.1
Solenopsis invicta SINV15101
Wasmannia auropunctata 23046241
Camponotus floridanus CFLO15060
Monomorium pharaonis 2164390404
Vollenhovia emeryi 19032465
Cardiocondyla obscurior Cobs 07586
Pogonomyrmex barbatus PB19112
Atta cephalotes ACEP26694
Acromyrmex echinatior AECH21206
Linepithema humile LH19233
Harpegnathos saltator HSAL14877
Polistes dominula 6843
Apis dorsata 51575333
Apis florea 2147486689
Bombus terrestris 15211875
Bombus impatiens Bimp 2.0
Melipona quadrifasciata MQUAD 001837
Apis mellifera GB48072
Apis cerana ACSNU02615T0
Habropoda laboriosa HLABO 001559
Dufourea novaeangliae DNOVA 002a61
Lasioglossum albipes Lalb 10698
Megachile rotundata MROTU 000c23
Trichogramma pretiosum TPRE002688
Nasonia vitripennis Nasvi2EG013384
Copidosoma floridanum CFLO005537
Fopius arisanus 6705701
Orussus abietinus OABI005455
Cephus cinctus CCINC 0003c8
Athalia rosae AROS008977
Oncopeltus fasciatus OFAS007507
Halyomorpha halys HHAL004258
Rhodnius prolixus RPRC009587
Cimex lectularius CLEC003938
Gerris buenoi GBUE015652
Pediculus humanus PHUM544800
Blattella germanica BGER004051
Zootermopsis nevadensis Znev 10138
Frankliniella occidentalis FOCC007599
Pachypsylla venusta PVEN003266
Diaphorina citri ref0119656
Homalodisca vitripennis HVIT013908
Ladona fulva LFUL006445
Calopteryx splendens size535716
Ephemera danica EDAN011749
Diptera
Lepidoptera
Trichoptera
Coleoptera
Strepsiptera
Hymenoptera
Psocodea
Thysanoptera
Hemiptera
Blattodea

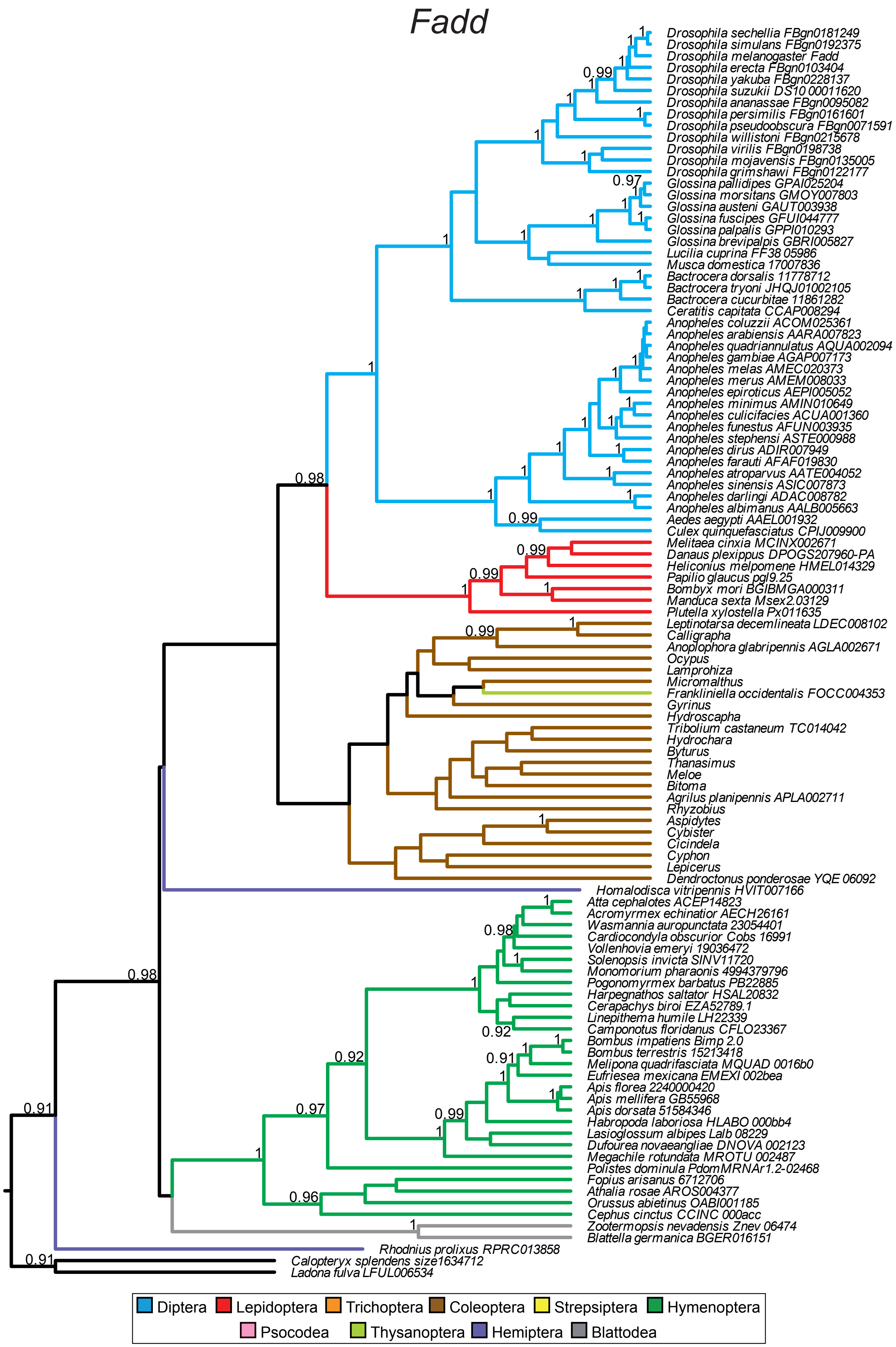
Fadd
Drosophila sechellia FBgn0181249
Drosophila simulans FBgn0192375
Drosophila melanogaster Fadd
Drosophila erecta FBgn0103404
Drosophila yakuba FBgn0228137
Drosophila suzukii DS10 00011620
Drosophila ananassae FBgn0095082
Drosophila persimilis FBgn0161601
Drosophila pseudoobscura FBgn0071591
Drosophila willistoni FBgn0215678
Drosophila virilis FBgn0198738
Drosophila mojavensis FBgn0135005
Drosophila grimshawi FBgn0122177
Glossina pallidipes GPAI025204
Glossina morsitans GMOY007803
Glossina austeni GAUT003938
Glossina fuscipes GFUI044777
Glossina palpalis GPPI010293
Glossina brevipalpis GBRI005827
Lucilia cuprina FF38 05986
Musca domestica 17007836
Bactrocera dorsalis 11778712
Bactrocera tryoni JHQJ01002105
Bactrocera cucurbitae 11861282
Ceratitis capitata CCAP008294
Anopheles coluzzii ACOM025361
Anopheles arabiensis AARA007823
Anopheles quadriannulatus AQUA002094
Anopheles gambiae AGAP007173
Anopheles melas AMEC020373
Anopheles merus AMEM008033
Anopheles epiroticus AEPI005052
Anopheles minimus AMIN010649
Anopheles culicifacies ACUA001360
Anopheles funestus AFUN003935
Anopheles stephensi ASTE000988
Anopheles dirus ADIR007949
Anopheles farauti AFAF019830
Anopheles atroparvus AATE004052
Anopheles sinensis ASIC007873
Anopheles darlingi ADAC008782
Anopheles albimanus AALB005663
Aedes aegypti AAEL001932
Culex quinquefasciatus CPIJ009900
Melitaea cinxia MCINX002671
Danaus plexippus DPOGS207960-PA
Heliconius melpomene HMEL014329
Papilio glaucus pgl9.25
Bombyx mori BGIBMGA000311
Manduca sexta Msex2.03129
Plutella xylostella Px011635
Leptinotarsa decemlineata LDEC008102
Calligrapha
Anoplophora glabripennis AGLA002671
Ocypus
Lamprohiza
Micromalthus
Frankliniella occidentalis FOCC004353
Gyrinus
Hydroscapha
Tribolium castaneum TC014042
Hydrochara
Byturus
Thanasimus
Meloe
Bitoma
Agrilus planipennis APLA002711
Rhyzobius
Aspidytes
Cybister
Cicindela
Cyphon
Lepicerus
Dendroctonus ponderosae YQE 06092
Homalodisca vitripennis HVIT007166
Atta cephalotes ACEP14823
Acromyrmex echinatior AECH26161
Wasmannia auropunctata 23054401
Cardiocondyla obscurior Cobs 16991
Vollenhovia emeryi 19036472
Solenopsis invicta SINV11720
Monomorium pharaonis 4994379796
Pogonomyrmex barbatus PB22885
Harpegnathos saltator HSAL20832
Cerapachys biroi EZA52789.1
Linepithema humile LH22339
Camponotus floridanus CFLO23367
Bombus impatiens Bimp 2.0
Bombus terrestris 15213418
Melipona quadrifasciata MQUAD 0016b0
Eufriesea mexicana EMEXI 002bea
Apis florea 2240000420
Apis mellifera GB55968
Apis dorsata 51584346
Habropoda laboriosa HLABO 000bb4
Lasioglossum albipes Lalb 08229
Dufourea novaeangliae DNOVA 002123
Megachile rotundata MROTU 002487
Polistes dominula PdomMRNAr1.2-02468
Fopius arisanus 6712706
Athalia rosae AROS004377
Orussus abietinus OABI001185
Cephus cinctus CCINC 000acc
Zootermopsis nevadensis Znev 06474
Blattella germanica BGER016151
Rhodnius prolixus RPRC013858
Calopteryx splendens size1634712
Ladona fulva LFUL006534
Diptera
Lepidoptera
Trichoptera
Coleoptera
Strepsiptera
Hymenoptera
Psocodea
Thysanoptera
Hemiptera
Blattodea

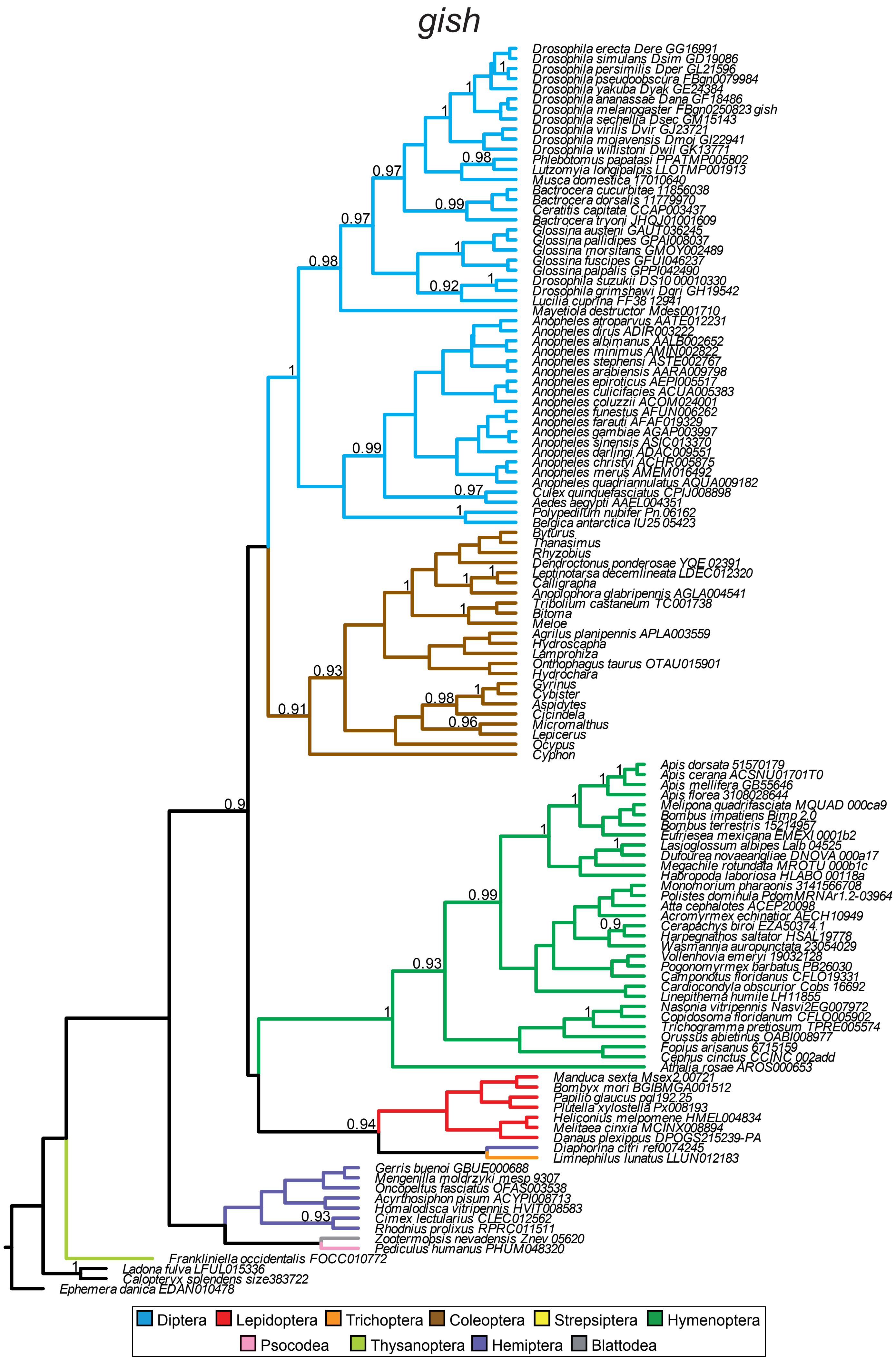

gish
Drosophila erecta Dere GG16991
Drosophila simulans Dsim GD19086
Drosophila persimilis Dper GL21596
Drosophila pseudoobscura FBgn0079984
Drosophila yakuba Dyak GE24384
Drosophila ananassae Dana GF18486
Drosophila melanogaster FBgn0250823 gish
Drosophila sechellia Dsec GM15143
Drosophila virilis Dvir GJ23721
Drosophila mojavensis Dmoj GI22941
Drosophila willistoni Dwil GK13771
Phlebotomus papatasi PPATMP005802
Lutzomyia longipalpis LLOTMP001913
Musca domestica 17010640
Bactrocera cucurbitae 11856038
Bactrocera dorsalis 11779970
Ceratitis capitata CCAP003437
Bactrocera tryoni JHQJ01001609
Glossina austeni GAUT036245
Glossina pallidipes GPAI008037
Glossina morsitans GMOY002489
Glossina fuscipes GFUI046237
Glossina palpalis GPPI042490
Drosophila suzukii DS10 00010330
Drosophila grimshawi Dgri GH19542
Lucilia cuprina FF38 12941
Mayetiola destructor Mdes001710
Anopheles atroparvus AATE012231
Anopheles dirus ADIR003222
Anopheles albimanus AALB002652
Anopheles minimus AMIN002822
Anopheles stephensi ASTE002767
Anopheles arabiensis AARA009798
Anopheles epiroticus AEPI005517
Anopheles culicifacies ACUA005383
Anopheles coluzzii ACOM024001
Anopheles funestus AFUN006262
Anopheles farauti AFAF019329
Anopheles gambiae AGAP003997
Anopheles sinensis ASIC013370
Anopheles darlingi ADAC009551
Anopheles christyi ACHR005875
Anopheles merus AMEM016492
Anopheles quadriannulatus AQUA009182
Culex quinquefasciatus CPIJ008898
Aedes aegypti AAEL004351
Polypedilum nubifer Pn.06162
Belgica antarctica IU25 05423
Byturus
Thanasimus
Rhyzobius
Dendroctonus ponderosae YQE 02391
Leptinotarsa decemlineata LDEC012320
Calligrapha
Anoplophora glabripennis AGLA004541
Tribolium castaneum TC001738
Bitoma
Meloe
Agrilus planipennis APLA003559
Hydroscapha
Lamprohiza
Onthophagus taurus OTAU015901
Hydrochara
Gyrinus
Cybister
Aspidytes
Cicindela
Micromalthus
Lepicerus
Ocypus
Cyphon
Apis dorsata 51570179
Apis cerana ACSNU01701T0
Apis mellifera GB55646
Apis florea 3108028644
Melipona quadrifasciata MQUAD 000ca9
Bombus impatiens Bimp 2.0
Bombus terrestris 15214957
Eufriesea mexicana EMEXI 0001b2
Lasioglossum albipes Lalb 04525
Dufourea novaeangliae DNOVA 000a17
Megachile rotundata MROTU 000b1c
Habropoda laboriosa HLABO 00118a
Monomorium pharaonis 3141566708
Polistes dominula PdomMRNAr1.2-03964
Atta cephalotes ACEP20098
Acromyrmex echinatior AECH10949
Cerapachys biroi EZA50374.1
Harpegnathos saltator HSAL19778
Wasmannia auropunctata 23054029
Vollenhovia emeryi 19032128
Pogonomyrmex barbatus PB26030
Camponotus floridanus CFLO19331
Cardiocondyla obscurior Cobs 16692
Linepithema humile LH11855
Nasonia vitripennis Nasvi2EG007972
Copidosoma floridanum CFLO005902
Trichogramma pretiosum TPRE005574
Orussus abietinus OABI008977
Fopius arisanus 6715159
Cephus cinctus CCINC 002add
Athalia rosae AROS000653
Manduca sexta Msex2.00721
Bombyx mori BGIBMGA001512
Papilio glaucus pgl192.25
Plutella xylostella Px008193
Heliconius melpomene HMEL004834
Melitaea cinxia MCINX008894
Danaus plexippus DPOGS215239-PA
Diaphorina citri ref0074245
Limnephilus lunatus LLUN012183
Gerris buenoi GBUE000688
Mengenilla moldrzyki mesp 9307
Oncopeltus fasciatus OFAS003538
Acyrthosiphon pisum ACYPI008713
Homalodisca vitripennis HVIT008583
Cimex lectularius CLEC012562
Rhodnius prolixus RPRC011511
Zootermopsis nevadensis Znev 05620
Pediculus humanus PHUM048320
Frankliniella occidentalis FOCC010772
Ladona fulva LFUL015336
Calopteryx splendens size383722
Ephemera danica EDAN010478
1
1
1
0.98
0.97
0.99
0.97
1
0.92
0.98
1
1
1
0.99
0.97
1
1
1
1
1
0.93
0.98
1
0.96
0.91
0.9
1
1
1
1
1
0.99
0.9
0.93
1
1
0.94
0.93
1
Diptera
Lepidoptera
Trichoptera
Coleoptera
Strepsiptera
Hymenoptera
Psocodea
Thysanoptera
Hemiptera
Blattodea

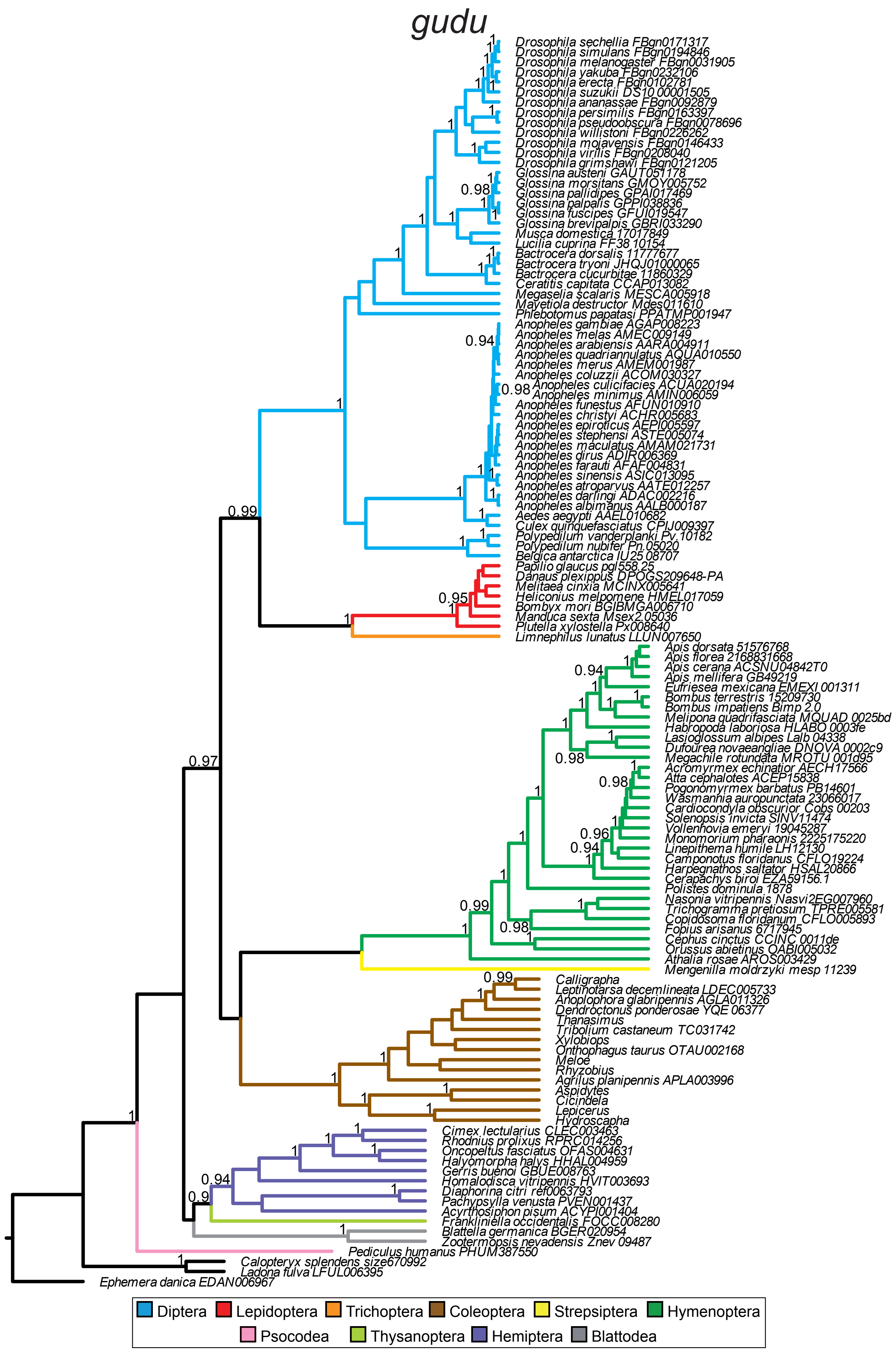
gudu
Drosophila sechellia FBgn0171317
Drosophila simulans FBgn0194846
Drosophila melanogaster FBgn0031905
Drosophila yakuba FBgn0232106
Drosophila erecta FBgn0102781
Drosophila suzukii DS10 00001505
Drosophila ananassae FBgn0092879
Drosophila persimilis FBgn0163397
Drosophila pseudoobscura FBgn0078696
Drosophila willistoni FBgn0226262
Drosophila mojavensis FBgn0146433
Drosophila virilis FBgn0208040
Drosophila grimshawi FBgn0121205
Glossina austeni GAUT051178
Glossina morsitans GMOY005752
Glossina pallidipes GPAI017469
Glossina palpalis GPPI038836
Glossina fuscipes GFUI019547
Glossina brevipalpis GBRI033290
Musca domestica 17017849
Lucilia cuprina FF38 10154
Bactrocera dorsalis 11777677
Bactrocera tryoni JHQJ01000065
Bactrocera cucurbitae 11860329
Ceratitis capitata CCAP013082
Megaselia scalaris MESCA005918
Mayetiola destructor Mdes011610
Phlebotomus papatasi PPATMP001947
Anopheles gambiae AGAP008223
Anopheles melas AMEC009149
Anopheles arabiensis AARA004911
Anopheles quadriannulatus AQUA010550
Anopheles merus AMEM001987
Anopheles coluzzii ACOM030327
Anopheles culicifacies ACUA020194
Anopheles minimus AMIN006059
Anopheles funestus AFUN010910
Anopheles christyi ACHR005683
Anopheles epiroticus AEPI005597
Anopheles stephensi ASTE005074
Anopheles maculatus AMAM021731
Anopheles dirus ADIR006369
Anopheles farauti AFAF004831
Anopheles sinensis ASIC013095
Anopheles atroparvus AATE012257
Anopheles darlingi ADAC002216
Anopheles albimanus AALB000187
Aedes aegypti AAEL010682
Culex quinquefasciatus CPIJ009397
Polypedilum vanderplanki Pv.10182
Polypedilum nubifer Pn.05020
Belgica antarctica IU25 08707
Papilio glaucus pgl558.25
Danaus plexippus DPOGS209648-PA
Melitaea cinxia MCINX005641
Heliconius melpomene HMEL017059
Bombyx mori BGIBMGA006710
Manduca sexta Msex2.05036
Plutella xylostella Px008640
Limnephilus lunatus LLUN007650
Apis dorsata 51576768
Apis florea 2168831668
Apis cerana ACSNU04842T0
Apis mellifera GB49219
Eufriesea mexicana EMEXI 001311
Bombus terrestris 15209730
Bombus impatiens Bimp 2.0
Melipona quadrifasciata MQUAD 0025bd
Habropoda laboriosa HLABO 0003fe
Lasioglossum albipes Lalb 04338
Dufourea novaeangliae DNOVA 0002c9
Megachile rotundata MROTU 001d95
Acromyrmex echinatior AECH17566
Atta cephalotes ACEP15838
Pogonomyrmex barbatus PB14601
Wasmannia auropunctata 23066017
Cardiocondyla obscurior Cobs 00203
Solenopsis invicta SINV11474
Vollenhovia emeryi 19045287
Monomorium pharaonis 2225175220
Linepithema humile LH12130
Camponotus floridanus CFLO19224
Harpegnathos saltator HSAL20866
Cerapachys biroi EZA59156.1
Polistes dominula 1878
Nasonia vitripennis Nasvi2EG007960
Trichogramma pretiosum TPRE005581
Copidosoma floridanum CFLO005893
Fopius arisanus 6717945
Cephus cinctus CCINC 0011de
Orussus abietinus OABI005032
Athalia rosae AROS003429
Mengenilla moldrzyki mesp 11239
Calligrapha
Leptinotarsa decemlineata LDEC005733
Anoplophora glabripennis AGLA011326
Dendroctonus ponderosae YQE 06377
Thanasimus
Tribolium castaneum TC031742
Xylobiops
Onthophagus taurus OTAU002168
Meloe
Rhyzobius
Agrilus planipennis APLA003996
Aspidytes
Cicindela
Lepicerus
Hydroscapha
Cimex lectularius CLEC003463
Rhodnius prolixus RPRC014256
Oncopeltus fasciatus OFAS004631
Halyomorpha halys HHAL004959
Gerris buenoi GBUE008763
Homalodisca vitripennis HVIT003693
Diaphorina citri ref0063793
Pachypsylla venusta PVEN001437
Acyrthosiphon pisum ACYPI001404
Frankliniella occidentalis FOCC008280
Blattella germanica BGER020954
Zootermopsis nevadensis Znev 09487
Pediculus humanus PHUM387550
Calopteryx splendens size670992
Ladona fulva LFUL006395
Ephemera danica EDAN006967
Diptera
Lepidoptera
Trichoptera
Coleoptera
Strepsiptera
Hymenoptera
Psocodea
Thysanoptera
Hemiptera
Blattodea

heph

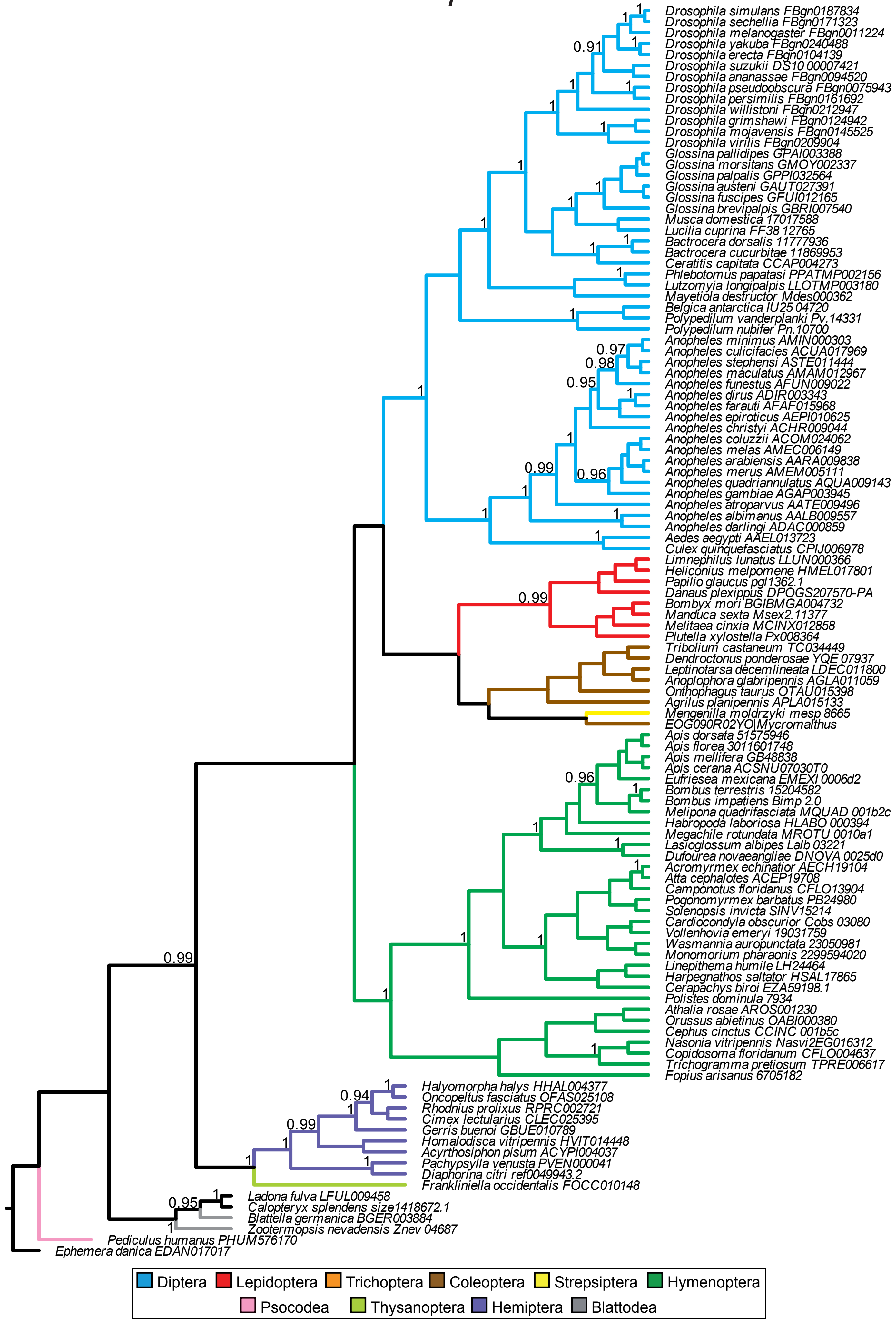
Drosophila simulans FBgn0187834
Drosophila sechellia FBgn0171323
Drosophila melanogaster FBgn0011224
Drosophila yakuba FBgn0240488
Drosophila erecta FBgn0104139
Drosophila suzukii DS10 00007421
Drosophila ananassae FBgn0094520
Drosophila pseudoobscura FBgn0075943
Drosophila persimilis FBgn0161692
Drosophila willistoni FBgn0212947
Drosophila grimshawi FBgn0124942
Drosophila mojavensis FBgn0145525
Drosophila virilis FBgn0209904
Glossina pallidipes GPAI003388
Glossina morsitans GMOY002337
Glossina palpalis GPPI032564
Glossina austeni GAUT027391
Glossina fuscipes GFUI012165
Glossina brevipalpis GBRI007540
Musca domestica 17017588
Lucilia cuprina FF38 12765
Bactrocera dorsalis 11777936
Bactrocera cucurbitae 11869953
Ceratitis capitata CCAP004273
Phlebotomus papatasi PPATMP002156
Lutzomyia longipalpis LLOTMP003180
Mayetiola destructor Mdes000362
Belgica antarctica IU25 04720
Polypedilum vanderplanki Pv.14331
Polypedilum nubifer Pn.10700
Anopheles minimus AMIN000303
Anopheles culicifacies ACUA017969
Anopheles stephensi ASTE011444
Anopheles maculatus AMAM012967
Anopheles funestus AFUN009022
Anopheles dirus ADIR003343
Anopheles farauti AFAF015968
Anopheles epiroticus AEPI010625
Anopheles christyi ACHR009044
Anopheles coluzzii ACOM024062
Anopheles melas AMEC006149
Anopheles arabiensis AARA009838
Anopheles merus AMEM005111
Anopheles quadriannulatus AQUA009143
Anopheles gambiae AGAP003945
Anopheles atroparvus AATE009496
Anopheles albimanus AALB009557
Anopheles darlingi ADAC000859
Aedes aegypti AAEL013723
Culex quinquefasciatus CPIJ006978
Limnephilus lunatus LLUN000366
Heliconius melpomene HMEL017801
Papilio glaucus pgl1362.1
Danaus plexippus DPOGS207570-PA
Bombyx mori BGIBMGA004732
Manduca sexta Msex2.11377
Melitaea cinxia MCINX012858
Plutella xylostella Px008364
Tribolium castaneum TC034449
Dendroctonus ponderosae YQE 07937
Leptinotarsa decemlineata LDEC011800
Anoplophora glabripennis AGLA011059
Onthophagus taurus OTAU015398
Agrilus planipennis APLA015133
Mengenilla moldrzyki mesp 8665
EOG090R02YO|Mycromalthus
Apis dorsata 51575946
Apis florea 3011601748
Apis mellifera GB48838
Apis cerana ACSNU07030T0
Eufriesea mexicana EMEXI 0006d2
Bombus terrestris 15204582
Bombus impatiens Bimp 2.0
Melipona quadrifasciata MQUAD 001b2c
Habropoda laboriosa HLABO 000394
Megachile rotundata MROTU 0010a1
Lasioglossum albipes Lalb 03221
Dufourea novaeangliae DNOVA 0025d0
Acromyrmex echinatior AECH19104
Atta cephalotes ACEP19708
Camponotus floridanus CFLO13904
Pogonomyrmex barbatus PB24980
Solenopsis invicta SINV15214
Cardiocondyla obscurior Cobs 03080
Vollenhovia emeryi 19031759
Wasmannia auropunctata 23050981
Monomorium pharaonis 2299594020
Linepithema humile LH24464
Harpegnathos saltator HSAL17865
Cerapachys biroi EZA59198.1
Polistes dominula 7934
Athalia rosae AROS001230
Orussus abietinus OABI000380
Cephus cinctus CCINC 001b5c
Nasonia vitripennis Nasvi2EG016312
Copidosoma floridanum CFLO004637
Trichogramma pretiosum TPRE006617
Fopius arisanus 6705182
Halyomorpha halys HHAL004377
Oncopeltus fasciatus OFAS025108
Rhodnius prolixus RPRC002721
Cimex lectularius CLEC025395
Gerris buenoi GBUE010789
Homalodisca vitripennis HVIT014448
Acyrthosiphon pisum ACYPI004037
Pachypsylla venusta PVEN000041
Diaphorina citri ref0049943.2
Frankliniella occidentalis FOCC010148
Ladona fulva LFUL009458
Calopteryx splendens size1418672.1
Blattella germanica BGER003884
Zootermopsis nevadensis Znev 04687
Pediculus humanus PHUM576170
Ephemera danica EDAN017017
Diptera
Lepidoptera
Trichoptera
Coleoptera
Strepsiptera
Hymenoptera
Psocodea
Thysanoptera
Hemiptera
Blattodea

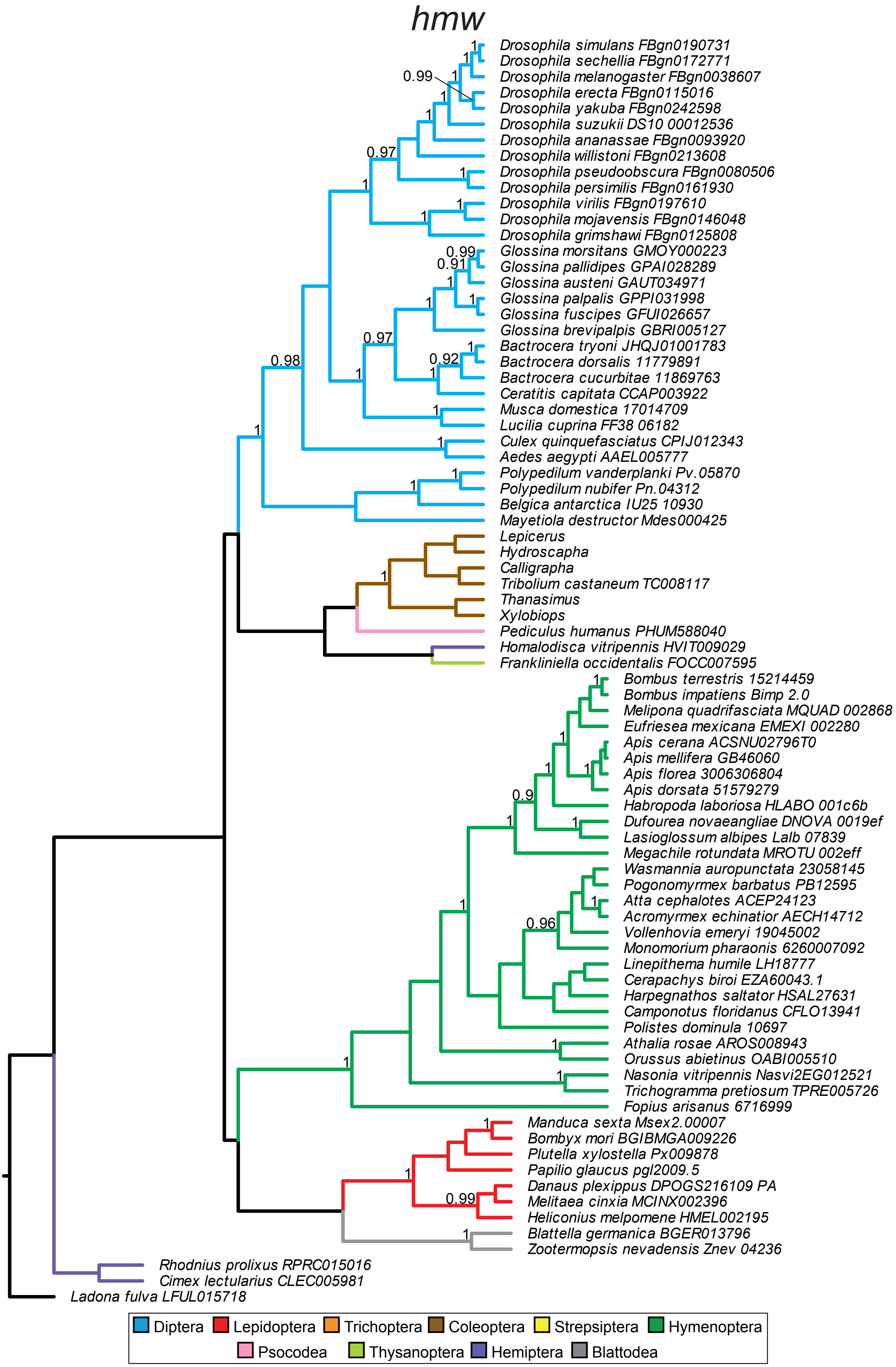

hmw
Drosophila simulans FBgn0190731
Drosophila sechellia FBgn0172771
Drosophila melanogaster FBgn0038607
Drosophila erecta FBgn0115016
Drosophila yakuba FBgn0242598
Drosophila suzukii DS10 00012536
Drosophila ananassae FBgn0093920
Drosophila willistoni FBgn0213608
Drosophila pseudoobscura FBgn0080506
Drosophila persimilis FBgn0161930
Drosophila virilis FBgn0197610
Drosophila mojavensis FBgn0146048
Drosophila grimshawi FBgn0125808
Glossina morsitans GMOY000223
Glossina pallidipes GPAI028289
Glossina austeni GAUT034971
Glossina palpalis GPPI031998
Glossina fuscipes GFUI026657
Glossina brevipalpis GBRI005127
Bactrocera tryoni JHQJ01001783
Bactrocera dorsalis 11779891
Bactrocera cucurbitae 11869763
Ceratitis capitata CCAP003922
Musca domestica 17014709
Lucilia cuprina FF38 06182
Culex quinquefasciatus CPIJ012343
Aedes aegypti AAEL005777
Polypedilum vanderplanki Pv.05870
Polypedilum nubifer Pn.04312
Belgica antarctica IU25 10930
Mayetiola destructor Mdes000425
Lepicerus
Hydroscapha
Calligrapha
Tribolium castaneum TC008117
Thanasimus
Xylobiops
Pediculus humanus PHUM588040
Homalodisca vitripennis HVIT009029
Frankliniella occidentalis FOCC007595
Bombus terrestris 15214459
Bombus impatiens Bimp 2.0
Melipona quadrifasciata MQUAD 002868
Eufriesea mexicana EMEXI 002280
Apis cerana ACSNU02796T0
Apis mellifera GB46060
Apis florea 3006306804
Apis dorsata 51579279
Habropoda laboriosa HLABO 001c6b
Dufourea novaeangliae DNOVA 0019ef
Lasioglossum albipes Lalb 07839
Megachile rotundata MROTU 002eff
Wasmannia auropunctata 23058145
Pogonomyrmex barbatus PB12595
Atta cephalotes ACEP24123
Acromyrmex echinatior AECH14712
Vollenhovia emeryi 19045002
Monomorium pharaonis 6260007092
Linepithema humile LH18777
Cerapachys biroi EZA60043.1
Harpegnathos saltator HSAL27631
Camponotus floridanus CFLO13941
Polistes dominula 10697
Athalia rosae AROS008943
Orussus abietinus OABI005510
Nasonia vitripennis Nasvi2EG012521
Trichogramma pretiosum TPRE005726
Fopius arisanus 6716999
Manduca sexta Msex2.00007
Bombyx mori BGIBMGA009226
Plutella xylostella Px009878
Papilio glaucus pgl2009.5
Danaus plexippus DPOGS216109 PA
Melitaea cinxia MCINX002396
Heliconius melpomene HMEL002195
Blattella germanica BGER013796
Zootermopsis nevadensis Znev 04236
Rhodnius prolixus RPRC015016
Cimex lectularius CLEC005981
Ladona fulva LFUL015718
Diptera
Lepidoptera
Trichoptera
Coleoptera
Strepsiptera
Hymenoptera
Psocodea
Thysanoptera
Hemiptera
Blattodea

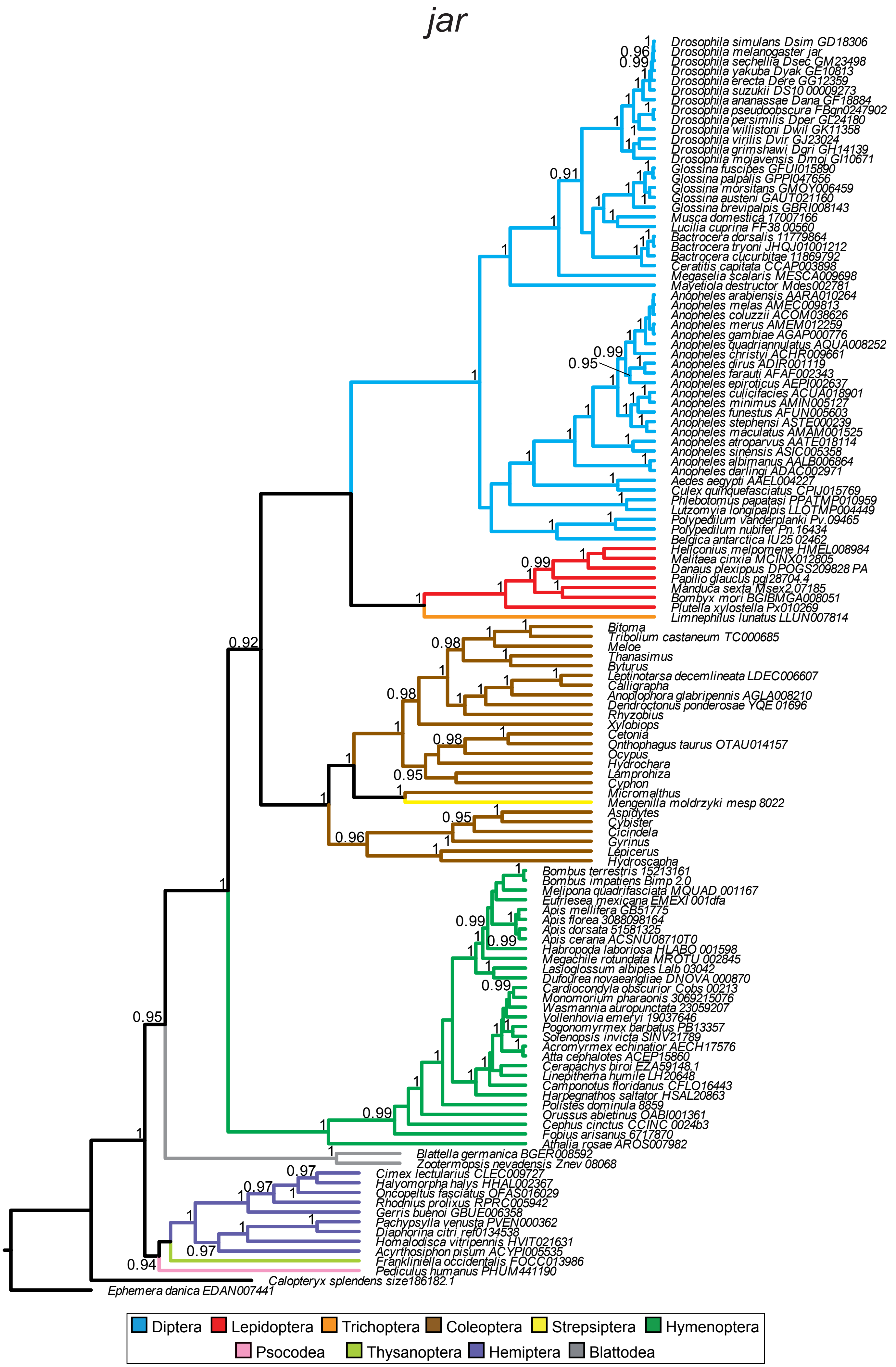
jar
Drosophila simulans Dsim GD18306
Drosophila melanogaster jar
Drosophila sechellia Dsec GM23498
Drosophila yakuba Dyak GE10813
Drosophila erecta Dere GG12359
Drosophila suzukii DS10 00009273
Drosophila ananassae Dana GF18884
Drosophila pseudoobscura FBgn0247902
Drosophila persimilis Dper GL24180
Drosophila willistoni Dwil GK11358
Drosophila virilis Dvir GJ23024
Drosophila grimshawi Dgri GH14139
Drosophila mojavensis Dmoj GI10671
Glossina fuscipes GFUI015890
Glossina palpalis GPPI047656
Glossina morsitans GMOY006459
Glossina austeni GAUT021160
Glossina brevipalpis GBRI008143
Musca domestica 17007166
Lucilia cuprina FF38 00560
Bactrocera dorsalis 11779864
Bactrocera tryoni JHQJ01001212
Bactrocera cucurbitae 11869792
Ceratitis capitata CCAP003898
Megaselia scalaris MESCA009698
Mayetiola destructor Mdes002781
Anopheles arabiensis AARA010264
Anopheles melas AMEC009813
Anopheles coluzzii ACOM038626
Anopheles merus AMEM012259
Anopheles gambiae AGAP000776
Anopheles quadriannulatus AQUA008252
Anopheles christyi ACHR009661
Anopheles dirus ADIR001119
Anopheles farauti AFAF002343
Anopheles epiroticus AEPI002637
Anopheles culicifacies ACUA018901
Anopheles minimus AMIN005127
Anopheles funestus AFUN005603
Anopheles stephensi ASTE000239
Anopheles maculatus AMAM001525
Anopheles atroparvus AATE018114
Anopheles sinensis ASIC005358
Anopheles albimanus AALB006864
Anopheles darlingi ADAC002971
Aedes aegypti AAEL004227
Culex quinquefasciatus CPIJ015769
Phlebotomus papatasi PPATMP010959
Lutzomyia longipalpis LLOTMP004449
Polypedilum vanderplanki Pv.09465
Polypedilum nubifer Pn.16434
Belgica antarctica IU25 02462
Heliconius melpomene HMEL008984
Melitaea cinxia MCINX012805
Danaus plexippus DPOGS209828 PA
Papilio glaucus pgl28704.4
Manduca sexta Msex2.07185
Bombyx mori BGIBMGA008051
Plutella xylostella Px010269
Limnephilus lunatus LLUN007814
Bitoma
Tribolium castaneum TC000685
Meloe
Thanasimus
Byturus
Leptinotarsa decemlineata LDEC006607
Calligrapha
Anoplophora glabripennis AGLA008210
Dendroctonus ponderosae YQE 01696
Rhyzobius
Xylobiops
Cetonia
Onthophagus taurus OTAU014157
Ocypus
Hydrochara
Lamprohiza
Cyphon
Micromalthus
Mengenilla moldrzyki mesp 8022
Aspidytes
Cybister
Cicindela
Gyrinus
Lepicerus
Hydroscapha
Bombus terrestris 15213161
Bombus impatiens Bimp 2.0
Melipona quadrifasciata MQUAD 001167
Eufriesea mexicana EMEXI 001dfa
Apis mellifera GB51775
Apis florea 3088098164
Apis dorsata 51581325
Apis cerana ACSNU08710T0
Habropoda laboriosa HLABO 001598
Megachile rotundata MROTU 002845
Lasioglossum albipes Lalb 03042
Dufourea novaeangliae DNOVA 000870
Cardiocondyla obscurior Cobs 00213
Monomorium pharaonis 3069215076
Wasmannia auropunctata 23059207
Vollenhovia emeryi 19037646
Pogonomyrmex barbatus PB13357
Solenopsis invicta SINV21789
Acromyrmex echinatior AECH17576
Atta cephalotes ACEP15860
Cerapachys biroi EZA59148.1
Linepithema humile LH20648
Camponotus floridanus CFLO16443
Harpegnathos saltator HSAL20863
Polistes dominula 8859
Orussus abietinus OABI001361
Cephus cinctus CCINC 0024b3
Fopius arisanus 6717870
Athalia rosae AROS007982
Blattella germanica BGER008592
Zootermopsis nevadensis Znev 08068
Cimex lectularius CLEC009727
Halyomorpha halys HHAL002367
Oncopeltus fasciatus OFAS016029
Rhodnius prolixus RPRC005942
Gerris buenoi GBUE006358
Pachypsylla venusta PVEN000362
Diaphorina citri ref0134538
Homalodisca vitripennis HVIT021631
Acyrthosiphon pisum ACYPI005535
Frankliniella occidentalis FOCC013986
Pediculus humanus PHUM441190
Calopteryx splendens size186182.1
Ephemera danica EDAN007441
Diptera
Lepidoptera
Trichoptera
Coleoptera
Strepsiptera
Hymenoptera
Psocodea
Thysanoptera
Hemiptera
Blattodea

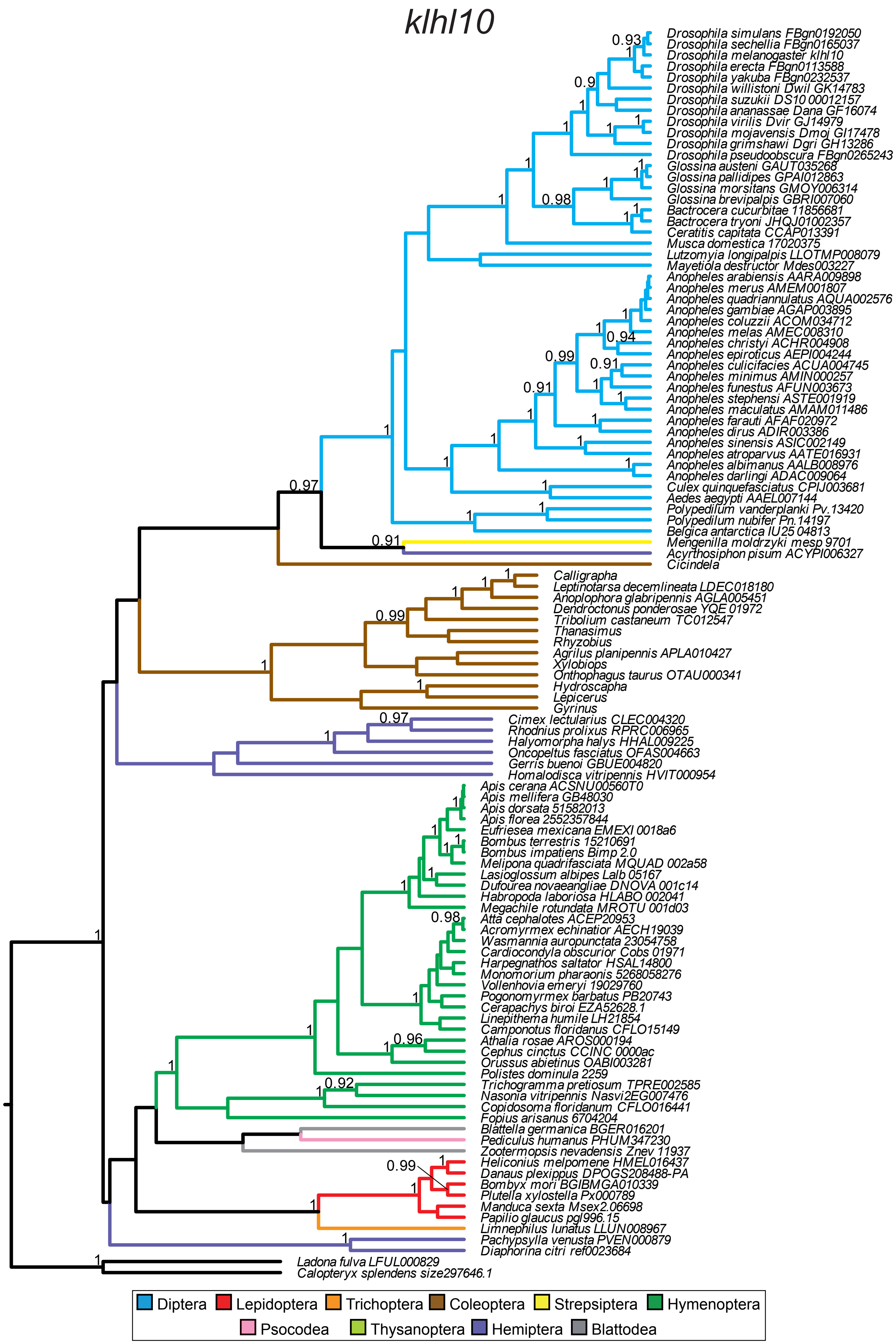
klhl10
Drosophila simulans FBgn0192050
Drosophila sechellia FBgn0165037
Drosophila melanogaster klhl10
Drosophila erecta FBgn0113588
Drosophila yakuba FBgn0232537
Drosophila willistoni Dwil GK14783
Drosophila suzukii DS10 00012157
Drosophila ananassae Dana GF16074
Drosophila virilis Dvir GJ14979
Drosophila mojavensis Dmoj GI17478
Drosophila grimshawi Dgri GH13286
Drosophila pseudoobscura FBgn0265243
Glossina austeni GAUT035268
Glossina pallidipes GPAI012863
Glossina morsitans GMOY006314
Glossina brevipalpis GBRI007060
Bactrocera cucurbitae 11856681
Bactrocera tryoni JHQJ01002357
Ceratitis capitata CCAP013391
Musca domestica 17020375
Lutzomyia longipalpis LLOTMP008079
Mayetiola destructor Mdes003227
Anopheles arabiensis AARA009898
Anopheles merus AMEM001807
Anopheles quadriannulatus AQUA002576
Anopheles gambiae AGAP003895
Anopheles coluzzii ACOM034712
Anopheles melas AMEC008310
Anopheles christyi ACHR004908
Anopheles epiroticus AEPI004244
Anopheles culicifacies ACUA004745
Anopheles minimus AMIN000257
Anopheles funestus AFUN003673
Anopheles stephensi ASTE001919
Anopheles maculatus AMAM011486
Anopheles farauti AFAF020972
Anopheles dirus ADIR003386
Anopheles sinensis ASIC002149
Anopheles atroparvus AATE016931
Anopheles albimanus AALB008976
Anopheles darlingi ADAC009064
Culex quinquefasciatus CPIJ003681
Aedes aegypti AAEL007144
Polypedilum vanderplanki Pv.13420
Polypedilum nubifer Pn.14197
Belgica antarctica IU25 04813
Mengenilla moldrzyki mesp 9701
Acyrthosiphon pisum ACYPI006327
Cicindela
Calligrapha
Leptinotarsa decemlineata LDEC018180
Anoplophora glabripennis AGLA005451
Dendroctonus ponderosae YQE 01972
Tribolium castaneum TC012547
Thanasimus
Rhyzobius
Agrilus planipennis APLA010427
Xylobiops
Onthophagus taurus OTAU000341
Hydroscapha
Lepicerus
Gyrinus
Cimex lectularius CLEC004320
Rhodnius prolixus RPRC006965
Halyomorpha halys HHAL009225
Oncopeltus fasciatus OFAS004663
Gerris buenoi GBUE004820
Homalodisca vitripennis HVIT000954
Apis cerana ACSNU00560T0
Apis mellifera GB48030
Apis dorsata 51582013
Apis florea 2552357844
Eufriesea mexicana EMEXI 0018a6
Bombus terrestris 15210691
Bombus impatiens Bimp 2.0
Melipona quadrifasciata MQUAD 002a58
Lasioglossum albipes Lalb 05167
Dufourea novaeangliae DNOVA 001c14
Habropoda laboriosa HLABO 002041
Megachile rotundata MROTU 001d03
Atta cephalotes ACEP20953
Acromyrmex echinatior AECH19039
Wasmannia auropunctata 23054758
Cardiocondyla obscurior Cobs 01971
Harpegnathos saltator HSAL14800
Monomorium pharaonis 5268058276
Vollenhovia emeryi 19029760
Pogonomyrmex barbatus PB20743
Cerapachys biroi EZA52628.1
Linepithema humile LH21854
Camponotus floridanus CFLO15149
Athalia rosae AROS000194
Cephus cinctus CCINC 0000ac
Orussus abietinus OABI003281
Polistes dominula 2259
Trichogramma pretiosum TPRE002585
Nasonia vitripennis Nasvi2EG007476
Copidosoma floridanum CFLO016441
Fopius arisanus 6704204
Blattella germanica BGER016201
Pediculus humanus PHUM347230
Zootermopsis nevadensis Znev 11937
Heliconius melpomene HMEL016437
Danaus plexippus DPOGS208488-PA
Bombyx mori BGIBMGA010339
Plutella xylostella Px000789
Manduca sexta Msex2.06698
Papilio glaucus pgl996.15
Limnephilus lunatus LLUN008967
Pachypsylla venusta PVEN000879
Diaphorina citri ref0023684
Ladona fulva LFUL000829
Calopteryx splendens size297646.1
Diptera
Lepidoptera
Trichoptera
Coleoptera
Strepsiptera
Hymenoptera
Psocodea
Thysanoptera
Hemiptera
Blattodea

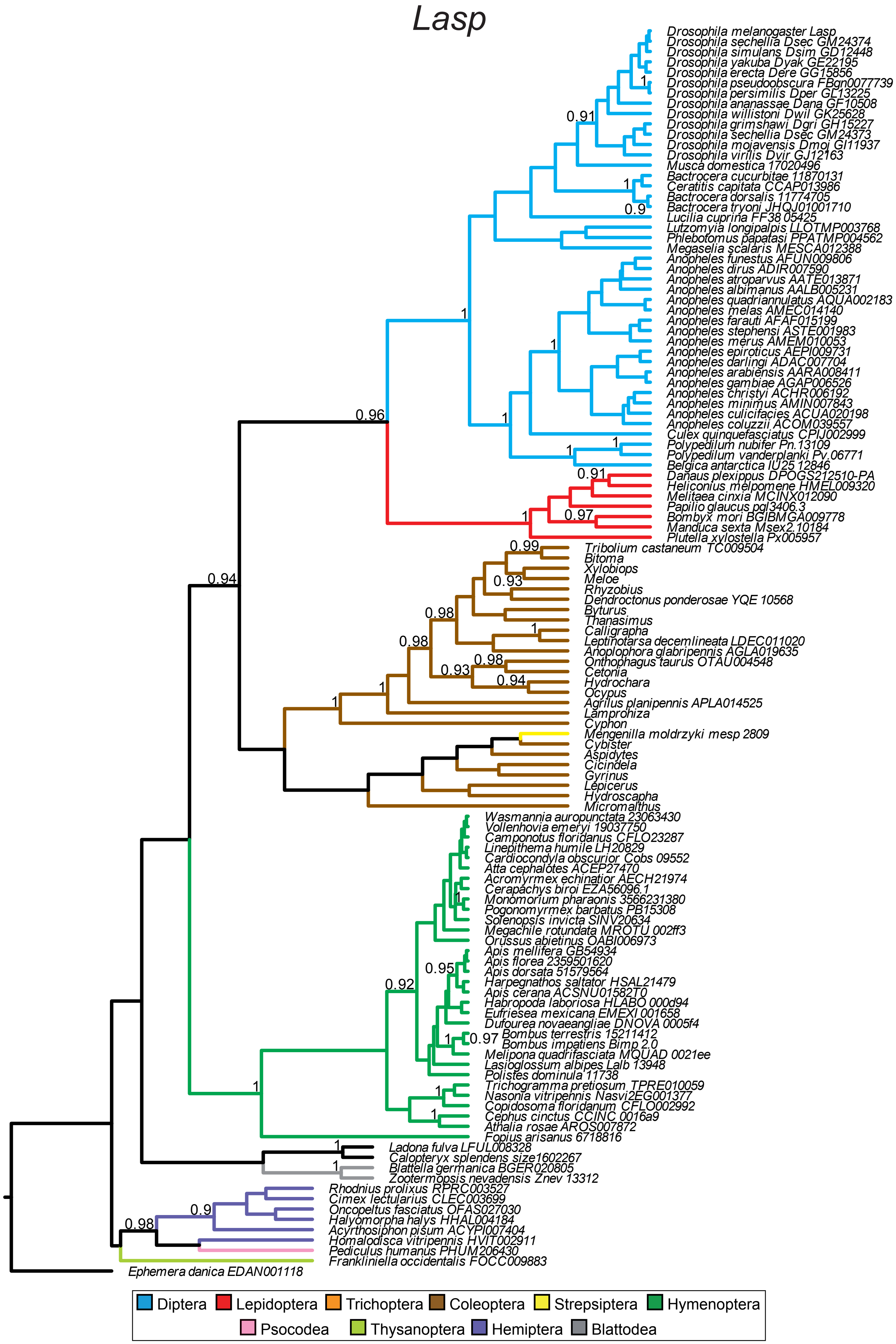

Lasp
Drosophila melanogaster Lasp
Drosophila sechellia Dsec GM24374
Drosophila simulans Dsim GD12448
Drosophila yakuba Dyak GE22195
Drosophila erecta Dere GG15856
Drosophila pseudoobscura FBgn0077739
Drosophila persimilis Dper GL13225
Drosophila ananassae Dana GF10508
Drosophila willistoni Dwil GK25628
Drosophila grimshawi Dgri GH15227
Drosophila sechellia Dsec GM24373
Drosophila mojavensis Dmoj GI11937
Drosophila virilis Dvir GJ12163
Musca domestica 17020496
Bactrocera cucurbitae 11870131
Ceratitis capitata CCAP013986
Bactrocera dorsalis 11774705
Bactrocera tryoni JHQJ01001710
Lucilia cuprina FF38 05425
Lutzomyia longipalpis LLOTMP003768
Phlebotomus papatasi PPATMP004562
Megaselia scalaris MESCA012388
Anopheles funestus AFUN009806
Anopheles dirus ADIR007590
Anopheles atroparvus AATE013871
Anopheles albimanus AALB005231
Anopheles quadriannulatus AQUA002183
Anopheles melas AMEC014140
Anopheles farauti AFAF015199
Anopheles stephensi ASTE001983
Anopheles merus AMEM010053
Anopheles epiroticus AEPI009731
Anopheles darlingi ADAC007704
Anopheles arabiensis AARA008411
Anopheles gambiae AGAP006526
Anopheles christyi ACHR006192
Anopheles minimus AMIN007843
Anopheles culicifacies ACUA020198
Anopheles coluzzii ACOM039557
Culex quinquefasciatus CPIJ002999
Polypedilum nubifer Pn.13109
Polypedilum vanderplanki Pv.06771
Belgica antarctica IU25 12846
Danaus plexippus DPOGS212510-PA
Heliconius melpomene HMEL009320
Melitaea cinxia MCINX012090
Papilio glaucus pgl3406.3
Bombyx mori BGIBMGA009778
Manduca sexta Msex2.10184
Plutella xylostella Px005957
Tribolium castaneum TC009504
Bitoma
Xylobiops
Meloe
Rhyzobius
Dendroctonus ponderosae YQE 10568
Byturus
Thanasimus
Calligrapha
Leptinotarsa decemlineata LDEC011020
Anoplophora glabripennis AGLA019635
Onthophagus taurus OTAU004548
Cetonia
Hydrochara
Ocypus
Agrilus planipennis APLA014525
Lamprohiza
Cyphon
Mengenilla moldrzyki mesp 2809
Cybister
Aspidytes
Cicindela
Gyrinus
Lepicerus
Hydroscapha
Micromalthus
Wasmannia auropunctata 23063430
Vollenhovia emeryi 19037750
Camponotus floridanus CFLO23287
Linepithema humile LH20829
Cardiocondyla obscurior Cobs 09552
Atta cephalotes ACEP27470
Acromyrmex echinatior AECH21974
Cerapachys biroi EZA56096.1
Monomorium pharaonis 3566231380
Pogonomyrmex barbatus PB15308
Solenopsis invicta SINV20634
Megachile rotundata MROTU 002ff3
Orussus abietinus OABI006973
Apis mellifera GB54934
Apis florea 2359501620
Apis dorsata 51579564
Harpegnathos saltator HSAL21479
Apis cerana ACSNU01582T0
Habropoda laboriosa HLABO 000d94
Eufriesea mexicana EMEXI 001658
Dufourea novaeangliae DNOVA 0005f4
Bombus terrestris 15211412
Bombus impatiens Bimp 2.0
Melipona quadrifasciata MQUAD 0021ee
Lasioglossum albipes Lalb 13948
Polistes dominula 11738
Trichogramma pretiosum TPRE010059
Nasonia vitripennis Nasvi2EG001377
Copidosoma floridanum CFLO002992
Cephus cinctus CCINC 0016a9
Athalia rosae AROS007872
Fopius arisanus 6718816
Ladona fulva LFUL008328
Calopteryx splendens size1602267
Blattella germanica BGER020805
Zootermopsis nevadensis Znev 13312
Rhodnius prolixus RPRC003527
Cimex lectularius CLEC003699
Oncopeltus fasciatus OFAS027030
Halyomorpha halys HHAL004184
Acyrthosiphon pisum ACYPI007404
Homalodisca vitripennis HVIT002911
Pediculus humanus PHUM206430
Frankliniella occidentalis FOCC009883
Ephemera danica EDAN001118
Diptera
Lepidoptera
Trichoptera
Coleoptera
Strepsiptera
Hymenoptera
Psocodea
Thysanoptera
Hemiptera
Blattodea

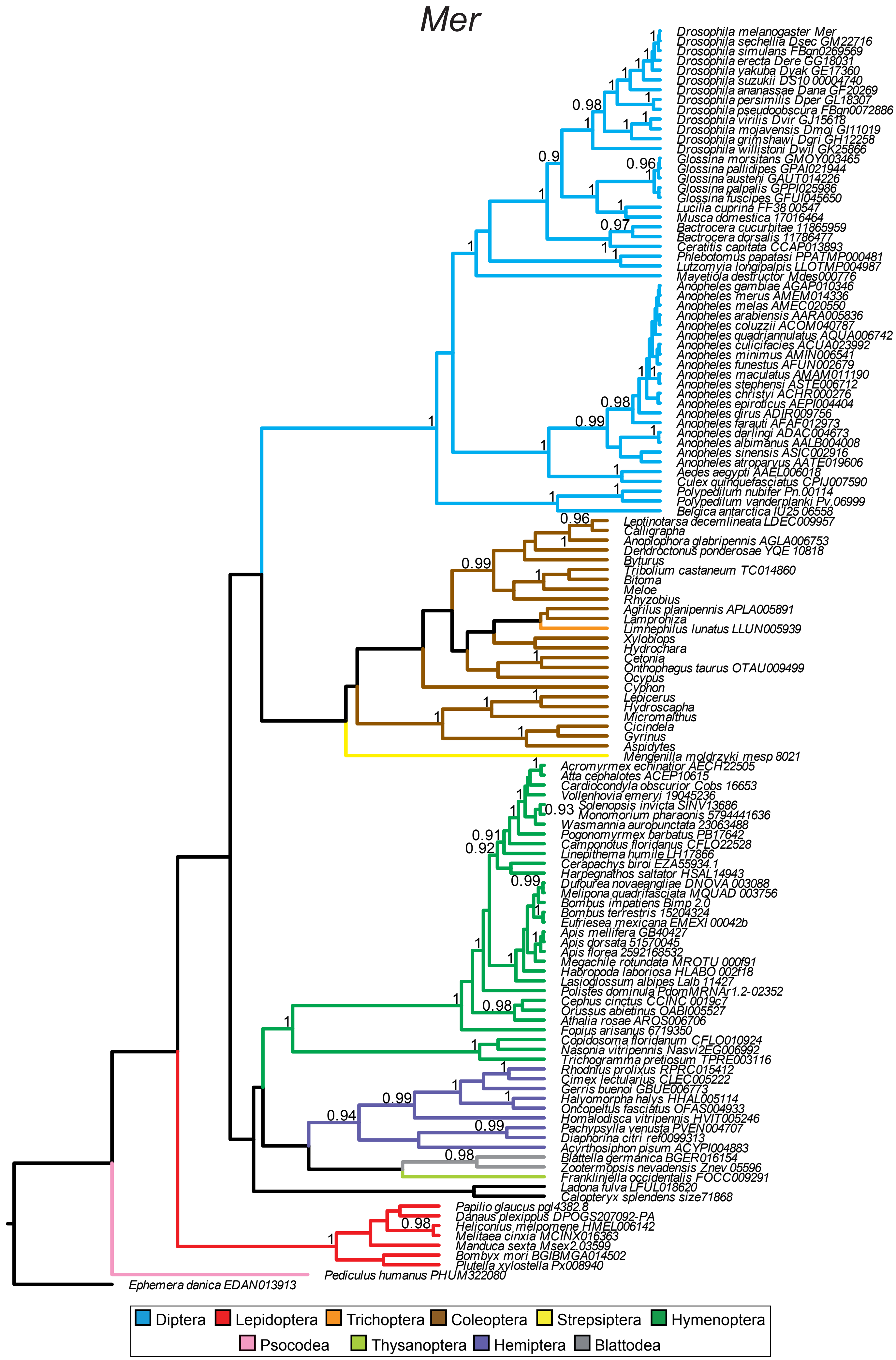
Mer
Drosophila melanogaster Mer
Drosophila sechellia Dsec GM22716
Drosophila simulans FBgn0269569
Drosophila erecta Dere GG18031
Drosophila yakuba Dyak GE17360
Drosophila suzukii DS10 00004740
Drosophila ananassae Dana GF20269
Drosophila persimilis Dper GL18307
Drosophila pseudoobscura FBgn0072886
Drosophila virilis Dvir GJ15618
Drosophila mojavensis Dmoj GI11019
Drosophila grimshawi Dgri GH12258
Drosophila willistoni Dwil GK25866
Glossina morsitans GMOY003465
Glossina pallidipes GPAI021944
Glossina austeni GAUT014226
Glossina palpalis GPPI025986
Glossina fuscipes GFUI045650
Lucilia cuprina FF38 00547
Musca domestica 17016464
Bactrocera cucurbitae 11865959
Bactrocera dorsalis 11786477
Ceratitis capitata CCAP013893
Phlebotomus papatasi PPATMP000481
Lutzomyia longipalpis LLOTMP004987
Mayetiola destructor Mdes000776
Anopheles gambiae AGAP010346
Anopheles merus AMEM014336
Anopheles melas AMEC020550
Anopheles arabiensis AARA005836
Anopheles coluzzii ACOM040787
Anopheles quadriannulatus AQUA006742
Anopheles culicifacies ACUA023992
Anopheles minimus AMIN006541
Anopheles funestus AFUN002679
Anopheles maculatus AMAM011190
Anopheles stephensi ASTE006712
Anopheles christyi ACHR000276
Anopheles epiroticus AEPI004404
Anopheles dirus ADIR009756
Anopheles farauti AFAF012973
Anopheles darlingi ADAC004673
Anopheles albimanus AALB004008
Anopheles sinensis ASIC002916
Anopheles atroparvus AATE019606
Aedes aegypti AAEL006018
Culex quinquefasciatus CPIJ007590
Polypedilum nubifer Pn.00114
Polypedilum vanderplanki Pv.06999
Belgica antarctica IU25 06558
Leptinotarsa decemlineata LDEC009957
Calligrapha
Anoplophora glabripennis AGLA006753
Dendroctonus ponderosae YQE 10818
Byturus
Tribolium castaneum TC014860
Bitoma
Meloe
Rhyzobius
Agrilus planipennis APLA005891
Lamprohiza
Limnephilus lunatus LLUN005939
Xylobiops
Hydrochara
Cetonia
Onthophagus taurus OTAU009499
Ocypus
Cyphon
Lepicerus
Hydroscapha
Micromalthus
Cicindela
Gyrinus
Aspidytes
Mengenilla moldrzyki mesp 8021
Acromyrmex echinatior AECH22505
Atta cephalotes ACEP10615
Cardiocondyla obscurior Cobs 16653
Vollenhovia emeryi 19045236
Solenopsis invicta SINV13686
Monomorium pharaonis 5794441636
Wasmannia auropunctata 23063488
Pogonomyrmex barbatus PB17642
Camponotus floridanus CFLO22528
Linepithema humile LH17866
Cerapachys biroi EZA55934.1
Harpegnathos saltator HSAL14943
Dufourea novaeangliae DNOVA 003088
Melipona quadrifasciata MQUAD 003756
Bombus impatiens Bimp 2.0
Bombus terrestris 15204324
Eufriesea mexicana EMEXI 00042b
Apis mellifera GB40427
Apis dorsata 51570045
Apis florea 2592168532
Megachile rotundata MROTU 000f91
Habropoda laboriosa HLABO 002f18
Lasioglossum albipes Lalb 11427
Polistes dominula PdomMRNAr1.2-02352
Cephus cinctus CCINC 0019c7
Orussus abietinus OABI005527
Athalia rosae AROS006706
Fopius arisanus 6719350
Copidosoma floridanum CFLO010924
Nasonia vitripennis Nasvi2EG006992
Trichogramma pretiosum TPRE003116
Rhodnius prolixus RPRC015412
Cimex lectularius CLEC005222
Gerris buenoi GBUE006773
Halyomorpha halys HHAL005114
Oncopeltus fasciatus OFAS004933
Homalodisca vitripennis HVIT005246
Pachypsylla venusta PVEN004707
Diaphorina citri ref0099313
Acyrthosiphon pisum ACYPI004883
Blattella germanica BGER016154
Zootermopsis nevadensis Znev 05596
Frankliniella occidentalis FOCC009291
Ladona fulva LFUL018620
Calopteryx splendens size71868
Papilio glaucus pgl4382.8
Danaus plexippus DPOGS207092-PA
Heliconius melpomene HMEL006142
Melitaea cinxia MCINX016363
Manduca sexta Msex2.03599
Bombyx mori BGIBMGA014502
Plutella xylostella Px008940
Pediculus humanus PHUM322080
Ephemera danica EDAN013913
Diptera
Lepidoptera
Trichoptera
Coleoptera
Strepsiptera
Hymenoptera
Psocodea
Thysanoptera
Hemiptera
Blattodea

mlt

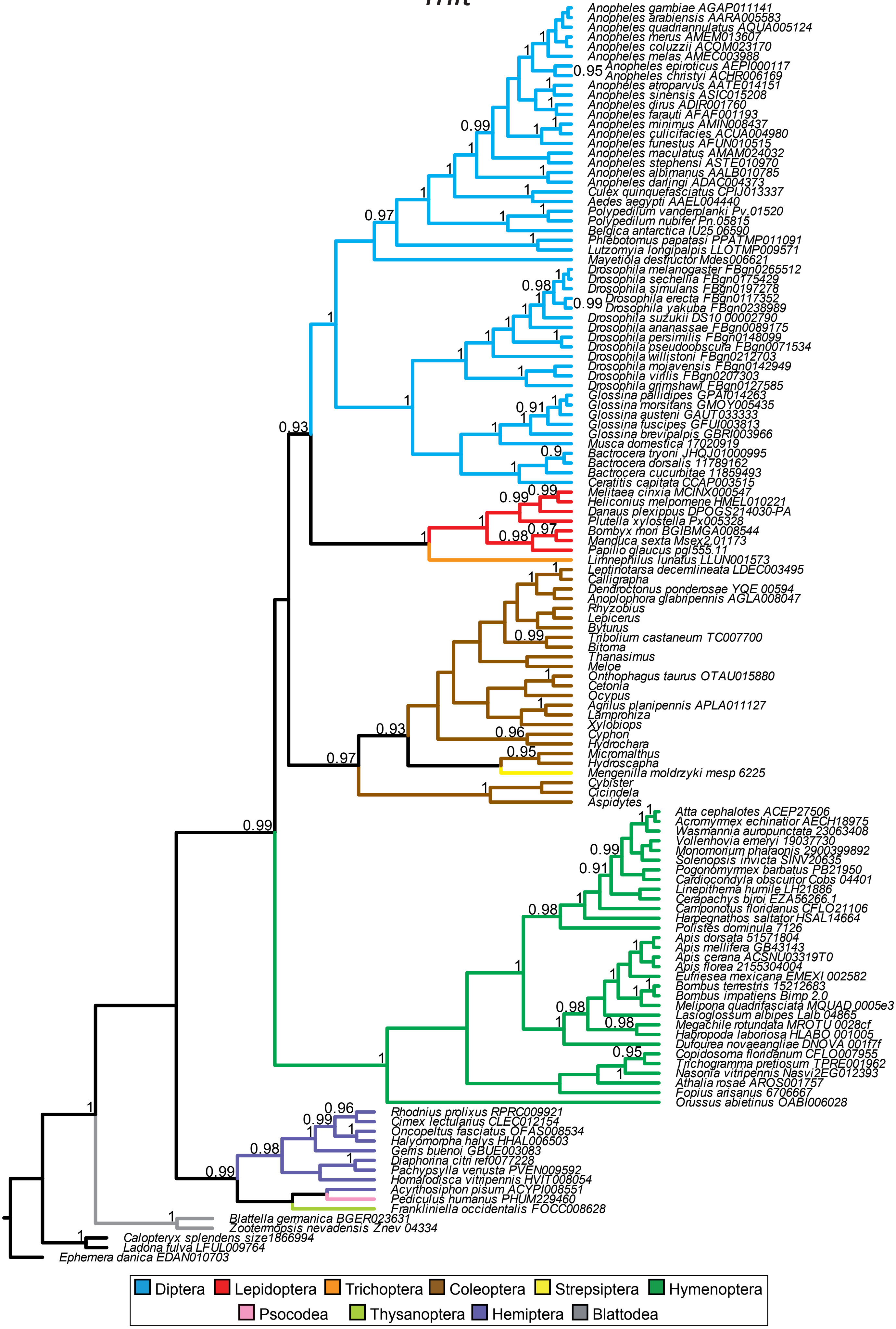

Anopheles gambiae AGAP011141
Anopheles arabiensis AARA005583
Anopheles quadriannulatus AQUA005124
Anopheles merus AMEM013607
Anopheles coluzzii ACOM023170
Anopheles melas AMEC003988
Anopheles epiroticus AEPI000117
Anopheles christyi ACHR006169
Anopheles atroparvus AATE014151
Anopheles sinensis ASIC015208
Anopheles dirus ADIR001760
Anopheles farauti AFAF001193
Anopheles minimus AMIN008437
Anopheles culicifacies ACUA004980
Anopheles funestus AFUN010515
Anopheles maculatus AMAM024032
Anopheles stephensi ASTE010970
Anopheles albimanus AALB010785
Anopheles darlingi ADAC004373
Culex quinquefasciatus CPIJ013337
Aedes aegypti AAEL004440
Polypedilum vanderplanki Pv.01520
Polypedilum nubifer Pn.05815
Belgica antarctica IU25 06590
Phlebotomus papatasi PPATMP011091
Lutzomyia longipalpis LLOTMP009571
Mayetiola destructor Mdes006621
Drosophila melanogaster FBgn0265512
Drosophila sechellia FBgn0175429
Drosophila simulans FBgn0197278
Drosophila erecta FBgn0117352
Drosophila yakuba FBgn0238989
Drosophila suzukii DS10 00002790
Drosophila ananassae FBgn0089175
Drosophila persimilis FBgn0148099
Drosophila pseudoobscura FBgn0071534
Drosophila willistoni FBgn0212703
Drosophila mojavensis FBgn0142949
Drosophila virilis FBgn0207303
Drosophila grimshawi FBgn0127585
Glossina pallidipes GPAI014263
Glossina morsitans GMOY005435
Glossina austeni GAUT033333
Glossina fuscipes GFUI003813
Glossina brevipalpis GBRI003966
Musca domestica 17020919
Bactrocera tryoni JHQJ01000995
Bactrocera dorsalis 11789162
Bactrocera cucurbitae 11859493
Ceratitis capitata CCAP003515
Melitaea cinxia MCINX000547
Heliconius melpomene HMEL010221
Danaus plexippus DPOGS214030-PA
Plutella xylostella Px005328
Bombyx mori BGIBMGA008544
Manduca sexta Msex2.01173
Papilio glaucus pgl555.11
Limnephilus lunatus LLUN001573
Leptinotarsa decemlineata LDEC003495
Calligrapha
Dendroctonus ponderosae YQE 00594
Anoplophora glabripennis AGLA008047
Rhyzobius
Lepicerus
Byturus
Tribolium castaneum TC007700
Bitoma
Thanasimus
Meloe
Onthophagus taurus OTAU015880
Cetonia
Ocypus
Agrilus planipennis APLA011127
Lamprohiza
Xylobiops
Cyphon
Hydrochara
Micromalthus
Hydroscapha
Mengenilla moldrzyki mesp 6225
Cybister
Cicindela
Aspidytes
Atta cephalotes ACEP27506
Acromyrmex echinatior AECH18975
Wasmannia auropunctata 23063408
Vollenhovia emeryi 19037730
Monomorium pharaonis 2900399892
Solenopsis invicta SINV20635
Pogonomyrmex barbatus PB21950
Cardiocondyla obscurior Cobs 04401
Linepithema humile LH21886
Cerapachys biroi EZA56266.1
Camponotus floridanus CFLO21106
Harpegnathos saltator HSAL14664
Polistes dominula 7126
Apis dorsata 51571804
Apis mellifera GB43143
Apis cerana ACSNU03319T0
Apis florea 2155304004
Eufriesea mexicana EMEXI 002582
Bombus terrestris 15212683
Bombus impatiens Bimp 2.0
Melipona quadrifasciata MQUAD 0005e3
Lasioglossum albipes Lalb 04865
Megachile rotundata MROTU 0028cf
Habropoda laboriosa HLABO 001005
Dufourea novaeangliae DNOVA 001f7f
Copidosoma floridanum CFLO007955
Trichogramma pretiosum TPRE001962
Nasonia vitripennis Nasvi2EG012393
Athalia rosae AROS001757
Fopius arisanus 6706667
Orussus abietinus OABI006028
Rhodnius prolixus RPRC009921
Cimex lectularius CLEC012154
Oncopeltus fasciatus OFAS008534
Halyomorpha halys HHAL006503
Gerris buenoi GBUE003083
Diaphorina citri ref0077228
Pachypsylla venusta PVEN009592
Homalodisca vitripennis HVIT008054
Acyrthosiphon pisum ACYPI008551
Pediculus humanus PHUM229460
Frankliniella occidentalis FOCC008628
Blattella germanica BGER023631
Zootermopsis nevadensis Znev 04334
Calopteryx splendens size1866994
Ladona fulva LFUL009764
Ephemera danica EDAN010703
Diptera
Lepidoptera
Trichoptera
Coleoptera
Strepsiptera
Hymenoptera
Psocodea
Thysanoptera
Hemiptera
Blattodea

nes

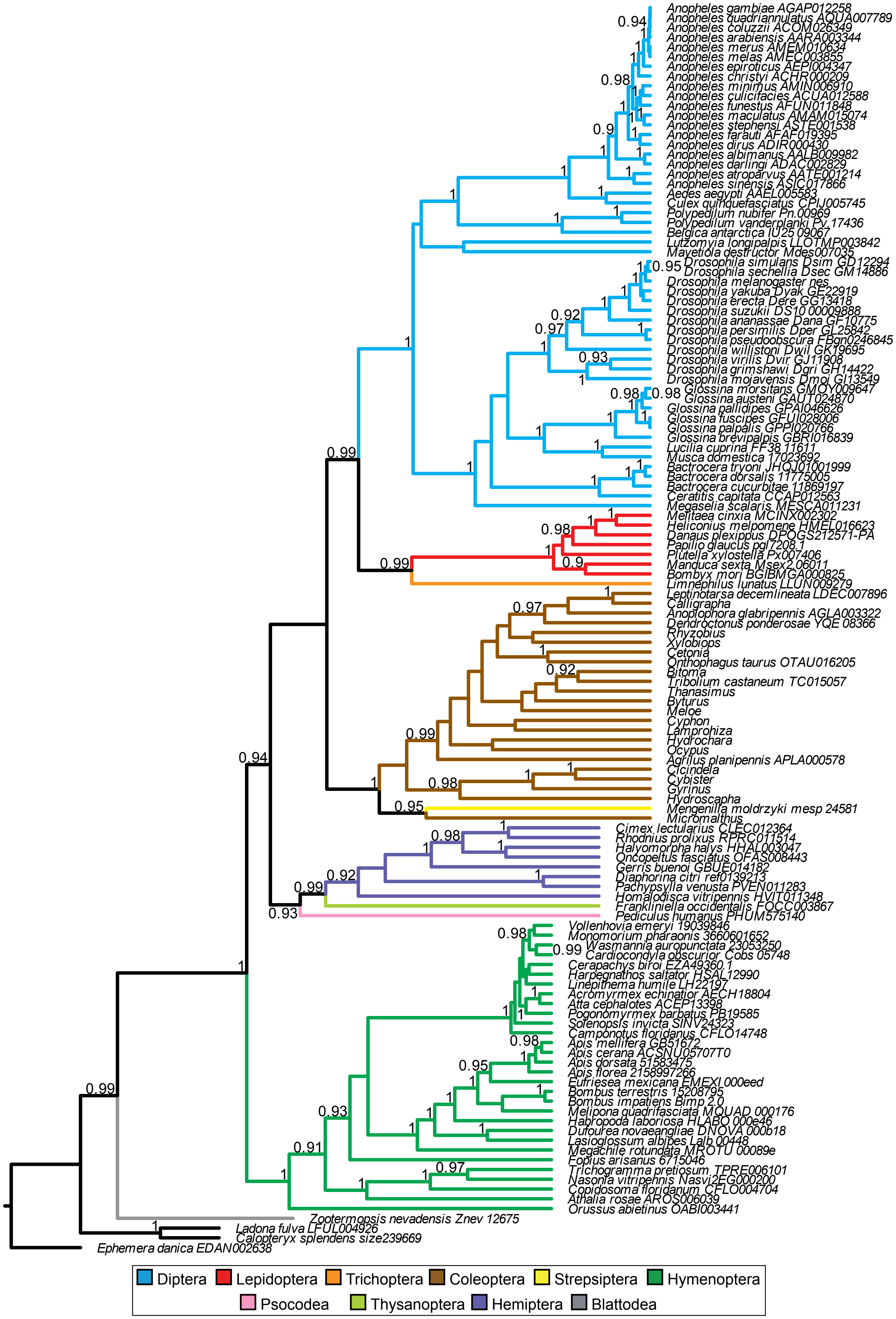

Anopheles gambiae AGAP012258
Anopheles quadriannulatus AQUA007789
Anopheles coluzzii ACOM026349
Anopheles arabiensis AARA003344
Anopheles merus AMEM010634
Anopheles melas AMEC003855
Anopheles epiroticus AEPI004347
Anopheles christyi ACHR000209
Anopheles minimus AMIN006910
Anopheles culicifacies ACUA012588
Anopheles funestus AFUN011848
Anopheles maculatus AMAM015074
Anopheles stephensi ASTE001538
Anopheles farauti AFAF019395
Anopheles dirus ADIR000430
Anopheles albimanus AALB009982
Anopheles darlingi ADAC002829
Anopheles atroparvus AATE001214
Anopheles sinensis ASIC017866
Aedes aegypti AAEL005583
Culex quinquefasciatus CPIJ005745
Polypedilum nubifer Pn.00969
Polypedilum vanderplanki Pv.17436
Belgica antarctica IU25 09067
Lutzomyia longipalpis LLOTMP003842
Mayetiola destructor Mdes007035
Drosophila simulans Dsim GD12294
Drosophila sechellia Dsec GM14886
Drosophila melanogaster nes
Drosophila yakuba Dyak GE22919
Drosophila erecta Dere GG13418
Drosophila suzukii DS10 00009888
Drosophila ananassae Dana GF10775
Drosophila persimilis Dper GL25842
Drosophila pseudoobscura FBgn0246845
Drosophila willistoni Dwil GK19695
Drosophila virilis Dvir GJ11908
Drosophila grimshawi Dgri GH14422
Drosophila mojavensis Dmoj GI13549
Glossina morsitans GMOY009647
Glossina austeni GAUT024870
Glossina pallidipes GPAI046626
Glossina fuscipes GFUI028006
Glossina palpalis GPPI020766
Glossina brevipalpis GBRI016839
Lucilia cuprina FF38 11611
Musca domestica 17023692
Bactrocera tryoni JHQJ01001999
Bactrocera dorsalis 11775005
Bactrocera cucurbitae 11869197
Ceratitis capitata CCAP012563
Megaselia scalaris MESCA011231
Melitaea cinxia MCINX002302
Heliconius melpomene HMEL016623
Danaus plexippus DPOGS212571-PA
Papilio glaucus pgl7208.1
Plutella xylostella Px007406
Manduca sexta Msex2.06011
Bombyx mori BGIBMGA000825
Limnephilus lunatus LLUN009279
Leptinotarsa decemlineata LDEC007896
Calligrapha
Anoplophora glabripennis AGLA003322
Dendroctonus ponderosae YQE 08366
Rhyzobius
Xylobiops
Cetonia
Onthophagus taurus OTAU016205
Bitoma
Tribolium castaneum TC015057
Thanasimus
Byturus
Meloe
Cyphon
Lamprohiza
Hydrochara
Ocypus
Agrilus planipennis APLA000578
Cicindela
Cybister
Gyrinus
Hydroscapha
Mengenilla moldrzyki mesp 24581
Micromalthus
Cimex lectularius CLEC012364
Rhodnius prolixus RPRC011514
Halyomorpha halys HHAL003047
Oncopeltus fasciatus OFAS008443
Gerris buenoi GBUE014182
Diaphorina citri ref0139213
Pachypsylla venusta PVEN011283
Homalodisca vitripennis HVIT011348
Frankliniella occidentalis FOCC003867
Pediculus humanus PHUM575140
Vollenhovia emeryi 19039846
Monomorium pharaonis 3660601652
Wasmannia auropunctata 23053250
Cardiocondyla obscurior Cobs 05748
Cerapachys biroi EZA49360.1
Harpegnathos saltator HSAL12990
Linepithema humile LH22197
Acromyrmex echinatior AECH18804
Atta cephalotes ACEP13398
Pogonomyrmex barbatus PB19585
Solenopsis invicta SINV24323
Camponotus floridanus CFLO14748
Apis mellifera GB51672
Apis cerana ACSNU05707T0
Apis dorsata 51583475
Apis florea 2158997266
Eufriesea mexicana EMEXI 000eed
Bombus terrestris 15208795
Bombus impatiens Bimp 2.0
Melipona quadrifasciata MQUAD 000176
Habropoda laboriosa HLABO 000e46
Dufourea novaeangliae DNOVA 000b18
Lasioglossum albipes Lalb 00448
Megachile rotundata MROTU 00089e
Fopius arisanus 6715046
Trichogramma pretiosum TPRE006101
Nasonia vitripennis Nasvi2EG000200
Copidosoma floridanum CFLO004704
Athalia rosae AROS006039
Orussus abietinus OABI003441
Zootermopsis nevadensis Znev 12675
Ladona fulva LFUL004926
Calopteryx splendens size239669
Ephemera danica EDAN002638
Diptera
Lepidoptera
Trichoptera
Coleoptera
Strepsiptera
Hymenoptera
Psocodea
Thysanoptera
Hemiptera
Blattodea

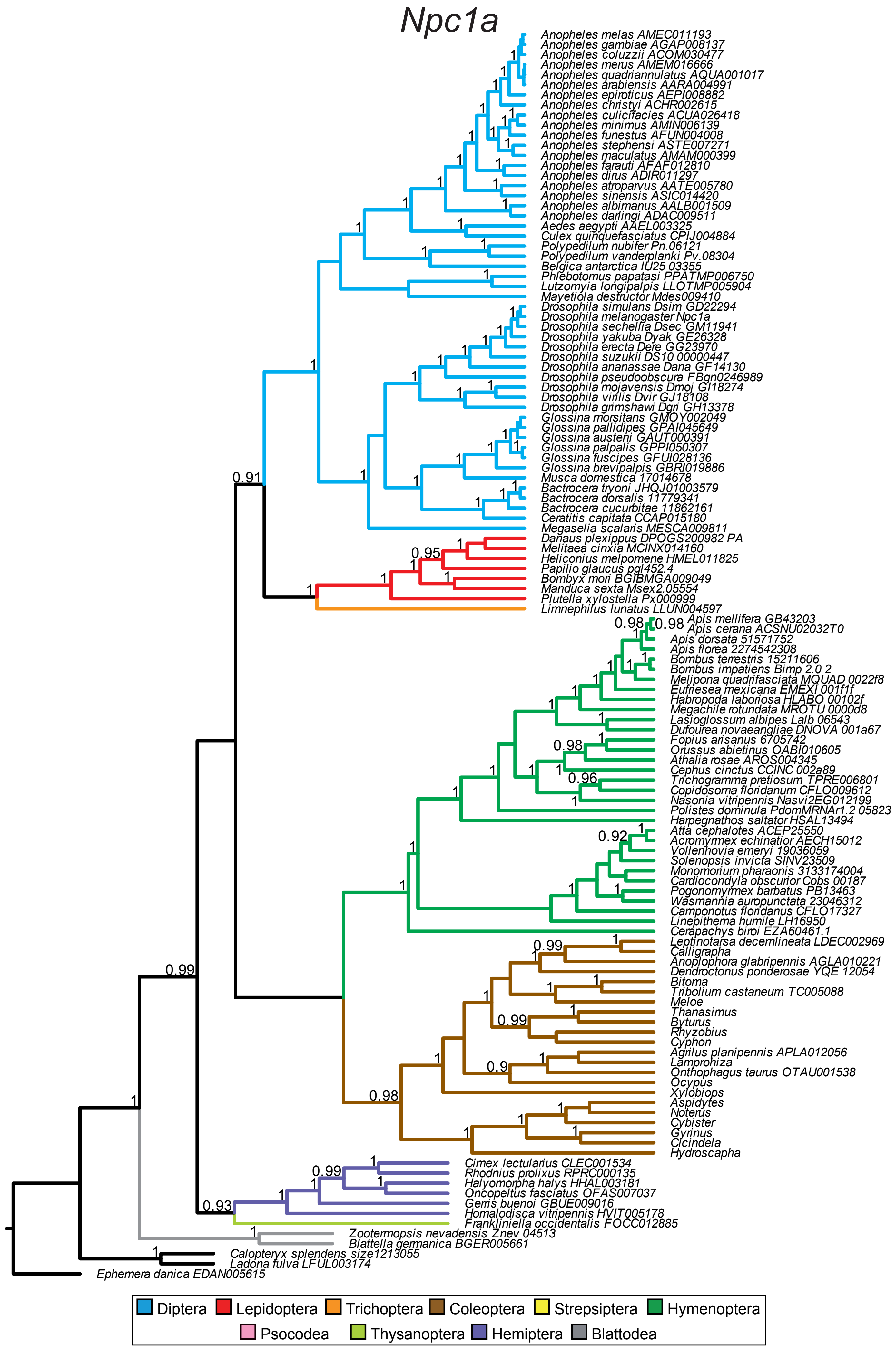
Npc1a
Anopheles melas AMEC011193
Anopheles gambiae AGAP008137
Anopheles coluzzii ACOM030477
Anopheles merus AMEM016666
Anopheles quadriannulatus AQUA001017
Anopheles arabiensis AARA004991
Anopheles epiroticus AEPI008882
Anopheles christyi ACHR002615
Anopheles culicifacies ACUA026418
Anopheles minimus AMIN006139
Anopheles funestus AFUN004008
Anopheles stephensi ASTE007271
Anopheles maculatus AMAM000399
Anopheles farauti AFAF012810
Anopheles dirus ADIR011297
Anopheles atroparvus AATE005780
Anopheles sinensis ASIC014420
Anopheles albimanus AALB001509
Anopheles darlingi ADAC009511
Aedes aegypti AAEL003325
Culex quinquefasciatus CPIJ004884
Polypedilum nubifer Pn.06121
Polypedilum vanderplanki Pv.08304
Belgica antarctica IU25 03355
Phlebotomus papatasi PPATMP006750
Lutzomyia longipalpis LLOTMP005904
Mayetiola destructor Mdes009410
Drosophila simulans Dsim GD22294
Drosophila melanogaster Npc1a
Drosophila sechellia Dsec GM11941
Drosophila yakuba Dyak GE26328
Drosophila erecta Dere GG23970
Drosophila suzukii DS10 00000447
Drosophila ananassae Dana GF14130
Drosophila pseudoobscura FBgn0246989
Drosophila mojavensis Dmoj GI18274
Drosophila virilis Dvir GJ18108
Drosophila grimshawi Dgri GH13378
Glossina morsitans GMOY002049
Glossina pallidipes GPAI045649
Glossina austeni GAUT000391
Glossina palpalis GPPI050307
Glossina fuscipes GFUI028136
Glossina brevipalpis GBRI019886
Musca domestica 17014678
Bactrocera tryoni JHQJ01003579
Bactrocera dorsalis 11779341
Bactrocera cucurbitae 11862161
Ceratitis capitata CCAP015180
Megaselia scalaris MESCA009811
Danaus plexippus DPOGS200982 PA
Melitaea cinxia MCINX014160
Heliconius melpomene HMEL011825
Papilio glaucus pgl452.4
Bombyx mori BGIBMGA009049
Manduca sexta Msex2.05554
Plutella xylostella Px000999
Limnephilus lunatus LLUN004597
Apis mellifera GB43203
Apis cerana ACSNU02032T0
Apis dorsata 51571752
Apis florea 2274542308
Bombus terrestris 15211606
Bombus impatiens Bimp 2.0 2
Melipona quadrifasciata MQUAD 0022f8
Eufriesea mexicana EMEXI 001f1f
Habropoda laboriosa HLABO 00102f
Megachile rotundata MROTU 0000d8
Lasioglossum albipes Lalb 06543
Dufourea novaeangliae DNOVA 001a67
Fopius arisanus 6705742
Orussus abietinus OABI010605
Athalia rosae AROS004345
Cephus cinctus CCINC 002a89
Trichogramma pretiosum TPRE006801
Copidosoma floridanum CFLO009612
Nasonia vitripennis Nasvi2EG012199
Polistes dominula PdomMRNAr1.2 05823
Harpegnathos saltator HSAL13494
Atta cephalotes ACEP25550
Acromyrmex echinatior AECH15012
Vollenhovia emeryi 19036059
Solenopsis invicta SINV23509
Monomorium pharaonis 3133174004
Cardiocondyla obscurior Cobs 00187
Pogonomyrmex barbatus PB13463
Wasmannia auropunctata 23046312
Camponotus floridanus CFLO17327
Linepithema humile LH16950
Cerapachys biroi EZA60461.1
Leptinotarsa decemlineata LDEC002969
Calligrapha
Anoplophora glabripennis AGLA010221
Dendroctonus ponderosae YQE 12054
Bitoma
Tribolium castaneum TC005088
Meloe
Thanasimus
Byturus
Rhyzobius
Cyphon
Agrilus planipennis APLA012056
Lamprohiza
Onthophagus taurus OTAU001538
Ocypus
Xylobiops
Aspidytes
Noterus
Cybister
Gyrinus
Cicindela
Hydroscapha
Cimex lectularius CLEC001534
Rhodnius prolixus RPRC000135
Halyomorpha halys HHAL003181
Oncopeltus fasciatus OFAS007037
Gerris buenoi GBUE009016
Homalodisca vitripennis HVIT005178
Frankliniella occidentalis FOCC012885
Zootermopsis nevadensis Znev 04513
Blattella germanica BGER005661
Calopteryx splendens size1213055
Ladona fulva LFUL003174
Ephemera danica EDAN005615
0.91
0.95
0.98
0.98
0.98
0.96
0.92
0.99
0.99
0.9
0.98
0.99
0.99
0.93
Diptera
Lepidoptera
Trichoptera
Coleoptera
Strepsiptera
Hymenoptera
Psocodea
Thysanoptera
Hemiptera
Blattodea

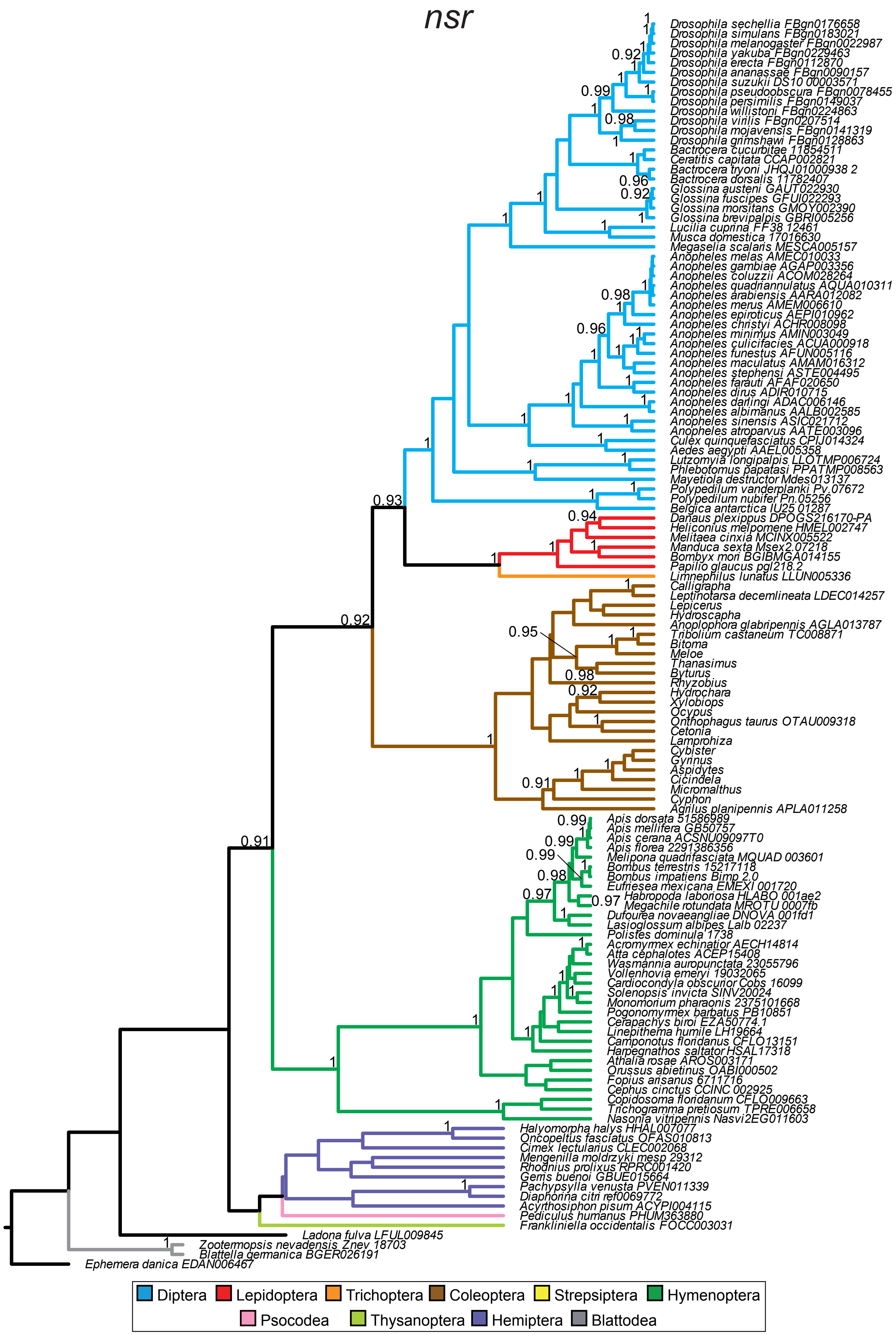
nsr
Drosophila sechellia FBgn0176658
Drosophila simulans FBgn0183021
Drosophila melanogaster FBgn0022987
Drosophila yakuba FBgn0229463
Drosophila erecta FBgn0112870
Drosophila ananassae FBgn0090157
Drosophila suzukii DS10 00003571
Drosophila pseudoobscura FBgn0078455
Drosophila persimilis FBgn0149037
Drosophila willistoni FBgn0224863
Drosophila virilis FBgn0207514
Drosophila mojavensis FBgn0141319
Drosophila grimshawi FBgn0128863
Bactrocera cucurbitae 11854511
Ceratitis capitata CCAP002821
Bactrocera tryoni JHQJ01000938 2
Bactrocera dorsalis 11782407
Glossina austeni GAUT022930
Glossina fuscipes GFUI022293
Glossina morsitans GMOY002390
Glossina brevipalpis GBRI005256
Lucilia cuprina FF38 12461
Musca domestica 17016630
Megaselia scalaris MESCA005157
Anopheles melas AMEC010033
Anopheles gambiae AGAP003356
Anopheles coluzzii ACOM028264
Anopheles quadriannulatus AQUA010311
Anopheles arabiensis AARA012082
Anopheles merus AMEM006610
Anopheles epiroticus AEPI010962
Anopheles christyi ACHR008098
Anopheles minimus AMIN003049
Anopheles culicifacies ACUA000918
Anopheles funestus AFUN005116
Anopheles maculatus AMAM016312
Anopheles stephensi ASTE004495
Anopheles farauti AFAF020650
Anopheles dirus ADIR010715
Anopheles darlingi ADAC006146
Anopheles albimanus AALB002585
Anopheles sinensis ASIC021712
Anopheles atroparvus AATE003096
Culex quinquefasciatus CPIJ014324
Aedes aegypti AAEL005358
Lutzomyia longipalpis LLOTMP006724
Phlebotomus papatasi PPATMP008563
Mayetiola destructor Mdes013137
Polypedilum vanderplanki Pv.07672
Polypedilum nubifer Pn.05256
Belgica antarctica IU25 01287
Danaus plexippus DPOGS216170-PA
Heliconius melpomene HMEL002747
Melitaea cinxia MCINX005522
Manduca sexta Msex2.07218
Bombyx mori BGIBMGA014155
Papilio glaucus pgl218.2
Limnephilus lunatus LLUN005336
Calligrapha
Leptinotarsa decemlineata LDEC014257
Lepicerus
Hydroscapha
Anoplophora glabripennis AGLA013787
Tribolium castaneum TC008871
Bitoma
Meloe
Thanasimus
Byturus
Rhyzobius
Hydrochara
Xylobiops
Ocypus
Onthophagus taurus OTAU009318
Cetonia
Lamprohiza
Cybister
Gyrinus
Aspidytes
Cicindela
Micromalthus
Cyphon
Agrilus planipennis APLA011258
Apis dorsata 51586989
Apis mellifera GB50757
Apis cerana ACSNU09097T0
Apis florea 2291386356
Melipona quadrifasciata MQUAD 003601
Bombus terrestris 15217118
Bombus impatiens Bimp 2.0
Eufriesea mexicana EMEXI 001720
Habropoda laboriosa HLABO 001ae2
Megachile rotundata MROTU 0007fb
Dufourea novaeangliae DNOVA 001fd1
Lasioglossum albipes Lalb 02237
Polistes dominula 1738
Acromyrmex echinatior AECH14814
Atta cephalotes ACEP15408
Wasmannia auropunctata 23055796
Vollenhovia emeryi 19032065
Cardiocondyla obscurior Cobs 16099
Solenopsis invicta SINV20024
Monomorium pharaonis 2375101668
Pogonomyrmex barbatus PB10851
Cerapachys biroi EZA50774.1
Linepithema humile LH19664
Camponotus floridanus CFLO13151
Harpegnathos saltator HSAL17318
Athalia rosae AROS003171
Orussus abietinus OABI000502
Fopius arisanus 6711716
Cephus cinctus CCINC 002925
Copidosoma floridanum CFLO009663
Trichogramma pretiosum TPRE006658
Nasonia vitripennis Nasvi2EG011603
Halyomorpha halys HHAL007077
Oncopeltus fasciatus OFAS010813
Cimex lectularius CLEC002068
Mengenilla moldrzyki mesp 29312
Rhodnius prolixus RPRC001420
Gerris buenoi GBUE015664
Pachypsylla venusta PVEN011339
Diaphorina citri ref0069772
Acyrthosiphon pisum ACYPI004115
Pediculus humanus PHUM363880
Frankliniella occidentalis FOCC003031
Ladona fulva LFUL009845
Zootermopsis nevadensis Znev 18703
Blattella germanica BGER026191
Ephemera danica EDAN006467
Diptera
Lepidoptera
Trichoptera
Coleoptera
Strepsiptera
Hymenoptera
Psocodea
Thysanoptera
Hemiptera
Blattodea

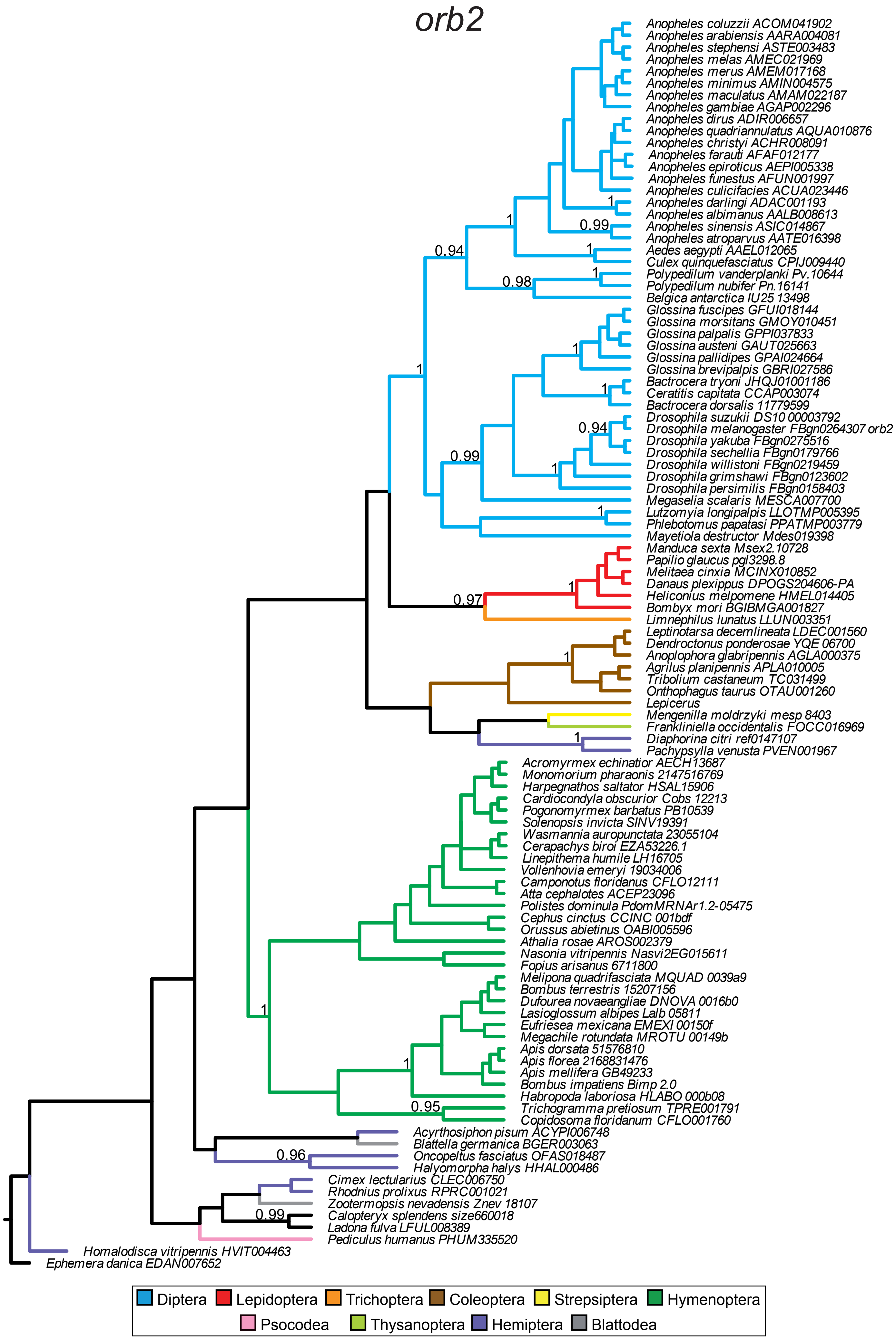

orb2
Anopheles coluzzii ACOM041902
Anopheles arabiensis AARA004081
Anopheles stephensi ASTE003483
Anopheles melas AMEC021969
Anopheles merus AMEM017168
Anopheles minimus AMIN004575
Anopheles maculatus AMAM022187
Anopheles gambiae AGAP002296
Anopheles dirus ADIR006657
Anopheles quadriannulatus AQUA010876
Anopheles christyi ACHR008091
Anopheles farauti AFAF012177
Anopheles epiroticus AEPI005338
Anopheles funestus AFUN001997
Anopheles culicifacies ACUA023446
Anopheles darlingi ADAC001193
Anopheles albimanus AALB008613
Anopheles sinensis ASIC014867
Anopheles atroparvus AATE016398
Aedes aegypti AAEL012065
Culex quinquefasciatus CPIJ009440
Polypedilum vanderplanki Pv.10644
Polypedilum nubifer Pn.16141
Belgica antarctica IU25 13498
Glossina fuscipes GFUI018144
Glossina morsitans GMOY010451
Glossina palpalis GPPI037833
Glossina austeni GAUT025663
Glossina pallidipes GPAI024664
Glossina brevipalpis GBRI027586
Bactrocera tryoni JHQJ01001186
Ceratitis capitata CCAP003074
Bactrocera dorsalis 11779599
Drosophila suzukii DS10 00003792
Drosophila melanogaster FBgn0264307 orb2
Drosophila yakuba FBgn0275516
Drosophila sechellia FBgn0179766
Drosophila willistoni FBgn0219459
Drosophila grimshawi FBgn0123602
Drosophila persimilis FBgn0158403
Megaselia scalaris MESCA007700
Lutzomyia longipalpis LLOTMP005395
Phlebotomus papatasi PPATMP003779
Mayetiola destructor Mdes019398
Manduca sexta Msex2.10728
Papilio glaucus pgl3298.8
Melitaea cinxia MCINX010852
Danaus plexippus DPOGS204606-PA
Heliconius melpomene HMEL014405
Bombyx mori BGIBMGA001827
Limnephilus lunatus LLUN003351
Leptinotarsa decemlineata LDEC001560
Dendroctonus ponderosae YQE 06700
Anoplophora glabripennis AGLA000375
Agrilus planipennis APLA010005
Tribolium castaneum TC031499
Onthophagus taurus OTAU001260
Lepicerus
Mengenilla moldrzyki mesp 8403
Frankliniella occidentalis FOCC016969
Diaphorina citri ref0147107
Pachypsylla venusta PVEN001967
Acromyrmex echinatior AECH13687
Monomorium pharaonis 2147516769
Harpegnathos saltator HSAL15906
Cardiocondyla obscurior Cobs 12213
Pogonomyrmex barbatus PB10539
Solenopsis invicta SINV19391
Wasmannia auropunctata 23055104
Cerapachys biroi EZA53226.1
Linepithema humile LH16705
Vollenhovia emeryi 19034006
Camponotus floridanus CFLO12111
Atta cephalotes ACEP23096
Polistes dominula PdomMRNAr1.2-05475
Cephus cinctus CCINC 001bdf
Orussus abietinus OABI005596
Athalia rosae AROS002379
Nasonia vitripennis Nasvi2EG015611
Fopius arisanus 6711800
Melipona quadrifasciata MQUAD 0039a9
Bombus terrestris 15207156
Dufourea novaeangliae DNOVA 0016b0
Lasioglossum albipes Lalb 05811
Eufriesea mexicana EMEXI 00150f
Megachile rotundata MROTU 00149b
Apis dorsata 51576810
Apis florea 2168831476
Apis mellifera GB49233
Bombus impatiens Bimp 2.0
Habropoda laboriosa HLABO 000b08
Trichogramma pretiosum TPRE001791
Copidosoma floridanum CFLO001760
Acyrthosiphon pisum ACYPI006748
Blattella germanica BGER003063
Oncopeltus fasciatus OFAS018487
Halyomorpha halys HHAL000486
Cimex lectularius CLEC006750
Rhodnius prolixus RPRC001021
Zootermopsis nevadensis Znev 18107
Calopteryx splendens size660018
Ladona fulva LFUL008389
Pediculus humanus PHUM335520
Homalodisca vitripennis HVIT004463
Ephemera danica EDAN007652
Diptera
Lepidoptera
Trichoptera
Coleoptera
Strepsiptera
Hymenoptera
Psocodea
Thysanoptera
Hemiptera
Blattodea

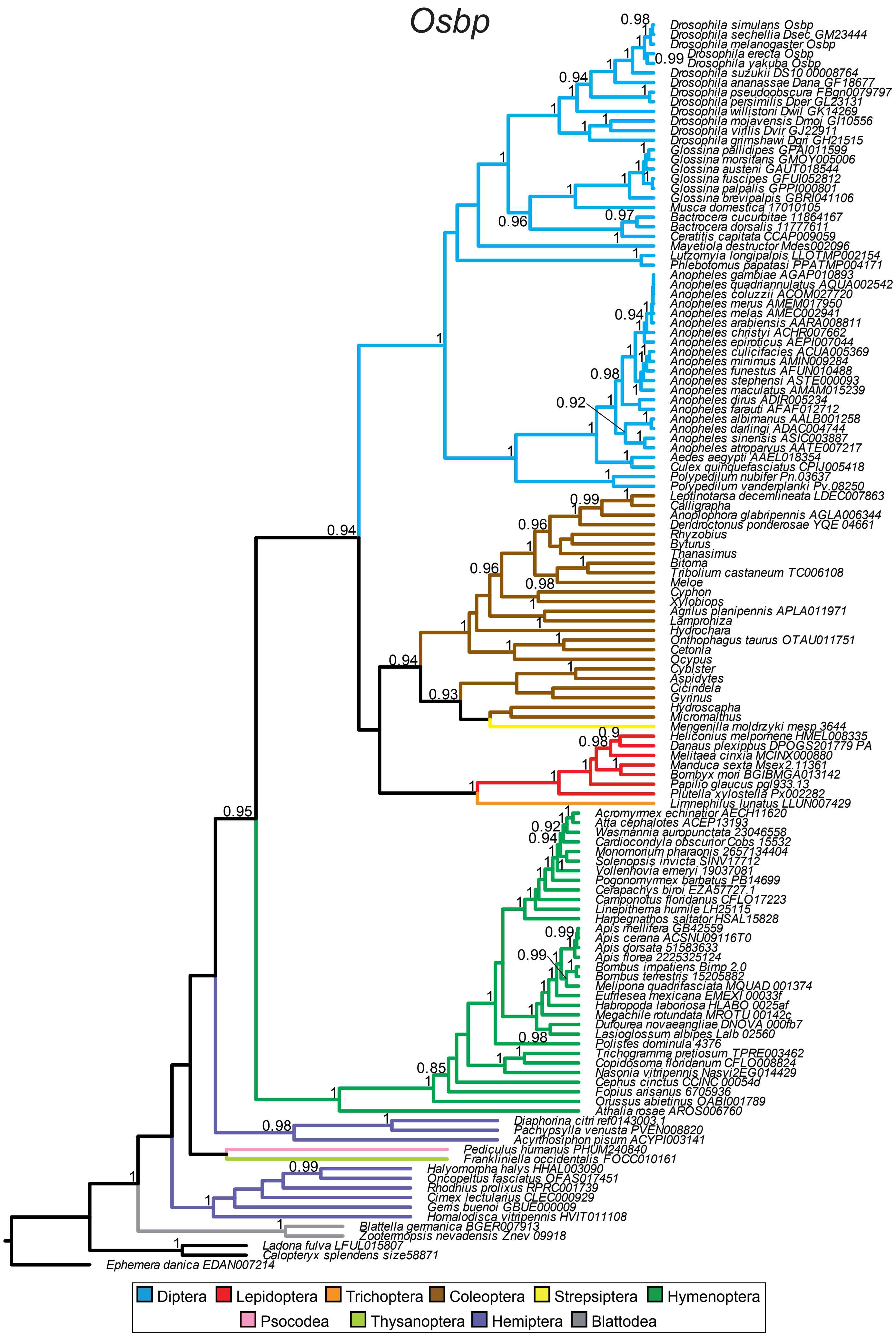
Osbp
Drosophila simulans Osbp
Drosophila sechellia Dsec GM23444
Drosophila melanogaster Osbp
Drosophila erecta Osbp
Drosophila yakuba Osbp
Drosophila suzukii DS10 00008764
Drosophila ananassae Dana GF18677
Drosophila pseudoobscura FBgn0079797
Drosophila persimilis Dper GL23131
Drosophila willistoni Dwil GK14269
Drosophila mojavensis Dmoj GI10556
Drosophila virilis Dvir GJ22911
Drosophila grimshawi Dgri GH21515
Glossina pallidipes GPAI011599
Glossina morsitans GMOY005006
Glossina austeni GAUT018544
Glossina fuscipes GFUI052812
Glossina palpalis GPPI000801
Glossina brevipalpis GBRI041106
Musca domestica 17010105
Bactrocera cucurbitae 11864167
Bactrocera dorsalis 11777611
Ceratitis capitata CCAP009059
Mayetiola destructor Mdes002096
Lutzomyia longipalpis LLOTMP002154
Phlebotomus papatasi PPATMP004171
Anopheles gambiae AGAP010893
Anopheles quadriannulatus AQUA002542
Anopheles coluzzii ACOM027720
Anopheles merus AMEM017950
Anopheles melas AMEC002941
Anopheles arabiensis AARA008811
Anopheles christyi ACHR007662
Anopheles epiroticus AEPI007044
Anopheles culicifacies ACUA005369
Anopheles minimus AMIN009284
Anopheles funestus AFUN010488
Anopheles stephensi ASTE000093
Anopheles maculatus AMAM015239
Anopheles dirus ADIR005234
Anopheles farauti AFAF012712
Anopheles albimanus AALB001258
Anopheles darlingi ADAC004744
Anopheles sinensis ASIC003887
Anopheles atroparvus AATE007217
Aedes aegypti AAEL018354
Culex quinquefasciatus CPIJ005418
Polypedilum nubifer Pn.03637
Polypedilum vanderplanki Pv.08250
Leptinotarsa decemlineata LDEC007863
Calligrapha
Anoplophora glabripennis AGLA006344
Dendroctonus ponderosae YQE 04661
Rhyzobius
Byturus
Thanasimus
Bitoma
Tribolium castaneum TC006108
Meloe
Cyphon
Xylobiops
Agrilus planipennis APLA011971
Lamprohiza
Hydrochara
Onthophagus taurus OTAU011751
Cetonia
Ocypus
Cybister
Aspidytes
Cicindela
Gyrinus
Hydroscapha
Micromalthus
Mengenilla moldrzyki mesp 3644
Heliconius melpomene HMEL008335
Danaus plexippus DPOGS201779 PA
Melitaea cinxia MCINX000880
Manduca sexta Msex2.11361
Bombyx mori BGIBMGA013142
Papilio glaucus pgl933.13
Plutella xylostella Px002282
Limnephilus lunatus LLUN007429
Acromyrmex echinatior AECH11620
Atta cephalotes ACEP13193
Wasmannia auropunctata 23046558
Cardiocondyla obscurior Cobs 15532
Monomorium pharaonis 2657134404
Solenopsis invicta SINV17712
Vollenhovia emeryi 19037081
Pogonomyrmex barbatus PB14699
Cerapachys biroi EZA57727.1
Camponotus floridanus CFLO17223
Linepithema humile LH25115
Harpegnathos saltator HSAL15828
Apis mellifera GB42559
Apis cerana ACSNU09116T0
Apis dorsata 51583633
Apis florea 2225325124
Bombus impatiens Bimp 2.0
Bombus terrestris 15205882
Melipona quadrifasciata MQUAD 001374
Eufriesea mexicana EMEXI 00033f
Habropoda laboriosa HLABO 0025af
Megachile rotundata MROTU 00142c
Dufourea novaeangliae DNOVA 000fb7
Lasioglossum albipes Lalb 02560
Polistes dominula 4376
Trichogramma pretiosum TPRE003462
Copidosoma floridanum CFLO008824
Nasonia vitripennis Nasvi2EG014429
Cephus cinctus CCINC 00054d
Fopius arisanus 6705936
Orussus abietinus OABI001789
Athalia rosae AROS006760
Diaphorina citri ref0143003.1
Pachypsylla venusta PVEN008820
Acyrthosiphon pisum ACYPI003141
Pediculus humanus PHUM240840
Frankliniella occidentalis FOCC010161
Halyomorpha halys HHAL003090
Oncopeltus fasciatus OFAS017451
Rhodnius prolixus RPRC001739
Cimex lectularius CLEC000929
Gerris buenoi GBUE000009
Homalodisca vitripennis HVIT011108
Blattella germanica BGER007913
Zootermopsis nevadensis Znev 09918
Ladona fulva LFUL015807
Calopteryx splendens size58871
Ephemera danica EDAN007214
Diptera
Lepidoptera
Trichoptera
Coleoptera
Strepsiptera
Hymenoptera
Psocodea
Thysanoptera
Hemiptera
Blattodea

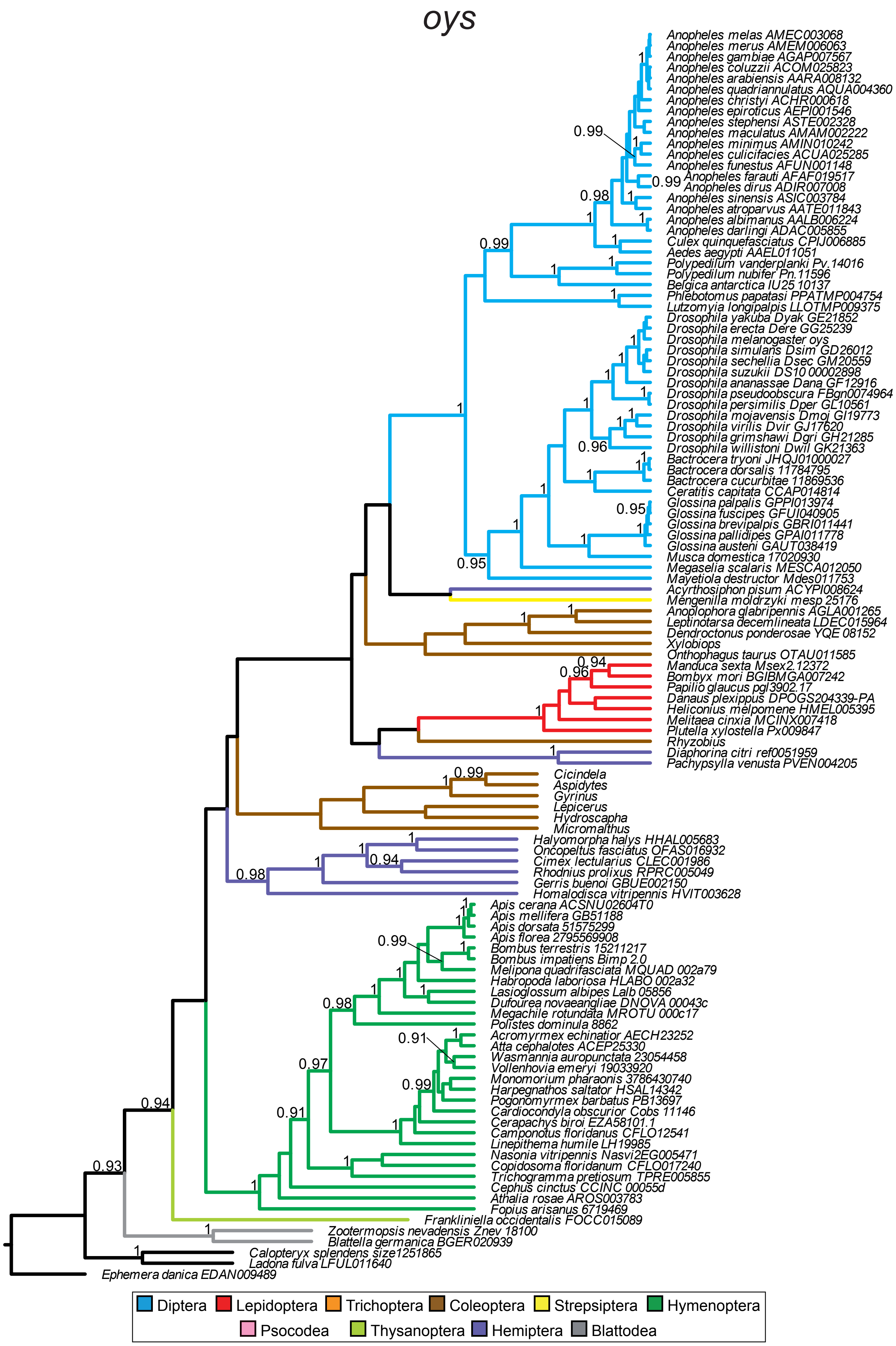
oys
Anopheles melas AMEC003068
Anopheles merus AMEM006063
Anopheles gambiae AGAP007567
Anopheles coluzzii ACOM025823
Anopheles arabiensis AARA008132
Anopheles quadriannulatus AQUA004360
Anopheles christyi ACHR000618
Anopheles epiroticus AEPI001546
Anopheles stephensi ASTE002328
Anopheles maculatus AMAM002222
Anopheles minimus AMIN010242
Anopheles culicifacies ACUA025285
Anopheles funestus AFUN001148
Anopheles farauti AFAF019517
Anopheles dirus ADIR007008
Anopheles sinensis ASIC003784
Anopheles atroparvus AATE011843
Anopheles albimanus AALB006224
Anopheles darlingi ADAC005855
Culex quinquefasciatus CPIJ006885
Aedes aegypti AAEL011051
Polypedilum vanderplanki Pv.14016
Polypedilum nubifer Pn.11596
Belgica antarctica IU25 10137
Phlebotomus papatasi PPATMP004754
Lutzomyia longipalpis LLOTMP009375
Drosophila yakuba Dyak GE21852
Drosophila erecta Dere GG25239
Drosophila melanogaster oys
Drosophila simulans Dsim GD26012
Drosophila sechellia Dsec GM20559
Drosophila suzukii DS10 00002898
Drosophila ananassae Dana GF12916
Drosophila pseudoobscura FBgn0074964
Drosophila persimilis Dper GL10561
Drosophila mojavensis Dmoj GI19773
Drosophila virilis Dvir GJ17620
Drosophila grimshawi Dgri GH21285
Drosophila willistoni Dwil GK21363
Bactrocera tryoni JHQJ01000027
Bactrocera dorsalis 11784795
Bactrocera cucurbitae 11869536
Ceratitis capitata CCAP014814
Glossina palpalis GPPI013974
Glossina fuscipes GFUI040905
Glossina brevipalpis GBRI011441
Glossina pallidipes GPAI011778
Glossina austeni GAUT038419
Musca domestica 17020930
Megaselia scalaris MESCA012050
Mayetiola destructor Mdes011753
Acyrthosiphon pisum ACYPI008624
Mengenilla moldrzyki mesp 25176
Anoplophora glabripennis AGLA001265
Leptinotarsa decemlineata LDEC015964
Dendroctonus ponderosae YQE 08152
Xylobiops
Onthophagus taurus OTAU011585
Manduca sexta Msex2.12372
Bombyx mori BGIBMGA007242
Papilio glaucus pgl3902.17
Danaus plexippus DPOGS204339-PA
Heliconius melpomene HMEL005395
Melitaea cinxia MCINX007418
Plutella xylostella Px009847
Rhyzobius
Diaphorina citri ref0051959
Pachypsylla venusta PVEN004205
Cicindela
Aspidytes
Gyrinus
Lepicerus
Hydroscapha
Micromalthus
Halyomorpha halys HHAL005683
Oncopeltus fasciatus OFAS016932
Cimex lectularius CLEC001986
Rhodnius prolixus RPRC005049
Gerris buenoi GBUE002150
Homalodisca vitripennis HVIT003628
Apis cerana ACSNU02604T0
Apis mellifera GB51188
Apis dorsata 51575299
Apis florea 2795569908
Bombus terrestris 15211217
Bombus impatiens Bimp 2.0
Melipona quadrifasciata MQUAD 002a79
Habropoda laboriosa HLABO 002a32
Lasioglossum albipes Lalb 05856
Dufourea novaeangliae DNOVA 00043c
Megachile rotundata MROTU 000c17
Polistes dominula 8862
Acromyrmex echinatior AECH23252
Atta cephalotes ACEP25330
Wasmannia auropunctata 23054458
Vollenhovia emeryi 19033920
Monomorium pharaonis 3786430740
Harpegnathos saltator HSAL14342
Pogonomyrmex barbatus PB13697
Cardiocondyla obscurior Cobs 11146
Cerapachys biroi EZA58101.1
Camponotus floridanus CFLO12541
Linepithema humile LH19985
Nasonia vitripennis Nasvi2EG005471
Copidosoma floridanum CFLO017240
Trichogramma pretiosum TPRE005855
Cephus cinctus CCINC 00055d
Athalia rosae AROS003783
Fopius arisanus 6719469
Frankliniella occidentalis FOCC015089
Zootermopsis nevadensis Znev 18100
Blattella germanica BGER020939
Calopteryx splendens size1251865
Ladona fulva LFUL011640
Ephemera danica EDAN009489
Diptera
Lepidoptera
Trichoptera
Coleoptera
Strepsiptera
Hymenoptera
Psocodea
Thysanoptera
Hemiptera
Blattodea

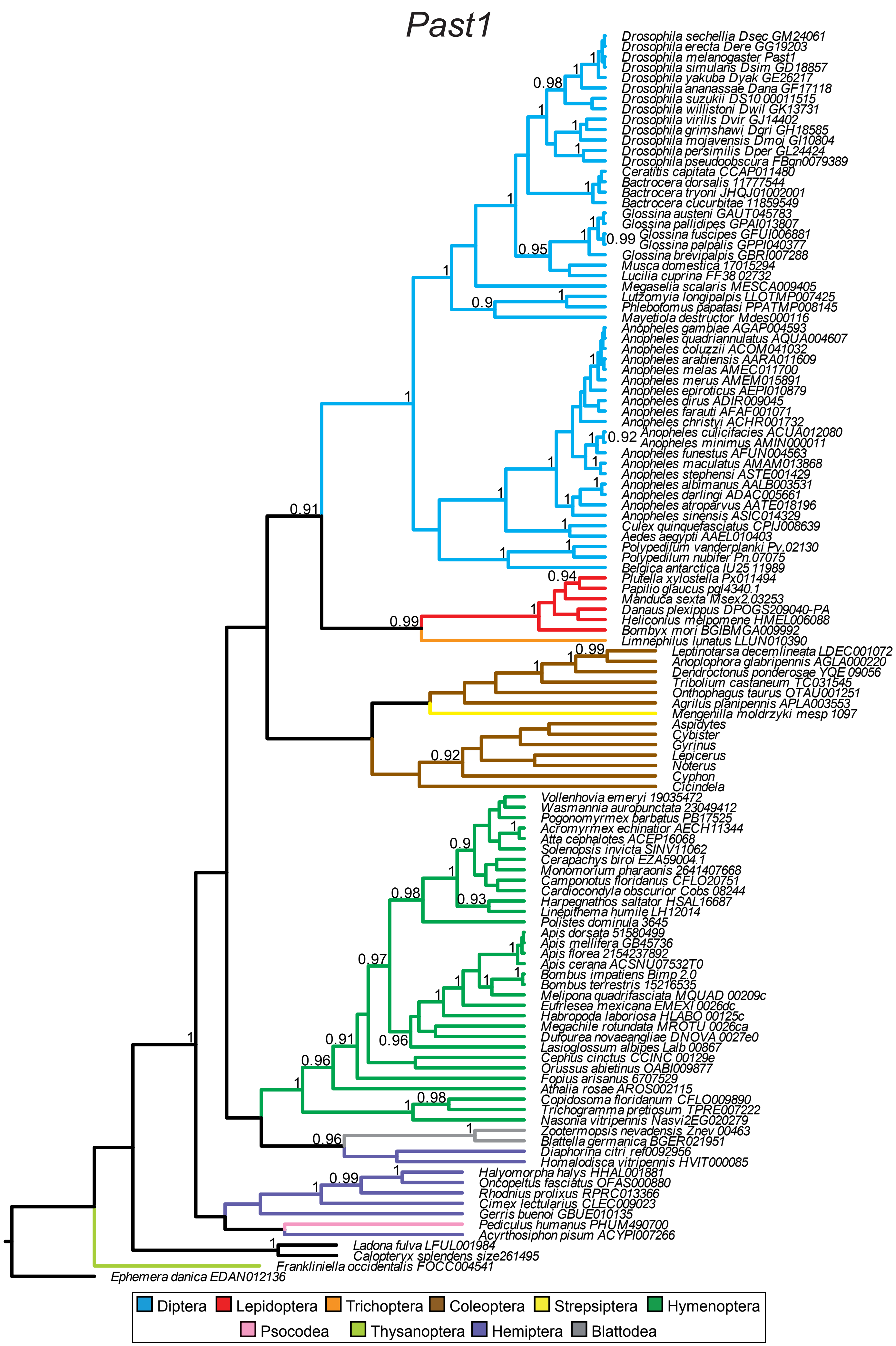
Past1
Drosophila sechellia Dsec GM24061
Drosophila erecta Dere GG19203
Drosophila melanogaster Past1
Drosophila simulans Dsim GD18857
Drosophila yakuba Dyak GE26217
Drosophila ananassae Dana GF17118
Drosophila suzukii DS10 00011515
Drosophila willistoni Dwil GK13731
Drosophila virilis Dvir GJ14402
Drosophila grimshawi Dgri GH18585
Drosophila mojavensis Dmoj GI10804
Drosophila persimilis Dper GL24424
Drosophila pseudoobscura FBgn0079389
Ceratitis capitata CCAP011480
Bactrocera dorsalis 11777544
Bactrocera tryoni JHQJ01002001
Bactrocera cucurbitae 11859549
Glossina austeni GAUT045783
Glossina pallidipes GPAI013807
Glossina fuscipes GFUI006881
Glossina palpalis GPPI040377
Glossina brevipalpis GBRI007288
Musca domestica 17015294
Lucilia cuprina FF38 02732
Megaselia scalaris MESCA009405
Lutzomyia longipalpis LLOTMP007425
Phlebotomus papatasi PPATMP008145
Mayetiola destructor Mdes000116
Anopheles gambiae AGAP004593
Anopheles quadriannulatus AQUA004607
Anopheles coluzzii ACOM041032
Anopheles arabiensis AARA011609
Anopheles melas AMEC011700
Anopheles merus AMEM015891
Anopheles epiroticus AEPI010879
Anopheles dirus ADIR009045
Anopheles farauti AFAF001071
Anopheles christyi ACHR001732
Anopheles culicifacies ACUA012080
Anopheles minimus AMIN000011
Anopheles funestus AFUN004563
Anopheles maculatus AMAM013868
Anopheles stephensi ASTE001429
Anopheles albimanus AALB003531
Anopheles darlingi ADAC005661
Anopheles atroparvus AATE018196
Anopheles sinensis ASIC014329
Culex quinquefasciatus CPIJ008639
Aedes aegypti AAEL010403
Polypedilum vanderplanki Pv.02130
Polypedilum nubifer Pn.07075
Belgica antarctica IU25 11989
Plutella xylostella Px011494
Papilio glaucus pgl4340.1
Manduca sexta Msex2.03253
Danaus plexippus DPOGS209040-PA
Heliconius melpomene HMEL006088
Bombyx mori BGIBMGA009992
Limnephilus lunatus LLUN010390
Leptinotarsa decemlineata LDEC001072
Anoplophora glabripennis AGLA000220
Dendroctonus ponderosae YQE 09056
Tribolium castaneum TC031545
Onthophagus taurus OTAU001251
Agrilus planipennis APLA003553
Mengenilla moldrzyki mesp 1097
Aspidytes
Cybister
Gyrinus
Lepicerus
Noterus
Cyphon
Cicindela
Vollenhovia emeryi 19035472
Wasmannia auropunctata 23049412
Pogonomyrmex barbatus PB17525
Acromyrmex echinatior AECH11344
Atta cephalotes ACEP16068
Solenopsis invicta SINV11062
Cerapachys biroi EZA59004.1
Monomorium pharaonis 2641407668
Camponotus floridanus CFLO20751
Cardiocondyla obscurior Cobs 08244
Harpegnathos saltator HSAL16687
Linepithema humile LH12014
Polistes dominula 3645
Apis dorsata 51580499
Apis mellifera GB45736
Apis florea 2154237892
Apis cerana ACSNU07532T0
Bombus impatiens Bimp 2.0
Bombus terrestris 15216535
Melipona quadrifasciata MQUAD 00209c
Eufriesea mexicana EMEXI 0026dc
Habropoda laboriosa HLABO 00125c
Megachile rotundata MROTU 0026ca
Dufourea novaeangliae DNOVA 0027e0
Lasioglossum albipes Lalb 00867
Cephus cinctus CCINC 00129e
Orussus abietinus OABI009877
Fopius arisanus 6707529
Athalia rosae AROS002115
Copidosoma floridanum CFLO009890
Trichogramma pretiosum TPRE007222
Nasonia vitripennis Nasvi2EG020279
Zootermopsis nevadensis Znev 00463
Blattella germanica BGER021951
Diaphorina citri ref0092956
Homalodisca vitripennis HVIT000085
Halyomorpha halys HHAL001881
Oncopeltus fasciatus OFAS000880
Rhodnius prolixus RPRC013366
Cimex lectularius CLEC009023
Gerris buenoi GBUE010135
Pediculus humanus PHUM490700
Acyrthosiphon pisum ACYPI007266
Ladona fulva LFUL001984
Calopteryx splendens size261495
Frankliniella occidentalis FOCC004541
Ephemera danica EDAN012136
Diptera
Lepidoptera
Trichoptera
Coleoptera
Strepsiptera
Hymenoptera
Psocodea
Thysanoptera
Hemiptera
Blattodea

Pen

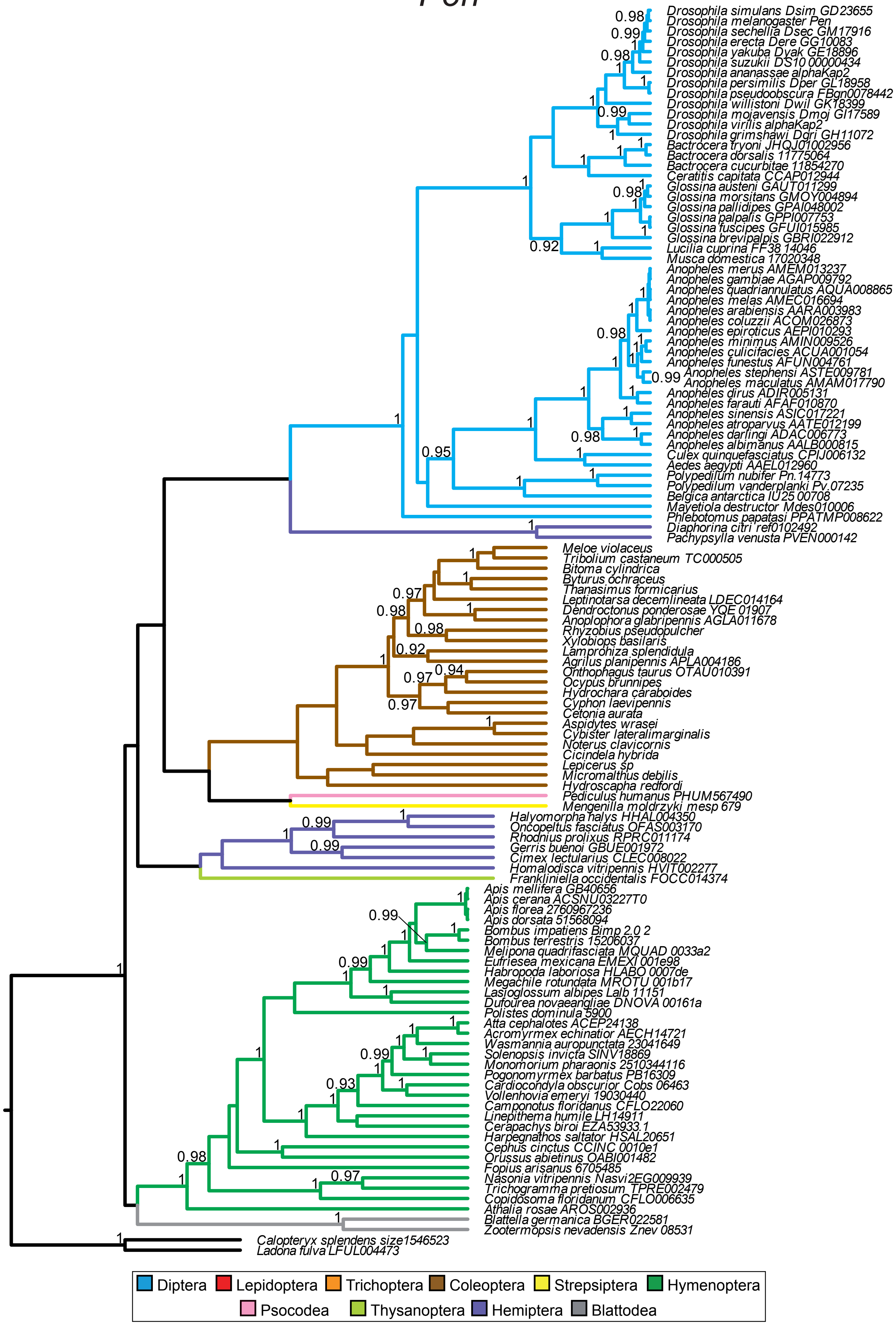
Drosophila simulans Dsim GD23655
Drosophila melanogaster Pen
Drosophila sechellia Dsec GM17916
Drosophila erecta Dere GG10083
Drosophila yakuba Dyak GE18896
Drosophila suzukii DS10 00000434
Drosophila ananassae alphaKap2
Drosophila persimilis Dper GL18958
Drosophila pseudoobscura FBgn0078442
Drosophila willistoni Dwil GK18399
Drosophila mojavensis Dmoj GI17589
Drosophila virilis alphaKap2
Drosophila grimshawi Dgri GH11072
Bactrocera tryoni JHQJ01002956
Bactrocera dorsalis 11775064
Bactrocera cucurbitae 11854270
Ceratitis capitata CCAP012944
Glossina austeni GAUT011299
Glossina morsitans GMOY004894
Glossina pallidipes GPAI048002
Glossina palpalis GPPI007753
Glossina fuscipes GFUI015985
Glossina brevipalpis GBRI022912
Lucilia cuprina FF38 14046
Musca domestica 17020348
Anopheles merus AMEM013237
Anopheles gambiae AGAP009792
Anopheles quadriannulatus AQUA008865
Anopheles melas AMEC016694
Anopheles arabiensis AARA003983
Anopheles coluzzii ACOM026873
Anopheles epiroticus AEPI010293
Anopheles minimus AMIN009526
Anopheles culicifacies ACUA001054
Anopheles funestus AFUN004761
Anopheles stephensi ASTE009781
Anopheles maculatus AMAM017790
Anopheles dirus ADIR005131
Anopheles farauti AFAF010870
Anopheles sinensis ASIC017221
Anopheles atroparvus AATE012199
Anopheles darlingi ADAC006773
Anopheles albimanus AALB000815
Culex quinquefasciatus CPIJ006132
Aedes aegypti AAEL012960
Polypedilum nubifer Pn.14773
Polypedilum vanderplanki Pv.07235
Belgica antarctica IU25 00708
Mayetiola destructor Mdes010006
Phlebotomus papatasi PPATMP008622
Diaphorina citri ref0102492
Pachypsylla venusta PVEN000142
Meloe violaceus
Tribolium castaneum TC000505
Bitoma cylindrica
Byturus ochraceus
Thanasimus formicarius
Leptinotarsa decemlineata LDEC014164
Dendroctonus ponderosae YQE 01907
Anoplophora glabripennis AGLA011678
Rhyzobius pseudopulcher
Xylobiops basilaris
Lamprohiza splendidula
Agrilus planipennis APLA004186
Onthophagus taurus OTAU010391
Ocypus brunnipes
Hydrochara caraboides
Cyphon laevipennis
Cetonia aurata
Aspidytes wrasei
Cybister lateralimarginalis
Noterus clavicornis
Cicindela hybrida
Lepicerus sp
Micromalthus debilis
Hydroscapha redfordi
Pediculus humanus PHUM567490
Mengenilla moldrzyki mesp 679
Halyomorpha halys HHAL004350
Oncopeltus fasciatus OFAS003170
Rhodnius prolixus RPRC011174
Gerris buenoi GBUE001972
Cimex lectularius CLEC008022
Homalodisca vitripennis HVIT002277
Frankliniella occidentalis FOCC014374
Apis mellifera GB40656
Apis cerana ACSNU03227T0
Apis florea 2760967236
Apis dorsata 51568094
Bombus impatiens Bimp 2.0 2
Bombus terrestris 15206037
Melipona quadrifasciata MQUAD 0033a2
Eufriesea mexicana EMEXI 001e98
Habropoda laboriosa HLABO 0007de
Megachile rotundata MROTU 001b17
Lasioglossum albipes Lalb 11151
Dufourea novaeangliae DNOVA 00161a
Polistes dominula 5900
Atta cephalotes ACEP24138
Acromyrmex echinatior AECH14721
Wasmannia auropunctata 23041649
Solenopsis invicta SINV18869
Monomorium pharaonis 2510344116
Pogonomyrmex barbatus PB16309
Cardiocondyla obscurior Cobs 06463
Vollenhovia emeryi 19030440
Camponotus floridanus CFLO22060
Linepithema humile LH14911
Cerapachys biroi EZA53933.1
Harpegnathos saltator HSAL20651
Cephus cinctus CCINC 0010e1
Orussus abietinus OABI001482
Fopius arisanus 6705485
Nasonia vitripennis Nasvi2EG009939
Trichogramma pretiosum TPRE002479
Copidosoma floridanum CFLO006635
Athalia rosae AROS002936
Blattella germanica BGER022581
Zootermopsis nevadensis Znev 08531
Calopteryx splendens size1546523
Ladona fulva LFUL004473
Diptera
Lepidoptera
Trichoptera
Coleoptera
Strepsiptera
Hymenoptera
Psocodea
Thysanoptera
Hemiptera
Blattodea

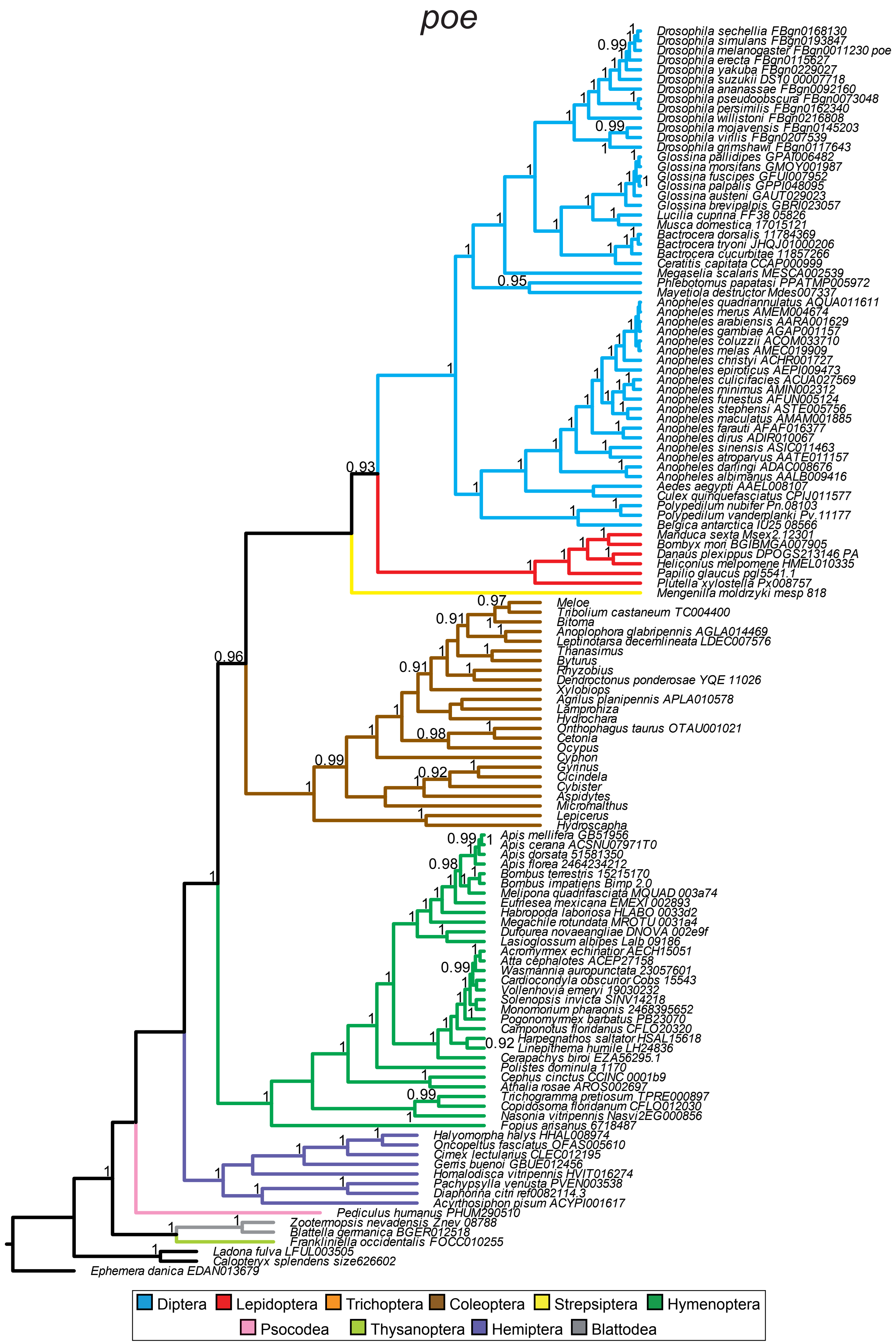
poe
Drosophila sechellia FBgn0168130
Drosophila simulans FBgn0193847
Drosophila melanogaster FBgn0011230 poe
Drosophila erecta FBgn0115627
Drosophila yakuba FBgn0229027
Drosophila suzukii DS10 00007718
Drosophila ananassae FBgn0092160
Drosophila pseudoobscura FBgn0073048
Drosophila persimilis FBgn0162340
Drosophila willistoni FBgn0216808
Drosophila mojavensis FBgn0145203
Drosophila virilis FBgn0207539
Drosophila grimshawi FBgn0117643
Glossina pallidipes GPAI006482
Glossina morsitans GMOY001987
Glossina fuscipes GFUI007952
Glossina palpalis GPPI048095
Glossina austeni GAUT029023
Glossina brevipalpis GBRI023057
Lucilia cuprina FF38 05826
Musca domestica 17015121
Bactrocera dorsalis 11784369
Bactrocera tryoni JHQJ01000206
Bactrocera cucurbitae 11857266
Ceratitis capitata CCAP000999
Megaselia scalaris MESCA002539
Phlebotomus papatasi PPATMP005972
Mayetiola destructor Mdes007337
Anopheles quadriannulatus AQUA011611
Anopheles merus AMEM004674
Anopheles arabiensis AARA001629
Anopheles gambiae AGAP001157
Anopheles coluzzii ACOM033710
Anopheles melas AMEC019909
Anopheles christyi ACHR001727
Anopheles epiroticus AEPI009473
Anopheles culicifacies ACUA027569
Anopheles minimus AMIN002312
Anopheles funestus AFUN005124
Anopheles stephensi ASTE005756
Anopheles maculatus AMAM001885
Anopheles farauti AFAF016377
Anopheles dirus ADIR010067
Anopheles sinensis ASIC011463
Anopheles atroparvus AATE011157
Anopheles darlingi ADAC008676
Anopheles albimanus AALB009416
Aedes aegypti AAEL008107
Culex quinquefasciatus CPIJ011577
Polypedilum nubifer Pn.08103
Polypedilum vanderplanki Pv.11177
Belgica antarctica IU25 08566
Manduca sexta Msex2.12301
Bombyx mori BGIBMGA007905
Danaus plexippus DPOGS213146 PA
Heliconius melpomene HMEL010335
Papilio glaucus pgl5541.1
Plutella xylostella Px008757
Mengenilla moldrzyki mesp 818
Meloe
Tribolium castaneum TC004400
Bitoma
Anoplophora glabripennis AGLA014469
Leptinotarsa decemlineata LDEC007576
Thanasimus
Byturus
Rhyzobius
Dendroctonus ponderosae YQE 11026
Xylobiops
Agrilus planipennis APLA010578
Lamprohiza
Hydrochara
Onthophagus taurus OTAU001021
Cetonia
Ocypus
Cyphon
Gyrinus
Cicindela
Cybister
Aspidytes
Micromalthus
Lepicerus
Hydroscapha
Apis mellifera GB51956
Apis cerana ACSNU07971T0
Apis dorsata 51581350
Apis florea 2464234212
Bombus terrestris 15215170
Bombus impatiens Bimp 2.0
Melipona quadrifasciata MQUAD 003a74
Eufriesea mexicana EMEXI 002893
Habropoda laboriosa HLABO 0033d2
Megachile rotundata MROTU 0031a4
Dufourea novaeangliae DNOVA 002e9f
Lasioglossum albipes Lalb 09186
Acromyrmex echinatior AECH15051
Atta cephalotes ACEP27158
Wasmannia auropunctata 23057601
Cardiocondyla obscurior Cobs 15543
Vollenhovia emeryi 19030232
Solenopsis invicta SINV14218
Monomorium pharaonis 2468395652
Pogonomyrmex barbatus PB23070
Camponotus floridanus CFLO20320
Harpegnathos saltator HSAL15618
Linepithema humile LH24836
Cerapachys biroi EZA56295.1
Polistes dominula 1170
Cephus cinctus CCINC 0001b9
Athalia rosae AROS002697
Trichogramma pretiosum TPRE000897
Copidosoma floridanum CFLO012030
Nasonia vitripennis Nasvi2EG000856
Fopius arisanus 6718487
Halyomorpha halys HHAL008974
Oncopeltus fasciatus OFAS005610
Cimex lectularius CLEC012195
Gerris buenoi GBUE012456
Homalodisca vitripennis HVIT016274
Pachypsylla venusta PVEN003538
Diaphorina citri ref0082114.3
Acyrthosiphon pisum ACYPI001617
Pediculus humanus PHUM290510
Zootermopsis nevadensis Znev 08788
Blattella germanica BGER012518
Frankliniella occidentalis FOCC010255
Ladona fulva LFUL003505
Calopteryx splendens size626602
Ephemera danica EDAN013679
Diptera
Lepidoptera
Trichoptera
Coleoptera
Strepsiptera
Hymenoptera
Psocodea
Thysanoptera
Hemiptera
Blattodea

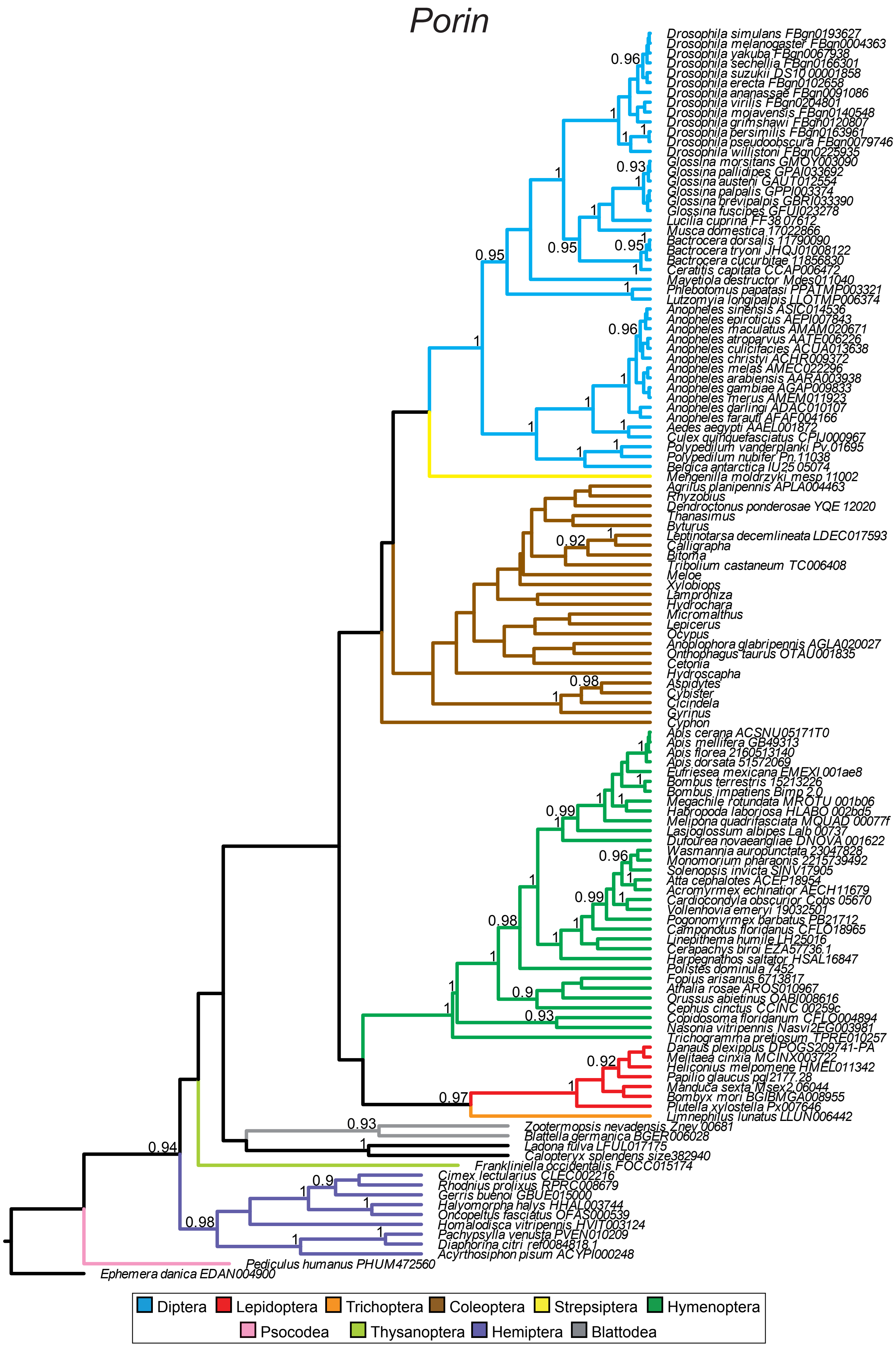
Porin
Drosophila simulans FBgn0193627
Drosophila melanogaster FBgn0004363
Drosophila yakuba FBgn0067938
Drosophila sechellia FBgn0166301
Drosophila suzukii DS10 00001858
Drosophila erecta FBgn0102658
Drosophila ananassae FBgn0091086
Drosophila virilis FBgn0204801
Drosophila mojavensis FBgn0140548
Drosophila grimshawi FBgn0120807
Drosophila persimilis FBgn0163961
Drosophila pseudoobscura FBgn0079746
Drosophila willistoni FBgn0225935
Glossina morsitans GMOY003090
Glossina pallidipes GPAI033692
Glossina austeni GAUT012554
Glossina palpalis GPPI003374
Glossina brevipalpis GBRI033390
Glossina fuscipes GFUI023278
Lucilia cuprina FF38 07612
Musca domestica 17022866
Bactrocera dorsalis 11790090
Bactrocera tryoni JHQJ01008122
Bactrocera cucurbitae 11856830
Ceratitis capitata CCAP006472
Mayetiola destructor Mdes011040
Phlebotomus papatasi PPATMP003321
Lutzomyia longipalpis LLOTMP006374
Anopheles sinensis ASIC014536
Anopheles epiroticus AEPI007843
Anopheles maculatus AMAM020671
Anopheles atroparvus AATE006226
Anopheles culicifacies ACUA013638
Anopheles christyi ACHR009372
Anopheles melas AMEC022296
Anopheles arabiensis AARA003938
Anopheles gambiae AGAP009833
Anopheles merus AMEM011923
Anopheles darlingi ADAC010107
Anopheles farauti AFAF004166
Aedes aegypti AAEL001872
Culex quinquefasciatus CPIJ000967
Polypedilum vanderplanki Pv.01695
Polypedilum nubifer Pn.11038
Belgica antarctica IU25 05074
Mengenilla moldrzyki mesp 11002
Agrilus planipennis APLA004463
Rhyzobius
Dendroctonus ponderosae YQE 12020
Thanasimus
Byturus
Leptinotarsa decemlineata LDEC017593
Calligrapha
Bitoma
Tribolium castaneum TC006408
Meloe
Xylobiops
Lamprohiza
Hydrochara
Micromalthus
Lepicerus
Ocypus
Anoplophora glabripennis AGLA020027
Onthophagus taurus OTAU001835
Cetonia
Hydroscapha
Aspidytes
Cybister
Cicindela
Gyrinus
Cyphon
Apis cerana ACSNU05171T0
Apis mellifera GB49313
Apis florea 2160513140
Apis dorsata 51572069
Eufriesea mexicana EMEXI 001ae8
Bombus terrestris 15213226
Bombus impatiens Bimp 2.0
Megachile rotundata MROTU 001b06
Habropoda laboriosa HLABO 002bd5
Melipona quadrifasciata MQUAD 00077f
Lasioglossum albipes Lalb 00737
Dufourea novaeangliae DNOVA 001622
Wasmannia auropunctata 23047828
Monomorium pharaonis 2215739492
Solenopsis invicta SINV17905
Atta cephalotes ACEP18954
Acromyrmex echinatior AECH11679
Cardiocondyla obscurior Cobs 05670
Vollenhovia emeryi 19032501
Pogonomyrmex barbatus PB21712
Camponotus floridanus CFLO18965
Linepithema humile LH25016
Cerapachys biroi EZA57736.1
Harpegnathos saltator HSAL16847
Polistes dominula 7452
Fopius arisanus 6713817
Athalia rosae AROS010967
Orussus abietinus OABI008616
Cephus cinctus CCINC 00259c
Copidosoma floridanum CFLO004894
Nasonia vitripennis Nasvi2EG003981
Trichogramma pretiosum TPRE010257
Danaus plexippus DPOGS209741-PA
Melitaea cinxia MCINX003722
Heliconius melpomene HMEL011342
Papilio glaucus pgl2177.28
Manduca sexta Msex2.06044
Bombyx mori BGIBMGA008955
Plutella xylostella Px007646
Limnephilus lunatus LLUN006442
Zootermopsis nevadensis Znev 00681
Blattella germanica BGER006028
Ladona fulva LFUL017175
Calopteryx splendens size382940
Frankliniella occidentalis FOCC015174
Cimex lectularius CLEC002216
Rhodnius prolixus RPRC008679
Gerris buenoi GBUE015000
Halyomorpha halys HHAL003744
Oncopeltus fasciatus OFAS000539
Homalodisca vitripennis HVIT003124
Pachypsylla venusta PVEN010209
Diaphorina citri ref0084818.1
Acyrthosiphon pisum ACYPI000248
Pediculus humanus PHUM472560
Ephemera danica EDAN004900
Diptera
Lepidoptera
Trichoptera
Coleoptera
Strepsiptera
Hymenoptera
Psocodea
Thysanoptera
Hemiptera
Blattodea

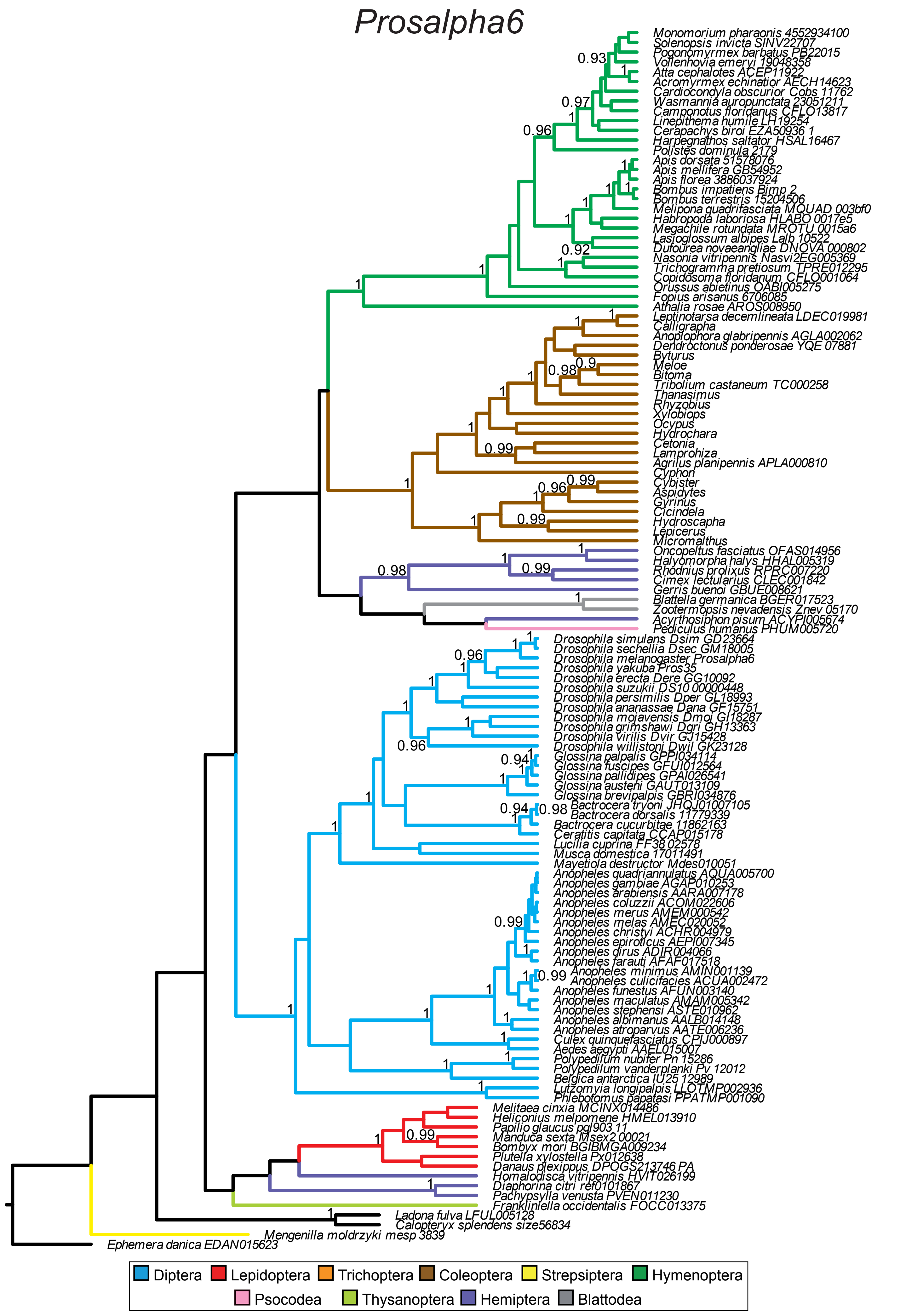
Prosalpha6
Monomorium pharaonis 4552934100
Solenopsis invicta SINV22707
Pogonomyrmex barbatus PB22015
Vollenhovia emeryi 19048358
Atta cephalotes ACEP11922
Acromyrmex echinatior AECH14623
Cardiocondyla obscurior Cobs 11762
Wasmannia auropunctata 23051211
Camponotus floridanus CFLO13817
Linepithema humile LH19254
Cerapachys biroi EZA50936 1
Harpegnathos saltator HSAL16467
Polistes dominula 2179
Apis dorsata 51578076
Apis mellifera GB54952
Apis florea 3886037924
Bombus impatiens Bimp 2
Bombus terrestris 15204506
Melipona quadrifasciata MQUAD 003bf0
Habropoda laboriosa HLABO 0017e5
Megachile rotundata MROTU 0015a6
Lasioglossum albipes Lalb 10522
Dufourea novaeangliae DNOVA 000802
Nasonia vitripennis Nasvi2EG005369
Trichogramma pretiosum TPRE012295
Copidosoma floridanum CFLO001064
Orussus abietinus OABI005275
Fopius arisanus 6706085
Athalia rosae AROS008950
Leptinotarsa decemlineata LDEC019981
Calligrapha
Anoplophora glabripennis AGLA002062
Dendroctonus ponderosae YQE 07881
Byturus
Meloe
Bitoma
Tribolium castaneum TC000258
Thanasimus
Rhyzobius
Xylobiops
Ocypus
Hydrochara
Cetonia
Lamprohiza
Agrilus planipennis APLA000810
Cyphon
Cybister
Aspidytes
Gyrinus
Cicindela
Hydroscapha
Lepicerus
Micromalthus
Oncopeltus fasciatus OFAS014956
Halyomorpha halys HHAL005319
Rhodnius prolixus RPRC007220
Cimex lectularius CLEC001842
Gerris buenoi GBUE008621
Blattella germanica BGER017523
Zootermopsis nevadensis Znev 05170
Acyrthosiphon pisum ACYPI005674
Pediculus humanus PHUM005720
Drosophila simulans Dsim GD23664
Drosophila sechellia Dsec GM18005
Drosophila melanogaster Prosalpha6
Drosophila yakuba Pros35
Drosophila erecta Dere GG10092
Drosophila suzukii DS10 00000448
Drosophila persimilis Dper GL18993
Drosophila ananassae Dana GF15751
Drosophila mojavensis Dmoj GI18287
Drosophila grimshawi Dgri GH13363
Drosophila virilis Dvir GJ15428
Drosophila willistoni Dwil GK23128
Glossina palpalis GPPI034114
Glossina fuscipes GFUI012564
Glossina pallidipes GPAI026541
Glossina austeni GAUT013109
Glossina brevipalpis GBRI034876
Bactrocera tryoni JHQJ01007105
Bactrocera dorsalis 11779339
Bactrocera cucurbitae 11862163
Ceratitis capitata CCAP015178
Lucilia cuprina FF38 02578
Musca domestica 17011491
Mayetiola destructor Mdes010051
Anopheles quadriannulatus AQUA005700
Anopheles gambiae AGAP010253
Anopheles arabiensis AARA007178
Anopheles coluzzii ACOM022606
Anopheles merus AMEM000542
Anopheles melas AMEC020052
Anopheles christyi ACHR004979
Anopheles epiroticus AEPI007345
Anopheles dirus ADIR004066
Anopheles farauti AFAF017518
Anopheles minimus AMIN001139
Anopheles culicifacies ACUA002472
Anopheles funestus AFUN003140
Anopheles maculatus AMAM005342
Anopheles stephensi ASTE010962
Anopheles albimanus AALB014148
Anopheles atroparvus AATE006236
Culex quinquefasciatus CPIJ000897
Aedes aegypti AAEL015007
Polypedilum nubifer Pn 15286
Polypedilum vanderplanki Pv 12012
Belgica antarctica IU25 12989
Lutzomyia longipalpis LLOTMP002936
Phlebotomus papatasi PPATMP001090
Melitaea cinxia MCINX014486
Heliconius melpomene HMEL013910
Papilio glaucus pgl903 11
Manduca sexta Msex2 00021
Bombyx mori BGIBMGA009234
Plutella xylostella Px012638
Danaus plexippus DPOGS213746 PA
Homalodisca vitripennis HVIT026199
Diaphorina citri ref0101867
Pachypsylla venusta PVEN011230
Frankliniella occidentalis FOCC013375
Ladona fulva LFUL005128
Calopteryx splendens size56834
Mengenilla moldrzyki mesp 3839
Ephemera danica EDAN015623
Diptera
Lepidoptera
Trichoptera
Coleoptera
Strepsiptera
Hymenoptera
Psocodea
Thysanoptera
Hemiptera
Blattodea

scat

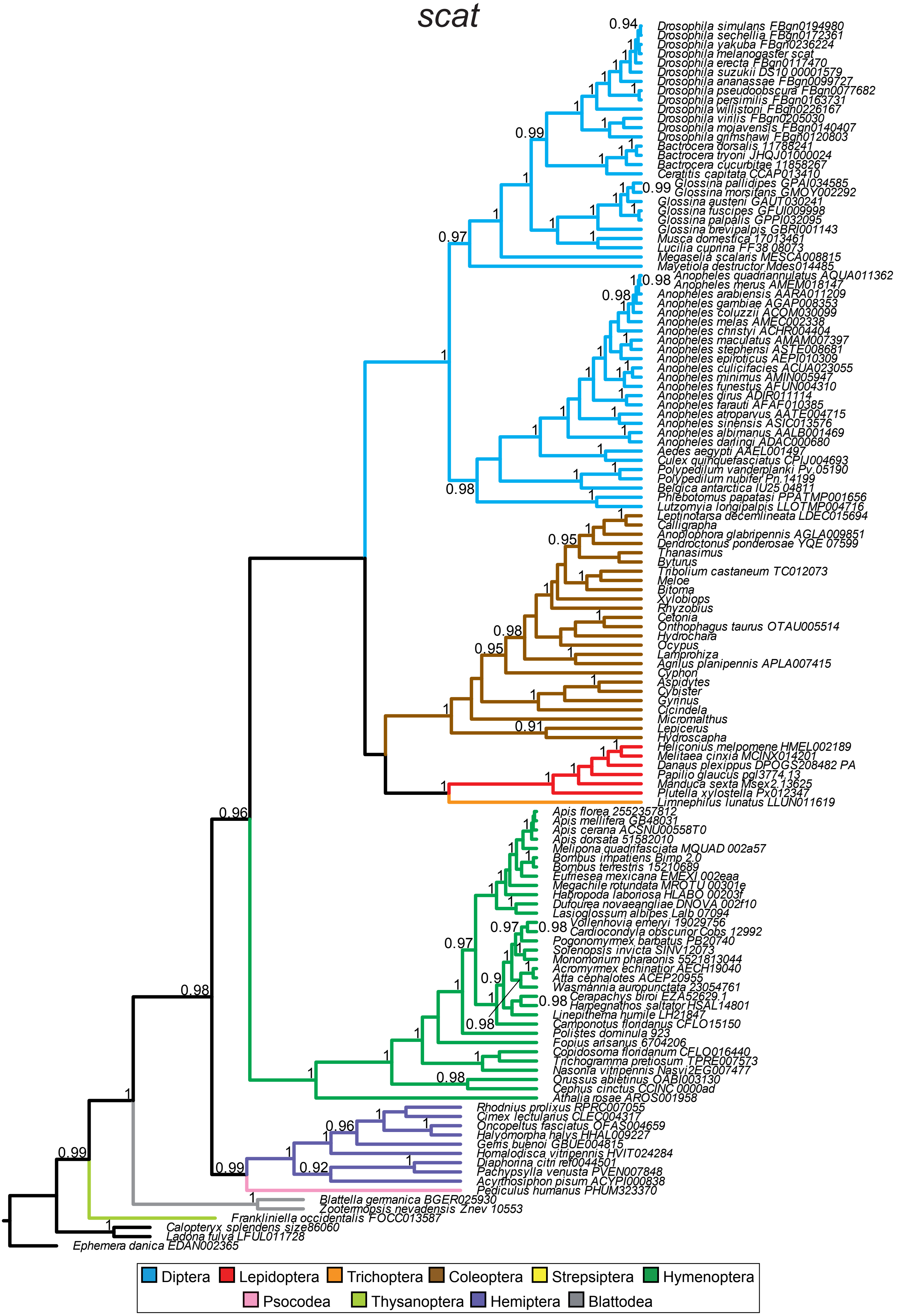

Drosophila simulans FBgn0194980
Drosophila sechellia FBgn0172361
Drosophila yakuba FBgn0236224
Drosophila melanogaster scat
Drosophila erecta FBgn0117470
Drosophila suzukii DS10 00001579
Drosophila ananassae FBgn0099727
Drosophila pseudoobscura FBgn0077682
Drosophila persimilis FBgn0163731
Drosophila willistoni FBgn0226167
Drosophila virilis FBgn0205030
Drosophila mojavensis FBgn0140407
Drosophila grimshawi FBgn0120803
Bactrocera dorsalis 11788241
Bactrocera tryoni JHQJ01000024
Bactrocera cucurbitae 11858267
Ceratitis capitata CCAP013410
Glossina pallidipes GPAI034585
Glossina morsitans GMOY002292
Glossina austeni GAUT030241
Glossina fuscipes GFUI009998
Glossina palpalis GPPI032095
Glossina brevipalpis GBRI001143
Musca domestica 17013461
Lucilia cuprina FF38 08073
Megaselia scalaris MESCA008815
Mayetiola destructor Mdes014485
Anopheles quadriannulatus AQUA011362
Anopheles merus AMEM018147
Anopheles arabiensis AARA011209
Anopheles gambiae AGAP008353
Anopheles coluzzii ACOM030099
Anopheles melas AMEC002338
Anopheles christyi ACHR004404
Anopheles maculatus AMAM007397
Anopheles stephensi ASTE008681
Anopheles epiroticus AEPI010309
Anopheles culicifacies ACUA023055
Anopheles minimus AMIN005947
Anopheles funestus AFUN004310
Anopheles dirus ADIR011114
Anopheles farauti AFAF010385
Anopheles atroparvus AATE004715
Anopheles sinensis ASIC013576
Anopheles albimanus AALB001469
Anopheles darlingi ADAC000680
Aedes aegypti AAEL001497
Culex quinquefasciatus CPIJ004693
Polypedilum vanderplanki Pv.05190
Polypedilum nubifer Pn.14199
Belgica antarctica IU25 04811
Phlebotomus papatasi PPATMP001656
Lutzomyia longipalpis LLOTMP004716
Leptinotarsa decemlineata LDEC015694
Calligrapha
Anoplophora glabripennis AGLA009851
Dendroctonus ponderosae YQE 07599
Thanasimus
Byturus
Tribolium castaneum TC012073
Meloe
Bitoma
Xylobiops
Rhyzobius
Cetonia
Onthophagus taurus OTAU005514
Hydrochara
Ocypus
Lamprohiza
Agrilus planipennis APLA007415
Cyphon
Aspidytes
Cybister
Gyrinus
Cicindela
Micromalthus
Lepicerus
Hydroscapha
Heliconius melpomene HMEL002189
Melitaea cinxia MCINX014201
Danaus plexippus DPOGS208482 PA
Papilio glaucus pgl3774.13
Manduca sexta Msex2.13625
Plutella xylostella Px012347
Limnephilus lunatus LLUN011619
Apis florea 2552357812
Apis mellifera GB48031
Apis cerana ACSNU00558T0
Apis dorsata 51582010
Melipona quadrifasciata MQUAD 002a57
Bombus impatiens Bimp 2.0
Bombus terrestris 15210689
Eufriesea mexicana EMEXI 002eaa
Megachile rotundata MROTU 00301e
Habropoda laboriosa HLABO 00203f
Dufourea novaeangliae DNOVA 002f10
Lasioglossum albipes Lalb 07094
Vollenhovia emeryi 19029756
Cardiocondyla obscurior Cobs 12992
Pogonomyrmex barbatus PB20740
Solenopsis invicta SINV12073
Monomorium pharaonis 5521813044
Acromyrmex echinatior AECH19040
Atta cephalotes ACEP20955
Wasmannia auropunctata 23054761
Cerapachys biroi EZA52629.1
Harpegnathos saltator HSAL14801
Linepithema humile LH21847
Camponotus floridanus CFLO15150
Polistes dominula 923
Fopius arisanus 6704206
Copidosoma floridanum CFLO016440
Trichogramma pretiosum TPRE007573
Nasonia vitripennis Nasvi2EG007477
Orussus abietinus OABI003130
Cephus cinctus CCINC 0000ad
Athalia rosae AROS001958
Rhodnius prolixus RPRC007055
Cimex lectularius CLEC004317
Oncopeltus fasciatus OFAS004659
Halyomorpha halys HHAL009227
Gerris buenoi GBUE004815
Homalodisca vitripennis HVIT024284
Diaphorina citri ref0044501
Pachypsylla venusta PVEN007848
Acyrthosiphon pisum ACYPI000838
Pediculus humanus PHUM323370
Blattella germanica BGER025930
Zootermopsis nevadensis Znev 10553
Frankliniella occidentalis FOCC013587
Calopteryx splendens size86060
Ladona fulva LFUL011728
Ephemera danica EDAN002365
Diptera
Lepidoptera
Trichoptera
Coleoptera
Strepsiptera
Hymenoptera
Psocodea
Thysanoptera
Hemiptera
Blattodea

shi

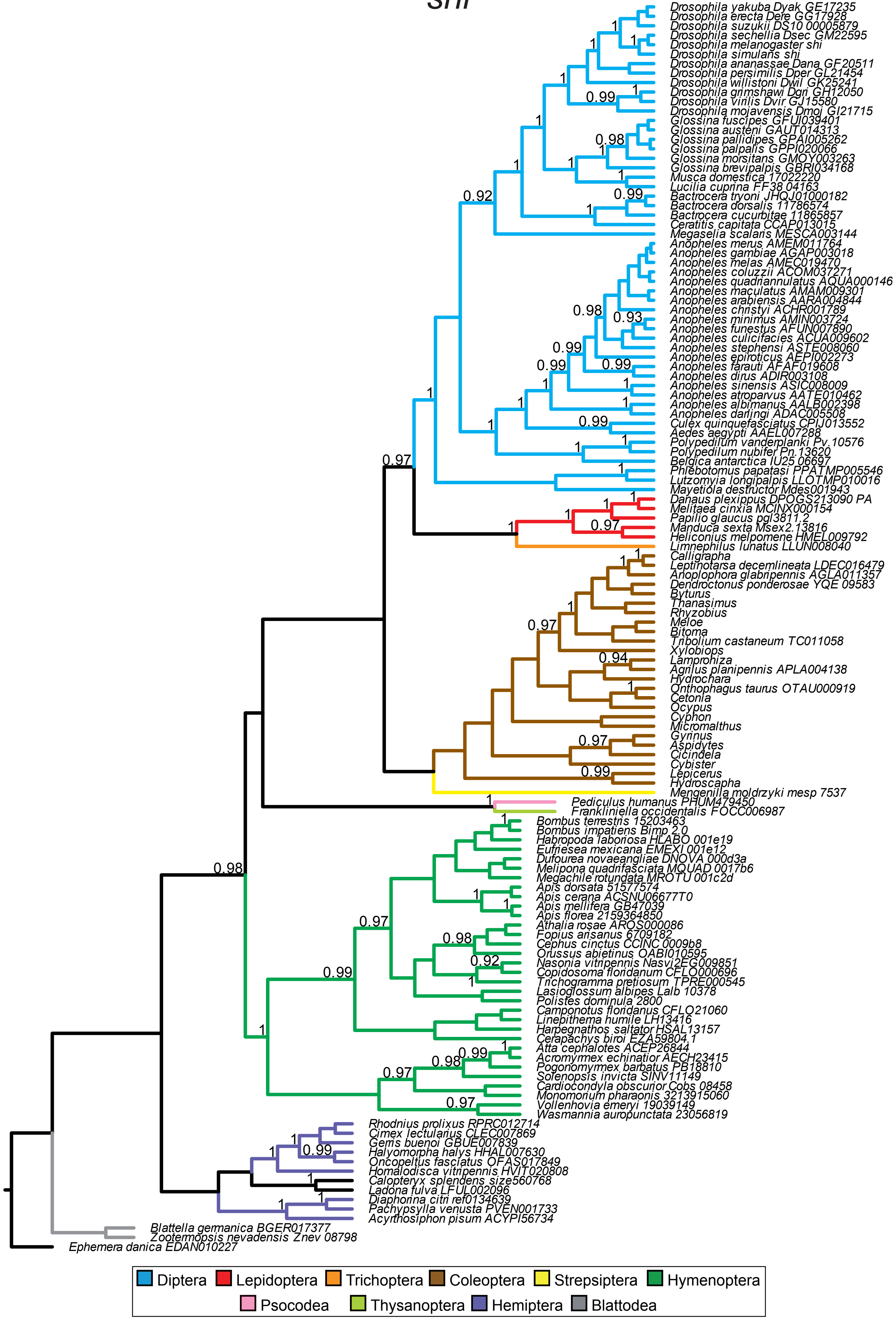
Drosophila yakuba Dyak GE17235
Drosophila erecta Dere GG17928
Drosophila suzukii DS10 00005879
Drosophila sechellia Dsec GM22595
Drosophila melanogaster shi
Drosophila simulans shi
Drosophila ananassae Dana GF20511
Drosophila persimilis Dper GL21454
Drosophila willistoni Dwil GK25241
Drosophila grimshawi Dgri GH12050
Drosophila virilis Dvir GJ15580
Drosophila mojavensis Dmoj GI21715
Glossina fuscipes GFUI039401
Glossina austeni GAUT014313
Glossina pallidipes GPAI005262
Glossina palpalis GPPI020066
Glossina morsitans GMOY003263
Glossina brevipalpis GBRI034168
Musca domestica 17022220
Lucilia cuprina FF38 04163
Bactrocera tryoni JHQJ01000182
Bactrocera dorsalis 11786574
Bactrocera cucurbitae 11865857
Ceratitis capitata CCAP013015
Megaselia scalaris MESCA003144
Anopheles merus AMEM011764
Anopheles gambiae AGAP003018
Anopheles melas AMEC019470
Anopheles coluzzii ACOM037271
Anopheles quadriannulatus AQUA000146
Anopheles maculatus AMAM009301
Anopheles arabiensis AARA004844
Anopheles christyi ACHR001789
Anopheles minimus AMIN003724
Anopheles funestus AFUN007890
Anopheles culicifacies ACUA009602
Anopheles stephensi ASTE008060
Anopheles epiroticus AEPI002273
Anopheles farauti AFAF019608
Anopheles dirus ADIR003108
Anopheles sinensis ASIC008009
Anopheles atroparvus AATE010462
Anopheles albimanus AALB002398
Anopheles darlingi ADAC005508
Culex quinquefasciatus CPIJ013552
Aedes aegypti AAEL007288
Polypedilum vanderplanki Pv.10576
Polypedilum nubifer Pn.13620
Belgica antarctica IU25 06697
Phlebotomus papatasi PPATMP005546
Lutzomyia longipalpis LLOTMP010016
Mayetiola destructor Mdes001943
Danaus plexippus DPOGS213090 PA
Melitaea cinxia MCINX000154
Papilio glaucus pgl3811.2
Manduca sexta Msex2.13816
Heliconius melpomene HMEL009792
Limnephilus lunatus LLUN008040
Calligrapha
Leptinotarsa decemlineata LDEC016479
Anoplophora glabripennis AGLA011357
Dendroctonus ponderosae YQE 09583
Byturus
Thanasimus
Rhyzobius
Meloe
Bitoma
Tribolium castaneum TC011058
Xylobiops
Lamprohiza
Agrilus planipennis APLA004138
Hydrochara
Onthophagus taurus OTAU000919
Cetonia
Ocypus
Cyphon
Micromalthus
Gyrinus
Aspidytes
Cicindela
Cybister
Lepicerus
Hydroscapha
Mengenilla moldrzyki mesp 7537
Pediculus humanus PHUM479450
Frankliniella occidentalis FOCC006987
Bombus terrestris 15203463
Bombus impatiens Bimp 2.0
Habropoda laboriosa HLABO 001e19
Eufriesea mexicana EMEXI 001e12
Dufourea novaeangliae DNOVA 000d3a
Melipona quadrifasciata MQUAD 0017b6
Megachile rotundata MROTU 001c2d
Apis dorsata 51577574
Apis cerana ACSNU06677T0
Apis mellifera GB47039
Apis florea 2159364850
Athalia rosae AROS000086
Fopius arisanus 6709182
Cephus cinctus CCINC 0009b8
Orussus abietinus OABI010595
Nasonia vitripennis Nasvi2EG009851
Copidosoma floridanum CFLO000696
Trichogramma pretiosum TPRE000545
Lasioglossum albipes Lalb 10378
Polistes dominula 2800
Camponotus floridanus CFLO21060
Linepithema humile LH13416
Harpegnathos saltator HSAL13157
Cerapachys biroi EZA59804.1
Atta cephalotes ACEP26844
Acromyrmex echinatior AECH23415
Pogonomyrmex barbatus PB18810
Solenopsis invicta SINV11149
Cardiocondyla obscurior Cobs 08458
Monomorium pharaonis 3213915060
Vollenhovia emeryi 19039149
Wasmannia auropunctata 23056819
Rhodnius prolixus RPRC012714
Cimex lectularius CLEC007869
Gerris buenoi GBUE007839
Halyomorpha halys HHAL007630
Oncopeltus fasciatus OFAS017849
Homalodisca vitripennis HVIT020808
Calopteryx splendens size560768
Ladona fulva LFUL002096
Diaphorina citri ref0134639
Pachypsylla venusta PVEN001733
Acyrthosiphon pisum ACYPI56734
Blattella germanica BGER017377
Zootermopsis nevadensis Znev 08798
Ephemera danica EDAN010227
Diptera
Lepidoptera
Trichoptera
Coleoptera
Strepsiptera
Hymenoptera
Psocodea
Thysanoptera
Hemiptera
Blattodea

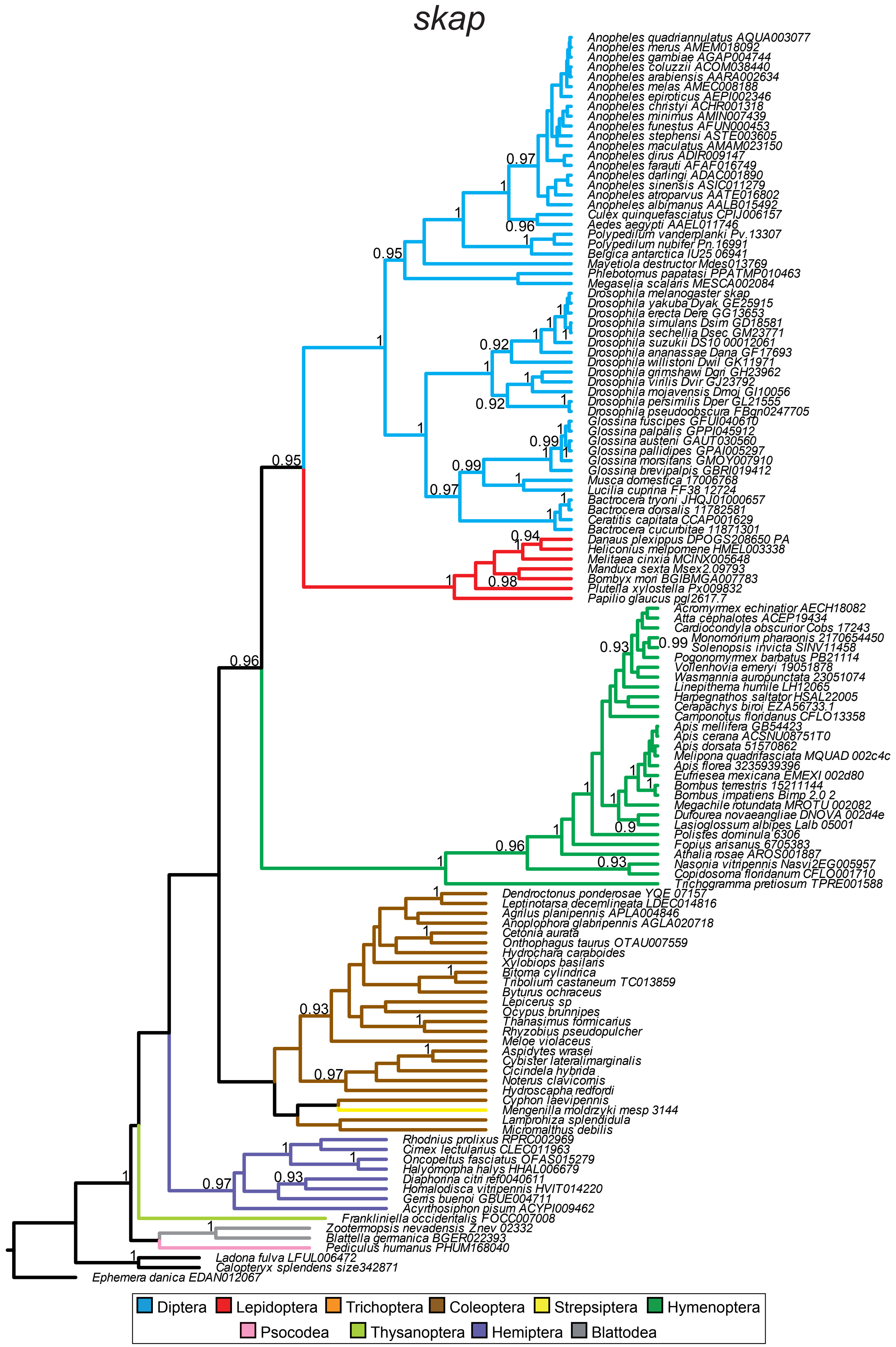
skap
Anopheles quadriannulatus AQUA003077
Anopheles merus AMEM018092
Anopheles gambiae AGAP004744
Anopheles coluzzii ACOM038440
Anopheles arabiensis AARA002634
Anopheles melas AMEC008188
Anopheles epiroticus AEPI002346
Anopheles christyi ACHR001318
Anopheles minimus AMIN007439
Anopheles funestus AFUN000453
Anopheles stephensi ASTE003605
Anopheles maculatus AMAM023150
Anopheles dirus ADIR009147
Anopheles farauti AFAF016749
Anopheles darlingi ADAC001890
Anopheles sinensis ASIC011279
Anopheles atroparvus AATE016802
Anopheles albimanus AALB015492
Culex quinquefasciatus CPIJ006157
Aedes aegypti AAEL011746
Polypedilum vanderplanki Pv.13307
Polypedilum nubifer Pn.16991
Belgica antarctica IU25 06941
Mayetiola destructor Mdes013769
Phlebotomus papatasi PPATMP010463
Megaselia scalaris MESCA002084
Drosophila melanogaster skap
Drosophila yakuba Dyak GE25915
Drosophila erecta Dere GG13653
Drosophila simulans Dsim GD18581
Drosophila sechellia Dsec GM23771
Drosophila suzukii DS10 00012061
Drosophila ananassae Dana GF17693
Drosophila willistoni Dwil GK11971
Drosophila grimshawi Dgri GH23962
Drosophila virilis Dvir GJ23792
Drosophila mojavensis Dmoj GI10056
Drosophila persimilis Dper GL21555
Drosophila pseudoobscura FBgn0247705
Glossina fuscipes GFUI040610
Glossina palpalis GPPI045912
Glossina austeni GAUT030560
Glossina pallidipes GPAI005297
Glossina morsitans GMOY007910
Glossina brevipalpis GBRI019412
Musca domestica 17006768
Lucilia cuprina FF38 12724
Bactrocera tryoni JHQJ01000657
Bactrocera dorsalis 11782581
Ceratitis capitata CCAP001629
Bactrocera cucurbitae 11871301
Danaus plexippus DPOGS208650 PA
Heliconius melpomene HMEL003338
Melitaea cinxia MCINX005648
Manduca sexta Msex2.09793
Bombyx mori BGIBMGA007783
Plutella xylostella Px009832
Papilio glaucus pgl2617.7
Acromyrmex echinatior AECH18082
Atta cephalotes ACEP19434
Cardiocondyla obscurior Cobs 17243
Monomorium pharaonis 2170654450
Solenopsis invicta SINV11458
Pogonomyrmex barbatus PB21114
Vollenhovia emeryi 19051878
Wasmannia auropunctata 23051074
Linepithema humile LH12065
Harpegnathos saltator HSAL22005
Cerapachys biroi EZA56733.1
Camponotus floridanus CFLO13358
Apis mellifera GB54423
Apis cerana ACSNU08751T0
Apis dorsata 51570862
Melipona quadrifasciata MQUAD 002c4c
Apis florea 3235939396
Eufriesea mexicana EMEXI 002d80
Bombus terrestris 15211144
Bombus impatiens Bimp 2.0 2
Megachile rotundata MROTU 002082
Dufourea novaeangliae DNOVA 002d4e
Lasioglossum albipes Lalb 05001
Polistes dominula 6306
Fopius arisanus 6705383
Athalia rosae AROS001887
Nasonia vitripennis Nasvi2EG005957
Copidosoma floridanum CFLO001710
Trichogramma pretiosum TPRE001588
Dendroctonus ponderosae YQE 07157
Leptinotarsa decemlineata LDEC014816
Agrilus planipennis APLA004846
Anoplophora glabripennis AGLA020718
Cetonia aurata
Onthophagus taurus OTAU007559
Hydrochara caraboides
Xylobiops basilaris
Bitoma cylindrica
Tribolium castaneum TC013859
Byturus ochraceus
Lepicerus sp
Ocypus brunnipes
Thanasimus formicarius
Rhyzobius pseudopulcher
Meloe violaceus
Aspidytes wrasei
Cybister lateralimarginalis
Cicindela hybrida
Noterus clavicornis
Hydroscapha redfordi
Cyphon laevipennis
Mengenilla moldrzyki mesp 3144
Lamprohiza splendidula
Micromalthus debilis
Rhodnius prolixus RPRC002969
Cimex lectularius CLEC011963
Oncopeltus fasciatus OFAS015279
Halyomorpha halys HHAL006679
Diaphorina citri ref0040611
Homalodisca vitripennis HVIT014220
Gerris buenoi GBUE004711
Acyrthosiphon pisum ACYPI009462
Frankliniella occidentalis FOCC007008
Zootermopsis nevadensis Znev 02332
Blattella germanica BGER022393
Pediculus humanus PHUM168040
Ladona fulva LFUL006472
Calopteryx splendens size342871
Ephemera danica EDAN012067
Diptera
Lepidoptera
Trichoptera
Coleoptera
Strepsiptera
Hymenoptera
Psocodea
Thysanoptera
Hemiptera
Blattodea

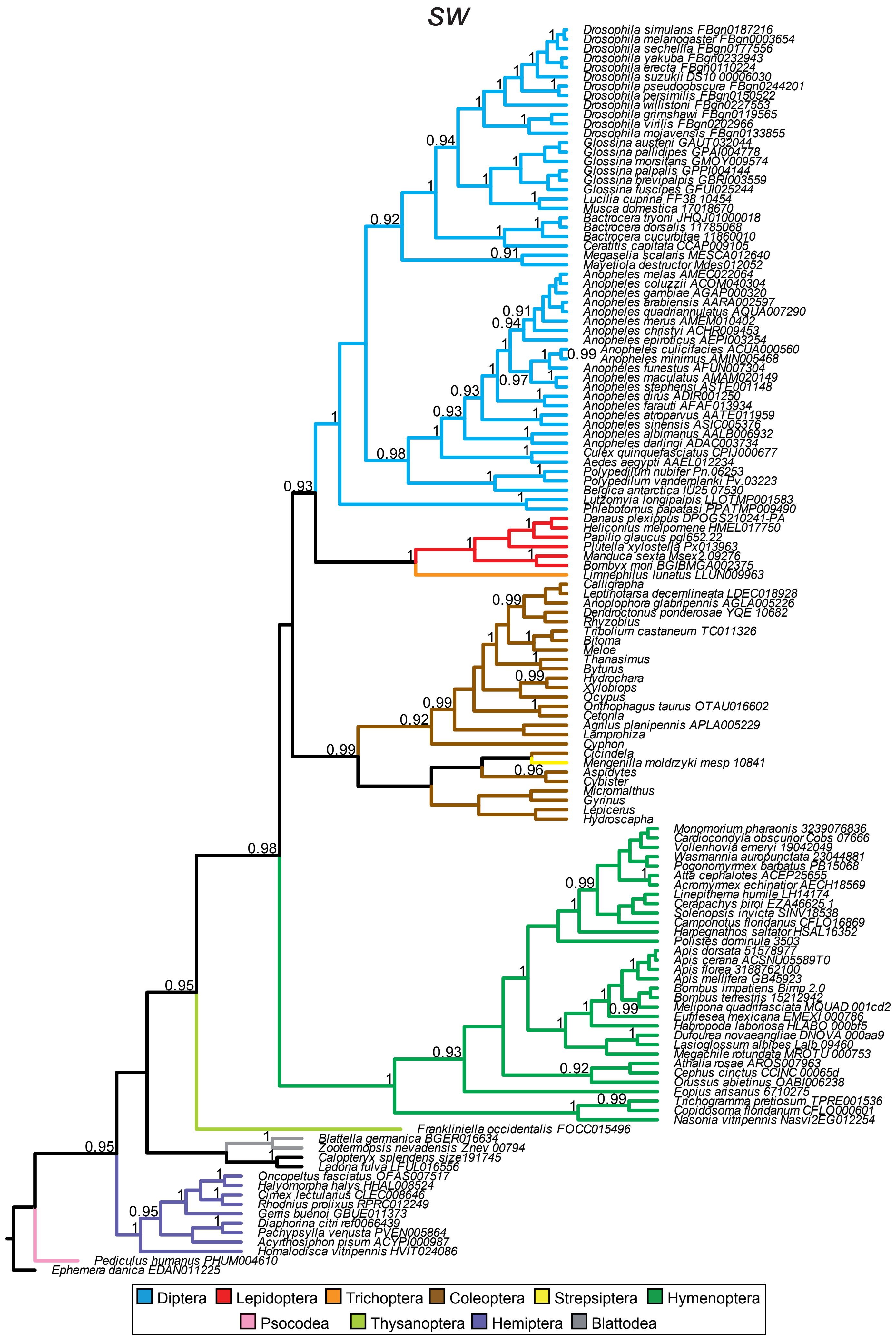
SW
Drosophila simulans FBgn0187216
Drosophila melanogaster FBgn0003654
Drosophila sechellia FBgn0177556
Drosophila yakuba FBgn0232943
Drosophila erecta FBgn0110224
Drosophila suzukii DS10 00006030
Drosophila pseudoobscura FBgn0244201
Drosophila persimilis FBgn0150522
Drosophila willistoni FBgn0227553
Drosophila grimshawi FBgn0119565
Drosophila virilis FBgn0202966
Drosophila mojavensis FBgn0133855
Glossina austeni GAUT032044
Glossina pallidipes GPAI004778
Glossina morsitans GMOY009574
Glossina palpalis GPPI004144
Glossina brevipalpis GBRI003559
Glossina fuscipes GFUI025244
Lucilia cuprina FF38 10454
Musca domestica 17018670
Bactrocera tryoni JHQJ01000018
Bactrocera dorsalis 11785068
Bactrocera cucurbitae 11860010
Ceratitis capitata CCAP009105
Megaselia scalaris MESCA012640
Mayetiola destructor Mdes012052
Anopheles melas AMEC022064
Anopheles coluzzii ACOM040304
Anopheles gambiae AGAP000320
Anopheles arabiensis AARA002597
Anopheles quadriannulatus AQUA007290
Anopheles merus AMEM010402
Anopheles christyi ACHR009453
Anopheles epiroticus AEPI003254
Anopheles culicifacies ACUA000560
Anopheles minimus AMIN005468
Anopheles funestus AFUN007304
Anopheles maculatus AMAM020149
Anopheles stephensi ASTE001148
Anopheles dirus ADIR001250
Anopheles farauti AFAF013934
Anopheles atroparvus AATE011959
Anopheles sinensis ASIC005376
Anopheles albimanus AALB006932
Anopheles darlingi ADAC003734
Culex quinquefasciatus CPIJ000677
Aedes aegypti AAEL012234
Polypedilum nubifer Pn.06253
Polypedilum vanderplanki Pv.03223
Belgica antarctica IU25 07530
Lutzomyia longipalpis LLOTMP001583
Phlebotomus papatasi PPATMP009490
Danaus plexippus DPOGS210241-PA
Heliconius melpomene HMEL017750
Papilio glaucus pgl652.22
Plutella xylostella Px013963
Manduca sexta Msex2.09276
Bombyx mori BGIBMGA002375
Limnephilus lunatus LLUN009963
Calligrapha
Leptinotarsa decemlineata LDEC018928
Anoplophora glabripennis AGLA005226
Dendroctonus ponderosae YQE 10682
Rhyzobius
Tribolium castaneum TC011326
Bitoma
Meloe
Thanasimus
Byturus
Hydrochara
Xylobiops
Ocypus
Onthophagus taurus OTAU016602
Cetonia
Agrilus planipennis APLA005229
Lamprohiza
Cyphon
Cicindela
Mengenilla moldrzyki mesp 10841
Aspidytes
Cybister
Micromalthus
Gyrinus
Lepicerus
Hydroscapha
Monomorium pharaonis 3239076836
Cardiocondyla obscurior Cobs 07666
Vollenhovia emeryi 19042049
Wasmannia auropunctata 23044881
Pogonomyrmex barbatus PB15068
Atta cephalotes ACEP25655
Acromyrmex echinatior AECH18569
Linepithema humile LH14174
Cerapachys biroi EZA46625.1
Solenopsis invicta SINV18538
Camponotus floridanus CFLO16869
Harpegnathos saltator HSAL16352
Polistes dominula 3503
Apis dorsata 51578977
Apis cerana ACSNU05589T0
Apis florea 3188762100
Apis mellifera GB45923
Bombus impatiens Bimp 2.0
Bombus terrestris 15212942
Melipona quadrifasciata MQUAD 001cd2
Eufriesea mexicana EMEXI 000786
Habropoda laboriosa HLABO 000bf5
Dufourea novaeangliae DNOVA 000aa9
Lasioglossum albipes Lalb 09460
Megachile rotundata MROTU 000753
Athalia rosae AROS007963
Cephus cinctus CCINC 00065d
Orussus abietinus OABI006238
Fopius arisanus 6710275
Trichogramma pretiosum TPRE001536
Copidosoma floridanum CFLO000601
Nasonia vitripennis Nasvi2EG012254
Frankliniella occidentalis FOCC015496
Blattella germanica BGER016634
Zootermopsis nevadensis Znev 00794
Calopteryx splendens size191745
Ladona fulva LFUL016556
Oncopeltus fasciatus OFAS007517
Halyomorpha halys HHAL008524
Cimex lectularius CLEC008646
Rhodnius prolixus RPRC012249
Gerris buenoi GBUE011373
Diaphorina citri ref0066439
Pachypsylla venusta PVEN005864
Acyrthosiphon pisum ACYPI000987
Homalodisca vitripennis HVIT024086
Pediculus humanus PHUM004610
Ephemera danica EDAN011225
Diptera
Lepidoptera
Trichoptera
Coleoptera
Strepsiptera
Hymenoptera
Psocodea
Thysanoptera
Hemiptera
Blattodea

Taz

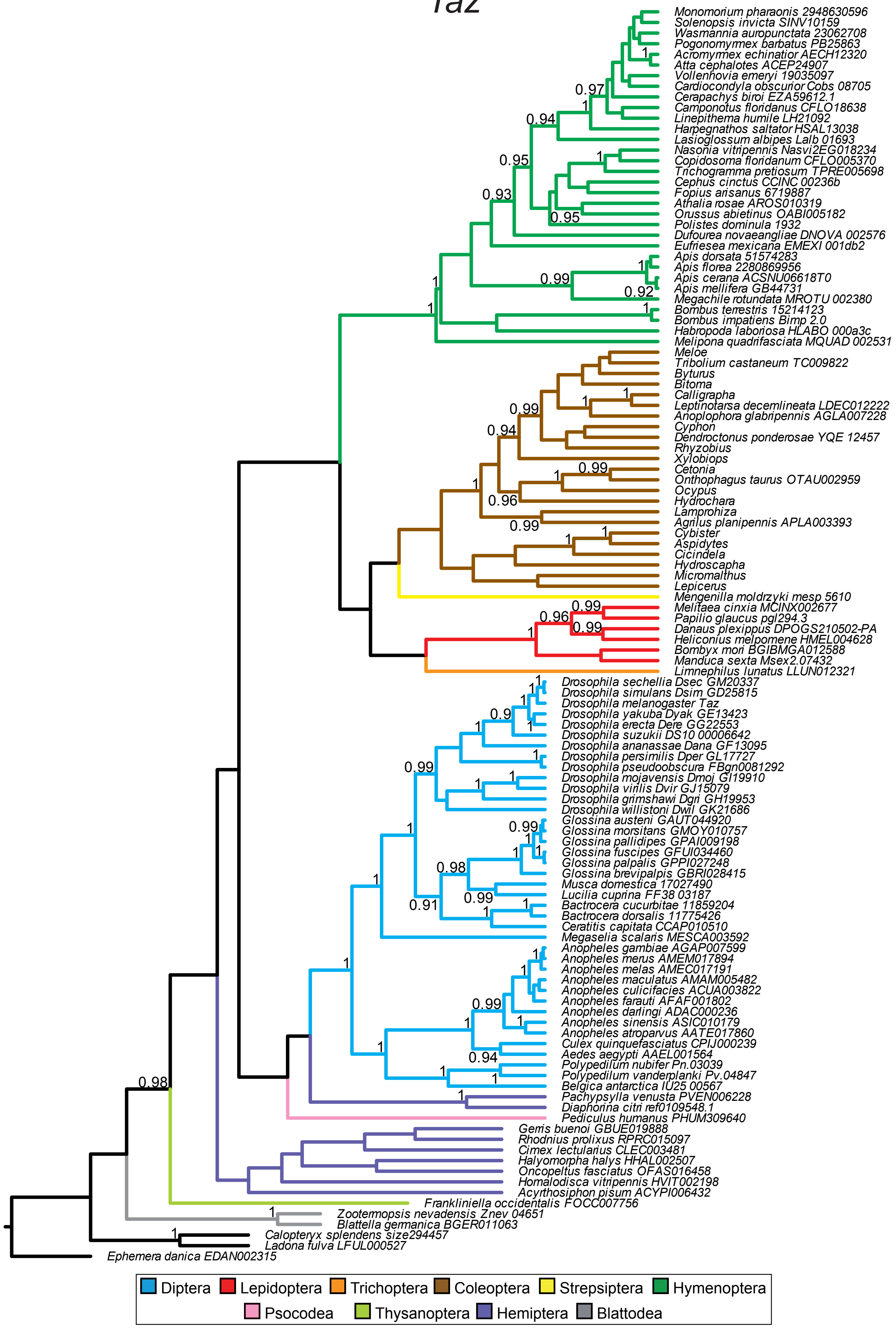
Monomorium pharaonis 2948630596
Solenopsis invicta SINV10159
Wasmannia auropunctata 23062708
Pogonomyrmex barbatus PB25863
Acromyrmex echinatior AECH12320
Atta cephalotes ACEP24907
Vollenhovia emeryi 19035097
Cardiocondyla obscurior Cobs 08705
Cerapachys biroi EZA59612.1
Camponotus floridanus CFLO18638
Linepithema humile LH21092
Harpegnathos saltator HSAL13038
Lasioglossum albipes Lalb 01693
Nasonia vitripennis Nasvi2EG018234
Copidosoma floridanum CFLO005370
Trichogramma pretiosum TPRE005698
Cephus cinctus CCINC 00236b
Fopius arisanus 6719887
Athalia rosae AROS010319
Orussus abietinus OABI005182
Polistes dominula 1932
Dufourea novaeangliae DNOVA 002576
Eufriesea mexicana EMEXI 001db2
Apis dorsata 51574283
Apis florea 2280869956
Apis cerana ACSNU06618T0
Apis mellifera GB44731
Megachile rotundata MROTU 002380
Bombus terrestris 15214123
Bombus impatiens Bimp 2.0
Habropoda laboriosa HLABO 000a3c
Melipona quadrifasciata MQUAD 002531
Meloe
Tribolium castaneum TC009822
Byturus
Bitoma
Calligrapha
Leptinotarsa decemlineata LDEC012222
Anoplophora glabripennis AGLA007228
Cyphon
Dendroctonus ponderosae YQE 12457
Rhyzobius
Xylobiops
Cetonia
Onthophagus taurus OTAU002959
Ocypus
Hydrochara
Lamprohiza
Agrilus planipennis APLA003393
Cybister
Aspidytes
Cicindela
Hydroscapha
Micromalthus
Lepicerus
Mengenilla moldrzyki mesp 5610
Melitaea cinxia MCINX002677
Papilio glaucus pgl294.3
Danaus plexippus DPOGS210502-PA
Heliconius melpomene HMEL004628
Bombyx mori BGIBMGA012588
Manduca sexta Msex2.07432
Limnephilus lunatus LLUN012321
Drosophila sechellia Dsec GM20337
Drosophila simulans Dsim GD25815
Drosophila melanogaster Taz
Drosophila yakuba Dyak GE13423
Drosophila erecta Dere GG22553
Drosophila suzukii DS10 00006642
Drosophila ananassae Dana GF13095
Drosophila persimilis Dper GL17727
Drosophila pseudoobscura FBgn0081292
Drosophila mojavensis Dmoj GI19910
Drosophila virilis Dvir GJ15079
Drosophila grimshawi Dgri GH19953
Drosophila willistoni Dwil GK21686
Glossina austeni GAUT044920
Glossina morsitans GMOY010757
Glossina pallidipes GPAI009198
Glossina fuscipes GFUI034460
Glossina palpalis GPPI027248
Glossina brevipalpis GBRI028415
Musca domestica 17027490
Lucilia cuprina FF38 03187
Bactrocera cucurbitae 11859204
Bactrocera dorsalis 11775426
Ceratitis capitata CCAP010510
Megaselia scalaris MESCA003592
Anopheles gambiae AGAP007599
Anopheles merus AMEM017894
Anopheles melas AMEC017191
Anopheles maculatus AMAM005482
Anopheles culicifacies ACUA003822
Anopheles farauti AFAF001802
Anopheles darlingi ADAC000236
Anopheles sinensis ASIC010179
Anopheles atroparvus AATE017860
Culex quinquefasciatus CPIJ000239
Aedes aegypti AAEL001564
Polypedilum nubifer Pn.03039
Polypedilum vanderplanki Pv.04847
Belgica antarctica IU25 00567
Pachypsylla venusta PVEN006228
Diaphorina citri ref0109548.1
Pediculus humanus PHUM309640
Gerris buenoi GBUE019888
Rhodnius prolixus RPRC015097
Cimex lectularius CLEC003481
Halyomorpha halys HHAL002507
Oncopeltus fasciatus OFAS016458
Homalodisca vitripennis HVIT002198
Acyrthosiphon pisum ACYPI006432
Frankliniella occidentalis FOCC007756
Zootermopsis nevadensis Znev 04651
Blattella germanica BGER011063
Calopteryx splendens size294457
Ladona fulva LFUL000527
Ephemera danica EDAN002315
Diptera
Lepidoptera
Trichoptera
Coleoptera
Strepsiptera
Hymenoptera
Psocodea
Thysanoptera
Hemiptera
Blattodea

Vps28

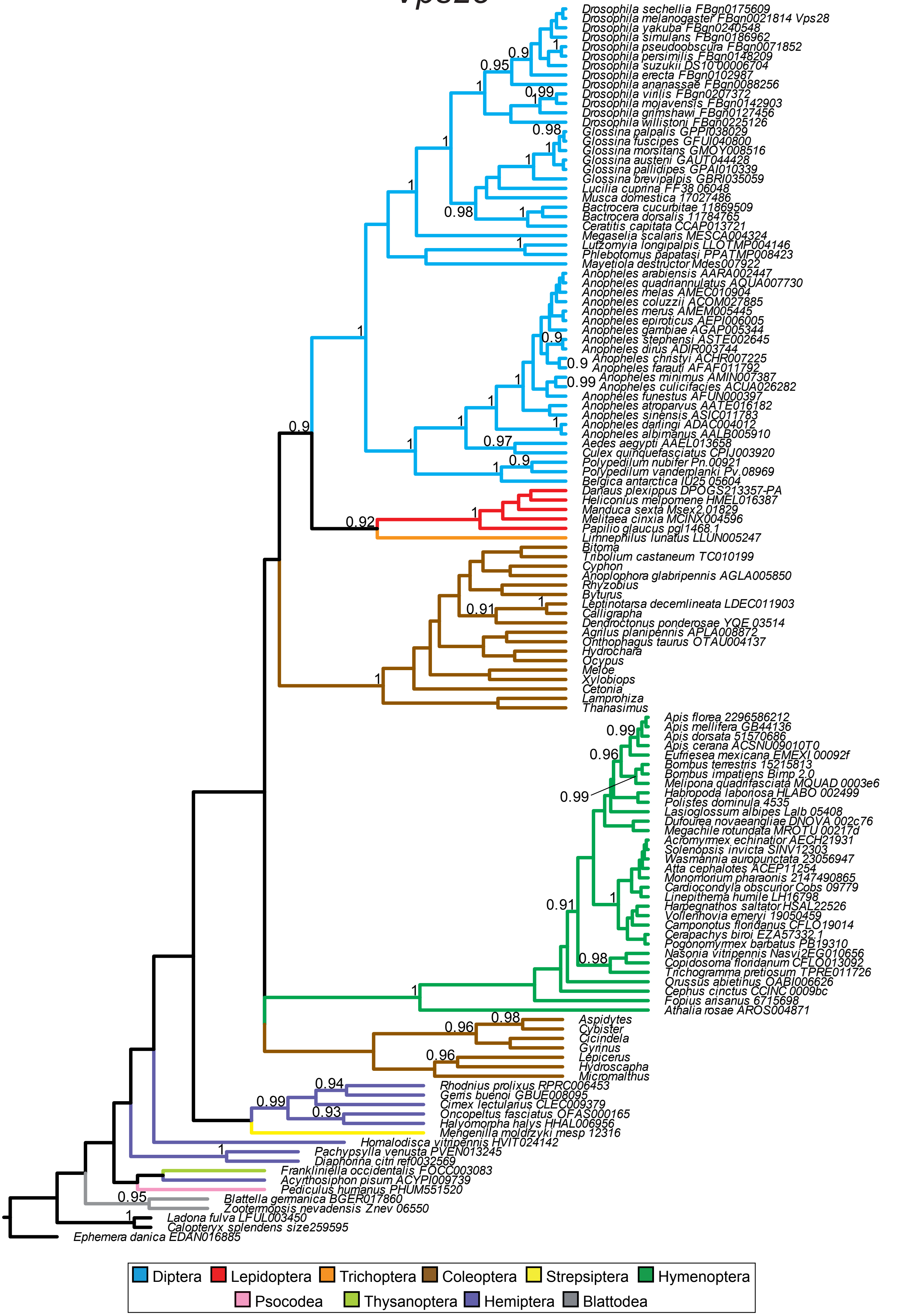
Drosophila sechellia FBgn0175609
Drosophila melanogaster FBgn0021814 Vps28
Drosophila yakuba FBgn0240548
Drosophila simulans FBgn0186962
Drosophila pseudoobscura FBgn0071852
Drosophila persimilis FBgn0148209
Drosophila suzukii DS10 00006704
Drosophila erecta FBgn0102987
Drosophila ananassae FBgn0088256
Drosophila virilis FBgn0207372
Drosophila mojavensis FBgn0142903
Drosophila grimshawi FBgn0127456
Drosophila willistoni FBgn0225126
Glossina palpalis GPPI038029
Glossina fuscipes GFUI040800
Glossina morsitans GMOY008516
Glossina austeni GAUT044428
Glossina pallidipes GPAI010339
Glossina brevipalpis GBRI035059
Lucilia cuprina FF38 06048
Musca domestica 17027486
Bactrocera cucurbitae 11869509
Bactrocera dorsalis 11784765
Ceratitis capitata CCAP013721
Megaselia scalaris MESCA004324
Lutzomyia longipalpis LLOTMP004146
Phlebotomus papatasi PPATMP008423
Mayetiola destructor Mdes007922
Anopheles arabiensis AARA002447
Anopheles quadriannulatus AQUA007730
Anopheles melas AMEC010904
Anopheles coluzzii ACOM027885
Anopheles merus AMEM005445
Anopheles epiroticus AEPI006005
Anopheles gambiae AGAP005344
Anopheles stephensi ASTE002645
Anopheles dirus ADIR003744
Anopheles christyi ACHR007225
Anopheles farauti AFAF011792
Anopheles minimus AMIN007387
Anopheles culicifacies ACUA026282
Anopheles funestus AFUN000397
Anopheles atroparvus AATE016182
Anopheles sinensis ASIC011783
Anopheles darlingi ADAC004012
Anopheles albimanus AALB005910
Aedes aegypti AAEL013658
Culex quinquefasciatus CPIJ003920
Polypedilum nubifer Pn.00921
Polypedilum vanderplanki Pv.08969
Belgica antarctica IU25 05604
Danaus plexippus DPOGS213357-PA
Heliconius melpomene HMEL016387
Manduca sexta Msex2.01829
Melitaea cinxia MCINX004596
Papilio glaucus pgl1468.1
Limnephilus lunatus LLUN005247
Bitoma
Tribolium castaneum TC010199
Cyphon
Anoplophora glabripennis AGLA005850
Rhyzobius
Byturus
Leptinotarsa decemlineata LDEC011903
Calligrapha
Dendroctonus ponderosae YQE 03514
Agrilus planipennis APLA008872
Onthophagus taurus OTAU004137
Hydrochara
Ocypus
Meloe
Xylobiops
Cetonia
Lamprohiza
Thanasimus
Apis florea 2296586212
Apis mellifera GB44136
Apis dorsata 51570686
Apis cerana ACSNU09010T0
Eufriesea mexicana EMEXI 00092f
Bombus terrestris 15215813
Bombus impatiens Bimp 2.0
Melipona quadrifasciata MQUAD 0003e6
Habropoda laboriosa HLABO 002499
Polistes dominula 4535
Lasioglossum albipes Lalb 05408
Dufourea novaeangliae DNOVA 002c76
Megachile rotundata MROTU 00217d
Acromyrmex echinatior AECH21931
Solenopsis invicta SINV12303
Wasmannia auropunctata 23056947
Atta cephalotes ACEP11254
Monomorium pharaonis 2147490865
Cardiocondyla obscurior Cobs 09779
Linepithema humile LH16798
Harpegnathos saltator HSAL22526
Vollenhovia emeryi 19050459
Camponotus floridanus CFLO19014
Cerapachys biroi EZA57332.1
Pogonomyrmex barbatus PB19310
Nasonia vitripennis Nasvi2EG010656
Copidosoma floridanum CFLO013092
Trichogramma pretiosum TPRE011726
Orussus abietinus OABI006626
Cephus cinctus CCINC 0009bc
Fopius arisanus 6715698
Athalia rosae AROS004871
Aspidytes
Cybister
Cicindela
Gyrinus
Lepicerus
Hydroscapha
Micromalthus
Rhodnius prolixus RPRC006453
Gerris buenoi GBUE008095
Cimex lectularius CLEC009379
Oncopeltus fasciatus OFAS000165
Halyomorpha halys HHAL006956
Mengenilla moldrzyki mesp 12316
Homalodisca vitripennis HVIT024142
Pachypsylla venusta PVEN013245
Diaphorina citri ref0032569
Frankliniella occidentalis FOCC003083
Acyrthosiphon pisum ACYPI009739
Pediculus humanus PHUM551520
Blattella germanica BGER017860
Zootermopsis nevadensis Znev 06550
Ladona fulva LFUL003450
Calopteryx splendens size259595
Ephemera danica EDAN016885
Diptera
Lepidoptera
Trichoptera
Coleoptera
Strepsiptera
Hymenoptera
Psocodea
Thysanoptera
Hemiptera
Blattodea

File S3. Maximum likelihood trees based on the nucleotide alignments of different sperm individualization genes in beetles.

Tree	Gene	Model	Gamma	Invariant	Log likelihood
**1**	*Ance*	GTR	0.660	0.146	-24961.88327
**2**	*aux*	GTR	0.386	0.070	-27681.59804
**3**	*blanks*	GTR	0.931	0.100	-12607.08759
**4**	*Bug22*	GTR	0.222	NA	-3454.61489
**5**	*CdsA*	GTR	0.290	0.154	-17968.57896
**6**	*Chc*	GTR	0.368	0.379	-55858.11062
**7**	*Ctp*	GTR	0.096	NA	-1384.6439
**8**	*Cul3*	TN93	0.175	0.204	-28997.57548
**9**	*Dark*	GTR	1.265	0.092	-20388.14695
**10**	*didum*	GTR	0.759	0.209	-60072.64694
**11**	*Dredd*	GTR	1.197	0.096	-14618.72638
**12**	*Dronc*	GTR	1.355	0.099	-15698.08633
**13**	*Duba*	GTR	0.527	0.176	-12010.48828
**14**	*EcR*	GTR	0.449	0.365	-12199.45136
**15**	*eIF3m*	GTR	0.329	0.137	-18855.00527
**16**	*Fadd*	GTR	1.321	0.084	-4356.88960
**17**	*gish*	GTR	0.211	0.170	-15923.19717
**18**	*gudu*	GTR	0.618	0.135	-22338.05316
**19**	*heph*	TN93	0.396	0.275	-8479.41719
**20**	*hmw*	GTR	0.898	NA	-1707.47296
**21**	*jar*	GTR	0.708	0.241	-52172.98272
**22**	*klhl10*	GTR	0.504	0.239	-17745.77407
**23**	*Lasp*	GTR	0.474	0.351	-5407.13219
**24**	*Mer*	GTR	0.598	0.356	-22473.10341
**25**	*mlt*	GTR	0.593	0.196	-19352.46023
**26**	*nes*	GTR	0.911	0.148	-25494.50312
**27**	*Npc1a*	GTR	0.724	0.204	-58520.58794
**28**	*nsr*	GTR	0.287	0.143	-11564.48392
**29**	*orb2*	GTR	0.327	0.450	-4091.42072
**30**	*Osbp*	GTR	0.699	0.244	-29993.57419
**31**	*oys*	GTR	0.773	0.158	-15795.25364
**32**	*Past1*	GTR	0.474	0.325	-14648.80870
**33**	*Pen*	GTR	0.676	0.232	-26388.05898
**34**	*poe*	GTR	0.590	0.253	-50399.32806
**35**	*porin*	GTR	0.674	0.191	-15348.95453
**36**	*Prosalpha6T*	GTR	0.659	0.312	-13745.91664
**37**	*scat*	GTR	0.556	0.147	-34071.26981
**38**	*shi*	GTR	0.552	0.395	-34985.87502
**39**	*skap*	GTR	0.596	0.317	-20864.74113
**40**	*sw*	GTR	0.473	0.265	-21539.32783
**41**	*Taz*	GTR	0.717	0.284	-11982.00743
**42**	*Vps28*	GTR	0.241	0.154	-7981.57649

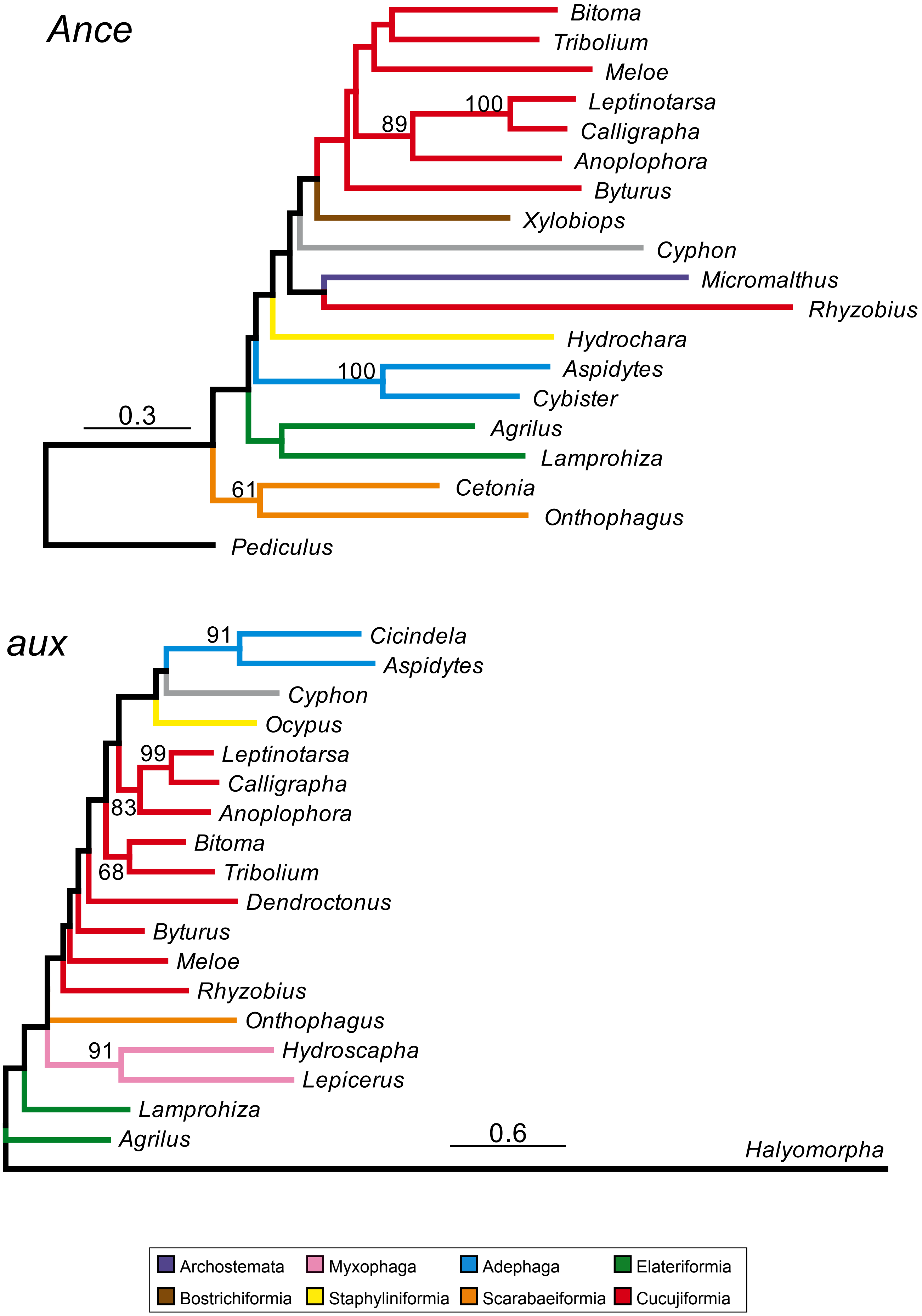

Ance
Bitoma
Tribolium
Meloe
100
Leptinotarsa
89
Calligrapha
Anoplophora
Byturus
Xylobiops
Cyphon
Micromalthus
Rhyzobius
Hydrochara
100
Aspidytes
Cybister
0.3
Agrilus
Lamprohiza
61
Cetonia
Onthophagus
Pediculus
aux
91
Cicindela
Aspidytes
Cyphon
Ocypus
99
Leptinotarsa
Calligrapha
83
Anoplophora
Bitoma
68
Tribolium
Dendroctonus
Byturus
Meloe
Rhyzobius
Onthophagus
91
Hydroscapha
Lepicerus
Lamprohiza
Agrilus
0.6
Halyomorpha
Archostemata
Myxophaga
Adephaga
Elateriformia
Bostrichiformia
Staphyliniformia
Scarabaeiformia
Cucujiformia

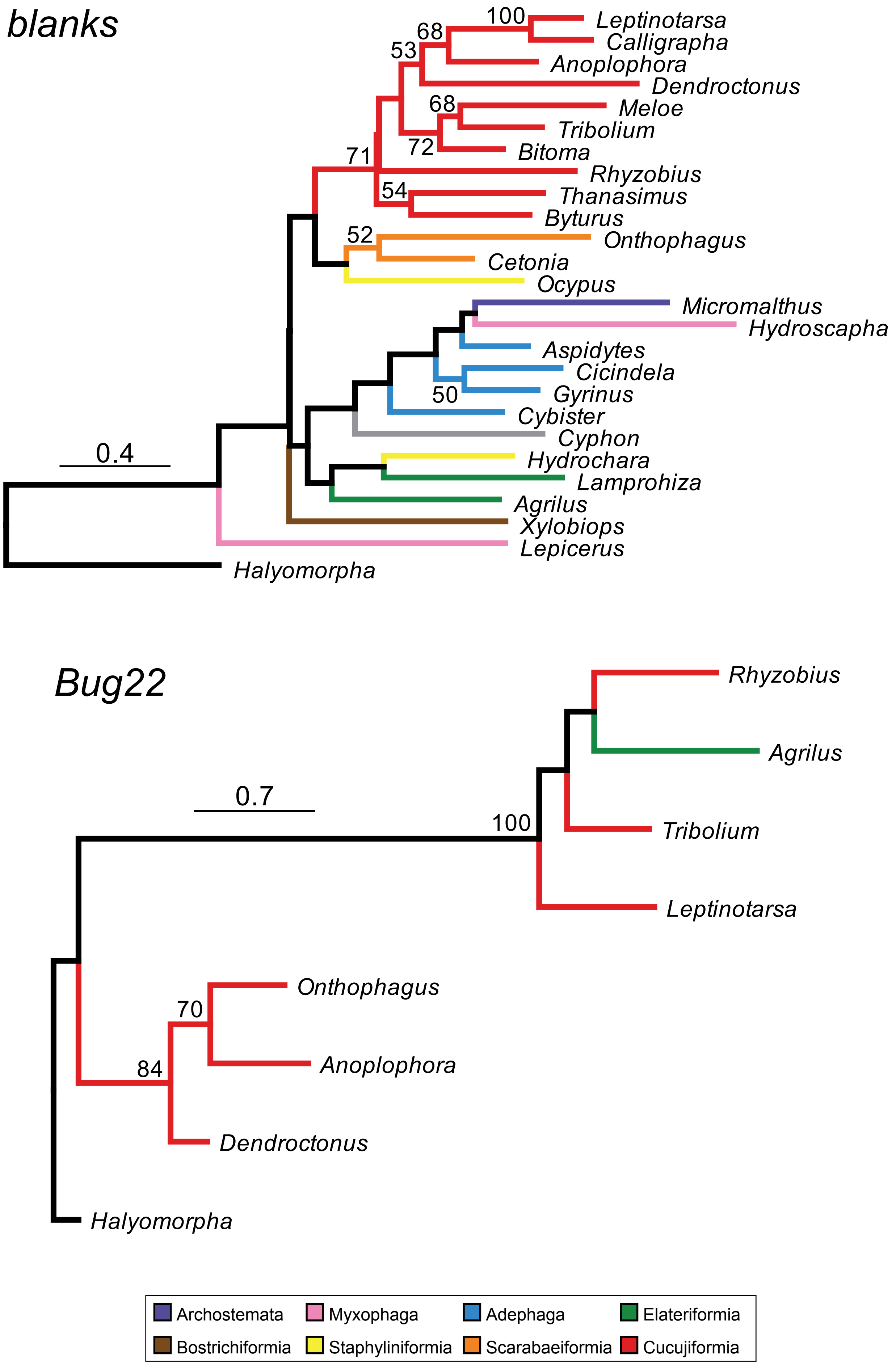
blanks
100
68
53
68
72
71
54
52
50
Leptinotarsa
Calligrapha
Anoplophora
Dendroctonus
Meloe
Tribolium
Bitoma
Rhyzobius
Thanasimus
Byturus
Onthophagus
Cetonia
Ocypus
Micromalthus
Hydroscapha
Aspidytes
Cicindela
Gyrinus
Cybister
Cyphon
Hydrochara
Lamprohiza
Agrilus
Xylobiops
Lepicerus
Halyomorpha
0.4
Bug22
0.7
100
Rhyzobius
Agrilus
Tribolium
Leptinotarsa
70
84
Onthophagus
Anoplophora
Dendroctonus
Halyomorpha
Archostemata
Myxophaga
Adephaga
Elateriformia
Bostrichiformia
Staphyliniformia
Scarabaeiformia
Cucujiformia

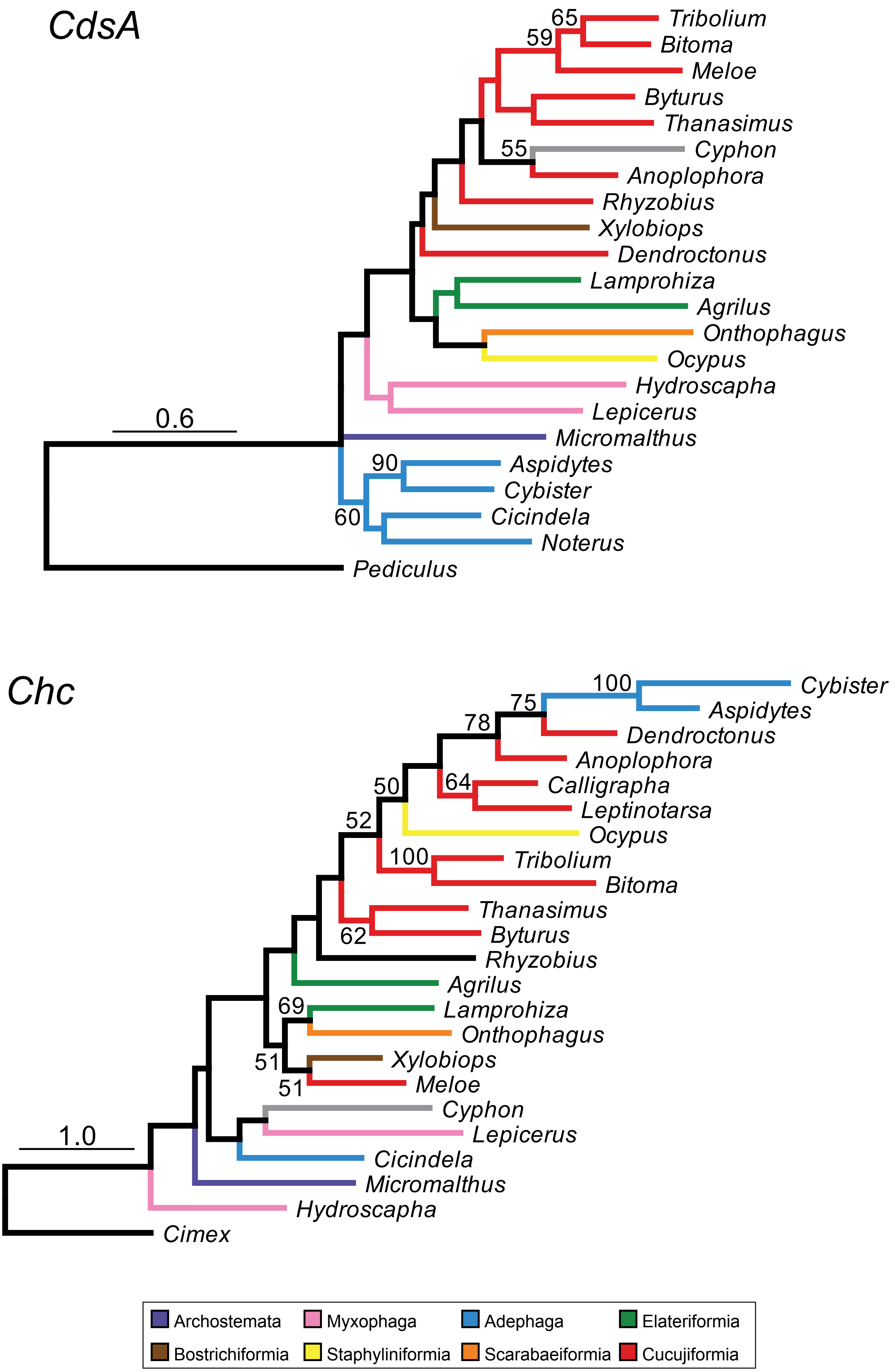

CdsA
0.6
65
59
55
90
60
Tribolium
Bitoma
Meloe
Byturus
Thanasimus
Cyphon
Anoplophora
Rhyzobius
Xylobiops
Dendroctonus
Lamprohiza
Agrilus
Onthophagus
Ocypus
Hydroscapha
Lepicerus
Micromalthus
Aspidytes
Cybister
Cicindela
Noterus
Pediculus
Chc
1.0
100
75
78
64
50
52
100
62
69
51
51
Cybister
Aspidytes
Dendroctonus
Anoplophora
Calligrapha
Leptinotarsa
Ocypus
Tribolium
Bitoma
Thanasimus
Byturus
Rhyzobius
Agrilus
Lamprohiza
Onthophagus
Xylobiops
Meloe
Cyphon
Lepicerus
Cicindela
Micromalthus
Hydroscapha
Cimex
Archostemata
Myxophaga
Adephaga
Elateriformia
Bostrichiformia
Staphyliniformia
Scarabaeiformia
Cucujiformia

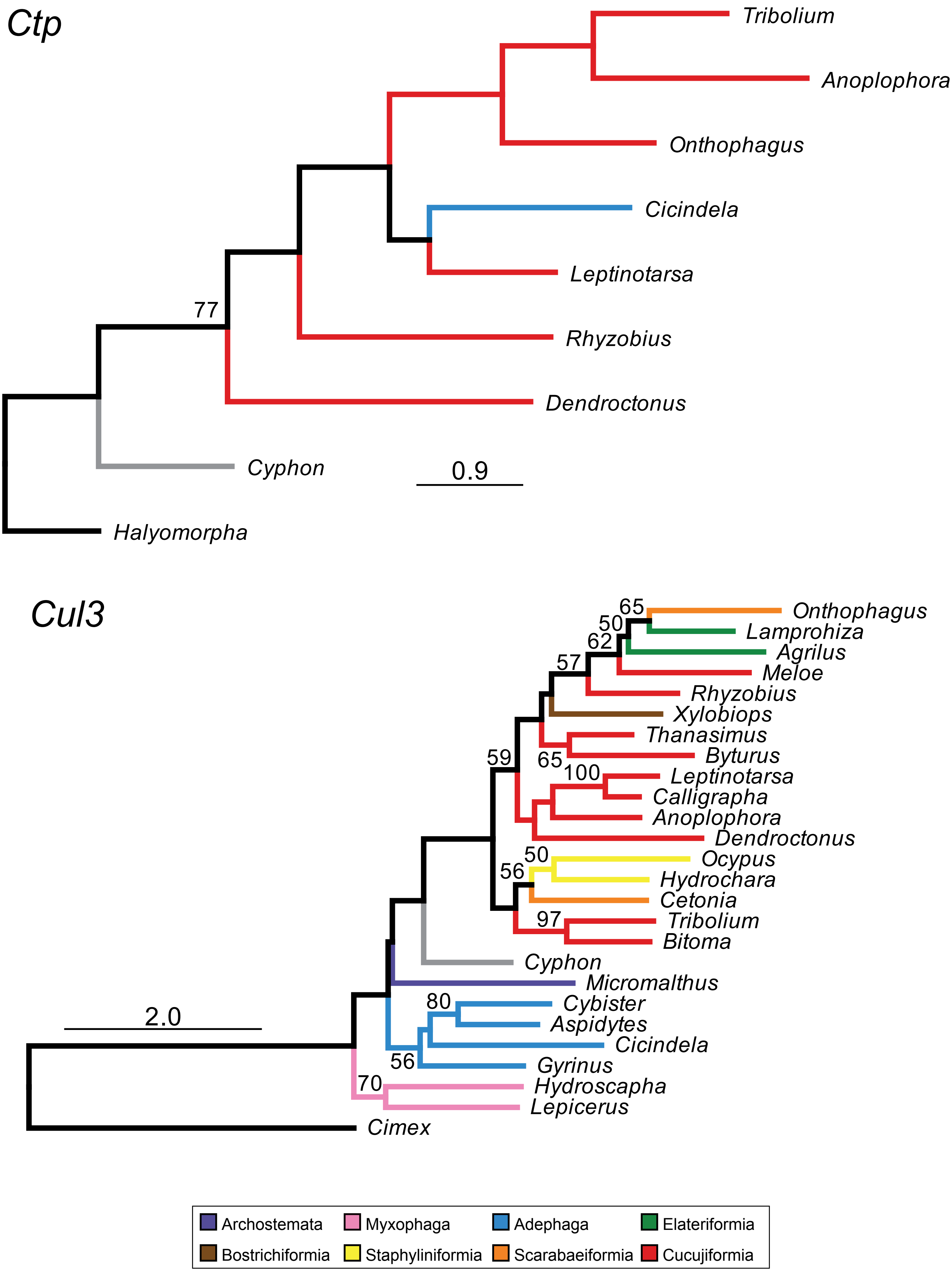
Ctp
Tribolium
Anoplophora
Onthophagus
Cicindela
Leptinotarsa
Rhyzobius
Dendroctonus
Cyphon
Halyomorpha
77
0.9
Cul3
Onthophagus
Lamprohiza
Agrilus
Meloe
Rhyzobius
Xylobiops
Thanasimus
Byturus
Leptinotarsa
Calligrapha
Anoplophora
Dendroctonus
Ocypus
Hydrochara
Cetonia
Tribolium
Bitoma
Cyphon
Micromalthus
Cybister
Aspidytes
Cicindela
Gyrinus
Hydroscapha
Lepicerus
Cimex
65
50
62
57
59
65
100
50
56
97
80
56
70
2.0
Archostemata
Myxophaga
Adephaga
Elateriformia
Bostrichiformia
Staphyliniformia
Scarabaeiformia
Cucujiformia

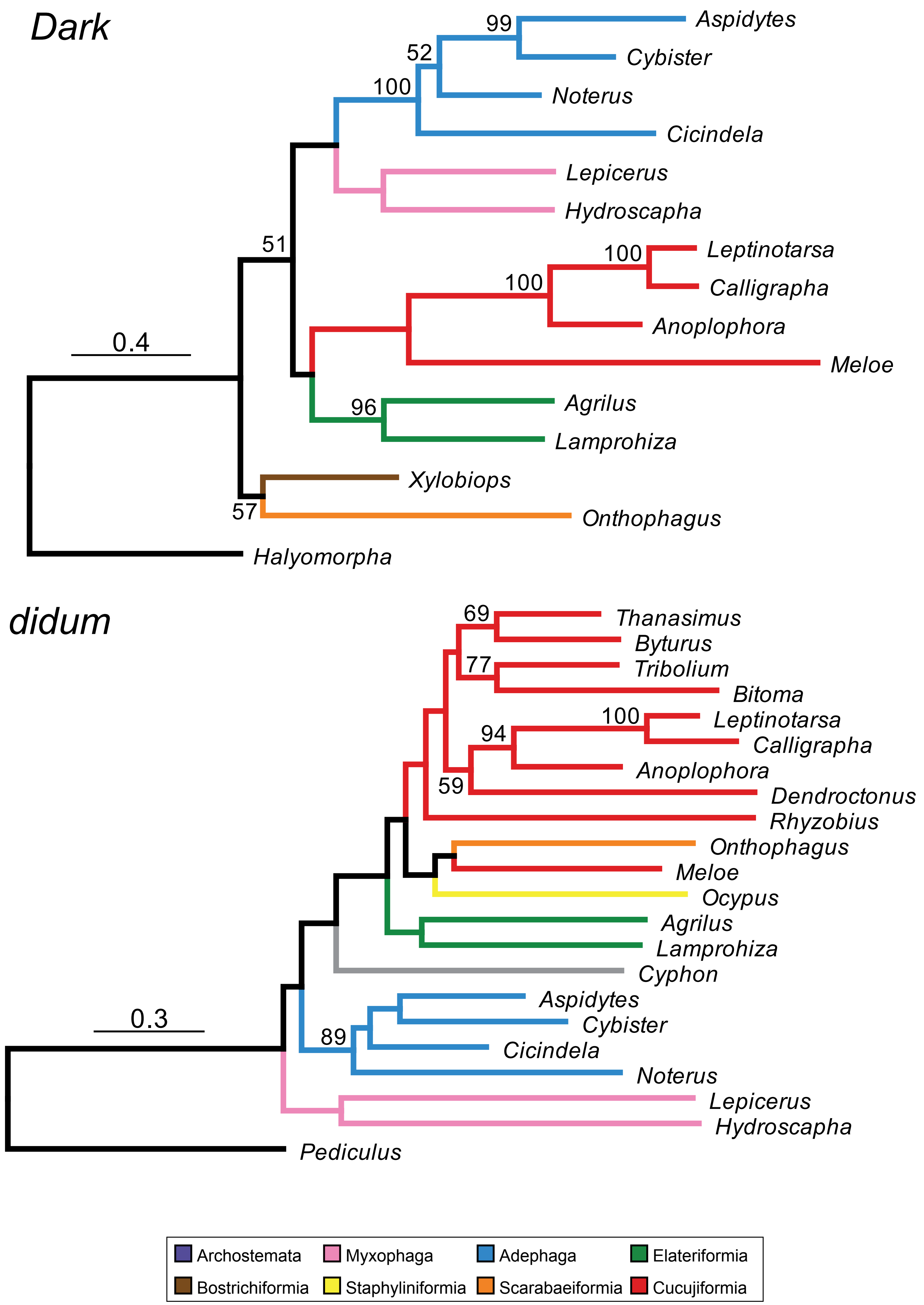
Dark
99
52
100
Aspidytes
Cybister
Noterus
Cicindela
Lepicerus
Hydroscapha
51
100
100
Leptinotarsa
Calligrapha
Anoplophora
Meloe
0.4
96
Agrilus
Lamprohiza
Xylobiops
57
Onthophagus
Halyomorpha
didum
69
Thanasimus
Byturus
77
Tribolium
Bitoma
100
94
Leptinotarsa
Calligrapha
Anoplophora
59
Dendroctonus
Rhyzobius
Onthophagus
Meloe
Ocypus
Agrilus
Lamprohiza
Cyphon
Aspidytes
0.3
89
Cybister
Cicindela
Noterus
Lepicerus
Hydroscapha
Pediculus
Archostemata
Myxophaga
Adephaga
Elateriformia
Bostrichiformia
Staphyliniformia
Scarabaeiformia
Cucujiformia

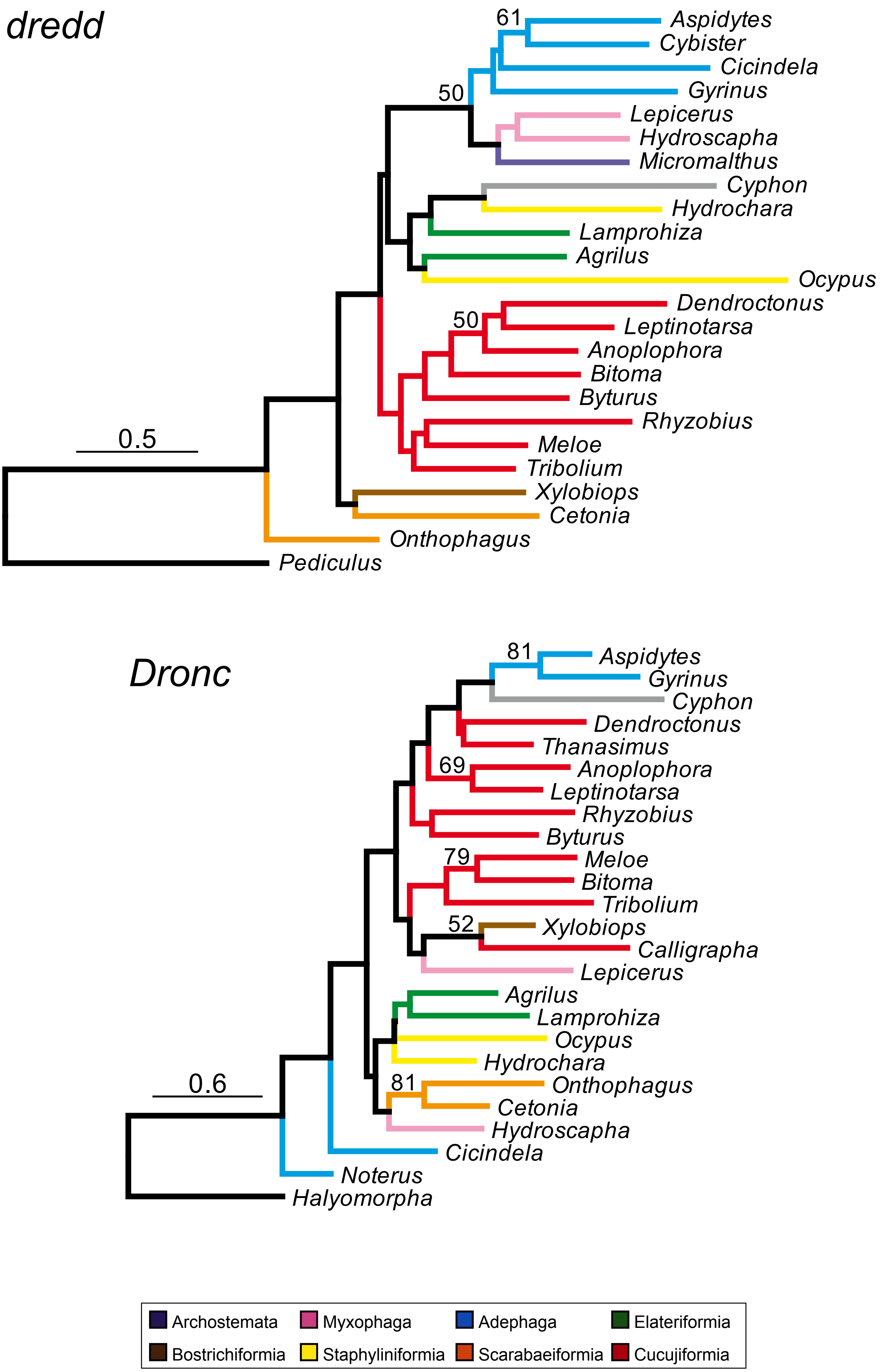

dredd
61
50
Aspidytes
Cybister
Cicindela
Gyrinus
Lepicerus
Hydroscapha
Micromalthus
Cyphon
Hydrochara
Lamprohiza
Agrilus
Ocypus
50
Dendroctonus
Leptinotarsa
Anoplophora
Bitoma
Byturus
Rhyzobius
Meloe
Tribolium
Xylobiops
Cetonia
Onthophagus
Pediculus
0.5
Dronc
81
Aspidytes
Gyrinus
Cyphon
Dendroctonus
Thanasimus
69
Anoplophora
Leptinotarsa
Rhyzobius
Byturus
79
Meloe
Bitoma
Tribolium
52
Xylobiops
Calligrapha
Lepicerus
Agrilus
Lamprohiza
Ocypus
Hydrochara
81
Onthophagus
Cetonia
Hydroscapha
Cicindela
Noterus
Halyomorpha
0.6
Archostemata
Myxophaga
Adephaga
Elateriformia
Bostrichiformia
Staphyliniformia
Scarabaeiformia
Cucujiformia

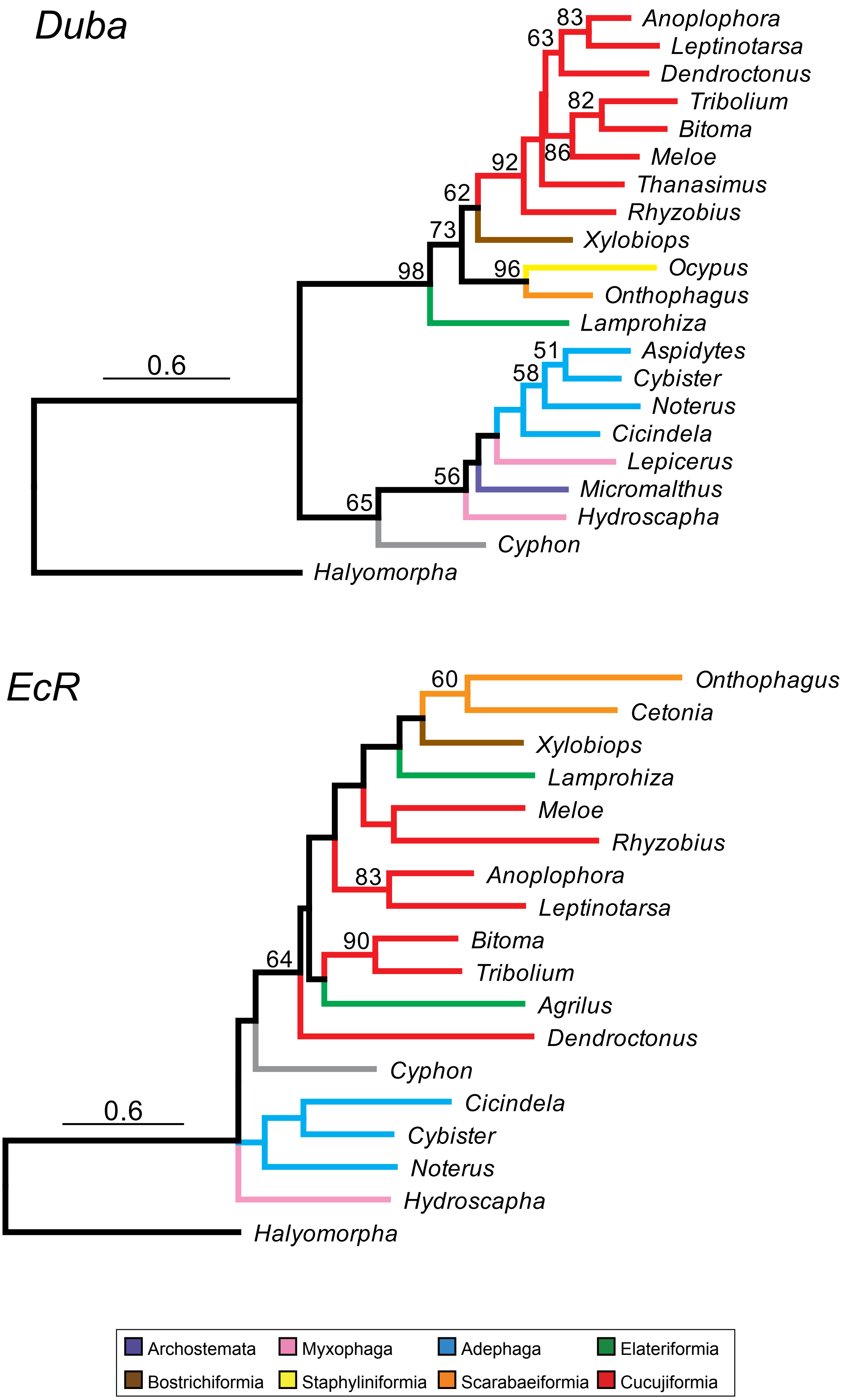
Duba
83
63
82
92
86
62
73
96
98
51
58
56
65
0.6
Anoplophora
Leptinotarsa
Dendroctonus
Tribolium
Bitoma
Meloe
Thanasimus
Rhyzobius
Xylobiops
Ocypus
Onthophagus
Lamprohiza
Aspidytes
Cybister
Noterus
Cicindela
Lepicerus
Micromalthus
Hydroscapha
Cyphon
Halyomorpha
EcR
60
83
90
64
0.6
Onthophagus
Cetonia
Xylobiops
Lamprohiza
Meloe
Rhyzobius
Anoplophora
Leptinotarsa
Bitoma
Tribolium
Agrilus
Dendroctonus
Cyphon
Cicindela
Cybister
Noterus
Hydroscapha
Halyomorpha
Archostemata
Myxophaga
Adephaga
Elateriformia
Bostrichiformia
Staphyliniformia
Scarabaeiformia
Cucujiformia

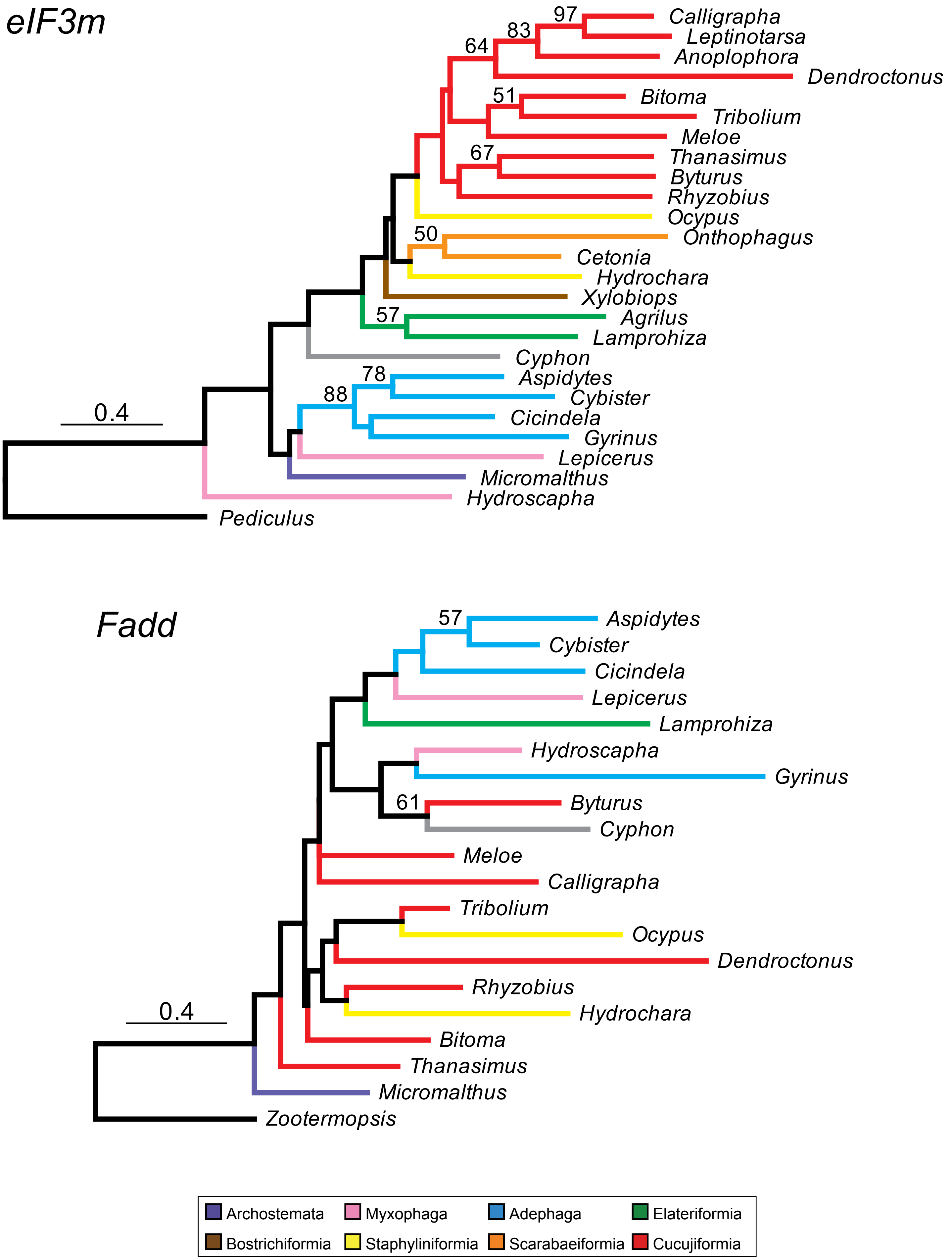

eIF3m
97
83
64
Calligrapha
Leptinotarsa
Anoplophora
Dendroctonus
51
Bitoma
Tribolium
Meloe
67
Thanasimus
Byturus
Rhyzobius
Ocypus
50
Onthophagus
Cetonia
Hydrochara
Xylobiops
57
Agrilus
Lamprohiza
Cyphon
78
88
Aspidytes
Cybister
Cicindela
Gyrinus
Lepicerus
Micromalthus
Hydroscapha
Pediculus
0.4
Fadd
57
Aspidytes
Cybister
Cicindela
Lepicerus
Lamprohiza
Hydroscapha
Gyrinus
61
Byturus
Cyphon
Meloe
Calligrapha
Tribolium
Ocypus
Dendroctonus
Rhyzobius
Hydrochara
Bitoma
Thanasimus
Micromalthus
Zootermopsis
0.4
Archostemata
Myxophaga
Adephaga
Elateriformia
Bostrichiformia
Staphyliniformia
Scarabaeiformia
Cucujiformia

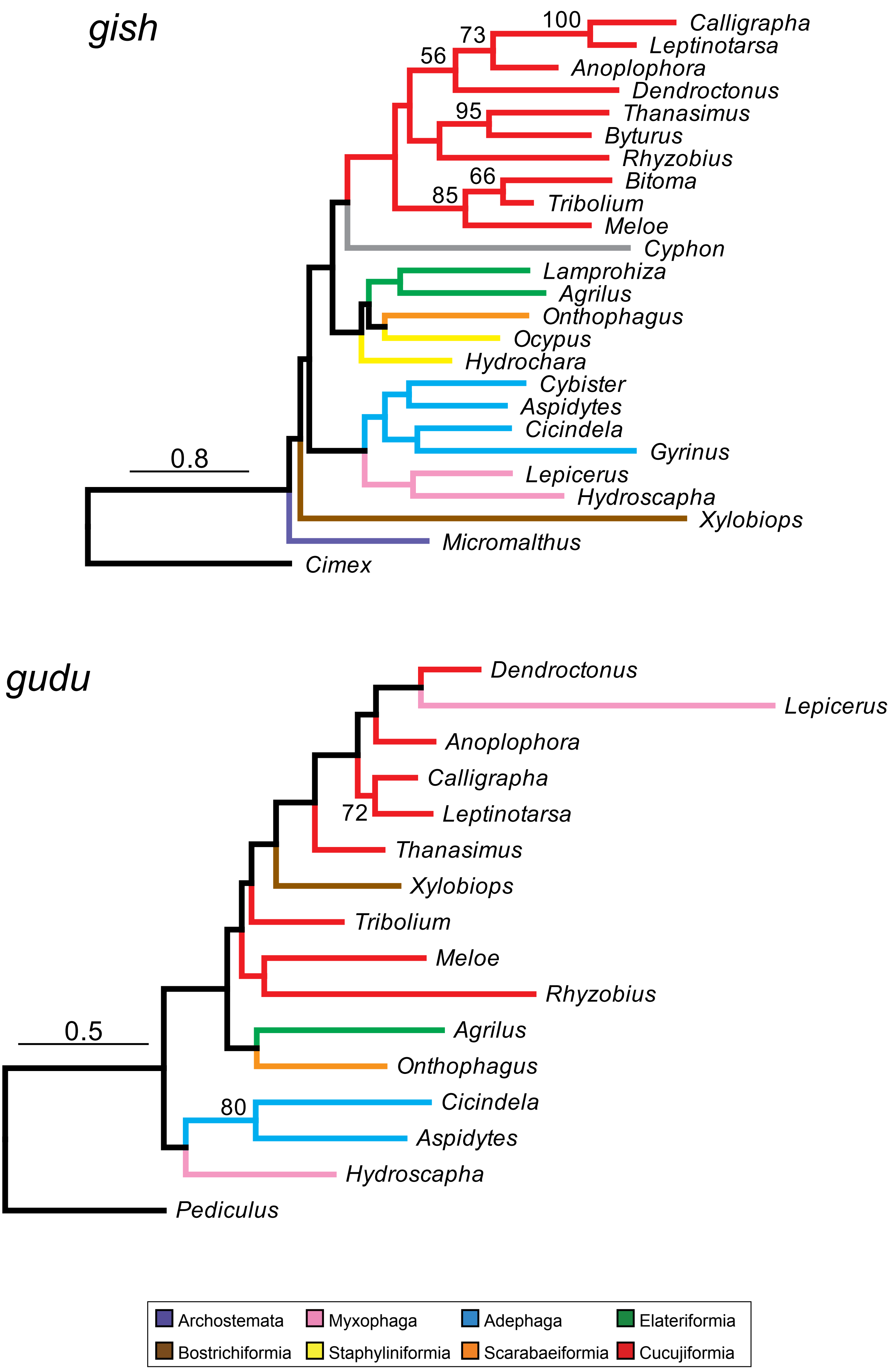
gish
100
73
56
95
66
85
Calligrapha
Leptinotarsa
Anoplophora
Dendroctonus
Thanasimus
Byturus
Rhyzobius
Bitoma
Tribolium
Meloe
Cyphon
Lamprohiza
Agrilus
Onthophagus
Ocypus
Hydrochara
Cybister
Aspidytes
Cicindela
Gyrinus
Lepicerus
Hydroscapha
Xylobiops
Micromalthus
Cimex
0.8
gudu
Dendroctonus
Lepicerus
Anoplophora
Calligrapha
72
Leptinotarsa
Thanasimus
Xylobiops
Tribolium
Meloe
Rhyzobius
Agrilus
Onthophagus
80
Cicindela
Aspidytes
Hydroscapha
Pediculus
0.5
Archostemata
Myxophaga
Adephaga
Elateriformia
Bostrichiformia
Staphyliniformia
Scarabaeiformia
Cucujiformia

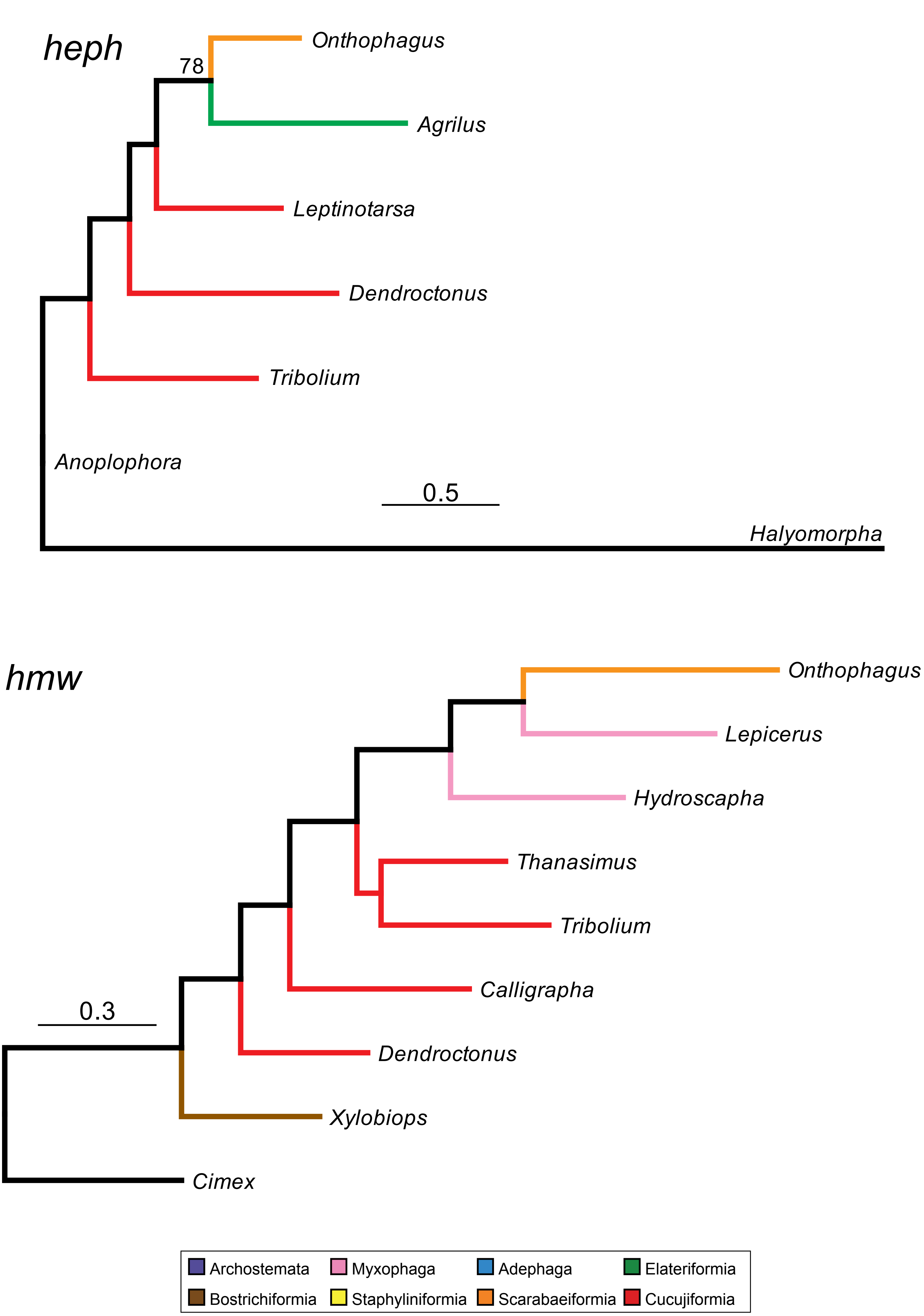
heph
78
Onthophagus
Agrilus
Leptinotarsa
Dendroctonus
Tribolium
Anoplophora
0.5
Halyomorpha
hmw
Onthophagus
Lepicerus
Hydroscapha
Thanasimus
Tribolium
Calligrapha
Dendroctonus
0.3
Xylobiops
Cimex
Archostemata
Myxophaga
Adephaga
Elateriformia
Bostrichiformia
Staphyliniformia
Scarabaeiformia
Cucujiformia

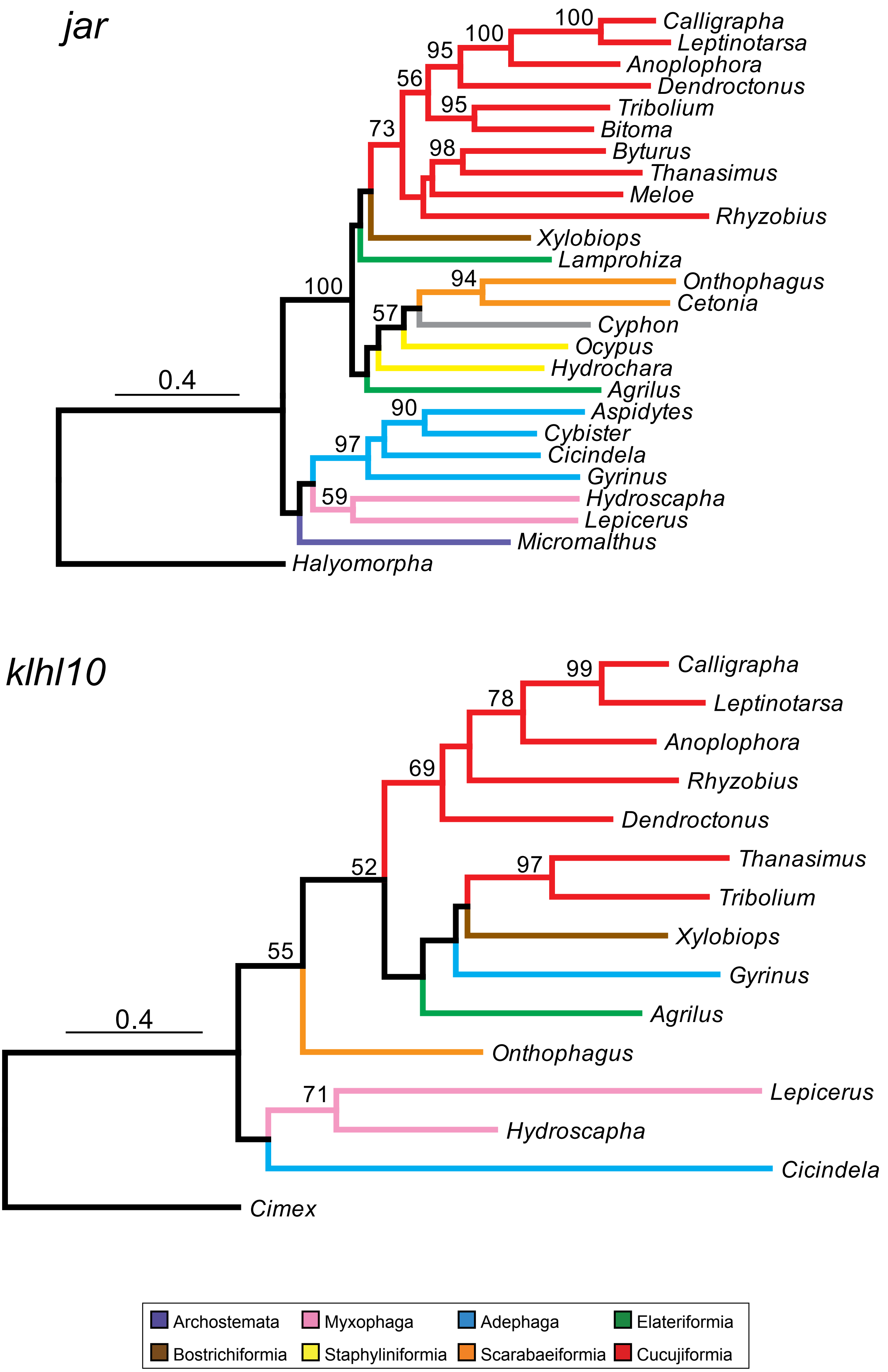

jar
100
Calligrapha
100
Leptinotarsa
95
Anoplophora
56
Dendroctonus
95
Tribolium
73
Bitoma
98
Byturus
Thanasimus
Meloe
Rhyzobius
Xylobiops
Lamprohiza
94
Onthophagus
100
Cetonia
57
Cyphon
0.4
Ocypus
Hydrochara
Agrilus
90
Aspidytes
97
Cybister
Cicindela
Gyrinus
59
Hydroscapha
Lepicerus
Micromalthus
Halyomorpha
klhl10
99
Calligrapha
78
Leptinotarsa
Anoplophora
69
Rhyzobius
Dendroctonus
97
Thanasimus
52
Tribolium
Xylobiops
55
Gyrinus
Agrilus
0.4
Onthophagus
71
Lepicerus
Hydroscapha
Cicindela
Cimex
Archostemata
Myxophaga
Adephaga
Elateriformia
Bostrichiformia
Staphyliniformia
Scarabaeiformia
Cucujiformia

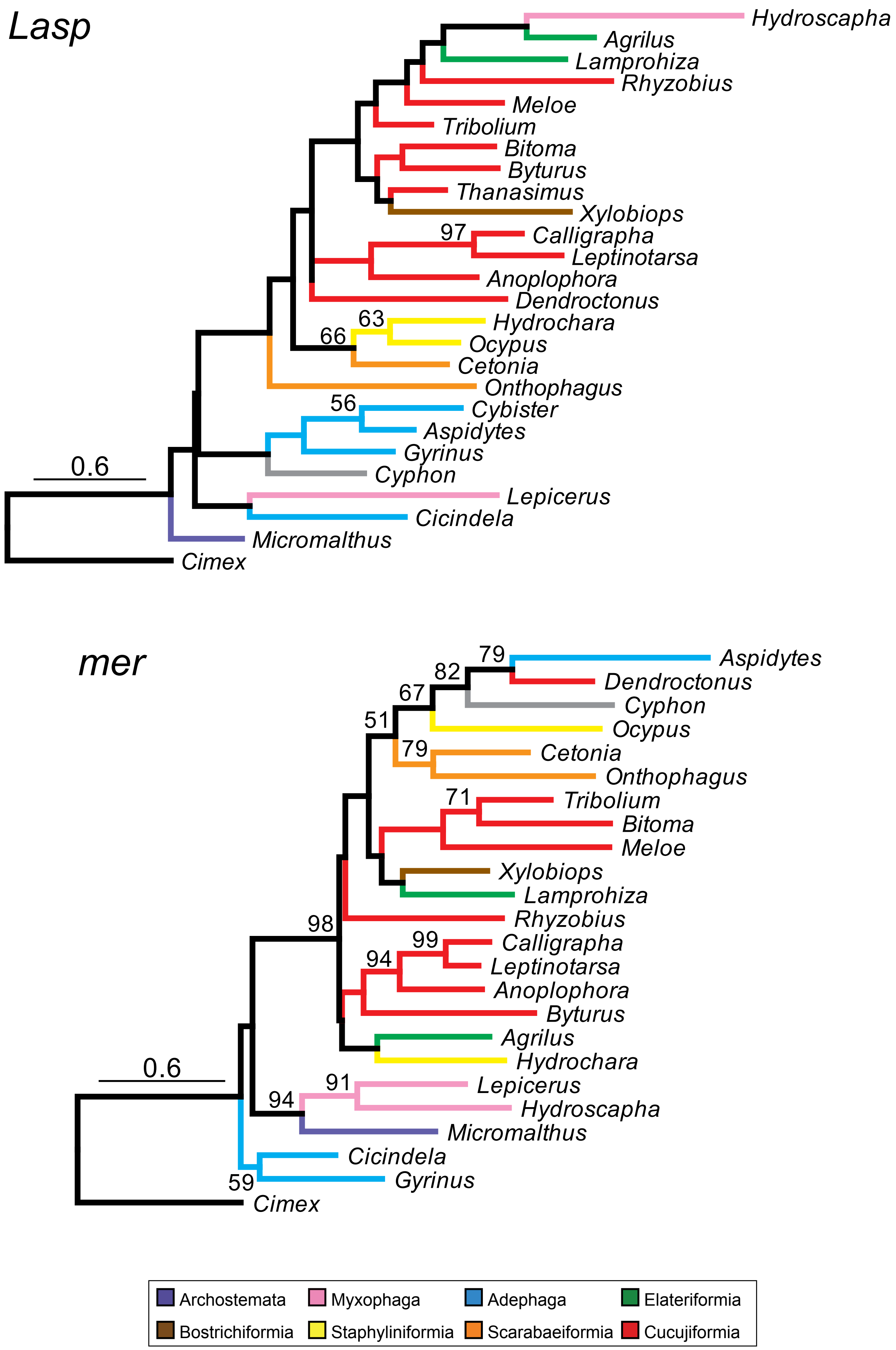

Lasp
Hydroscapha
Agrilus
Lamprohiza
Rhyzobius
Meloe
Tribolium
Bitoma
Byturus
Thanasimus
Xylobiops
97
Calligrapha
Leptinotarsa
Anoplophora
Dendroctonus
63
66
Hydrochara
Ocypus
Cetonia
Onthophagus
56
Cybister
Aspidytes
Gyrinus
Cyphon
0.6
Lepicerus
Cicindela
Micromalthus
Cimex
mer
79
82
67
51
Aspidytes
Dendroctonus
Cyphon
Ocypus
79
Cetonia
Onthophagus
71
Tribolium
Bitoma
Meloe
Xylobiops
Lamprohiza
98
Rhyzobius
99
94
Calligrapha
Leptinotarsa
Anoplophora
Byturus
Agrilus
Hydrochara
0.6
91
94
Lepicerus
Hydroscapha
Micromalthus
Cicindela
59
Gyrinus
Cimex
Archostemata
Myxophaga
Adephaga
Elateriformia
Bostrichiformia
Staphyliniformia
Scarabaeiformia
Cucujiformia

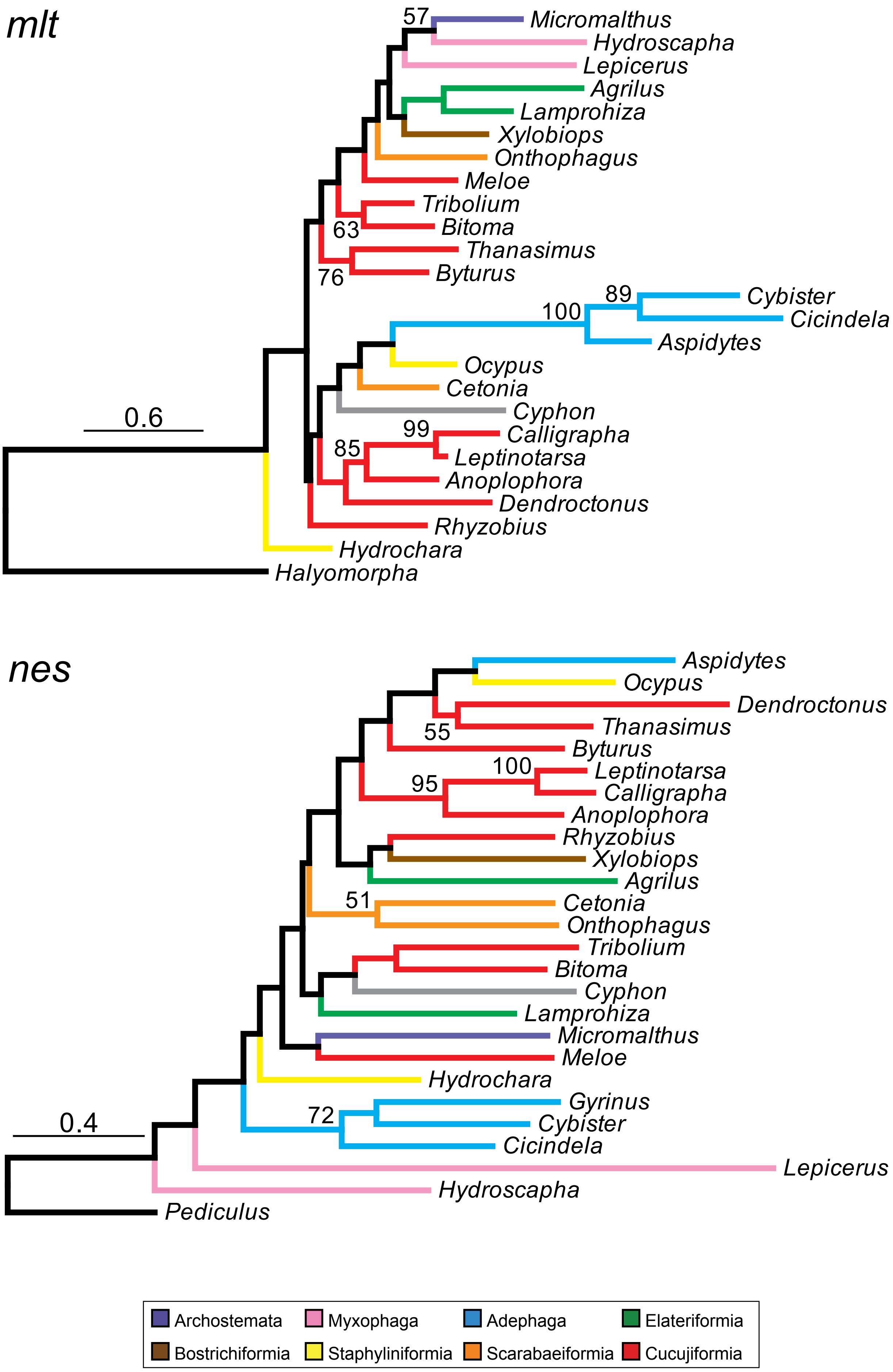
mlt
57
Micromalthus
Hydroscapha
Lepicerus
Agrilus
Lamprohiza
Xylobiops
Onthophagus
Meloe
Tribolium
63
Bitoma
Thanasimus
76
Byturus
89
Cybister
100
Cicindela
Aspidytes
Ocypus
Cetonia
Cyphon
0.6
99
Calligrapha
85
Leptinotarsa
Anoplophora
Dendroctonus
Rhyzobius
Hydrochara
Halyomorpha
nes
Aspidytes
Ocypus
Dendroctonus
Thanasimus
55
Byturus
100
Leptinotarsa
95
Calligrapha
Anoplophora
Rhyzobius
Xylobiops
Agrilus
51
Cetonia
Onthophagus
Tribolium
Bitoma
Cyphon
Lamprohiza
Micromalthus
Meloe
Hydrochara
Gyrinus
72
Cybister
0.4
Cicindela
Lepicerus
Hydroscapha
Pediculus
Archostemata
Myxophaga
Adephaga
Elateriformia
Bostrichiformia
Staphyliniformia
Scarabaeiformia
Cucujiformia

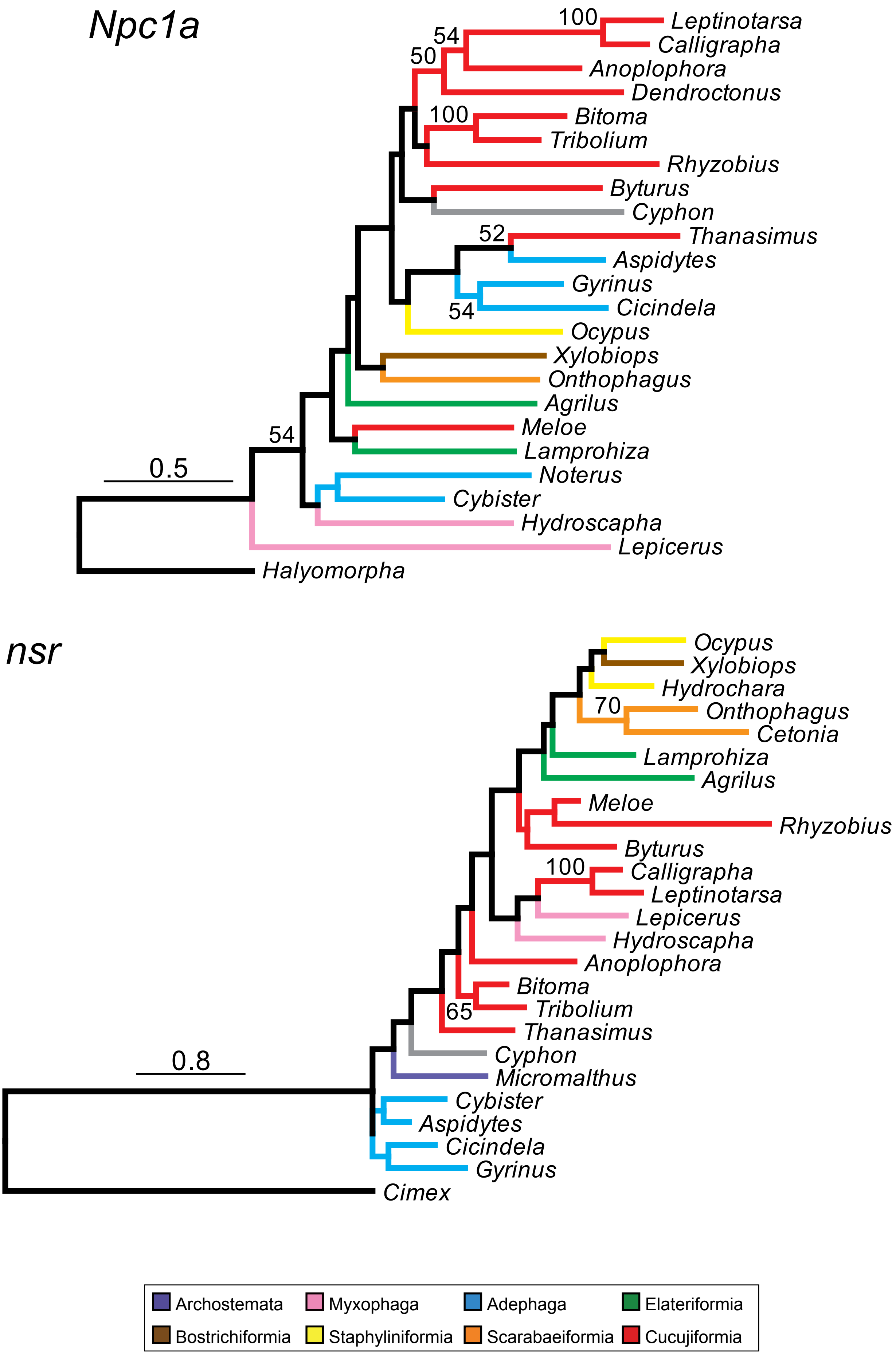

Npc1a
100
Leptinotarsa
Calligrapha
54
50
Anoplophora
Dendroctonus
100
Bitoma
Tribolium
Rhyzobius
Byturus
Cyphon
52
Thanasimus
Aspidytes
Gyrinus
54
Cicindela
Ocypus
Xylobiops
Onthophagus
Agrilus
Meloe
Lamprohiza
54
0.5
Noterus
Cybister
Hydroscapha
Lepicerus
Halyomorpha
nsr
Ocypus
Xylobiops
Hydrochara
70
Onthophagus
Cetonia
Lamprohiza
Agrilus
Meloe
Rhyzobius
Byturus
100
Calligrapha
Leptinotarsa
Lepicerus
Hydroscapha
Anoplophora
Bitoma
65
Tribolium
Thanasimus
Cyphon
Micromalthus
0.8
Cybister
Aspidytes
Cicindela
Gyrinus
Cimex
Archostemata
Myxophaga
Adephaga
Elateriformia
Bostrichiformia
Staphyliniformia
Scarabaeiformia
Cucujiformia

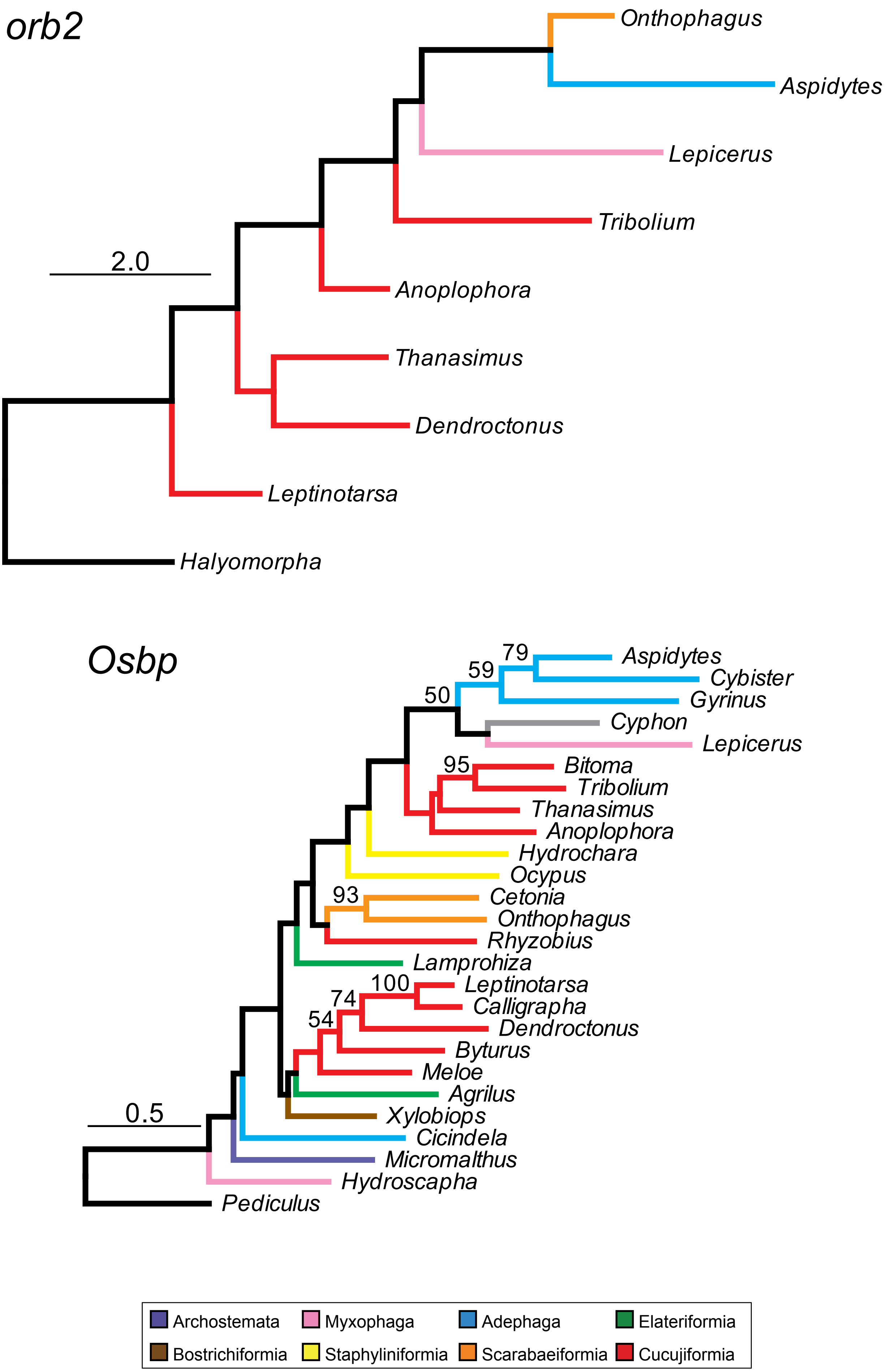
orb2
Onthophagus
Aspidytes
Lepicerus
Tribolium
2.0
Anoplophora
Thanasimus
Dendroctonus
Leptinotarsa
Halyomorpha
Osbp
79
59
50
Aspidytes
Cybister
Gyrinus
Cyphon
Lepicerus
95
Bitoma
Tribolium
Thanasimus
Anoplophora
Hydrochara
Ocypus
93
Cetonia
Onthophagus
Rhyzobius
Lamprohiza
100
74
54
Leptinotarsa
Calligrapha
Dendroctonus
Byturus
Meloe
Agrilus
Xylobiops
0.5
Cicindela
Micromalthus
Hydroscapha
Pediculus
Archostemata
Myxophaga
Adephaga
Elateriformia
Bostrichiformia
Staphyliniformia
Scarabaeiformia
Cucujiformia

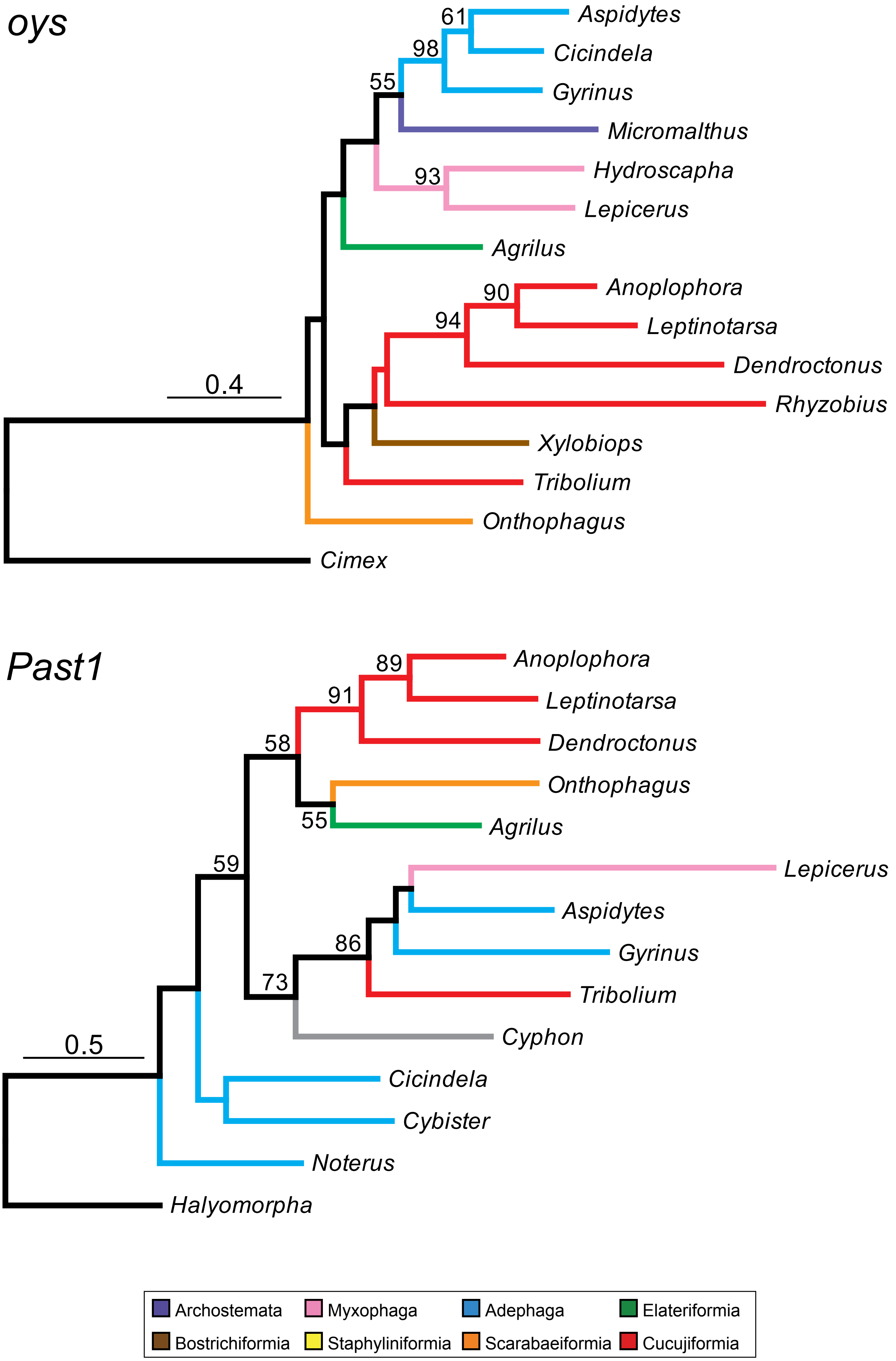
oys
61
98
55
93
90
94
0.4
Aspidytes
Cicindela
Gyrinus
Micromalthus
Hydroscapha
Lepicerus
Agrilus
Anoplophora
Leptinotarsa
Dendroctonus
Rhyzobius
Xylobiops
Tribolium
Onthophagus
Cimex
Past1
89
91
58
55
59
86
73
0.5
Anoplophora
Leptinotarsa
Dendroctonus
Onthophagus
Agrilus
Lepicerus
Aspidytes
Gyrinus
Tribolium
Cyphon
Cicindela
Cybister
Noterus
Halyomorpha
Archostemata
Myxophaga
Adephaga
Elateriformia
Bostrichiformia
Staphyliniformia
Scarabaeiformia
Cucujiformia

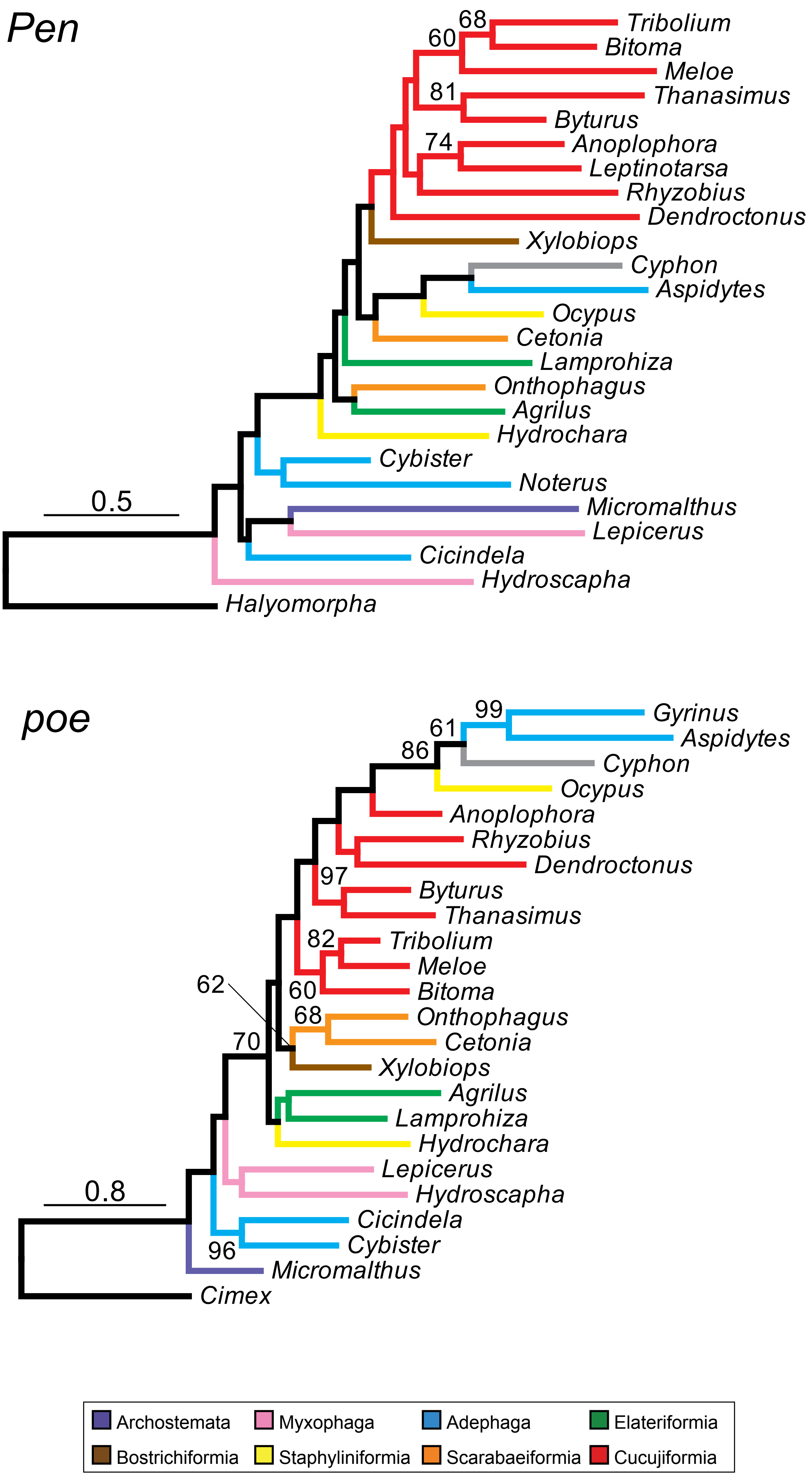
Pen
68
60
81
74
Tribolium
Bitoma
Meloe
Thanasimus
Byturus
Anoplophora
Leptinotarsa
Rhyzobius
Dendroctonus
Xylobiops
Cyphon
Aspidytes
Ocypus
Cetonia
Lamprohiza
Onthophagus
Agrilus
Hydrochara
Cybister
Noterus
0.5
Micromalthus
Lepicerus
Cicindela
Hydroscapha
Halyomorpha
poe
99
61
86
Gyrinus
Aspidytes
Cyphon
Ocypus
Anoplophora
Rhyzobius
Dendroctonus
97
Byturus
Thanasimus
82
Tribolium
Meloe
62
60
Bitoma
68
Onthophagus
70
Cetonia
Xylobiops
Agrilus
Lamprohiza
Hydrochara
Lepicerus
0.8
Hydroscapha
Cicindela
96
Cybister
Micromalthus
Cimex
Archostemata
Myxophaga
Adephaga
Elateriformia
Bostrichiformia
Staphyliniformia
Scarabaeiformia
Cucujiformia

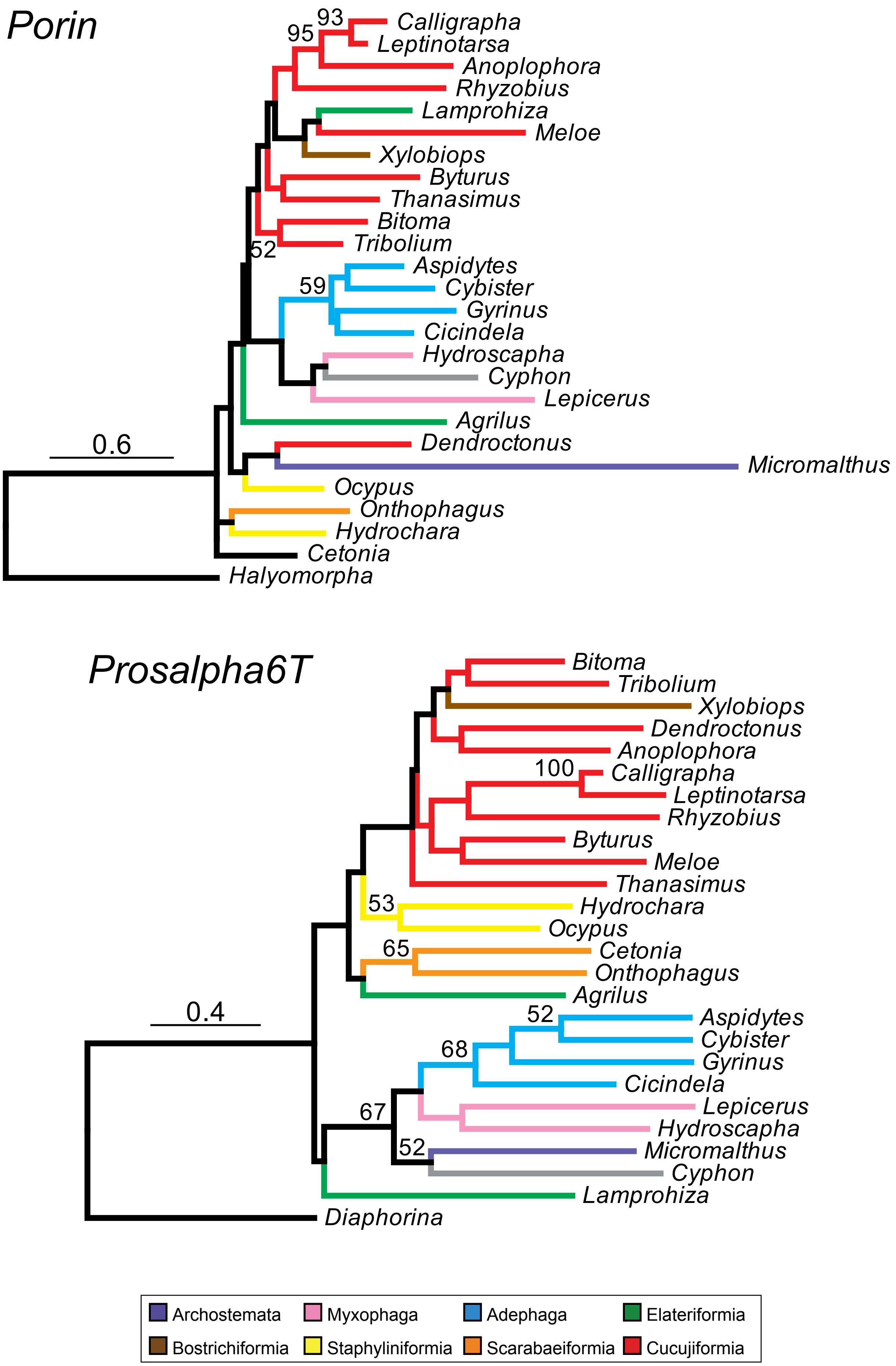
Porin
93
95
Calligrapha
Leptinotarsa
Anoplophora
Rhyzobius
Lamprohiza
Meloe
Xylobiops
Byturus
Thanasimus
Bitoma
Tribolium
52
Aspidytes
59
Cybister
Gyrinus
Cicindela
Hydroscapha
Cyphon
Lepicerus
Agrilus
0.6
Dendroctonus
Micromalthus
Ocypus
Onthophagus
Hydrochara
Cetonia
Halyomorpha
Prosalpha6T
Bitoma
Tribolium
Xylobiops
Dendroctonus
Anoplophora
100
Calligrapha
Leptinotarsa
Rhyzobius
Byturus
Meloe
Thanasimus
53
Hydrochara
Ocypus
65
Cetonia
Onthophagus
Agrilus
0.4
52
Aspidytes
Cybister
68
Gyrinus
Cicindela
67
Lepicerus
Hydroscapha
52
Micromalthus
Cyphon
Lamprohiza
Diaphorina
Archostemata
Myxophaga
Adephaga
Elateriformia
Bostrichiformia
Staphyliniformia
Scarabaeiformia
Cucujiformia

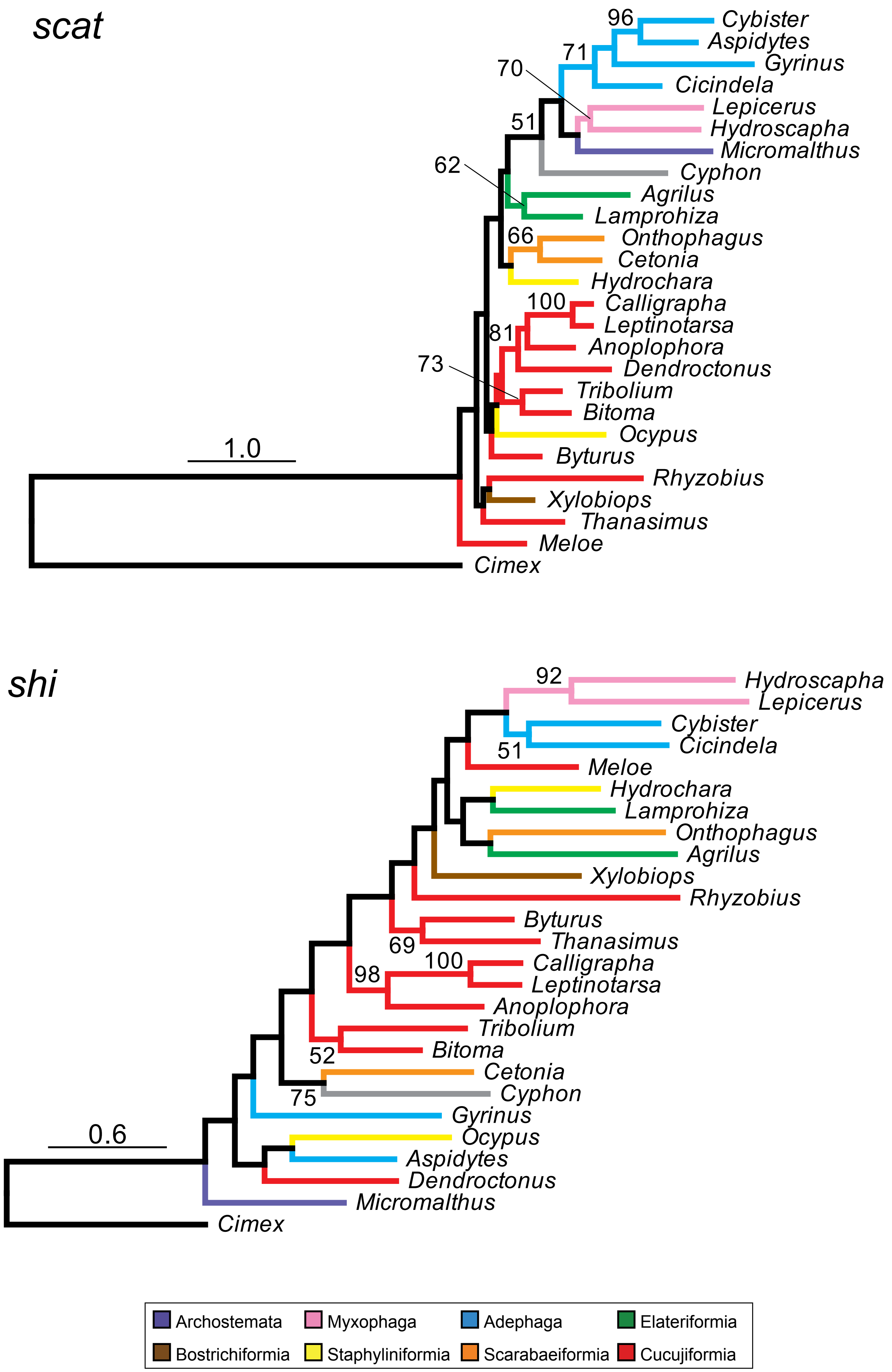
scat
96
71
70
51
62
66
100
81
73
1.0
Cybister
Aspidytes
Gyrinus
Cicindela
Lepicerus
Hydroscapha
Micromalthus
Cyphon
Agrilus
Lamprohiza
Onthophagus
Cetonia
Hydrochara
Calligrapha
Leptinotarsa
Anoplophora
Dendroctonus
Tribolium
Bitoma
Ocypus
Byturus
Rhyzobius
Xylobiops
Thanasimus
Meloe
Cimex
shi
92
51
69
100
98
52
75
0.6
Hydroscapha
Lepicerus
Cybister
Cicindela
Meloe
Hydrochara
Lamprohiza
Onthophagus
Agrilus
Xylobiops
Rhyzobius
Byturus
Thanasimus
Calligrapha
Leptinotarsa
Anoplophora
Tribolium
Bitoma
Cetonia
Cyphon
Gyrinus
Ocypus
Aspidytes
Dendroctonus
Micromalthus
Cimex
Archostemata
Myxophaga
Adephaga
Elateriformia
Bostrichiformia
Staphyliniformia
Scarabaeiformia
Cucujiformia

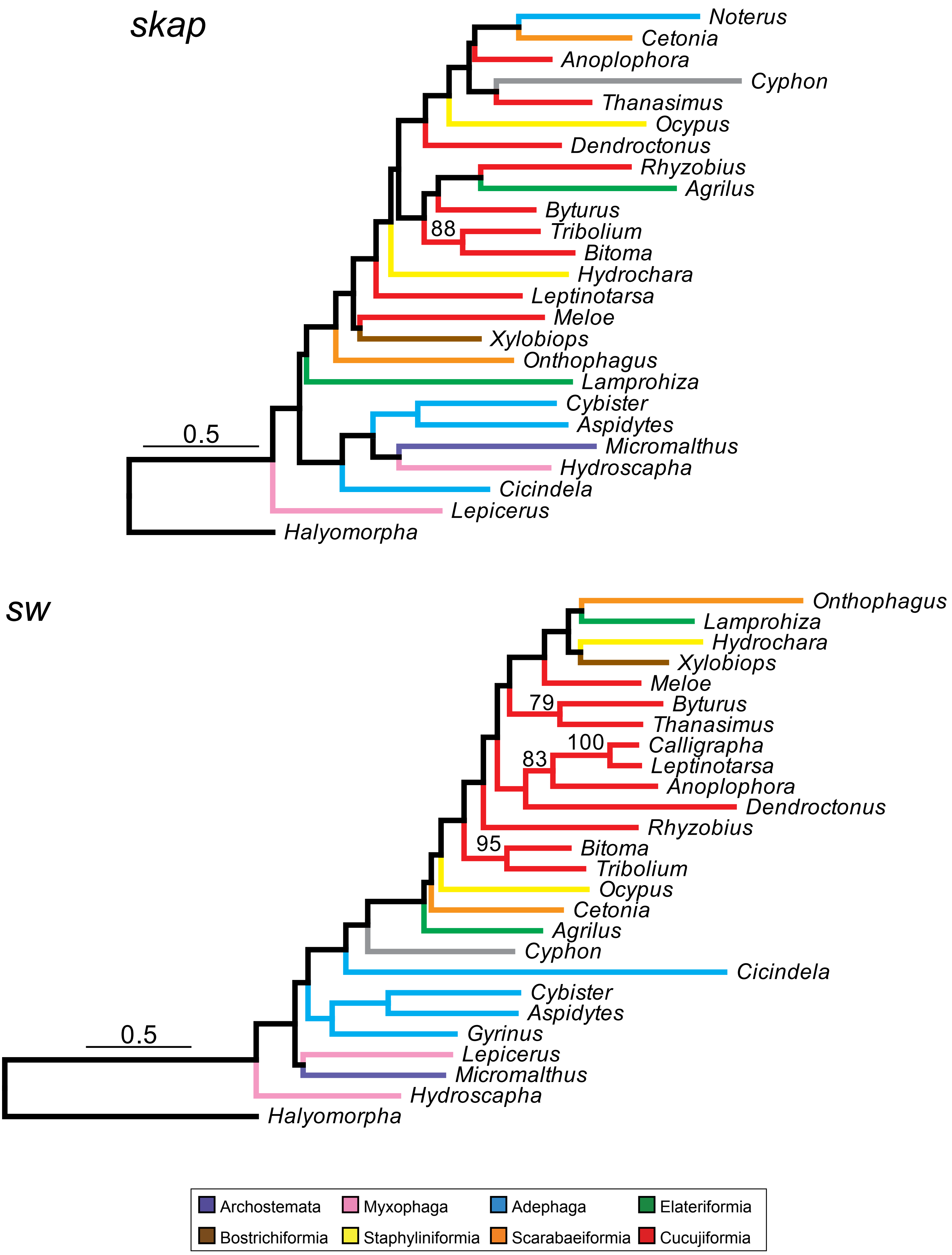
skap
Noterus
Cetonia
Anoplophora
Cyphon
Thanasimus
Ocypus
Dendroctonus
Rhyzobius
Agrilus
Byturus
88
Tribolium
Bitoma
Hydrochara
Leptinotarsa
Meloe
Xylobiops
Onthophagus
Lamprohiza
Cybister
Aspidytes
0.5
Micromalthus
Hydroscapha
Cicindela
Lepicerus
Halyomorpha
sw
Onthophagus
Lamprohiza
Hydrochara
Xylobiops
Meloe
79
Byturus
Thanasimus
100
Calligrapha
83
Leptinotarsa
Anoplophora
Dendroctonus
Rhyzobius
95
Bitoma
Tribolium
Ocypus
Cetonia
Agrilus
Cyphon
Cicindela
Cybister
Aspidytes
0.5
Gyrinus
Lepicerus
Micromalthus
Hydroscapha
Halyomorpha
Archostemata
Myxophaga
Adephaga
Elateriformia
Bostrichiformia
Staphyliniformia
Scarabaeiformia
Cucujiformia

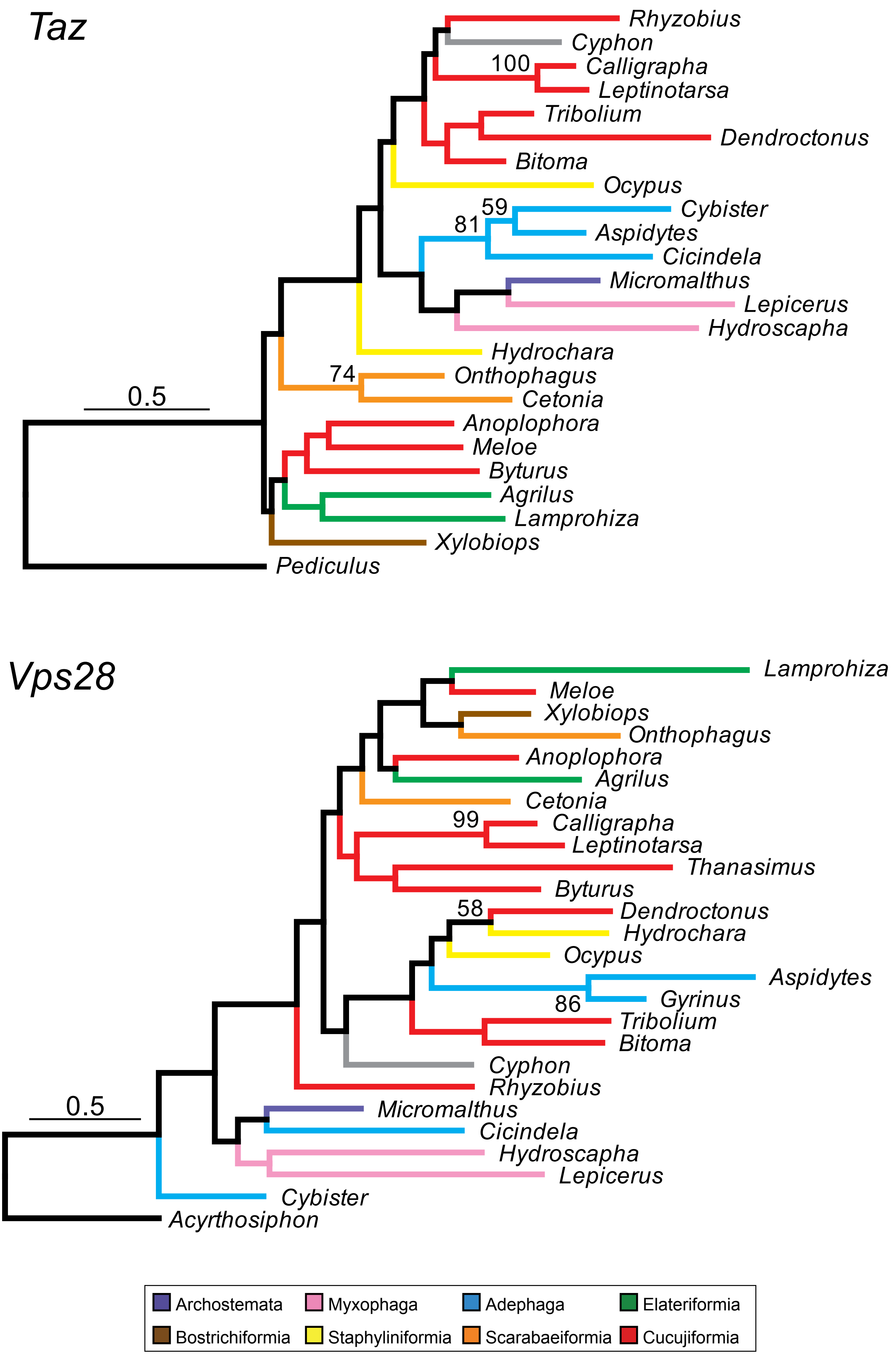
Taz
Rhyzobius
Cyphon
100
Calligrapha
Leptinotarsa
Tribolium
Dendroctonus
Bitoma
Ocypus
59
81
Cybister
Aspidytes
Cicindela
Micromalthus
Lepicerus
Hydroscapha
Hydrochara
0.5
74
Onthophagus
Cetonia
Anoplophora
Meloe
Byturus
Agrilus
Lamprohiza
Xylobiops
Pediculus
Vps28
Lamprohiza
Meloe
Xylobiops
Onthophagus
Anoplophora
Agrilus
Cetonia
99
Calligrapha
Leptinotarsa
Thanasimus
Byturus
58
Dendroctonus
Hydrochara
Ocypus
Aspidytes
86
Gyrinus
Tribolium
Bitoma
Cyphon
Rhyzobius
0.5
Micromalthus
Cicindela
Hydroscapha
Lepicerus
Cybister
Acyrthosiphon
Archostemata
Myxophaga
Adephaga
Elateriformia
Bostrichiformia
Staphyliniformia
Scarabaeiformia
Cucujiformia

File S4. Bayesian inference trees based on the nucleotide alignments of different sperm individualization genes in beetles.

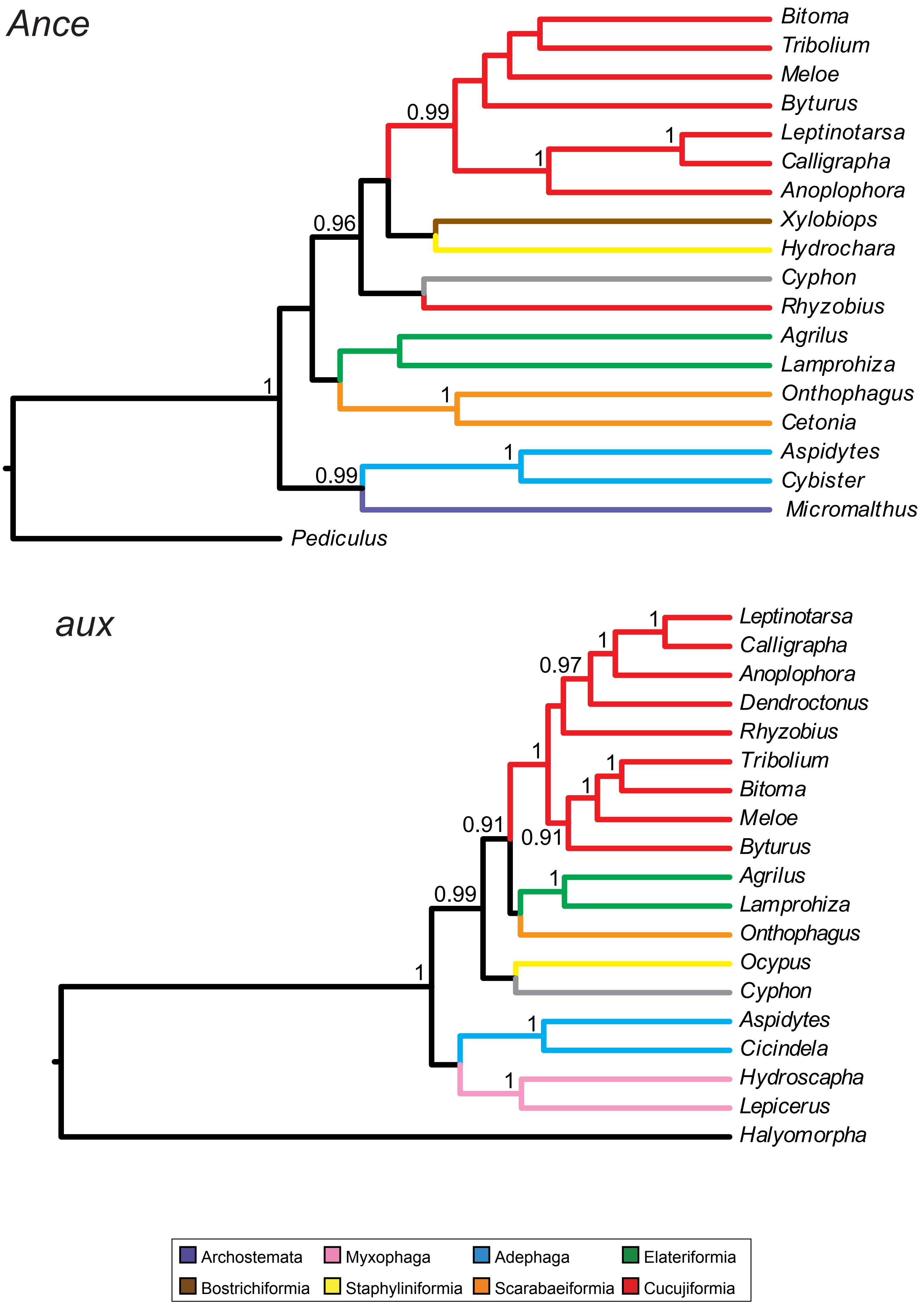

Ance
Bitoma
Tribolium
Meloe
Byturus
Leptinotarsa
Calligrapha
Anoplophora
Xylobiops
Hydrochara
Cyphon
Rhyzobius
Agrilus
Lamprohiza
Onthophagus
Cetonia
Aspidytes
Cybister
Micromalthus
Pediculus
0.99
1
1
0.96
1
1
0.99
1
aux
Leptinotarsa
Calligrapha
Anoplophora
Dendroctonus
Rhyzobius
Tribolium
Bitoma
Meloe
Byturus
Agrilus
Lamprohiza
Onthophagus
Ocypus
Cyphon
Aspidytes
Cicindela
Hydroscapha
Lepicerus
Halyomorpha
1
1
0.97
1
1
1
0.91
0.91
0.99
1
1
1
1
Archostemata
Myxophaga
Adephaga
Elateriformia
Bostrichiformia
Staphyliniformia
Scarabaeiformia
Cucujiformia

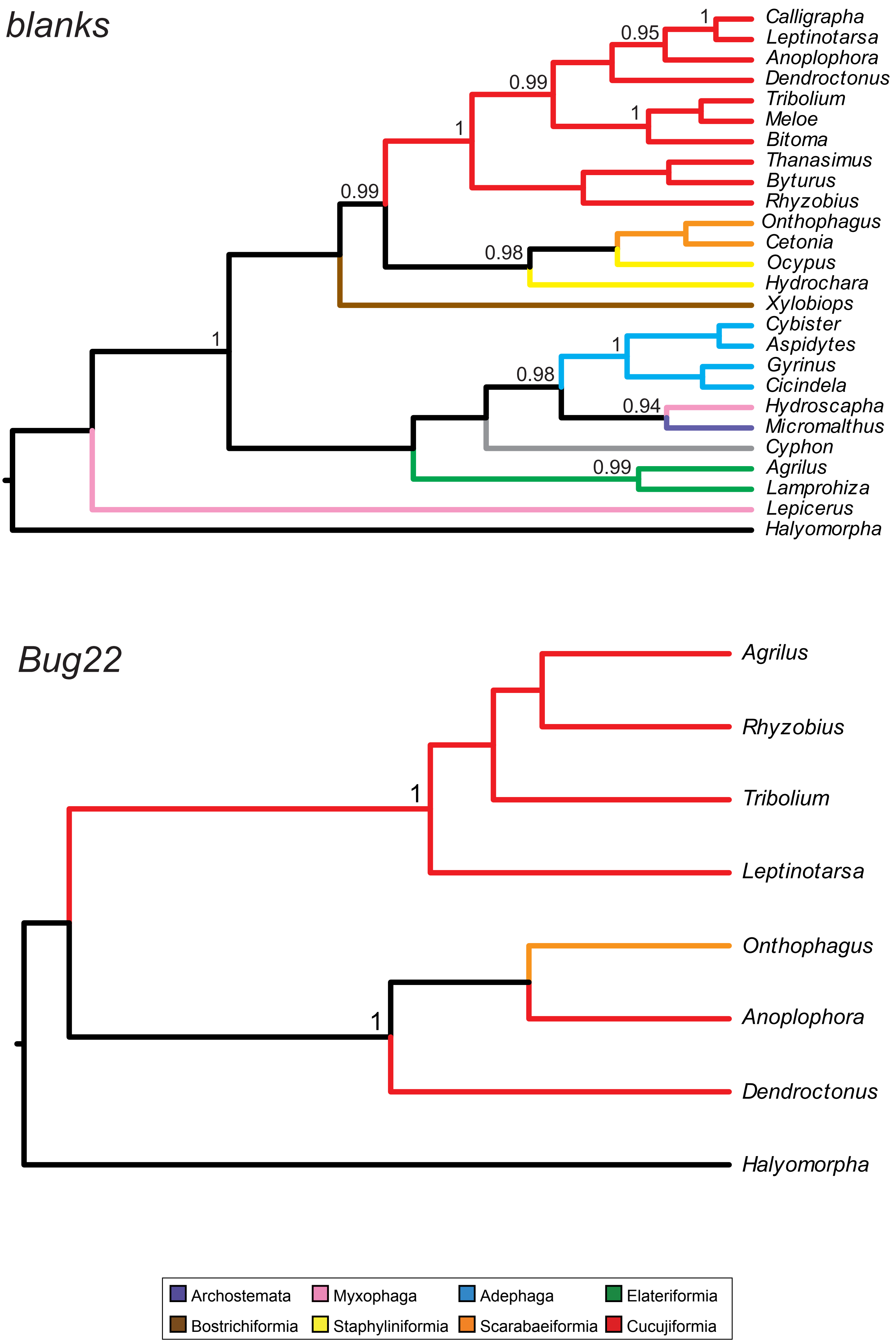
blanks
1
0.95
0.99
1
1
0.99
0.98
1
1
0.98
0.94
0.99
Calligrapha
Leptinotarsa
Anoplophora
Dendroctonus
Tribolium
Meloe
Bitoma
Thanasimus
Byturus
Rhyzobius
Onthophagus
Cetonia
Ocypus
Hydrochara
Xylobiops
Cybister
Aspidytes
Gyrinus
Cicindela
Hydroscapha
Micromalthus
Cyphon
Agrilus
Lamprohiza
Lepicerus
Halyomorpha
Bug22
1
1
Agrilus
Rhyzobius
Tribolium
Leptinotarsa
Onthophagus
Anoplophora
Dendroctonus
Halyomorpha
Archostemata
Myxophaga
Adephaga
Elateriformia
Bostrichiformia
Staphyliniformia
Scarabaeiformia
Cucujiformia

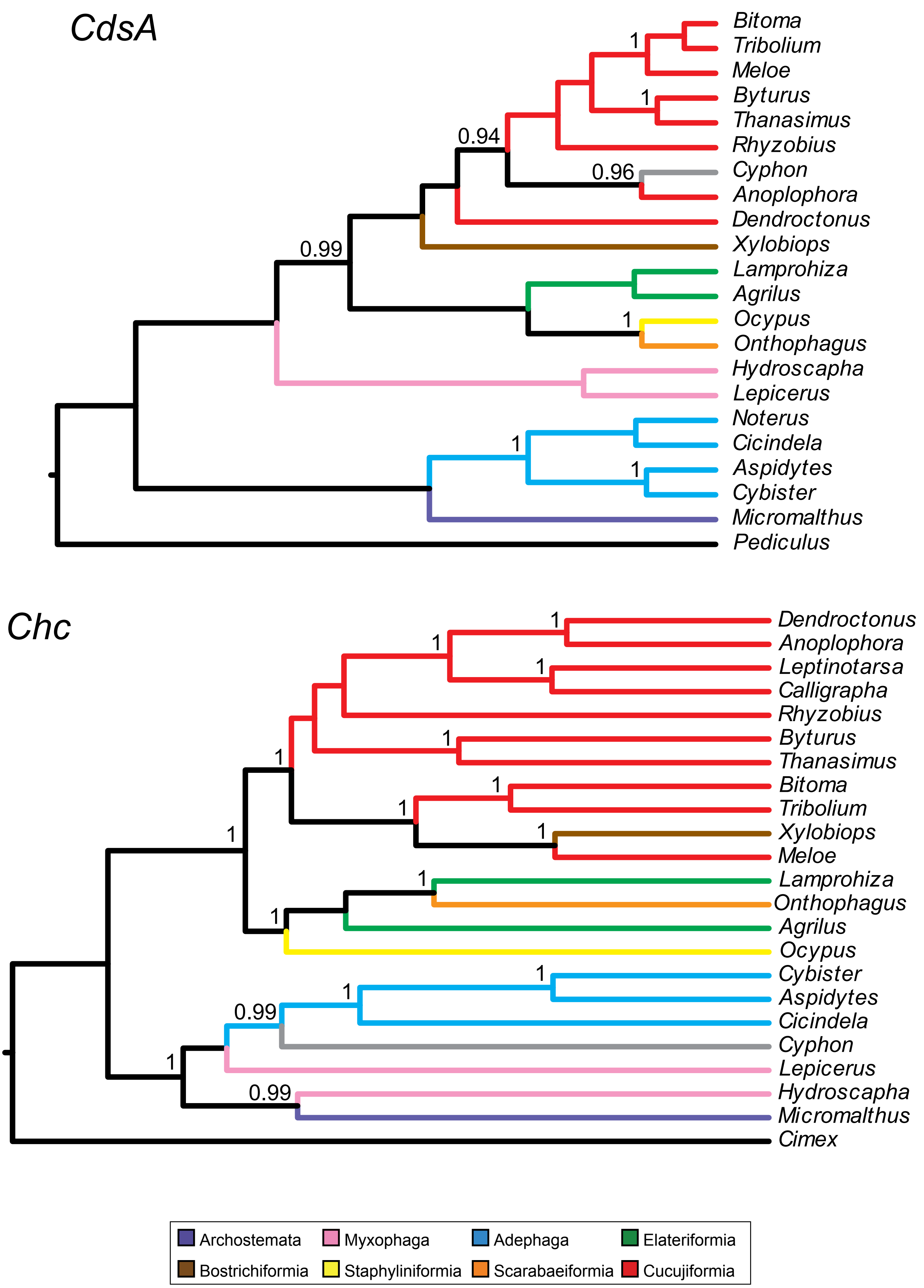
CdsA
Bitoma
Tribolium
Meloe
Byturus
Thanasimus
Rhyzobius
Cyphon
Anoplophora
Dendroctonus
Xylobiops
Lamprohiza
Agrilus
Ocypus
Onthophagus
Hydroscapha
Lepicerus
Noterus
Cicindela
Aspidytes
Cybister
Micromalthus
Pediculus
0.94
0.96
0.99
Chc
Dendroctonus
Anoplophora
Leptinotarsa
Calligrapha
Rhyzobius
Byturus
Thanasimus
Bitoma
Tribolium
Xylobiops
Meloe
Lamprohiza
Onthophagus
Agrilus
Ocypus
Cybister
Aspidytes
Cicindela
Cyphon
Lepicerus
Hydroscapha
Micromalthus
Cimex
0.99
0.99
Archostemata
Myxophaga
Adephaga
Elateriformia
Bostrichiformia
Staphyliniformia
Scarabaeiformia
Cucujiformia

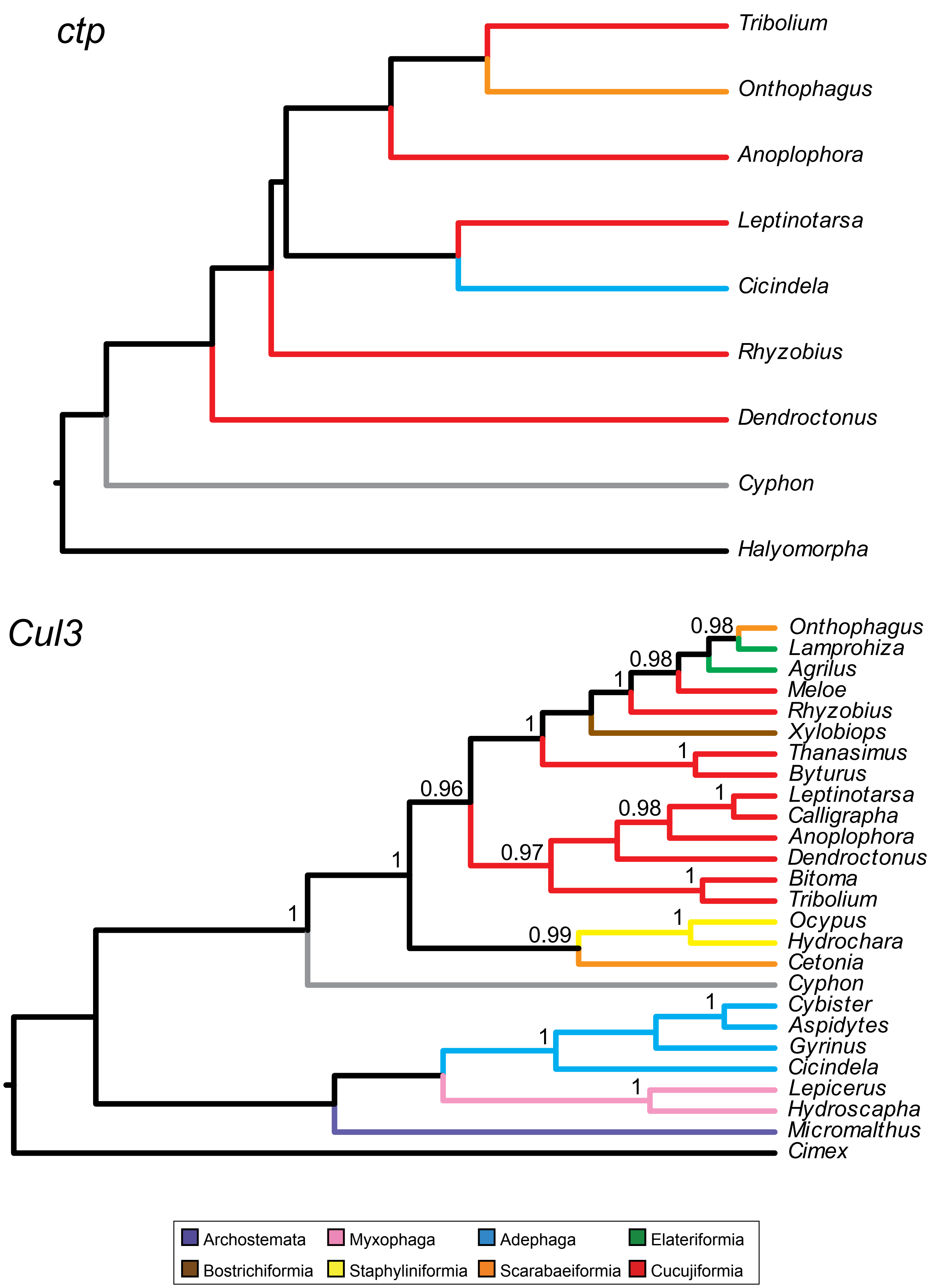

ctp
Tribolium
Onthophagus
Anoplophora
Leptinotarsa
Cicindela
Rhyzobius
Dendroctonus
Cyphon
Halyomorpha
Cul3
0.98
Onthophagus
Lamprohiza
0.98
Agrilus
1
Meloe
1
Rhyzobius
Xylobiops
1
Thanasimus
Byturus
0.96
1
Leptinotarsa
0.98
Calligrapha
Anoplophora
0.97
Dendroctonus
1
Bitoma
1
Tribolium
1
Ocypus
0.99
Hydrochara
1
Cetonia
Cyphon
1
Cybister
1
Aspidytes
Gyrinus
Cicindela
1
Lepicerus
Hydroscapha
Micromalthus
Cimex
Archostemata
Myxophaga
Adephaga
Elateriformia
Bostrichiformia
Staphyliniformia
Scarabaeiformia
Cucujiformia

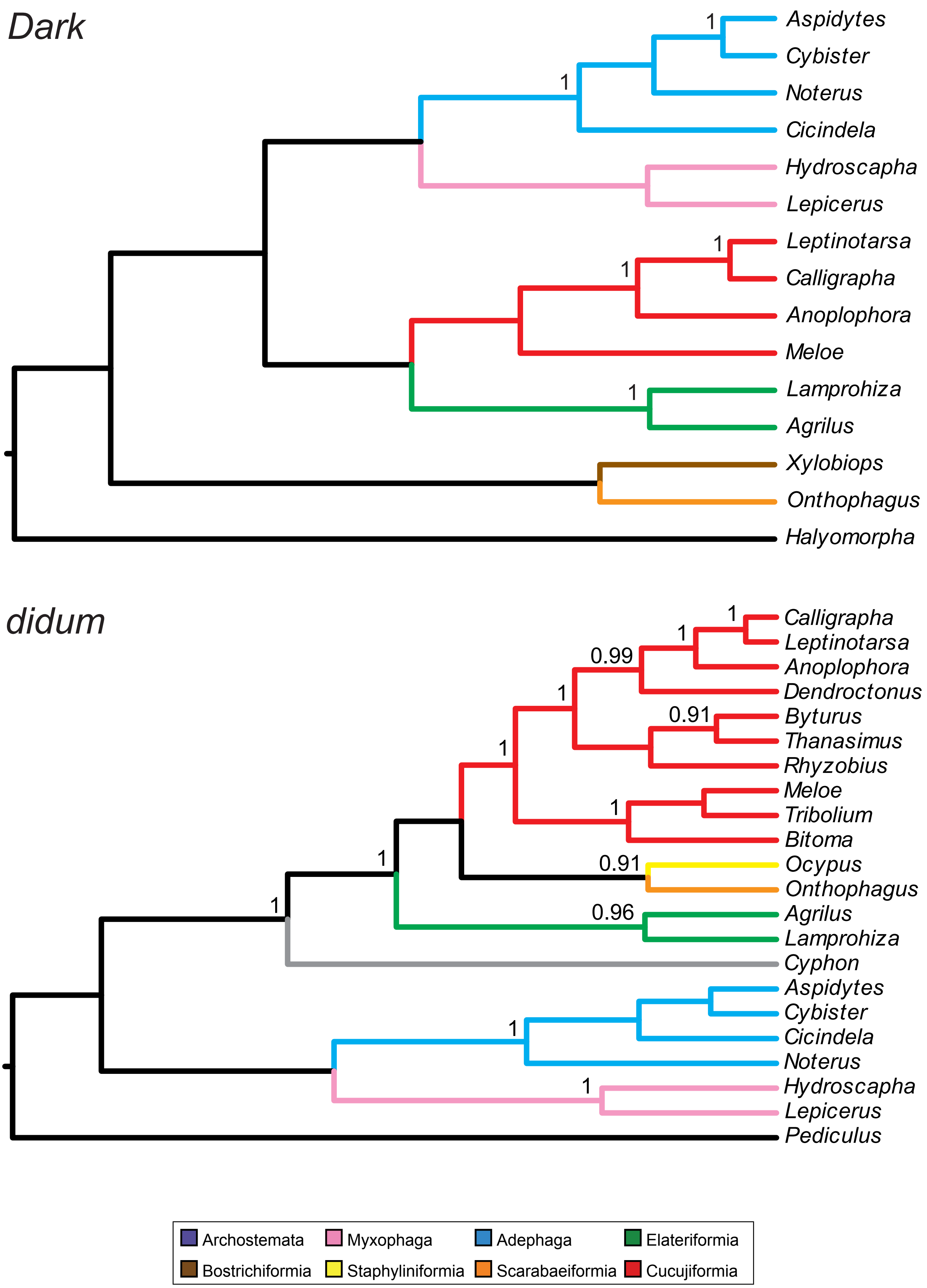

Dark
Aspidytes
Cybister
Noterus
Cicindela
Hydroscapha
Lepicerus
Leptinotarsa
Calligrapha
Anoplophora
Meloe
Lamprohiza
Agrilus
Xylobiops
Onthophagus
Halyomorpha
didum
Calligrapha
Leptinotarsa
Anoplophora
Dendroctonus
Byturus
Thanasimus
Rhyzobius
Meloe
Tribolium
Bitoma
Ocypus
Onthophagus
Agrilus
Lamprohiza
Cyphon
Aspidytes
Cybister
Cicindela
Noterus
Hydroscapha
Lepicerus
Pediculus
0.99
0.91
0.91
0.96
Archostemata
Myxophaga
Adephaga
Elateriformia
Bostrichiformia
Staphyliniformia
Scarabaeiformia
Cucujiformia

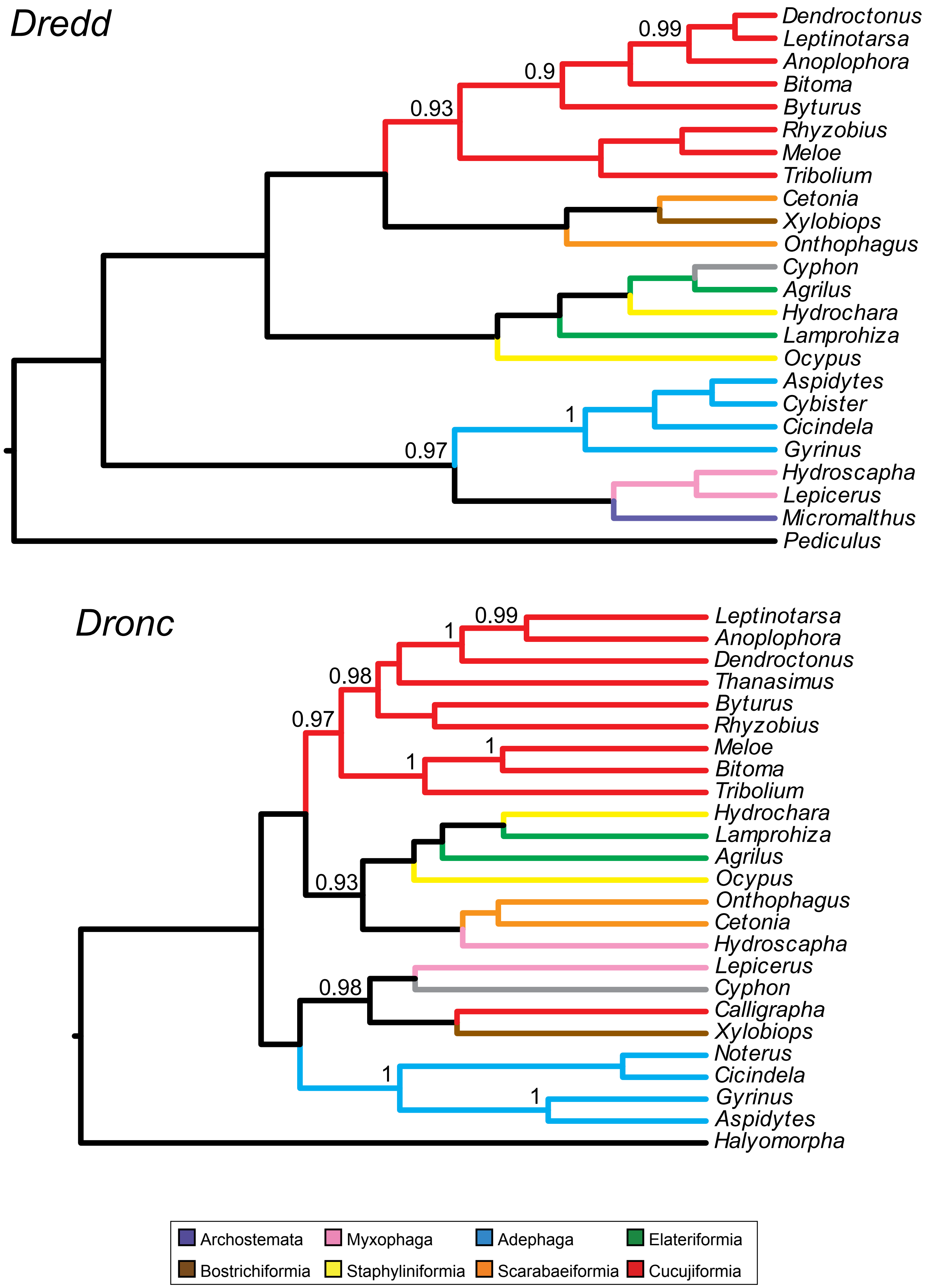
Dredd
0.99
0.9
0.93
1
0.97
Dendroctonus
Leptinotarsa
Anoplophora
Bitoma
Byturus
Rhyzobius
Meloe
Tribolium
Cetonia
Xylobiops
Onthophagus
Cyphon
Agrilus
Hydrochara
Lamprohiza
Ocypus
Aspidytes
Cybister
Cicindela
Gyrinus
Hydroscapha
Lepicerus
Micromalthus
Pediculus
Dronc
0.99
1
0.98
0.97
1
1
0.93
0.98
1
1
Leptinotarsa
Anoplophora
Dendroctonus
Thanasimus
Byturus
Rhyzobius
Meloe
Bitoma
Tribolium
Hydrochara
Lamprohiza
Agrilus
Ocypus
Onthophagus
Cetonia
Hydroscapha
Lepicerus
Cyphon
Calligrapha
Xylobiops
Noterus
Cicindela
Gyrinus
Aspidytes
Halyomorpha
Archostemata
Myxophaga
Adephaga
Elateriformia
Bostrichiformia
Staphyliniformia
Scarabaeiformia
Cucujiformia

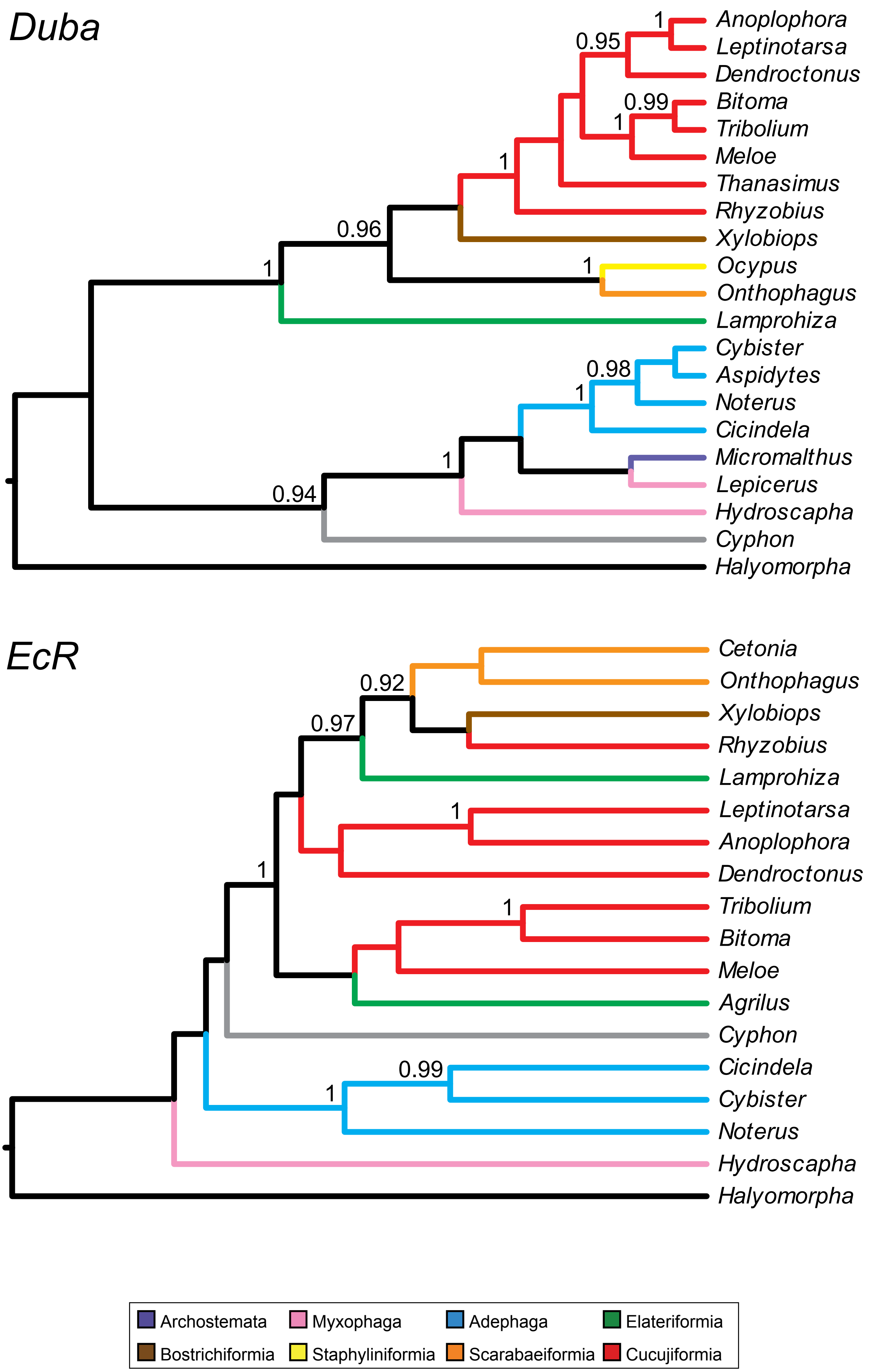
Duba
1
0.95
Anoplophora
Leptinotarsa
Dendroctonus
0.99
1
Bitoma
Tribolium
Meloe
1
Thanasimus
Rhyzobius
0.96
Xylobiops
1
1
Ocypus
Onthophagus
Lamprohiza
0.98
1
Cybister
Aspidytes
Noterus
Cicindela
1
Micromalthus
Lepicerus
0.94
Hydroscapha
Cyphon
Halyomorpha
EcR
0.92
Cetonia
Onthophagus
0.97
Xylobiops
Rhyzobius
Lamprohiza
1
Leptinotarsa
Anoplophora
1
Dendroctonus
1
Tribolium
Bitoma
Meloe
Agrilus
Cyphon
0.99
Cicindela
1
Cybister
Noterus
Hydroscapha
Halyomorpha
Archostemata
Myxophaga
Adephaga
Elateriformia
Bostrichiformia
Staphyliniformia
Scarabaeiformia
Cucujiformia

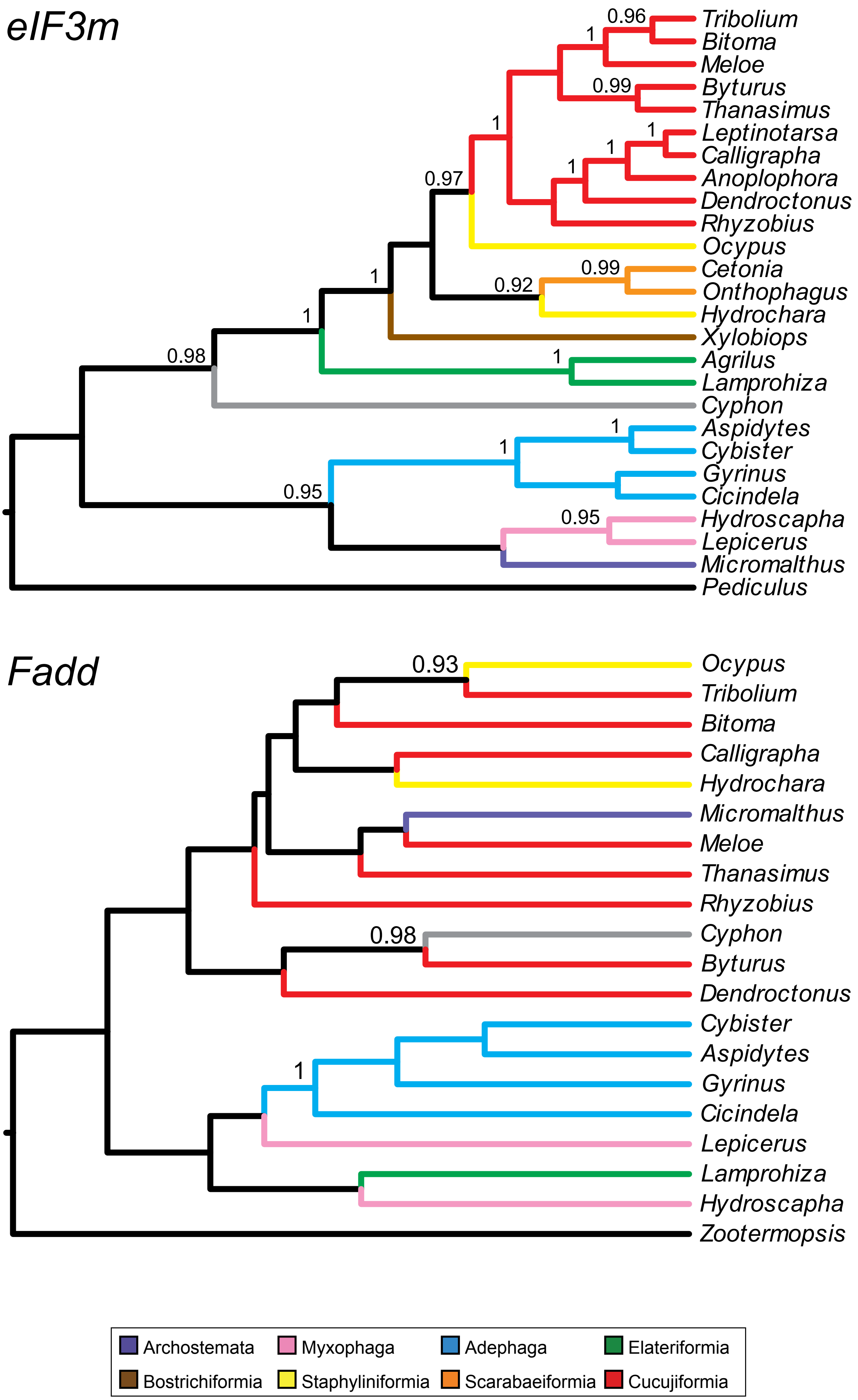
eIF3m
0.96
1
Tribolium
Bitoma
Meloe
0.99
Bytures
Thanasimus
1
1
1
1
Leptinotarsa
Calligrapha
Anoplophora
Dendroctonus
Rhyzobius
0.97
Ocypus
0.99
Cetonia
Onthophagus
0.92
Hydrochara
1
Xylobiops
1
1
Agrilus
Lamprohiza
0.98
Cyphon
1
Aspidytes
1
Cybister
Gyrinus
Cicindela
0.95
0.95
Hydroscapha
Lepicerus
Micromalthus
Pediculus
Fadd
0.93
Ocypus
Tribolium
Bitoma
Calligrapha
Hydrochara
Micromalthus
Meloe
Thanasimus
Rhyzobius
0.98
Cyphon
Byturus
Dendroctonus
Cybister
Aspidytes
Gyrinus
1
Cicindela
Lepicerus
Lamprohiza
Hydroscapha
Zootermopsis
Archostemata
Myxophaga
Adephaga
Elateriformia
Bostrichiformia
Staphyliniformia
Scarabaeiformia
Cucujiformia

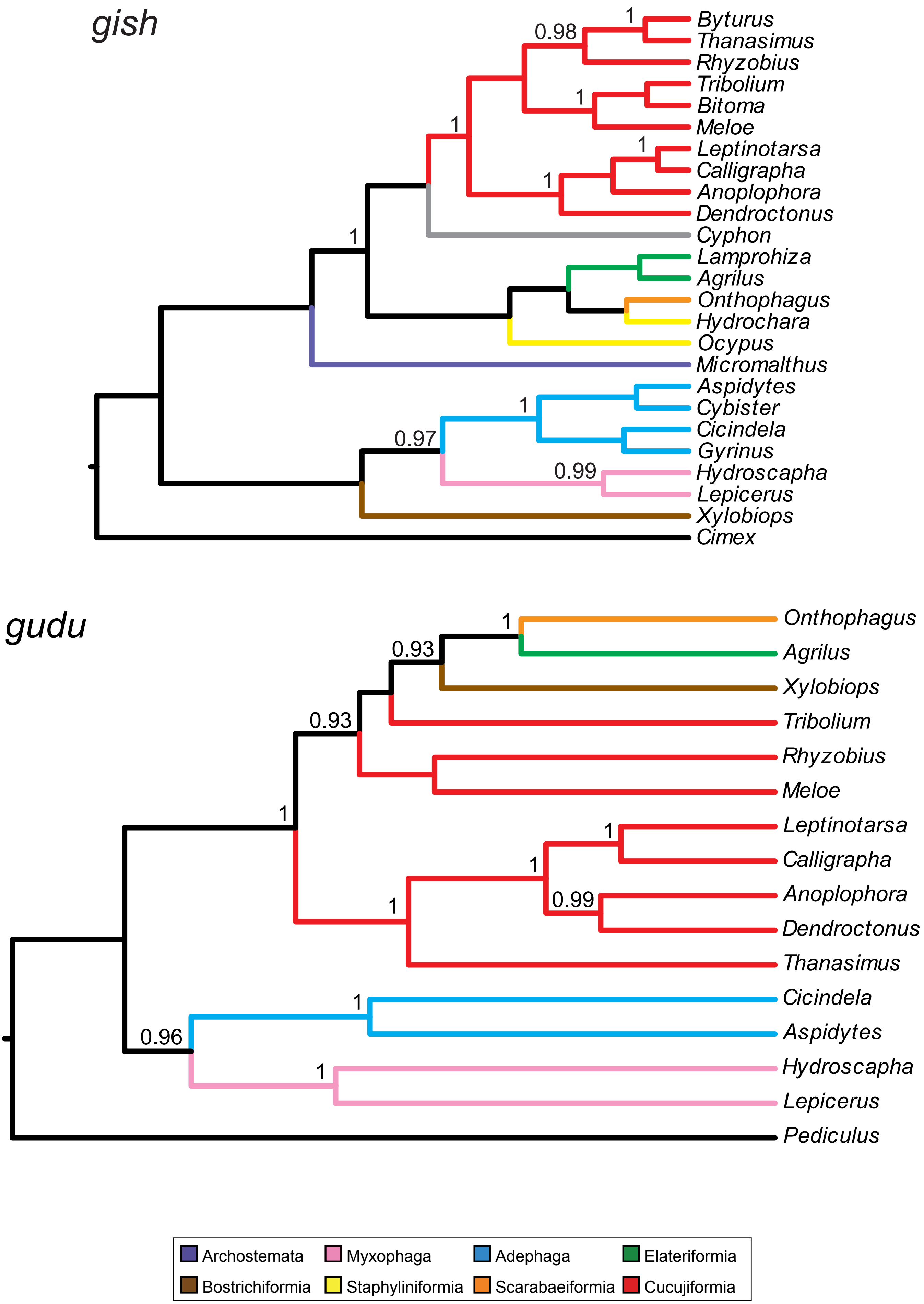
gish
Byturus
Thanasimus
Rhyzobius
Tribolium
Bitoma
Meloe
Leptinotarsa
Calligrapha
Anoplophora
Dendroctonus
Cyphon
Lamprohiza
Agrilus
Onthophagus
Hydrochara
Ocypus
Micromalthus
Aspidytes
Cybister
Cicindela
Gyrinus
Hydroscapha
Lepicerus
Xylobiops
Cimex
1
0.98
1
1
1
1
1
0.97
1
0.99
gudu
Onthophagus
Agrilus
Xylobiops
Tribolium
Rhyzobius
Meloe
Leptinotarsa
Calligrapha
Anoplophora
Dendroctonus
Thanasimus
Cicindela
Aspidytes
Hydroscapha
Lepicerus
Pediculus
1
0.93
0.93
1
1
1
1
0.99
0.96
1
1
Archostemata
Myxophaga
Adephaga
Elateriformia
Bostrichiformia
Staphyliniformia
Scarabaeiformia
Cucujiformia

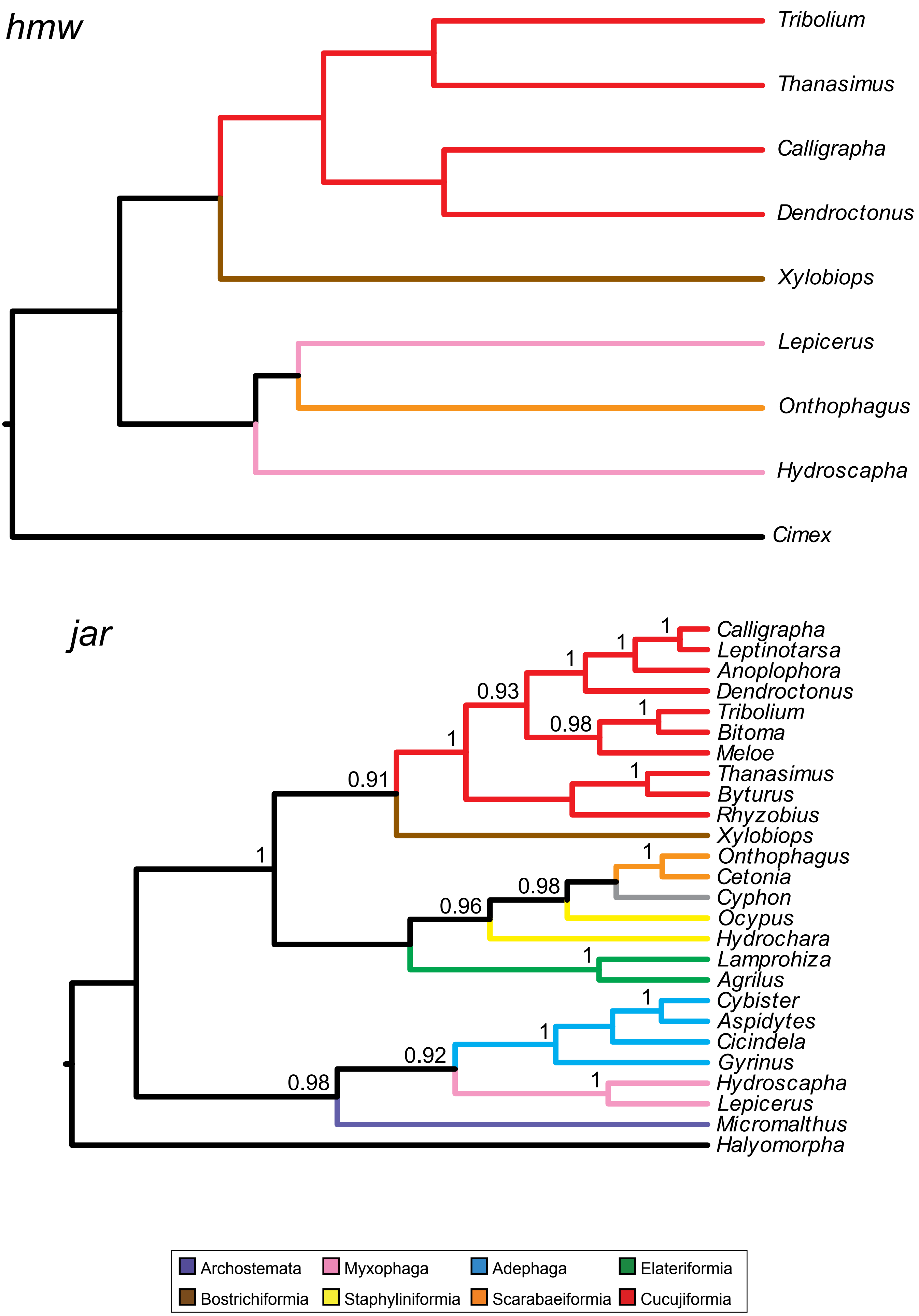
hmw
Tribolium
Thanasimus
Calligrapha
Dendroctonus
Xylobiops
Lepicerus
Onthophagus
Hydroscapha
Cimex
jar
Calligrapha
Leptinotarsa
Anoplophora
Dendroctonus
Tribolium
Bitoma
Meloe
Thanasimus
Byturus
Rhyzobius
Xylobiops
Onthophagus
Cetonia
Cyphon
Ocypus
Hydrochara
Lamprohiza
Agrilus
Cybister
Aspidytes
Cicindela
Gyrinus
Hydroscapha
Lepicerus
Micromalthus
Halyomorpha
1
1
1
0.93
0.98
1
1
1
0.91
1
1
0.98
0.96
1
1
1
0.92
1
0.98
Archostemata
Myxophaga
Adephaga
Elateriformia
Bostrichiformia
Staphyliniformia
Scarabaeiformia
Cucujiformia

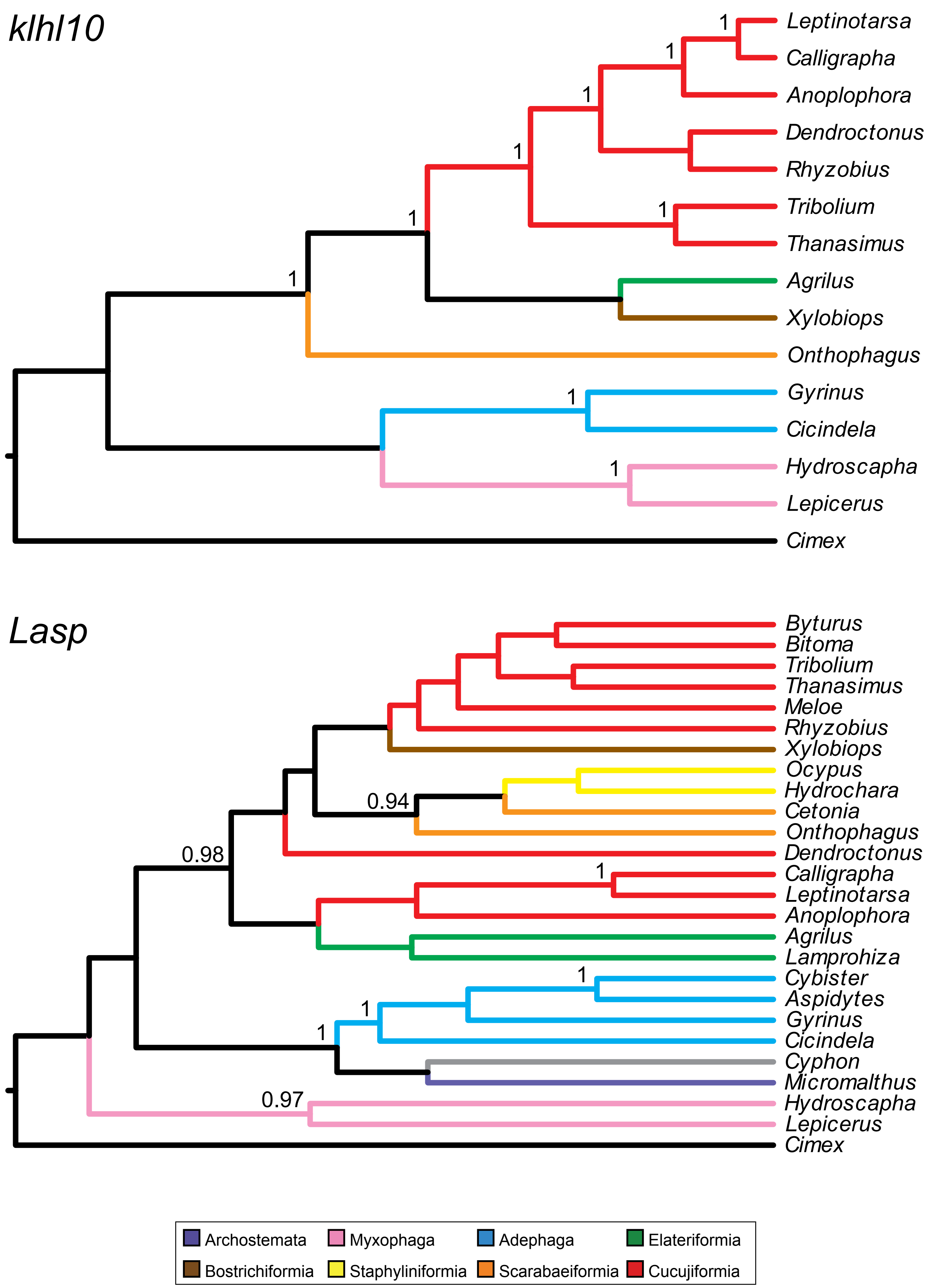
klhl10
Leptinotarsa
Calligrapha
Anoplophora
Dendroctonus
Rhyzobius
Tribolium
Thanasimus
Agrilus
Xylobiops
Onthophagus
Gyrinus
Cicindela
Hydroscapha
Lepicerus
Cimex
Lasp
Byturus
Bitoma
Tribolium
Thanasimus
Meloe
Rhyzobius
Xylobiops
Ocypus
Hydrochara
Cetonia
Onthophagus
Dendroctonus
Calligrapha
Leptinotarsa
Anoplophora
Agrilus
Lamprohiza
Cybister
Aspidytes
Gyrinus
Cicindela
Cyphon
Micromalthus
Hydroscapha
Lepicerus
Cimex
0.94
0.98
0.97
Archostemata
Myxophaga
Adephaga
Elateriformia
Bostrichiformia
Staphyliniformia
Scarabaeiformia
Cucujiformia

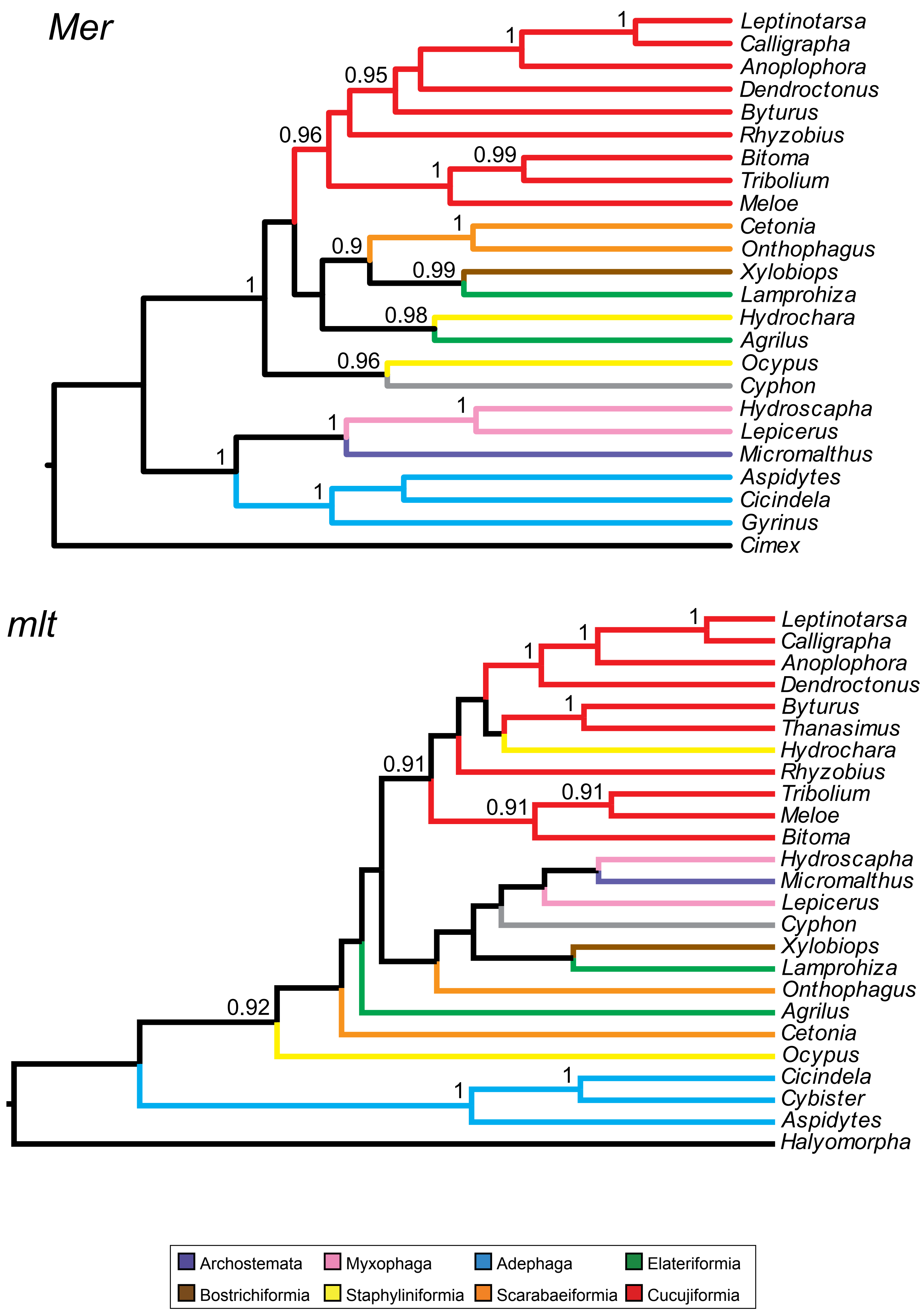

Mer
Leptinotarsa
Calligrapha
Anoplophora
Dendroctonus
Byturus
Rhyzobius
Bitoma
Tribolium
Meloe
Cetonia
Onthophagus
Xylobiops
Lamprohiza
Hydrochara
Agrilus
Ocypus
Cyphon
Hydroscapha
Lepicerus
Micromalthus
Aspidytes
Cicindela
Gyrinus
Cimex
1
1
0.95
0.96
0.99
1
1
0.9
0.99
1
0.98
0.96
1
1
1
1
mlt
Leptinotarsa
Calligrapha
Anoplophora
Dendroctonus
Byturus
Thanasimus
Hydrochara
Rhyzobius
Tribolium
Meloe
Bitoma
Hydroscapha
Micromalthus
Lepicerus
Cyphon
Xylobiops
Lamprohiza
Onthophagus
Agrilus
Cetonia
Ocypus
Cicindela
Cybister
Aspidytes
Halyomorpha
1
1
1
1
0.91
0.91
0.91
0.92
1
1
Archostemata
Myxophaga
Adephaga
Elateriformia
Bostrichiformia
Staphyliniformia
Scarabaeiformia
Cucujiformia

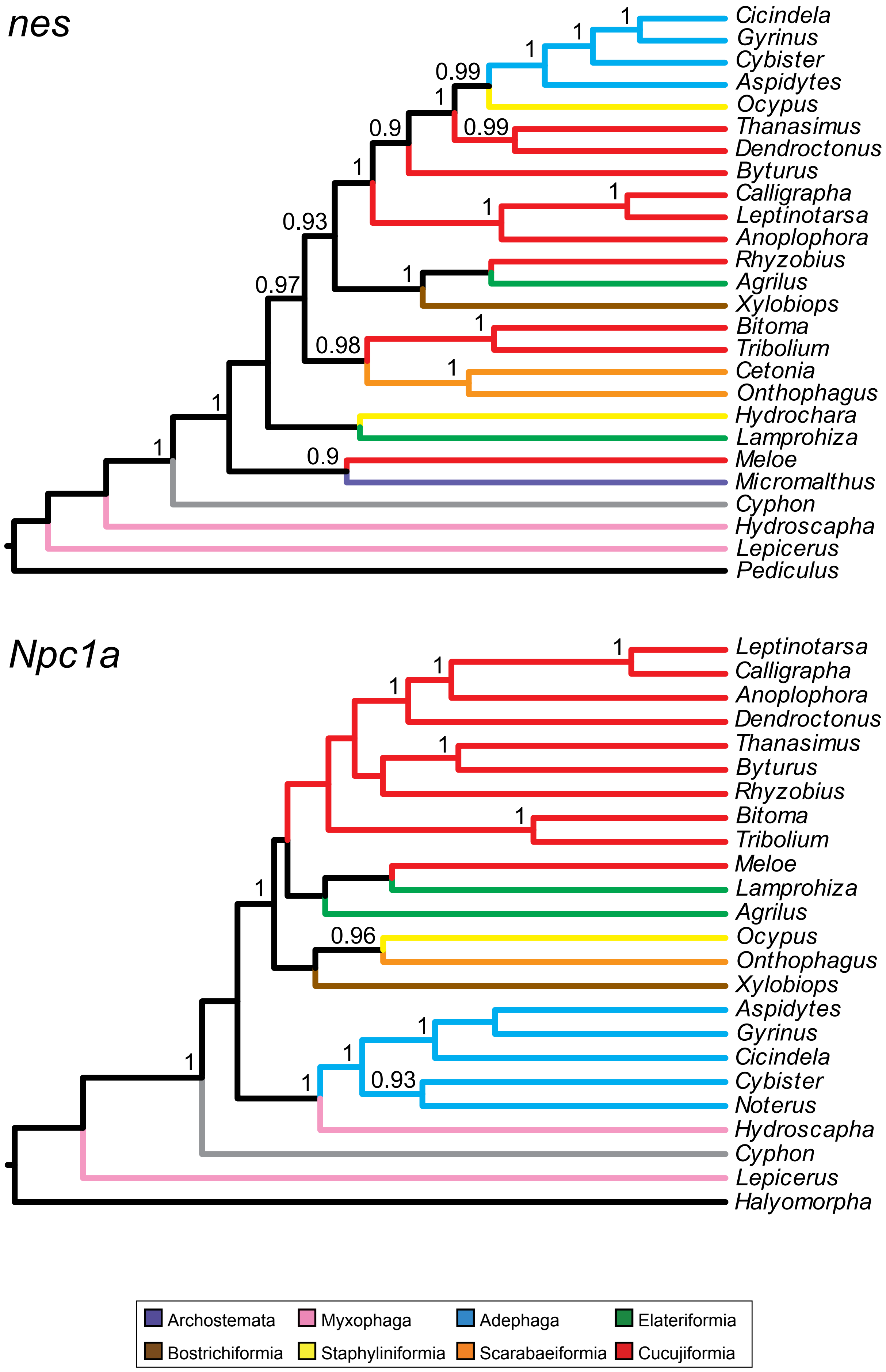

nes
Cicindela
Gyrinus
Cybister
Aspidytes
Ocypus
Thanasimus
Dendroctonus
Byturus
Calligrapha
Leptinotarsa
Anoplophora
Rhyzobius
Agrilus
Xylobiops
Bitoma
Tribolium
Cetonia
Onthophagus
Hydrochara
Lamprohiza
Meloe
Micromalthus
Cyphon
Hydroscapha
Lepicerus
Pediculus
Npc1a
Leptinotarsa
Calligrapha
Anoplophora
Dendroctonus
Thanasimus
Byturus
Rhyzobius
Bitoma
Tribolium
Meloe
Lamprohiza
Agrilus
Ocypus
Onthophagus
Xylobiops
Aspidytes
Gyrinus
Cicindela
Cybister
Noterus
Hydroscapha
Cyphon
Lepicerus
Halyomorpha
Archostemata
Myxophaga
Adephaga
Elateriformia
Bostrichiformia
Staphyliniformia
Scarabaeiformia
Cucujiformia

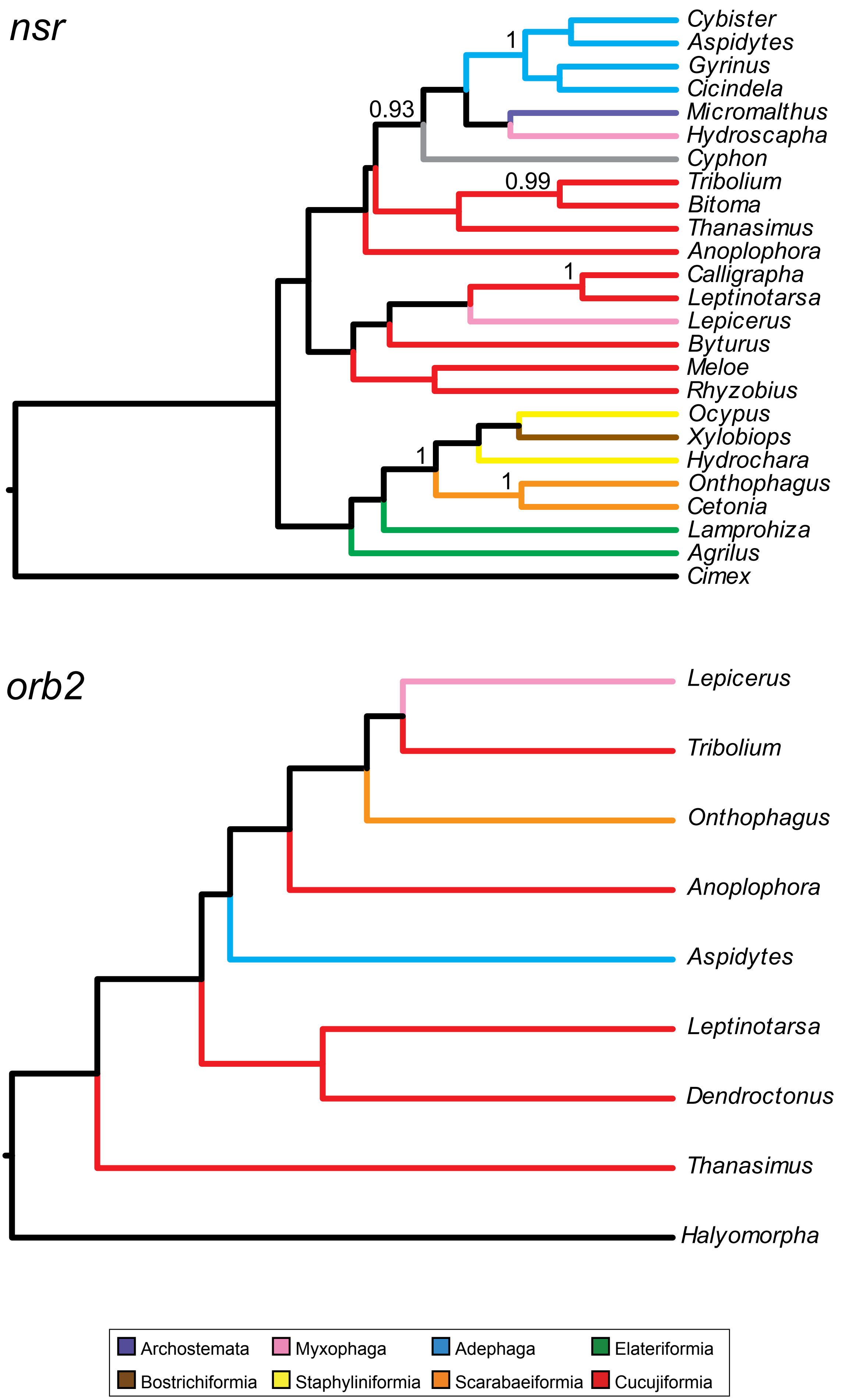

nsr
1
0.93
0.99
1
1
1
Cybister
Aspidytes
Gyrinus
Cicindela
Micromalthus
Hydroscapha
Cyphon
Tribolium
Bitoma
Thanasimus
Anoplophora
Calligrapha
Leptinotarsa
Lepicerus
Byturus
Meloe
Rhyzobius
Ocypus
Xylobiops
Hydrochara
Onthophagus
Cetonia
Lamprohiza
Agrilus
Cimex
orb2
Lepicerus
Tribolium
Onthophagus
Anoplophora
Aspidytes
Leptinotarsa
Dendroctonus
Thanasimus
Halyomorpha
Archostemata
Myxophaga
Adephaga
Elateriformia
Bostrichiformia
Staphyliniformia
Scarabaeiformia
Cucujiformia

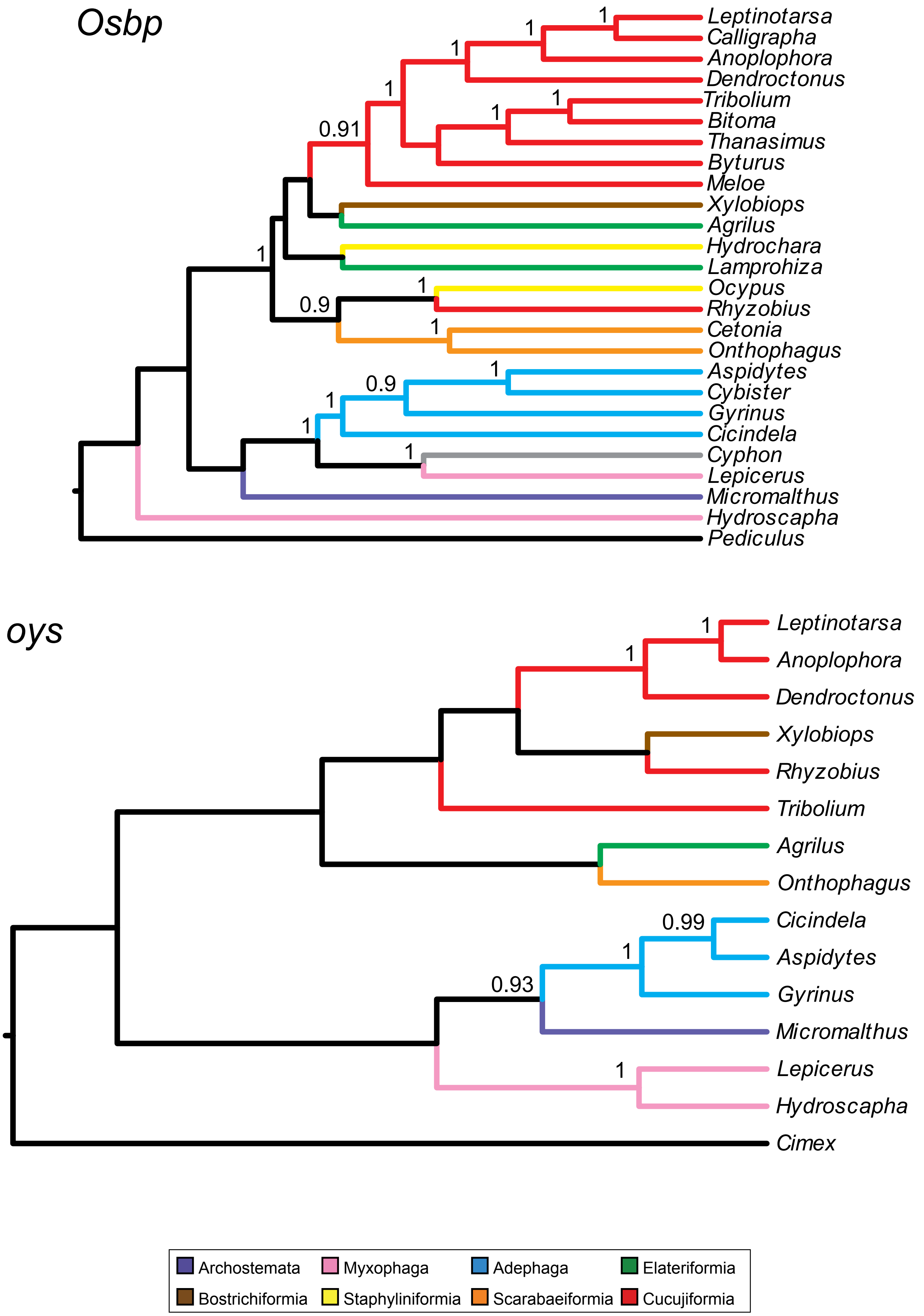
Osbp
Leptinotarsa
Calligrapha
Anoplophora
Dendroctonus
Tribolium
Bitoma
Thanasimus
Byturus
Meloe
Xylobiops
Agrilus
Hydrochara
Lamprohiza
Ocypus
Rhyzobius
Cetonia
Onthophagus
Aspidytes
Cybister
Gyrinus
Cicindela
Cyphon
Lepicerus
Micromalthus
Hydroscapha
Pediculus
0.91
0.9
0.9
oys
Leptinotarsa
Anoplophora
Dendroctonus
Xylobiops
Rhyzobius
Tribolium
Agrilus
Onthophagus
Cicindela
Aspidytes
Gyrinus
Micromalthus
Lepicerus
Hydroscapha
Cimex
0.99
0.93
Archostemata
Myxophaga
Adephaga
Elateriformia
Bostrichiformia
Staphyliniformia
Scarabaeiformia
Cucujiformia

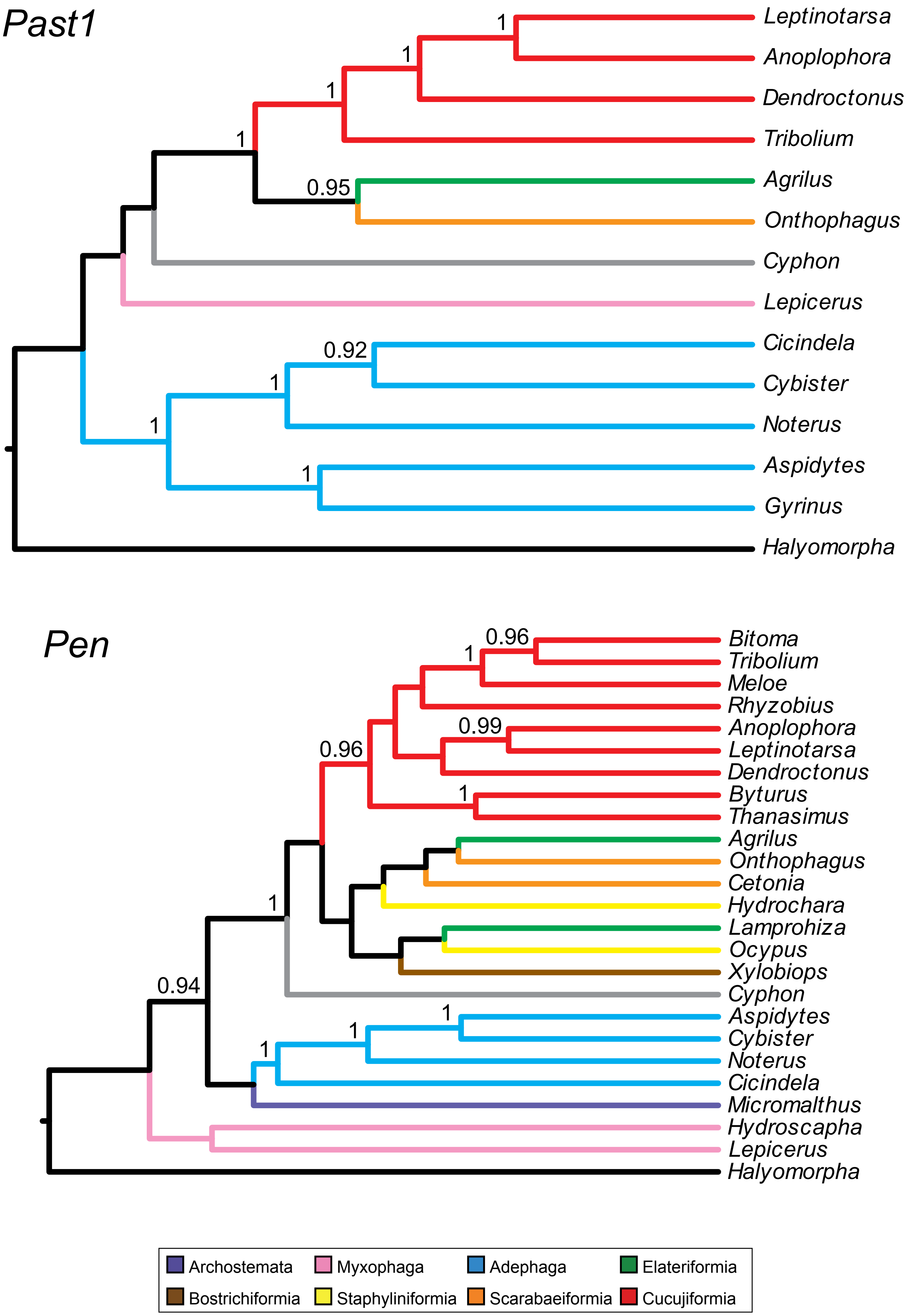

Past1
1
1
1
1
0.95
0.92
1
1
1
Leptinotarsa
Anoplophora
Dendroctonus
Tribolium
Agrilus
Onthophagus
Cyphon
Lepicerus
Cicindela
Cybister
Noterus
Aspidytes
Gyrinus
Halyomorpha
Pen
0.96
1
0.99
0.96
1
1
0.94
1
1
1
Bitoma
Tribolium
Meloe
Rhyzobius
Anoplophora
Leptinotarsa
Dendroctonus
Byturus
Thanasimus
Agrilus
Onthophagus
Cetonia
Hydrochara
Lamprohiza
Ocypus
Xylobiops
Cyphon
Aspidytes
Cybister
Noterus
Cicindela
Micromalthus
Hydroscapha
Lepicerus
Halyomorpha
Archostemata
Myxophaga
Adephaga
Elateriformia
Bostrichiformia
Staphyliniformia
Scarabaeiformia
Cucujiformia

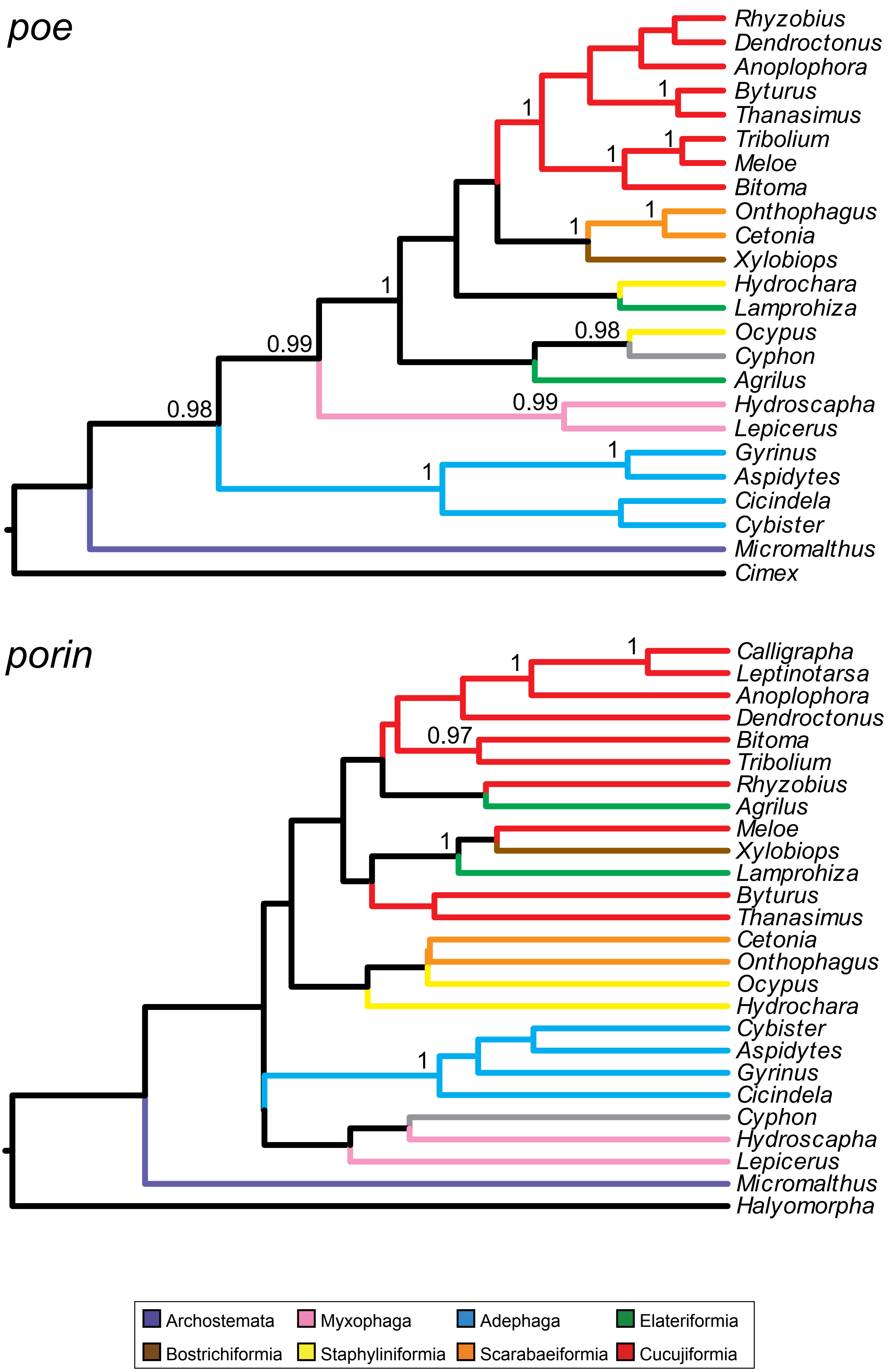

poe
Rhyzobius
Dendroctonus
Anoplophora
Byturus
Thanasimus
Tribolium
Meloe
Bitoma
Onthophagus
Cetonia
Xylobiops
Hydrochara
Lamprohiza
Ocypus
Cyphon
Agrilus
Hydroscapha
Lepicerus
Gyrinus
Aspidytes
Cicindela
Cybister
Micromalthus
Cimex
0.98
0.99
0.99
0.98
porin
Calligrapha
Leptinotarsa
Anoplophora
Dendroctonus
Bitoma
Tribolium
Rhyzobius
Agrilus
Meloe
Xylobiops
Lamprohiza
Byturus
Thanasimus
Cetonia
Onthophagus
Ocypus
Hydrochara
Cybister
Aspidytes
Gyrinus
Cicindela
Cyphon
Hydroscapha
Lepicerus
Micromalthus
Halyomorpha
0.97
Archostemata
Myxophaga
Adephaga
Elateriformia
Bostrichiformia
Staphyliniformia
Scarabaeiformia
Cucujiformia

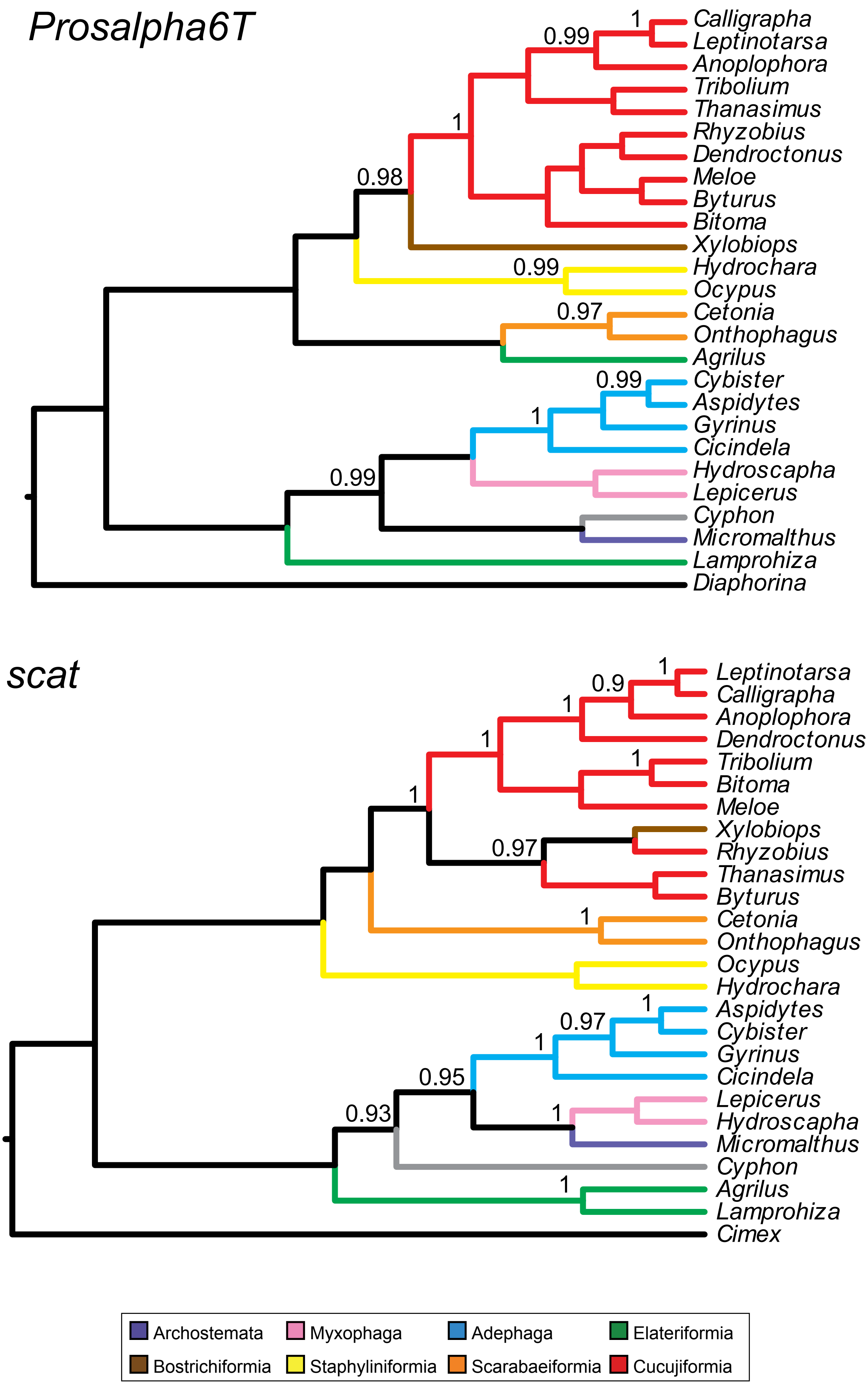

Prosalpha6T
1
0.99
1
0.98
0.99
0.97
0.99
1
0.99
Calligrapha
Leptinotarsa
Anoplophora
Tribolium
Thanasimus
Rhyzobius
Dendroctonus
Meloe
Byturus
Bitoma
Xylobiops
Hydrochara
Ocypus
Cetonia
Onthophagus
Agrilus
Cybister
Aspidytes
Gyrinus
Cicindela
Hydroscapha
Lepicerus
Cyphon
Micromalthus
Lamprohiza
Diaphorina
scat
1
0.9
1
1
1
1
0.97
1
1
0.97
1
0.95
1
0.93
1
Leptinotarsa
Calligrapha
Anoplophora
Dendroctonus
Tribolium
Bitoma
Meloe
Xylobiops
Rhyzobius
Thanasimus
Byturus
Cetonia
Onthophagus
Ocypus
Hydrochara
Aspidytes
Cybister
Gyrinus
Cicindela
Lepicerus
Hydroscapha
Micromalthus
Cyphon
Agrilus
Lamprohiza
Cimex
Archostemata
Myxophaga
Adephaga
Elateriformia
Bostrichiformia
Staphyliniformia
Scarabaeiformia
Cucujiformia

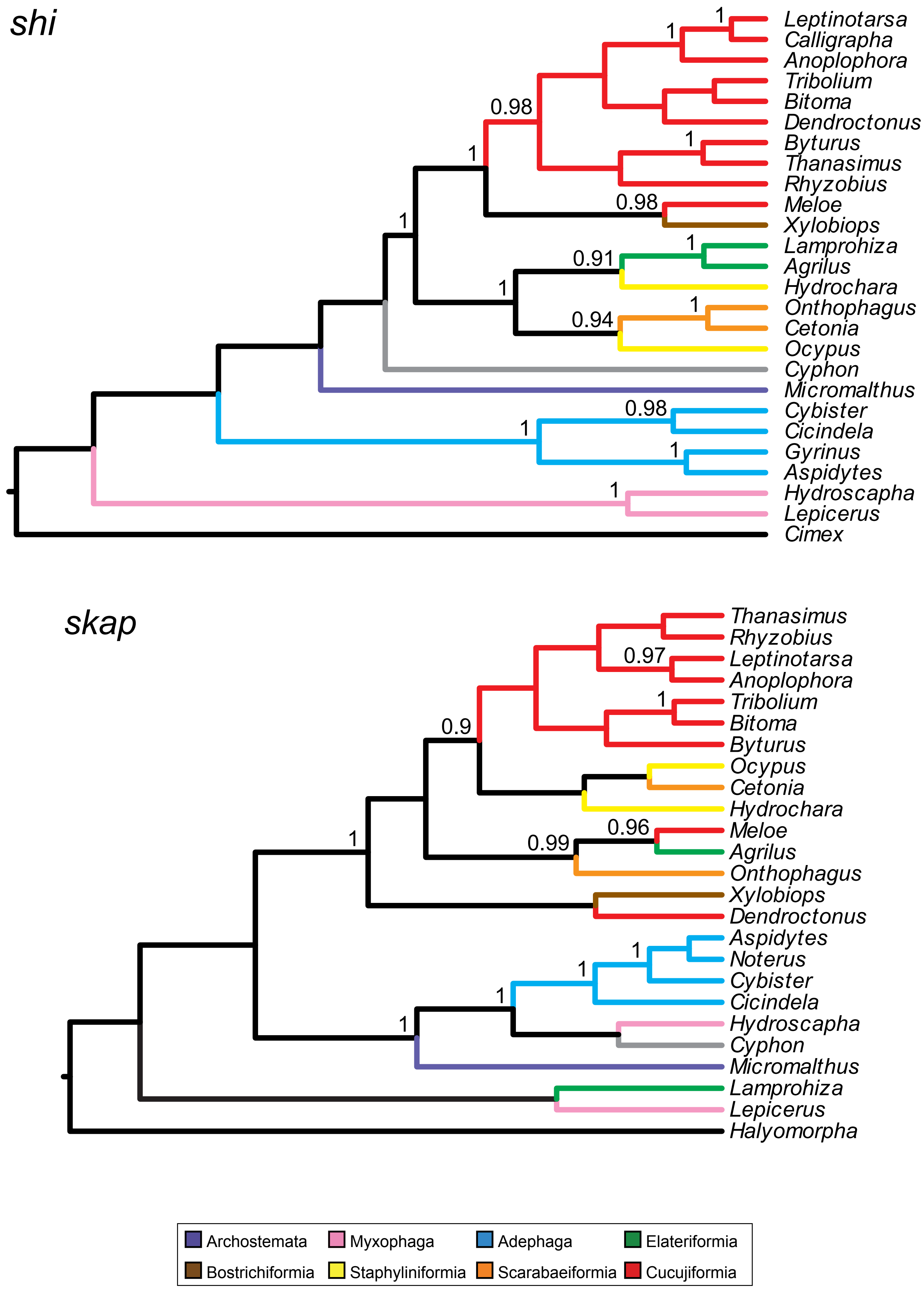

shi
Leptinotarsa
Calligrapha
Anoplophora
Tribolium
Bitoma
Dendroctonus
Byturus
Thanasimus
Rhyzobius
Meloe
Xylobiops
Lamprohiza
Agrilus
Hydrochara
Onthophagus
Cetonia
Ocypus
Cyphon
Micromalthus
Cybister
Cicindela
Gyrinus
Aspidytes
Hydroscapha
Lepicerus
Cimex
1
1
0.98
1
1
1
0.98
0.91
1
1
0.94
1
0.98
1
1
1
skap
Thanasimus
Rhyzobius
Leptinotarsa
Anoplophora
Tribolium
Bitoma
Byturus
Ocypus
Cetonia
Hydrochara
Meloe
Agrilus
Onthophagus
Xylobiops
Dendroctonus
Aspidytes
Noterus
Cybister
Cicindela
Hydroscapha
Cyphon
Micromalthus
Lamprohiza
Lepicerus
Halyomorpha
0.97
1
0.9
0.96
0.99
1
1
1
1
1
Archostemata
Myxophaga
Adephaga
Elateriformia
Bostrichiformia
Staphyliniformia
Scarabaeiformia
Cucujiformia

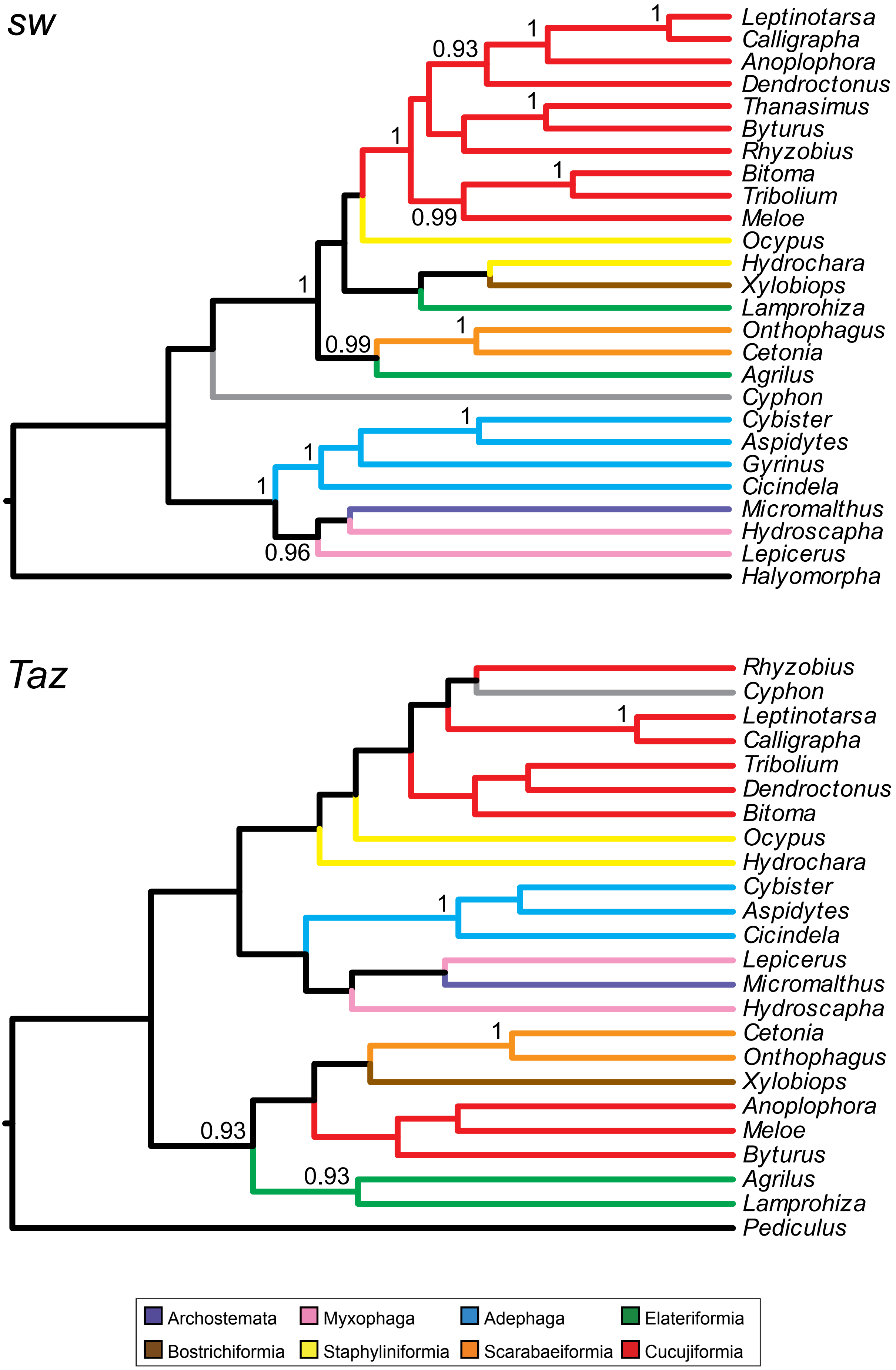

SW
Leptinotarsa
Calligrapha
Anoplophora
Dendroctonus
Thanasimus
Byturus
Rhyzobius
Bitoma
Tribolium
Meloe
Ocypus
Hydrochara
Xylobiops
Lamprohiza
Onthophagus
Cetonia
Agrilus
Cyphon
Cybister
Aspidytes
Gyrinus
Cicindela
Micromalthus
Hydroscapha
Lepicerus
Halyomorpha
1
1
0.93
1
1
1
0.99
1
0.99
1
1
1
1
0.96
Taz
Rhyzobius
Cyphon
Leptinotarsa
Calligrapha
Tribolium
Dendroctonus
Bitoma
Ocypus
Hydrochara
Cybister
Aspidytes
Cicindela
Lepicerus
Micromalthus
Hydroscapha
Cetonia
Onthophagus
Xylobiops
Anoplophora
Meloe
Byturus
Agrilus
Lamprohiza
Pediculus
1
1
1
0.93
0.93
Archostemata
Myxophaga
Adephaga
Elateriformia
Bostrichiformia
Staphyliniformia
Scarabaeiformia
Cucujiformia

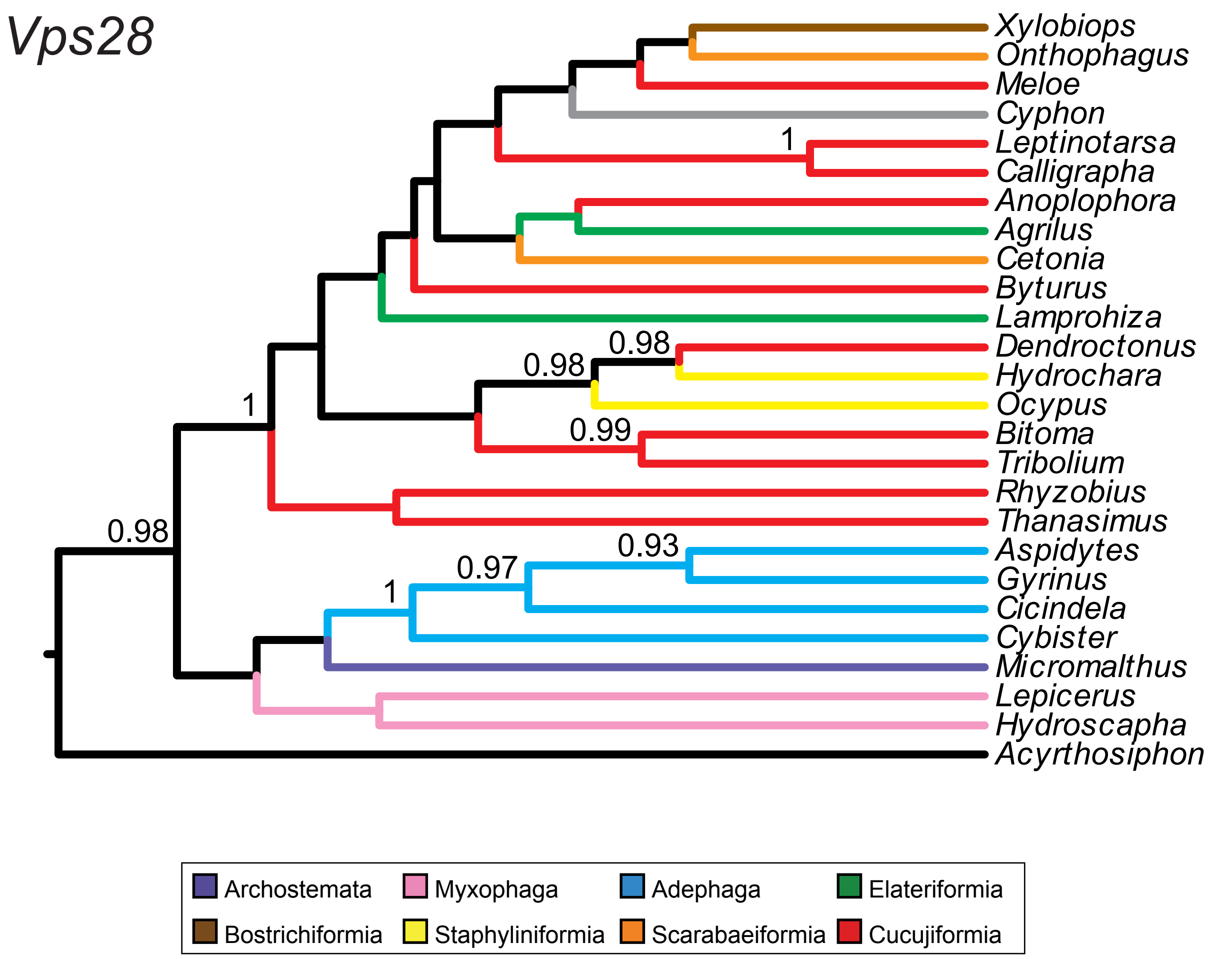
Vps28
Xylobiops
Onthophagus
Meloe
Cyphon
Leptinotarsa
Calligrapha
Anoplophora
Agrilus
Cetonia
Byturus
Lamprohiza
Dendroctonus
Hydrochara
Ocypus
Bitoma
Tribolium
Rhyzobius
Thanasimus
Aspidytes
Gyrinus
Cicindela
Cybister
Micromalthus
Lepicerus
Hydroscapha
Acyrthosiphon
1
0.98
0.98
0.99
1
0.98
0.93
0.97
1
Archostemata
Myxophaga
Adephaga
Elateriformia
Bostrichiformia
Staphyliniformia
Scarabaeiformia
Cucujiformia
